# Metal-Free C–C/C–N/C–C Bond Formation Cascade for the Synthesis of (Trifluoromethyl)sulfonylated Cyclopenta[*b*]indolines

**DOI:** 10.1021/acs.orglett.1c00557

**Published:** 2021-04-01

**Authors:** Carlos Lázaro-Milla, Hikaru Yanai, Pedro Almendros

**Affiliations:** † Grupo de Lactamas y Heterociclos Bioactivos, Departamento de Química Orgánica, Unidad Asociada al CSIC, Facultad de Química, Universidad Complutense de Madrid, 28040 Madrid, Spain; ‡ School of Pharmacy, Tokyo University of Pharmacy and Life Sciences, 1432-1 Horinouchi, Hachioji, Tokyo 192-0392, Japan; § Instituto de Química Orgánica General, IQOG, CSIC, Juan de la Cierva 3, 28006 Madrid, Spain

## Abstract

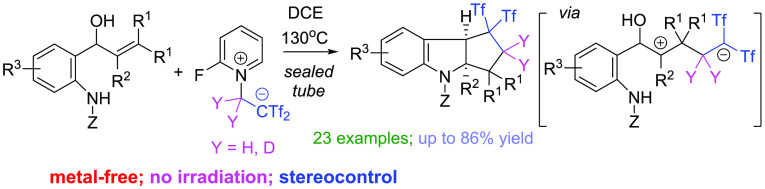

A bis(triflyl)ethylation [triflyl = (trifluoromethyl)sulfonyl] inserted into a sequential cyclization cascade resulted in the direct formation of *gem*-bis(triflyl)ated cyclopenta[*b*]indolines from anilide-derived allenols and alkenols. This catalyst- and irradiation-free sequence facilitated the efficient preparation of functionalized tricyclic indoline cores bearing two contiguous stereocenters. The formed cyclopenta[*b*]indolines can be easily transformed into a wide variety of triflylated indolines, including the tetracycle ring system found in polyveoline.

Cyclopenta[*b*]indole/indoline is a privileged scaffold present in various biologically active compounds. This structural motif is widely found in the molecular structures of natural indole alkaloids such as fischerindole L, yuehchukene, and polyveoline (Figure 1).^1^ Azacyclopenta[*b*]indolines, exemplified by physostigmine, also make up an important family of bioactive alkaloids.^2^ Synthetic efforts involving these polycyclic systems focus on the development of the dearomatization of indoles^3^ either by the electrophilic activation of indole substrates bearing nucleophilic sites (Scheme 1a)^4^ or by dearomative cycloadditions (Scheme 1b).^5^ The *de novo* synthesis of polycyclic indolines, chemical transformations of sophisticated aniline-derived substrates, despite being interesting, required expensive transition metal catalysts.^6^ Consequently, we were interested in the development of a novel cascade reaction to produce the polycyclic indolines from easily available aniline-derived substrate **A** with Tf_2_C=CH_2_ (Tf = SO_2_CF_3_), which exhibits outstanding high electrophilicity (Scheme 1c). In general, the domino reactions consisting of several bond formation steps without the isolation of intermediates are efficient, sustainable, and economically favorable processes in organic synthesis because they are associated with the reduction of reagents, solvents, and waste.^7^ Additionally, the reaction system presented here, including the sequential C–C/C–N/C–C bond-forming process, is certainly challenging from the following points of view: (1) realization of chemo- and regioselectivities in each reaction step and (2) the difficulty with C–C bond formation through intramolecular nucleophilic substitution by the [Tf_2_CR]^−^ moiety. The [Tf_2_CR]^−^ species is known to be a chemically inert carbanion owing to two triflyl groups on the anionic carbon atom,^8^ and C–C bond formation from this species is limited in specific cases mediated by highly reactive vinyl-type carbocation intermediates.^9^ Herein, we describe our methodology, applying high-energy species as a reaction partner that does not require the use of any catalyst/activator including photochemical activation. In addition, taking advantage of the chameleonic reactivity of sulfonyl compounds, the *gem*-bis(triflyl)ated indoline products were successfully derivatized into the highly functionalized indolines with fluorinated substituents, which may yield interesting properties.^10,11^

**Figure 1:**
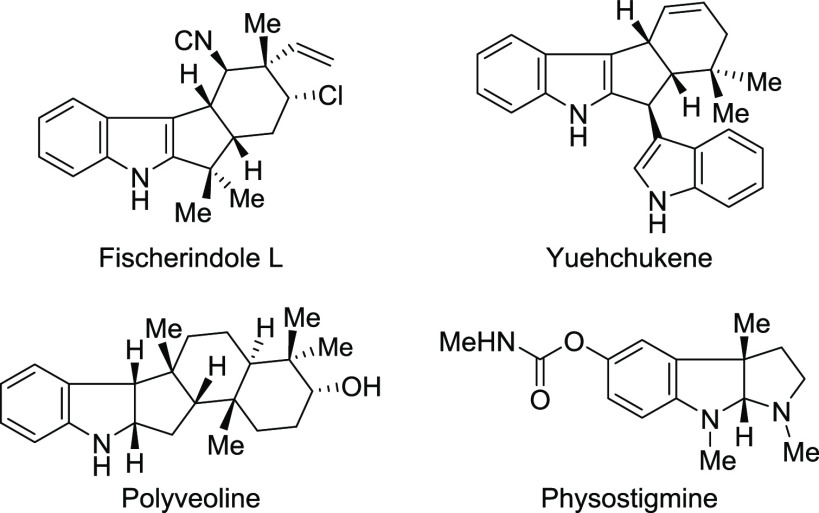
Bioactive and natural products having the polycyclic indoline core moiety.

**Scheme 1:**
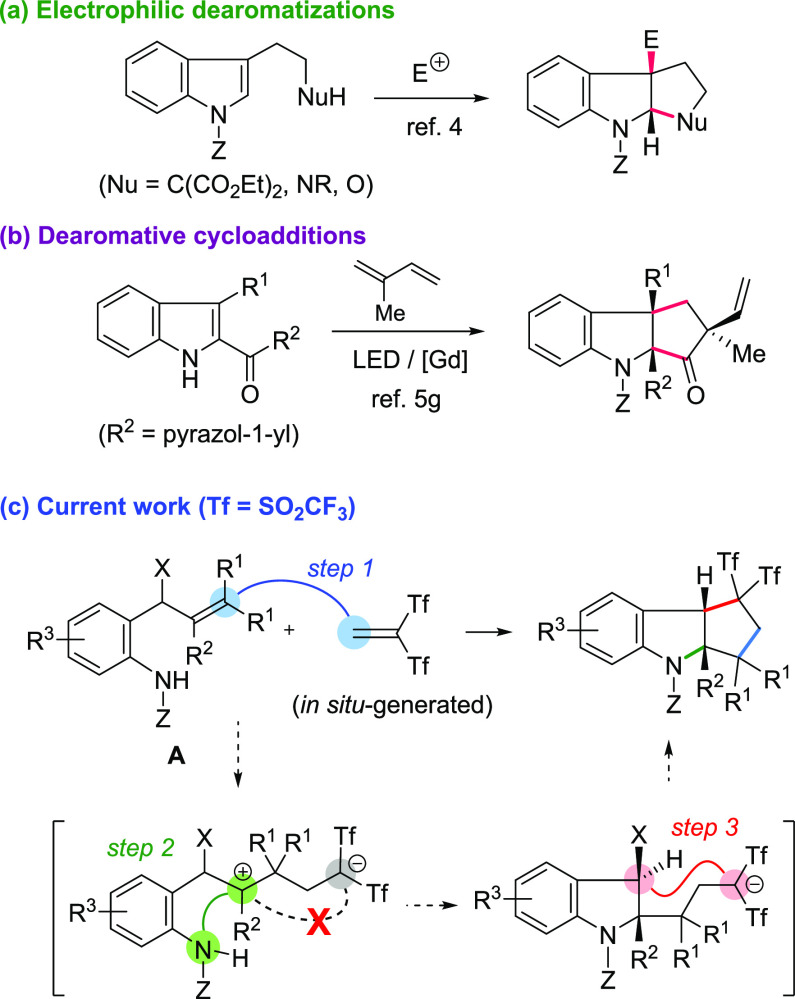
Background and Current Design for Cyclopenta[*b*]indolines

The project began from an unexpected reaction of aniline-derived substrate **2a** with 2-(2-fluoropyridinium-1-yl)-1,1-bis(triflyl)ethan-1-ide **1**, a reagent developed by Yanai et al. as an easily available, shelf-stable compound to serve as a latent source of Tf_2_C=CH_2_ (Scheme 2).^12^ We recently reported that the reaction of several allenols with Yanai’s reagent **1** smoothly proceeded in acetonitrile at room temperature to give bis(triflyl)enones through electrophilic attack of Tf_2_C=CH_2_ on the terminal carbon atom of the allene moiety.^13^ Surprisingly, when aniline-derived allenol **2a** was used as a reaction substrate, tricyclic indoline **3a** rather than bis(triflyl)enone **4** or bis(triflyl)ethylated anilide **5** was obtained as the major product (35% yield) along with several unidentified compounds. The structure of **3a** was proven through its X-ray crystallographic analysis.^14^ After carefully exploring several reaction solvents and temperatures, we concluded that the use of 1,2-dichloroethane (DCE) at 130 °C was the optimal reaction condition. In this reaction, use of bench grade solvents did not affect the reaction outcome. Fused indoline **3a** was obtained as a single *cis* isomer in a reasonable 62% yield.^15,16^ We concluded that a carbonyl moiety on the nitrogen was necessary as benzyl- or sulfonyl-protected allenyl anilines **2** decomposed upon reaction with **1**.

**Scheme 2:**
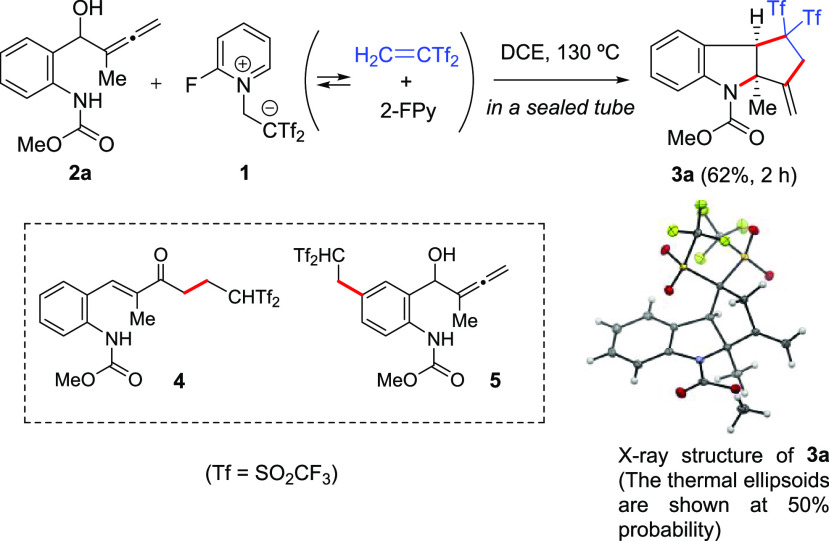
Reaction of Carbamate-Derived Substrate **2a**

The scope of this transformation is summarized in Figure 2. Introducing substituents on the allene moiety by replacing the *C*-methyl group with *C*-phenyl and *C*-aryl groups with different electronic and steric characteristics gave the product indoline (**3e–3g**) in fair yields and total selectivity. Pleasingly, when halogen (I, Br, and Cl), alkyl (Me), or alkoxy (MeO) substituents were incorporated into the aromatic ring of the anilide moiety, the products (**3i–3o**) were obtained in 40–68% yields. The NMR spectra of tricycles **3d** and **3o** showed signals of a minor isomer (8% and 12%, respectively, as estimated by ^1^H NMR). As only *cis*-fused 5,5-systems are typically observed in any reaction outcome, the *trans* isomer should be ruled out and these signals should be ascribed to the corresponding rotamers.^17^ Interestingly, tetracyclic indoline **3h** bearing an extra fused benzene ring was formed in a good 77% yield as a single isomer. Similarly, deuterated *gem*-bis(triflyl)indoline [D]-**3a** was smoothly formed through the reaction between aniline-derived allenol **2a** and reagent [D]-**1**. The tricycle structure of **3e** and its relative stereochemistry were proved through X-ray crystallographic analysis.^14^

**Figure 2:**
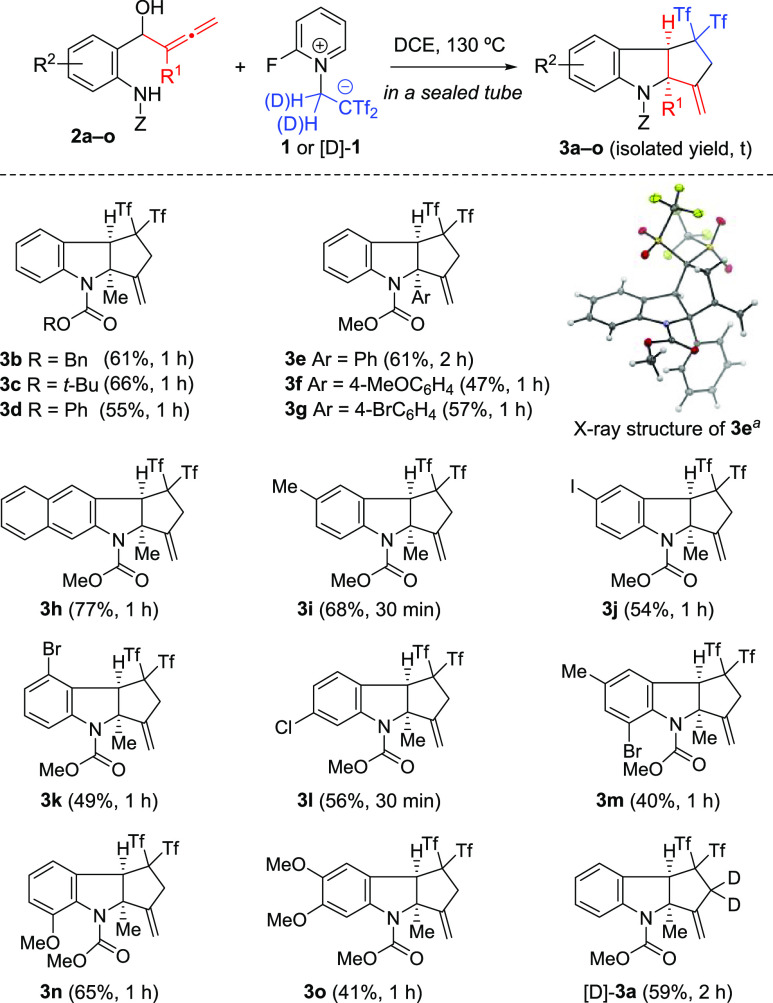
Synthesis of bis(triflyl)-containing tricyclic indolines **3a–3o** and [D]-**3a**. ^*a*^The thermal ellipsoids are shown at 50% probability.

Motivated by the results presented above, we decided to expand the substrate scope by exploring other precursors in place of anilide-derived allenols **2**. It is noteworthy that when anilide **6a** having an electronically unbiased allylic alcohol was exposed to the standard conditions, desired indoline **7a** was obtained in 64% yield (Figure 3). The position and electronic nature of the substituents on the arene core of **6** do not seem to have a decisive influence on the transformation and provided tricyclic *gem*-bis(triflyl)indolines **7** in a competent way. In addition, chloro substitutions in anilide precursors are well accommodated, which brings about the possibility of postfunctionalization. Sterically bulky substituents, such as the phenyl group in allene precursor **6d**, attenuated neither reactivity nor selectivity. However, the diminished yield of tricyclic product **7c**, having an acetate instead a carbamate group, showed the pivotal role of the protecting group. Structures of compounds **7d** and **7g** were determined unambiguously by single-crystal X-ray diffraction analysis.^14^

**Figure 3:**
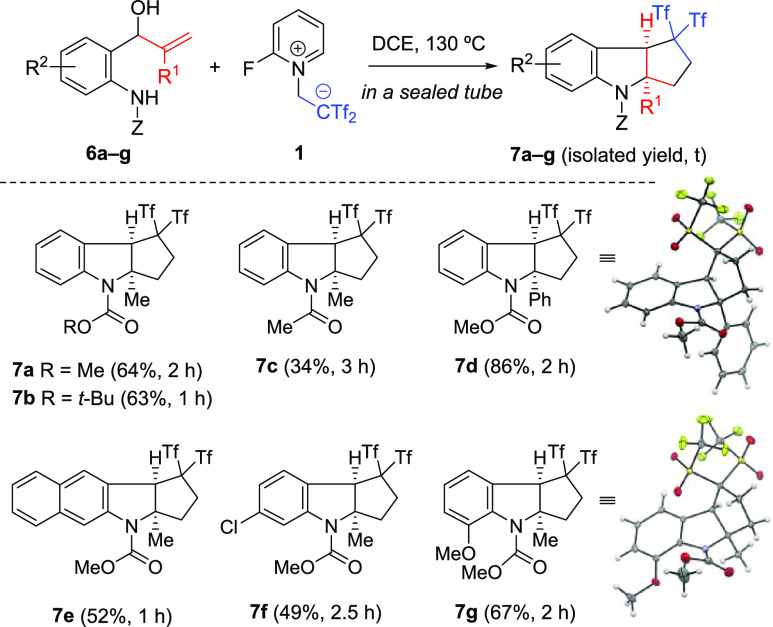
Synthesis of bis(triflyl)-containing tricyclic indolines **7a–7g**. The thermal ellipsoids are shown at 50% probability.

To showcase the applicability of the protocol, tricyclic indolines **3a–3c** and **7a** were subjected to further synthetic transformations (Scheme 3). As depicted in Scheme 3a, the derivatization of indoline **3a** under basic conditions resulted in dienyl triflone **8**, while bromination afforded product **9**. The facile transformation of the benzylic position in tricycles **3** and **7** enabled the formation of bis(triflyl)ethyl-decorated bicyclic indolines **10**, **11**, **13**, and **19** under reductive conditions (Scheme 3b,c,f).^18^ Likewise, *N*-deprotected indoline **12** could easily be obtained by acid treatment of *N*-Boc indoline **3c** (Scheme 3c). On the contrary, the nucleophilic 1,4-addition of amines and thiols to conjugate diene **8** occurred in one pot from **3a** to give the functionalized derivatives **14** and **15** (Scheme 3d). Treatment of iodoindoline **3j** under Suzuki–Miyaura conditions resulted in cross-coupled adduct **16** in which detriflylation also occurred, while conjugate diene **8** proved to be an excellent dienophile in the Diels–Alder reaction with 2,3-dimethylbuta-1,3-diene to form **17** stereoselectively (Scheme 3e). Compound **17** bears the tetracyclic core of indole sesquiterpene polyveoline (Figure 1). Though functionalized polycycles **15** and **17** can be directly accessed from **3a**, higher yields were observed when dienyl triflone **8** was the immediate precursor. Finally, elimination of CF_3_SO_2_H can be smoothly accomplished in **7a** to give alkenyl triflone **18** after treatment with potassium carbonate (Scheme 3f). In contrast with indolines **3**, the absence of the terminal alkene moiety in **7a** directs the elimination toward the formation of the more substituted alkene.

**Scheme 3:**
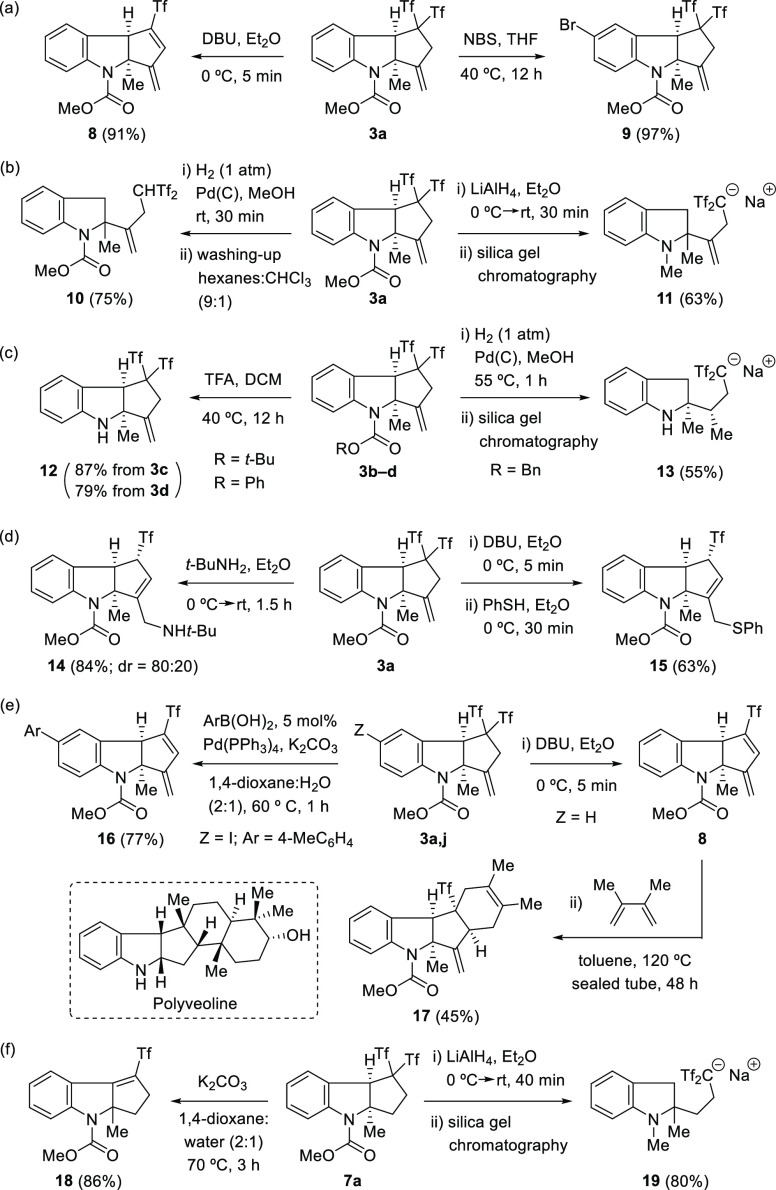
Synthetic Transformations of *gem*-Bis(triflyl)indolines **3** and **7**

The mechanistic hypothesis for the formation of **3** and **7** is depicted in Scheme 4. First, electrophilic attack of Tf_2_C=CH_2_ (generated *in situ* along with 2-fluoropyridine from betaine **1**) proceeds with **2** or **6** on the β-carbon atom of the alkenol (or allenol) moiety to afford putative zwitterionic intermediate **INT-1**. Thereafter, key bicyclic intermediate **INT-2** was formed by cyclization with the amide nitrogen, which after proton release and further protonation forms species **INT-3**. Oxonium intermediate **INT-3** suffers a dehydration to generate 2*H*-indol-1-ium **INT-4**. Finally, **INT-4** can react in an intramolecular fashion through an ionic carbocyclization to deliver the required *gem*-bis(triflyl)indolines **3** and **7**. The 2-fluoropyridine liberated in the medium should facilitate the protonation and deprotonation steps. This reaction pathway was supported by DFT simulation of the reaction of allenol **2a** with Tf_2_C=CH_2_ at the PCM(DCE)-M06-2X/6-31+G(d) level of theory (for details, see the Supporting Information).^19^ For the first C–C bond-forming step, 23.1 kcal mol^–1^ of the activation barrier was obtained and carbocation **INT-1** was found as the reaction intermediate. The very low barrier (1.5 kcal mol^–1^) of the following C–N bond-forming step implies that this process rapidly proceeds to give **INT-2**. Although the *cis*-fused tricyclic indolines were selectively obtained in the experiment, this stereochemical outcome can be attributed to the kinetically favorable approach of the anionic carbon atom to the C1 atom from the less hindered *cis* site in the last step (**INT-4** → **3a**).

**Scheme 4:**
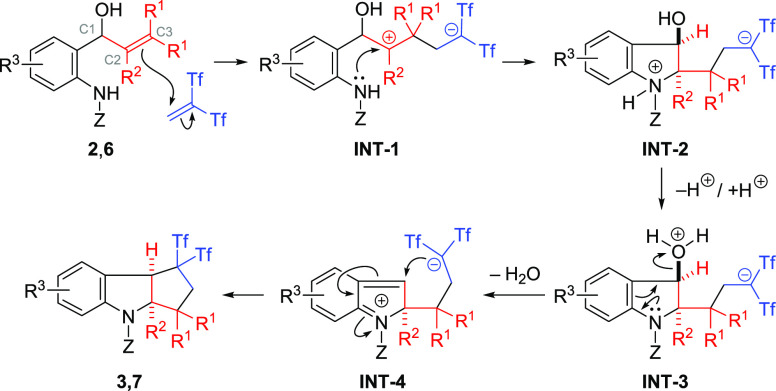
Tentative Pathway for the Formation of *gem*-Bis(triflyl)indolines **3** and **7**

In summary, tricyclic *gem*-bis(triflyl)indolines have been selectively formed by reaction of easily preparable anilide-derived substrates with Tf_2_C=CH_2_ without catalysts or light irradiation. The cascade reaction presented here for the formation of one C–N bond and two C–C bonds is facilitated by initial intermolecular electrophilic attack of Tf_2_C=CH_2_ on the double bond,^20^ which is followed by intramolecular capture (azacyclization) of the carbocation intermediate and subsequent carbocyclization of the resulting carbanion. This method provides interesting tricyclic indolines bearing the triflyl group, which can endow the nonfluorinated derivatives with interesting properties. The chameleonic reactivity of the triflyl group allowed us to derivatize the indolines toward less accessible fluorinated polycyclic heterocycles, including the tetracycle core found in the alkaloid polyveoline.

## Supporting Information Available

Experimental procedures, characterization data of new compounds, copies of NMR spectra, crystallographic data, and computational details (PDF)

## Accession Codes

CCDC 2056816–2056819 contain the supplementary crystallographic data for this paper. These data can be obtained free of charge via www.ccdc.cam.ac.uk/data_request/cif, or by emailing data_request@ccdc.cam.ac.uk, or by contacting The Cambridge Crystallographic Data Centre, 12 Union Road, Cambridge CB2 1EZ, UK; fax: +44 1223 336033.
