# Supplementary material for: Metal-Free C–C/C–N/C–C Bond Formation Cascade for the Synthesis of (Trifluoromethyl)sulfonylated Cyclopenta[b]indolines
Source: Org Lett. 2021 Apr 1;23(8):2921–6. doi: 10.1021/acs.orglett.1c00557 (PMC8479863; doi:10.1021/acs.orglett.1c00557)
Supplement: Supplementary file 1 — ol1c00557_si_001.pdf [file ol1c00557_si_001.pdf]

Supplementary Information for the Paper

Metal-Free C–C/C–N/C–C Bonds Formation Cascade for the Synthesis of (Trifluoromethyl)sulfonylated Cyclopenta[b]indolines

Carlos Lázaro-Milla,[†] Hikaru Yanai,[‡] and Pedro Almendros*,[§]

[†]*Grupo de Lactamas y Heterociclos Bioactivos, Departamento de Química Orgánica, Unidad Asociada al CSIC, Facultad de Química, Universidad Complutense de Madrid, 28040-Madrid, Spain*

[§]*Instituto de Química Orgánica General, IQOG-CSIC, Juan de la Cierva 3, 28006-Madrid, Spain*

E-mail: palmendros@iqog.csic.es

[‡]*School of Pharmacy, Tokyo University of Pharmacy and Life Sciences, 1432-1 Horinouchi, Hachioji, Tokyo 192-0392, Japan*

Table of Contents

Figure S1	2
General Methods	2
Experimental Section	3–52
^{1}H NMR, ^{13}C NMR, ^{19}F NMR, and D(^{2}H) NMR Spectra	53–227
Computational Details	228–244
Crystallographic Data	245–252

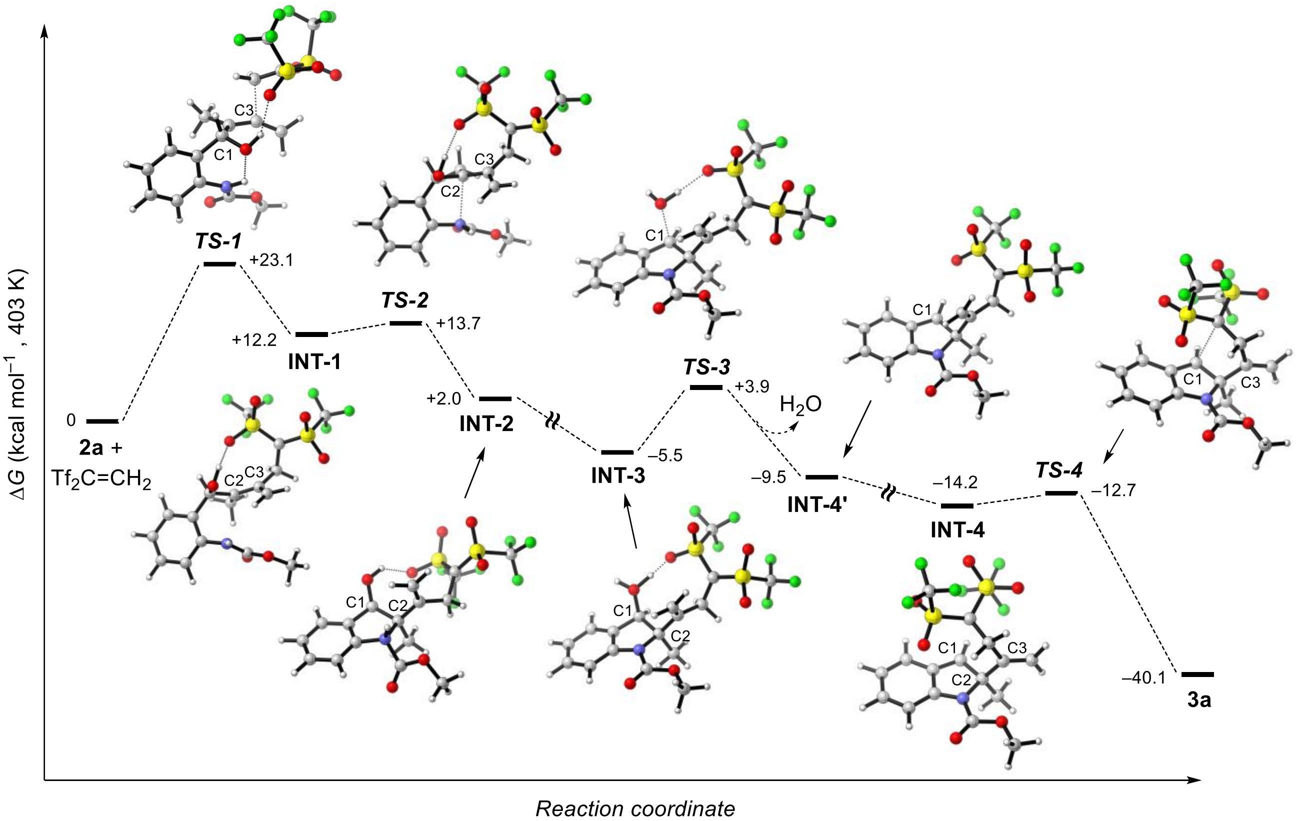

Figure S1. Reaction profile for the reaction of **2a** with $Tf_2C{=}CH_2$ (403.15 K)

General Methods: ^{1}H NMR, ^{13}C NMR, ^{19}F NMR, and D(^{2}H) NMR spectra were recorded on a Bruker Avance AMX-700, Bruker AMX-500, or Bruker Avance-DPX 300. NMR spectra were recorded in $CDCl_3$ or acetone-d_6 solutions, except otherwise stated. Chemical shifts are given in ppm relative to TMS (^{1}H, 0.0 ppm), or $CDCl_3$ (^{1}H, 7.27 ppm; ^{13}C, 76.9 ppm), or acetone-d_6 (^{1}H, 2.05 ppm; ^{13}C, 206.3 ppm) or 1,1,2,2-tetrachloroethane-d_2 (^{1}H, 6.00 ppm). Chemical shifts in ^{19}F are given in ppm relative to (trifluoromethyl)benzene ($C_6H_5CF_3$) in $CDCl_3$ (^{19}F, –63.7 ppm). Low and high resolution mass spectra were taken on an AGILENT 6520 Accurate-Mass QTOF LC/MS spectrometer using the electronic impact (EI) or electrospray modes (ES) unless otherwise stated. IR spectra were recorded on a Bruker Tensor 27 spectrometer. All commercially available compounds were used without further purification. Microwave irradiation was carried out in a Monowave 300 from Anton Paar GmbH. The reaction temperatures during microwave heating were measured with

an internal infrared sensor. Column chromatography was carried out using silica gel 60, 0.04-0.06 mm, for flash chromatography (230-400 mesh ASTM) provided by Scharlau. For reactions that require heating, a heating-on block was used.

Yanai's reagent **1** was synthesized according to a literature procedure: H. Yanai, Y. Takahashi, H. Fukaya, Y. Dobashi, T. Matsumoto, *Chem. Commun.* **2013**, *49*, 10091. Deuterated Yanai's reagent [D]-**1** was prepared adapting the same procedure (B. Alcaide, P. Almendros, C. Lázaro-Milla, *Chem. Eur. J.* **2019**, *25*, 7547).

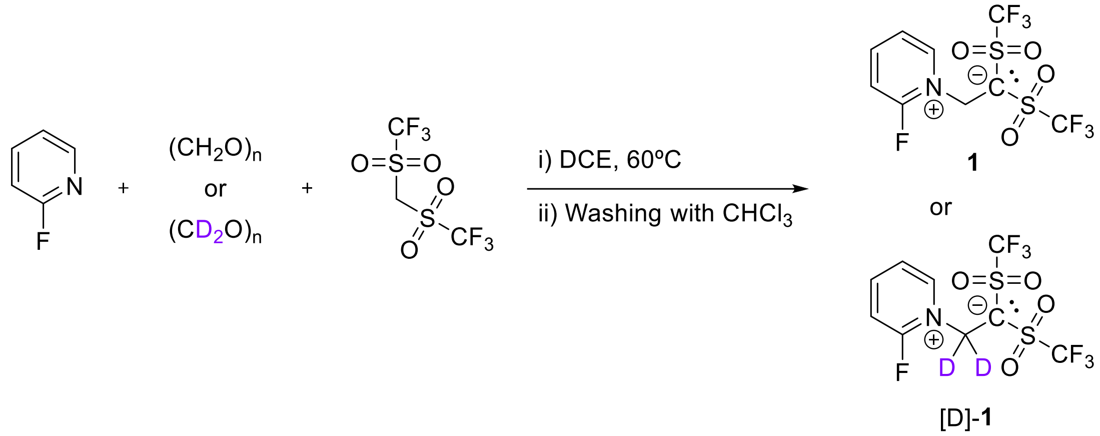

To a solution of Tf_2CH_2 (281 mg, 1.00 mmol) in 1,2-dichloroethane (6.0 mL), paraformaldehyde (90% purity, 73.0 mg, 2.19 mmol) or paraformaldehyde-d_2 (98% purity, 98 atom % D, 64 mg, 2.00 mmol) and 2-fluoropyridine (172 µL, 2.00 mmol) were added at room temperature. After being stirred for 8 h at 60 °C, the reaction mixture was concentrated under reduced pressure. The resulting residue was washed with $CHCl_3$ (1.0 mL x 3) to give zwitterion **1** in 91% yield (356 mg, 0.915 mmol) or [D]-**1** in 86% yield (336 mg, 0.858 mmol).

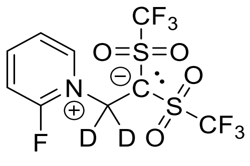

[D]-**1**

Deuterated Yanais'reagent [D]-**1.** From 281 mg (1.0 mmol) of CH_2Tf_2, 336 mg (86%) of compound [D]-**1** was obtained as a colorless solid; mp 161–163 ºC; ^1H NMR (700 MHz, CD_3CN, 25 ºC): δ = 8.99 (s, 1H, CH^{Ar}), 8.64 (m, 1H, CH^{Ar}), 7.95 (m, 1H, CH^{Ar}), 7.78 (m, 1, CH^{Ar}); ^{13}C NMR (175 MHz, CD_3CN, 25 ºC): δ = 158.7 (d, J_{CF} = 278.8 Hz, $C^{Ar\text{-}q}$-F), 151.1 (d, J_{CF} = 9.9 Hz, CH^{Ar}), 142.0 (CH^{Ar}), 124.4 (CH^{Ar}), 120.7 (q, J_{CF} = 325.4 Hz, $2CF_3$), 114.3 (d, J_{CF} = 21.3 Hz, CH^{Ar}), 67.1 (CTf_2-*broad*), 56.6 (CD_2- *broad*); ^{19}F NMR (282 MHz, CD_3CN, 25 ºC): δ = –79.6 (s, 1F, F), –80.5 (s, 6F, $2CF_3$); D(^2H) NMR (107 MHz, CD_3CN, 25 ºC): δ = 5.63 (s, 2D, CD_2); IR (KBr): ν = 1345, 1101 (O=S=O), 1192 (C–F) cm^{-1}.

Novel allenols 2a–o and alkenols 6a–g were prepared as follow:

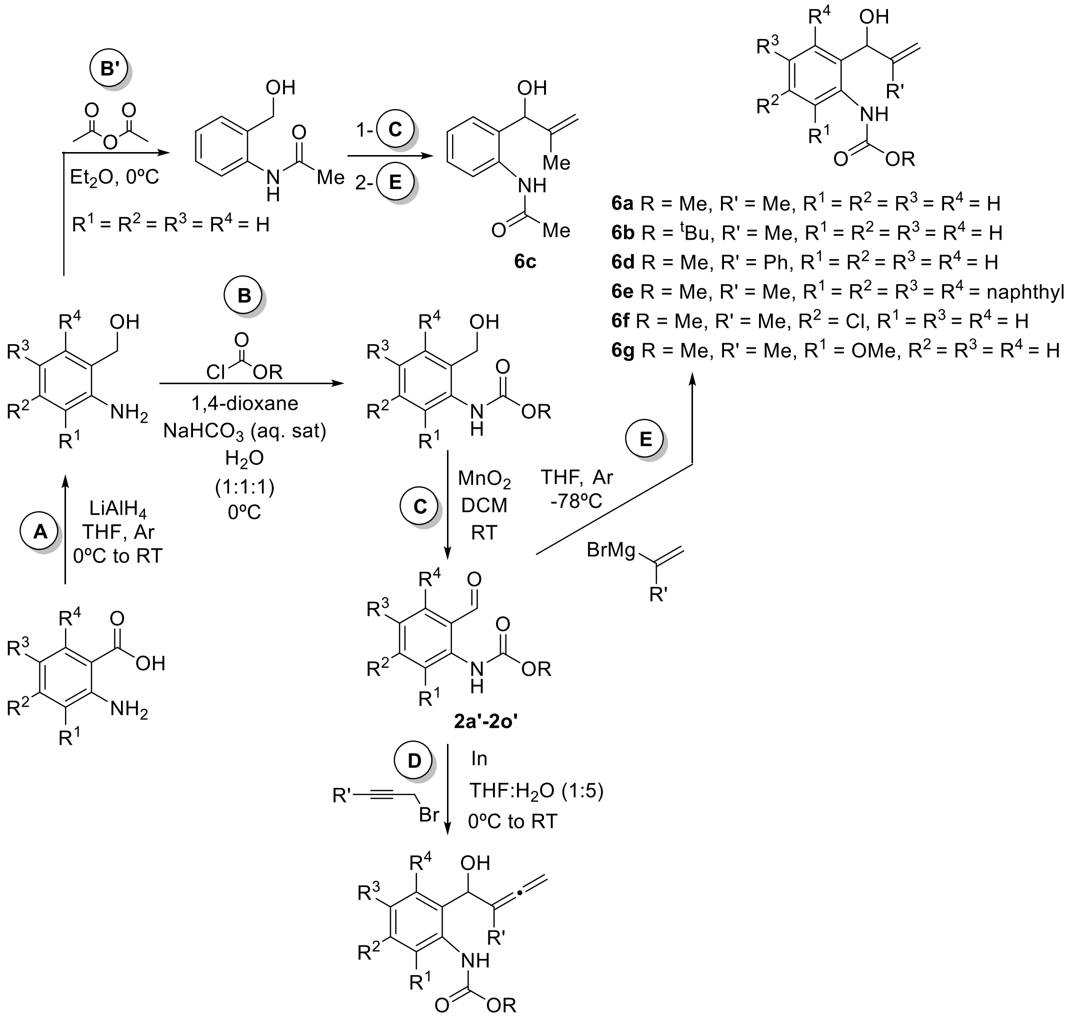

2a R = Me, R' = Me, $R^1 = R^2 = R^3 = R^4 = H$
2b R = Bn, R' = Me, $R^1 = R^2 = R^3 = R^4 = H$
2c R = ^{t}Bu, R' = Me, $R^1 =R^2 = R^3 = R^4 = H$
2d R = Ph, R' = Me, $R^1 = R^2 = R^3 = R^4 = H$
2e R = Me, R' = Ph, $R^1 = R^2 = R^3 = R^4 = H$
2f R = Me, R' = 4-MeOC$_6$H$_4$, $R^1 = R^2 = R^3 = R^4 = H$
2g R = Me, R' = 4-BrC$_6$H$_4$, $R^1 = R^2 = R^3 = R^4 = H$
2h R = Me, R' = Me, $R^1 = R^2 = R^3 = R^4$ = naphthyl
2i R = Me, R' = Me, R^3 = Me, $R^1 = R^2 = R^4 = H$
2j R = Me, R' = Me, R^3 = I, $R^1 = R^2 = R^4 = H$
2k R = Me, R' = Me, R^4 = Br, $R^1 = R^2 = R^3 = H$
2l R = Me, R' = Me, R^2 = Cl, $R^1 = R^3 = R^4 = H$
2m R = Me, R' = Me, R^1 = Br, R^3 = Me, $R^2 =R^4 = H$
2n R = Me, R' = Me, R^1 = OMe, $R^2 = R^3 = R^4 = H$
2o R = Me, R' = Me, $R^2 = R^3$ = OMe, $R^1 = R^4 = H$

Step A: To a suspension of $LiAlH_4$ (2.5 mmol) in dry THF (2 mL), cooled to 0°C under argon, was added the corresponding carboxylic acid (1.0 mmol) in portions and then stirred at room temperature until complete conversion (product monitored by TLC). The reaction was carefully quenched with H_2O, NaOH (15% aq.), at 0 °C, and then stirred at room temperature for 30 min. The resulting

mixture was filtered through a pad of celite and extracted with AcOEt, dried over $MgSO_4$ and concentrated to afford a crude product, which was used directly in the next reaction.

Step B: To a stirring solution of the appropriate aminobenzyl alcohol (1.0 mmol) in 0.6 mL of dioxane, 0.6 mL of saturated $NaHCO_3$ solution, and 0.6 mL of water at 0 °C was added the corresponding chloroformate (1.2 mmol) dropwise. The resulting mixture was stirred at room temperature. After complete conversion the reaction was diluted with brine and extracted with AcOEt. The organic layers were dried over $MgSO_4$ and concentrated under reduced pressure to afford a crude product, which was used directly in the next reaction (Procedure described in: P. Y. Chong, S. Z. Janicki, P. A. Petillo, *J. Org. Chem.* **1998**, *63*, 85153).

Step B': To a stirring solution of 2-aminobenzyl alcohol (1.0 mmol) in Et_2O (2 mL) at 0 ºC was added Ac_2O (3.0 mmol) dropwise. After 5 min stirring at the same temperature, the precipitate was collected by filtration and dry under reduced pressure. The product was used directly in the next reaction (Procedure described in: K. Kobayashi, N. Matsumoto, *Helv. Chim. Acta* **2014**, *97*, 923).

Step C: The appropriate *N*-carbamate alcohol (1.0 mmol) was dissolved in DCM (20 mL) and activated MnO_2 (15 mmol) was added to the solution. The suspension was stirred at rt after complete conversion (product monitored by TLC). Then, the solution was filtered through a pad of celite, and the filtrate was concentrated to afford the crude mixture. Purification by flash column chromatography on silica gel gave aldehydes **2a'–2o'**.

Aldehydes 2a'–2c', 2i', 2l' and 2n' were prepared as described in the literature: 2a' (X. Wen, Y. Wang, X. P. Zhang, *Chem. Sci.* **2018**, *9*, 5082); **2b'** (Y.-T. Lee, Y.-J. Jang, S.-e. Syu, S.-C. Chou, C.-J. Lee, W. Lin, *Chem. Commun.* **2012**, *48*, 8135); **2c'** (I. Muthukrishnan, M. Karuppasamy, B. S. Vachan, D. Rajput, N. Subbiah, C. U. Maheswari, V. Sridharan, *Org. Chem. Front.* **2020**, *7*, 1616); **2i'** (L. A. Leth, F. Glaus, M. Meazza, L. Fu, M. K. Thøgersen, E. A. Bitsch, K. A. Jørgensen, *Angew. Chem. Int. Ed.* **2016**, *55*, 15272); **2l'** (R. T. Sawant, M. Y. Stevens, L. R. Odell, *ACS Omega* **2018**,

3, 14258); **2n'** (M. Y. Stevens, K. Wieckowski, P. Wu, R. T. Sawanta, L. R. Odell, *Org. Biomol. Chem.* **2015**, *13*, 2044).

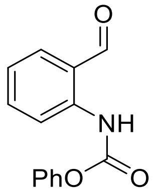

2d'

Aldehyde 2d'. From 987 mg (4.06 mmol) of the corresponding alcohol, and after flash chromatography of the residue using hexanes/ethyl acetate (9:1) as eluent gave compound **2d'** (778 mg, 79%) as a colorless solid; mp 88–90 ºC; ^{1}H NMR (300 MHz, $CDCl_3$, 25 ºC): δ = 10.90 (s, 1H, NH), 9.89 (d, 1H, *J* = 0.5 Hz, CHO), 8.40 (d, 1H, *J* = 8.5 Hz, CH^{Ar}), 7.62 (dd, 1H, *J* = 7.6, 1.6 Hz, CH^{Ar}), 7.55 (m, 1H, CH^{Ar}), 7.33 (m, 2H, $2CH^{Ar}$), 7.17 (m, 4H, $4CH^{Ar}$); ^{13}C NMR (75 MHz, $CDCl_3$, 25 ºC): δ = 195.2 (HC=O), 151.9 (C=O), 150.4 ($C^{Ar\text{-}q}$), 140.7 ($C^{Ar\text{-}q}$), 136.1 (CH^{Ar}), 136.0 (CH^{Ar}), 129.4 ($2CH^{Ar}$), 125.8 (CH^{Ar}), 122.5 (CH^{Ar}), 121.6 ($2CH^{Ar}$), 118.5 (CH^{Ar}); IR ($CHCl_3$): ν = 3278 (NH), 1714 (C=O), 1668 (C=O) cm^{-1}. HRMS (ESI) m/z: $[M + H]^+$ Calcd for $C_{14}H_{12}NO_3$ 242.0812; Found 242.0817.

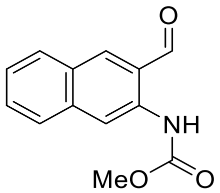

2h'

Aldehyde 2h'. From 264 mg (1.14 mmol) of the corresponding alcohol, and after flash chromatography of the residue using hexanes/ethyl acetate (9:1) as eluent gave compound **2h'** (209 mg, 80%) as a yellow solid; mp 125–127 ºC; ^{1}H NMR (300 MHz, $CDCl_3$, 25 ºC): δ = 10.40 (s, 1H, NH), 10.05 (d, 1H, *J* = 0.6 Hz, CHO), 8.77 (s, 1H, CH^{Ar}), 8.17 (s, 1H, CH^{Ar}), 7.85 (m, 2H, $2CH^{Ar}$), 7.61 (m, 1H, CH^{Ar}), 7.44 (m, 1H, CH^{Ar}), 3.84 (s, 3H, OCH_3); ^{13}C NMR (75 MHz, $CDCl_3$, 25 ºC): δ = 195.0 (HC=O), 154.3 (C=O), 140.1 (CH^{Ar}), 137.1 ($C^{Ar\text{-}q}$), 135.8 ($C^{Ar\text{-}q}$), 130.3 (CH^{Ar}), 128.9

(CH^{Ar}), 128.2 ($C^{Ar\text{-}q}$), 127.7 (CH^{Ar}), 125.4 (CH^{Ar}), 122.6 ($C^{Ar\text{-}q}$), 115.2 (CH^{Ar}), 52.4 (OCH_3); IR ($CHCl_3$): ν = 1729 (C=O), 1654 (C=O) cm^{-1}; HRMS (ESI) m/z: $[M + H]^+$ Calcd for $C_{13}H_{12}NO_3$ 230.0812; Found 230.0811.

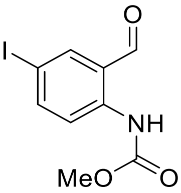

2j'

Aldehyde 2j'. From 614 mg (2.0 mmol) of the corresponding alcohol, and after flash chromatography of the residue using hexanes/ethyl acetate (9:1) as eluent gave compound **2j'** (228 mg, 37%) as a colorless solid; mp 126–128 ºC; ^1H NMR (300 MHz, $CDCl_3$, 25 ºC): δ = 10.51 (s, 1H, NH), 9.82 (d, 1H, *J* = 0.6 Hz, CHO), 8.27 (d, 1H, *J* = 8.9 Hz, CH^{Ar}), 7.92 (d, 1H, *J* = 2.1 Hz, CH^{Ar}), 7.85 (ddd, 1H, *J* = 8.9, 2.2, 0.4 Hz, CH^{Ar}), 3.81 (s, 3H, OCH_3); ^{13}C NMR (75 MHz, $CDCl_3$, 25 ºC): δ = 193.7 (HC=O), 153.9 (C=O), 144.4 (CH^{Ar}), 144.0 (CH^{Ar}), 140.8 ($C^{Ar\text{-}q}$), 123.0 ($C^{Ar\text{-}q}$), 120.4 (CH^{Ar}), 83.5 ($C^{Ar\text{-}q}$), 52.6 (OCH_3); IR ($CHCl_3$): ν = 1732 (C=O), 1656 (C=O) cm^{-1}. HRMS (ESI) m/z: $[M + H]^+$ Calcd for $C_9H_9INO_3$ 305.9622; Found 305.9625.

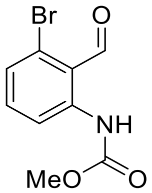

2k'

Aldehyde 2k'. From 512 mg (1.96 mmol) of the corresponding alcohol, and after flash chromatography of the residue using hexanes/ethyl acetate (95:5) as eluent gave compound **2k'** (259 mg, 57%) as a colorless solid; mp 122–124 ºC; ^1H NMR (300 MHz, $CDCl_3$, 25 ºC): δ = 11.10 (s, 1H, NH), 10.47 (d, 1H, *J* = 0.7 Hz, CHO), 8.46 (m, 1H, CH^{Ar}), 7.39 (m, 1H, CH^{Ar}), 7.31 (dd, 1H, *J* = 7.9, 1.2 Hz, CH^{Ar}), 3.81 (s, 3H, OCH_3); ^{13}C NMR (75 MHz, $CDCl_3$, 25 ºC): δ = 196.4 (HC=O), 153.9 (C=O), 143.5 ($C^{Ar\text{-}q}$), 136.6 (CH^{Ar}), 129.7 ($C^{Ar\text{-}q}$), 127.2 (CH^{Ar}), 118.2 (CH^{Ar}), 117.7 ($C^{Ar\text{-}q}$),

52.6 (OCH_3); IR ($CHCl_3$): ν = 3277(NH), 1731 (C=O), 1522 (C=O) cm^{-1}. HRMS (ESI) m/z: $[M + H]^+$ Calcd for $C_9H_9BrNO_3$ 257.9760; Found 257.9745.

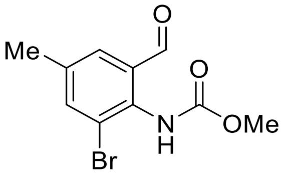

2m'

Aldehyde 2m'. From 759 mg (2.76 mmol) of the corresponding alcohol, and after flash chromatography of the residue using hexanes/ethyl acetate (9:1 → 8:2) as eluent gave compound **2m'** (492 mg, 65%) as a colorless solid; mp 137–139 ºC; ^{1}H NMR (300 MHz, $CDCl_3$, 25 ºC): δ = 9.99 (s, 1H, CHO), 7.65 (m, 2H, $2CH^{Ar}$), 7.04 (s, 1H, NH), 3.80 (s, 3H, OCH_3), 2.39 (s, 3H, CH_3); ^{13}C NMR (75 MHz, $CDCl_3$, 25 ºC): δ = 189.4 (HC=O), 155.3 (C=O), 138.2 (CH^{Ar}), 137.8 (C^{Ar-q}), 134.4 (C^{Ar-q}), 131.5 (C^{Ar-q}), 129.5 (CH^{Ar}), 120.3 (C^{Ar-q}), 53.3 (OCH_3), 20.5 (CH_3); IR ($CHCl_3$): ν = 2975 (NH), 1737 (C=O), 1660 (C=O) cm^{-1}. HRMS (ESI) m/z: $[M + H]^+$ Calcd for $C_{10}H_{11}BrNO_3$ 271.9917; Found 271.9921.

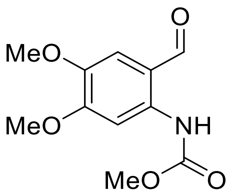

2o'

Aldehyde 2o'. From 1.2 g (4.97 mmol) of the corresponding alcohol, and after flash chromatography of the residue using hexanes/ethyl acetate (8:2) as eluent gave compound **2o'** (689 mg, 58%) as a colorless solid; mp 148–150 ºC; ^{1}H NMR (300 MHz, $CDCl_3$, 25 ºC): δ = 10.76 (s, 1H, NH), 9.69 (s, 1H, CHO), 8.10 (s, 1H, CH^{Ar}), 6.99 (s, 1H, CH^{Ar}), 3.96 (s, 3H, OCH_3), 3.87 (s, 3H, OCH_3), 3.76 (s, 3H, OCH_3); ^{13}C NMR (75 MHz, $CDCl_3$, 25 ºC): δ = 192.7 (HC=O), 155.5 (C=O), 154.3 (C^{Ar-q}), 143.8 (C^{Ar-q}), 137.7 (C^{Ar-q}), 116.3 (CH^{Ar}), 114.0 (CH^{Ar}), 101.3 (C^{Ar-q}), 56.2 (OCH_3), 56.1 (OCH_3),

52.2 (OCH_3); IR ($CHCl_3$): ν = 2983 (NH), 1728 (C=O), 1585 (C=O) cm^{-1}. HRMS (ESI) m/z: $[M + H]^+$ Calcd for $C_{11}H_{14}NO_5$ 240.0866; Found 240.0863.

Step D: The appropriate bromobutyne (3.0 mmol) was added to a well stirred suspension of the corresponding aldehyde **2'** (1.0 mmol) and indium powder (6.0 mmol) in THF/NH_4Cl (aq. sat.) (1:5, 5 mL) at 0 ºC. The resulting mixture was allowed to warm slowly to room temperature. After disappearance of the starting material (TLC) the mixture was extracted with ethyl acetate. The organic extract was washed with brine, dried over $MgSO_4$ and concentrated under reduced pressure. Chromatography of the residue gave allenols **2a–2o** (Procedure described in: B. Alcaide, P. Almendros, T. Martínez del Campo, R. Carrascosa, *Chem. Asian J.* **2008**, *3*, 1140).

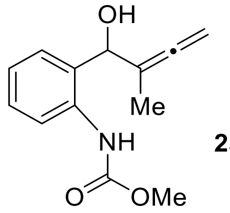

2a

Allenol 2a. From 150 mg (1.14 mmol) of the corresponding aldehyde **2'**, and after flash chromatography of the residue using hexanes/ethyl acetate (8:2) as eluent gave compound **2a** (120 mg, 62%) as a colorless oil; ^1H NMR (300 MHz, $CDCl_3$, 25 ºC): δ = 7.97 (m, 2H, CH^{Ar}, NH), 7.31 (m, 1H, CH^{Ar}), 7.15 (m, 1H, CH^{Ar}), 7.02 (m, 1H, CH^{Ar}), 5.07 (m, 1H, C*H*OH), 4.94 (m, 2H, C=·=CH_2), 3.74 (s, 3H, OCH_3), 2.96 (s, 1H, OH), 1.55 (t, 3H, *J* = 3.1 Hz, CH_3); ^{13}C NMR (75 MHz, $CDCl_3$, 25 ºC): δ = 203.9 (C=*C*=CH_2), 154.2 (C=O), 137.1 ($C^{Ar\text{-}q}$), 128.8 (CH^{Ar}), 128.6 (CH^{Ar}), 128.0 ($C^{Ar\text{-}q}$), 122.8 (CH^{Ar}), 121.0 (CH^{Ar}), 101.0 (*C*=C=CH_2), 78.7 (C=C=*C*H_2), 74.5 (CHOH), 52.1 (OCH_3), 15.3 (CH_3); IR ($CHCl_3$): ν = 3487 (OH), 1959 (C=C=C), 1764 (C=O) cm^{-1}; HRMS (ESI) m/z: $[M + Na]^+$ Calcd for $C_{13}H_{15}NO_3Na$ 256.0944; Found 256.0944.

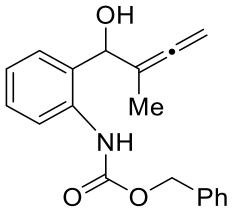

2b

Allenol 2b. From 150 mg (0.58 mmol) of the corresponding aldehyde **2'**, and after flash chromatography of the residue using hexanes/ethyl acetate (9:1) as eluent gave compound **2b** (134 mg, 74%) as a colorless oil; ^{1}H NMR (300 MHz, $CDCl_3$, 25 °C): δ = 8.05 (m, 2H, CH^{Ar}, NH), 7.36 (m, 6H, 6CH^{Ar}), 7.16 (dd, 1H, *J* = 7.6, 1.5 Hz, CH^{Ar}), 7.04 (td, 1H, *J* = 7.5, 1.1 Hz, CH^{Ar}), 5.24 (d, 1H, *J* = 12.3 Hz, OC*H*H), 5.17 (d, 1H, *J* = 12.3 Hz, OCH*H*), 5.08 (m, 1H, C*H*OH), 4.87 (m, 2H, C=·=CH_2), 2.78 (d, 1H, *J* = 2.7 Hz, OH), 1.55 (t, 3H, *J* = 3.0 Hz, CH_3); ^{13}C NMR (75 MHz, $CDCl_3$, 25 °C): δ = 203.8 (C=*C*=CH_2), 153.6 (C=O), 137.1 (*C*$^{Ar-q}$), 136.3 (*C*$^{Ar-q}$), 128.9 (CH^{Ar}), 128.7 (CH^{Ar}), 128.5 (2CH^{Ar}), 128.1 (3CH^{Ar}), 122.9 (CH^{Ar}), 121.2 (CH^{Ar}), 101.1 (*C*=C=CH_2), 78.9 (C=C=*C*H_2), 74.6 (CHOH), 66.7 (OCH_2), 15.4 (CH_3); IR ($CHCl_3$): ν = 3498 (OH), 1963 (C=C=C), 1724 (C=O) cm^{-1}; HRMS (ESI) m/z: $[M + Na]^+$ Calcd for $C_{19}H_{19}NO_3Na$ 332.1257; Found 332.1259.

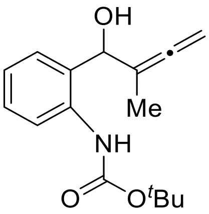

2c

Allenol 2c. From 164 mg (0.74 mmol) of the corresponding aldehyde **2'**, and after flash chromatography of the residue using hexanes/ethyl acetate (9:1) as eluent gave compound **2c** (195 mg, 96%) as a colorless oil; ^{1}H NMR (300 MHz, $CDCl_3$, 25 °C): δ = 7.99 (d, 1H, *J* = 8.1 Hz, CH^{Ar}), 7.69 (s, 1H, NH), 7.32 (m, 1H, CH^{Ar}), 7.18 (dd, 1H, *J* = 7.6, 1.6 Hz, CH^{Ar}), 7.01 (td, 1H, *J* = 7.5, 1.2 Hz, CH^{Ar}), 5.09 (m, 1H, C*H*OH), 5.01 (m, 2H, C=·=CH_2), 2.53 (d, 1H, *J* = 2.8 Hz, OH), 1.56 (t, 3H, *J* = 3.0 Hz, CH_3), 1.53 (s, 9H, 3CH_3); ^{13}C NMR (75 MHz, $CDCl_3$, 25 °C): δ = 203.9 (C=*C*=CH_2), 153.0 (C=O), 137.6 (*C*$^{Ar-q}$), 128.9 (CH^{Ar}), 128.6 (CH^{Ar}), 127.9 (*C*$^{Ar-q}$), 122.6 (CH^{Ar}), 121.2 (CH^{Ar}),

101.2 (C=C=CH$_2$), 80.0 (C^{Cq}), 78.9 (C=C=CH$_2$), 74.4 (CHOH), 28.4 (3CH$_3$), 15.4 (CH$_3$); IR (CHCl$_3$): ν = 3477 (OH), 1943 (C=C=C), 1764 (C=O) cm^{-1}; HRMS (ESI) m/z: [M + Na]$^+$ Calcd for $C_{16}H_{21}NO_3Na$ 298.1414; Found 298.1407.

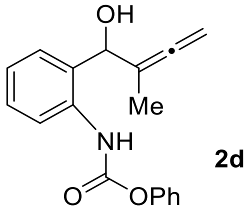

2d

Allenol 2d. From 150 mg (0.62 mmol) of the corresponding aldehyde **2'**, and after flash chromatography of the residue using hexanes/ethyl acetate (9:1) as eluent gave compound **2d** (102 mg, 55%) as a colorless oil; ^{1}H NMR (300 MHz, CDCl$_3$, 25 ºC): δ = 8.47 (s, 1H, NH), 8.07 (d, 1H, J = 7.7 Hz, CH^{Ar}), 7.38 (m, 3H, 3CH^{Ar}), 7.23 (m, 4H, 4CH^{Ar}), 7.08 (td, 1H, J = 7.5, 0.9 Hz, CH^{Ar}), 5.12 (m, 1H, CHOH), 5.03 (m, 2H, C=·=CH$_2$), 2.86 (d, 1H, J = 2.4 Hz, OH), 1.61 (t, 3H, J = 3.1 Hz, CH$_3$); ^{13}C NMR (75 MHz, CDCl$_3$, 25 ºC): δ = 203.9 (C=C=CH$_2$), 151.9 (C=O), 150.8 (C^{Ar-q}), 136.9 (C^{Ar-q}), 129.4 (2CH^{Ar}), 129.1 (CH^{Ar}), 128.9 (CH^{Ar}), 128.1 (C^{Ar-q}), 125.6 (CH^{Ar}), 123.4 (CH^{Ar}), 121.7 (2CH^{Ar}), 121.1 (CH^{Ar}), 101.3 (C=C=CH$_2$), 79.2 (C=C=CH$_2$), 74.9 (CHOH), 15.6 (CH$_3$); IR (CHCl$_3$): ν = 3481 (OH), 1950 (C=C=C), 1737 (C=O) cm^{-1}; HRMS (ESI) m/z: [M + Na]$^+$ Calcd for $C_{18}H_{17}NO_3Na$ 318.1101; Found 318.1108.

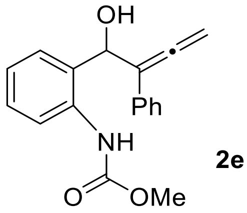

2e

Allenol 2e. From 100 mg (0.56 mmol) of the corresponding aldehyde **2'**, and after flash chromatography of the residue using hexanes/ethyl acetate (9:1→8:2) as eluent gave compound **2e** (136 mg, 82%) as a pale yellow oil; ^{1}H NMR (300 MHz, CDCl$_3$, 25 ºC): δ = 7.95 (s, 1H, NH), 7.84

(d, 1H, *J* = 7.9 Hz, CH^{Ar}), 7.19 (m, 5H, $5CH^{Ar}$), 7.11 (m, 2H, $2CH^{Ar}$), 6.88 (td, 1H, *J* = 7.5, 1.2 Hz, CH^{Ar}), 5.68 (s, 1H, C*H*OH), 5.19 (m, 2H, C=·=CH_2), 3.68 (s, 3H, OCH_3), 2.78 (s, 1H, OH); ^{13}C NMR (75 MHz, $CDCl_3$, 25 ºC): δ = 207.1 (C=*C*=CH_2), 154.5 (C=O), 137.3 ($C^{Ar\text{-}q}$), 133.6 ($C^{Ar\text{-}q}$), 129.4 ($C^{Ar\text{-}q}$), 129.0 (CH^{Ar}), 128.5 ($2CH^{Ar}$), 128.4 (CH^{Ar}), 127.3 (CH^{Ar}), 126.7 ($2CH^{Ar}$), 123.3 (CH^{Ar}), 121.6 (CH^{Ar}), 108.1 (*C*=C=CH_2), 81.8 (C=C=*C*H_2), 71.0 (CHOH), 52.2 (OCH_3); IR ($CHCl_3$): ν = 3481 (OH), 1969 (C=C=C), 1768 (C=O) cm^{-1}; HRMS (ESI) m/z: $[M + Na]^+$ Calcd for $C_{18}H_{17}NO_3Na$ 318.1101; Found 318.1010.

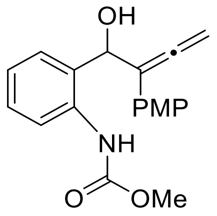

PMP = 4-MeOC_6H_4

2f

Allenol 2f. From 100 mg (0.55 mmol) of the corresponding aldehyde **2'**, and after flash chromatography of the residue using hexanes/ethyl acetate (8:2) as eluent gave compound **2f** (167 mg, 92%) as a pale yellow oil; ^1H NMR (300 MHz, $CDCl_3$, 25 ºC): δ = 8.08 (s, 1H, NH), 7.94 (d, 1H, *J* = 7.8 Hz, CH^{Ar}), 7.24 (m, 4H, $4CH^{Ar}$), 6.98 (m, 1H, $1CH^{Ar}$), 6.81 (m, 2H, $2CH^{Ar}$), 5.72 (s, 1H, C*H*OH), 5.25 (m, 2H, C=·=CH_2), 3.77 (s, 3H, OCH_3), 3.75 (s, 3H, OCH_3), 3.09 (s, 1H, OH); ^{13}C NMR (75 MHz, $CDCl_3$, 25 ºC): δ = 206.8 (C=*C*=CH_2), 158.7 (C=O), 154.5 ($C^{Ar\text{-}q}$), 137.2 ($C^{Ar\text{-}q}$), 129.5 ($C^{Ar\text{-}q}$), 128.8 (CH^{Ar}), 128.4 (CH^{Ar}), 127.9 ($2CH^{Ar}$), 125.7 ($C^{Ar\text{-}q}$), 123.2 (CH^{Ar}), 121.5 (CH^{Ar}), 113.9 ($2CH^{Ar}$), 107.6 (*C*=C=CH_2), 81.6 (C=C=*C*H_2), 71.1 (CHOH), 55.1 (OCH_3), 52.2 (OCH_3); IR ($CHCl_3$): ν = 3480 (OH), 1970 (C=C=C), 1769 (C=O) cm^{-1}; HRMS (ESI) m/z: $[M + Na]^+$ Calcd for $C_{19}H_{19}NO_4Na$ 348.1206; Found 348.1215.

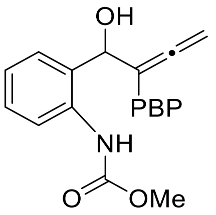

PBP = 4-BrC_6H_4

2g

Allenol 2g. From 100 mg (0.55 mmol) of the corresponding aldehyde **2'**, and after flash chromatography of the residue using hexanes/ethyl acetate (85:15→80:20) as eluent gave compound **2g** (175 mg, 85%) as a colorless oil; ^{1}H NMR (300 MHz, $CDCl_3$, 25 ºC): δ = 8.02 (s, 1H, NH), 7.90 (d, 1H, *J* = 7.9 Hz, CH^{Ar}), 7.38 (m, 2H, $2CH^{Ar}$), 7.28 (m, 1H, $1CH^{Ar}$), 7.16 (m, 3H, $3CH^{Ar}$), 6.98 (m, 1H, $1CH^{Ar}$), 5.70 (m, 1H, C*H*OH), 5.26 (m, 2H, C=·=CH_2), 3.76 (s, 3H, OCH_3), 3.15 (d, 1H, *J* = 4.6 Hz, OH); ^{13}C NMR (75 MHz, $CDCl_3$, 25 ºC): δ = 207.2 (C=*C*=CH_2), 154.5 (C=O), 137.1 ($C^{Ar\text{-}q}$), 132.7 ($C^{Ar\text{-}q}$), 131.5 ($2CH^{Ar}$), 129.3 ($C^{Ar\text{-}q}$), 129.1 (CH^{Ar}), 128.4 ($2CH^{Ar}$), 128.3 (CH^{Ar}), 123.4 (CH^{Ar}), 121.7 ($C^{Ar\text{-}q}$), 121.1 (CH^{Ar}), 107.3 (*C*=C=CH_2), 82.0 (C=C=*C*H_2), 70.9 (CHOH), 52.3 (OCH_3); IR ($CHCl_3$): ν = 3485 (OH), 1965 (C=C=C), 1764 (C=O) cm^{-1}; HRMS (ESI) m/z: $[M + Na]^+$ Calcd for $C_{18}H_{16}BrNO_3Na$ 396.0206; Found 396.0215.

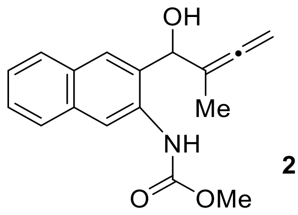

2h

Allenol 2h. From 100 mg (0.43 mmol) of the corresponding aldehyde **2'**, and after flash chromatography of the residue using hexanes/ethyl acetate (9:1) as eluent gave compound **2h** (113 mg, 91%) as a colorless oil; ^{1}H NMR (300 MHz, $CDCl_3$, 25 ºC): δ = 8.46 (s, 1H, NH), 8.20 (s, 1H, CH^{Ar}), 7.80 (d, 1H, *J* = 8.0 Hz, CH^{Ar}), 7.73 (d, 1H, *J* = 7.9 Hz, CH^{Ar}), 7.43 (m, 2H, $2CH^{Ar}$), 5.21 (s, 1H, C*H*OH), 4.98 (m, 2H, C=·=CH_2), 3.79 (s, 3H, OCH_3), 3.04 (m, 1H, OH), 1.56 (t, 3H, *J* = 3.0 Hz, CH_3); ^{13}C NMR (75 MHz, $CDCl_3$, 25 ºC): δ = 204.0 (C=*C*=CH_2), 154.3 (C=O), 134.6 ($C^{Ar\text{-}q}$), 133.7 ($C^{Ar\text{-}q}$), 129.2 ($C^{Ar\text{-}q}$), 128.1 (CH^{Ar}), 127.4 (CH^{Ar}), 127.3 (CH^{Ar}), 126.6 (CH^{Ar}), 124.9 (CH^{Ar}), 117.6 (CH^{Ar}), 101.1 (*C*=C=CH_2), 79.0 (C=C=*C*H_2), 75.0 (CHOH), 52.2 (OCH_3), 15.5 (CH_3); IR ($CHCl_3$): ν = 3485 (OH), 1948 (C=C=C), 1757 (C=O) cm^{-1}; HRMS (ESI) m/z: $[M + Na]^+$ Calcd for $C_{17}H_{17}NO_3Na$ 306.1101; Found 306.1103.

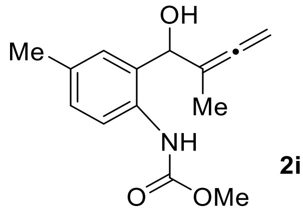

2i

Allenol 2i. From 100 mg (0.52 mmol) of the corresponding aldehyde **2'**, and after flash chromatography of the residue using hexanes/ethyl acetate (85:15) as eluent gave compound **2i** (129 mg, quantitative yield) as a colorless solid; mp 98–100 ºC; ^{1}H NMR (300 MHz, $CDCl_3$, 25 ºC): δ = 7.81 (m, 2H, CH^{Ar}, NH), 7.10 (d, 1H, J = 8.3 Hz, CH^{Ar}), 6.97 (s, 1H, CH^{Ar}), 5.03 (m, 1H, C*H*OH), 4.93 (m, 2H, C=·=CH_2), 3.73 (s, 3H, OCH_3), 2.96 (m, 1H, OH), 2.30 (s, 3H, CH_3), 1.55 (t, 3H, J = 3.1 Hz, CH_3); ^{13}C NMR (75 MHz, $CDCl_3$, 25 ºC): δ = 204.0 (C=*C*=CH_2), 154.4 (C=O), 134.4 ($C^{Ar\text{-}q}$), 132.5 ($C^{Ar\text{-}q}$), 129.3 (CH^{Ar}), 129.1 (CH^{Ar}), 128.4 ($C^{Ar\text{-}q}$), 121.3 (CH^{Ar}), 101.1 (*C*=C=CH_2), 78.6 (C=C=*C*H_2), 74.3 (CHOH), 52.1 (OCH_3), 20.7 (CH_3), 15.3 (CH_3); IR ($CHCl_3$): ν = 3352 (OH), 1955 (C=C=C), 1724 (C=O) cm^{-1}. Badly ionizing compound in MS.

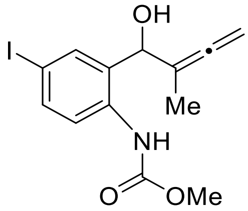

2j

Allenol 2j. From 150 mg (0.49 mmol) of the corresponding aldehyde **2'**, and after flash chromatography of the residue using hexanes/ethyl acetate (9:1) as eluent gave compound **2j** (155 mg, 88%) as a colorless oil; ^{1}H NMR (300 MHz, $CDCl_3$, 25 ºC): δ = 7.96 (s, 1H, NH), 7.75 (d, 1H, J = 8.4 Hz, CH^{Ar}), 7.57 (dd, 1H, J = 8.7, 1.9 Hz, CH^{Ar}), 7.46 (d, 1H, J = 2.0 Hz, CH^{Ar}), 4.99 (s, 1H, C*H*OH), 4.93 (m, 2H, C=·=CH_2), 3.73 (s, 3H, OCH_3), 3.11 (m, 1H, OH), 1.56 (t, 3H, J = 3.1 Hz, CH_3); ^{13}C NMR (75 MHz, $CDCl_3$, 25 ºC): δ = 204.1 (C=*C*=CH_2), 153.9 (C=O), 137.6 (CH^{Ar}), 136.9 (CH^{Ar}), 130.3 ($C^{Ar\text{-}q}$), 122.7 ($C^{Ar\text{-}q}$), 100.5 (*C*=C=CH_2), 86.0 ($C^{Ar\text{-}q}$), 78.9 (C=C=*C*H_2), 73.9 (CHOH),

52.3 (OCH_3), 15.2 (CH_3); IR ($CHCl_3$): ν = 3485 (OH), 1955 (C=C=C), 1757 (C=O) cm^{-1}; HRMS (ESI) m/z: $[M + Na]^+$ Calcd for $C_{13}H_{14}INO_3Na$ 381.9911; Found 381.9908.

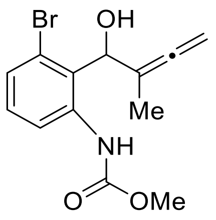

2k

Allenol 2k. From 150 mg (0.58 mmol) of the corresponding aldehyde **2'**, and after flash chromatography of the residue using hexanes/ethyl acetate (9:1) as eluent gave compound **2k** (155 mg, 85%) as a colorless solid; mp 105–107 ºC; ^1H NMR (300 MHz, $CDCl_3$, 25 ºC): δ = 8.55 (s, 1H, NH), 7.96 (d, 1H, *J* = 8.1 Hz, CH^{Ar}), 7.23 (d, 1H, *J* = 8.0, 1.2 Hz, CH^{Ar}), 7.10 (t, 1H, *J* = 8.1 Hz, CH^{Ar}), 5.90 (m, 1H, C*H*OH), 4.87 (m, 2H, C=·=CH_2), 3.73 (s, 3H, OCH_3), 3.26 (d, 1H, *J* = 2.6 Hz, OH), 1.60 (t, 3H, *J* = 2.9 Hz, CH_3); ^{13}C NMR (75 MHz, $CDCl_3$, 25 ºC): δ = 204.0 (C=*C*=CH_2), 154.1 (C=O), 139.3 ($C^{Ar\text{-}q}$), 129.5 (CH^{Ar}), 127.3 (CH^{Ar}), 126.7 ($C^{Ar\text{-}q}$), 132.9 ($C^{Ar\text{-}q}$), 120.4 (CH^{Ar}), 100.5 (*C*=C=CH_2), 78.9 (C=C=*C*H_2), 73.4 (CHOH), 52.2 (OCH_3), 15.4 (CH_3); IR ($CHCl_3$): ν = 3388 (OH), 1946 (C=C=C), 1725 (C=O) cm^{-1}; HRMS (ESI) m/z: $[M + Na]^+$ Calcd for $C_{13}H_{14}BrNO_3Na$ 334.0049; Found 334.0036.

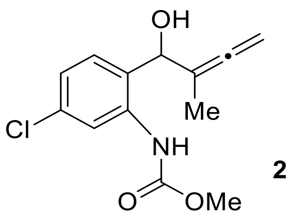

2l

Allenol 2l. From 100 mg (0.46 mmol) of the corresponding aldehyde **2'**, and after flash chromatography of the residue using hexanes/ethyl acetate (9:1) as eluent gave compound **2l** (88 mg, 70%) as a colorless solid; mp 95–97 ºC; ^1H NMR (300 MHz, $CDCl_3$, 25 ºC): δ = 8.09 (m, 2H, NH, CH^{Ar}), 7.07 (d, 1H, *J* = 8.2 Hz, CH^{Ar}), 6.98 (dd, 1H, *J* = 8.2, 2.1 Hz, CH^{Ar}), 5.06 (m, 1H, C*H*OH),

4.96 (m, 2H, C=·=CH$_2$), 3.76 (s, 3H, OCH$_3$), 2.79 (d, 1H, J = 2.6 Hz, OH), 1.55 (t, 3H, J = 3.0 Hz, CH$_3$); ^{13}C NMR (75 MHz, CDCl$_3$, 25 °C): δ = 203.9 (C=*C*=CH$_2$), 153.9 (C=O), 138.3 (C^{Ar-q}), 134.6 (C^{Ar-q}), 129.6 (CH^{Ar}), 125.9 (C^{Ar-q}), 122.6 (CH^{Ar}), 120.7 (CH^{Ar}), 100.8 (*C*=C=CH$_2$), 79.1 (C=C=*C*H$_2$), 74.3 (CHOH), 52.3 (OCH$_3$), 15.3 (CH$_3$); IR (CHCl$_3$): ν = 3426 (OH), 1956 (C=C=C), 1744 (C=O) cm^{-1}; HRMS (ESI) m/z: [M + Na]$^+$ Calcd for $C_{13}H_{14}ClNO_3Na$ 290.0554; Found 290.0561.

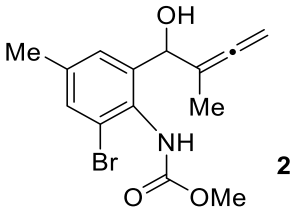

2m

Allenol 2m. From 150 mg (0.55 mmol) of the corresponding aldehyde **2'**, and after flash chromatography of the residue using hexanes/ethyl acetate (8:2) as eluent gave compound **2m** (143 mg, 80%) as a colorless oil; ^{1}H NMR (300 MHz, CDCl$_3$, 25 °C): δ = 7.37 (s, 1H, CH^{Ar}), 7.22 (s, 1H, CH^{Ar}), 6.58 (s, 1H, NH), 5.19 (s, 1H, C*H*OH), 4.87 (m, 2H, C=·=CH$_2$), 3.73 (m, 3H, OCH$_3$), 3.54 (s, 1H, OH), 2.31 (s, 3H, CH$_3$), 1.51 (t, 3H, J = 3.0 Hz, CH$_3$); ^{13}C NMR (75 MHz, CDCl$_3$, 25 °C): δ = 205.0 (C=*C*=CH$_2$), 155.6 (C=O), 140.1 (C^{Ar-q}), 138.7 (C^{Ar-q}), 132.8 (CH^{Ar}), 130.7 (C^{Ar-q}), 128.1 (CH^{Ar}), 101.0 (*C*=C=CH$_2$), 77.7 (C=C=*C*H$_2$), 71.0 (CHOH), 52.8 (OCH$_3$), 20.8 (CH$_3$), 15.1 (CH$_3$); IR (CHCl$_3$): ν = 3465 (OH), 1938 (C=C=C), 1724 (C=O) cm^{-1}; HRMS (ESI) m/z: [M + Na]$^+$ Calcd for $C_{14}H_{16}BrNO_3Na$ 348.0206; Found 348.0217.

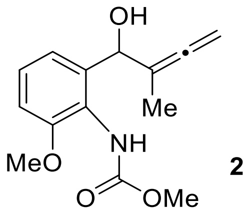

2n

Allenol 2n. From 100 mg (0.48 mmol) of the corresponding aldehyde **2'**, and after flash chromatography of the residue using hexanes/ethyl acetate (8:2) as eluent gave compound **2n** (100 mg, 79%) as a colorless oil; ^{1}H NMR (300 MHz, $CDCl_3$, 25 °C): δ = 7.24 (t, 1H, *J* = 8.0 Hz, CH^{Ar}), 7.10 (dd, 1H, *J* = 7.9, 1.2 Hz, CH^{Ar}), 6.86 (dd, 1H, *J* = 8.1, 1.3 Hz, CH^{Ar}), 6.43 (s, 1H, NH), 5.24 (m, 1H, C*H*OH), 4.90 (m, 2H, C=·=CH_2), 3.83 (s, 3H, OCH_3), 3.75 (s, 3H, OCH_3), 1.52 (t, 3H, *J* = 2.9 Hz, CH_3); ^{13}C NMR (75 MHz, $CDCl_3$, 25 °C): δ = 205.0 (C=*C*=CH_2), 156.5 (C=O), 154.0 ($C^{Ar\text{-}q}$), 138.9 ($C^{Ar\text{-}q}$), 127.5 (CH^{Ar}), 124.0 ($C^{Ar\text{-}q}$), 120.1 (CH^{Ar}), 110.5 (CH^{Ar}), 101.2 (*C*=C=CH_2), 77.6 (C=C=*C*H_2), 70.3 (CHOH), 55.7 (OCH_3), 52.7 (OCH_3), 15.5 (CH_3); IR ($CHCl_3$): ν = 3455 (OH), 1957 (C=C=C), 1736 (C=O) cm^{-1}; HRMS (ESI) m/z: $[M + Na]^+$ Calcd for $C_{14}H_{17}NO_4Na$ 286.1050; Found 286.1056.

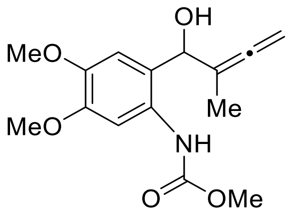

2o

Allenol 2o. From 150 mg (0.62 mmol) of the corresponding aldehyde **2'**, and after flash chromatography of the residue using hexanes/ethyl acetate (7:3→6:4) as eluent gave compound **2o** (138 mg, 76%) as a colorless oil; ^{1}H NMR (300 MHz, $CDCl_3$, 25 °C): δ = 7.75 (s, 1H, NH), 7.52 (s, 1H, CH^{Ar}), 6.63 (s, 1H, CH^{Ar}), 4.98 (s, 1H, C*H*OH), 4.84 (m, 2H, C=·=CH_2), 3.82 (s, 3H, OCH_3), 3.77 (s, 3H, OCH_3), 3.69 (s, 3H, OCH_3), 3.21 (s, 1H, OH), 1.51 (t, 3H, *J* = 3.0 Hz, CH_3); ^{13}C NMR (75 MHz, $CDCl_3$, 25 °C): δ = 204.0 (C=*C*=CH_2), 154.4 (C=O), 148.6 ($C^{Ar\text{-}q}$), 144.4 ($C^{Ar\text{-}q}$), 130.2 ($C^{Ar\text{-}q}$), 120.8 ($C^{Ar\text{-}q}$), 111.7 (CH^{Ar}), 105.6 (CH^{Ar}), 101.0 (*C*=C=CH_2), 78.1 (C=C=*C*H_2), 73.7 (CHOH), 56.1 (OCH_3), 55.7 (OCH_3), 52.0 (OCH_3), 15.1 (CH_3); IR ($CHCl_3$): ν = 3484 (OH), 1951 (C=C=C), 1767 (C=O) cm^{-1}; HRMS (ESI) m/z: $[M + Na]^+$ Calcd for $C_{15}H_{19}NO_5Na$ 316.1155; Found 316.1154.

Step E: The appropriate aldehyde **2'** (1.0 mmol) was dissolved in anhydrous THF (4 mL) and a solution of the corresponding alkenylmagnesium bromide (0.5 M THF solution; 3.0 mmol) was added at –78 ºC. The resulting mixture was stirring at –78 ºC under argon. On completion, the reaction mixture was quenched with NH_4Cl (aq. sat.). The aqueous phase was extracted with EtOAc and the combined organic layers were washed with brine, dried over anhydrous $MgSO_4$ and concentrated under reduced pressure. Purification by flash column chromatography on silica gel gave allyl alcohols **6a–6g**.

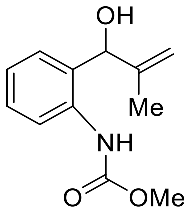

6a

Alkenol 6a. From 200 mg (1.11 mmol) of the corresponding aldehyde **2'**, and after flash chromatography of the residue using hexanes/ethyl acetate (9:1→8:2) as eluent gave compound **6a** (177 mg, 62%) as a colorless oil; ^{1}H NMR (300 MHz, $CDCl_3$, 25 ºC): δ = 7.94 (m, 2H, NH, CH^{Ar}), 7.31 (t, 1H, *J* = 7.8 Hz, CH^{Ar}), 7.17 (d, 1H, *J* = 7.2 Hz, CH^{Ar}), 7.05 (t, 1H, *J* = 7.4 Hz, CH^{Ar}), 5.22 (s, 1H, =C*H*H), 5.16 (s, 1H, =CH*H*), 5.06 (s, 1H, C*H*OH), 3.75 (s, 3H, OCH_3), 2.57 (br s, 1H, OH), 1.64 (s, 3H, CH_3); ^{13}C NMR (75 MHz, $CDCl_3$, 25 ºC): δ = 154.4 (C=O), 144.7 (*C*=CH_2), 137.0 ($C^{Ar\text{-}q}$), 128.8 (CH^{Ar}), 128.6 (CH^{Ar}), 123.2 (CH^{Ar}), 121.4 (CH^{Ar}), 111.3 (=CH_2), 77.1 (CHOH), 52.2 (OCH_3), 19.6 (CH_3); IR ($CHCl_3$): ν = 3475 (OH), 1722 (C=O) cm^{-1}; HRMS (ESI) m/z: $[M + Na]^+$ Calcd for $C_{12}H_{15}NO_3Na$ 244.0944; Found 244.0949.

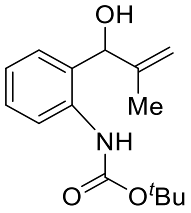

6b

Alkenol 6b. From 200 mg (0.90 mmol) of the corresponding aldehyde **2'**, and after flash chromatography of the residue using hexanes/ethyl acetate (9:1) as eluent gave compound **6b** (150 mg, 63%) as a colorless oil; ^{1}H NMR (300 MHz, $CDCl_3$, 25 °C): δ = 7.84 (d, 1H, *J* = 8.1 Hz, CH^{Ar}), 7.68 (s, 1H, NH), 7.28 (m, 1H, CH^{Ar}), 7.16 (dd, 1H, *J* = 7.6, 1.5 Hz, CH^{Ar}), 7.03 (td, 1H, *J* = 7.5, 1.2 Hz, CH^{Ar}), 5.22 (s, 1H, =C*H*H), 5.11 (s, 1H, =CH*H*), 5.05 (m, 1H, C*H*OH), 2.96 (s, 1H, OH), 1.63 (s, 3H, CH_3), 1.51 (s, 9H, $3CH_3$); ^{13}C NMR (75 MHz, $CDCl_3$, 25 °C): δ = 153.4 (C=O), 144.8 (*C*=CH_2), 137.2 ($C^{Ar\text{-}q}$), 129.7 ($C^{Ar\text{-}q}$), 128.6 (CH^{Ar}), 128.4 (CH^{Ar}), 123.1 (CH^{Ar}), 121.9 (CH^{Ar}), 111.0 (=CH_2), 80.1 (C^{Cq}), 76.5 (CHOH), 28.3 ($3CH_3$), 19.6 (CH_3); IR ($CHCl_3$): ν = 3486 (OH), 1699 (C=O) cm^{-1}; HRMS (ESI) m/z: $[M + Na]^+$ Calcd for $C_{15}H_{21}NO_3Na$ 286.1414; Found 286.1409.

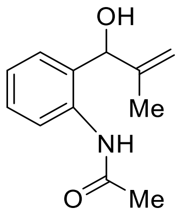

6c

Alkenol 6c. From 200 mg (1.22 mmol) of the corresponding aldehyde **2'**, and after flash chromatography of the residue using hexanes/ethyl acetate (6:4) as eluent gave compound **6c** (154 mg, 62%) as a colorless oil; ^{1}H NMR (300 MHz, $CDCl_3$, 25 °C): δ = 8.82 (s, 1H, NH), 7.94 (d, 1H, *J* = 8.1 Hz, CH^{Ar}), 7.23 (t, 1H, *J* = 7.7 Hz, CH^{Ar}), 7.12 (m, 1H, CH^{Ar}), 7.04 (m, 1H, CH^{Ar}), 5.17 (s, 1H, =C*H*H), 5.05 (s, 1H, =CH*H*), 4.98 (s, 1H, C*H*OH), 4.36 (br s, 1H, OH), 1.98 (s, 3H, CH_3), 1.55 (s, 3H, CH_3); ^{13}C NMR (75 MHz, $CDCl_3$, 25 °C): δ = 168.9 (C=O), 145.0 (*C*=CH_2), 136.5 ($C^{Ar\text{-}q}$), 130.4 ($C^{Ar\text{-}q}$), 128.8 (CH^{Ar}), 128.3 (CH^{Ar}), 124.0 (CH^{Ar}), 122.8 (CH^{Ar}), 110.2 (=CH_2), 76.8 (CHOH), 24.2 (CH_3), 19.6 (CH_3); IR ($CHCl_3$): ν = 3476 (OH), 1694 (C=O) cm^{-1}; HRMS (ESI) m/z: $[M + Na]^+$ Calcd for $C_{12}H_{15}NO_2Na$ 228.0995; Found 228.1001.

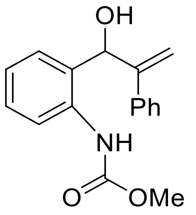

6d

Alkenol 6d. From 200 mg (1.11 mmol) of the corresponding aldehyde **2'**, and after flash chromatography of the residue using hexanes/ethyl acetate (85:15→80:20) as eluent gave compound **6d** (91 mg, 29%) as a colorless oil; ^{1}H NMR (300 MHz, $CDCl_3$, 25 ºC): δ = 7.84 (s, 1H, NH), 7.73 (d, 1H, *J* = 7.9 Hz, CH^{Ar}), 7.15 (m, 6H, 6CH^{Ar}), 7.03 (m, 1H, CH^{Ar}), 6.86 (m, 1H, CH^{Ar}), 5.65 (s, 1H, C*H*OH), 5.43 (s, 1H, =C*H*H), 5.32 (m, 1H, =CH*H*), 3.63 (s, 3H, OCH_3), 3.08 (br s, 1H, OH); ^{13}C NMR (75 MHz, $CDCl_3$, 25 ºC): δ = 154.6 (C=O), 148.6 (*C*=CH_2), 139.0 ($C^{Ar\text{-}q}$), 136.8 ($C^{Ar\text{-}q}$), 129.9 ($C^{Ar\text{-}q}$), 128.8 (CH^{Ar}), 128.3 (2CH^{Ar}), 127.7 (CH^{Ar}), 126.8 (2CH^{Ar}), 123.6 (CH^{Ar}), 121.8 (CH^{Ar}), 114.4 (=CH_2), 74.3 (CHOH), 52.3 (OCH_3); IR ($CHCl_3$): ν = 3503 (OH), 1705 (C=O) cm^{-1}; HRMS (ESI) m/z: $[M + Na]^+$ Calcd for $C_{17}H_{17}NO_3Na$ 306.1101; Found 306.1098.

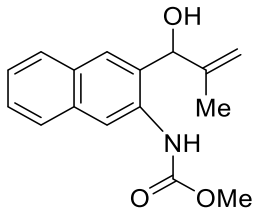

6e

Alkenol 6e. From 100 mg (0.43 mmol) of the corresponding aldehyde **2'**, and after flash chromatography of the residue using hexanes/ethyl acetate (85:15) as eluent gave compound **6e** (74 mg, 57%) as a colorless solid; mp 131–133 ºC; ^{1}H NMR (300 MHz, $CDCl_3$, 25 ºC): δ = 8.41 (s, 1H, NH), 8.15 (s, 1H, CH^{Ar}), 7.78 (d, 1H, *J* = 8.0 Hz, CH^{Ar}), 7.72 (d, 1H, *J* = 7.9 Hz, CH^{Ar}), 7.58 (s, 1H, CH^{Ar}), 7.42 (m, 2H, 2CH^{Ar}), 5.27 (s, 2H, =CH_2), 5.10 (s, 1H, C*H*OH), 3.77 (s, 3H, OCH_3), 2.91 (br s, 1H, OH), 1.65 (s, 3H, CH_3); ^{13}C NMR (75 MHz, $CDCl_3$, 25 ºC): δ = 154.4 (C=O), 144.6 (*C*=CH_2), 134.5 ($C^{Ar\text{-}q}$), 133.6 ($C^{Ar\text{-}q}$), 129.4 ($C^{Ar\text{-}q}$), 129.0 ($C^{Ar\text{-}q}$), 128.1 (CH^{Ar}), 127.4 (CH^{Ar}), 127.3 (CH^{Ar}), 126.6 (CH^{Ar}), 124.9 (CH^{Ar}), 117.8 (CH^{Ar}), 111.5 (=CH_2), 77.4 (CHOH), 52.2 (OCH_3), 19.8 (CH_3);

IR ($CHCl_3$): ν = 3483 (OH), 1697 (C=O) cm^{-1}; HRMS (ESI) m/z: $[M + Na]^+$ Calcd for $C_{16}H_{17}NO_3Na$ 294.1101; Found 294.1105.

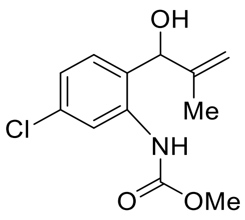

6f

Alkenol 6f. From 100 mg (0.47 mmol) of the corresponding aldehyde **2'**, and after flash chromatography of the residue using hexanes/ethyl acetate (9:1) as eluent gave compound **6f** (85 mg, 68%) as a colorless solid; mp 121–123 ºC; ^1H NMR (300 MHz, $CDCl_3$, 25 ºC): δ = 8.06 (m, 2H, NH, CH^{Ar}), 7.06 (d, 1H, *J* = 8.2 Hz, CH^{Ar}), 6.99 (dd, 1H, *J* = 8.2, 2.1 Hz, CH^{Ar}), 5.19 (s, 1H, =C*H*H), 5.11 (s, 1H, =CH*H*), 5.06 (m, 1H, C*H*OH), 3.74 (s, 3H, OCH_3), 2.79 (s, 1H, OH), 1.63 (s, 3H, CH_3); ^{13}C NMR (75 MHz, $CDCl_3$, 25 ºC): δ = 154.0 (C=O), 144.3 (*C*=CH_2), 138.1 ($C^{Ar\text{-}q}$), 134.5 ($C^{Ar\text{-}q}$), 129.6 (CH^{Ar}), 126.8 ($C^{Ar\text{-}q}$), 122.9 (CH^{Ar}), 120.8 (CH^{Ar}), 111.7 (=CH_2), 76.8 (CHOH), 52.4 (OCH_3), 19.4 (CH_3); IR ($CHCl_3$): ν = 3497 (OH), 1710 (C=O) cm^{-1}; HRMS (ESI) m/z: $[M + Na]^+$ Calcd for $C_{12}H_{14}ClNO_3Na$ 278.0554; Found 278.0563.

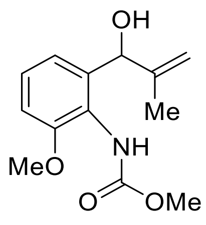

6g

Alkenol 6g. From 100 mg (0.47 mmol) of the corresponding aldehyde **2'**, and after flash chromatography of the residue using toluene/ethyl acetate (8:2) as eluent gave compound **6g** (83 mg, 70%) as a colorless oil; ^1H NMR (300 MHz, $CDCl_3$, 25 ºC): δ = 7.22 (t, 1H, *J* = 8.0 Hz, CH^{Ar}), 7.03 (dd, 1H, *J* = 7.9, 1.3 Hz, CH^{Ar}), 6.84 (dd, 1H, *J* = 8.2, 1.3 Hz, CH^{Ar}), 6.48 (br s, 1H, NH), 5.33 (s, 1H, =C*H*H), 5.22 (s, 1H, =CH*H*), 5.01 (m, 1H, C*H*OH), 4.02 (br s, 1H, OH), 3.83 (s, 3H, OCH_3),

3.76 (s, 3H, OCH_3), 1.51 (s, 3H, CH_3); ^{13}C NMR (75 MHz, $CDCl_3$, 25 °C): δ = 157.0 (C^{Ar-q}), 153.7 (C=O), 144.8 (*C*=CH_2), 139.4 (C^{Ar-q}), 127.6 (CH^{Ar}), 124.0 (C^{Ar-q}), 120.0 (CH^{Ar}), 110.3 (=CH_2), 110.1 (CH^{Ar}), 72.2 (CHOH), 55.7 (OCH_3), 52.9 (OCH_3), 19.6 (CH_3); IR ($CHCl_3$): ν = 3495 (OH), 1705 (C=O) cm^{-1}; HRMS (ESI) m/z: $[M + Na]^+$ Calcd for $C_{13}H_{17}NO_4Na$ 274.1050; Found 274.1050.

General procedure for the reaction of allenols 2a–o and alkenols 6a–g with Yanais'reagent 1 or deuterated Yanais'reagent [D]-1.

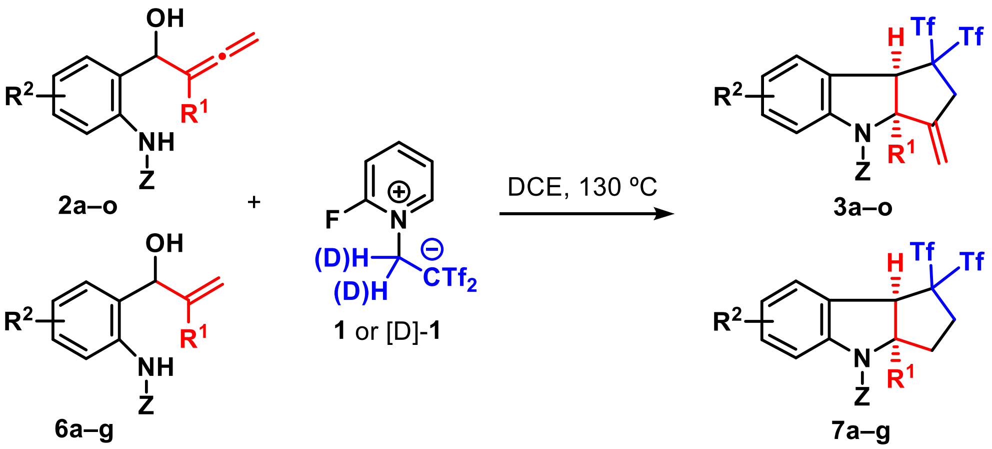

Yanai's reagent **1** or deuterated Yanai's reagent [D]-**1** (0.2 mmol) was added to a hot solution (130 °C, sealed tube) of the appropriate allenol **2** or alkenol **6** (0.2 mmol) in 1,2-dichloroethane (4 mL). The reaction was heated at 130 °C in a sealed tube until disappearance of the starting material (TLC), and then the mixture was concentrated under reduced pressure. Chromatography of the residue eluting with hexanes/ethyl acetate mixtures gave analytically pure compounds. Spectroscopic and analytical data for bis(triflyl)-decorated fused indolines **3a–o**, [D]-**3a**, and **7a–g** follow.

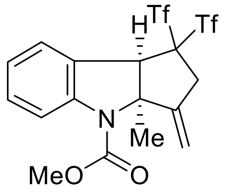

3a

Bis(triflyl)-decorated tricyclic indoline 3a. From 30 mg (0.13 mmol) of allenol **2a**, and after flash chromatography of the residue using hexanes/ethyl acetate (95:5) as eluent gave compound **3a** (40 mg, 62%) as a colorless solid; mp 119–121 ºC; ^{1}H NMR (500 MHz, $CHCl_3$, 25 ºC): δ = 7.74 (br s, 1H, CH^{Ar}), 7.45 (d, 1H, J = 7.6 Hz, CH^{Ar}), 7.38 (t, 1H, J = 7.9 Hz, CH^{Ar}), 7.09 (t, 1H, J = 7.5 Hz, CH^{Ar}), 5.61 (s, 1H, =C*H*H), 5.57 (d, 1H, J = 2.2 Hz, =CH*H*), 4.54 (s, 1H, CH), 3.87 (d, 1H, J = 17.6 Hz, C*H*H), 3.84 (s, 3H, OCH_3), 3.71 (d, 1H, J = 17.3 Hz, CH*H*), 1.66 (s, 3H, CH_3); ^{13}C NMR (125 MHz, $CHCl_3$, 25 ºC): δ = 152.8 (C=O), 143.8 ($C^{Ar\text{-}q}$), 142.1 (*C*=CH_2), 131.0 (CH^{Ar}), 128.7 (CH^{Ar}), 123.0 (CH^{Ar}), 120.5 ($C^{Ar\text{-}q}$), 120.4 (q, C-F_3, $^{1}J_{C\text{-}F}$ = 332.1 Hz), 119.4 (q, C-F_3, $^{1}J_{C\text{-}F}$ = 333.0 Hz), 117.1 (=CH_2), 115.4 (CH^{Ar}), 96.2 (CTf_2), 75.5 (C^{Cq}), 61.8 (CH), 52.4 (OCH_3), 40.1 (CH_2), 19.8 (CH_3); ^{19}F NMR (282 MHz, $CHCl_3$, 25 ºC): δ = –67.5 (s, 3F, CF_3), –69.8 (s, 3F, CF_3); IR ($CHCl_3$): ν = 1701 (C=O), 1392, 1203 (O=S=O), 1210 (C–F) cm^{-1}; HRMS (ESI) m/z: $[M + NH_4]^+$ Calcd for $C_{17}H_{19}F_6N_2O_6S_2$ 525.0583; Found 525.0586.

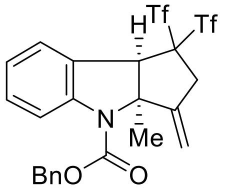

3b

Bis(triflyl)-decorated tricyclic indoline 3b. From 35 mg (0.11 mmol) of allenol **2b**, and after flash chromatography of the residue using toluene as eluent gave compound **3b** (40 mg, 61%) as a colorless oil; ^{1}H NMR (500 MHz, $CHCl_3$, 25 ºC): δ = 7.80 (br s, 1H, CH^{Ar}), 7.39 (m, 7H, 7CH^{Ar}), 7.09 (m, 1H, CH^{Ar}), 5.47 (m, 2H, =CH_2), 5.26 (s, 2H, OCH_2), 4.52 (s, 1H, CH), 3.85 (d, 1H, J = 17.2 Hz, C*H*H), 3.68 (d, 1H, J = 17.2 Hz, CH*H*), 1.63 (s, 3H, CH_3); ^{13}C NMR (125 MHz, $CHCl_3$, 25 ºC): δ = 152.2 (C=O), 143.9 ($C^{Ar\text{-}q}$), 141.8 (*C*=CH_2), 135.3 ($C^{Ar\text{-}q}$), 131.1 (CH^{Ar}), 128.7 (5CH^{Ar}), 128.6 (CH^{Ar}), 123.0 (CH^{Ar}), 120.5 ($C^{Ar\text{-}q}$), 120.4 (q, C-F_3, $^{1}J_{C\text{-}F}$ = 332.1 Hz), 119.4 (q, C-F_3, $^{1}J_{C\text{-}F}$ = 333.0 Hz), 117.4 (=CH_2), 115.8 (CH^{Ar}), 96.1 (CTf_2), 75.6 (C^{Cq}), 67.8 (OCH_2), 61.8 (CH), 40.1 (CH_2), 20.0 (CH_3); ^{19}F NMR (282 MHz, $CHCl_3$, 25 ºC): δ = –67.5 (s, 3F, CF_3), –69.8 (s, 3F, CF_3); IR ($CHCl_3$):

ν = 1700 (C=O), 1391, 1203 (O=S=O), 1207 (C–F) cm^{-1}; HRMS (ESI) m/z: $[M + NH_4]^+$ Calcd for $C_{23}H_{23}F_6N_2O_6S_2$ 601.0896; Found 601.0899.

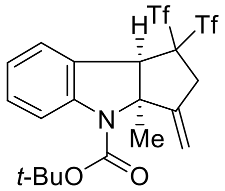

3c

Bis(triflyl)-decorated tricyclic indoline 3c. From 37 mg (0.13 mmol) of allenol **2c**, and after flash chromatography of the residue using toluene as eluent gave compound **3c** (50 mg, 66%) as a colorless oil; ^{1}H NMR (500 MHz, $CHCl_3$, 25 °C): δ = 7.81 (br d, 1H, J = 5.4 Hz, CH^{Ar}), 7.43 (d, 1H, J = 7.5 Hz, CH^{Ar}), 7.36 (t, 1H, J = 7.8 Hz, CH^{Ar}), 7.06 (t, 1H, J = 7.5 Hz, CH^{Ar}), 5.57 (s, 1H, $=CH_2$), 5.55 (s, 1H, $=CH_2$), 4.50 (s, 1H, CH), 3.85 (d, 1H, J = 17.1 Hz, C*H*H), 3.69 (d, 1H, J = 17.0 Hz, CH*H*), 1.64 (s, 3H, CH_3), 1.56 (s, 9H, $3CH_3$); ^{13}C NMR (125 MHz, $CHCl_3$, 25 °C): δ = 151.3 (C=O), 144.6 ($C^{Ar\text{-}q}$), 142.4 (*C*=CH_2), 130.9 (CH^{Ar}), 128.5 (CH^{Ar}), 122.5 (CH^{Ar}), 120.4 (q, C-F_3, $^1J_{C\text{-}F}$ = 332.2 Hz), 120.2 ($C^{Ar\text{-}q}$), 119.4 (q, C-F_3, $^1J_{C\text{-}F}$ = 333.2 Hz), 116.9 ($=CH_2$), 115.8 (CH^{Ar}), 96.2 (CTf_2), 82.4 (OC^{Cq}), 75.2 (C^{Cq}), 61.7 (CH), 40.1 (CH_2), 28.4 ($3CH_3$), 19.8 (CH_3); ^{19}F NMR (282 MHz, $CHCl_3$, 25 °C): δ = –67.6 (s, 3F, CF_3), –69.8 (s, 3F, CF_3); IR ($CHCl_3$): ν = 1703 (C=O), 1390, 1202 (O=S=O), 1205 (C–F) cm^{-1}; HRMS (ESI) m/z: $[M + NH_4]^+$ Calcd for $C_{20}H_{25}F_6N_2O_6S_2$ 567.1053; Found 567.1055.

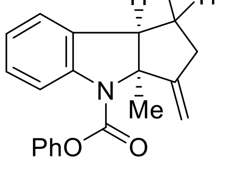

3d

Bis(triflyl)-decorated tricyclic indoline 3d. From 35 mg (0.12 mmol) of allenol **2d**, and after flash chromatography of the residue using hexanes/ethyl acetate (9:1) as eluent gave compound **3d** (37 mg, 55%) as a colorless oil, containing *ca.* 8% (^{1}H NMR spectroscopy) of a rotamer; ^{1}H NMR (500

MHz, $CHCl_3$, 25 ºC): δ = 7.80 (br s, 1H, CH^{Ar}), 7.43 (d, 1H, J = 7.6 Hz, CH^{Ar}), 7.33 (m, 3H, $3CH^{Ar}$), 7.19 (m, 1H, CH^{Ar}), 7.08 (m, 3H, $3CH^{Ar}$), 5.56 (s, 1H, =C*H*H), 5.52 (s, 1H, =CH*H*), 4.54 (s, 1H, CH), 3.89 (d, 1H, J = 17.2 Hz, C*H*H), 3.69 (d, 1H, J = 17.3 Hz, CH*H*), 1.70 (s, 3H, CH_3); ^{13}C NMR (125 MHz, $CHCl_3$, 25 ºC): δ = 150.6 (C=O), 150.0 ($C^{Ar\text{-}q}$), 143.5 ($C^{Ar\text{-}q}$), 141.7 (*C*=CH_2), 131.2 (CH^{Ar}), 129.6 ($3CH^{Ar}$), 128.8 (CH^{Ar}), 126.0 (CH^{Ar}), 123.5 (CH^{Ar}), 121.6 (CH^{Ar}), 120.7 ($C^{Ar\text{-}q}$), 120.4 (q, C-F_3, $^1J_{C\text{-}F}$ = 332.1 Hz), 119.4 (q, C-F_3, $^1J_{C\text{-}F}$ = 333.2 Hz), 117.6 (=CH_2), 116.1 (CH^{Ar}), 96.1 (CTf_2), 75.9 (C^{Cq}), 61.9 (CH), 40.1 (CH_2), 20.2 (CH_3); ^{19}F NMR (282 MHz, $CHCl_3$, 25 ºC): δ = –67.5 (s, 3F, CF_3), –69.7 (s, 3F, CF_3); IR ($CHCl_3$): ν = 1709 (C=O), 1379, 1196 (O=S=O), 1209 (C–F) cm^{-1}; HRMS (ESI) m/z: $[M + Na]^+$ Calcd for $C_{22}H_{17}F_6NO_6S_2Na$ 592.0294; Found 592.0293.

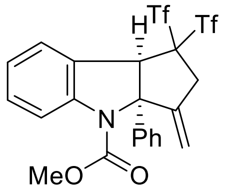

3e

Bis(triflyl)-decorated tricyclic indoline 3e. From 40 mg (0.13 mmol) of allenol **2e**, and after flash chromatography of the residue using hexanes/ethyl acetate (95:5) as eluent gave compound **3e** (47 mg, 61%) as a colorless solid; mp 186–188 ºC; ^1H NMR (500 MHz, $CHCl_3$, 25 ºC): δ = 7.95 (br s, 1H, CH^{Ar}), 7.42 (m, 1H, CH^{Ar}), 7.30 (m, 6H, $6CH^{Ar}$), 7.05 (td, 1H, J = 7.6, 0.9 Hz, CH^{Ar}), 5.76 (d, 1H, J = 2.2 Hz, =C*H*H), 5.30 (d, 1H, J = 1.6 Hz, =CH*H*), 4.71 (s, 1H, CH), 4.07 (d, 1H, J = 17.6 Hz, C*H*H), 3.92 (d, 1H, J = 17.6 Hz, CH), 3.78 (s, 3H, OCH_3); ^{13}C NMR (125 MHz, $CHCl_3$, 25 ºC): δ = 153.3 (C=O), 145.3 ($C^{Ar\text{-}q}$), 142.9 (*C*=CH_2), 136.8 ($C^{Ar\text{-}q}$), 131.3 (CH^{Ar}), 128.7 (CH^{Ar}), 128.6 ($2CH^{Ar}$), 128.2 (CH^{Ar}), 127.0 ($2CH^{Ar}$), 123.3 (CH^{Ar}), 120.4 (=CH_2), 120.4 (q, C-F_3, $^1J_{C\text{-}F}$ = 332.1 Hz), 120.0 ($C^{Ar\text{-}q}$), 119.5 (q, C-F_3, $^1J_{C\text{-}F}$= 333.4 Hz), 115.2 (CH^{Ar}), 96.0 (CTf_2), 82.2 (C^{Cq}), 64.9 (CH), 52.5 (OCH_3), 40.6 (CH_2); ^{19}F NMR (282 MHz, $CHCl_3$, 25 ºC): δ = –67.0 (s, 3F, CF_3), –69.3 (s, 3F, CF_3); IR ($CHCl_3$): ν = 1706 (C=O), 1392, 1202 (O=S=O), 1208 (C–F) cm^{-1}; HRMS (ES): calcd for

$C_{22}H_{21}F_6N_2O_6S_2$ [M+ NH_4]$^+$: 587.07397; found: 587.07269. HRMS (ESI) m/z: [M + NH_4]$^+$ Calcd for $C_{22}H_{21}F_6N_2O_6S_2$ 587.0740; Found 587.0727.

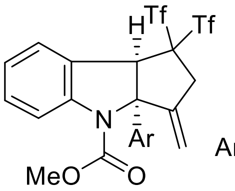

3f

Ar = 4-MeOC$_6$H$_4$

Bis(triflyl)-decorated tricyclic indoline 3f. From 40 mg (0.12 mmol) of allenol **2f**, and after flash chromatography of the residue using hexanes/ethyl acetate (95:5) as eluent gave compound **3f** (34 mg, 47%) as a colorless oil; ^{1}H NMR (500 MHz, $CHCl_3$, 25 ºC): δ = 7.93 (br s, 1H, CH^{Ar}), 7.41 (t, 1H, J = 7.8 Hz, 1H, CH^{Ar}), 7.28 (m, 1H, CH^{Ar}), 7.17 (m, 2H, $2CH^{Ar}$), 7.05 (t, 1H, J = 7.5 Hz, 1H, CH^{Ar}), 6.84 (m, 2H, $2CH^{Ar}$), 5.74 (d, 1H, J = 2.0 Hz, =C*H*H), 5.31 (s, 1H, CH), 4.66 (s, 1H, CH), 4.04 (d, 1H, J = 17.3 Hz, C*H*H), 3.90 (d, 1H, J = 17.7 Hz, =CH*H*), 3.77 (s, 6H, $2OCH_3$); ^{13}C NMR (125 MHz, $CHCl_3$, 25 ºC): δ = 159.3 ($C^{Ar\text{-}q}$), 153.3 (C=O), 145.4 (*C*=CH_2), 143.0 ($C^{Ar\text{-}q}$), 131.2 (CH^{Ar}), 128.8 (CH^{Ar}), 128.5 ($C^{Ar\text{-}q}$), 128.3 ($2CH^{Ar}$), 123.3 (CH^{Ar}), 120.4 (q, C-F_3, $^1J_{C\text{-}F}$ = 332.1 Hz), 120.3 (=CH_2), 120.1 ($C^{Ar\text{-}q}$), 119.5 (q, C-F_3, $^1J_{C\text{-}F}$ = 333.5 Hz), 115.2 (CH^{Ar}), 114.0 ($2CH^{Ar}$), 95.9 (CTf_2), 81.9 (C^{Cq}), 64.9 (CH), 55.2 (OCH_3), 52.5 (OCH_3), 40.5 (CH_2); ^{19}F NMR (282 MHz, $CHCl_3$, 25 ºC): δ = –67.0 (s, 3F, CF_3), –69.3 (s, 3F, CF_3); IR ($CHCl_3$): ν = 1697 (C=O), 1388, 1207 (O=S=O), 1208 (C–F) cm^{-1}; HRMS (ESI) m/z: [M + NH_4]$^+$ Calcd for $C_{23}H_{23}F_6N_2O_7S_2$ 617.0845; Found 617.0846.

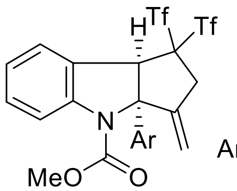

3g

Ar = 4-BrC$_6$H$_4$

Bis(triflyl)-decorated tricyclic indoline 3g. From 40 mg (0.10 mmol) of allenol **2g**, and after flash chromatography of the residue using toluene as eluent gave compound **3g** (39 mg, 57%) as a colorless

oil; ^{1}H NMR (500 MHz, $CHCl_3$, 25 °C): δ = 7.92 (br s, 1H, CH^{Ar}), 7.43 (m, 3H, $3CH^{Ar}$), 7.29 (m, 1H, CH^{Ar}), 7.14 (m, 2H, $2CH^{Ar}$), 7.07 (t, 1H, *J* = 7.5 Hz, 1H, CH^{Ar}), 5.76 (d, 1H, *J* = 2.2 Hz, =C*H*H), 5.30 (s, 1H, =CH*H*), 4.65 (s, 1H, CH), 4.05 (d, 1H, *J* = 17.6 Hz, C*H*H), 3.91 (d, 1H, *J* = 17.6 Hz, CH*H*), 3.78 (s, 3H, OCH_3); ^{13}C NMR (125 MHz, $CHCl_3$, 25 °C): δ = 153.1 (C=O), 145.0 (*C*=CH_2), 142.5 (C^{Ar-q}), 136.0 (C^{Ar-q}), 131.8 ($2CH^{Ar}$), 131.4 (CH^{Ar}), 128.8 ($2CH^{Ar}$), 128.7 (CH^{Ar}), 123.5 (CH^{Ar}), 122.5 (C^{Ar-q}), 120.5 (=CH_2), 120.4 (q, C-F_3, $^{1}J_{C-F}$ = 332.1 Hz), 119.6 (C^{Ar-q}), 119.5 (q, C-F_3, $^{1}J_{C-F}$ = 333.4 Hz), 115.1 (CH^{Ar}), 95.8 (CTf_2), 81.7 (C^{Cq}), 64.8 (CH), 52.7 (OCH_3), 40.6 (CH_2); ^{19}F NMR (282 MHz, $CHCl_3$, 25 °C): δ = –67.0 (s, 3F, CF_3), –69.3 (s, 3F, CF_3); IR ($CHCl_3$): ν = 1703 (C=O), 1386, 1198 (O=S=O), 1203 (C–F) cm^{-1}; HRMS (ESI) m/z: $[M + NH_4]^+$ Calcd for $C_{22}H_{20}BrF_6N_2O_6S_2$ 664.9845; Found 664.9850.

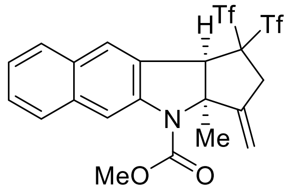

3h

Bis(triflyl)-decorated tricyclic indoline 3h. From 30 mg (0.10 mmol) of allenol **2h**, and after flash chromatography of the residue using hexanes/ethyl acetate (95:5→9:1) as eluent gave compound **3h** (45 mg, 77%) as a colorless oil; ^{1}H NMR (500 MHz, $CHCl_3$, 25 °C): δ = 8.11 (br s, 1H, CH^{Ar}), 7.91 (s, 1H, CH^{Ar}), 7.81 (m, 2H, $2CH^{Ar}$), 7.49 (m, 1H, CH^{Ar}), 7.41 (m, 1H, CH^{Ar}), 5.64 (d, 1H, *J* = 1.9 Hz, =C*H*H), 5.60 (d, 1H, *J* = 2.3 Hz, =CH*H*), 4.68 (s, 1H, CH), 3.92 (m, 4H, OCH_3, C*H*H), 3.74 (d, 1H, *J* = 17.5 Hz, CH*H*), 1.68 (s, 3H, CH_3); ^{13}C NMR (125 MHz, $CHCl_3$, 25 °C): δ = 153.0 (C=O), 142.1 (C^{Ar-q}), 140.8 (*C*=CH_2), 135.2 (C^{Ar-q}), 129.9 (C^{Ar-q}), 128.8 (CH^{Ar}), 128.0 (CH^{Ar}), 127.8 (CH^{Ar}), 127.3 (CH^{Ar}), 124.9 (CH^{Ar}), 122.0 (C^{Ar-q}), 120.5 (q, C-F_3, $^{1}J_{C-F}$ = 332.0 Hz), 119.4 (q, C-F_3, $^{1}J_{C-F}$ = 333.2 Hz), 117.1 (=CH_2), 112.0 (CH^{Ar}), 96.3 (CTf_2), 75.6 (C^{Cq}), 61.0 (CH), 52.6 (OCH_3), 40.0 (CH_2), 19.8 (CH_3); ^{19}F NMR (282 MHz, $CHCl_3$, 25 °C): δ = –67.4 (s, 3F, CF_3), –69.7 (s, 3F, CF_3); IR

($CHCl_3$): ν = 1706 (C=O), 1391, 1198 (O=S=O), 1207 (C–F) cm^{-1}; HRMS (ESI) m/z: $[M + H]^+$ Calcd for $C_{21}H_{18}F_6NO_6S_2$ 558.0474; Found 558.0490.

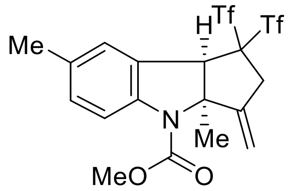

3i

Bis(triflyl)-decorated tricyclic indoline 3i. From 30 mg (0.12 mmol) of allenol **2i**, and after flash chromatography of the residue using hexanes/ethyl acetate (9:1) as eluent gave compound **3i** (42 mg, 68%) as a colorless oil; ^1H NMR (500 MHz, $CHCl_3$, 25 ºC): δ = 7.61 (br s, 1H, CH^{Ar}), 7.24 (s, 1H, CH^{Ar}), 7.17 (d, 1H, J = 8.3 Hz, CH^{Ar}), 5.60 (s, 1H, =C*H*H), 5.56 (d, 1H, J = 2.3 Hz, =CH*H*), 4.49 (s, 1H, CH), 3.87 (d, 1H, J = 17.3 Hz, C*H*H), 3.82 (s, 3H, OCH_3), 3.69 (d, 1H, J = 17.4 Hz, CH*H*), 2.35 (s, 3H, CH_3), 1.64 (s, 3H, CH_3); ^{13}C NMR (125 MHz, $CHCl_3$, 25 ºC): δ = 152.8 (C=O), 142.1 (C^{Ar-q}), 141.6 (*C*=CH_2), 132.6 (C^{Ar-q}), 131.6 (CH^{Ar}), 129.2 (CH^{Ar}), 120.5 (C^{Ar-q}), 120.4 (q, C-F_3, $^1J_{C-F}$ = 332.1 Hz), 119.4 (q, C-F_3, $^1J_{C-F}$ = 333.1 Hz), 116.9 (=CH_2), 115.3 (CH^{Ar}), 96.2 (CTf_2), 75.5 (C^{Cq}), 61.8 (CH), 52.3 (OCH_3), 40.1 (CH_2), 20.8 (CH_3), 19.7 (CH_3); ^{19}F NMR (282 MHz, $CHCl_3$, 25 ºC): δ = –67.6 (s, 3F, CF_3), –69.8 (s, 3F, CF_3); IR ($CHCl_3$): ν = 1699 (C=O), 1387, 1205 (O=S=O), 1210 (C–F) cm^{-1}; HRMS (ESI) m/z: $[M + NH_4]^+$ Calcd for $C_{18}H_{21}F_6N_2O_6S_2$ 539.0740; Found 539.0752.

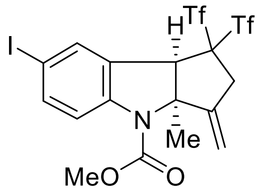

3j

Bis(triflyl)-decorated tricyclic indoline 3j. From 50 mg (0.14 mmol) of allenol **2j**, and after flash chromatography of the residue using hexanes/ethyl acetate (9:1→8:2) as eluent gave compound **3j** (47 mg, 54%) as a colorless oil; ^1H NMR (500 MHz, $CHCl_3$, 25 ºC): δ = 7.70 (s, 1H, CH^{Ar}), 7.65 (d, 1H, J = 8.7, 1.6 Hz, CH^{Ar}), 7.53 (br s, 1H, CH^{Ar}), 5.60 (s, 1H, =C*H*H), 5.57 (d, 1H, J = 2.3 Hz,

=CH*H*), 4.46 (s, 1H, CH), 3.82 (m, 4H, C*H*H, OCH_3), 3.70 (d, 1H, *J* = 17.4 Hz, CH*H*), 1.64 (s, 3H, CH_3); ^{13}C NMR (125 MHz, $CHCl_3$, 25 ºC): δ = 152.6 (C=O), 143.7 (C^{Ar-q}), 141.7 (*C*=CH_2), 139.8 (CH^{Ar}), 137.4 (CH^{Ar}), 123.0 (C^{Ar-q}), 120.4 (q, C-F_3, $^1J_{C-F}$ = 332.1 Hz), 119.4 (q, C-F_3, $^1J_{C-F}$ = 333.0 Hz), 117.6 (CH^{Ar}), 117.3 (=CH_2), 96.0 (CTf_2), 84.9 (C^{Ar-q}), 75.8 (C^{Cq}), 60.9 (CH), 52.6 (OCH_3), 40.1 (CH_2), 19.9 (CH_3); ^{19}F NMR (282 MHz, $CHCl_3$, 25 ºC): δ = –67.5 (s, 3F, CF_3), –69.7 (s, 3F, CF_3); IR ($CHCl_3$): ν = 1701 (C=O), 1389, 1203 (O=S=O), 1210 (C–F) cm^{-1}; HRMS (ES): calcd for $C_{17}H_{18}F_6IN_2O_6S_2$ [*M* + NH_4]$^+$: 650.95497; found: 650.95454. HRMS (ESI) m/z: [M + NH_4]$^+$ Calcd for $C_{17}H_{18}F_6IN_2O_6S_2$ 650.9550; Found 650.9545.

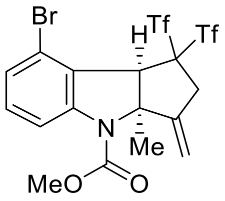

3k

Bis(triflyl)-decorated tricyclic indoline 3k. From 36 mg (0.11 mmol) of allenol **2k**, and after flash chromatography of the residue using hexanes/ethyl acetate (9:1) as eluent gave compound **3k** (33 mg, 49%) as a colorless oil; ^{1}H NMR (500 MHz, $CHCl_3$, 25 ºC): δ = 7.70 (d, 1H, *J* = 7.2 Hz, CH^{Ar}), 7.27 (m, 2H, 2CH^{Ar}), 5.66 (d, 1H, *J* = 2.5 Hz, =C*H*H), 5.59 (d, 1H, *J* = 2.5 Hz, =CH*H*), 4.57 (s, 1H, CH), 3.92 (d, 1H, *J* = 17.3 Hz, C*H*H), 3.85 (s, 3H, OCH_3), 3.69 (d, 1H, *J* = 17.3 Hz, CH*H*), 1.62 (s, 3H, CH_3); ^{13}C NMR (125 MHz, $CHCl_3$, 25 ºC): δ = 152.5 (C=O), 145.6 (C^{Ar-q}), 141.2 (*C*=CH_2), 132.4 (CH^{Ar}), 127.6 (CH^{Ar}), 122.7 (C^{Ar-q}), 122.1 (C^{Ar-q}), 119.9 (q, C-F_3, $^1J_{C-F}$ = 332.4 Hz), 119.8 (q, C-F_3, $^1J_{C-F}$ = 334.0 Hz), 117.8 (=CH_2), 114.2 (CH^{Ar}), 98.3 (CTf_2), 76.1 (C^{Cq}), 62.1 (CH), 52.6 (OCH_3), 40.3 (CH_2), 18.2 (CH_3); ^{19}F NMR (282 MHz, $CHCl_3$, 25 ºC): δ = –68.5 (s, 3F, CF_3), –68.9 (s, 3F, CF_3); IR ($CHCl_3$): ν = 1710 (C=O), 1387, 1198 (O=S=O), 1211 (C–F) cm^{-1}; HRMS (ESI) m/z: [M + H]$^+$ Calcd for $C_{17}H_{15}BrF_6NO_6S_2$ 587.9403; Found 587.9405.

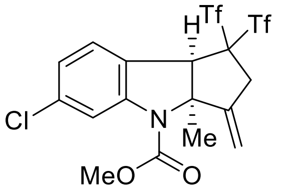

3l

Bis(triflyl)-decorated tricyclic indoline 3l. From 32 mg (0.12 mmol) of allenol **2l**, and after flash chromatography of the residue using hexanes/diethyl ethyl (8:2) as eluent gave compound **3l** (36 mg, 56%) as a colorless oil; ^{1}H NMR (500 MHz, $CHCl_3$, 25 ºC): δ = 7.76 (br s, 1H, CH^{Ar}), 7.35 (d, 1H, J = 8.1 Hz, CH^{Ar}), 7.06 (d, 1H, J = 8.1 Hz, CH^{Ar}), 5.61 (s, 1H, =C*H*H), 5.58 (s, 1H, =CH*H*), 4.48 (s, 1H, CH), 3.86 (m, 4H, OCH_3, C*H*H), 3.71 (d, 1H, J = 17.3 Hz, CH*H*), 1.65 (s, 3H, CH_3); ^{13}C NMR (125 MHz, $CHCl_3$, 25 ºC): δ = 152.5 (C=O), 144.8 (C^{Ar-q}), 141.7 (*C*=CH_2), 137.0 (C^{Ar-q}), 129.4 (CH^{Ar}), 123.1 (CH^{Ar}), 120.4 (q, C-F_3, $^{1}J_{C-F}$ = 332.1 Hz), 119.4 (q, C-F_3, $^{1}J_{C-F}$ = 333.0 Hz), 119.1 (C^{Ar-q}), 117.4 (=CH_2), 116.2 (CH^{Ar}), 95.9 (CTf_2), 76.3 (C^{Cq}), 61.1 (CH), 52.7 (OCH_3), 40.1 (CH_2), 19.9 (CH_3); ^{19}F NMR (282 MHz, $CHCl_3$, 25 ºC): δ = –67.5 (s, 3F, CF_3), –69.7 (s, 3F, CF_3); IR ($CHCl_3$): ν = 1706 (C=O), 1388, 1189 (O=S=O), 1201 (C–F) cm^{-1}; HRMS (ESI) m/z: $[M + NH_4]^+$ Calcd for $C_{17}H_{18}ClF_6N_2O_6S_2$ 559.0193; Found 559.0171.

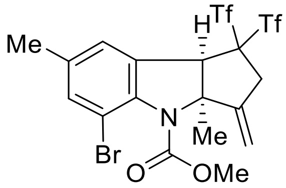

3m

Bis(triflyl)-decorated tricyclic indoline 3m. From 35 mg (0.11 mmol) of allenol **2m**, and after flash chromatography of the residue using hexanes/diethyl ethyl (9:1) as eluent gave compound **3m** (25 mg, 40%) as a colorless oil; ^{1}H NMR (500 MHz, $CHCl_3$, 25 ºC): δ = 7.37 (s, 1H, CH^{Ar}), 7.19 (s, 1H, CH^{Ar}), 5.61 (s, 2H, =CH_2), 4.26 (s, 1H, CH), 3.80 (s, 3H, OCH_3), 3.74 (m, 2H, CH_2), 2.33 (s, 3H, CH_3), 1.59 (s, 3H, CH_3); ^{13}C NMR (125 MHz, $CHCl_3$, 25 ºC): δ = 151.5 (C=O), 140.8 (C^{Ar-q}), 140.6 (*C*=CH_2), 135.9 (CH^{Ar}), 135.0 (C^{Ar-q}), 128.6 (CH^{Ar}), 126.9 (C^{Ar-q}), 120.4 (q, C-F_3, $^{1}J_{C-F}$ = 332.0 Hz),

119.4 (q, C-F3, ${}^{1}J_{C\text{-}F}$ = 333.0 Hz), 118.1 (=CH_2), 111.3 ($C^{Ar\text{-}q}$), 95.6 (CTf_2), 77.8 (C^{Cq}), 61.5 (CH), 52.7 (OCH_3), 40.6 (CH_2), 21.4 (CH_3), 20.6 (CH_3); ^{19}F NMR (282 MHz, $CHCl_3$, 25 °C): δ = –67.6 (s, 3F, CF_3), –69.6 (s, 3F, CF_3); IR ($CHCl_3$): ν = 1699 (C=O), 1391, 1203 (O=S=O), 1213 (C–F) cm^{-1}; HRMS (ESI) m/z: $[M + H]^+$ Calcd for $C_{18}H_{17}BrF_6NO_6S_2$ 601.9559; Found 601.9556.

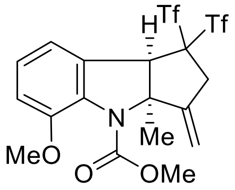

3n

Bis(triflyl)-decorated tricyclic indoline 3n. From 30 mg (0.11 mmol) of allenol **2n**, and after flash chromatography of the residue using hexanes/ethyl acetate (85:15→7:3) as eluent gave compound **3n** (39 mg, 65%) as a colorless oil; ^{1}H NMR (500 MHz, $CHCl_3$, 25 °C): δ = 7.07 (m, 2H, $2CH^{Ar}$), 6.95 (m, 1H, CH^{Ar}), 5.59 (m, 2H, =CH_2), 4.31 (s, 1H, CH), 3.86 (s, 3H, OCH_3), 3.81 (d, 1H, *J* = 18.2 Hz, C*H*H), 3.77 (s, 3H, OCH_3), 3.73 (d, 1H, *J* = 17.4 Hz, CH*H*), 1.61 (s, 3H, CH_3); ^{13}C NMR (125 MHz, $CHCl_3$, 25 °C): δ = 152.2 (C=O), 148.6 ($C^{Ar\text{-}q}$), 141.0 (*C*=CH_2), 133.0 ($C^{Ar\text{-}q}$), 125.0 ($C^{Ar\text{-}q}$), 124.4 (CH^{Ar}), 120.9 ($C^{Ar\text{-}q}$), 120.4 (q, C-F3, ${}^{1}J_{C\text{-}F}$ = 332.0 Hz), 119.4 (q, C-F3, ${}^{1}J_{C\text{-}F}$ = 333.0 Hz), 117.6 (=CH_2), 114.3 (CH^{Ar}), 95.8 (CTf_2), 77.6 (C^{Cq}), 61.7 (CH), 56.0 (OCH_3), 52.6 (OCH_3), 40.5 (CH_2), 20.9 (CH_3); ^{19}F NMR (282 MHz, $CHCl_3$, 25 °C): δ = –67.6 (s, 3F, CF_3), –69.7 (s, 3F, CF_3); IR ($CHCl_3$): ν = 1710 (C=O), 1391, 1207 (O=S=O), 1208 (C–F) cm^{-1}; HRMS (ESI) m/z: $[M + Na]^+$ Calcd for $C_{18}H_{17}F_6NO_7S_2Na$ 560.0243; Found 560.0253.

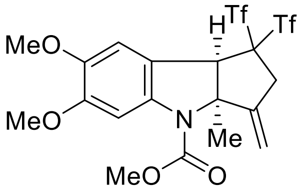

3o

Bis(triflyl)-decorated tricyclic indoline 3o. From 35 mg (0.12 mmol) of allenol **2o**, and after flash chromatography of the residue using hexanes/ethyl acetate (85:15) as eluent gave compound **3o** (27

mg, 41%) as a pale yellow oil, containing *ca.* 12% (^{1}H NMR spectroscopy) of a rotamer; ^{1}H NMR (500 MHz, $CHCl_3$, 25 °C): δ = 7.44 (br s, 1H, CH^{Ar}), 6.86 (s, 1H, CH^{Ar}), 5.48 (m, 1H, $=CH_2$), 4.42 (s, 1H, CH), 3.84 (s, 3H, OCH_3), 3.79 (s, 3H, OCH_3), 3.75 (m, 4H, OCH_3, C*H*H), 3.60 (d, 1H, *J* = 17.3 Hz, CH*H*), 1.57 (s, 3H, CH_3); ^{13}C NMR (125 MHz, $CHCl_3$, 25 °C): δ = 152.8 ($C^{Ar\text{-}q}$), 151.2 (C=O), 150.6 ($C^{Ar\text{-}q}$), 145.0 ($C^{Ar\text{-}q}$), 142.3 (*C*=CH_2), 120.4 (q, C-F_3, $^{1}J_{C\text{-}F}$ = 332.1 Hz), 119.4 (q, C-F_3, $^{1}J_{C\text{-}F}$ = 333.0 Hz), 116.8 ($=CH_2$), 111.5 (CH^{Ar}), 110.4 ($C^{Ar\text{-}q}$), 100.1 (CH^{Ar}), 95.9 (CTf_2), 75.9 (C^{Cq}), 62.4 (CH), 56.3 (OCH_3), 55.9 (OCH_3), 52.2 (OCH_3), 40.0 (CH_2), 20.1 (CH_3); ^{19}F NMR (282 MHz, $CHCl_3$, 25 °C): δ = –67.7 (s, 3F, CF_3), –69.9 (s, 3F, CF_3); IR ($CHCl_3$): ν = 1703 (C=O), 1387, 1206 (O=S=O), 1213 (C–F) cm^{-1}; HRMS (ESI) m/z: $[M + NH_4]^+$ Calcd for $C_{19}H_{23}F_6N_2O_8S_2$ 585.0794; Found 585.0774.

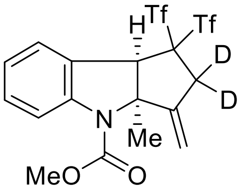

[D]-3a

Bis(triflyl)-decorated tricyclic indoline [D]-3a. From 30 mg (0.13 mmol) of allenol **2a**, and after flash chromatography of the residue using hexanes/ethyl acetate (9:1) as eluent gave compound [D]-**3a** (38 mg, 59%) as a colorless solid; mp 120–122 °C; ^{1}H NMR (500 MHz, $CHCl_3$, 25 °C): δ = 7.75 (br s, 1H, CH^{Ar}), 7.45 (d, 1H, *J* = 7.6 Hz, CH^{Ar}), 7.38 (t, 1H, *J* = 7.9 Hz, CH^{Ar}), 7.09 (t, 1H, *J* = 7.5 Hz, CH^{Ar}), 5.62 (s, 1H, =C*H*H), 5.57 (s, 1H, =CH*H*), 4.53 (s, 1H, CH), 3.84 (s, 3H, OCH_3), 1.65 (s, 3H, CH_3); ^{13}C NMR (125 MHz, $CHCl_3$, 25 °C): δ = 152.8 (C=O), 143.8 ($C^{Ar\text{-}q}$), 141.9 (*C*=CH_2), 131.0 (CH^{Ar}), 128.7 (CH^{Ar}), 123.0 (CH^{Ar}), 120.5 ($C^{Ar\text{-}q}$), 120.4 (q, C-F_3, $^{1}J_{C\text{-}F}$ = 332.2 Hz), 119.4 (q, C-F_3, $^{1}J_{C\text{-}F}$ = 333.1 Hz), 117.1 ($=CH_2$), 115.6 (CH^{Ar}), 96.0 (CTf_2), 75.5 (C^{Cq}), 61.7 (CH), 52.4 (OCH_3), 39.6 (m, CD_2), 19.8 (CH_3); ^{19}F NMR (282 MHz, $CHCl_3$, 25 °C): δ = –67.6 (s, 3F, CF_3), –69.8 (s, 3F, CF_3); D(^{2}H) NMR 107 MHz, $CHCl_3$, 25 °C): δ = 3.86 (s, 1D, C*D*D), 3.71 (s, 1D, CD*D*);

IR ($CHCl_3$): ν = 1701 (C=O), 1392, 1203 (O=S=O), 1210 (C–F) cm^{-1}; HRMS (ESI) m/z: $[M + NH_4]^+$ Calcd for $C_{17}H_{17}D_2F_6N_2O_6S_2$ 527.0709; Found 527.0708.

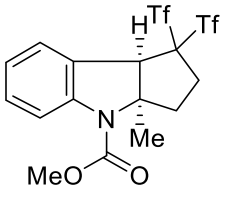

7a

Bis(triflyl)-decorated tricyclic indoline 7a. From 30 mg (0.13 mmol) of alkenol **6a**, and after flash chromatography of the residue using hexanes/ethyl acetate (9:1) as eluent gave compound **7a** (43 mg, 64%) as a colorless oil; ^{1}H NMR (500 MHz, $CHCl_3$, 25 ºC): δ = 7.74 (br s, 1H, CH^{Ar}), 7.46 (d, 1H, J = 7.6 Hz, CH^{Ar}), 7.38 (t, 1H, J = 7.9 Hz, CH^{Ar}), 7.10 (t, 1H, J = 7.5 Hz, CH^{Ar}), 4.51 (s, 1H, CH), 3.93 (s, 3H, OCH_3), 3.43 (br s, 1H, C*H*H), 3.11 (m, 2H, CH_2), 2.43 (m, 1H, CH*H*), 1.60 (s, 3H, CH_3); ^{13}C NMR (125 MHz, $CHCl_3$, 25 ºC): δ = 153.1 (C=O), 143.8 ($C^{Ar\text{-}q}$), 130.9 (CH^{Ar}), 128.7 (CH^{Ar}), 122.9 (CH^{Ar}), 120.6 ($C^{Ar\text{-}q}$), 120.5 (q, C-F_3, $^1J_{C\text{-}F}$ = 332.1 Hz), 119.5 (q, C-F_3, $^1J_{C\text{-}F}$ = 333.4 Hz), 115.7 (CH^{Ar}), 99.9 (CTf_2), 77.8 (C^{Cq}), 61.8 (CH), 52.7 (OCH_3), 35.7 (CH_2), 33.7 (CH_2), 23.0 (CH_3); ^{19}F NMR (282 MHz, $CHCl_3$, 25 ºC): δ = –67.3 (s, 3F, CF_3), –69.4 (s, 3F, CF_3); IR ($CHCl_3$): ν = 1705 (C=O), 1391, 1213 (O=S=O), 1210 (C–F) cm^{-1}; HRMS (ESI) m/z: $[M + NH_4]^+$ Calcd for $C_{16}H_{19}F_6N_2O_6S_2$ 513.0583; Found 513.0604.

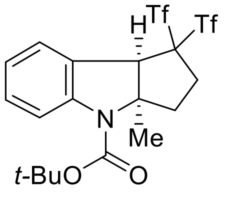

7b

Bis(triflyl)-decorated tricyclic indoline 7b. From 30 mg (0.11 mmol) of alkenol **6b**, and after flash chromatography of the residue using hexanes/ethyl acetate (95:5) as eluent gave compound **7b** (38 mg, 63%) as a colorless oil; ^{1}H NMR (500 MHz, $CHCl_3$, 25 ºC): δ = 7.77 (br s, 1H, CH^{Ar}), 7.43 (d, 1H, J = 7.6 Hz, CH^{Ar}), 7.34 (t, 1H, J = 7.9 Hz, CH^{Ar}), 7.05 (t, 1H, J = 7.6, 0.7 Hz, CH^{Ar}), 4.48 (s, 1H,

CH), 3.38 (br s, 1H, C*H*H), 3.08 (m, 2H, CH_2), 2.40 (m, 1H, CH*H*), 1.61 (s, 9H, $3CH_3$), 1.58 (s, 3H, CH_3); ^{13}C NMR (125 MHz, $CHCl_3$, 25 ºC): δ = 151.6 (C=O), 144.4 (C^{Ar-q}), 130.8 (CH^{Ar}), 128.5 (CH^{Ar}), 122.5 (CH^{Ar}), 120.5 (q, C-F_3, $^1J_{C-F}$ = 332.0 Hz), 120.2 (C^{Ar-q}), 119.5 (q, C-F_3, $^1J_{C-F}$ = 333.4 Hz), 115.7 (CH^{Ar}), 100.1 (CTf_2), 82.4 (C^{Cq}), 61.8 (CH), 36.2 (CH_2), 33.6 (CH_2), 28.4 ($3CH_3$), 23.3 (CH_3); ^{19}F NMR (282 MHz, $CHCl_3$, 25 ºC): δ = –67.3 (s, 3F, CF_3), –69.4 (s, 3F, CF_3); IR ($CHCl_3$): ν = 1699 (C=O), 1388, 1207 (O=S=O), 1208 (C–F) cm^{-1}; HRMS (ESI) m/z: $[M + NH_4]^+$ Calcd for $C_{19}H_{25}F_6N_2O_6S_2$ 555.1053; Found 555.1079.

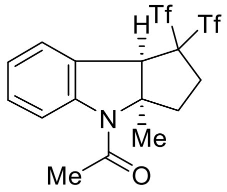

7c

Bis(triflyl)-decorated tricyclic indoline 7c. From 30 mg (0.14 mmol) of alkenol **6c**, and after flash chromatography of the residue using hexanes/ethyl acetate (9:1→8:2) as eluent gave compound **7c** (24 mg, 34%) as a colorless oil; ^1H NMR (500 MHz, $CHCl_3$, 25 ºC): δ = 7.50 (d, 1H, *J* = 7.6 Hz, CH^{Ar}), 7.37 (m, 1H, CH^{Ar}), 7.12 (m, 2H, $2CH^{Ar}$), 4.34 (s, 1H, CH), 3.60 (dd, 1H, *J* = 14.5, 6.4 Hz, C*H*H), 3.14 (dd, 1H, *J* = 15.6, 7.4 Hz, C*H*H), 2.96 (m, 1H, CH*H*), 2.46 (s, 3H, CH_3), 2.38 (m, 1H, CH*H*), 1.56 (s, 3H, CH_3); ^{13}C NMR (125 MHz, $CHCl_3$, 25 ºC): δ = 168.8 (C=O), 144.1 (C^{Ar-q}), 130.6 (CH^{Ar}), 129.6 (CH^{Ar}), 123.4 (CH^{Ar}), 123.0 (C^{Ar-q}), 120.5 (q, C-F_3, $^1J_{C-F}$ = 331.9 Hz), 119.5 (q, C-F_3, $^1J_{C-F}$ = 333.2 Hz), 115.2 (CH^{Ar}), 99.8 (CTf_2), 79.6 (C^{Cq}), 61.2 (CH), 34.7 (CH_2), 34.0 (CH_2), 25.8 (CH_3), 22.9 (CH_3); ^{19}F NMR (282 MHz, $CHCl_3$, 25 ºC): δ = –67.5 (s, 3F, CF_3), –69.4 (s, 3F, CF_3); IR ($CHCl_3$): ν = 1652 (C=O), 1392, 1203 (O=S=O), 1210 (C–F) cm^{-1}; HRMS (ESI) m/z: $[M + H]^+$ Calcd for $C_{16}H_{16}F_6NO_5S_2$ 480.0369; Found 480.0384.

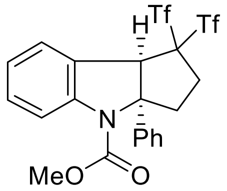

7d

Bis(triflyl)-decorated tricyclic indoline 7d. From 30 mg (0.10 mmol) of alkenol **6d**, and after flash chromatography of the residue using hexanes/ethyl acetate (9:1) as eluent gave compound **7d** (50 mg, 86%) as a colorless solid; mp 192–194 ºC; ^{1}H NMR (500 MHz, $CHCl_3$, 25 ºC): δ = 8.10 (br m, 1H, CH^{Ar}), 7.44 (t, 1H, J = 7.8 Hz, CH^{Ar}), 7.31 (m, 6H, 6CH^{Ar}), 7.09 (t, 1H, J = 7.5 Hz, CH^{Ar}), 4.74 (s, 1H, CH), 3.88 (br s, 4H, OCH_3, C*H*H), 3.38 (m, 1H, CH*H*), 3.26 (m, 2H, CH_2); ^{13}C NMR (125 MHz, $CHCl_3$, 25 ºC): δ = 153.4 (C=O), 145.3 ($C^{Ar\text{-}q}$), 139.3 ($C^{Ar\text{-}q}$), 131.2 (CH^{Ar}), 129.0 (2CH^{Ar}), 128.7 (CH^{Ar}), 128.2 (CH^{Ar}), 124.9 (2CH^{Ar}), 123.3 (CH^{Ar}), 120.5 (q, C-F_3, $^{1}J_{C\text{-}F}$ = 332.1 Hz), 120.4 ($C^{Ar\text{-}q}$), 119.6 (q, C-F_3, $^{1}J_{C\text{-}F}$ = 333.6 Hz), 115.3 (CH^{Ar}), 99.7 (CTf_2), 82.4 (C^{Cq}), 64.0 (CH), 52.9 (OCH_3), 35.9 (CH_2), 34.0 (CH_2); ^{19}F NMR (282 MHz, $CHCl_3$, 25 ºC): δ = –67.0 (s, 3F, CF_3), –69.0 (s, 3F, CF_3); IR ($CHCl_3$): ν = 1707 (C=O), 1396, 1205 (O=S=O), 1205 (C–F) cm^{-1}; HRMS (ESI) m/z: $[M + NH_4]^+$ Calcd for $C_{21}H_{21}F_6N_2O_6S_2$ 575.0740; Found 575.0744.

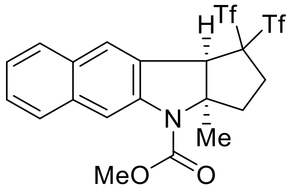

7e

Bis(triflyl)-decorated tricyclic indoline 7e. From 30 mg (0.11 mmol) of alkenol **6e**, and after flash chromatography of the residue using hexanes/ethyl acetate (9:1) as eluent gave compound **7e** (31 mg, 52%) as a colorless oil; ^{1}H NMR (500 MHz, $CHCl_3$, 25 ºC): δ = 8.09 (br s, 1H, CH^{Ar}), 7.89 (s, 1H, CH^{Ar}), 7.80 (m, 2H, 2CH^{Ar}), 7.48 (m, 1H, CH^{Ar}), 7.40 (m, 1H, CH^{Ar}), 4.64 (s, 1H, CH), 3.98 (s, 3H, OCH_3), 3.47 (br s, 1H, C*H*H), 3.14 (m, 2H, CH_2), 2.45 (m, 1H, CH*H*), 1.61 (s, 3H, CH_3); ^{13}C NMR (125 MHz, $CHCl_3$, 25 ºC): δ = 153.3 (C=O), 140.9 ($C^{Ar\text{-}q}$), 135.1 ($C^{Ar\text{-}q}$), 129.9 ($C^{Ar\text{-}q}$), 128.8 (CH^{Ar}),

128.0 (CH^{Ar}), 127.8 (CH^{Ar}), 127.2 (CH^{Ar}), 124.9 (CH^{Ar}), 122.1 ($C^{Ar\text{-}q}$), 120.6 (q, C-F_3, ${}^1J_{C\text{-}F}$ = 331.9 Hz), 119.4 (q, C-F_3, ${}^1J_{C\text{-}F}$ = 333.3 Hz), 112.1 (CH^{Ar}), 100.1 (CTf_2), 77.9 (C^{Cq}), 61.1 (CH), 52.9 (OCH_3), 35.8 (CH_2), 33.7 (CH_2), 23.1 (CH_3); ^{19}F NMR (282 MHz, $CHCl_3$, 25 °C): δ = –67.2 (s, 3F, CF_3), –69.4 (s, 3F, CF_3); IR ($CHCl_3$): ν = 1695 (C=O), 1389, 1205 (O=S=O), 1201 (C–F) cm^{-1}; HRMS (ESI) m/z: $[M + H]^+$ Calcd for $C_{20}H_{18}F_6NO_6S_2$ 546.0474; Found 546.0489.

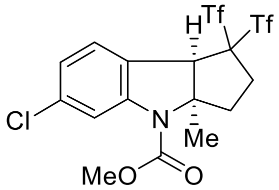

7f

Bis(triflyl)-decorated tricyclic indoline 7f. From 32 mg (0.12 mmol) of alkenol **6f**, and after flash chromatography of the residue using hexanes/ethyl acetate (9:1) as eluent gave compound **7f** (32 mg, 49%) as a colorless oil; ^{1}H NMR (500 MHz, $CHCl_3$, 25 °C): δ = 7.73 (br s, 1H, CH^{Ar}), 7.34 (d, 1H, *J* = 8.2 Hz, CH^{Ar}), 7.05 (dd, 1H, *J* = 8.2, 1.9 Hz, CH^{Ar}), 4.43 (s, 1H, CH), 3.92 (s, 3H, OCH_3), 3.38 (br s, 1H, C*H*H), 3.08 (m, 2H, CH_2), 2.40 (m, 1H, CH*H*), 1.58 (s, 3H, CH_3); ^{13}C NMR (125 MHz, $CHCl_3$, 25 °C): δ = 152.8 (C=O), 144.8 ($C^{Ar\text{-}q}$), 136.9 ($C^{Ar\text{-}q}$), 129.3 (CH^{Ar}), 123.1 (CH^{Ar}), 120.5 (q, C-F_3, ${}^1J_{C\text{-}F}$ = 332.0 Hz), 119.4 (q, C-F_3, ${}^1J_{C\text{-}F}$ = 333.2 Hz), 119.2 ($C^{Ar\text{-}q}$), 116.2 (CH^{Ar}), 99.7 (CTf_2), 78.5 (C^{Cq}), 61.2 (CH), 53.0 (OCH_3), 35.8 (CH_2), 33.6 (CH_2), 23.1 (CH_3); ^{19}F NMR (282 MHz, $CHCl_3$, 25 °C): δ = –67.3 (s, 3F, CF_3), –69.4 (s, 3F, CF_3); IR ($CHCl_3$): ν = 1699 (C=O), 1385, 1203 (O=S=O), 1213 (C–F) cm^{-1}; HRMS (ESI) m/z: $[M + NH_4]^+$ Calcd for $C_{16}H_{18}ClF_6N_2O_6S_2$ 547.0193; Found 547.0203.

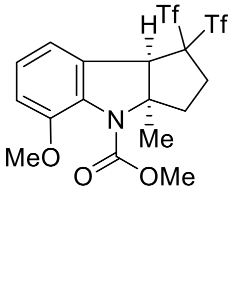

7g

Bis(triflyl)-decorated tricyclic indoline 7g. From 30 mg (0.11 mmol) of alkenol **6g**, and after flash chromatography of the residue using hexanes/ethyl acetate (9:1) as eluent gave compound **7g** (42 mg, 67%) as a colorless solid; mp 114–116 ºC; ^{1}H NMR (500 MHz, $CHCl_3$, 25 ºC): δ = 7.07 (m, 2H, 2CH^{Ar}), 6.94 (dd, 1H, *J* = 7.4, 1.9 Hz, CH^{Ar}), 4.24 (s, 1H, CH), 3.86 (s, 3H, OCH_3), 3.83 (s, 3H, OCH_3), 3.45 (dd, 1H, *J* = 14.8, 6.6 Hz, C*H*H), 3.17 (dd, 1H, *J* = 15.6, 7.2 Hz, C*H*H), 2.96 (m, 1H, CH*H*), 2.31 (m, 1H, CH*H*), 1.51 (s, 3H, CH_3); ^{13}C NMR (125 MHz, $CHCl_3$, 25 ºC): δ = 152.9 (C=O), 148.8 (C$^{Ar\text{-}q}$), 133.1 (C$^{Ar\text{-}q}$), 125.3 (C$^{Ar\text{-}q}$), 124.5 (CH^{Ar}), 120.8 (CH^{Ar}), 120.5 (q, C-F_3, $^1J_{C\text{-}F}$ = 331.9 Hz), 119.5 (q, C-F_3, $^1J_{C\text{-}F}$ = 333.1 Hz), 114.1 (CH^{Ar}), 99.3 (CTf_2), 79.7 (C^{Cq}), 61.6 (CH), 55.9 (OCH_3), 52.8 (OCH_3), 34.4 (CH_2), 33.7 (CH_2), 23.9 (CH_3); ^{19}F NMR (282 MHz, $CHCl_3$, 25 ºC): δ = –67.4 (s, 3F, CF_3), –69.5 (s, 3F, CF_3); IR ($CHCl_3$): ν = 1707 (C=O), 1393, 1206 (O=S=O), 1201 (C–F) cm^{-1}; HRMS (ESI) m/z: $[M + H]^+$ Calcd for $C_{17}H_{18}F_6NO_7S_2$ 526.0423; Found 526.0449.

Procedure for the preparation of tricyclic dienyl triflone 8.

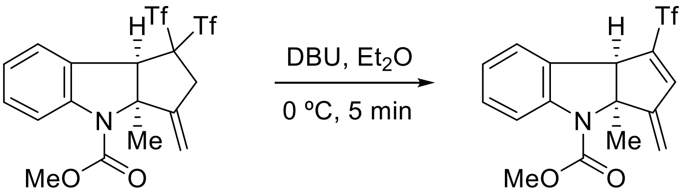

3a **8**

To a stirred mixture of bis(triflyl)-decorated tricyclic indoline **3a** (1.0 mmol) in diethyl ether (20 mL) cooled at 0 ºC, was added DBU (1.0 mmol). The mixture was stirred at 0 ºC until complete consumption of starting material (5 min) as monitored by TLC. This reaction mixture was transferred directly to a chromatography column filled with silica gel and was purified by column chromatography to provide product **8**. *Note: It is very important to be rigorous with the amount of DBU used, because small excesses quickly reduce the yield. Besides, it is convenient to avoid the concentration of the crude.*

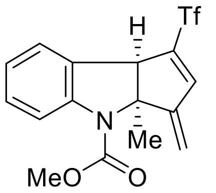

Tricyclic dienyl triflone 8. From 38 mg (0.07 mmol) of bis(triflyl)-decorated tricyclic indoline **3a**, and after flash chromatography of the residue using hexanes/ethyl acetate (9:1) as eluent gave compound **8** (25 mg, 91%) as a colorless oil; ^{1}H NMR (500 MHz, $CHCl_3$, 25 ºC): δ = 7.71 (br s, 1H, CH^{Ar}), 7.60 (d, 1H, J = 7.6 Hz, CH^{Ar}), 7.37 (s, 1H, =CH), 7.28 (t, 1H, J = 7.5 Hz, CH^{Ar}), 7.05 (t, 1H, J = 7.5 Hz, CH^{Ar}), 6.29 (br s, 1H, =C*H*H), 5.92 (s, 1H, =CH*H*), 4.47 (s, 1H, CH), 3.89 (s, 3H, OCH_3), 1.82 (s, 3H, CH_3); ^{13}C NMR (125 MHz, $CHCl_3$, 25 ºC): δ = 153.4 (=CH), 153.2 (C=O), 150.5 (TfC=), 141.6 (*C*=CH_2), 136.3 ($C^{Ar\text{-}q}$), 129.4 (CH^{Ar}), 127.0 (CH^{Ar}), 125.3 ($C^{Ar\text{-}q}$), 125.2 (=CH_2-*low intensity signal*), 123.2 (CH^{Ar}), 119.5 (q, C-F_3, $^{1}J_{C\text{-}F}$ = 326.6 Hz), 115.6 (CH^{Ar}), 74.5 (C^{Cq}), 60.3 (CH), 52.4 (OCH_3), 22.9 (CH_3); ^{19}F NMR (282 MHz, $CHCl_3$, 25 ºC): δ = –78.0 (s, 3F, CF_3); IR ($CHCl_3$): ν = 1715 (C=O), 1342, 1212 (O=S=O), 1205 (C–F) cm^{-1}; HRMS (ESI) m/z: $[M + H]^{+}$ Calcd for $C_{16}H_{15}F_3NO_4S$ 374.0668; Found 374.0671.

Procedure for the preparation of bromo-bis(triflyl)-decorated tricyclic indoline 9.

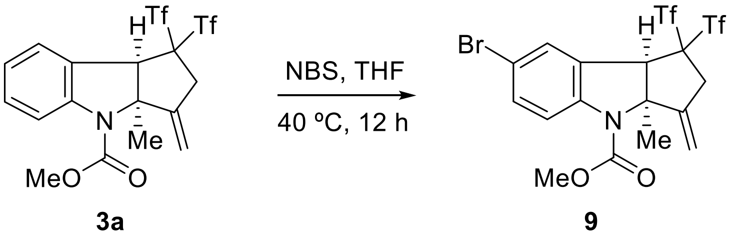

To a stirred mixture of bis(triflyl)-decorated tricyclic indoline **3a** (1.0 mmol) in THF (20 ml) was added NBS (3.0 mmol), and then the reaction was heated at 40 ºC until complete consumption of starting material as monitored by TLC. The reaction mixture was concentrated under vacuum and the crude product was purified by column chromatography to provide product **9**.

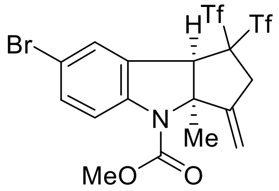

9

Bromo-bis(triflyl)-decorated tricyclic indoline 9. From 27 mg (0.05 mmol) of bis(triflyl)-decorated tricyclic indoline **3a**, and after flash chromatography of the residue using hexanes/ethyl acetate (9:1) as eluent gave compound **9** (30 mg, 97%) as a colorless solid; mp 142–144 ºC; ^{1}H NMR (500 MHz, $CHCl_3$, 25 ºC): δ = 7.64 (br s, 1H, CH^{Ar}), 7.53 (s, 1H, CH^{Ar}), 7.47 (dd, 1H, *J* = 8.8, 2.0 Hz, CH^{Ar}), 5.60 (s, 1H, =C*H*H), 5.57 (d, 1H, *J* = 2.3 Hz, =CH*H*), 4.47 (s, 1H, CH), 3.85 (m, 4H, C*H*H, OCH_3), 3.71 (d, 1H, *J* = 17.3 Hz, CH*H*), 1.64 (s, 3H, CH_3); ^{13}C NMR (125 MHz, $CHCl_3$, 25 ºC): δ = 152.6 (C=O), 143.0 (C^{Ar-q}), 141.7 (*C*=CH_2), 133.9 (CH^{Ar}), 131.5 (CH^{Ar}), 122.7 (C^{Ar-q}), 120.4 (q, C-F_3, $^{1}J_{C-F}$ = 332.0 Hz), 119.4 (q, C-F_3, $^{1}J_{C-F}$ = 333.0 Hz), 117.3 (=CH_2), 117.0 (CH^{Ar}), 115.1 (C^{Ar-q}), 96.0 (CTf_2), 75.9 (C^{Cq}), 61.1 (CH), 52.6 (OCH_3), 40.1 (CH_2), 19.9 (CH_3); ^{19}F NMR (282 MHz, $CHCl_3$, 25 ºC): δ = –67.5 (s, 3F, CF_3), –69.7 (s, 3F, CF_3); IR ($CHCl_3$): ν = 1699 (C=O), 1399, 1206 (O=S=O), 1217 (C–F) cm^{-1}; HRMS (ESI) m/z: $[M + H]^+$ Calcd for $C_{17}H_{15}BrF_6NO_6S_2$ 585.9423; Found 585.9447.

Procedure for the preparation of bis(triflyl)ethyl-decorated bicyclic indoline 10.

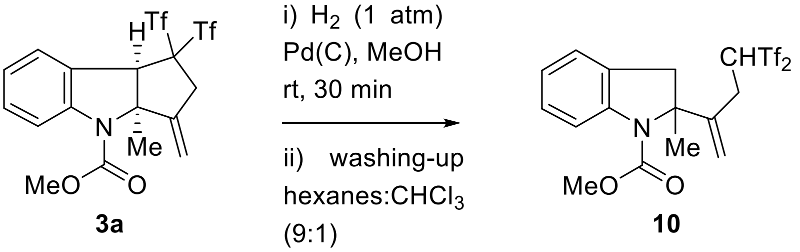

A mixture of bis(triflyl)-decorated tricyclic indoline **3a** (1.0 mmol) and Pd/C (10 mol %) in methanol (20 ml) was stirred at rt under an atmosphere of hydrogen (1 atm) until complete consumption of starting material as monitored by TLC. The reaction mixture was filtered through a Celite pad and

concentrated under reduced pressure. The crude semisolid residue was washing up 3 times with a hexane:$CHCl_3$ (9:1) mixture and then was vacuum dried to afford product **10**.

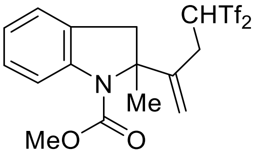

10

Bis(triflyl)ethyl-decorated bicyclic indoline 10. From 42 mg (0.08 mmol) of bis(triflyl)-decorated tricyclic indoline **3a**, and after washing up 3 times with a hexane:$CHCl_3$ (9:1) mixture gave compound **10** (31 mg, 75%) as a colorless solid; mp 89–91 ºC; ^{1}H NMR (500 MHz, $CHCl_3$, 25 ºC): δ = 7.75(br s, 1H, CH^{Ar}), 7.24 (t, 1H, J = 7.8 Hz, CH^{Ar}), 7.16 (d, 1H, J = 7.3 Hz, CH^{Ar}), 7.04 (t, 1H, J = 7.4 Hz, CH^{Ar}), 5.47 (br m, 1H, $CHTf_2$) 5.42 (s, 1H, =C*H*H), 5.11 (s, 1H, =CH*H*), 3.82 (s, 3H, OCH_3), 3.30 (d, 1H, J = 16.8 Hz, C*H*H), 3.25 (dd, 1H, J = 18.6, 6.2 Hz, C*H*H), 3.14 (dd, 1H, J = 17.9, 2.5 Hz, CH*H*), 3.05 (d, 1H, J = 16.8 Hz, CH*H*), 1.75 (s, 3H, CH_3); ^{13}C NMR (125 MHz, $CHCl_3$, 25 ºC): δ = 153.9 (C=O), 145.7 (*C*=CH_2), 141.5 ($C^{Ar\text{-}q}$), 128.1 (CH^{Ar}), 127.7 ($C^{Ar\text{-}q}$), 124.8 (CH^{Ar}), 123.4 (CH^{Ar}), 119.3 (q, C-F_3, $^1J_{C\text{-}F}$ = 330.2 Hz), 119.2 (q, C-F_3, $^1J_{C\text{-}F}$ = 329.9 Hz), 115.9 (CH^{Ar}), 112.6 (=CH_2-*low intensity signal*), 77.2 (*C*HTf_2), 69.3 (C^{Cq}), 52.5 (OCH_3), 44.2 (CH_2), 26.1 (CH_2), 24.9 (CH_3); ^{19}F NMR (282 MHz, $CHCl_3$, 25 ºC): δ = –71.8 (s, 3F, CF_3), –73.1 (s, 3F, CF_3); IR ($CHCl_3$): ν = 1687 (C=O), 1393, 1203 (O=S=O), 1208 (C–F) cm^{-1}; HRMS (ESI) m/z: $[M + Na]^+$ Calcd for $C_{17}H_{17}F_6NO_6S_2Na$ 532.0294; Found 532.0293.

Procedure for the preparation of bis(triflyl)ethyl-decorated bicyclic indoline 11.

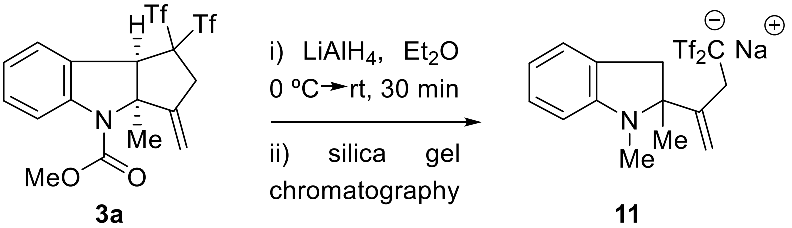

3a **11**

A round bottom flask equipped with a magnetic stir bar was charged with bis(triflyl)-decorated tricyclic indoline **3a** (1.0 mmol) and anhydrous diethyl ether (20 mL). $LiAlH_4$ (5.0 mmol) was added portionwise at 0 °C. The reaction mixture was warmed up to room temperature, and stirred until complete consumption of starting material as monitored by TLC. After then the reaction was quenched; water was added at 0 ºC and the mixture was extracted with AcOEt (3 x 20 mL). The combined organic layer was washed with brine and dried over $MgSO_4$. The solvent was removed under reduced pressure to afford the crude product, which was purified by column chromatography to give product **11**.

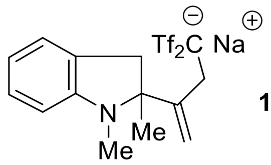

11

Bis(triflyl)ethyl-decorated bicyclic indoline 11. From 46 mg (0.09 mmol) of bis(triflyl)-decorated tricyclic indoline **3a**, and after flash chromatography of the residue using hexanes/ethyl acetate (1:1) as eluent gave compound **11** (27 mg, 63%) as a colorless oil; ^{1}H NMR (500 MHz, acetone-d$_6$, 25 ºC): δ = 6.91 (m, 2H, 2CH^{Ar}), 6.44 (t, 1H, J = 7.1 Hz, CH^{Ar}), 6.23 (d, 1H, J = 7.7 Hz, CH^{Ar}), 5.40 (s, 1H, =C*H*H), 5.12 (d, 1H, J = 1.4 Hz, =CH*H*), 3.14 (d, 1H, J = 15.8 Hz, C*H*H), 3.11 (m, 1H, C*H*H), 3.00 (d, 1H, J = 19.2 Hz, CH*H*), 2.58 (d, 1H, J = 15.9 Hz, CH*H*), 2.49 (s, 3H, NCH_3), 1.21 (s, 3H, CH_3); ^{13}C NMR (125 MHz, acetone-d$_6$, 25 ºC): δ = 152.7 (*C*=CH_2), 151.5 (C$^{Ar\text{-}q}$), 128.5 (C$^{Ar\text{-}q}$), 128.1 (CH^{Ar}), 124.7 (CH^{Ar}), 122.6 (q, 2C-F_3, $^1J_{C\text{-}F}$ = 329.8 Hz), 117.0 (CH^{Ar}), 112.3 (=CH_2), 106.1 (CH^{Ar}), 71.1 (C^{Cq}), 63.0 (CTf_2), 42.6 (CH_2), 31.2 (CH_2), 28.8 (NCH_3), 21.1 (CH_3); ^{19}F NMR (282 MHz, acetone-d$_6$, 25 ºC): δ = –79.1 (s, 6F, $2CF_3$); IR (acetone): ν = 1645 (C=C), 1396, 1207 (O=S=O), 1210 (C–F) cm^{-1}; HRMS (ESI) m/z: [M]$^-$ Calcd for $C_{16}H_{16}F_6NO_4S_2$ 464.0430; Found 464.0430.

Procedure for the preparation of NH-free bis(triflyl)-decorated tricyclic indoline 12.

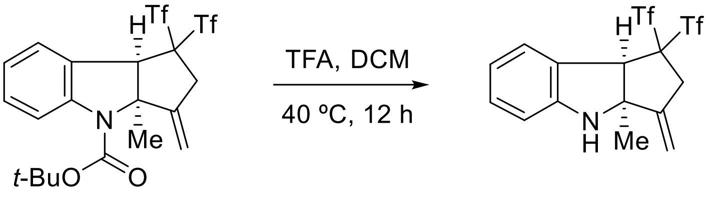

3c **12**

To a stirred solution of bis(triflyl)-decorated tricyclic indoline **3c** (1.0 mmol) in dichloromethane (20 ml) was added trifluoroacetic acid (15.0 mmol), and then the reaction was heated at 40 ºC until complete consumption of starting material (12 h) as monitored by TLC. The mixture was allowed to warm to rt and saturated aqueous sodium hydrogen carbonate (10 mL) was added before being partitioned between dichloromethane and water. The aqueous phase was extracted with dichloromethane (3 x 10 mL). The combined organic extract was washed with brine, dried ($MgSO_4$), and concentrated under reduced pressure. The resulting crude residue was purified by column chromatography to give product **12**.

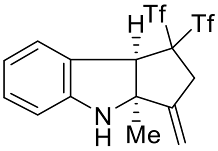

12

NH-free bis(triflyl)-decorated tricyclic indoline 12. From 30 mg (0.05 mmol) of bis(triflyl)-decorated tricyclic indoline **3c**, and after flash chromatography of the residue using hexanes/ethyl acetate (9:1) as eluent gave compound **12** (21 mg, 87%) as a colorless oil; ^{1}H NMR (500 MHz, $CHCl_3$, 25 ºC): δ = 7.37 (d, 1H, J = 7.6 Hz, CH^{Ar}), 7.21 (t, 1H, J = 7.7 Hz, CH^{Ar}), 6.83 (t, 1H, J = 7.5 Hz, CH^{Ar}), 6.66 (d, 1H, J = 7.9 Hz, CH^{Ar}), 5.27 (d, 1H, J = 2.8 Hz, =C*H*H), 5.25 (d, 1H, J = 2.4 Hz, =CH*H*), 4.51 (s, 1H, CH), 4.06 (d, 1H, J = 17.3 Hz, C*H*H), 3.69 (d, 1H, J = 17.3 Hz, CH*H*), 3.25 (br s, 1H, NH), 1.53 (s, 3H, CH_3); ^{13}C NMR (125 MHz, $CHCl_3$, 25 ºC): δ = 151.3 ($C^{Ar\text{-}q}$), 147.6 (*C*=CH_2), 130.8 (CH^{Ar}), 129.0 (CH^{Ar}), 119.7 ($C^{Ar\text{-}q}$), 119.2 (CH^{Ar}), 120.5 (q, C-F_3, $^{1}J_{C\text{-}F}$ = 332.3 Hz), 119.4 (q, C-F_3, $^{1}J_{C\text{-}F}$ = 333.0 Hz), 109.9 (=CH_2), 109.8 (CH^{Ar}), 96.9 (CTf_2), 73.9 (C^{Cq}), 61.7 (CH), 40.0 (CH_2),

21.6 (CH_3); ^{19}F NMR (282 MHz, $CHCl_3$, 25 ºC): δ = –67.3 (s, 3F, CF_3), –69.9 (s, 3F, CF_3); IR ($CHCl_3$): ν = 3335 (NH), 1388, 1203 (O=S=O), 1214 (C–F) cm^{-1}; HRMS (ESI) m/z: $[M + H]^+$ Calcd for $C_{15}H_{14}F_6NO_4S_2$ 450.0263; Found 450.0250.

Procedure for the preparation of NH-free bis(triflyl)ethyl-decorated bicyclic indoline 13.

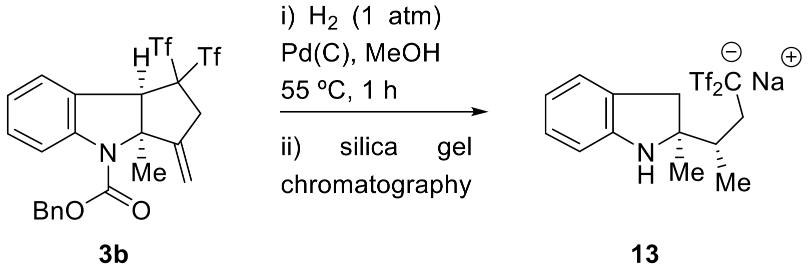

3b **13**

A mixture of bis(triflyl)-decorated tricyclic indoline **3b** (1.0 mmol) and Pd/C (10 mol %) in methanol (20 ml) was stirred at 55 ºC under an atmosphere of hydrogen (1 atm) until complete consumption of starting material (1 h) as monitored by TLC. The reaction mixture was filtered through a Celite pad and concentrated under reduced pressure. The resulting residue was purified by column chromatography to give product **13**.

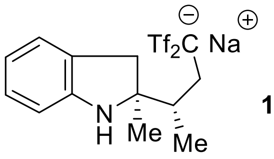

13

NH-free bis(triflyl)ethyl-decorated bicyclic indoline 13. From 25 mg (0.04 mmol) of bis(triflyl)-decorated tricyclic indoline **3b**, and after flash chromatography of the residue using hexanes/ethyl acetate (1:1) as eluent gave compound **13** (11 mg, 55%) as a colorless oil; ^{1}H NMR (500 MHz, acetone-d_6, 25 ºC): δ = 6.93 (d, 1H, *J* = 7.2 Hz, CH^{Ar}), 6.82 (t, 1H, *J* = 7.6 Hz, CH^{Ar}), 6.45 (m, 2H, CH^{Ar}), 2.97 (d, 1H, *J* = 15.6 Hz, C*H*H), 2.65 (m, 1H, C*H*H), 2.64 (d, 1H, *J* = 15.6 Hz, CH*H*), 2.02 (m, 1H, CH*H*), 1.96 (m, 1H, CH), 1.03 (s, 3H, CH_3), 0.99 (d, 1H, *J* = 6.2 Hz, CH_3); ^{13}C NMR (125 MHz, acetone-d_6, 25 ºC): δ = 151.8 (C^{Ar-q}), 129.0 (C^{Ar-q}), 127.8 (CH^{Ar}), 125.5 (CH^{Ar}), 122.8 (q, 2C-

F_3, $^1J_{C-F}$ = 330.7 Hz), 117.9 (CH^{Ar}), 109.5 (CH^{Ar}), 67.5 (C^{Cq}), 64.8 (CTf_2), 44.9 (CH), 42.3 (CH_2), 31.7 (CH_2), 22.7 (CH_3), 13.9 (CH_3); ^{19}F NMR (282 MHz, acetone-d_6, 25 °C): δ = –79.1 (s, 6F, $2CF_3$); IR (acetone): ν = 3345 (NH), 1395, 1198 (O=S=O), 1205 (C–F) cm^{-1}; HRMS (ESI) m/z: $[M]^-$ Calcd for $C_{15}H_{16}F_6NO_4S_2$ 452.0430; Found 452.0432.

Procedure for the preparation of tricyclic (*tert*-butylamino)methyl-triflone 14.

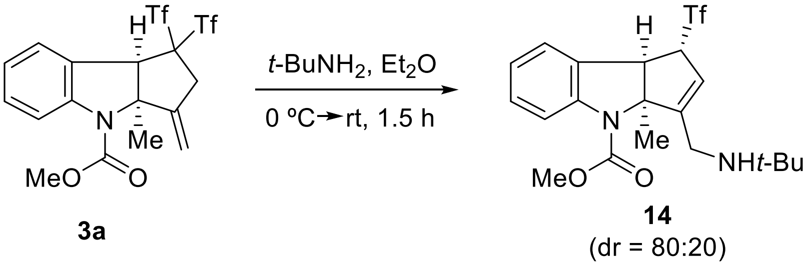

To a stirred mixture of bis(triflyl)-decorated tricyclic indoline **3a** (1.0 mmol) in diethyl ether (20 mL) cooled at 0 °C, was added *tert*-butylamine (3.0 mmol). The mixture was warmed up to rt and stirred until complete consumption of starting material as monitored by TLC. The solvent was removed under reduced pressure to afford the crude product, which was purified by column chromatography to give product **14**.

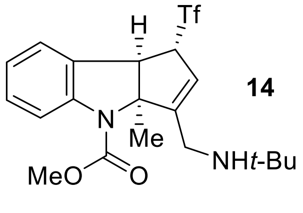

Tricyclic (*tert*-butylamino)methyl-triflone 14. From 26 mg (0.05 mmol) of bis(triflyl)-decorated tricyclic indoline **3a**, and after flash chromatography of the residue using hexanes/ethyl acetate (6:4) as eluent gave compound **14** (19 mg, 84%) as an orange oil, containing *ca.* 20% (^{1}H NMR spectroscopy) of its epimer at the Tf-bearing stereocenter; ^{1}H NMR (500 MHz, $CHCl_3$, 25 °C): δ = 7.72 (br s, 1H, CH^{Ar}), 7.27 (m, 1H, CH^{Ar}), 7.18 (d, 1H, *J* = 7.5 Hz, CH^{Ar}), 7.06 (m, 1H, CH^{Ar}), 5.81 (s, 1H, =CH), 4.45 (s, 1H, CHTf), 4.30 (s, 1H, CH), 3.90 (s, 3H, OCH_3), 3.63 (d, 1H, *J* = 17.9 Hz,

C*H*H), 3.40 (d, 1H, J = 17.9 Hz, CH*H*), 2.00 (s, 3H, CH_3), 1.13 (s, 9H, $3CH_3$); ^{13}C NMR (125 MHz, $CHCl_3$, 25 ºC): δ = 153.7 (C=O), 153.2 (*C*=CH), 129.4 (CH^{Ar}), 128.4 ($C^{Ar\text{-}q}$), 123.8 (CH^{Ar}), 123.2 (CH^{Ar}), 120.1 (q, C-F_3, $^{1}J_{C\text{-}F}$ = 329.3 Hz), 116.7 (CH^{Ar}), 115.6 ($C^{Ar\text{-}q}$), 114.2 (C=*C*H), 79.4 (C^{Cq}), 72.6 (Tf*C*H), 54.4 (CH), 52.5 (OCH_3), 50.6 (C^{Cq}), 41.2 (CH_2), 29.0 ($3CH_3$), 24.4 (CH_3); ^{19}F NMR (282 MHz, $CHCl_3$, 25 ºC): δ = –75.0 (s, 3F, CF_3); IR ($CHCl_3$): ν = 3347 (NH), 1707 (C=O), 1390, 1205 (O=S=O), 1211 (C–F) cm^{-1}; HRMS (ESI) m/z: $[M + H]^+$ Calcd for $C_{20}H_{26}F_3N_2O_4S$ 447.1560; Found 447.1558. *Note: Partial epimerization occurred during chromatographic purification, because just one isomer could be detected in the ^{1}H NMR of the crude material.*

Procedure for the preparation of tricyclic (phenylthio)methyl-triflone 15.

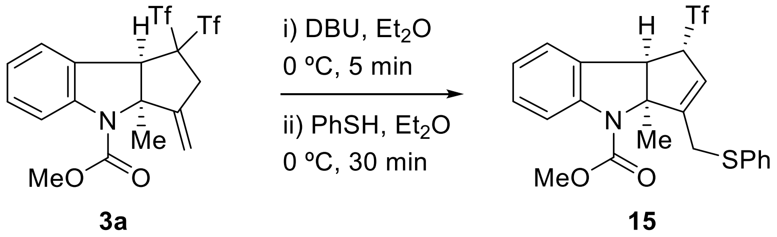

To a stirred mixture of bis(triflyl)-decorated tricyclic indoline **3a** (1.0 mmol) in diethyl ether (20 mL) cooled at 0 ºC, was added DBU (1.0 mmol). The mixture was stirred at 0 ºC until complete consumption of starting material (5 min) as monitored by TLC. The crude was transferred directly to a chromatography column filled with silica gel and was purified to provide product **8**, which was solved in diethyl ether (20 mL) and cooled down to 0 ºC. Then, thiophenol (2.0 mmol) was added and the mixture was stirred at 0 ºC until complete conversion (determined by TLC analysis). The reaction was concentrated in vacuo and purified by flash chromatography on silica gel to afford product **15**.

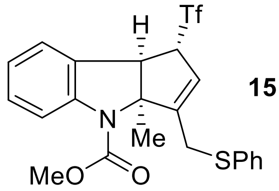

Tricyclic (phenylthio)methyl-triflone 15. From 48 mg (0.09 mmol) of bis(triflyl)-decorated tricyclic indoline **3a**, and after flash chromatography of the residue using hexanes/ethyl acetate (95:5) as eluent gave compound **15** (27 mg, 63%) as a colorless oil; ^{1}H NMR (500 MHz, $CHCl_3$, 25 ºC): δ = 7.76 (br s, 1H, CH^{Ar}), 7.31 (t, 1H, J = 7.8 Hz, CH^{Ar}), 7.20 (m, 5H, $5CH^{Ar}$), 7.16 (d, 1H, J = 7.6 Hz, CH^{Ar}), 7.08 (t, 1H, J = 7.4 Hz, CH^{Ar}), 5.47 (s, 1H, =CH), 4.34 (s, 1H, CHTf), 4.29 (s, 1H, CH), 3.91 (s, 3H, OCH_3), 3.88 (s, 2H, CH_2), 2.07 (s, 3H, CH_3); ^{13}C NMR (125 MHz, $CHCl_3$, 25 ºC): δ = 153.8 (C=O, $C^{Ar\text{-}q}$), 141.4 (*C*=CH), 134.8 ($C^{Ar\text{-}q}$), 130.9 ($2CH^{Ar}$), 129.6 (CH^{Ar}), 128.9 ($2CH^{Ar}$), 128.3 ($C^{Ar\text{-}q}$), 127.0 (CH^{Ar}), 123.8 (CH^{Ar}), 123.7 (CH^{Ar}), 120.0 (q, C-F_3, $^{1}J_{C\text{-}F}$ = 329.4 Hz), 118.5 (C=*C*H), 116.7 (CH^{Ar}), 79.3 (C^{Cq}), 72.2 (Tf*C*H), 54.3 (CH), 52.7 (OCH_3), 32.9 (CH_2), 24.2 (CH_3); ^{19}F NMR (282 MHz, $CHCl_3$, 25 ºC): δ = –75.1 (s, 3F, CF_3); IR ($CHCl_3$): ν = 1703 (C=O), 1395, 1207 (O=S=O), 1200 (C–F) cm^{-1}; HRMS (ESI) m/z: $[M + H]^+$ Calcd for $C_{22}H_{21}F_3NO_4S_2$ 484.0859; Found 484.0828.

Procedure for the preparation of tolyl-bis(triflyl)-decorated tricyclic indoline 16.

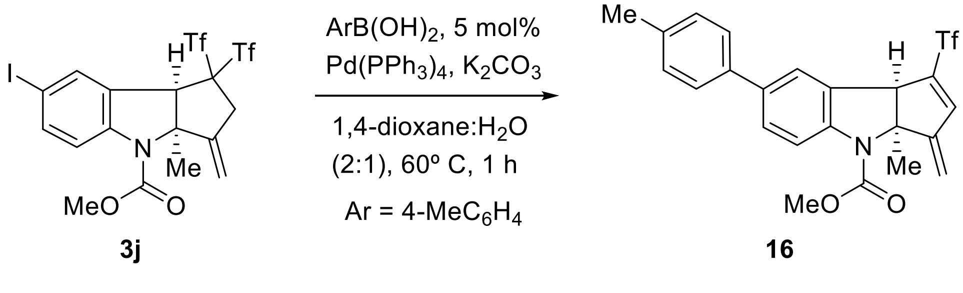

$Pd(PPh_3)_4$ (0.05 mmol, 5.0 mol %) was added to a stirred solution of bis(triflyl)-decorated tricyclic indoline **3j** (1.0 mmol), 4-tolylboronic acid (1.5 mmol) and K_2CO_3 (3.0 mmol) in 1,4-dioxane/water (2:1, 14 mL). The resulting mixture was heated at 60 ºC until disappearance of the starting material (TLC). The reaction was cooled to room temperature, water was added and the mixture was extracted

with AcOEt (3 x 15 mL). The organic phase was washed with water (2 × 5 mL), dried ($MgSO_4$) and concentrated under reduced pressure. The resulting residue was purified by column chromatography to give product **16**.

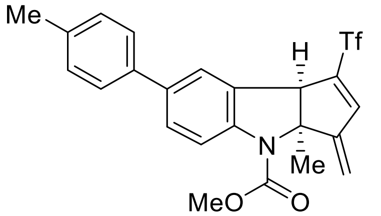

16

Tolyl-bis(triflyl)-decorated tricyclic indoline 16. From 25 mg (0.04 mmol) of bis(triflyl)-decorated tricyclic indoline **3j**, and after flash chromatography of the residue using hexanes/ethyl acetate (9:1) as eluent gave compound **16** (14 mg, 77%) as a pale yellow oil; ^{1}H NMR (500 MHz, $CHCl_3$, 25 °C): δ = 7.84 (s, 1H, CH^{Ar}), 7.70 (br s, 1H, CH^{Ar}), 7.49 (m, 3H, $3CH^{Ar}$), 7.40 (s, 1H, =CH), 7.25 (m, 1H, $2CH^{Ar}$), 6.33 (br s, 1H, =C*H*H), 5.93 (s, 1H, =CH*H*), 4.52 (s, 1H, CH), 3.91 (s, 3H, OCH_3), 2.40 (s, 3H, CH_3), 1.85 (s, 3H, CH_3); ^{13}C NMR (125 MHz, $CHCl_3$, 25 °C): δ = 153.5 (=CH), 153.2 (C=O), 150.5 (TfC=), 140.6 (*C*=CH_2-*low intensity signal*), 137.5 ($C^{Ar\text{-}q}$), 136.8 ($C^{Ar\text{-}q}$), 136.4 ($C^{Ar\text{-}q}$), 129.5 ($2CH^{Ar}$), 128.0 (CH^{Ar}), 126.6 ($2CH^{Ar}$), 125.9 ($C^{Ar\text{-}q}$), 125.3 (CH^{Ar}), 125.2 (=CH_2-*low intensity signal*), 119.6 (q, C-F3, $^{1}J_{C\text{-}F}$ = 326.6 Hz), 115.8 (CH^{Ar}), 74.8 (C^{Cq}), 60.3 (CH), 52.5 (OCH_3), 23.0 (CH_3), 21.1 (CH_3); ^{19}F NMR (282 MHz, $CHCl_3$, 25 °C): δ = –78.0 (s, 3F, CF_3); IR ($CHCl_3$): ν = 1708 (C=O), 1347, 1213 (O=S=O), 1205 (C–F) cm^{-1}; HRMS (ESI) m/z: $[M + NH_4]^+$ Calcd for $C_{23}H_{24}F_3N_2O_4S$ 481.1403; Found 481.1407.

Procedure for the preparation of tetracyclic triflone 17.

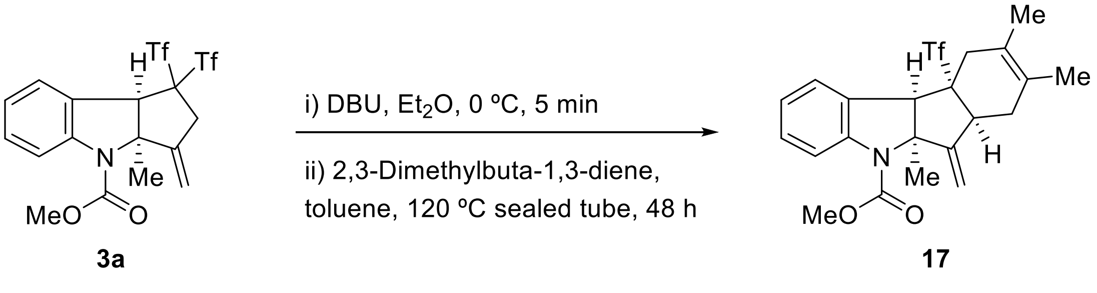

To a stirred mixture of bis(triflyl)-decorated tricyclic indoline **3a** (1.0 mmol) in diethyl ether (20 mL) cooled at 0 ºC, was added DBU (1.0 mmol). The mixture was stirred at 0 ºC until complete consumption of starting material (5 min) as monitored by TLC. The crude was transferred directly to a chromatography column filled with silica gel and was purified to provide product **8**, which was solved in toluene (20 mL). Then, 2,3-dimethyl-1,3-butadiene (3.0 mmol) was added and the mixture was heated at 120 ºC in a sealed tube until complete consumption of starting material as monitored by TLC. The reaction was allowed to cool to room temperature. The mixture was concentrated in vacuo and purified by flash chromatography on silica gel to afford product **17**.

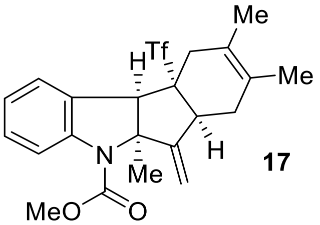

17

Tetracyclic triflone 17. From 65 mg (0.12 mmol) of bis(triflyl)-decorated tricyclic indoline **3a**, and after flash chromatography of the residue using hexanes/ethyl acetate (95:5) as eluent gave compound **17** (27 mg, 45%) as a colorless oil; ^{1}H NMR (500 MHz, $CHCl_3$, 25 ºC): δ = 7.72 (br s, 1H, CH^{Ar}), 7.35 (d, 1H, J = 7.4 Hz, CH^{Ar}), 7.29 (d, 1H, J = 8.3 Hz, CH^{Ar}), 7.07 (td, 1H, J = 7.5, 0.8 Hz, CH^{Ar}), 5.58 (s, 1H, =C*H*H), 5.43 (d, 1H, J = 1.2 Hz, =CH*H*), 4.15 (s, 1H, CH), 3.82 (s, 3H, OCH_3), 3.67 (m, 1H, CH), 2.37 (m, 1H, C*H*H), 2.32 (d, 1H, J = 16.4 Hz, C*H*H), 2.02 (dd, 1H, J = 15.9, 4.5 Hz, CH*H*), 1.91 (d, 1H, J = 16.1 Hz, C*H*H), 1.60 (s, 3H, CH_3), 1.51 (s, 3H, CH_3), 1.06 (s, 3H, CH_3); ^{13}C NMR (125 MHz, $CHCl_3$, 25 ºC): δ = 153.3 (C=O), 151.1 ($C^{Ar\text{-}q}$), 143.3 (*C*=CH_2), 129.5 (CH^{Ar}), 127.0 (*C*=C), 126.8 (CH^{Ar}), 126.0 (C=*C*), 123.1 (CH^{Ar}), 123.0 ($C^{Ar\text{-}q}$), 120.8 (q, C-F_3, $^{1}J_{C\text{-}F}$ = 332.1 Hz), 116.0 (CH^{Ar}), 115.8 (=CH_2), 77.8 (C^{Cq}-Tf), 75.2 (C^{Cq}), 59.5 (CH), 52.0 (OCH_3), 47.1 (CH), 36.7 (CH_2), 31.3 (CH_2), 22.9 (CH_3), 18.8 (CH_3), 17.8 (CH_3); ^{19}F NMR (282 MHz, $CHCl_3$, 25 ºC): δ = –68.9 (s, 3F, CF_3); IR ($CHCl_3$): ν = 1697 (C=O), 1389, 1195 (O=S=O), 1199 (C–F) cm^{-1}. Badly ionizing compound in MS.

Procedure for the preparation of tricyclic dienyl triflone 18.

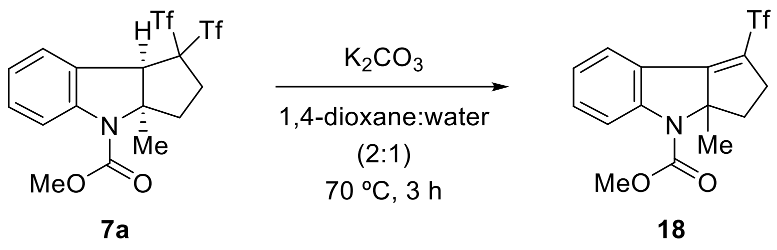

A stirred mixture of bis(triflyl)-decorated tricyclic indoline **7a** (1.0 mmol) and K_2CO_3 (5.0 mmol) in 1,4-dioxane/water (2:1, 10 mL) was heated at 70 ºC until disappearance of the starting material (TLC). The reaction was cooled to room temperature, water was added and the mixture was extracted with AcOEt (3 x 10 mL). The organic phase was washed with water (2 × 5 mL), dried ($MgSO_4$) and concentrated under reduced pressure. The resulting residue was purified by column chromatography to give product **18**.

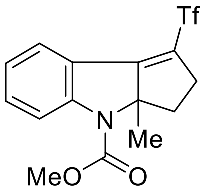

18

Tricyclic dienyl triflone 18. From 50 mg (0.10 mmol) of bis(triflyl)-decorated tricyclic indoline **7a**, and after flash chromatography of the residue using hexanes/ethyl acetate (9:1) as eluent gave compound **18** (31 mg, 86%) as a pale yellow oil; ^{1}H NMR (500 MHz, $CHCl_3$, 25 ºC): δ = 8.16 (d, 1H, J = 7.8 Hz, CH^{Ar}), 8.13 (br s, 1H, CH^{Ar}), 7.51 (t, 1H, J = 7.8 Hz, CH^{Ar}), 7.15 (t, 1H, J = 7.7 Hz, CH^{Ar}), 3.89 (s, 3H, OCH_3), 3.18 (m, 1H, C*H*H), 3.08 (dd, 1H, J = 16.2, 8.3 Hz, CH*H*), 2.54 (br s, 1H, C*H*H), 2.39 (dd, 1H, J = 19.9, 11.2 Hz, CH*H*), 1.50 (s, 3H, CH_3); ^{13}C NMR (125 MHz, $CHCl_3$, 25 ºC): δ = 169.9 (*C*=CTf), 152.1 (C=O), 150.2 (C=*C*Tf), 134.8 (CH^{Ar}), 128.4 (CH^{Ar}), 123.9 (CH^{Ar}), 120.2 (q, C-F_3, $^{1}J_{C\text{-}F}$ = 326.9 Hz), 118.7 ($C^{Ar\text{-}q}$), 117.2 ($C^{Ar\text{-}q}$), 116.4 (CH^{Ar}), 79.6 (C^{Cq}), 52.9 (OCH_3),

39.3 (CH_2), 36.1 (CH_2), 20.9 (CH_3); ^{19}F NMR (282 MHz, $CHCl_3$, 25 °C): δ = –78.6 (s, 3F, CF_3); IR ($CHCl_3$): ν = 1697 (C=O), 1345, 1216 (O=S=O), 1208 (C–F) cm^{-1}. Badly ionizing compound in MS.

Procedure for the preparation of bis(triflyl)ethyl-decorated bicyclic indoline 19.

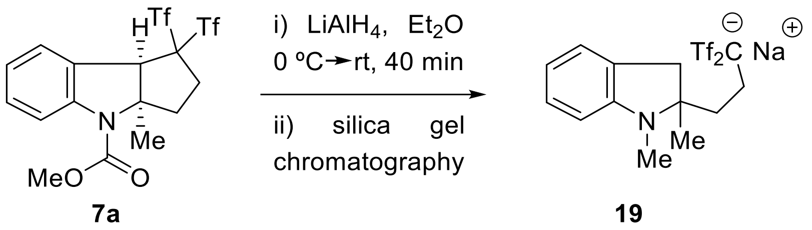

A round bottom flask equipped with a magnetic stir bar was charged with bis(triflyl)-decorated tricyclic indoline **7a** (1.0 mmol) and anhydrous diethyl ether (20 mL). $LiAlH_4$ (5.0 mmol) was added portionwise at 0 °C. The reaction mixture was warmed up to room temperature, and stirred until complete consumption of starting material as monitored by TLC. After then the reaction was quenched; water was added at 0 ºC and the mixture was extracted with AcOEt (3 x 20 mL). The combined organic layer was washed with brine and dried over $MgSO_4$. The solvent was removed under reduced pressure to afford the crude product, which was purified by column chromatography to give product **19**.

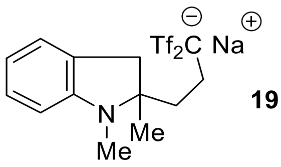

19

Bis(triflyl)ethyl-decorated bicyclic indoline 19. From 50 mg (0.10 mmol) of bis(triflyl)-decorated tricyclic indoline **7a**, and after flash chromatography of the residue using hexanes/ethyl acetate (1:1) as eluent gave compound **19** (38 mg, 80%) as a colorless oil; ^{1}H NMR (500 MHz, acetone-d_6, 25 ºC): δ = 6.88 (m, 2H, 2CH^{Ar}), 6.41 (t, 1H, J = 7.3 Hz, CH^{Ar}), 6.20 (d, 1H, J = 8.0 Hz, CH^{Ar}), 2.95 (d, 1H, J = 15.6 Hz, C*H*H), 2.59 (s, 3H, NCH_3), 2.56 (d, 1H, J = 15.6 Hz, CH*H*), 2.26 (m, 2H, CH_2), 1.91 (m, 1H, C*H*H), 1.80 (m, 1H, CH*H*), 1.03 (s, 3H, CH_3); ^{13}C NMR (125 MHz, acetone-d_6, 25 ºC): δ =

153.1 (C^{Ar-q}), 128.4 (C^{Ar-q}), 128.0 (CH^{Ar}), 124.5 (CH^{Ar}), 122.5 (q, 2C-F_3, $^1J_{C\text{-}F}$ = 328.6 Hz), 116.8 (CH^{Ar}), 105.7 (CH^{Ar}), 67.3 (C^{Cq}), 64.4 (CTf_2), 41.5 (CH_2), 40.6 (CH_2), 27.9 (NCH_3), 24.5 (CH_2), 23.1 (CH_3); ^{19}F NMR (282 MHz, acetone-d_6, 25 ºC): $\delta = -79.9$ (s, 6F, 2CF_3); IR (acetone): ν = 1393, 1205 (O=S=O), 1207 (C–F) cm^{-1}; HRMS (ESI) m/z: $[M]^-$ Calcd for $C_{15}H_{16}F_6NO_4S_2$ 452.0430; Found 452.0409.

^{1}H NMR (300 MHz, $CDCl_3$)

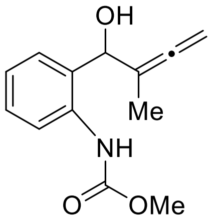

2a

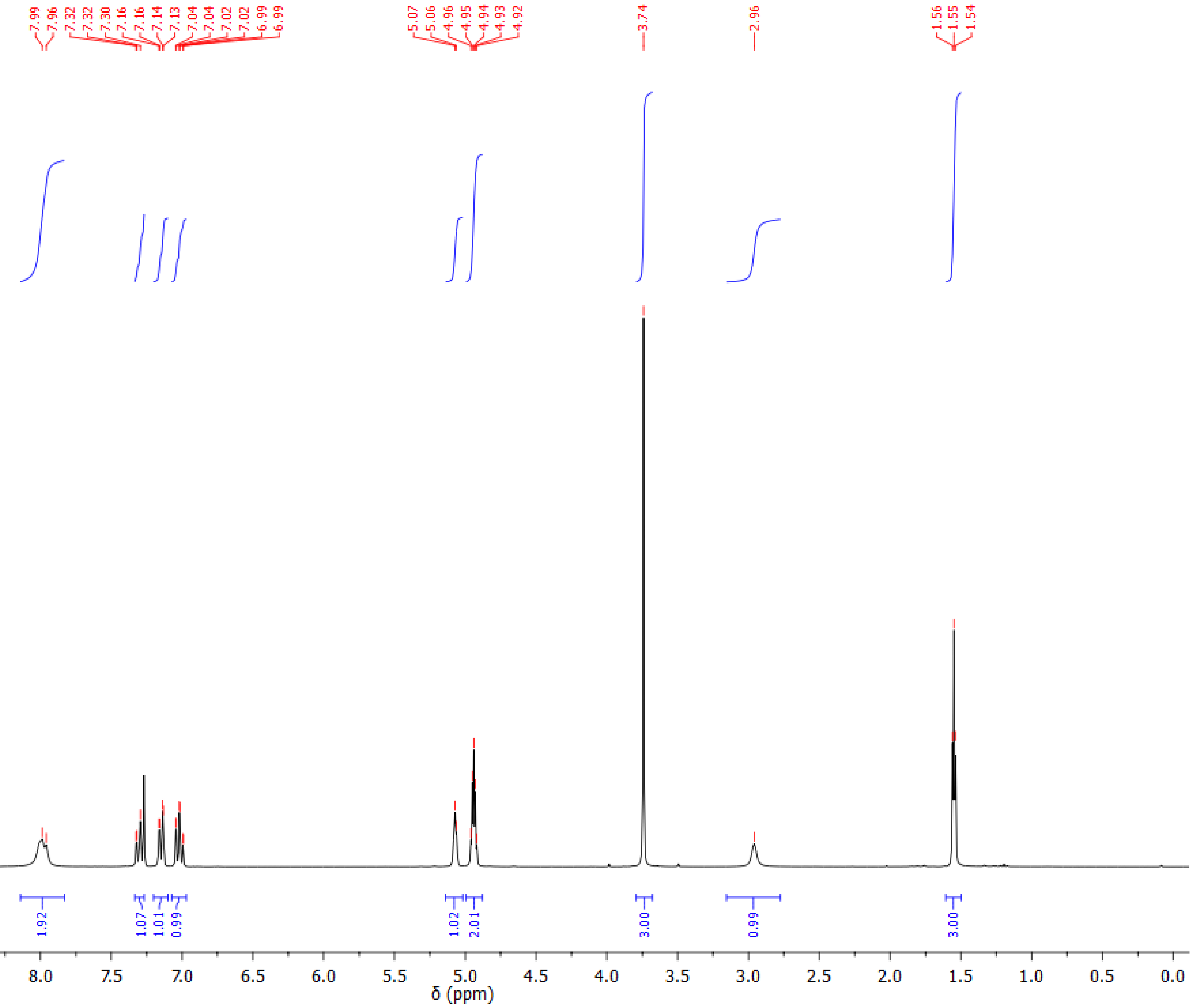

^{13}C NMR (75 MHz, $CDCl_3$)

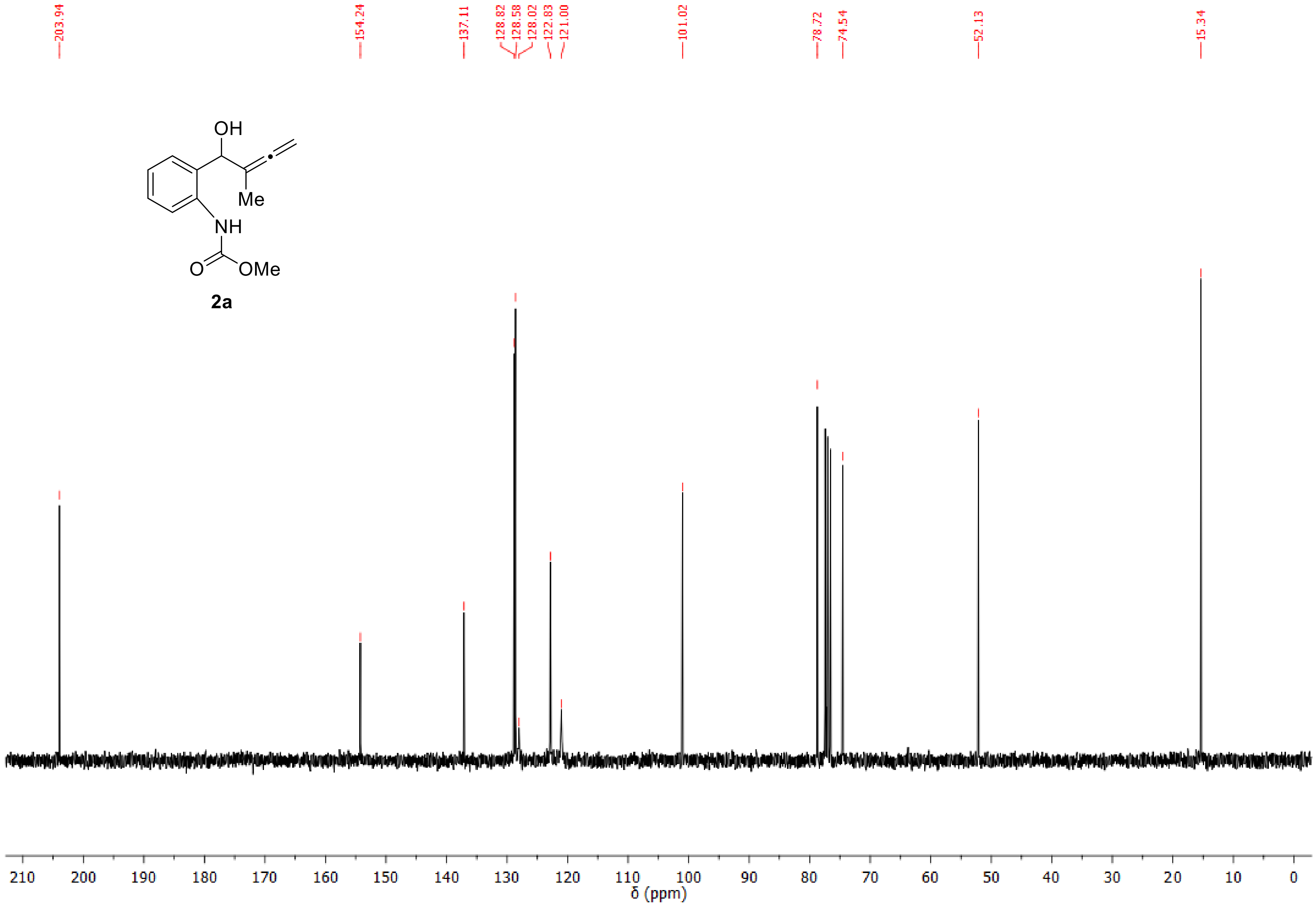

^{1}H NMR (300 MHz, $CDCl_3$)

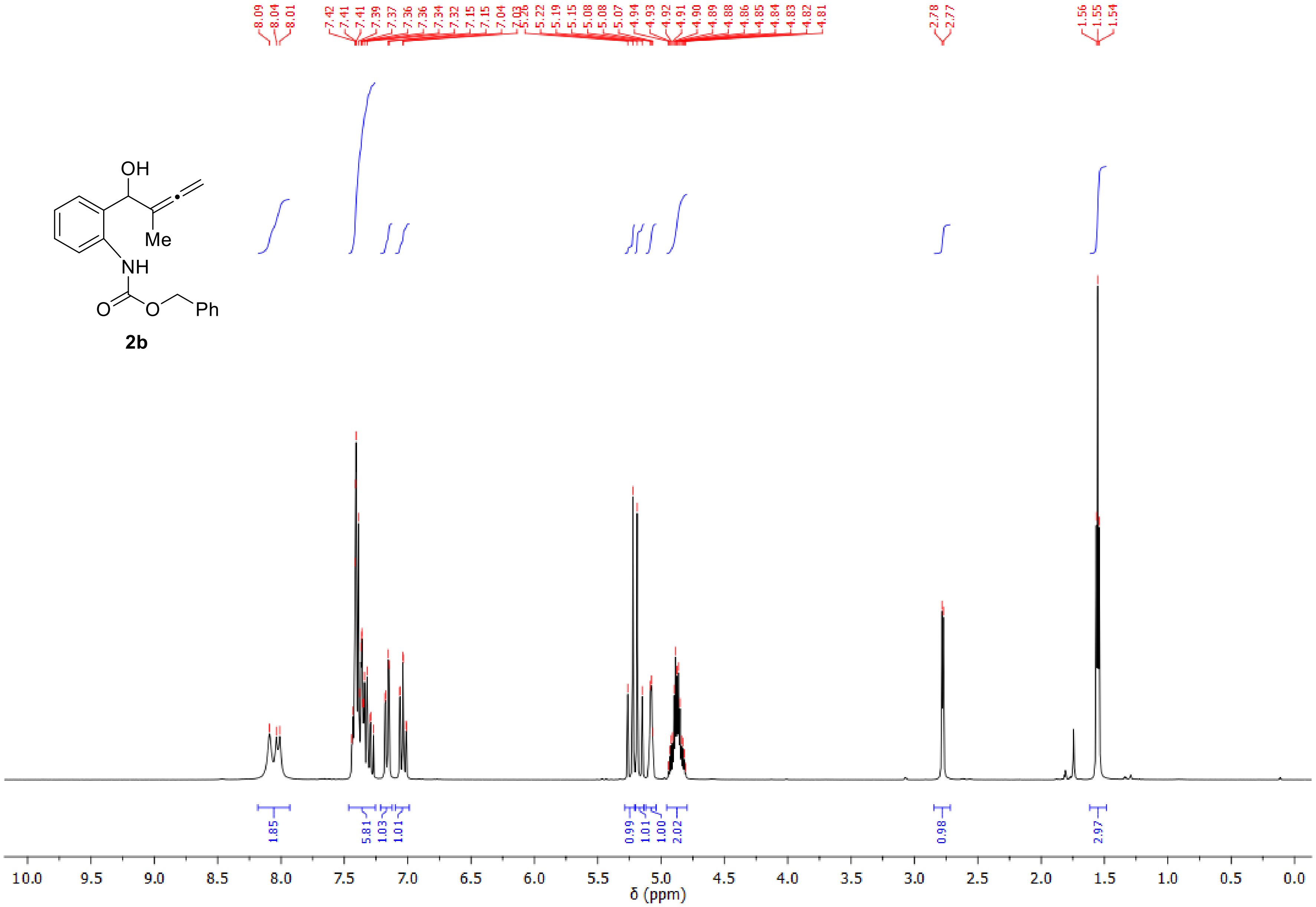

2b

^{13}C NMR (75 MHz, $CDCl_3$)

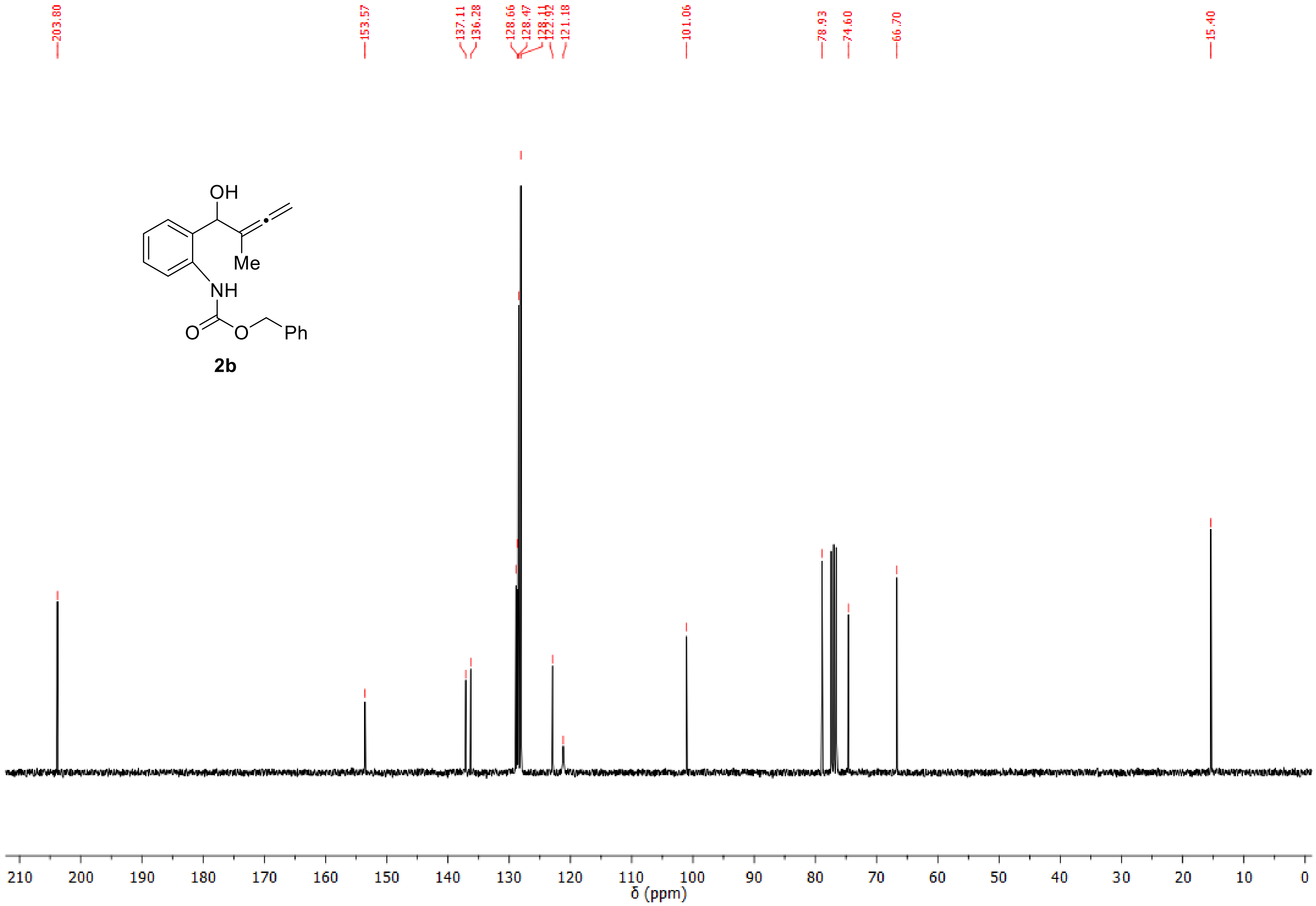

^{1}H NMR (300 MHz, $CDCl_3$)

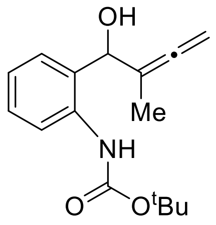

2c

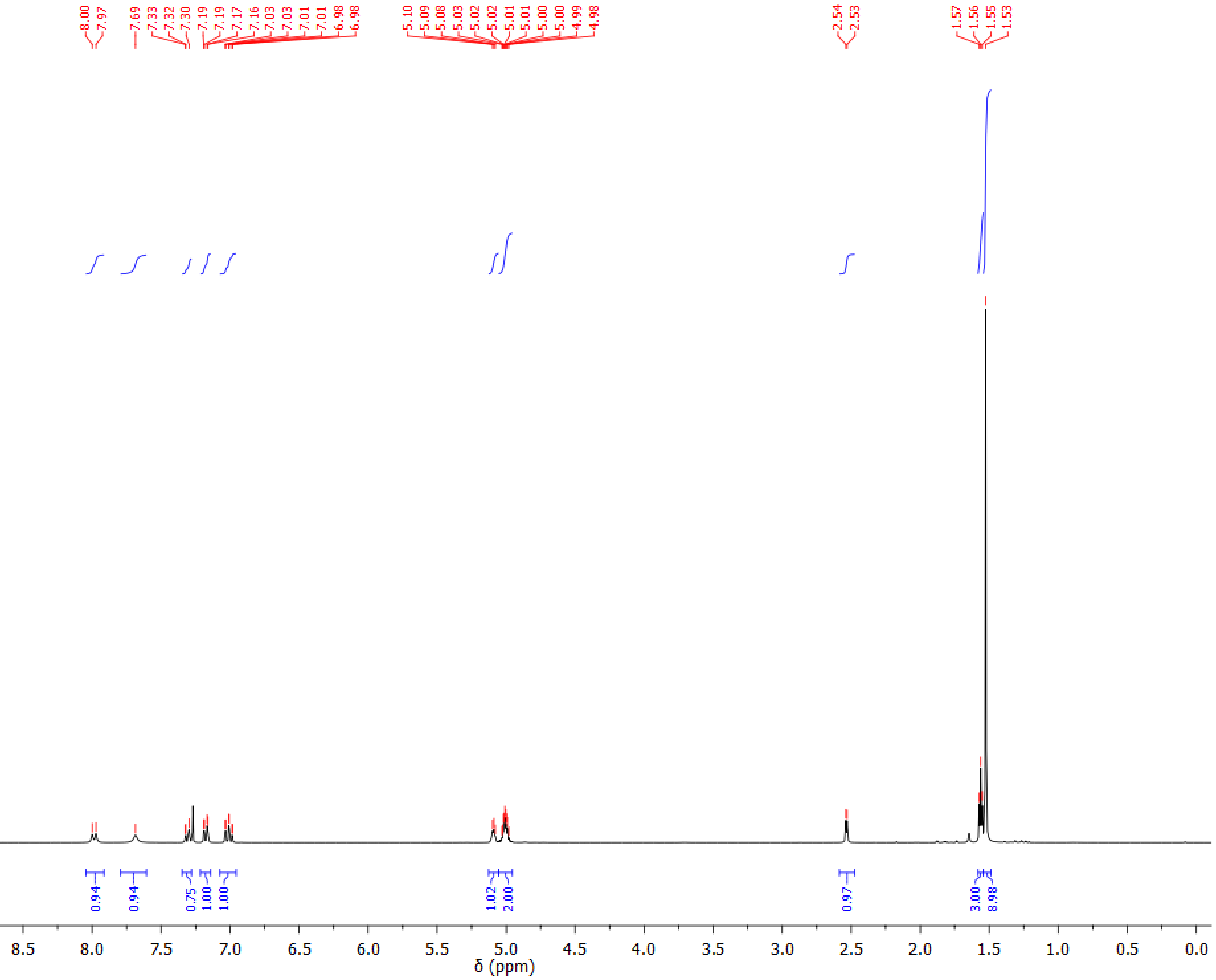

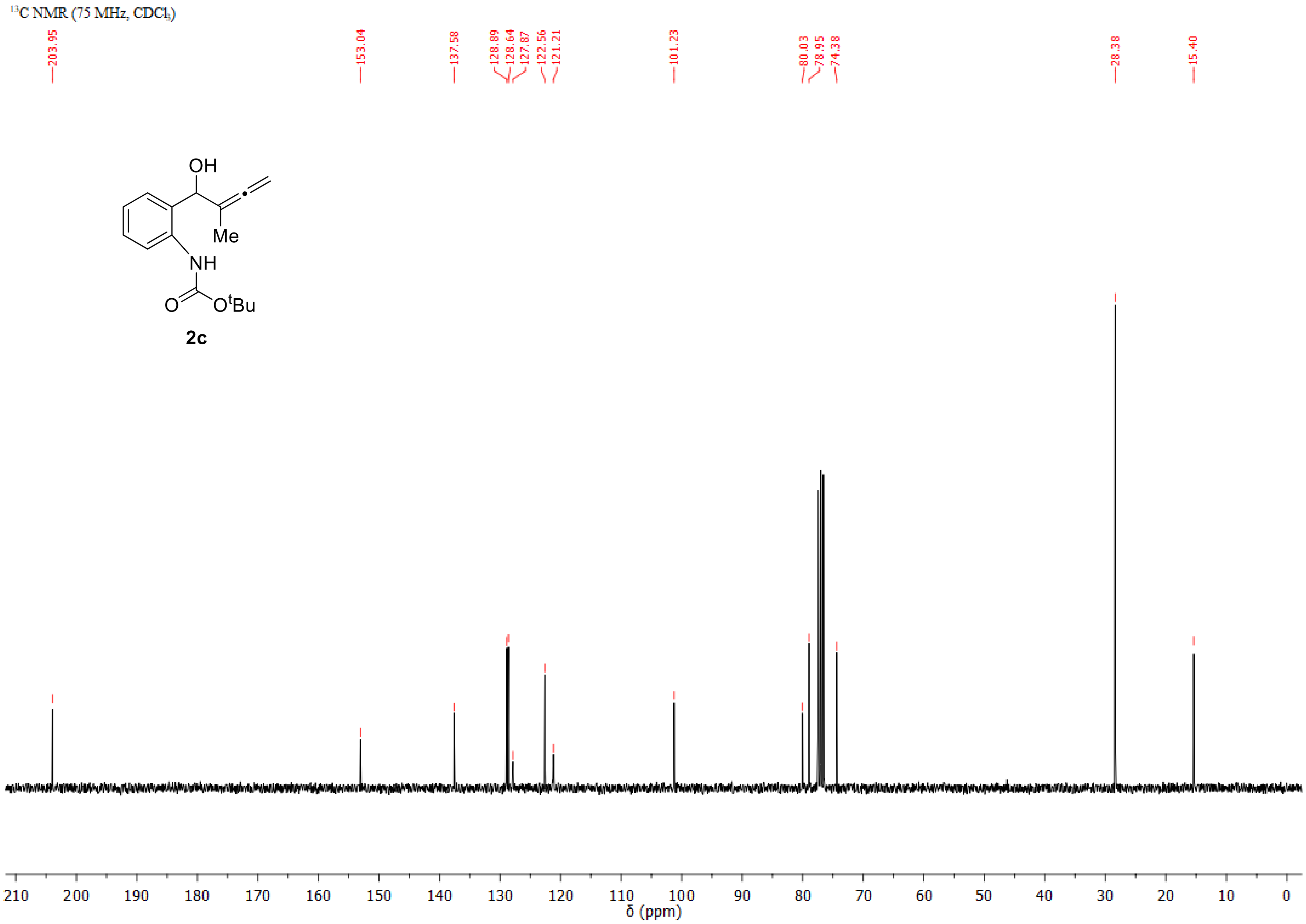
13C NMR (75 MHz, CDCl3)
203.95
153.04
137.58
128.89
128.64
127.87
122.56
121.21
101.23
80.03
78.95
74.38
28.38
15.40
OH
Me
NH
O
OtBu
2c
210
200
190
180
170
160
150
140
130
120
110
100
90
80
70
60
50
40
30
20
10
0
δ (ppm)

^{1}H NMR (300 MHz, $CDCl_3$)

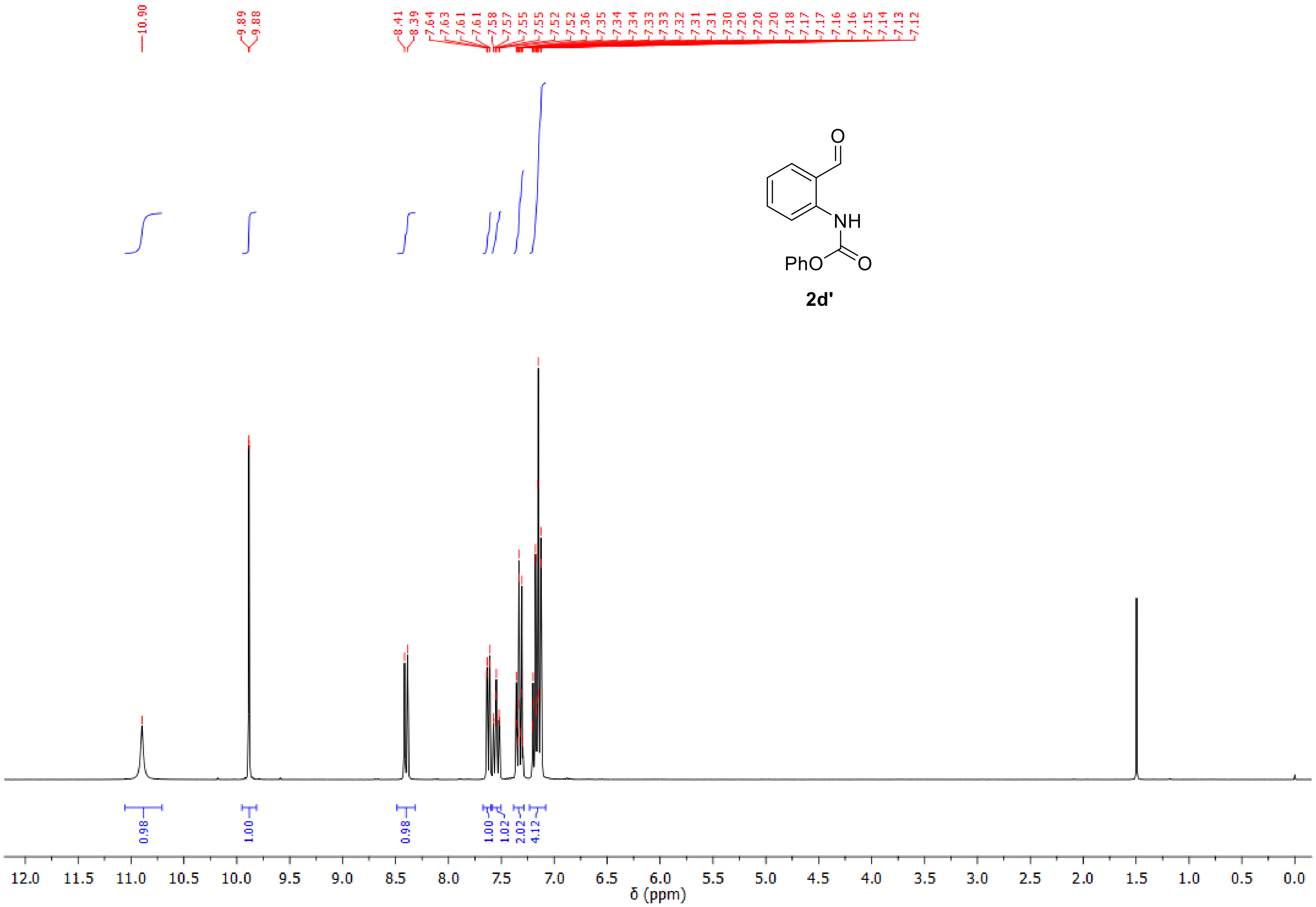

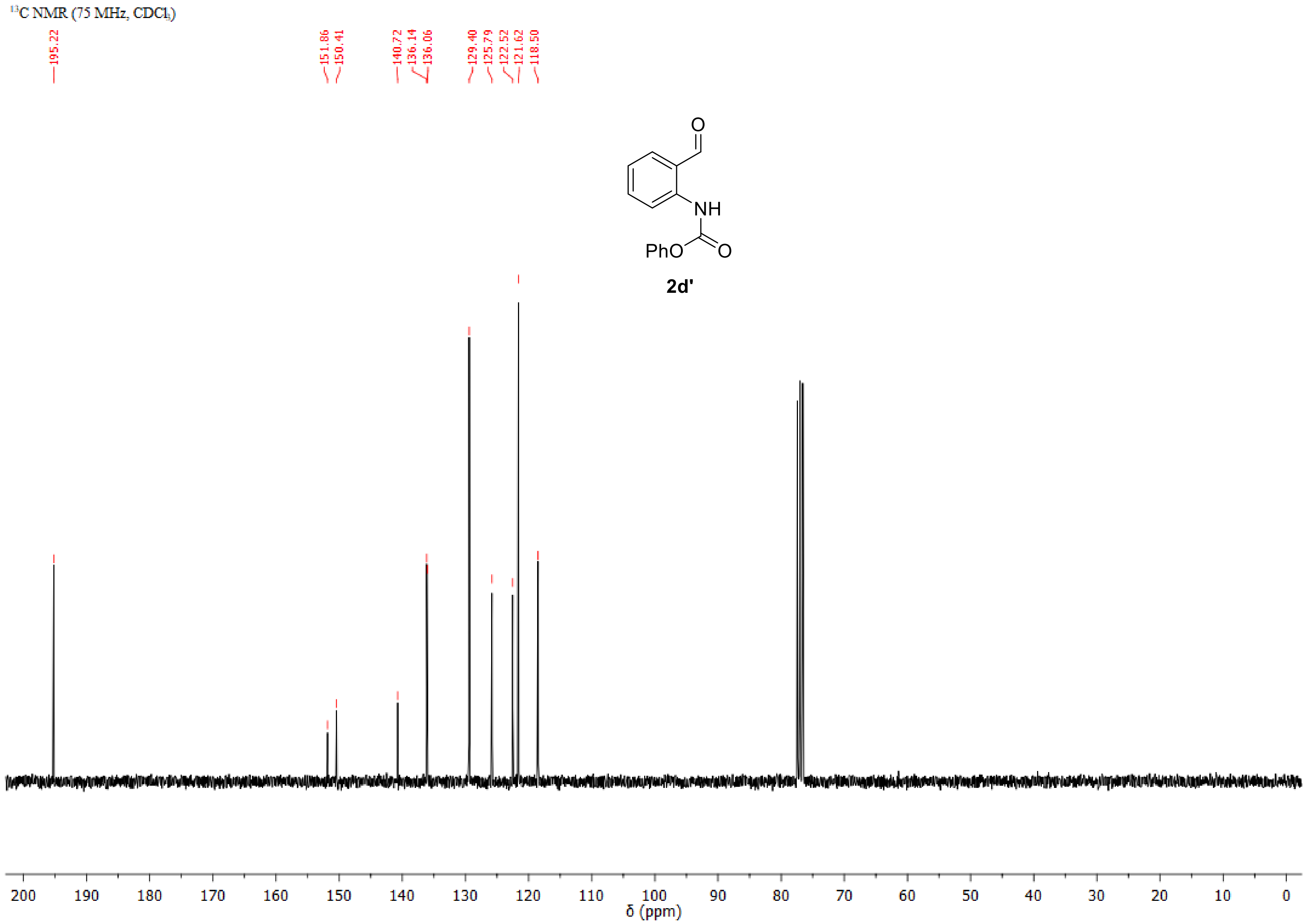
13C NMR (75 MHz, CDCl3)
195.22
151.86
150.41
140.72
136.14
136.06
129.40
125.79
122.52
121.62
118.50
O
NH
PhO
O
2d'
200 190 180 170 160 150 140 130 120 110 100 90 80 70 60 50 40 30 20 10 0
δ (ppm)

^{1}H NMR (300 MHz, CDCl$_3$)

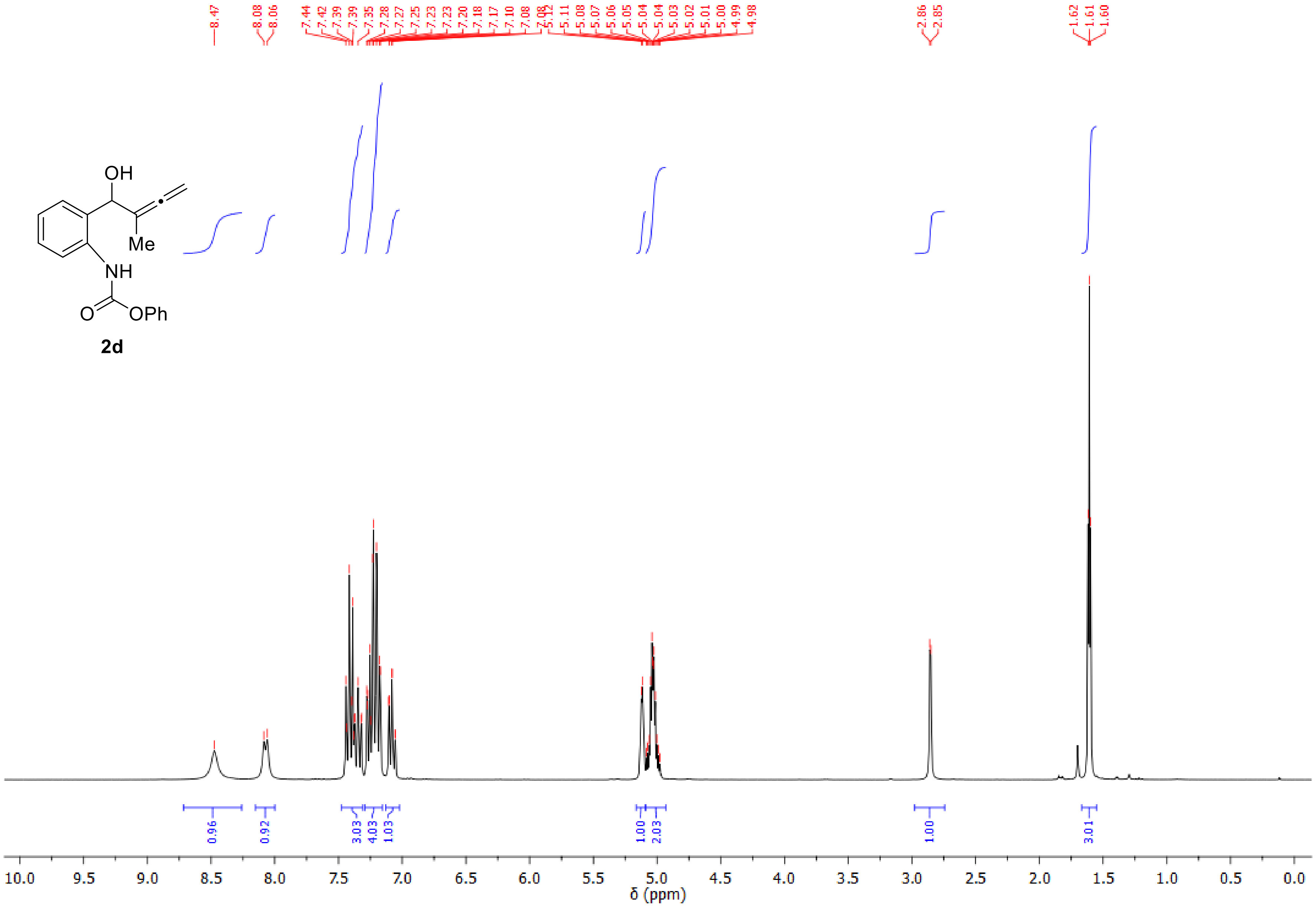

^{13}C NMR (75 MHz, $CDCl_3$)

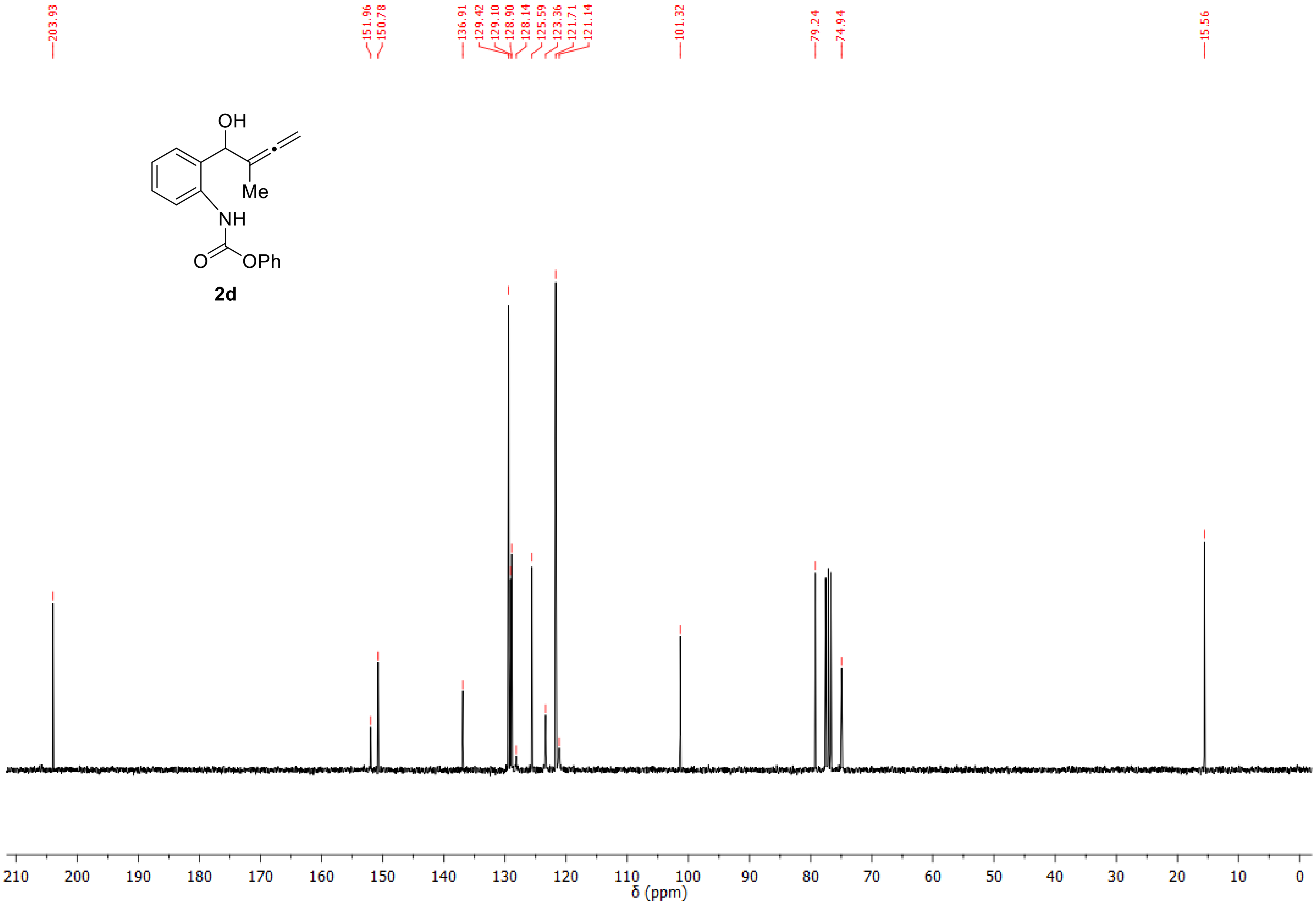

^{1}H NMR (300 MHz, $CDCl_3$)

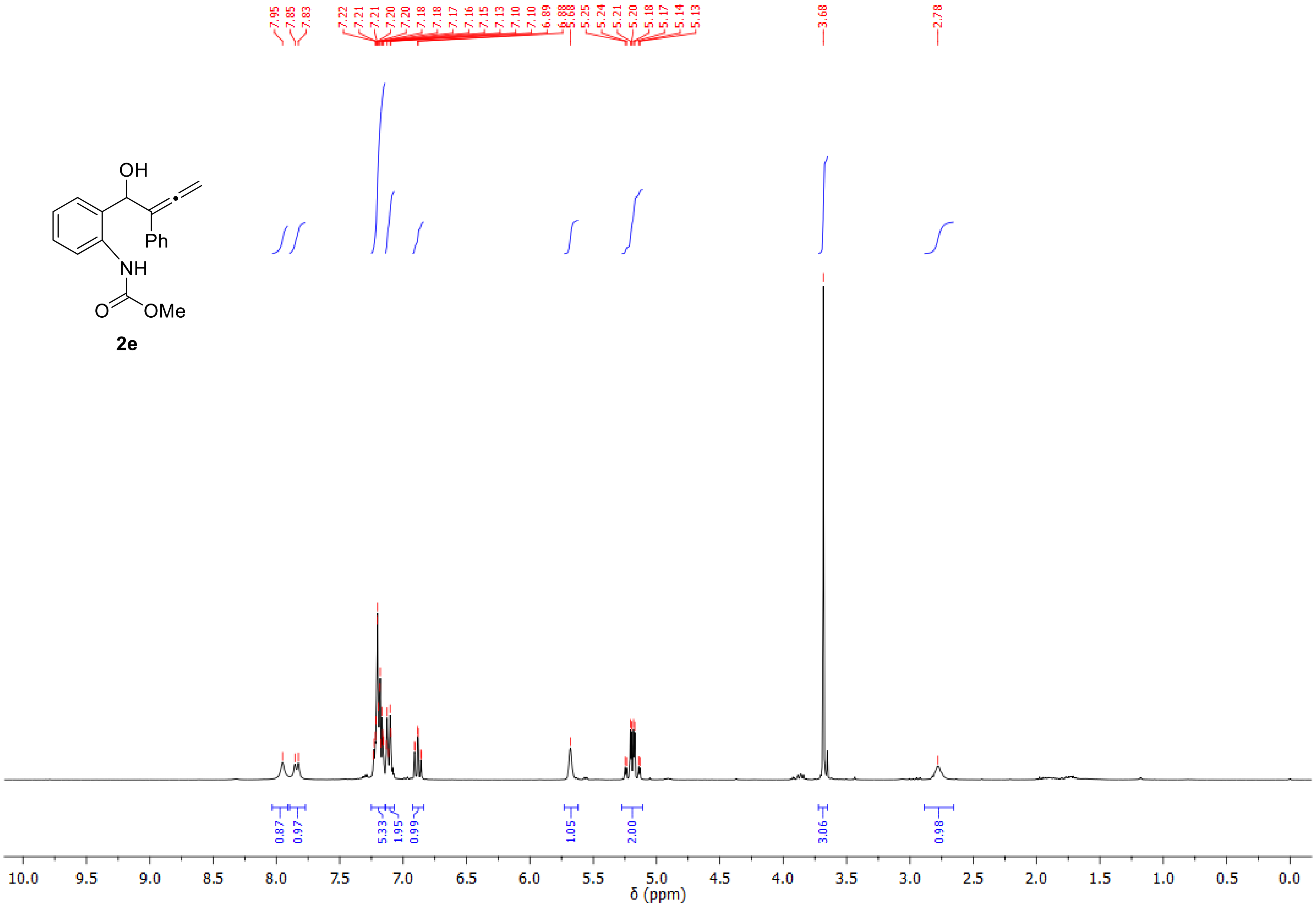

^{13}C NMR (75 MHz, $CDCl_3$)

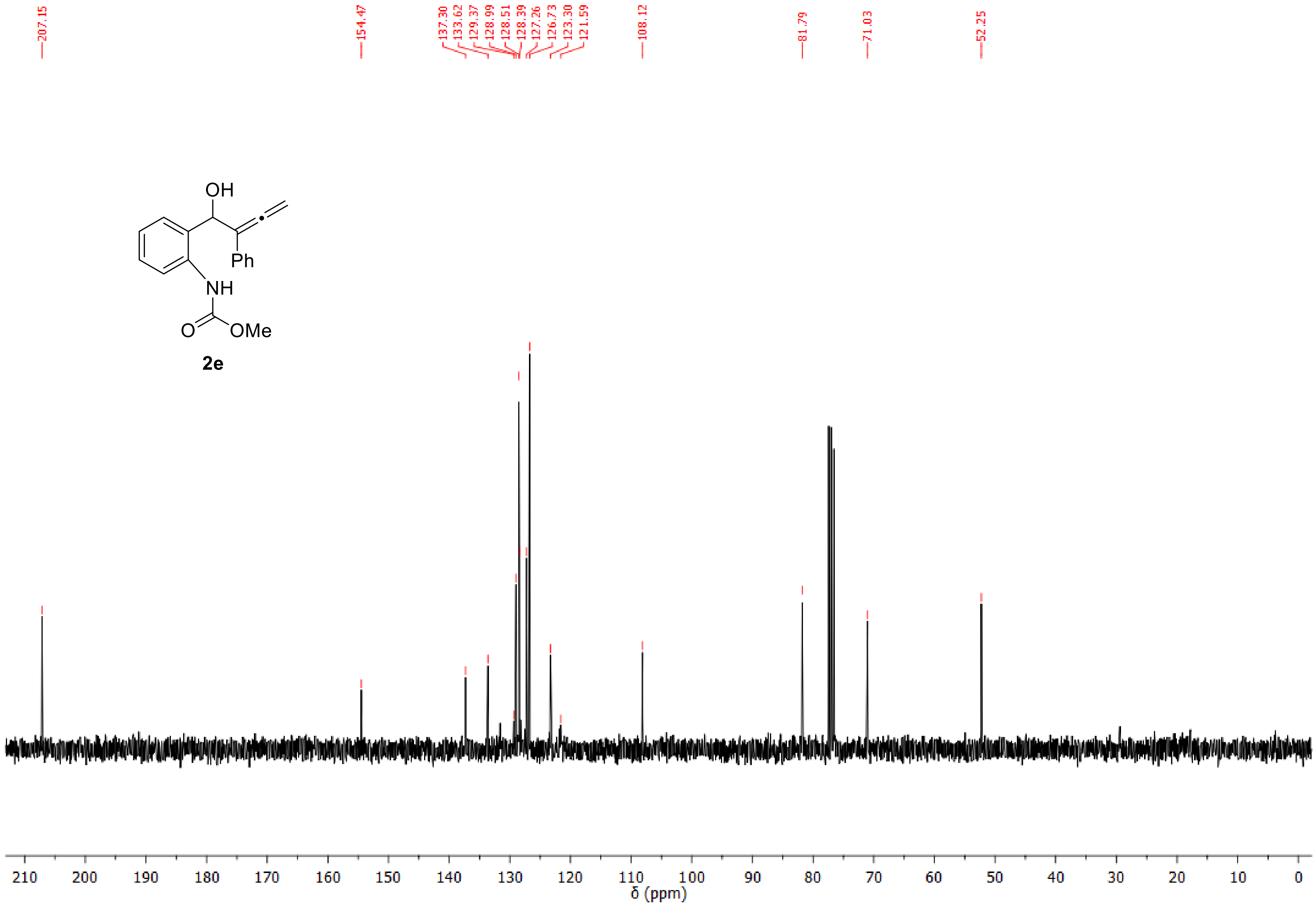

2e

^{1}H NMR (300 MHz, $CDCl_3$)

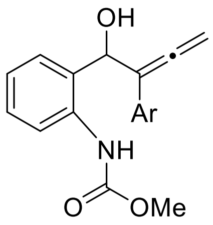

Ar = 4-$MeOC_6H_4$

2f

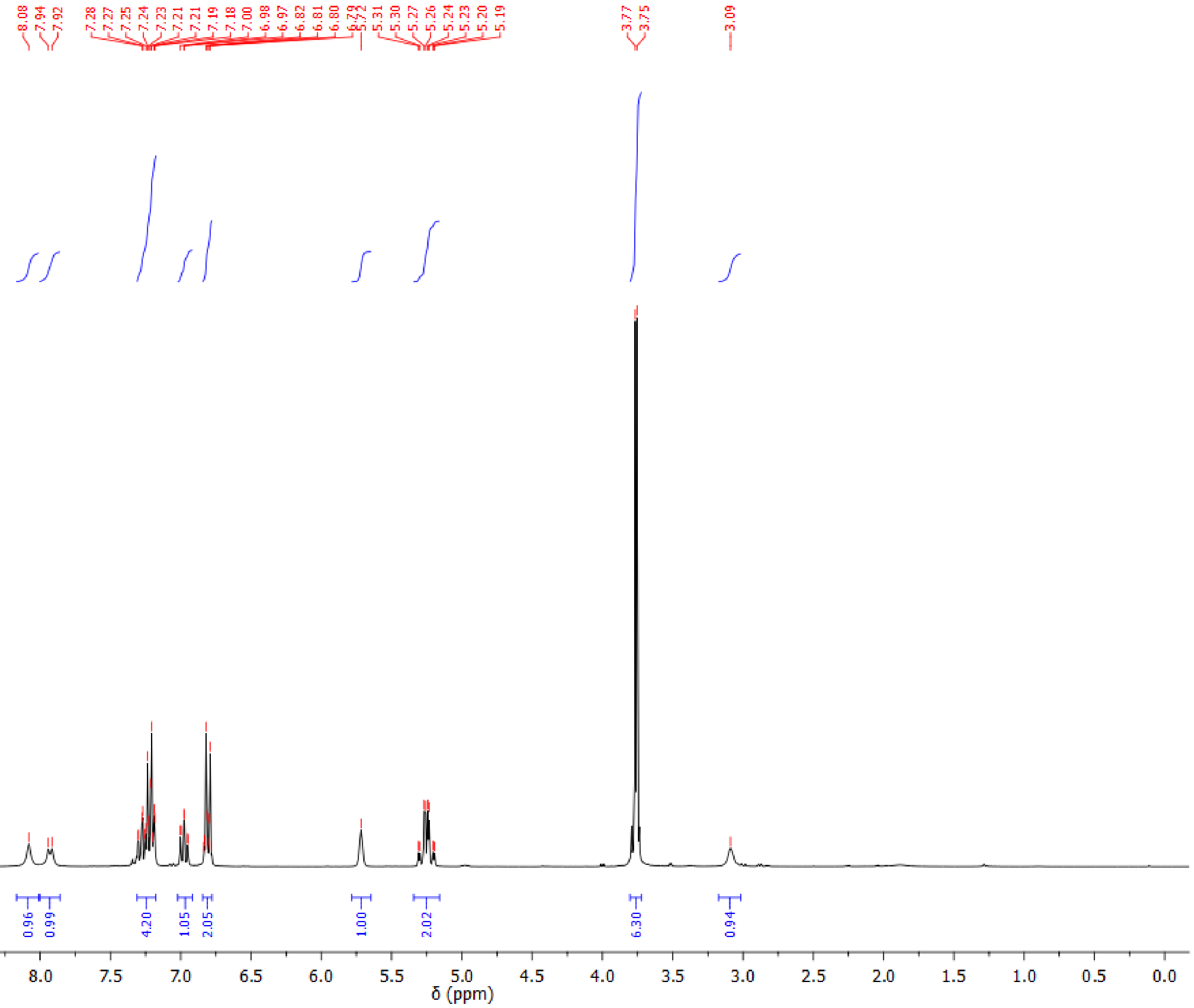

^{13}C NMR (75 MHz, $CDCl_3$)

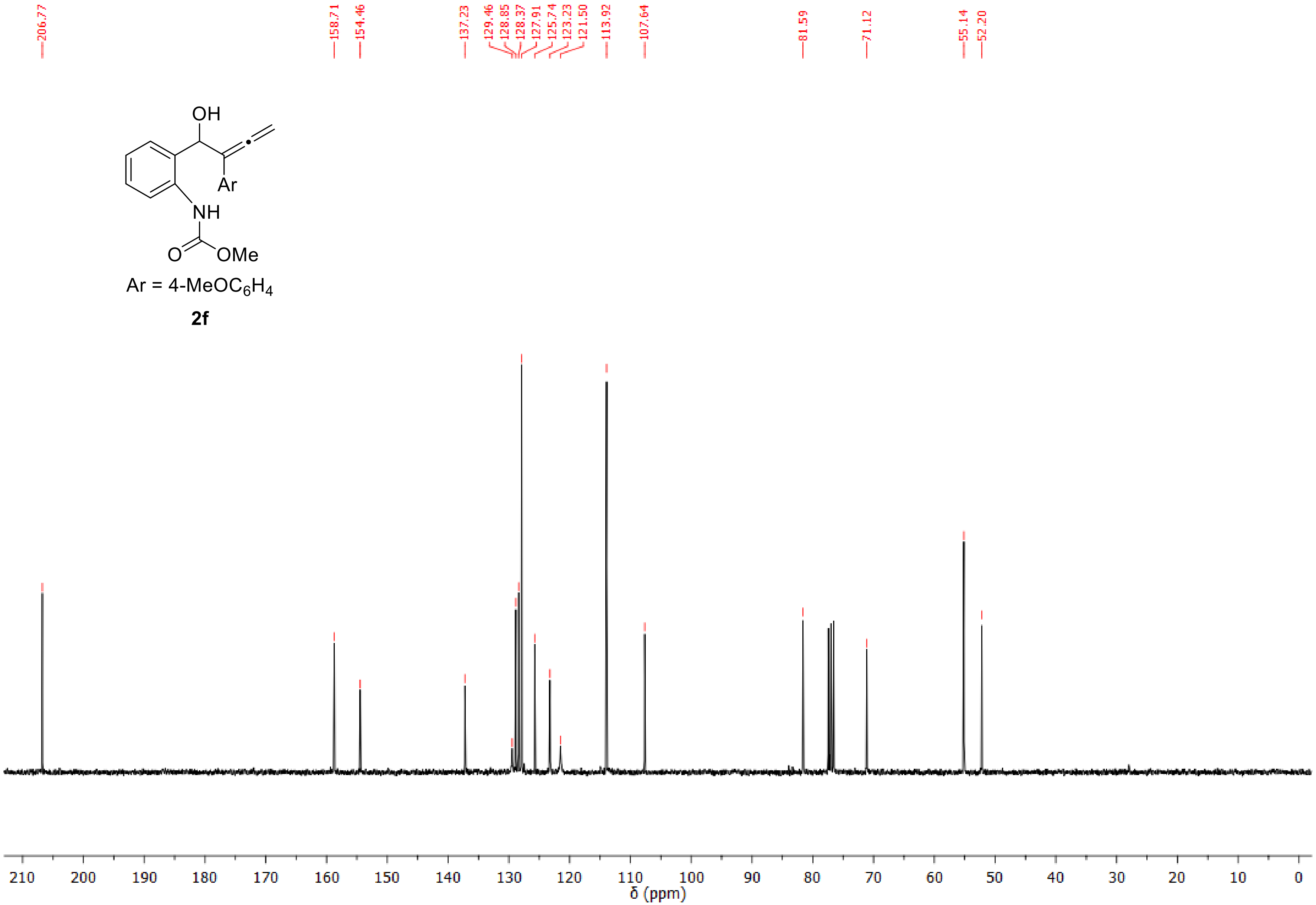

^{1}H NMR (300 MHz, $CDCl_3$)

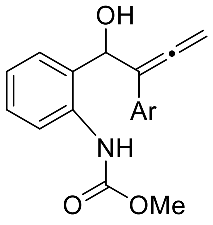

Ar = 4-BrC_6H_4

2g

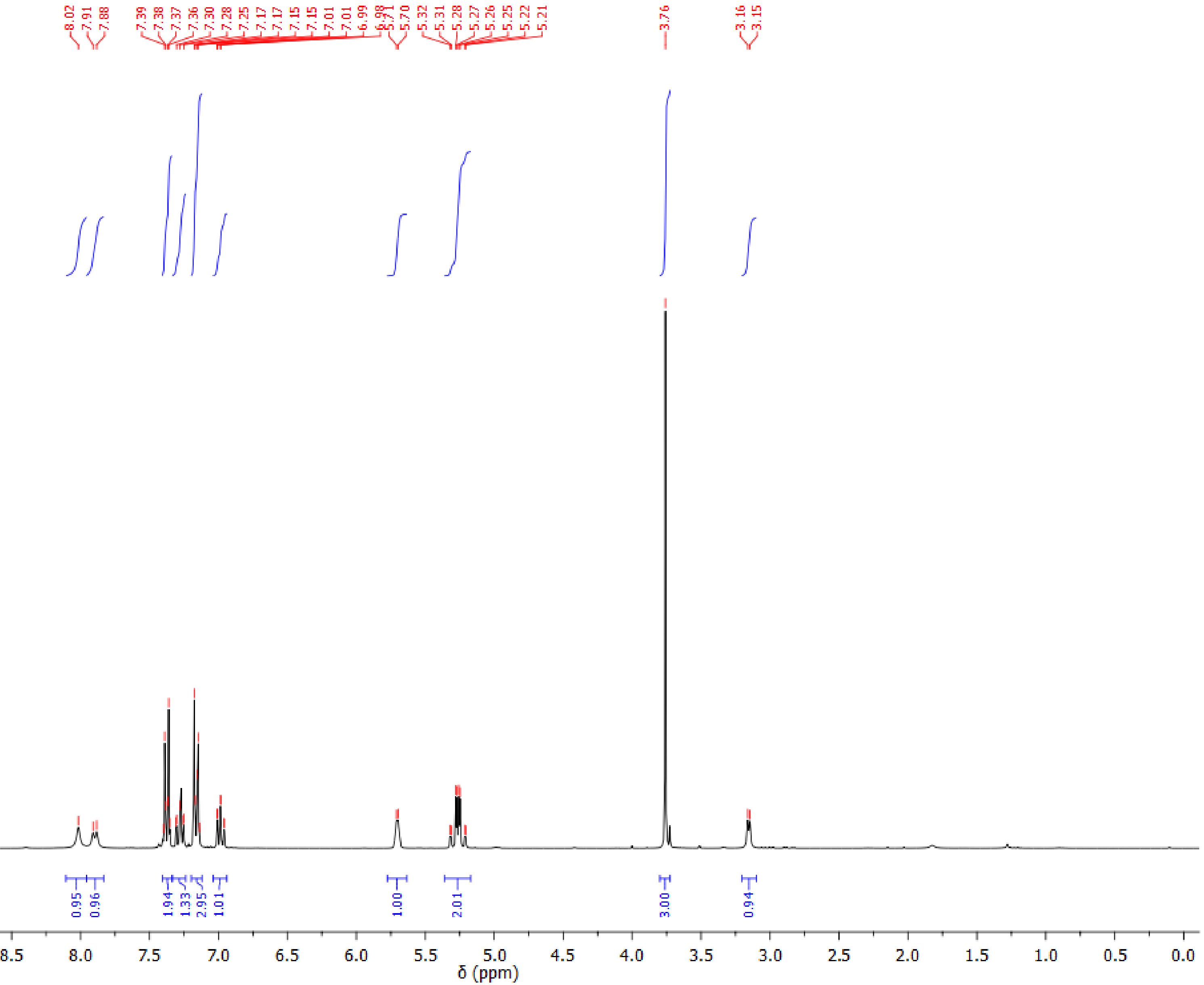

^{13}C NMR (75 MHz, $CDCl_3$)

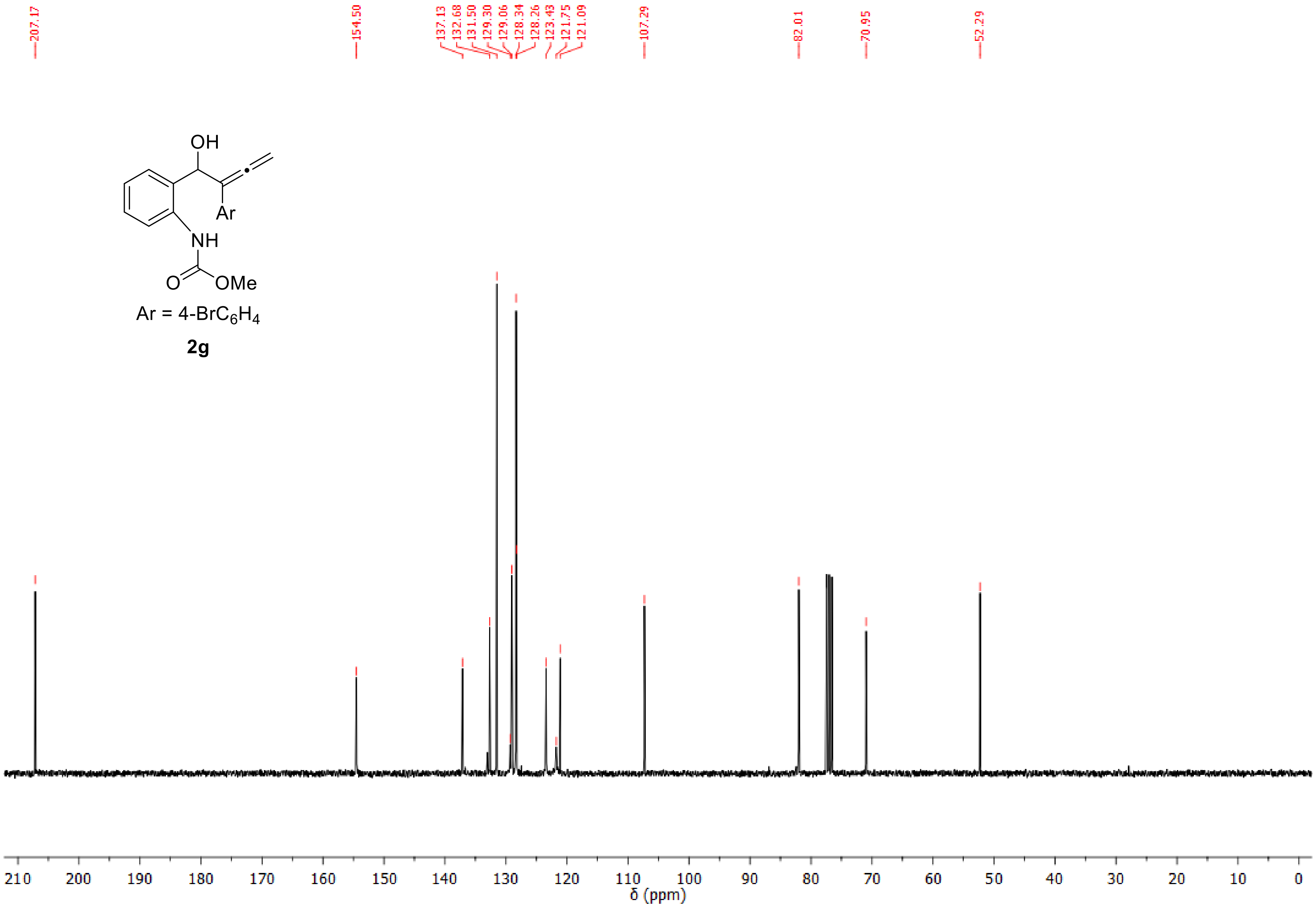

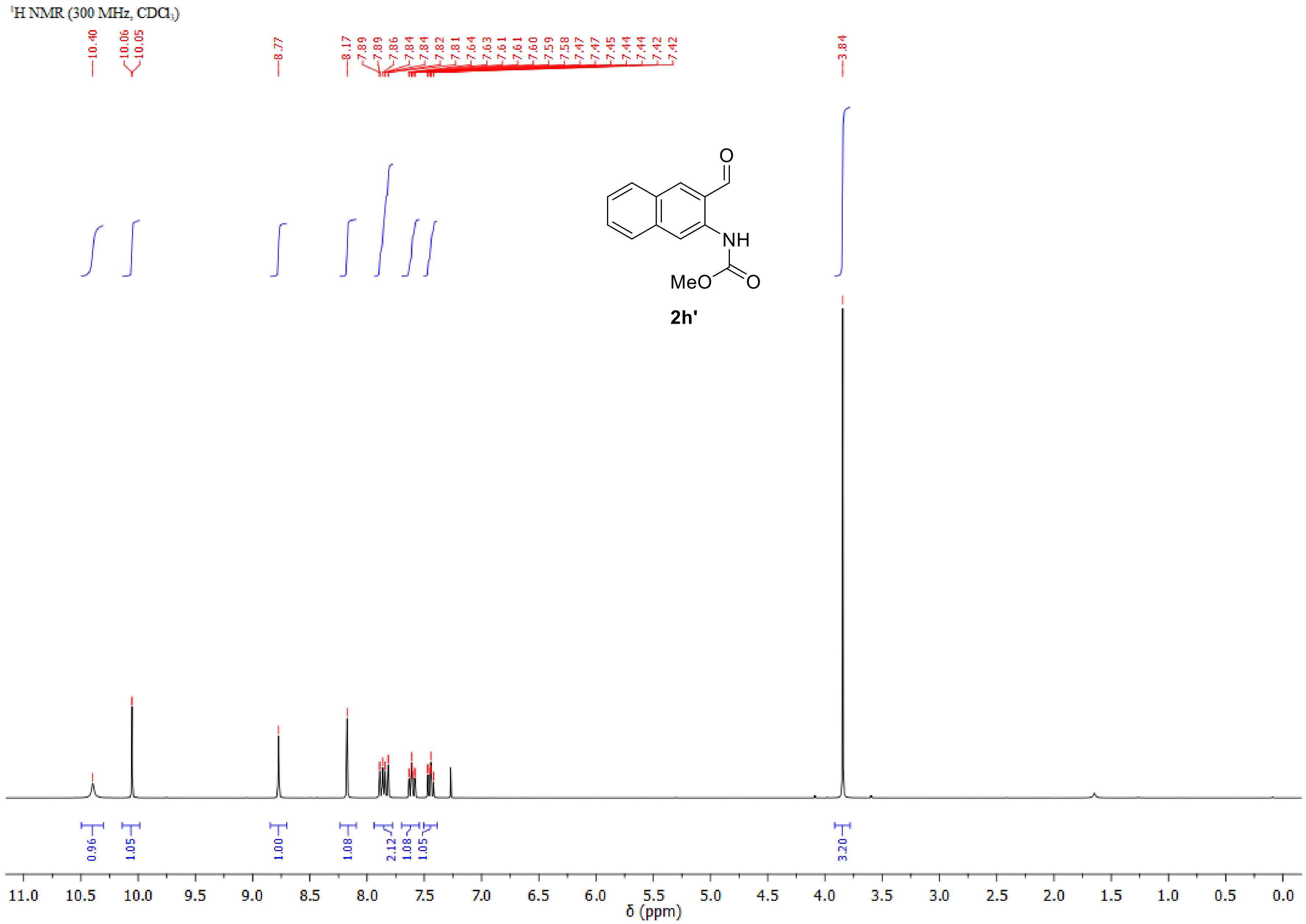

^{1}H NMR (300 MHz, CDCl3)
10.40
10.06
10.05
8.77
8.17
7.89
7.89
7.86
7.84
7.84
7.82
7.81
7.64
7.63
7.61
7.61
7.60
7.59
7.58
7.47
7.47
7.45
7.44
7.44
7.42
7.42
3.84
O
NH
MeO
O
2h'
0.96
1.05
1.00
1.08
2.12
1.08
1.05
3.20
11.0 10.5 10.0 9.5 9.0 8.5 8.0 7.5 7.0 6.5 6.0 5.5 5.0 4.5 4.0 3.5 3.0 2.5 2.0 1.5 1.0 0.5 0.0
δ (ppm)

^{13}C NMR (75 MHz, $CDCl_3$)

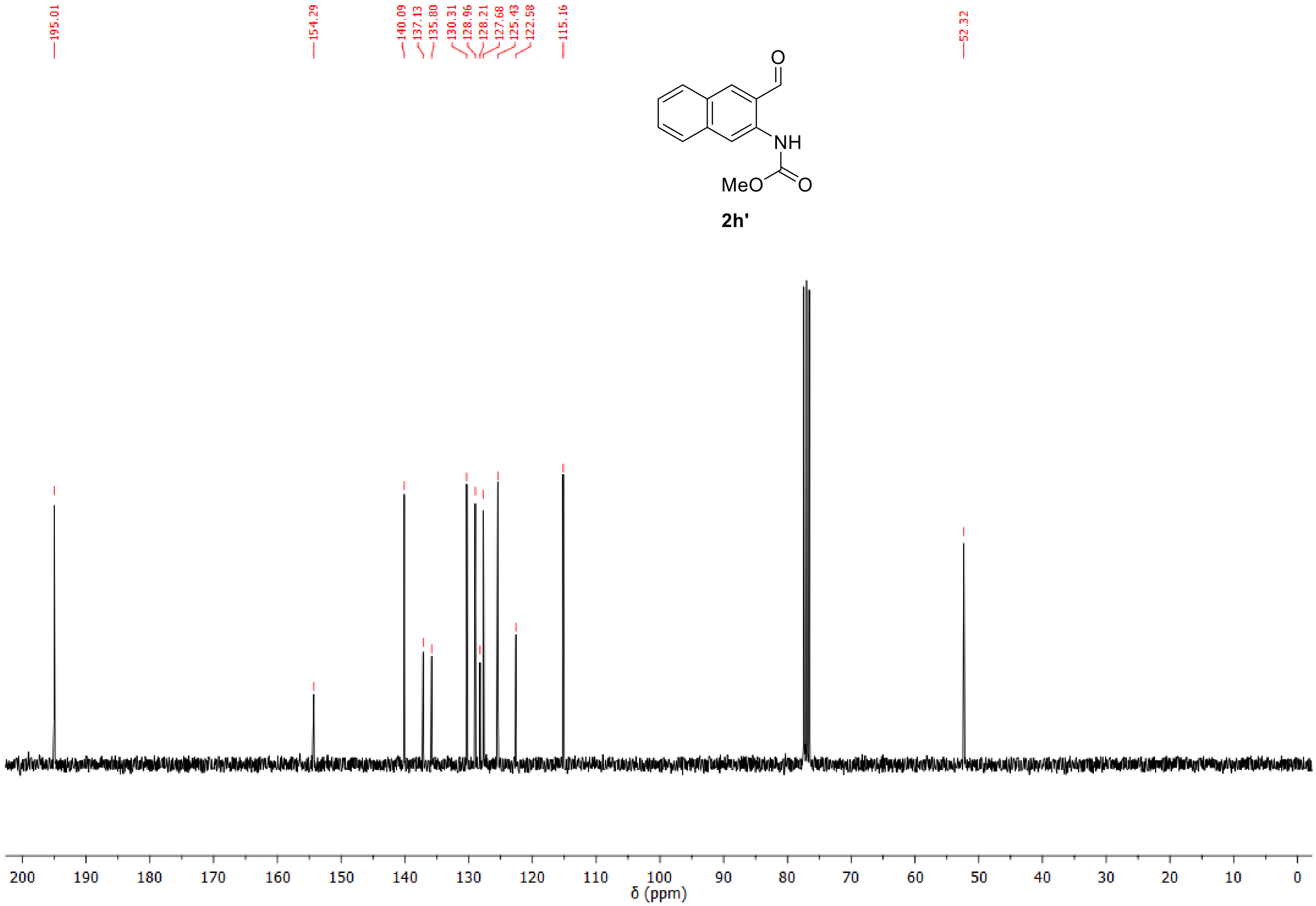

^{1}H NMR (300 MHz, $CDCl_3$)

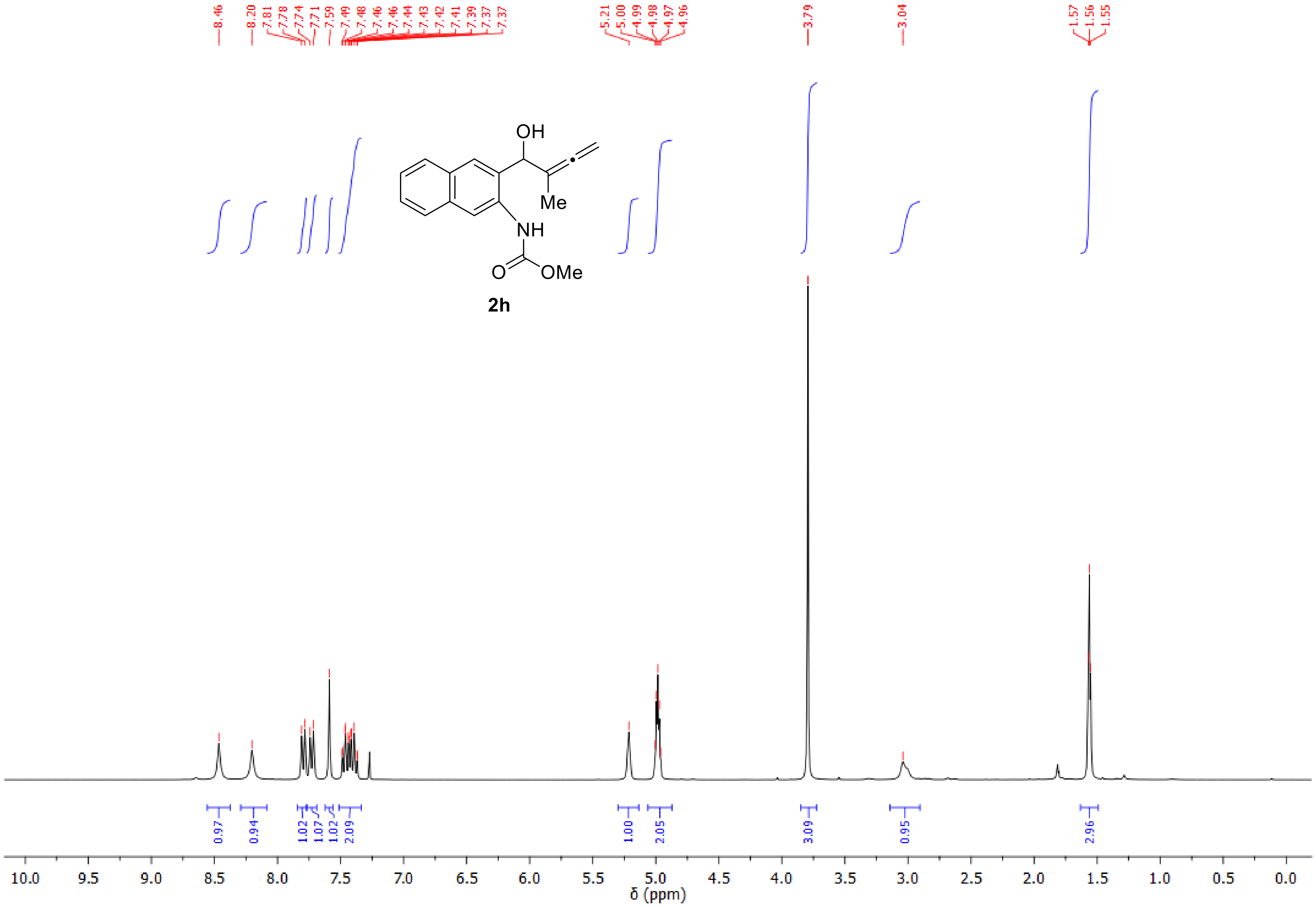

^{13}C NMR (75 MHz, $CDCl_3$)

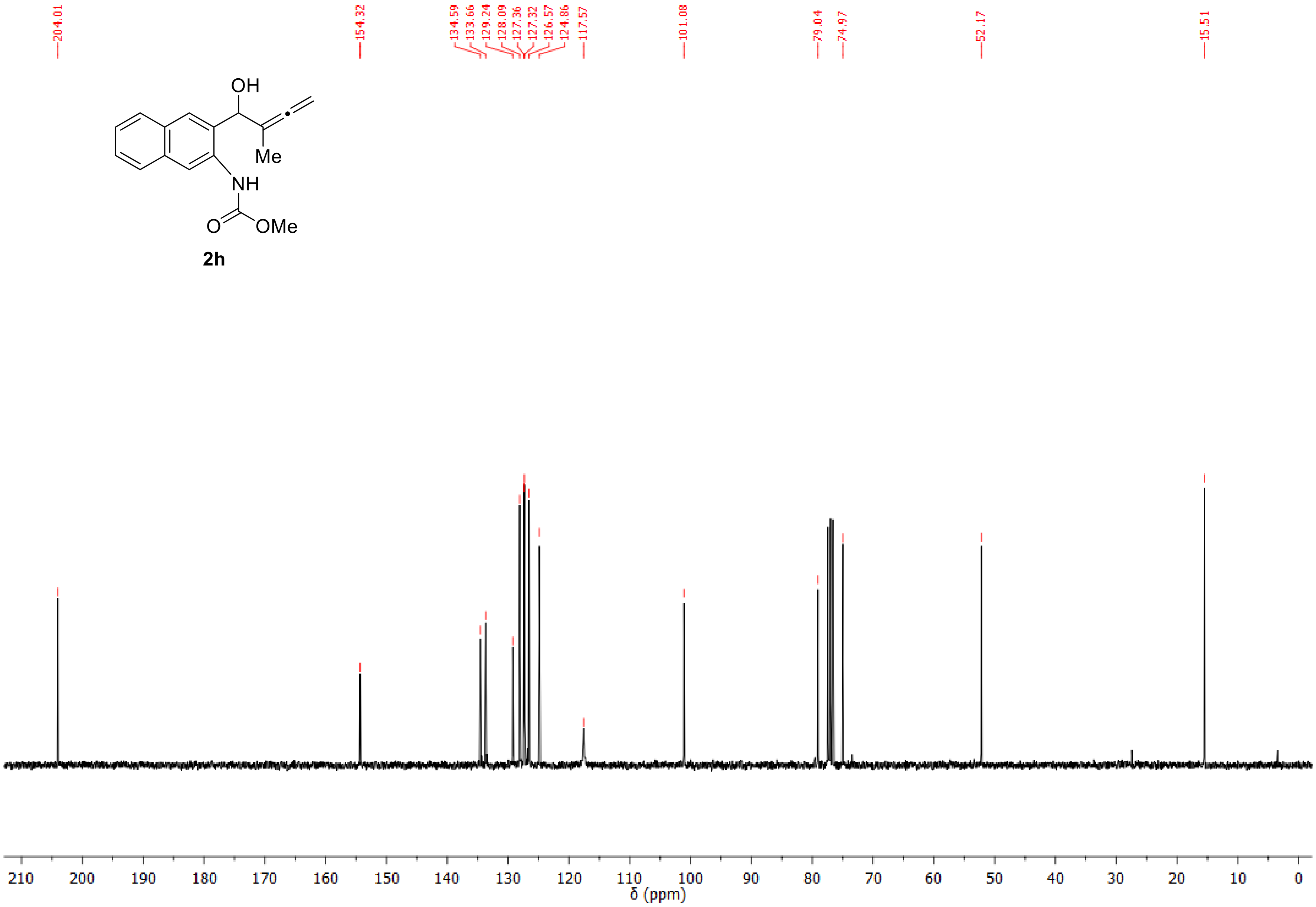

^{1}H NMR (300 MHz, $CDCl_3$)

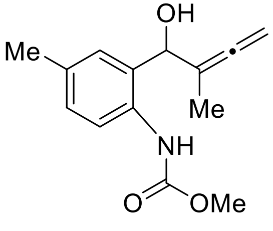

2i

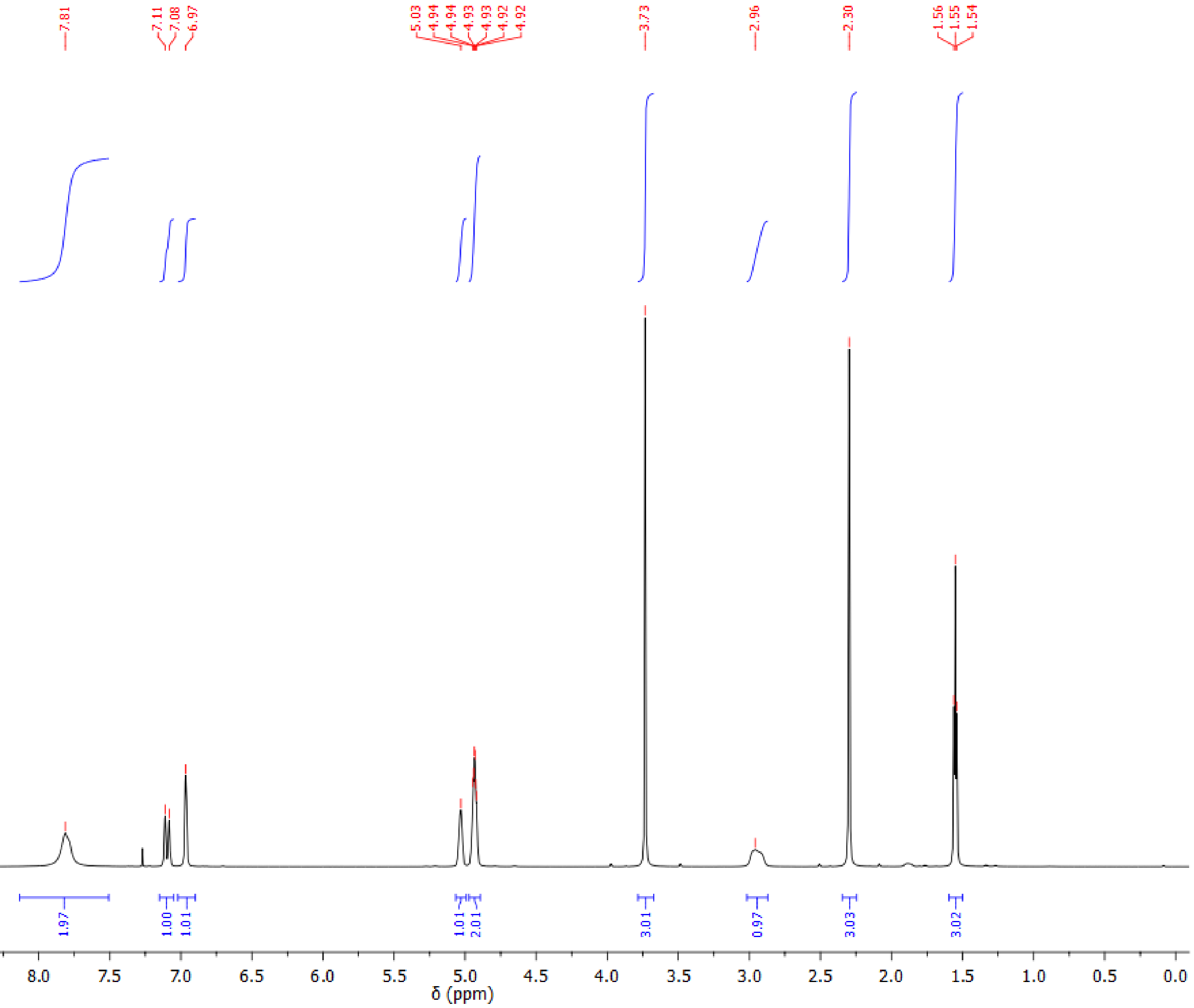

^{13}C NMR (75 MHz, $CDCl_3$)

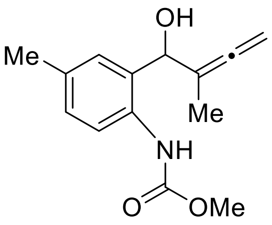

2i

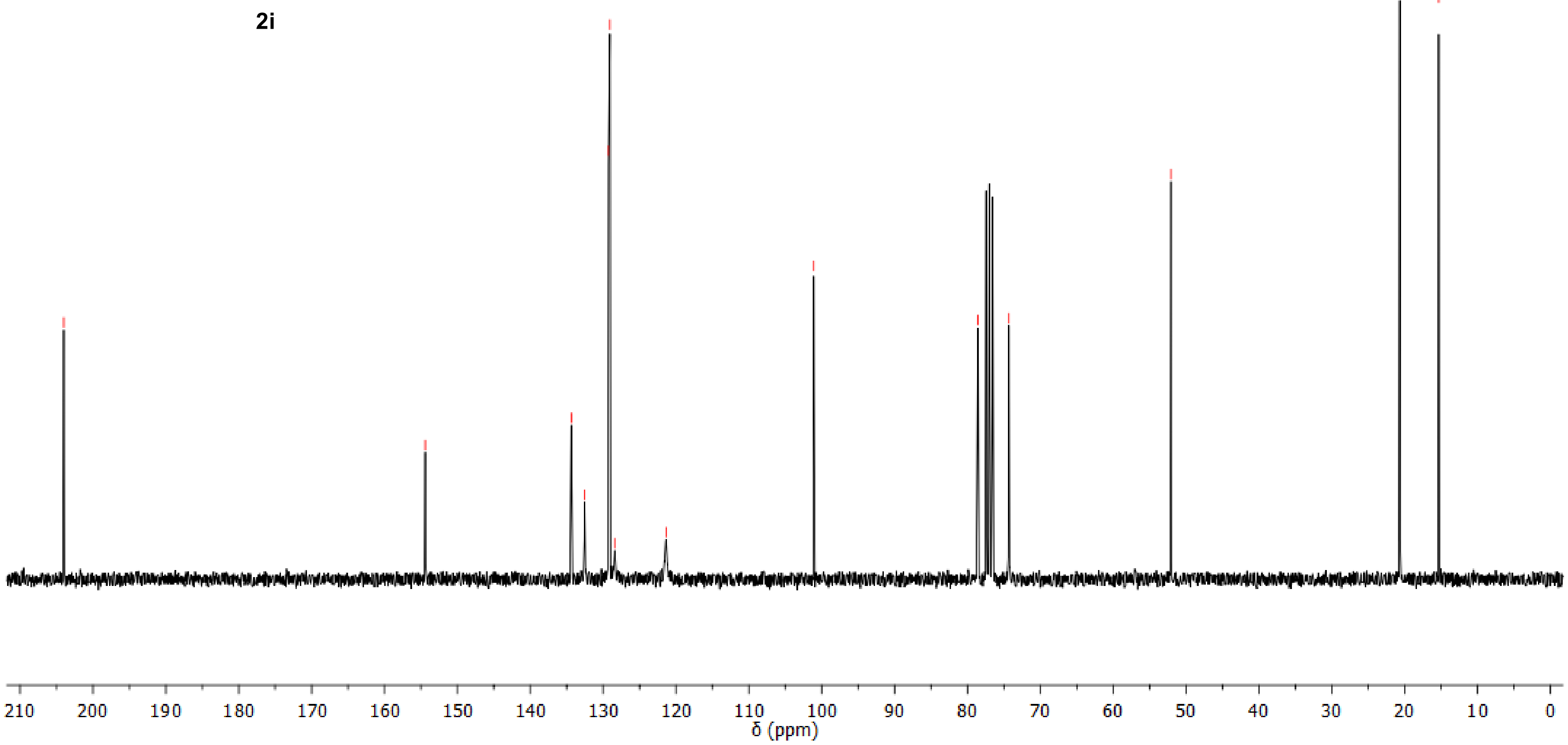

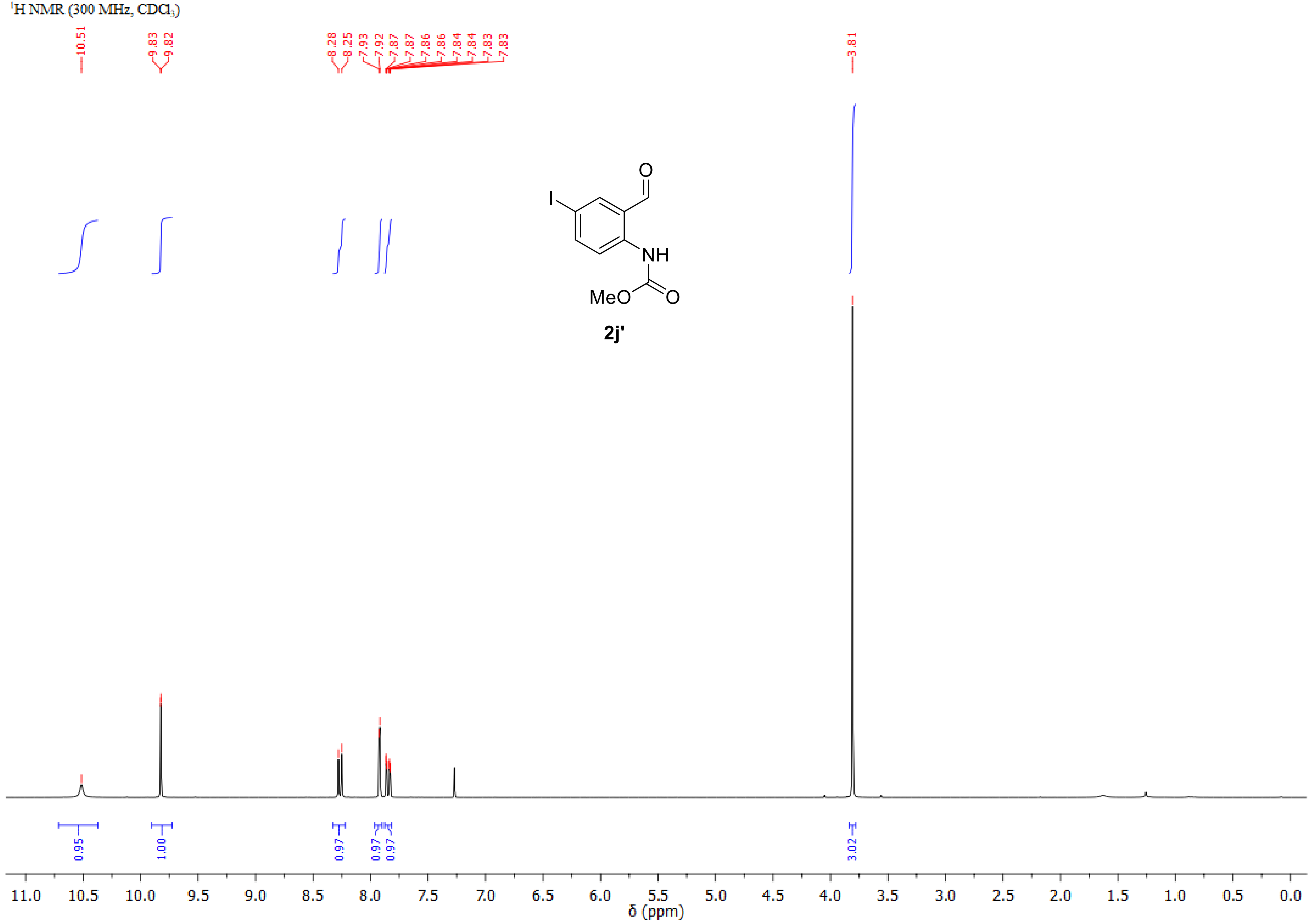

1H NMR (300 MHz, CDCl3)
10.51
9.83
9.82
8.28
8.25
7.93
7.92
7.87
7.87
7.86
7.86
7.84
7.84
7.83
7.83
3.81
I
O
NH
MeO
O
2j'
0.95
1.00
0.97
0.97
0.97
3.02
11.0
10.5
10.0
9.5
9.0
8.5
8.0
7.5
7.0
6.5
6.0
5.5
5.0
4.5
4.0
3.5
3.0
2.5
2.0
1.5
1.0
0.5
0.0
δ (ppm)

^{13}C NMR (75 MHz, $CDCl_3$)

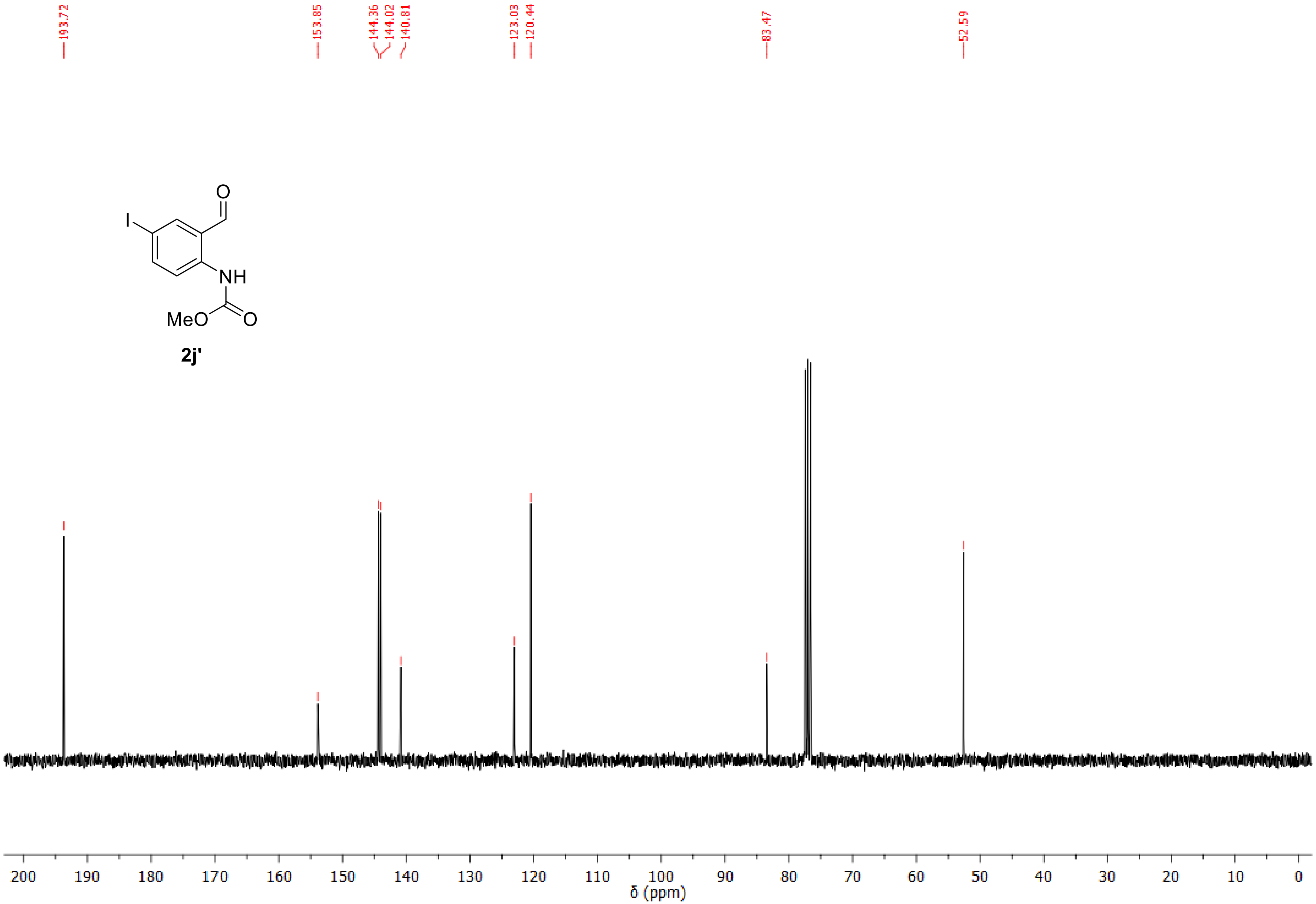

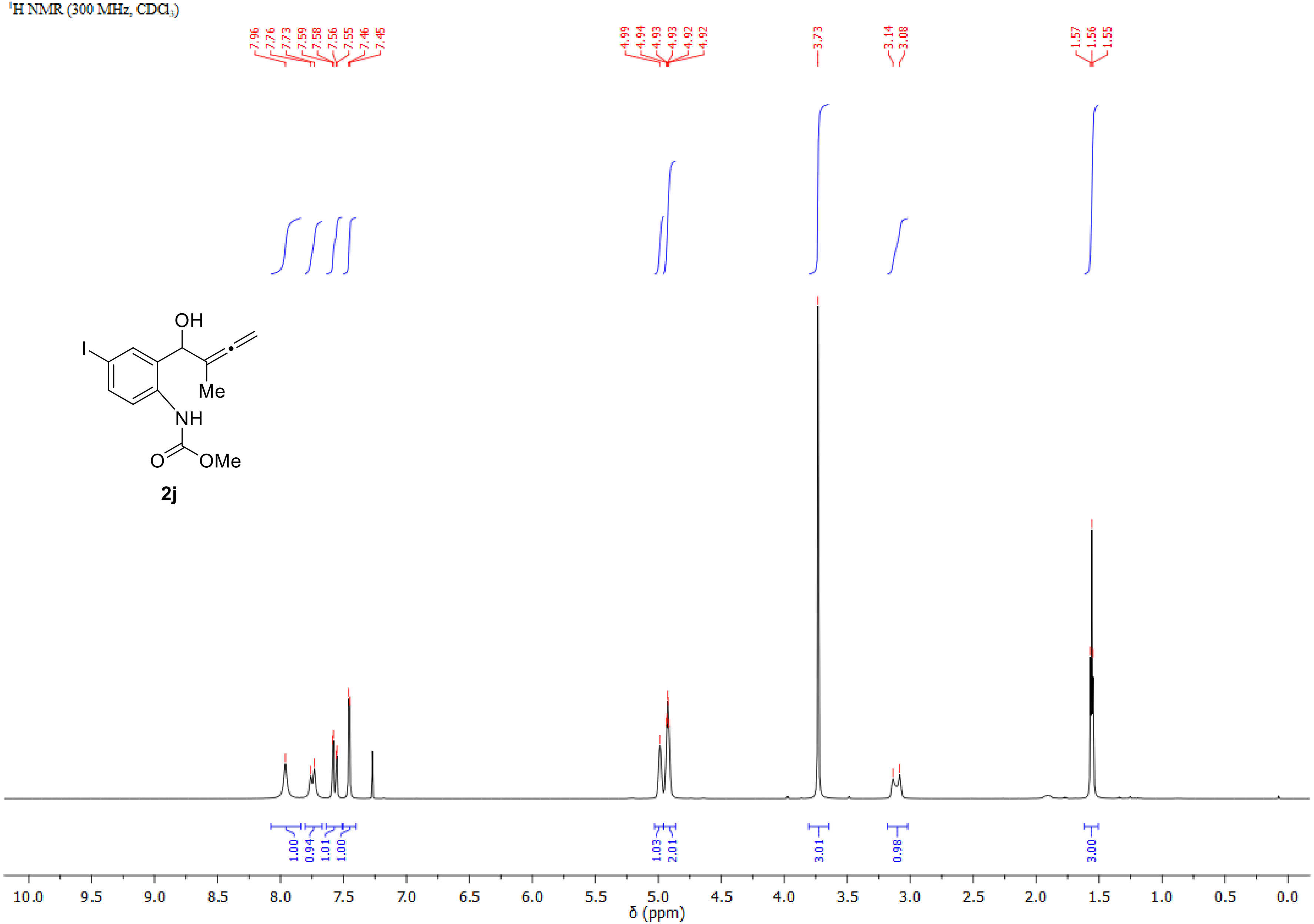
¹H NMR (300 MHz, CDCl₃)
7.96 7.76 7.73 7.59 7.58 7.56 7.55 7.46 7.45
4.99 4.94 4.93 4.93 4.92 4.92
3.73
3.14 3.08
1.57 1.56 1.55
OH
I
Me
NH
O
OMe
2j
1.00 0.94 1.01 1.00
1.03 2.01
3.01
0.98
3.00
10.0 9.5 9.0 8.5 8.0 7.5 7.0 6.5 6.0 5.5 5.0 4.5 4.0 3.5 3.0 2.5 2.0 1.5 1.0 0.5 0.0
δ (ppm)

^{13}C NMR (75 MHz, $CDCl_3$)

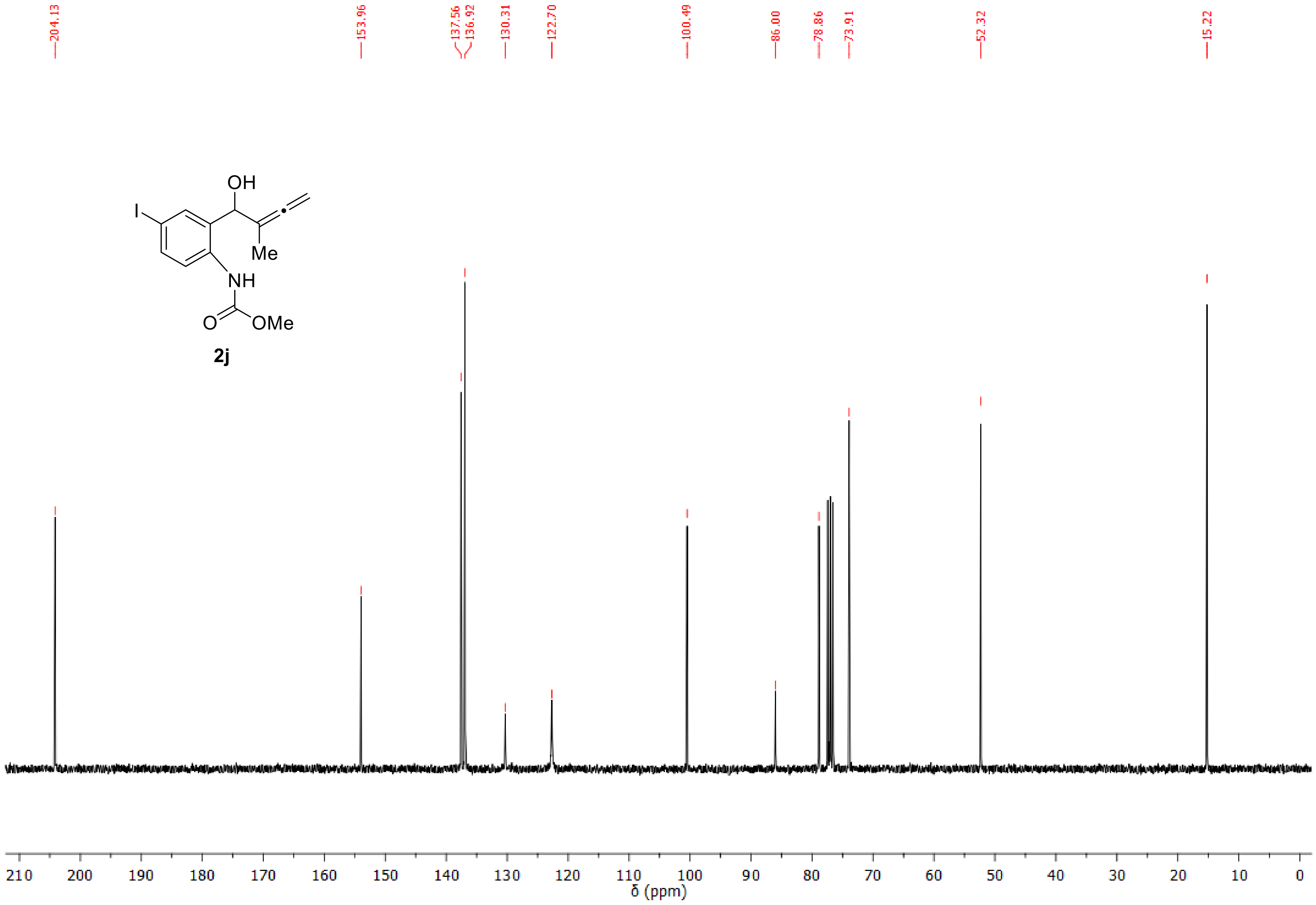

^{1}H NMR (300 MHz, $CDCl_3$)

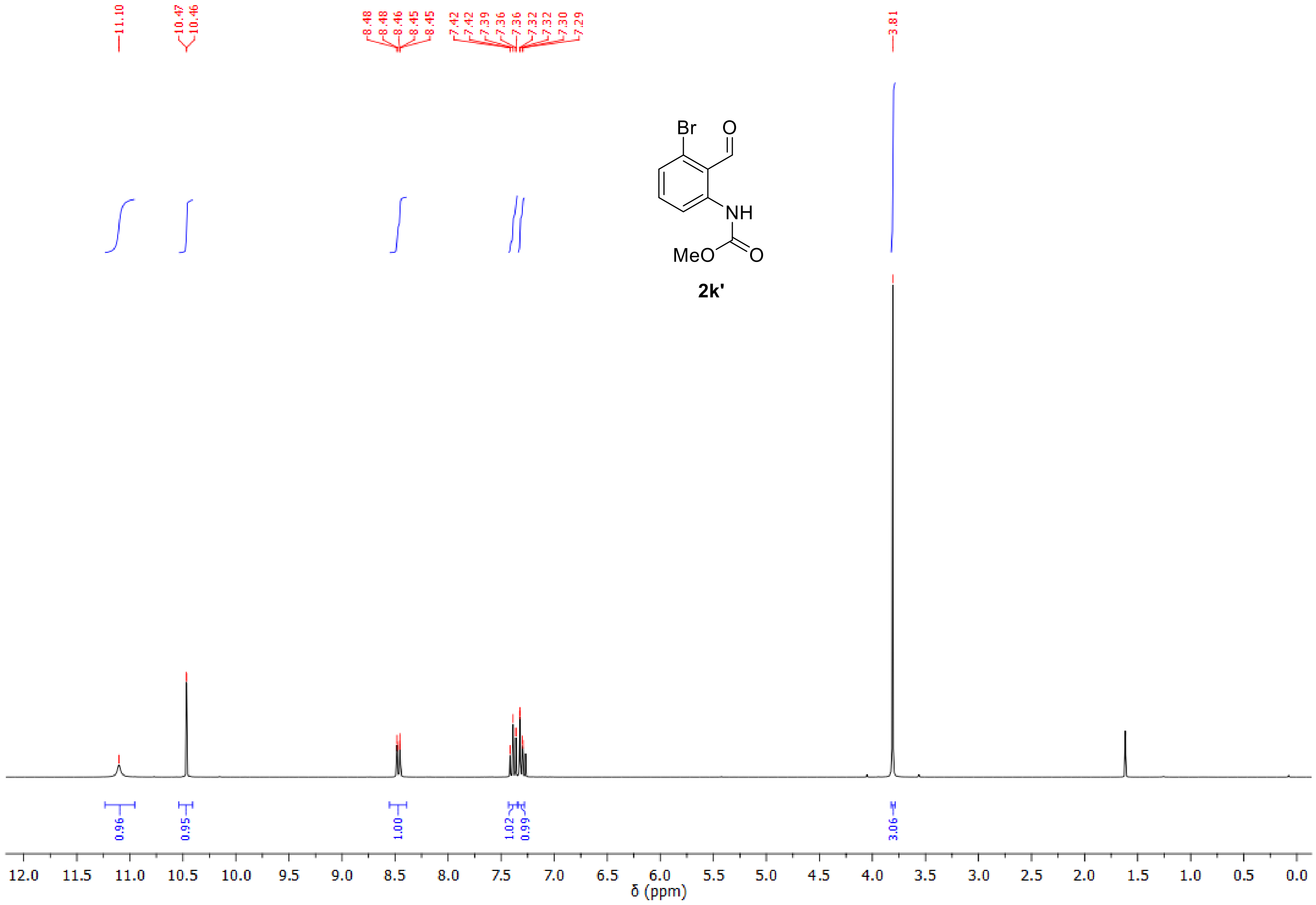

^{13}C NMR (75 MHz, $CDCl_3$)

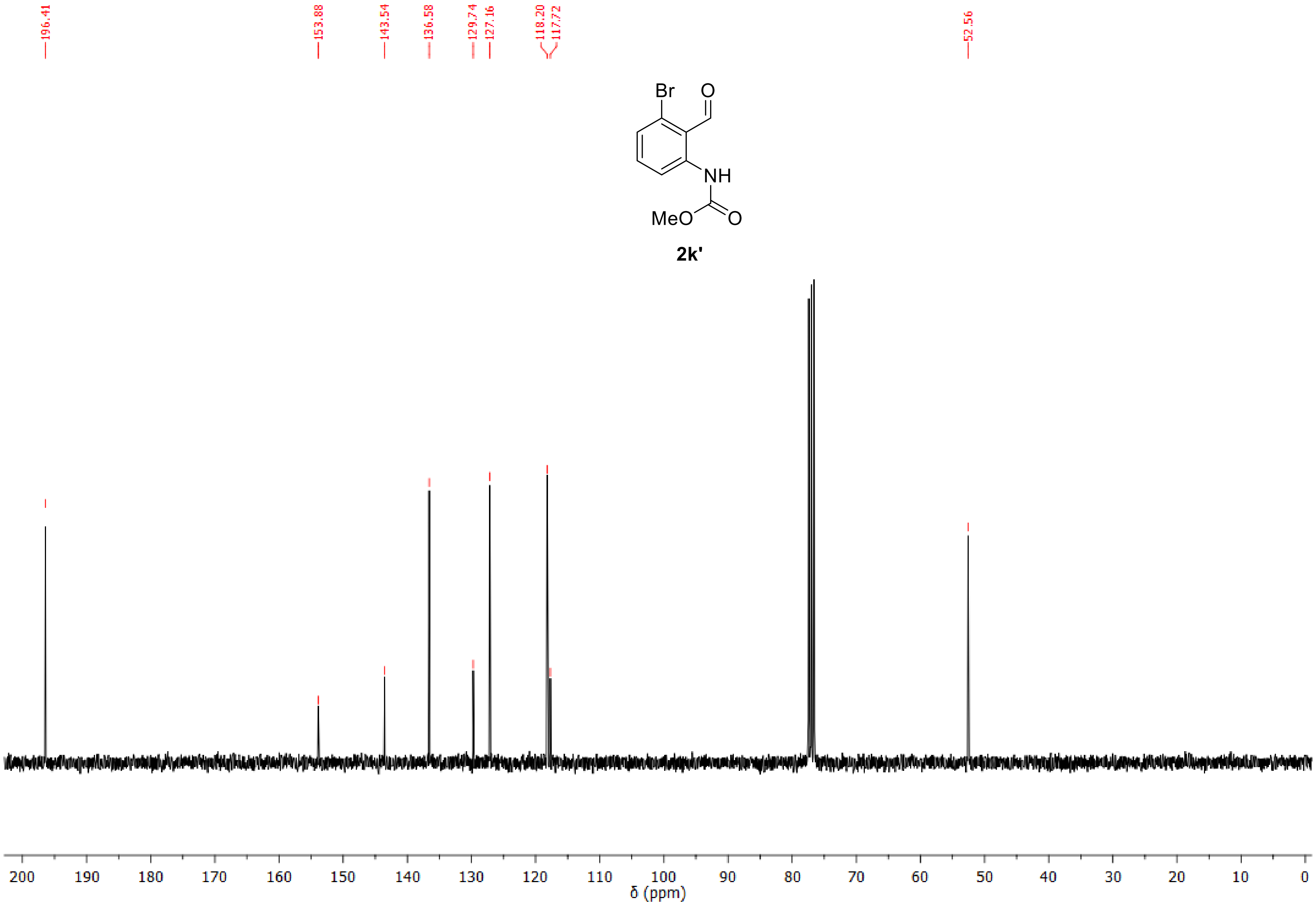

^{1}H NMR (300 MHz, $CDCl_3$)

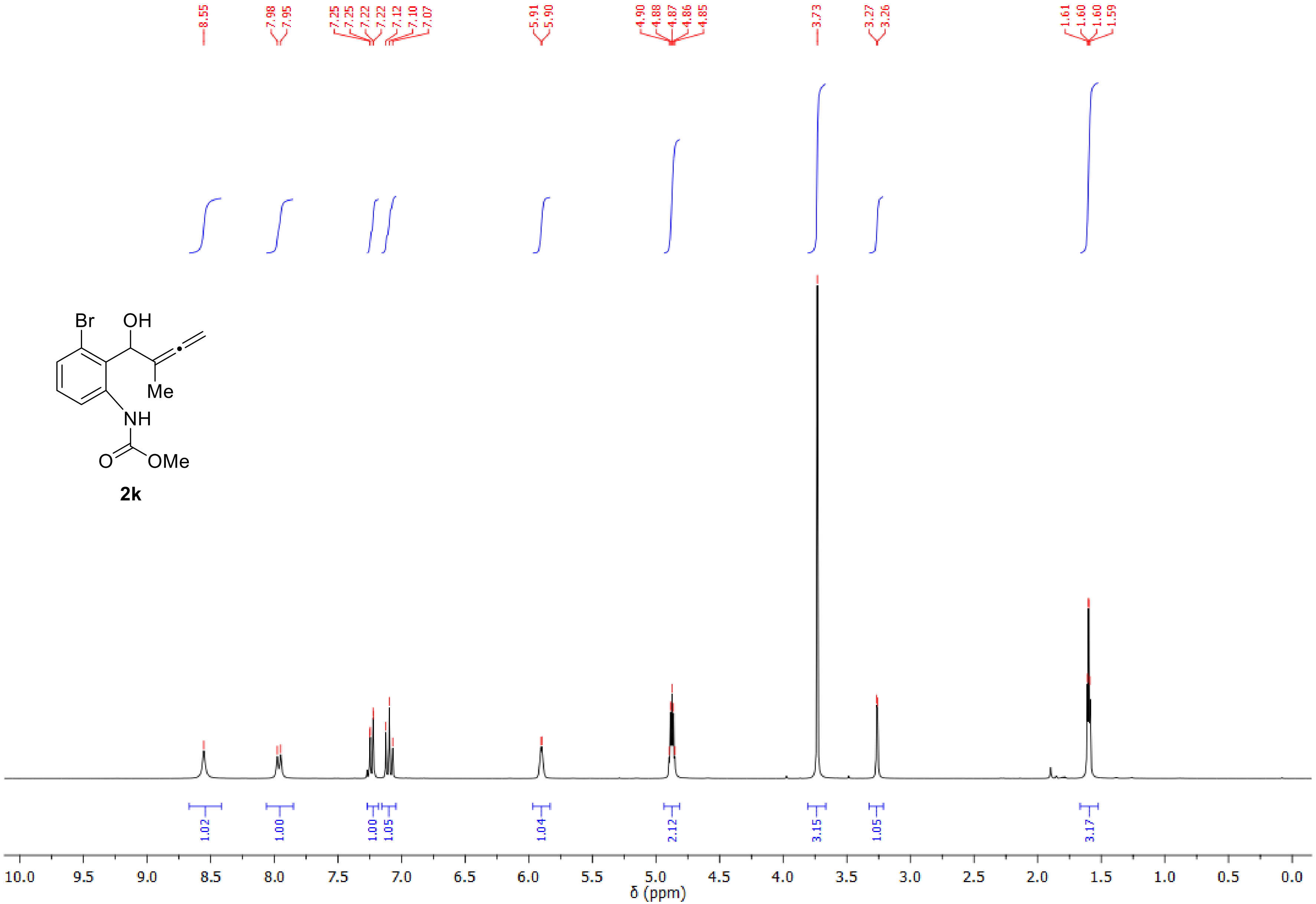

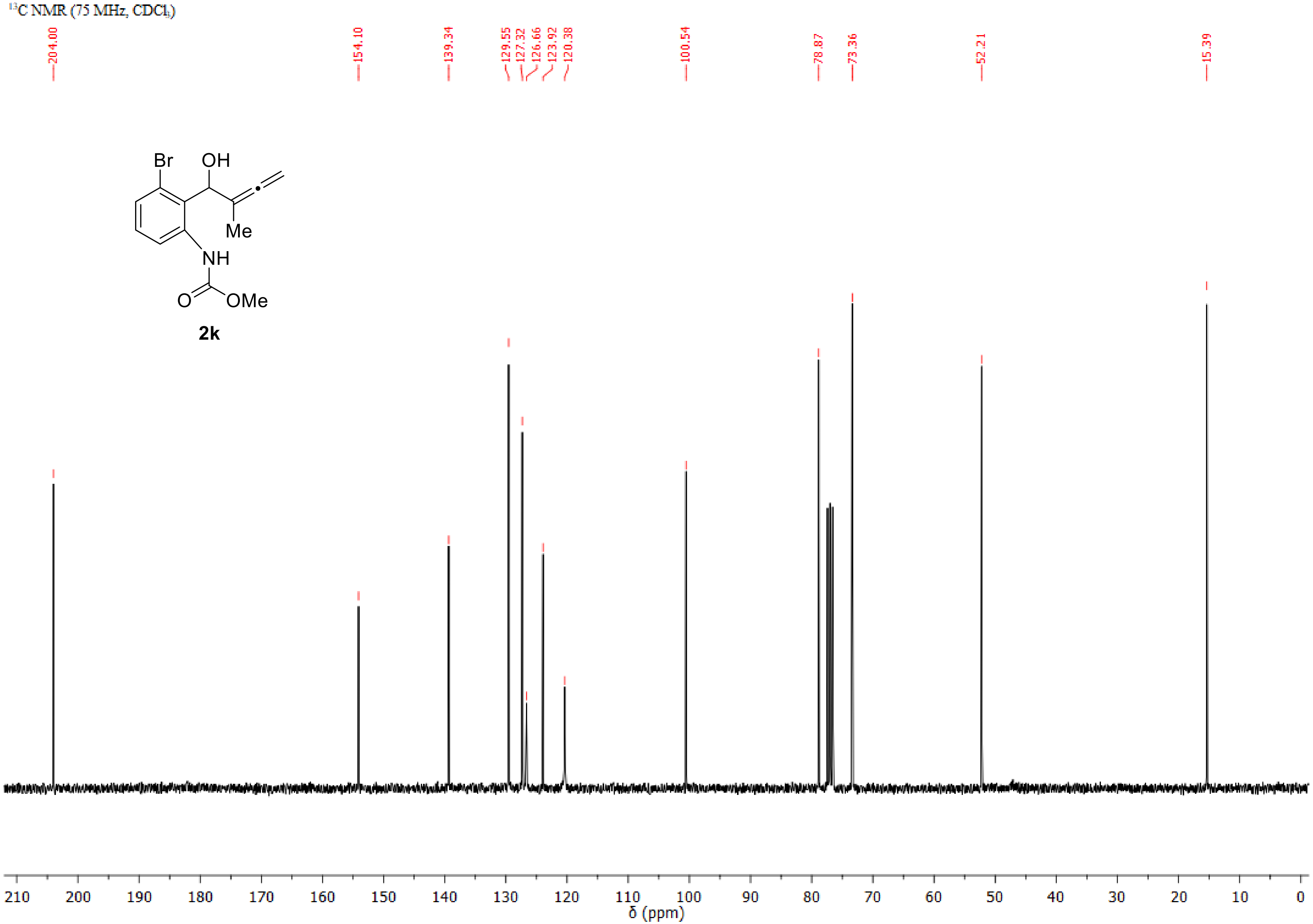

13C NMR (75 MHz, CDCl3)
204.00
154.10
139.34
129.55
127.32
126.66
123.92
120.38
100.54
78.87
73.36
52.21
15.39
Br
OH
Me
NH
O
OMe
2k
210
200
190
180
170
160
150
140
130
120
110
100
90
80
70
60
50
40
30
20
10
0
δ (ppm)

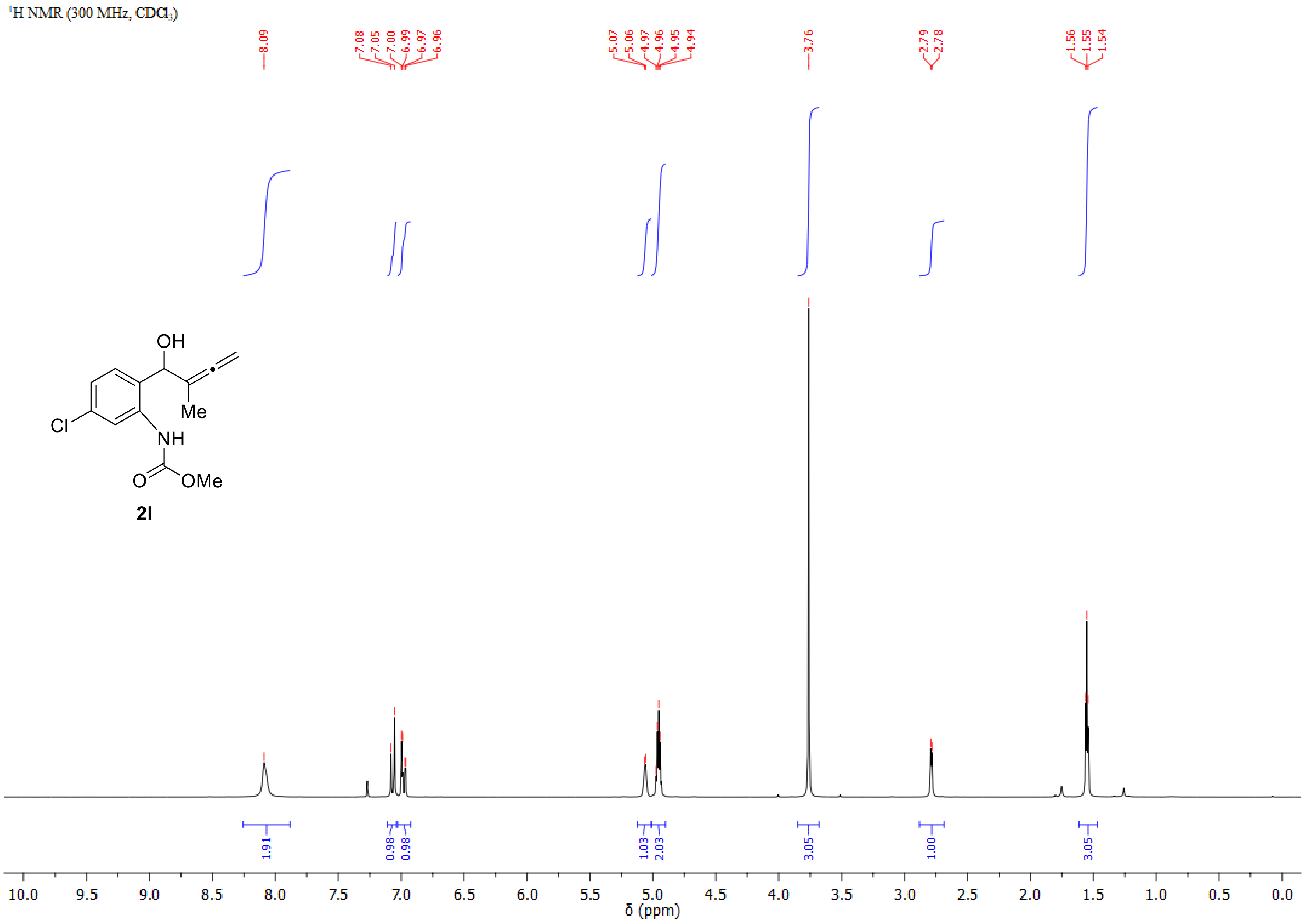

¹H NMR (300 MHz, CDCl3)
8.09
7.08
7.05
7.00
6.99
6.97
6.96
5.07
5.06
4.97
4.96
4.95
4.94
3.76
2.79
2.78
1.56
1.55
1.54
OH
Me
Cl
NH
O
OMe
2l
1.91
0.98
0.98
1.03
2.03
3.05
1.00
3.05
10.0
9.5
9.0
8.5
8.0
7.5
7.0
6.5
6.0
5.5
5.0
4.5
4.0
3.5
3.0
2.5
2.0
1.5
1.0
0.5
0.0
δ (ppm)

^{13}C NMR (75 MHz, $CDCl_3$)

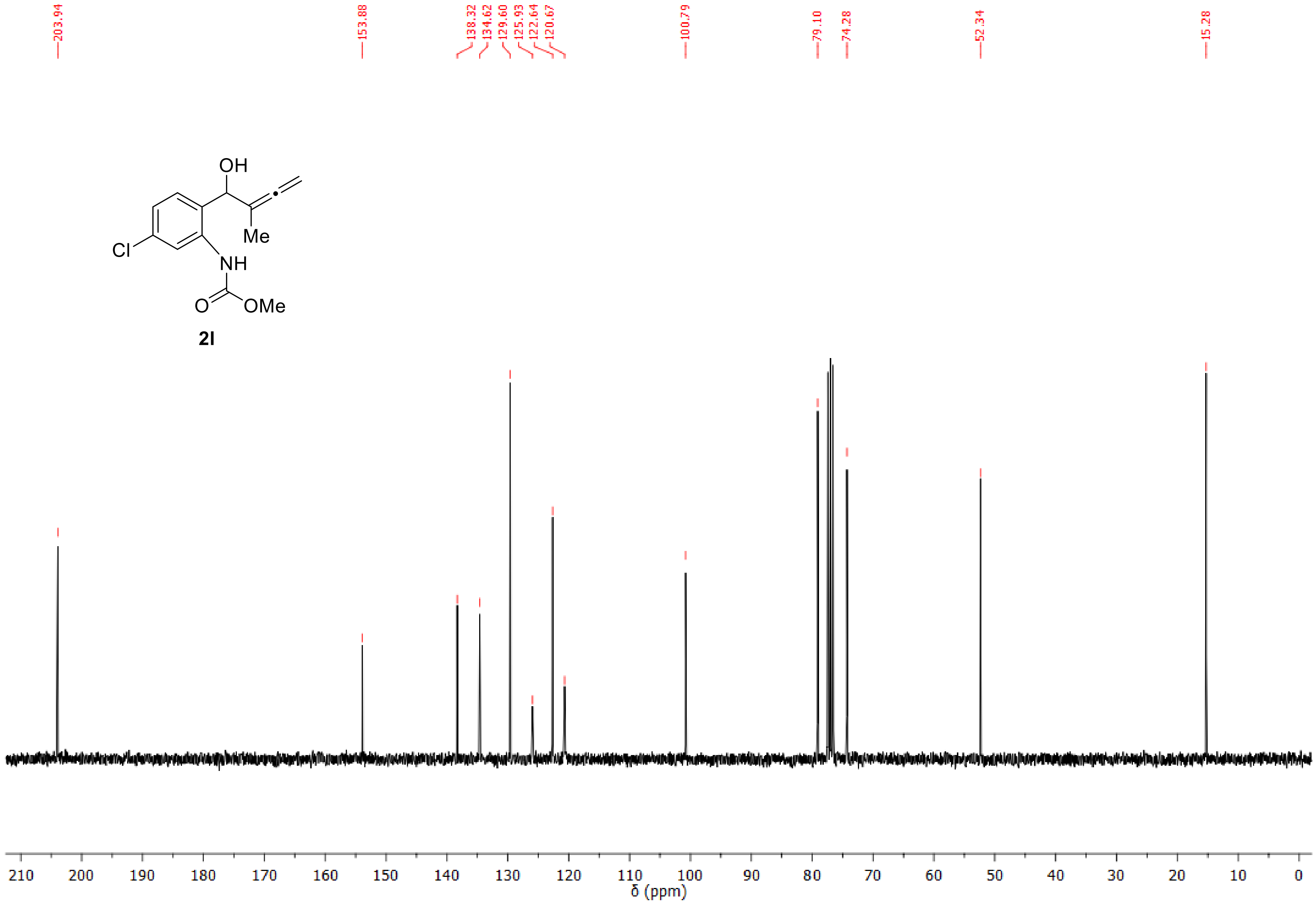

^1H NMR (300 MHz, $CDCl_3$)

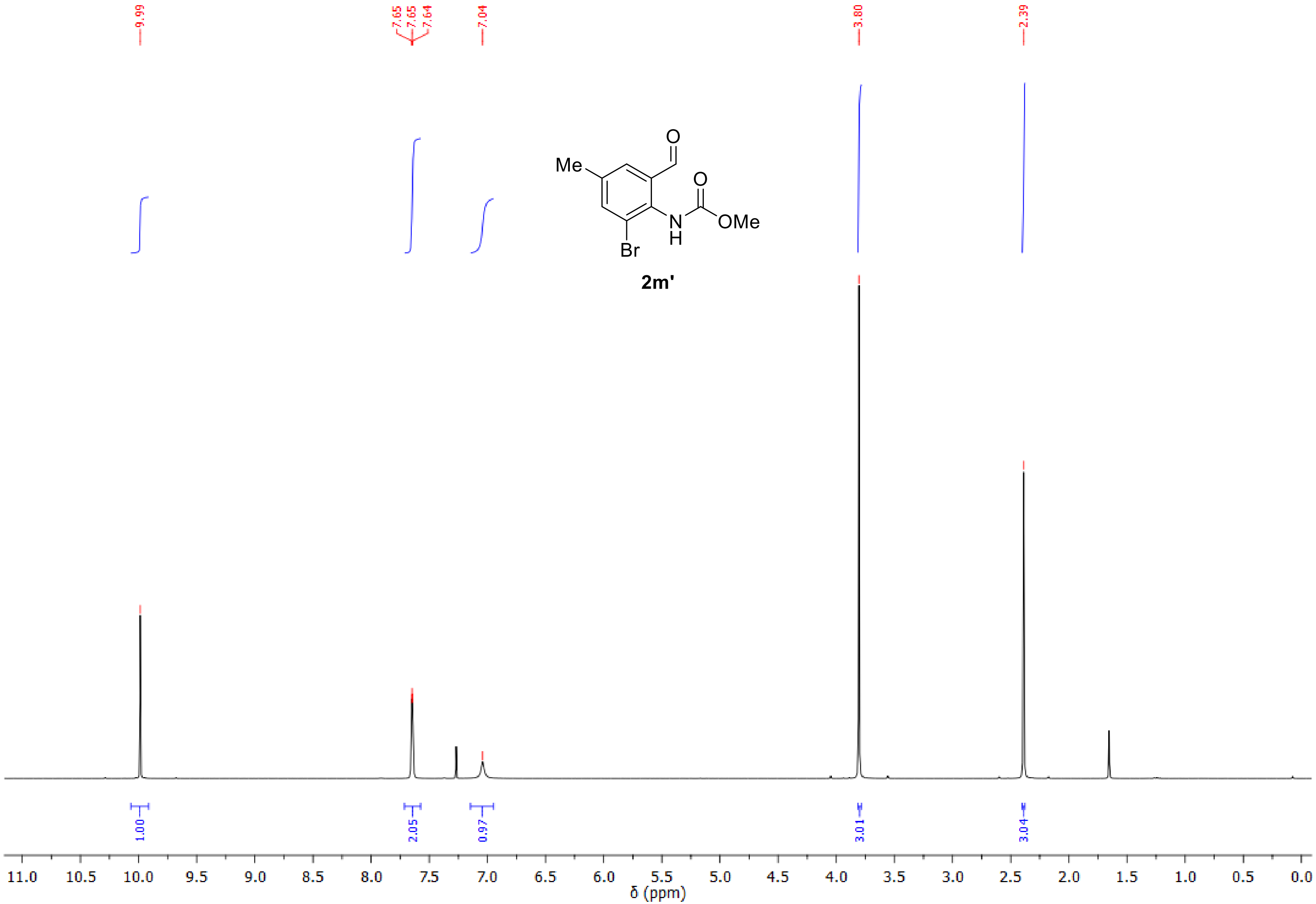

2m'

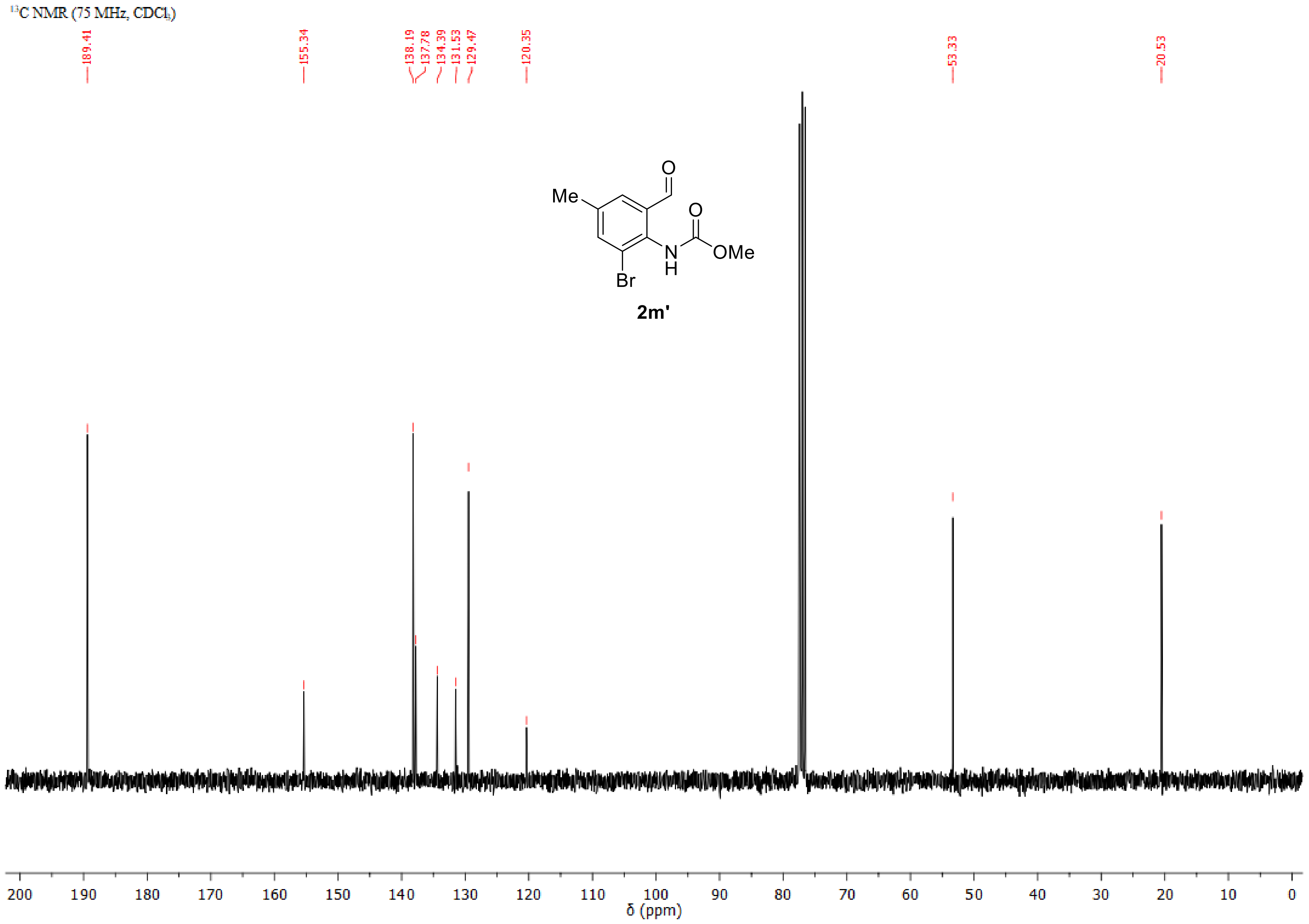

13C NMR (75 MHz, CDCl3)
189.41
155.34
138.19
137.78
134.39
131.53
129.47
120.35
53.33
20.53
Me
O
O
N
H
OMe
Br
2m'
200 190 180 170 160 150 140 130 120 110 100 90 80 70 60 50 40 30 20 10 0
δ (ppm)

^{1}H NMR (300 MHz, CDCl$_3$)

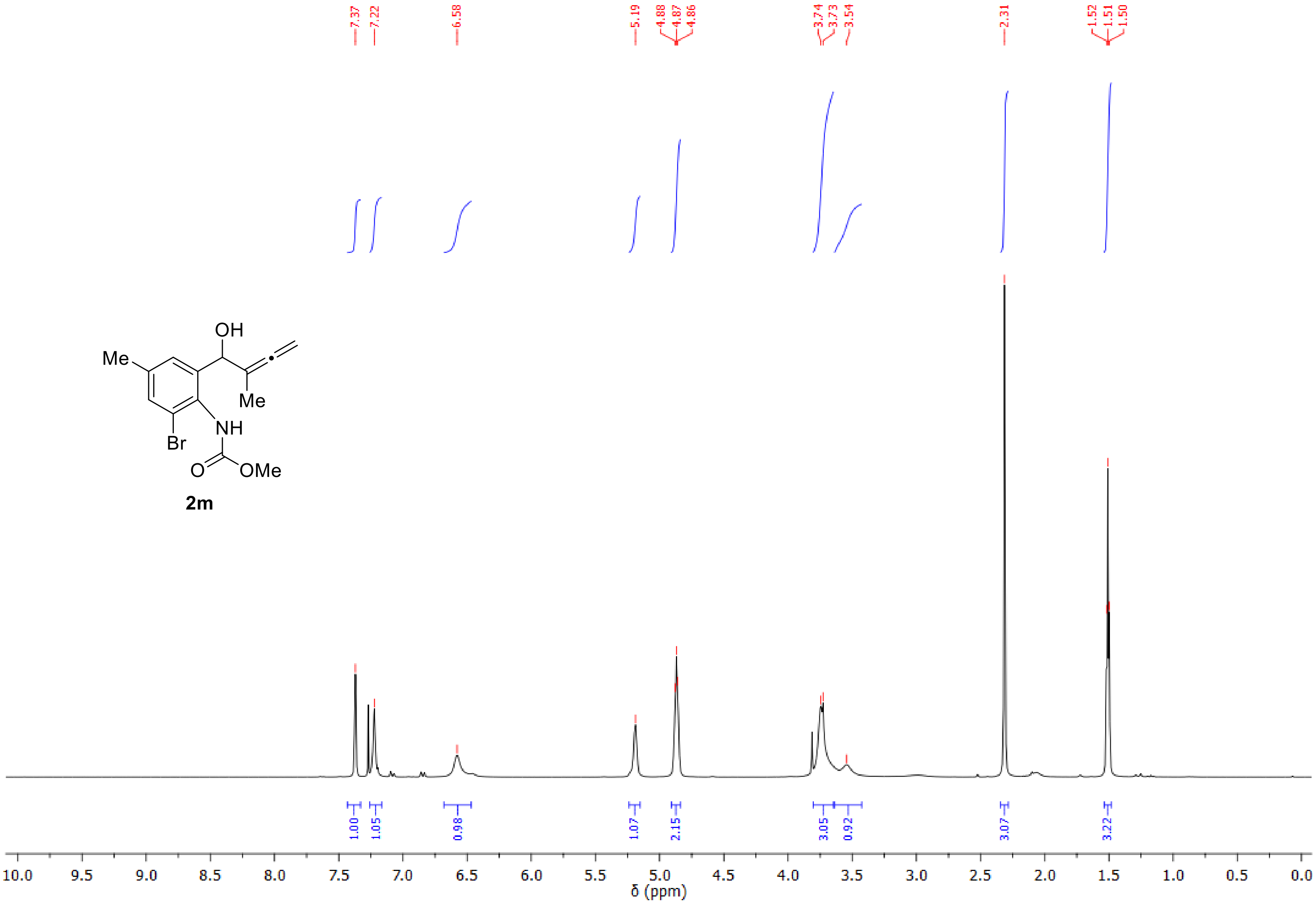

^{13}C NMR (75 MHz, $CDCl_3$)

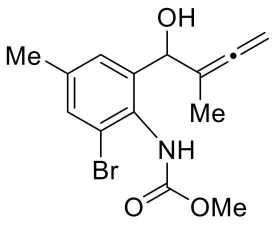

2m

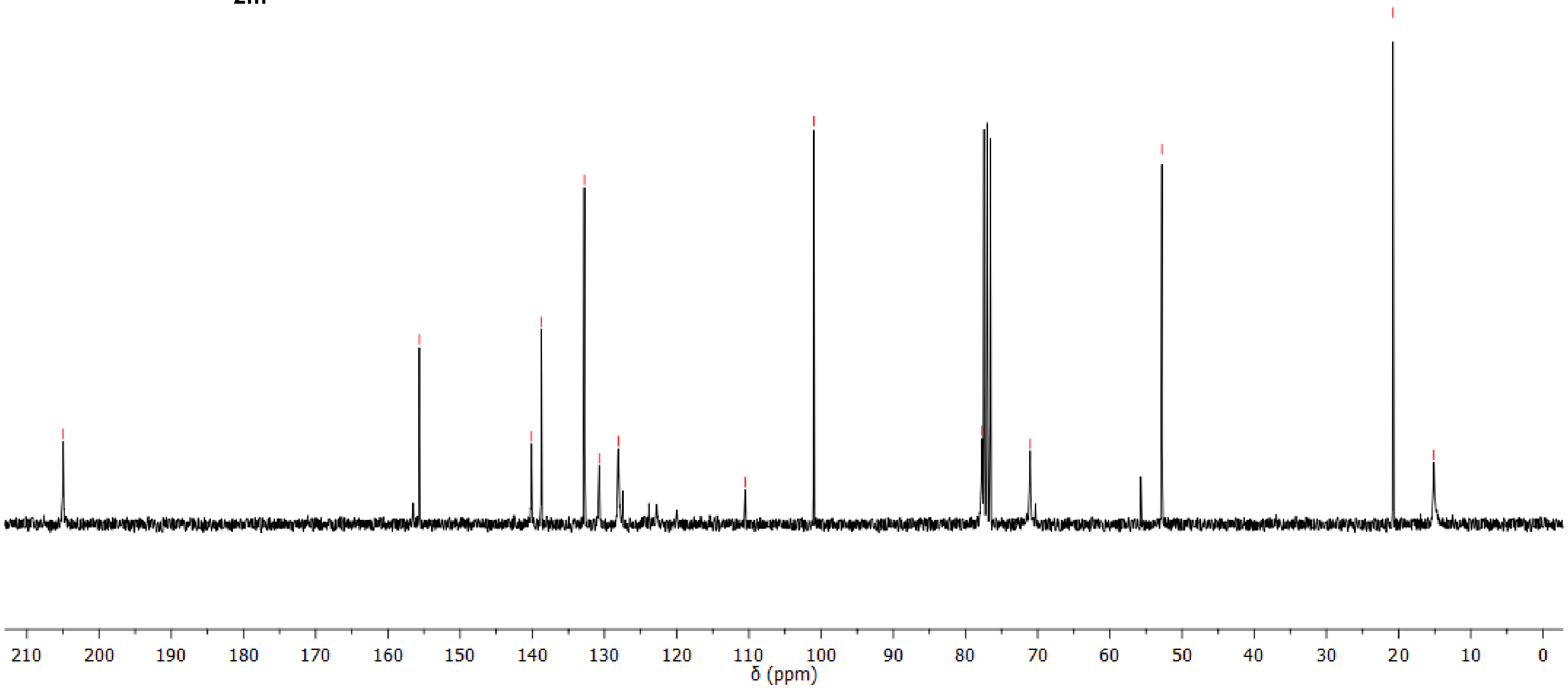

^1H NMR (300 MHz, $CDCl_3$)

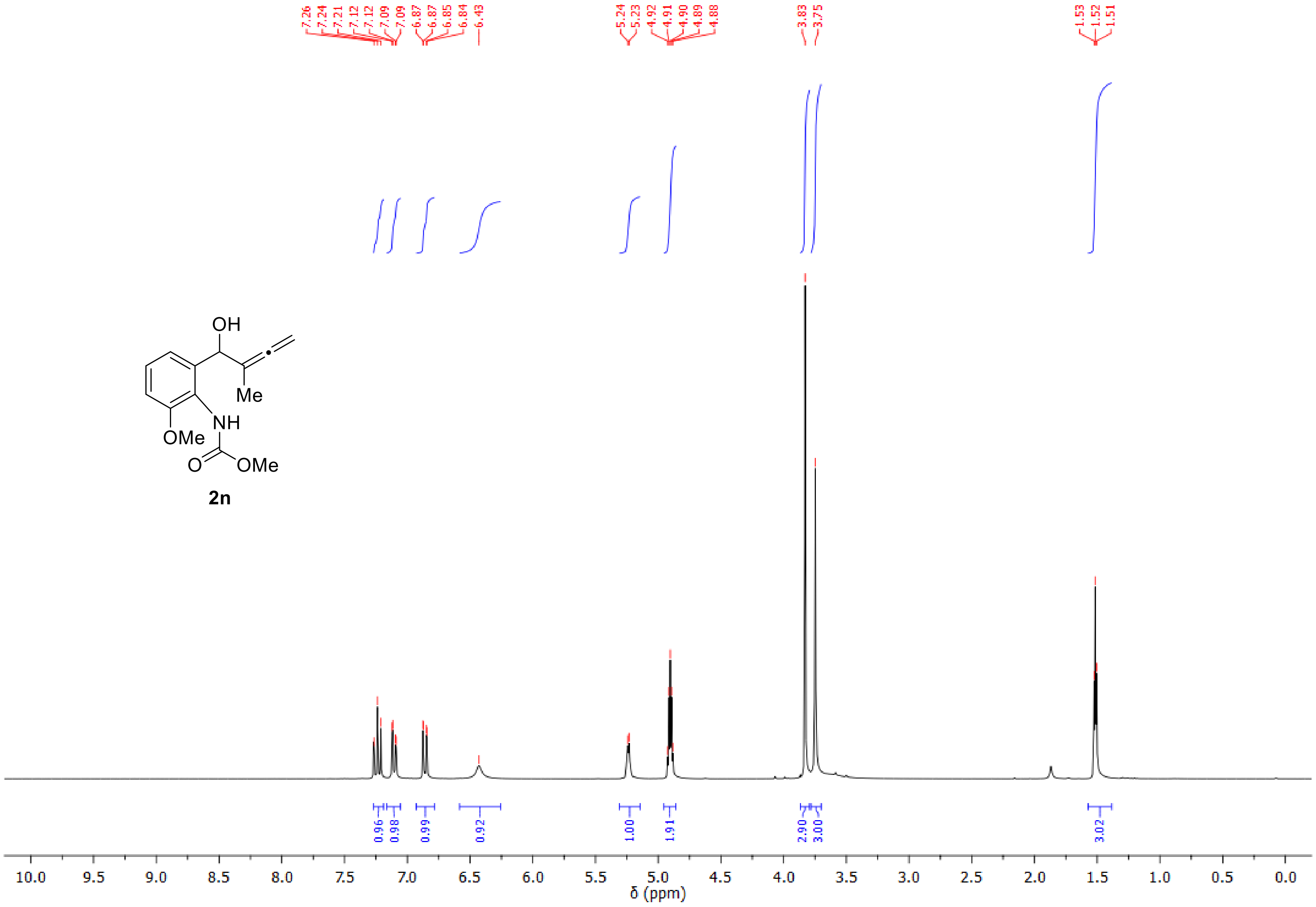

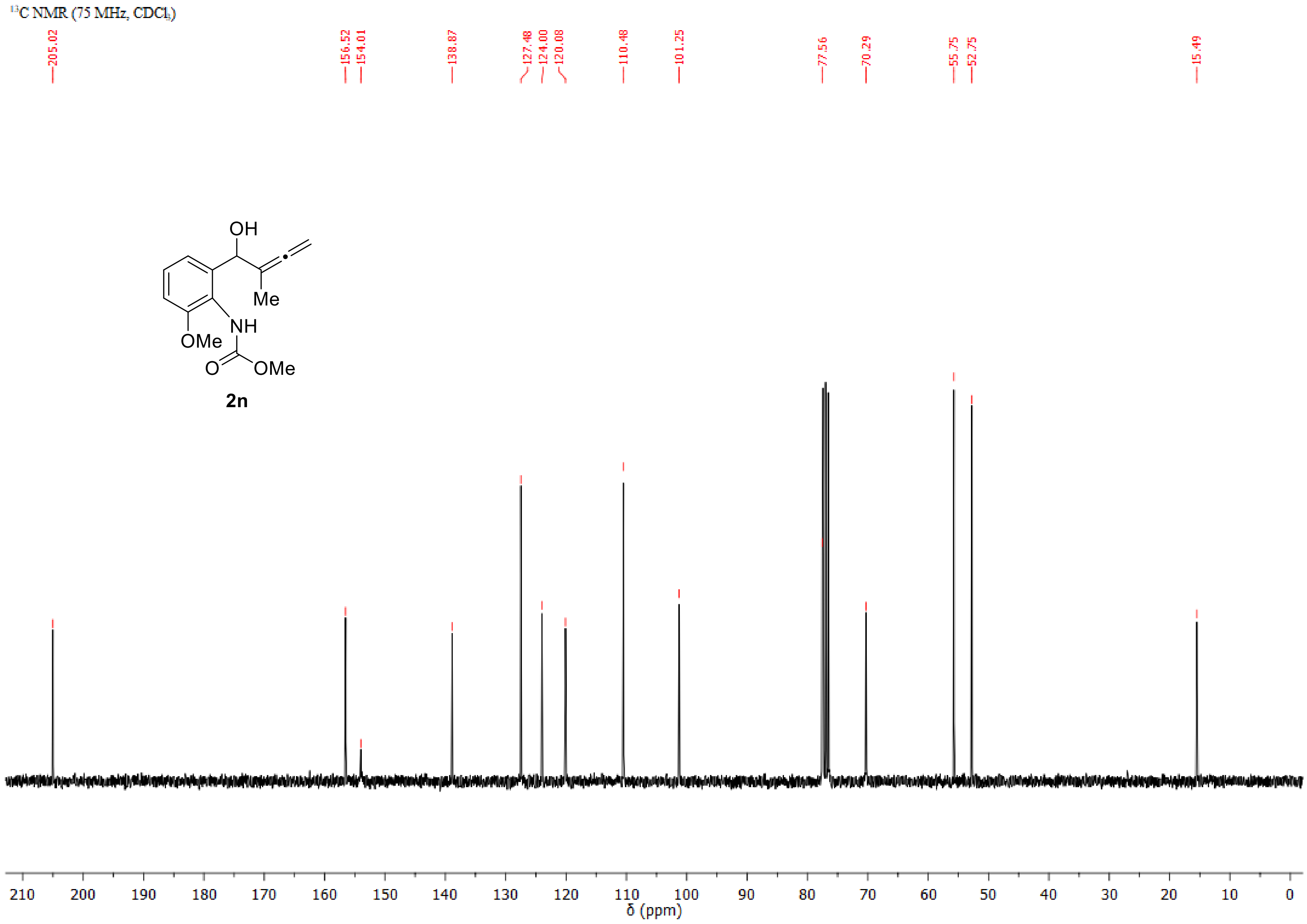

13C NMR (75 MHz, CDCl3)
205.02
156.52
154.01
138.87
127.48
124.00
120.08
110.48
101.25
77.56
70.29
55.75
52.75
15.49
OH
Me
NH
OMe
O
OMe
2n
210 200 190 180 170 160 150 140 130 120 110 100 90 80 70 60 50 40 30 20 10 0
δ (ppm)

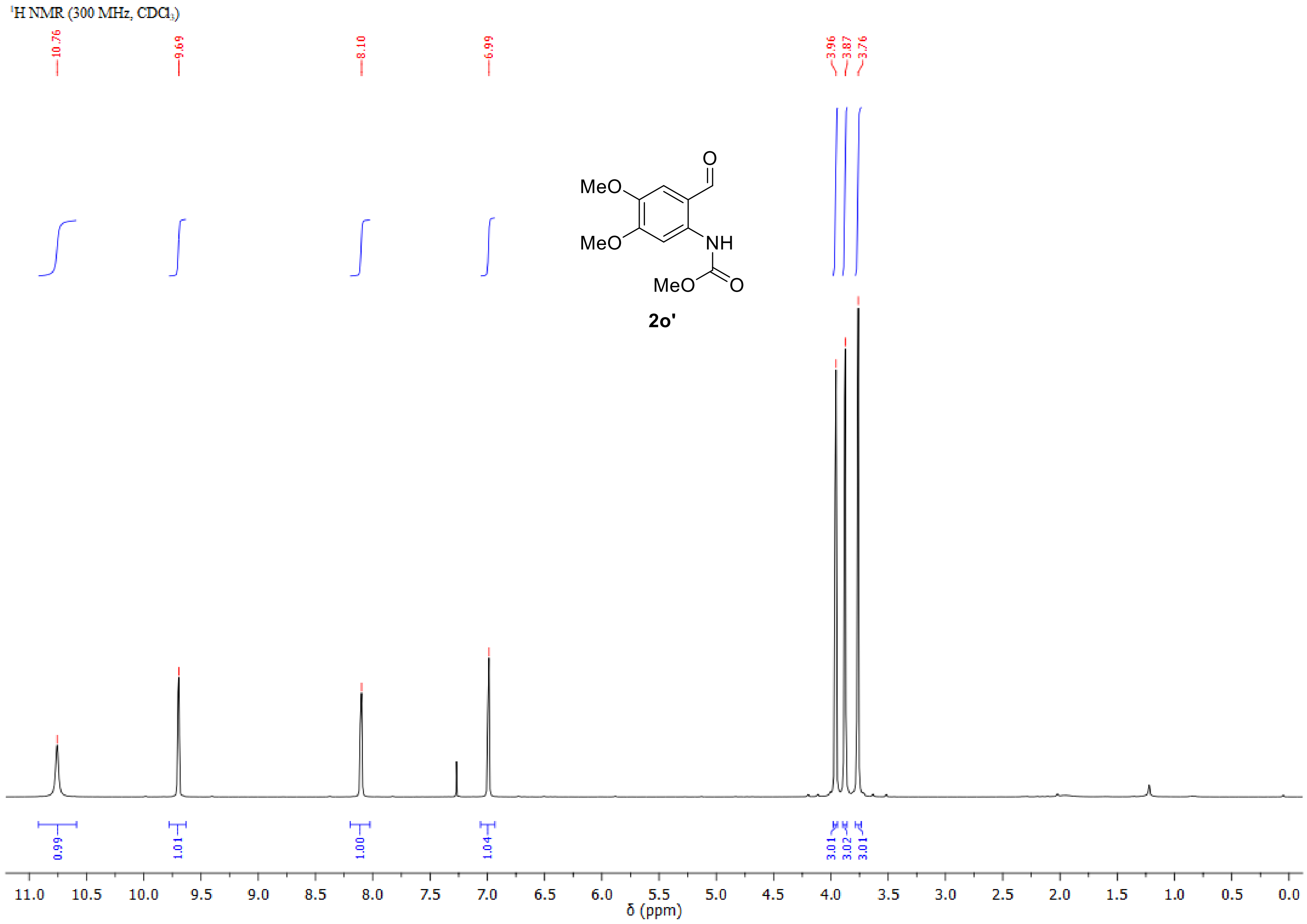

1H NMR (300 MHz, CDCl3)
10.76
9.69
8.10
6.99
3.96
3.87
3.76
MeO
MeO
O
NH
MeO
O
2o'
0.99
1.01
1.00
1.04
3.01
3.02
3.01
11.0 10.5 10.0 9.5 9.0 8.5 8.0 7.5 7.0 6.5 6.0 5.5 5.0 4.5 4.0 3.5 3.0 2.5 2.0 1.5 1.0 0.5 0.0
δ (ppm)

^{13}C NMR (75 MHz, $CDCl_3$)

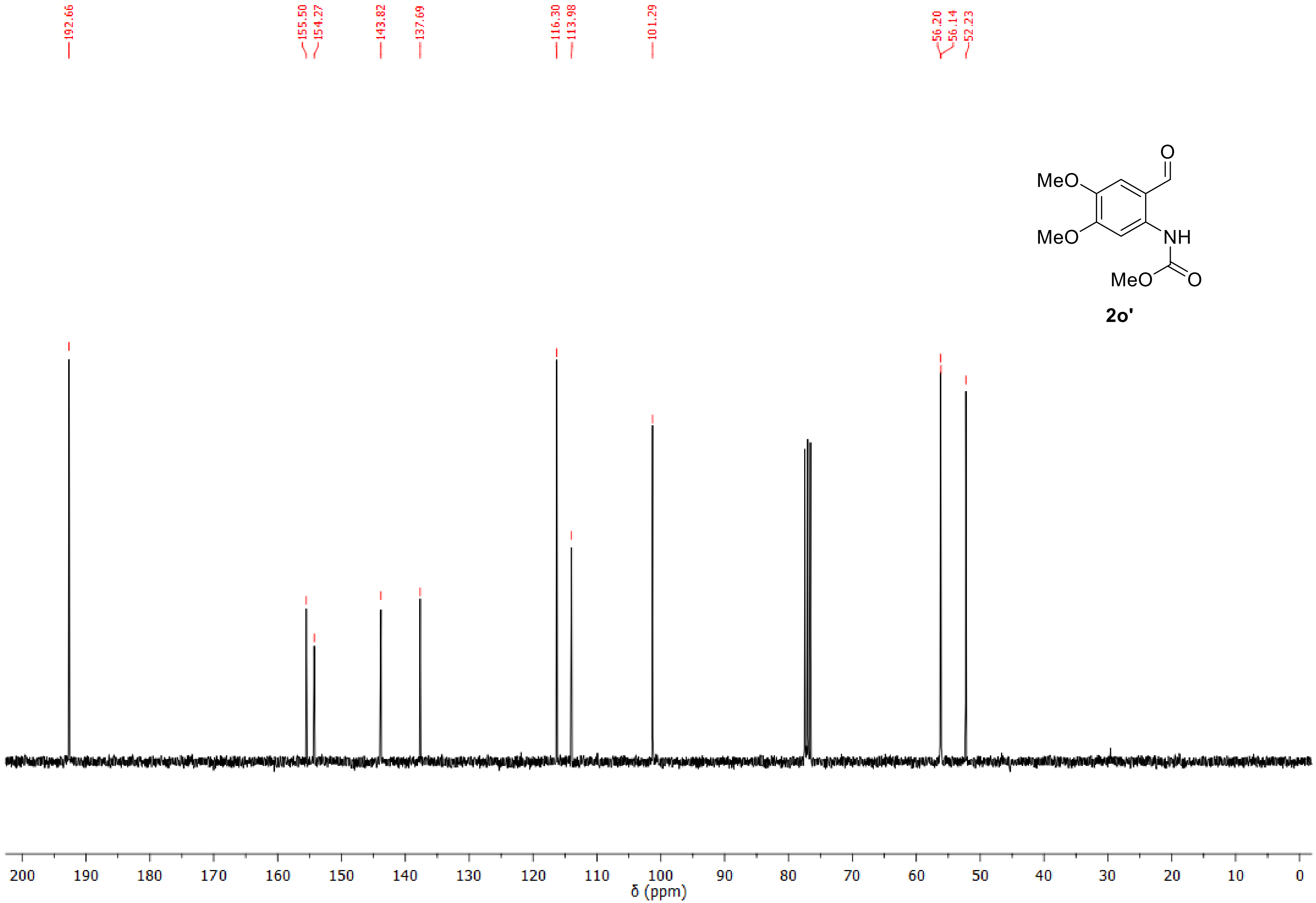

^{1}H NMR (300 MHz, $CDCl_3$)

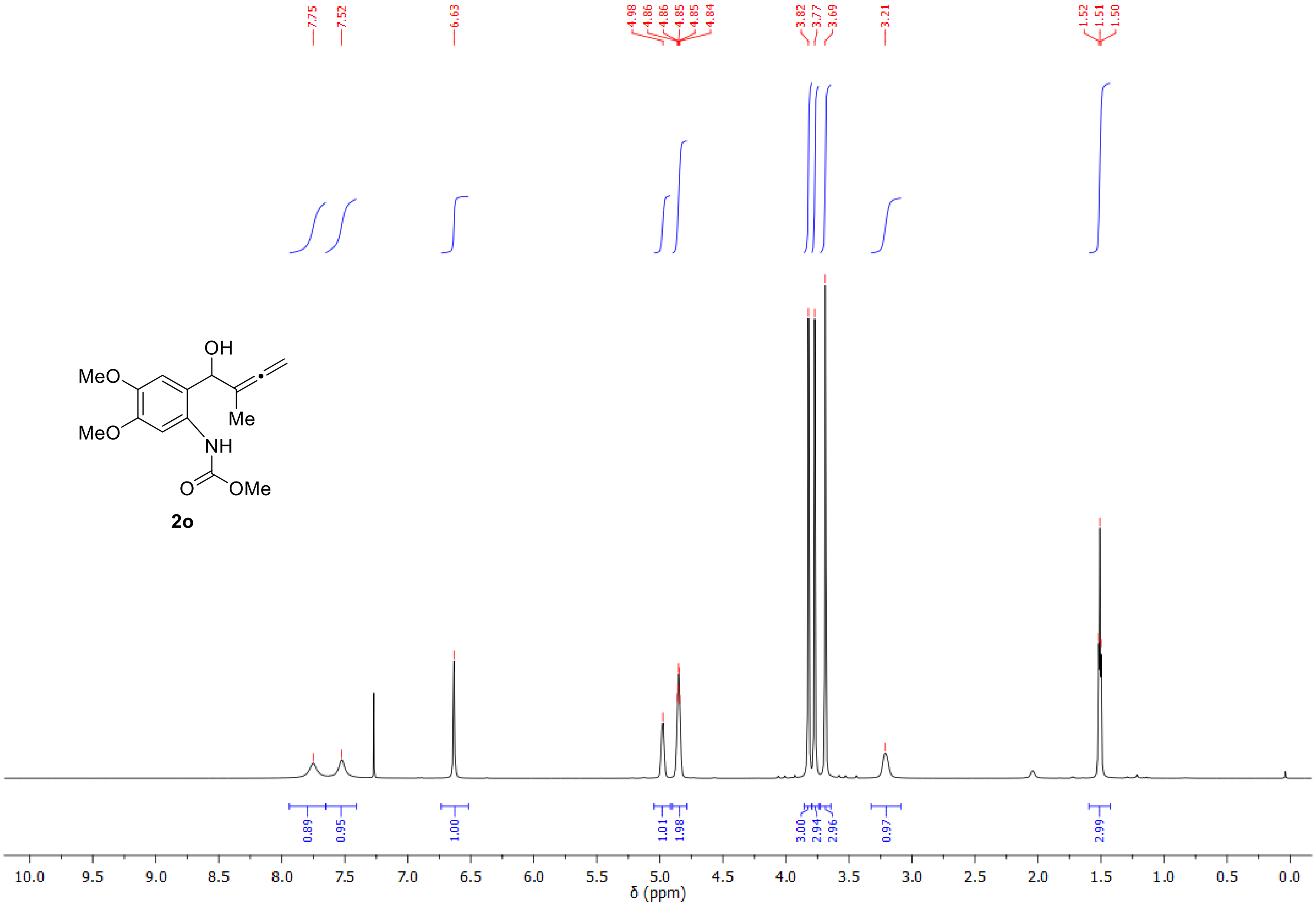

^{13}C NMR (75 MHz, $CDCl_3$)

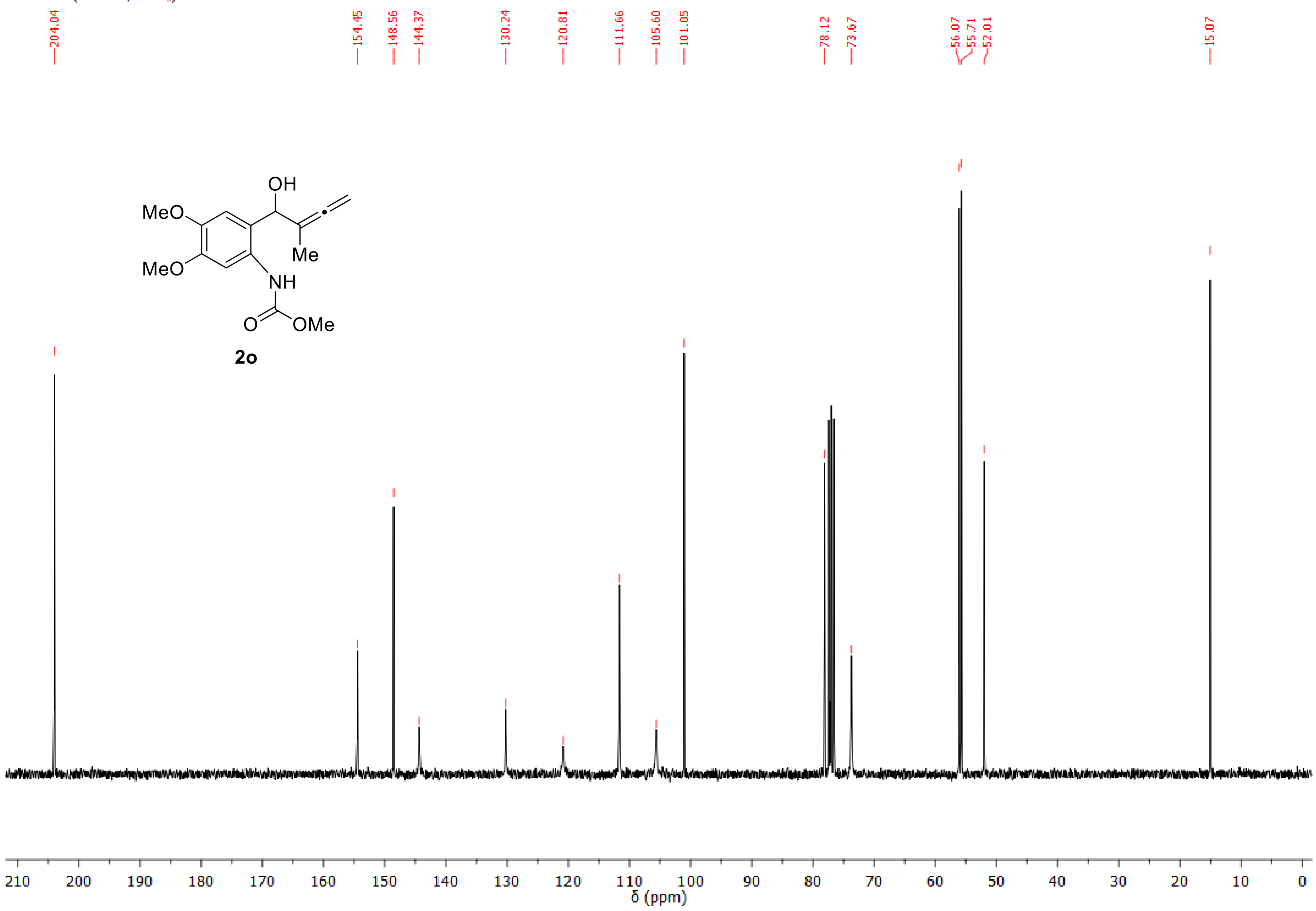

^{1}H NMR (500 MHz, CDCl$_3$)

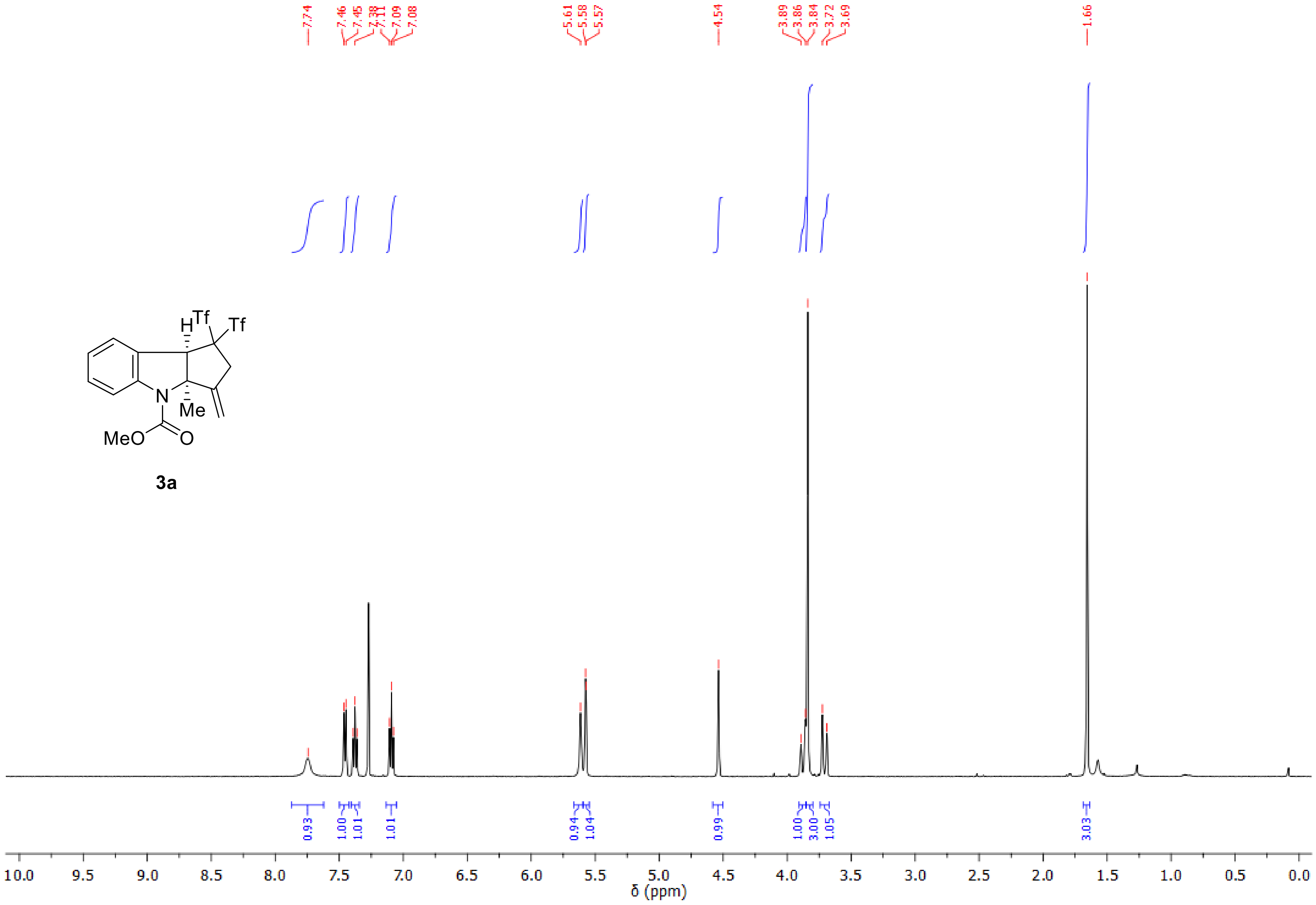

^{13}C NMR (125 MHz, $CDCl_3$)

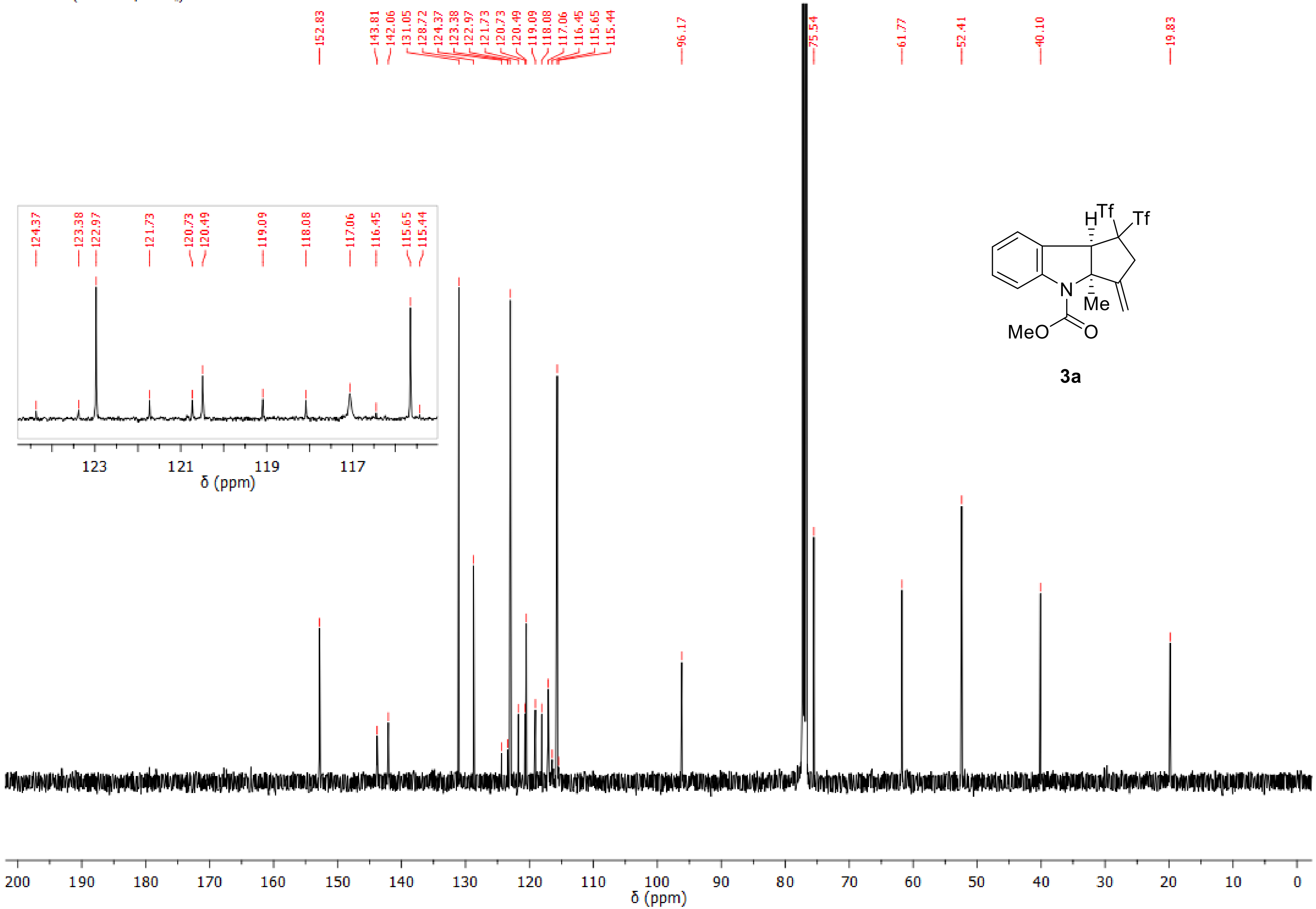

^{19}F NMR (282 MHz, CDCl$_3$)

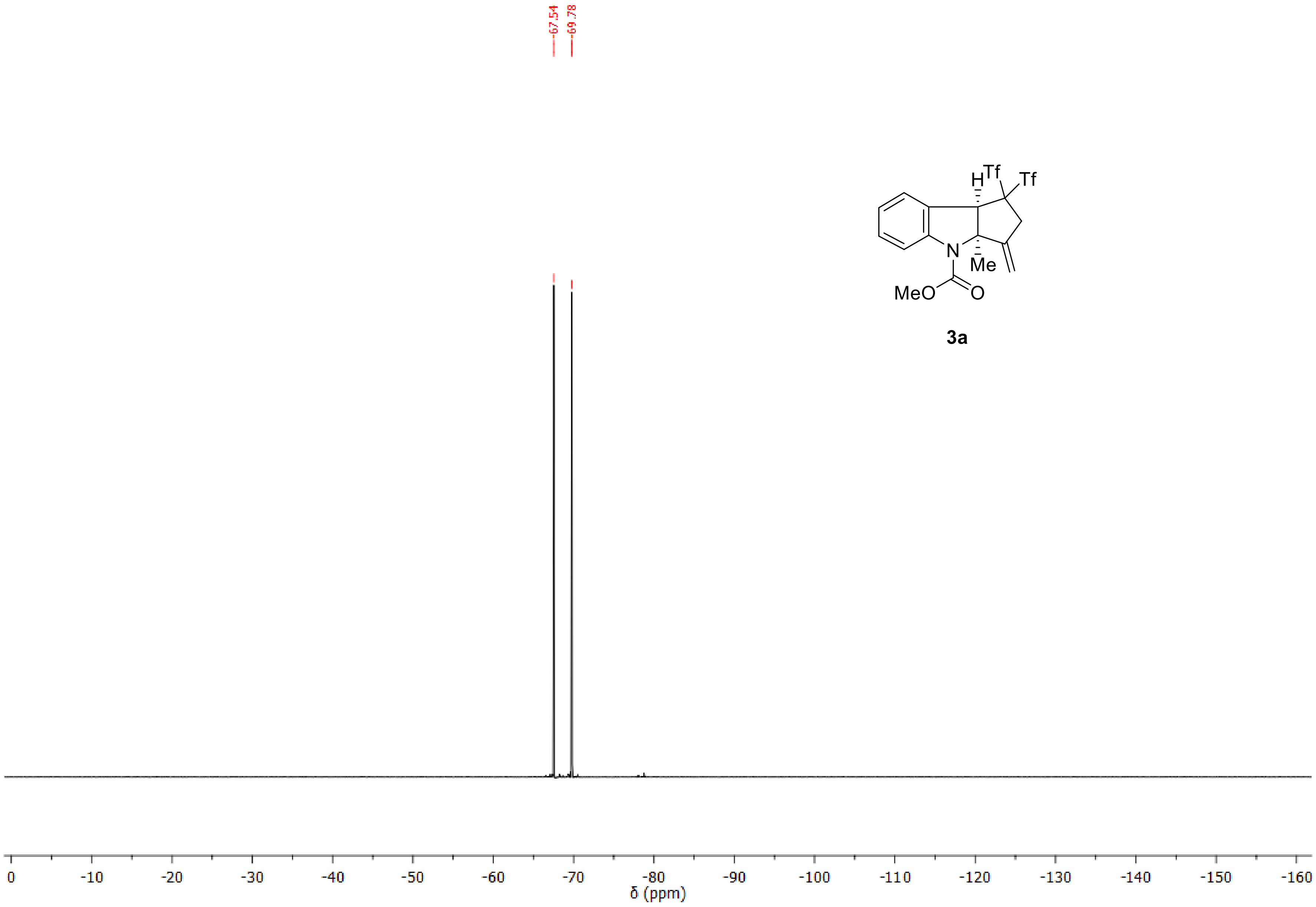

3a

2D - HH COSY - NMR ($CDCl_3$)

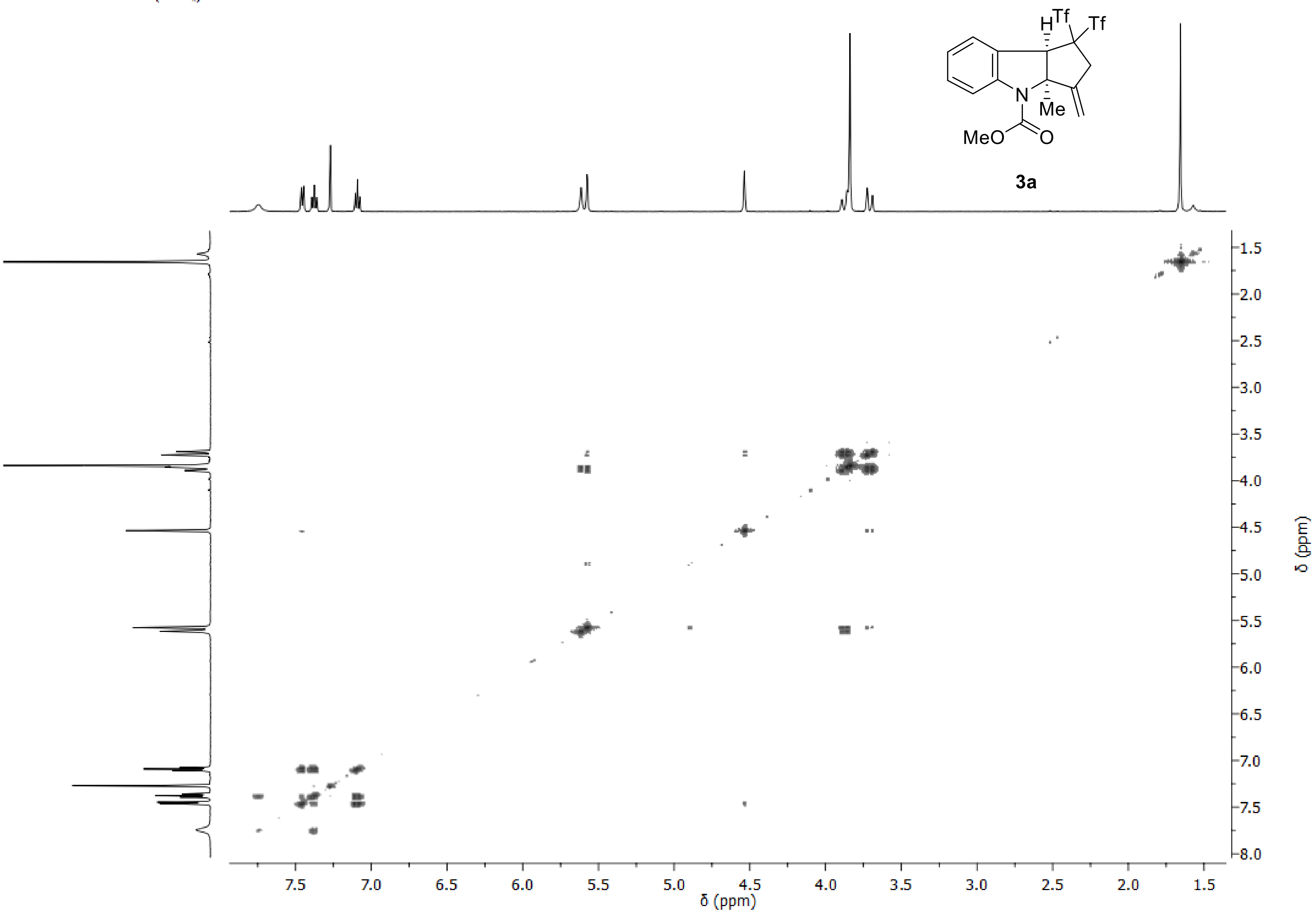

2D - HMQC - NMR ($CDCl_3$)

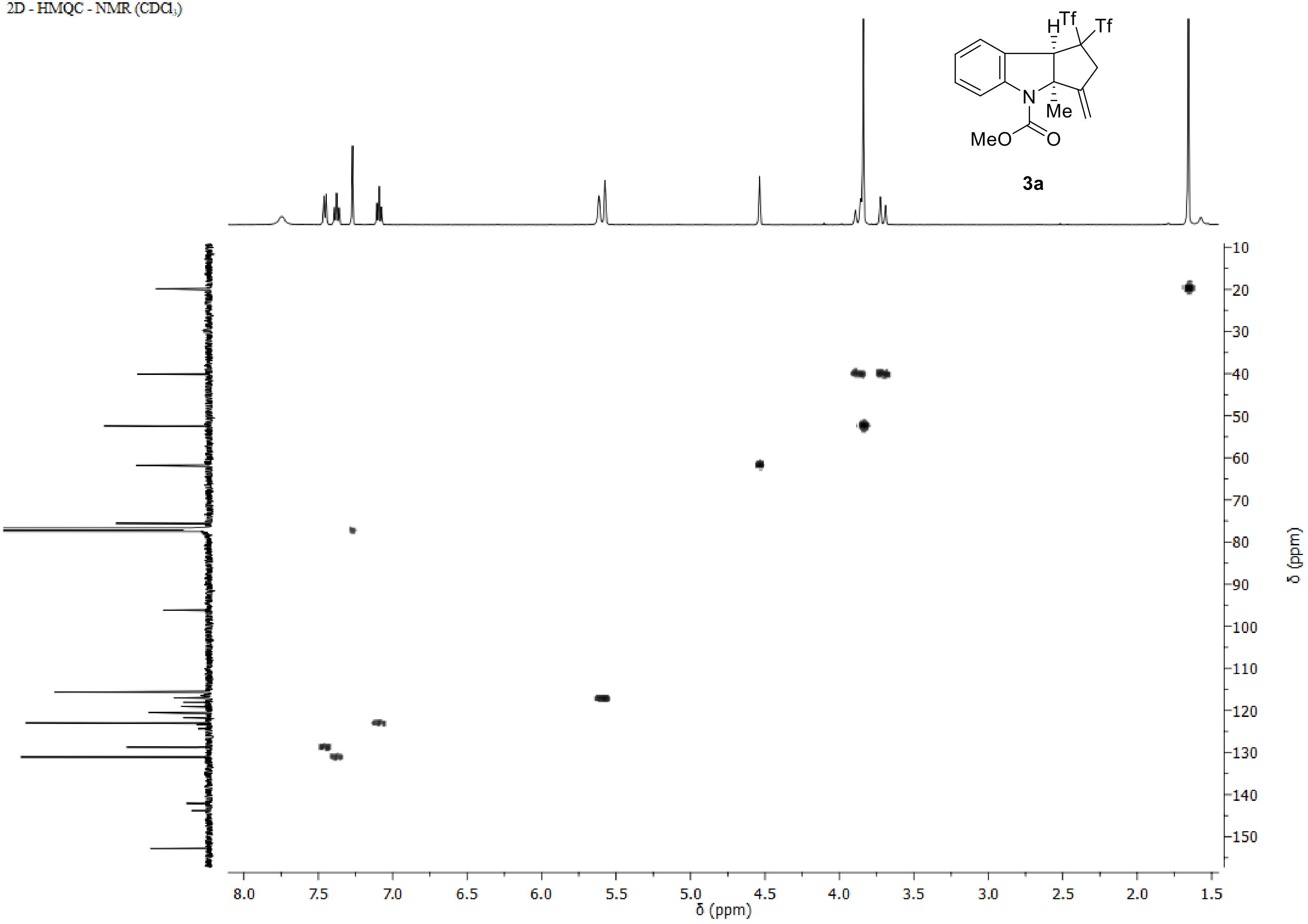

2D - HMBC - NMR ($CDCl_3$)

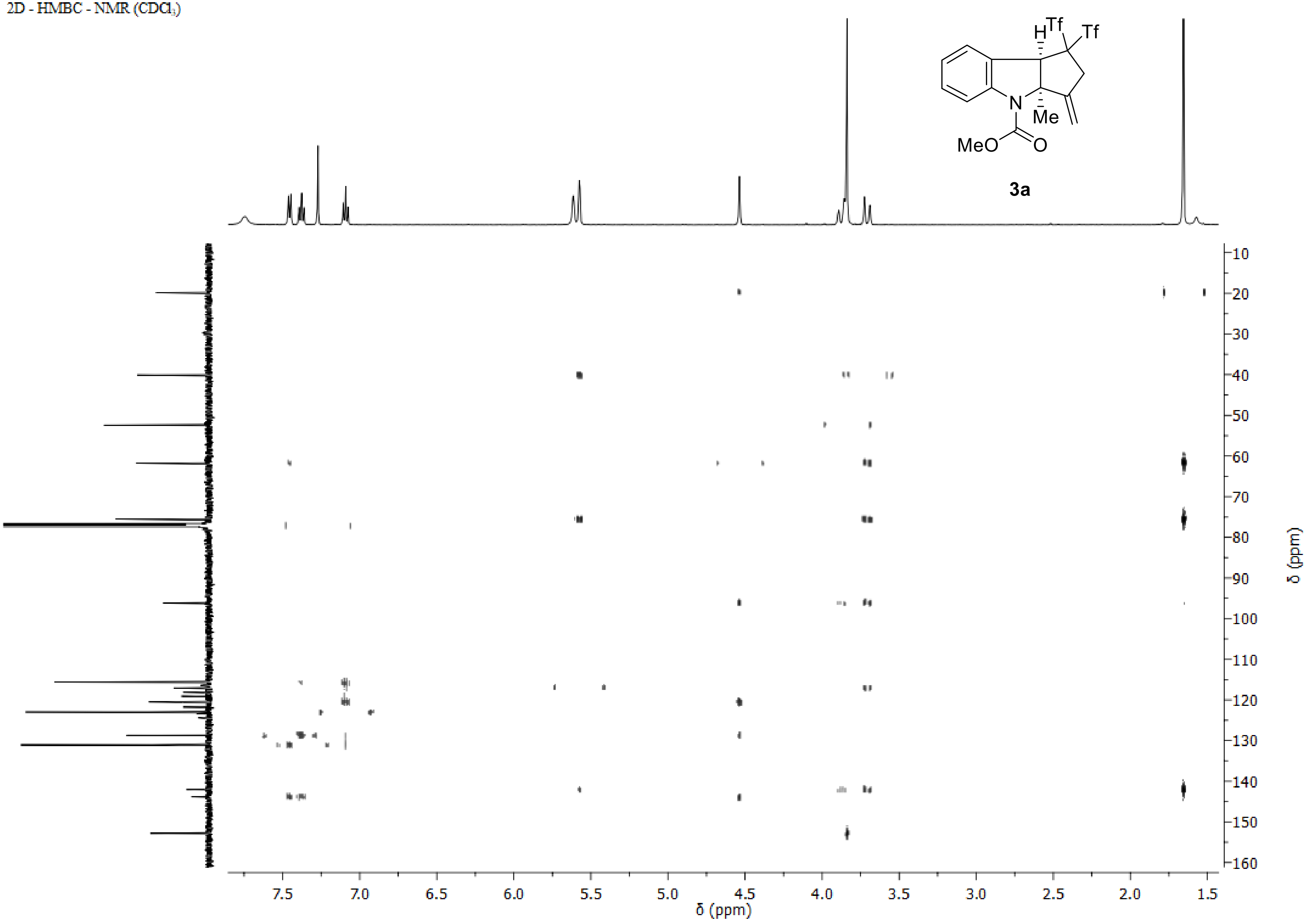

^{1}H NMR (500 MHz, $CDCl_3$)

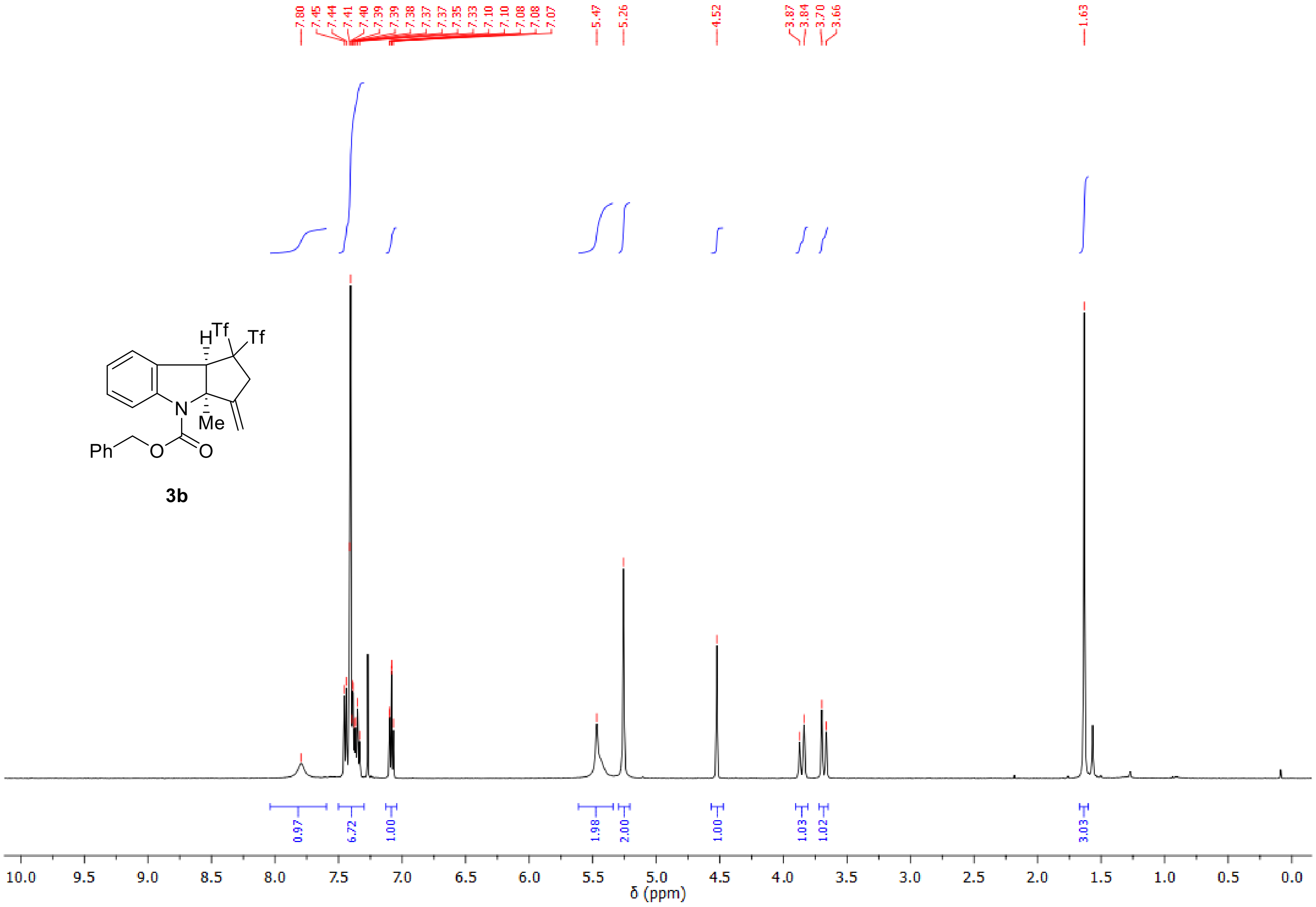

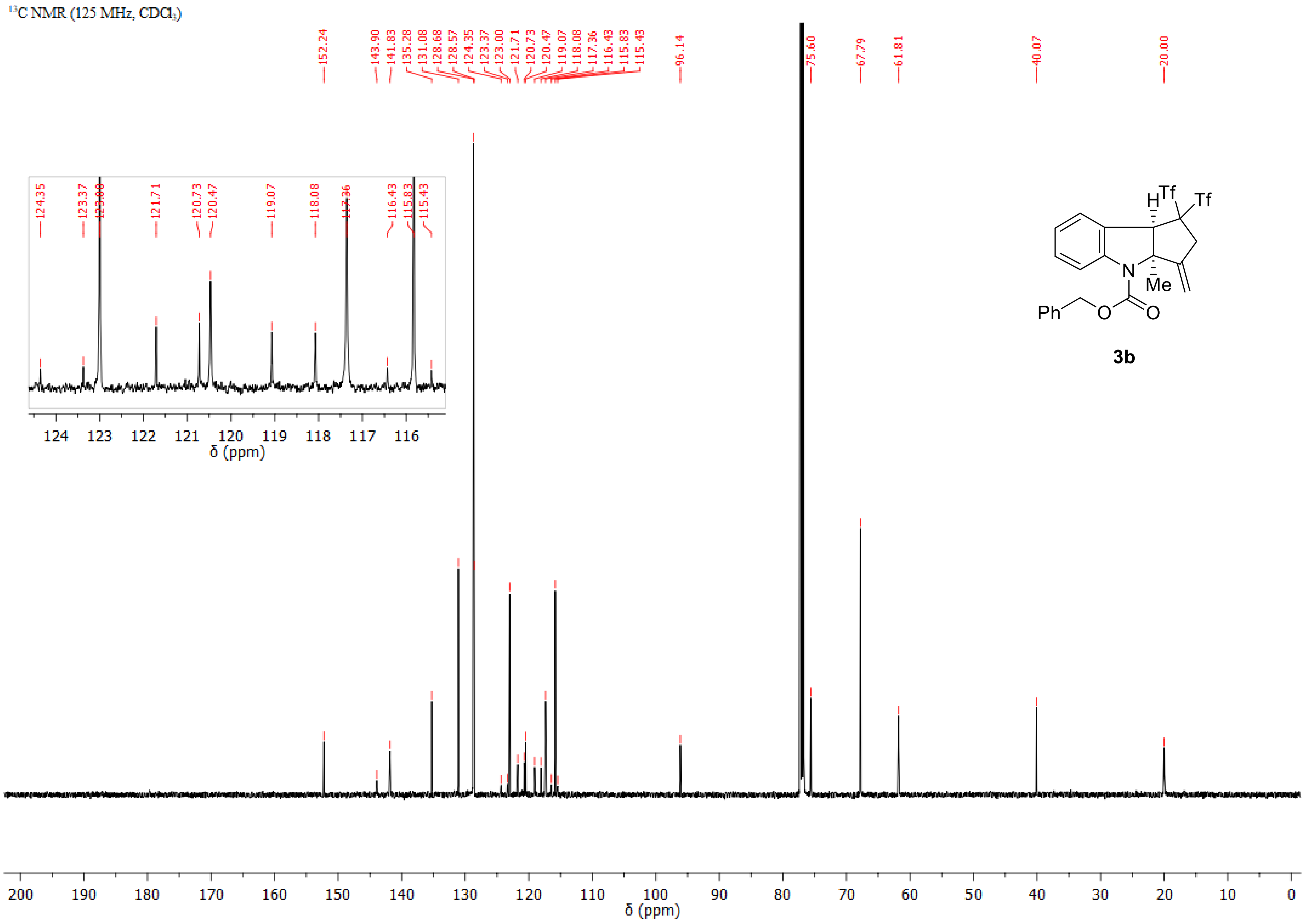
^{13}C NMR (125 MHz, CDCl$_3$)
152.24
143.90
141.83
135.28
131.08
128.68
128.57
124.35
123.37
123.00
121.71
120.73
120.47
119.07
118.08
117.36
116.43
115.83
115.43
96.14
75.60
67.79
61.81
40.07
20.00
δ (ppm)
H
Tf
Tf
N
Me
Ph
O
O

3b

^{19}F NMR (282 MHz, $CDCl_3$)

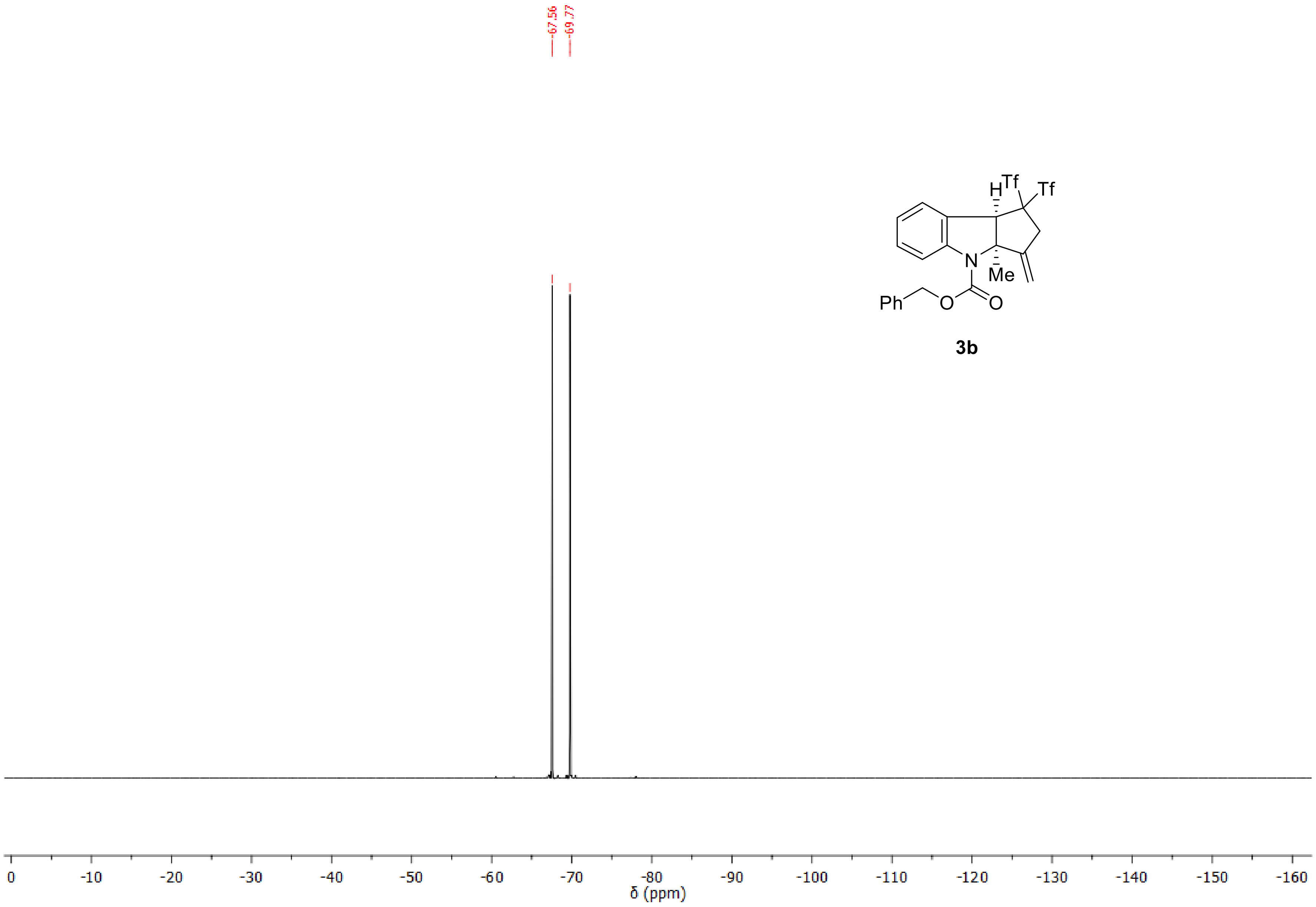

^{1}H NMR (500 MHz, CDCl$_3$)

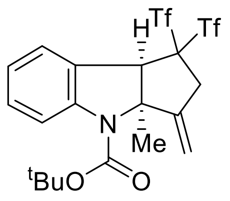

3c

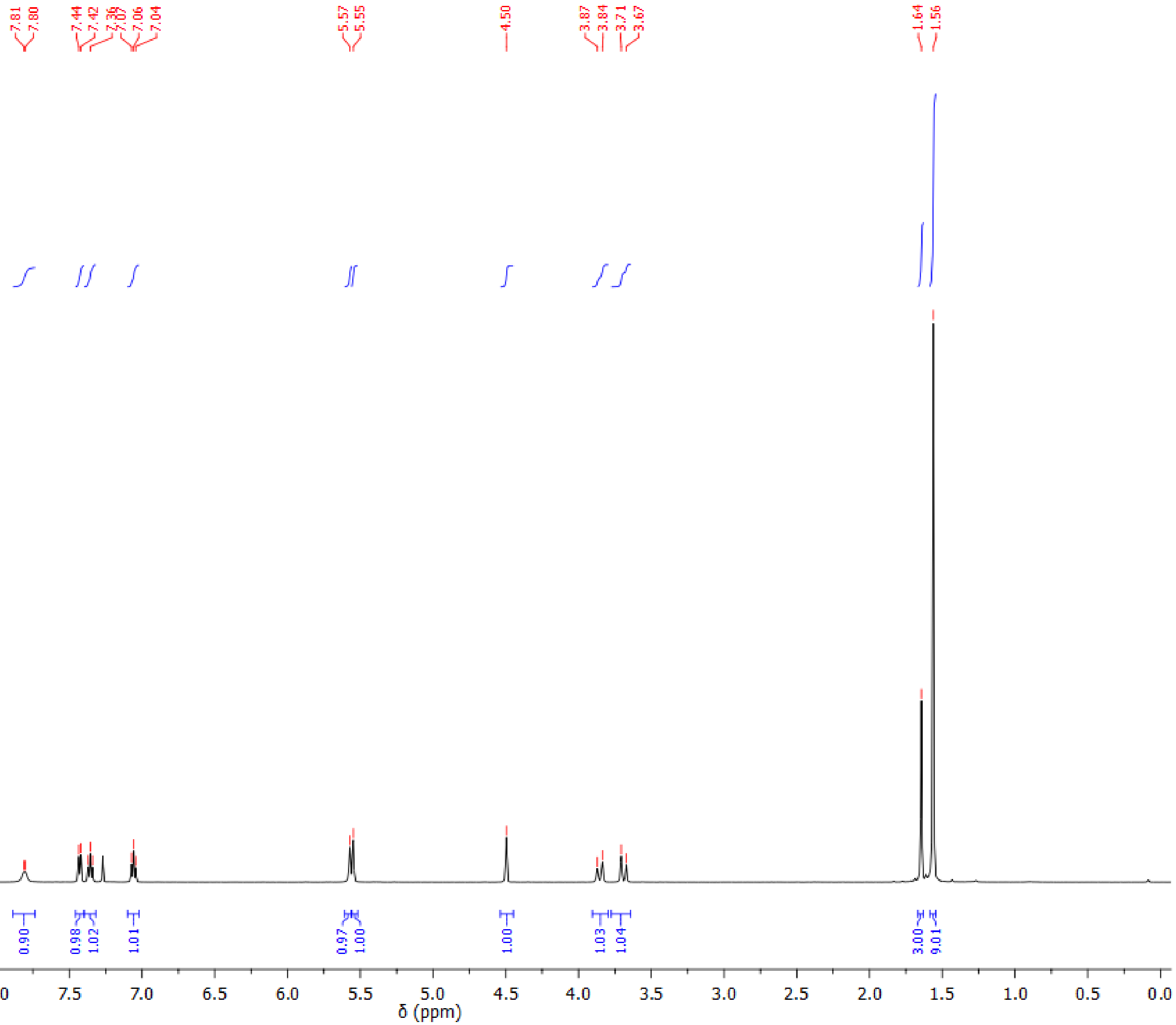

^{13}C NMR (125 MHz, $CDCl_3$)

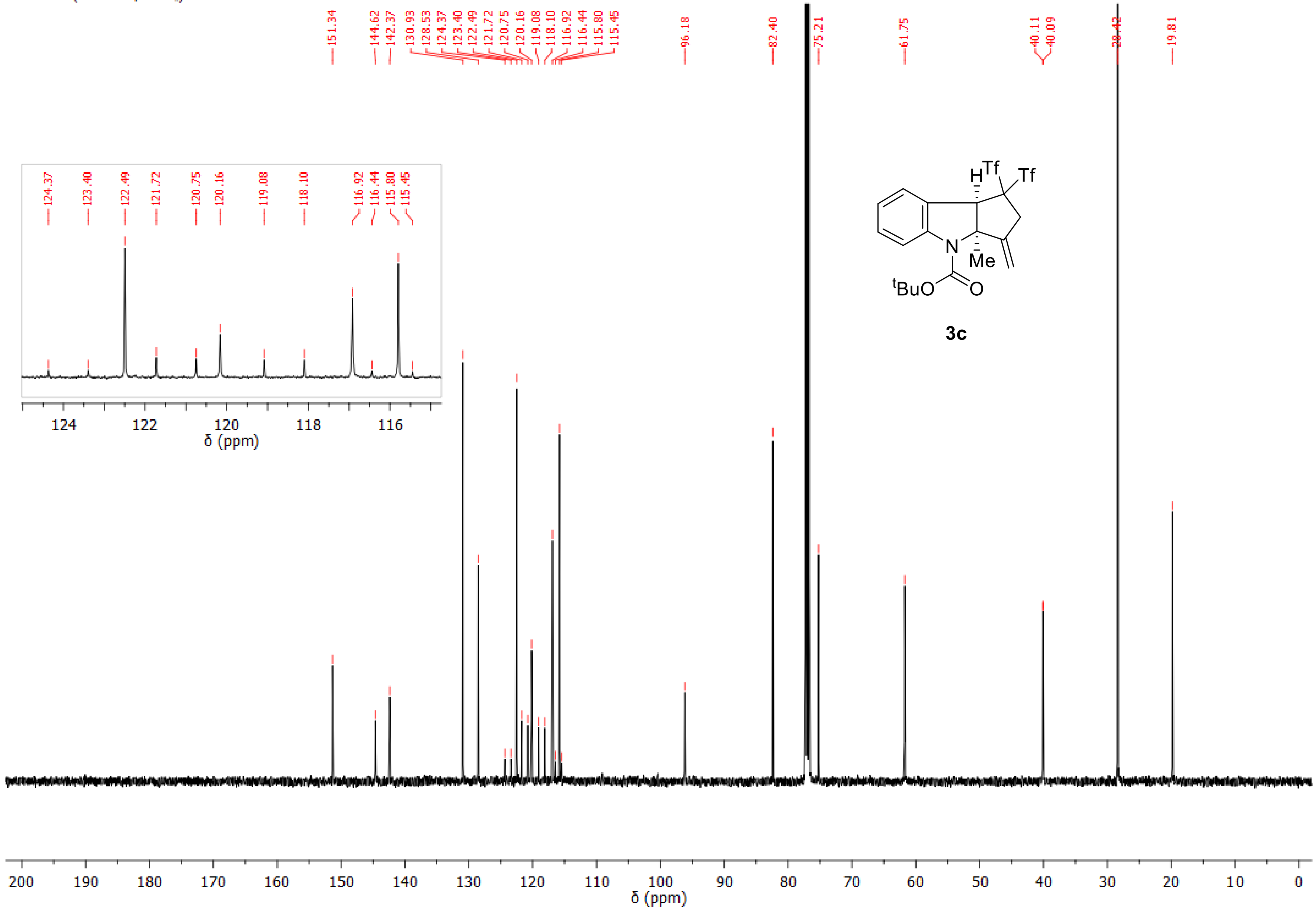

^{19}F NMR (282 MHz, $CDCl_3$)

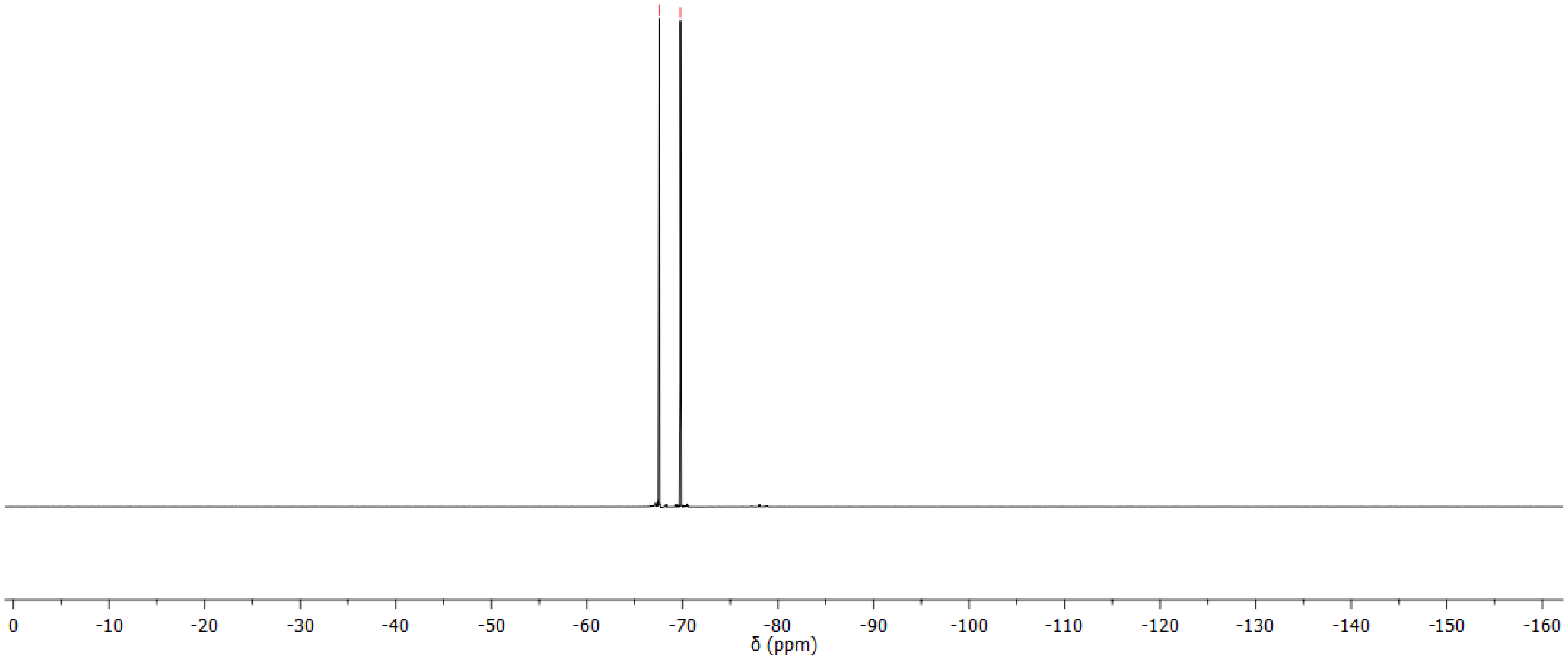

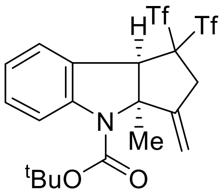

3c

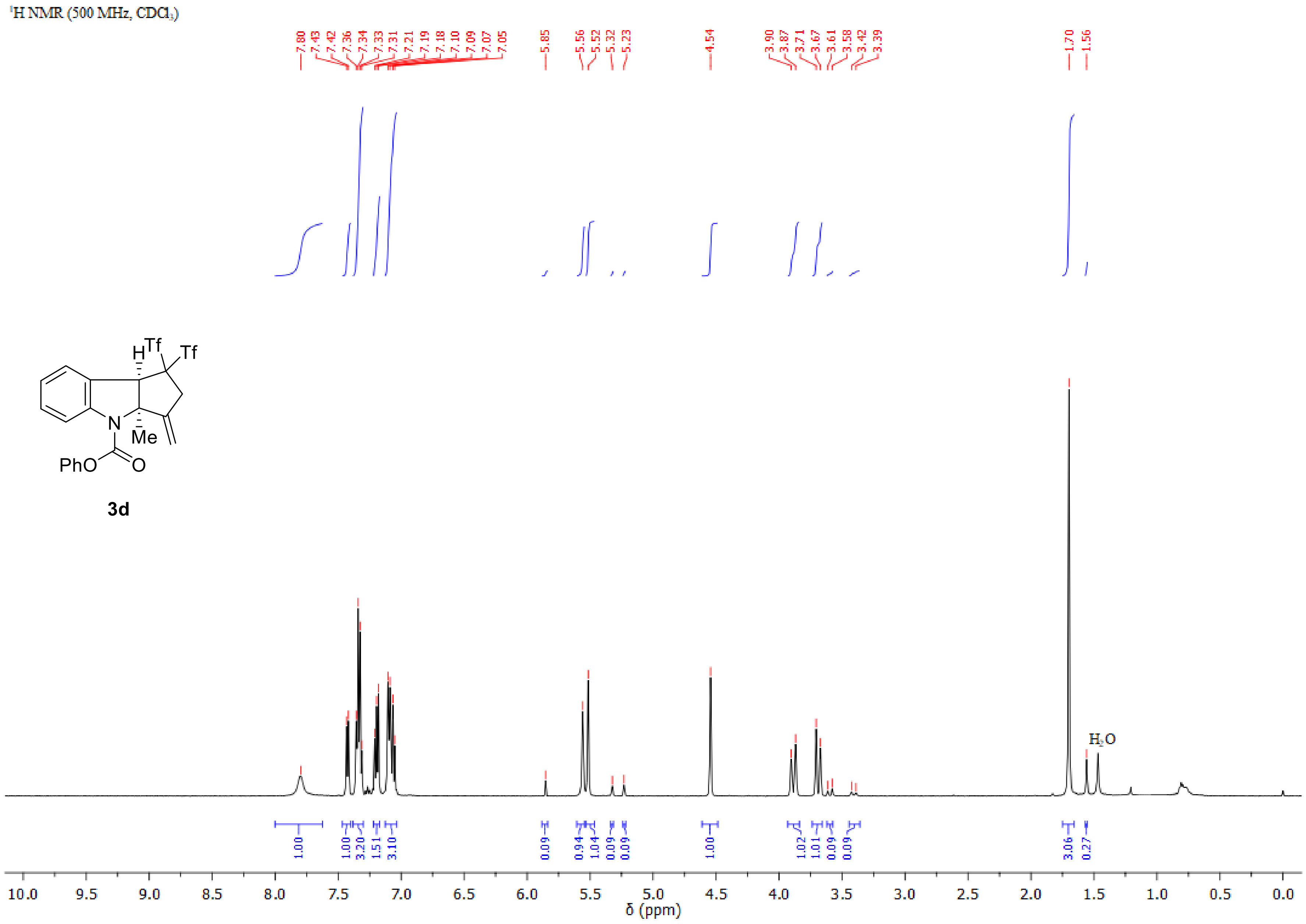

¹H NMR (500 MHz, CDCl₃)
7.80
7.43
7.42
7.36
7.34
7.33
7.31
7.21
7.19
7.18
7.10
7.09
7.07
7.05
5.85
5.56
5.52
5.32
5.23
4.54
3.90
3.87
3.71
3.67
3.61
3.58
3.42
3.39
1.70
1.56
H₂O
1.00
1.00
3.20
1.51
3.10
0.09
0.94
1.04
0.09
0.09
1.00
1.02
1.01
0.09
0.09
3.06
0.27
10.0
9.5
9.0
8.5
8.0
7.5
7.0
6.5
6.0
5.5
5.0
4.5
4.0
3.5
3.0
2.5
2.0
1.5
1.0
0.5
0.0
δ (ppm)
H
Tf
Tf
N
Me
PhO
O

3d

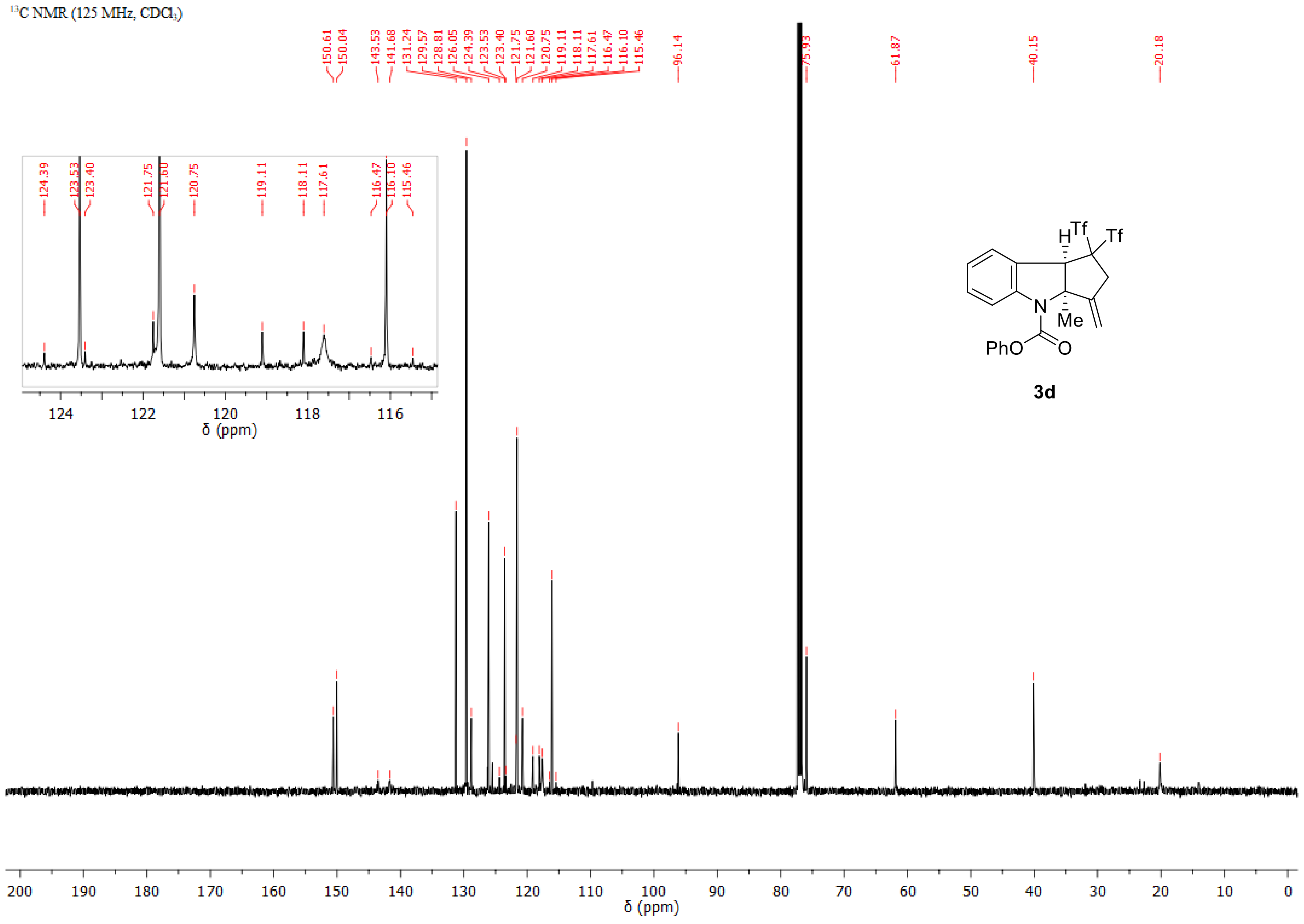

^{13}C NMR (125 MHz, CDCl$_3$)
150.61
150.04
143.53
141.68
131.24
129.57
128.81
126.05
124.39
123.53
123.40
121.75
121.60
120.75
119.11
118.11
117.61
116.47
116.10
115.46
96.14
75.93
61.87
40.15
20.18
δ (ppm)
H
Tf
Tf
N
Me
PhO
O
3d

^{19}F NMR (282 MHz, CDCl3)

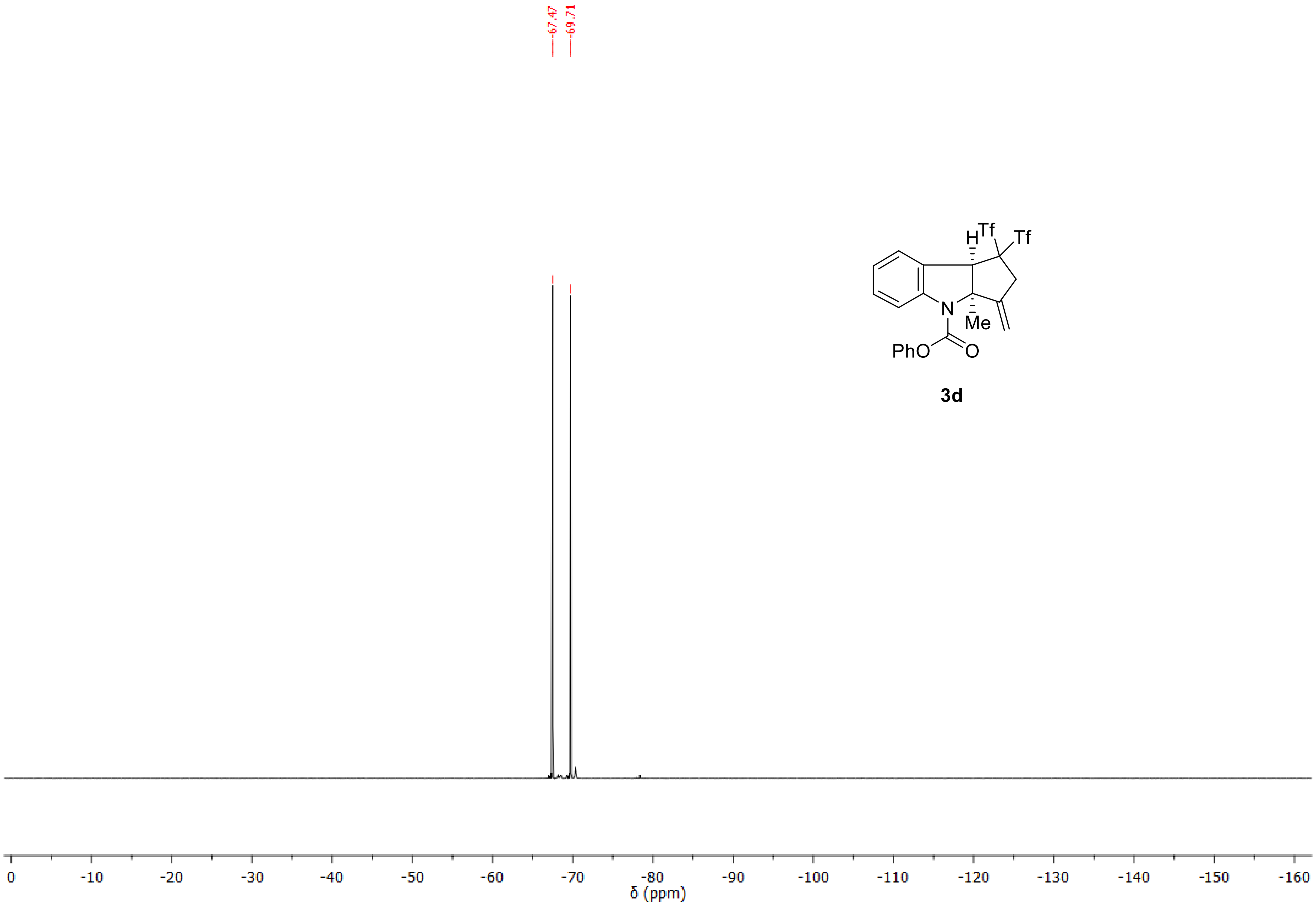

3d

^{1}H NMR (500 MHz, 25°C, 1,1,2,2-Tetrachloroethane-d_2)

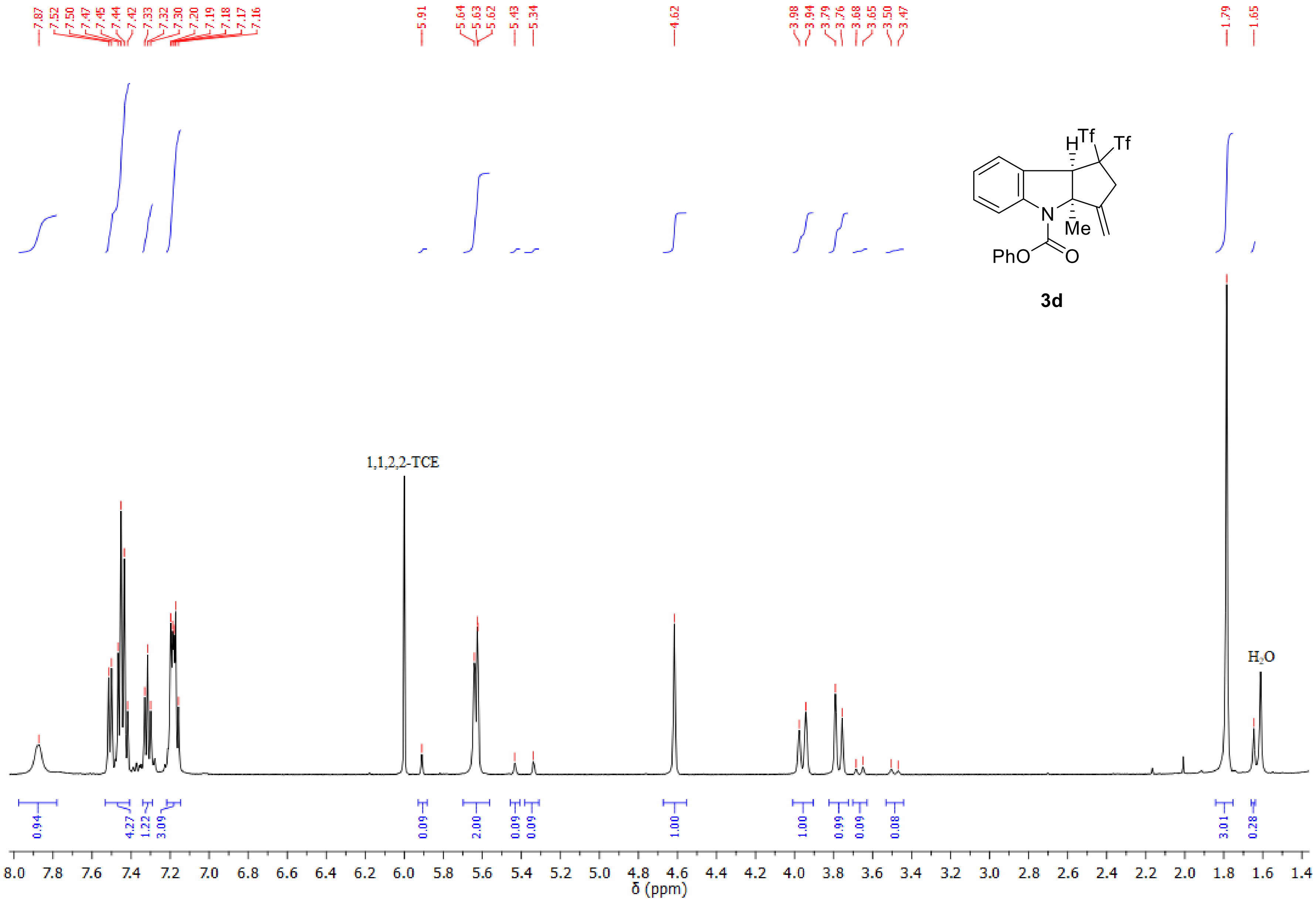

nOe irradiations of the protons of the methyl group and the methine moiety of both stereocenters at the minor set of signals in compound **3d** gave enhancements compatible with a *syn*-stereochemistry. Besides, in addition of the correlations with protons of the minor set of signals, enhancements on the signals of the protons of the major set of signals were observed, which points to the rotameric nature of both set of signals.

NOE Experiment (500 MHz, 1,1,2,2-Tetrachloroethane-d_2). Irradiation CH (minor compound)

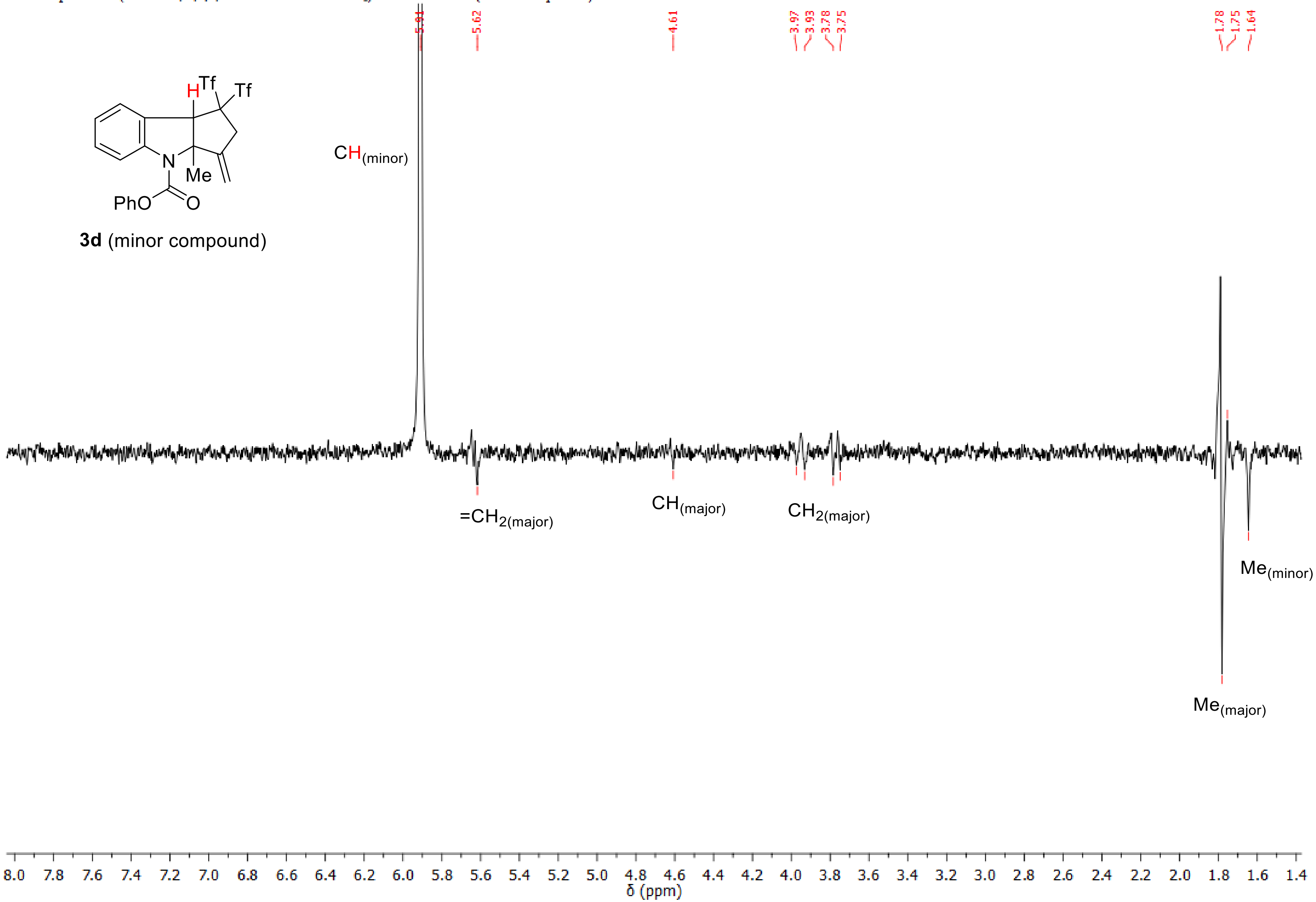

NOE Experiment (500 MHz, 1,1,2,2-Tetrachloroethane-d_2). Irradiation CH_3 (minor compound)

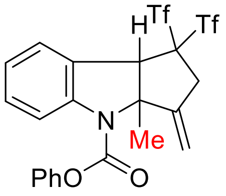

3d (minor compound)

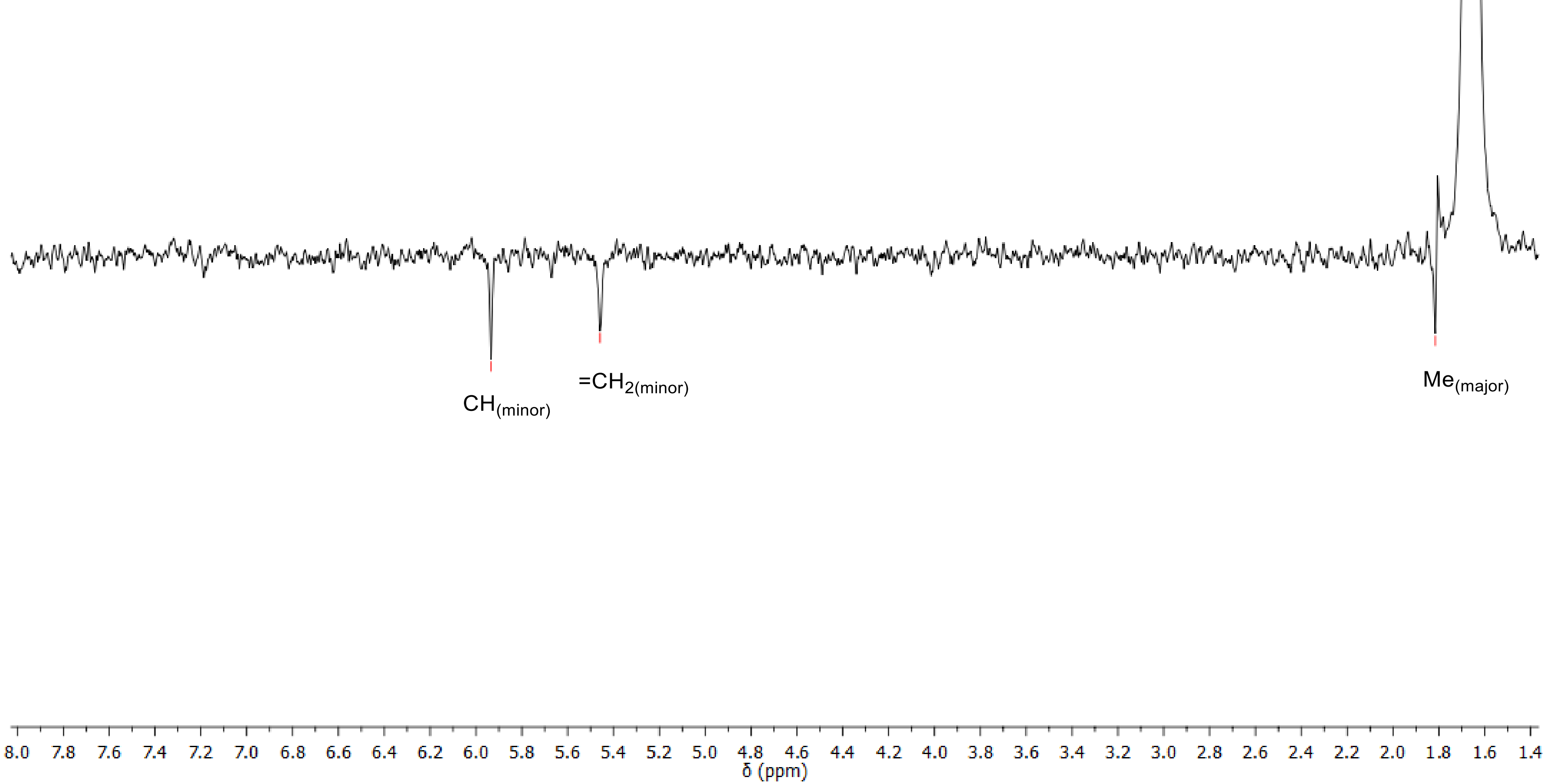

^{1}H NMR (500 MHz, $CDCl_3$)

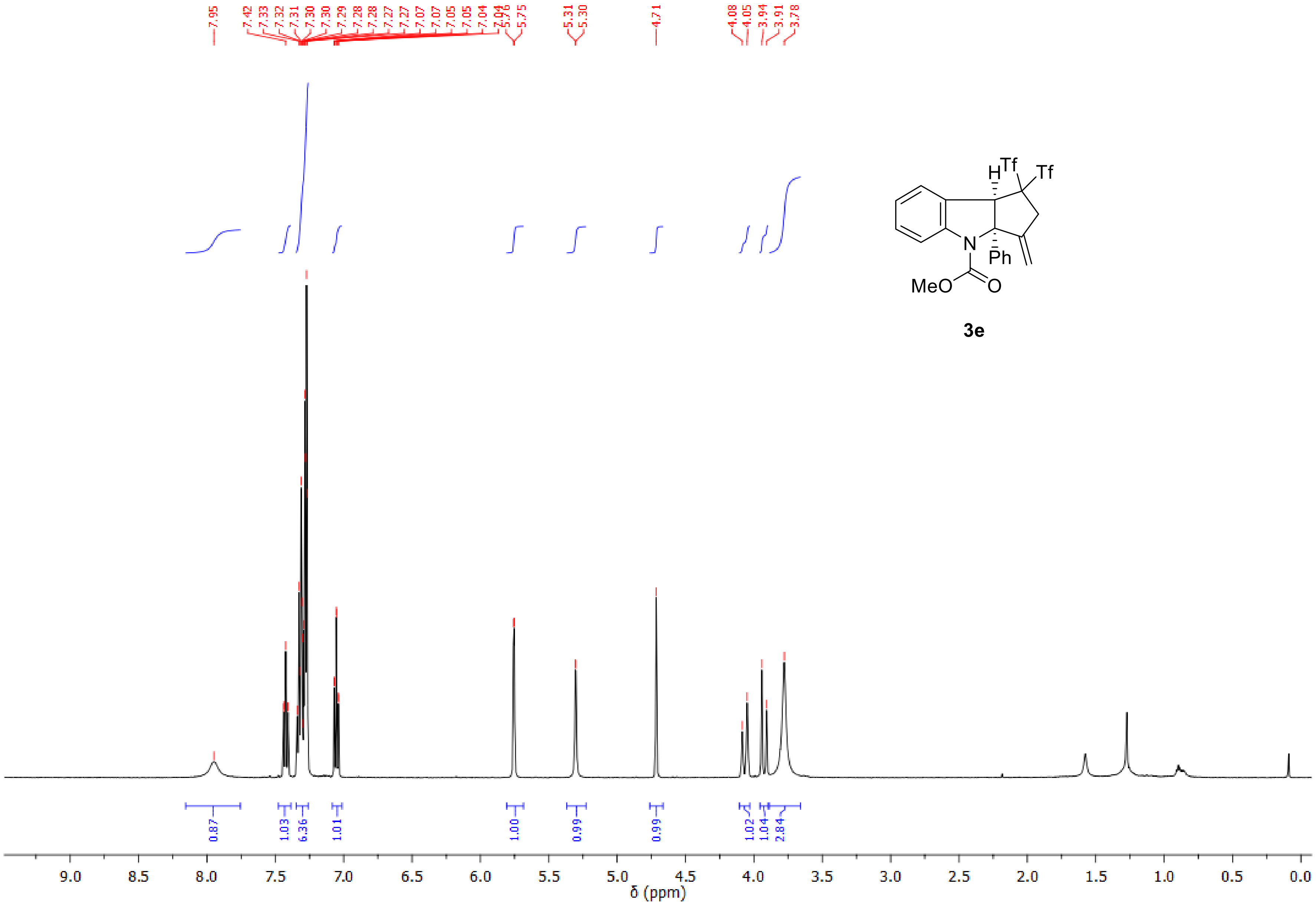

3e

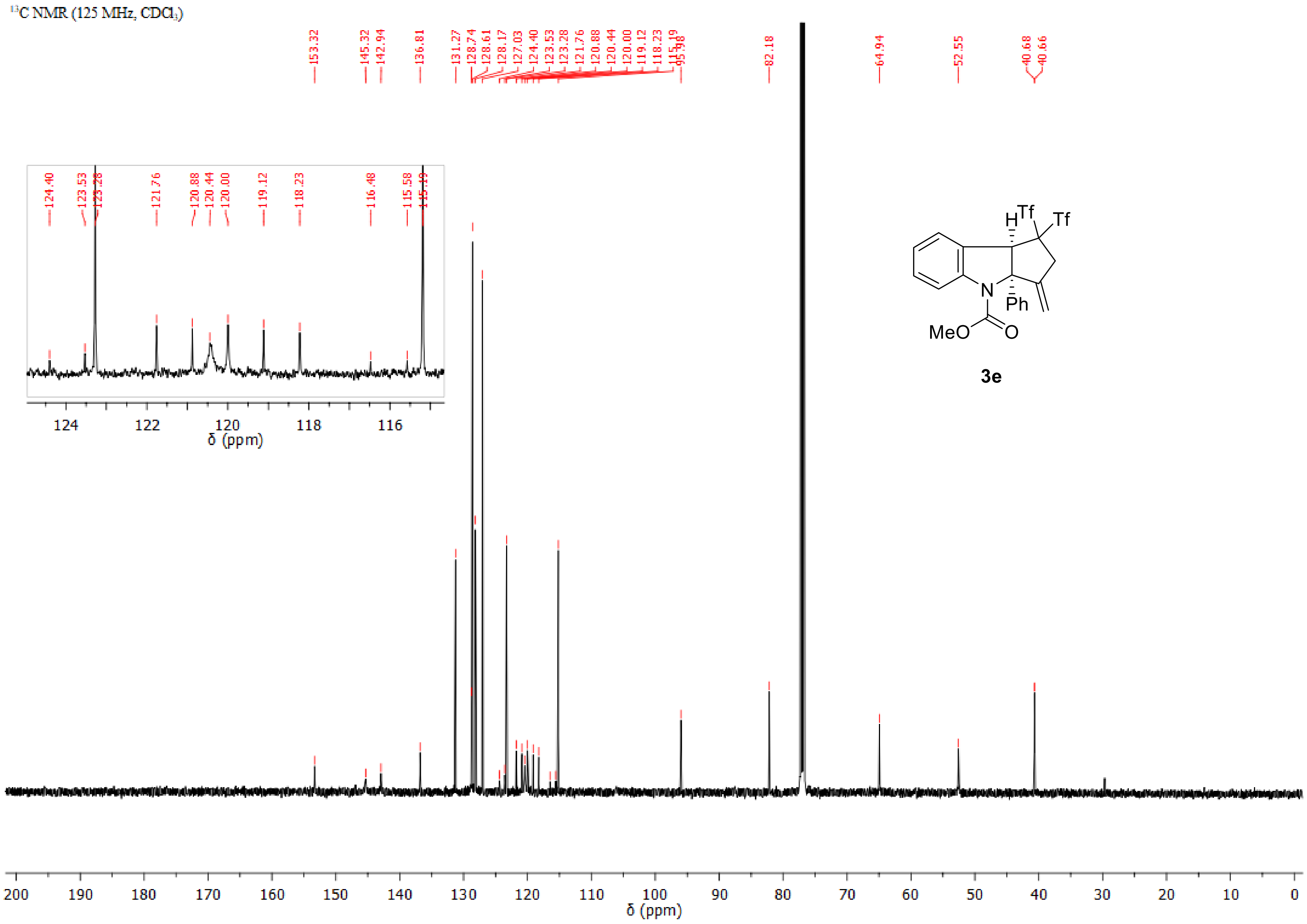
13C NMR (125 MHz, CDCl3)
153.32
145.32
142.94
136.81
131.27
128.74
128.61
128.17
127.03
124.40
123.53
123.28
121.76
120.88
120.44
120.00
119.12
118.23
115.19
95.98
82.18
64.94
52.55
40.68
40.66
116.48
115.58
δ (ppm)
H
Tf
Tf
N
Ph
MeO
O
3e

^{19}F NMR (282 MHz, $CDCl_3$)

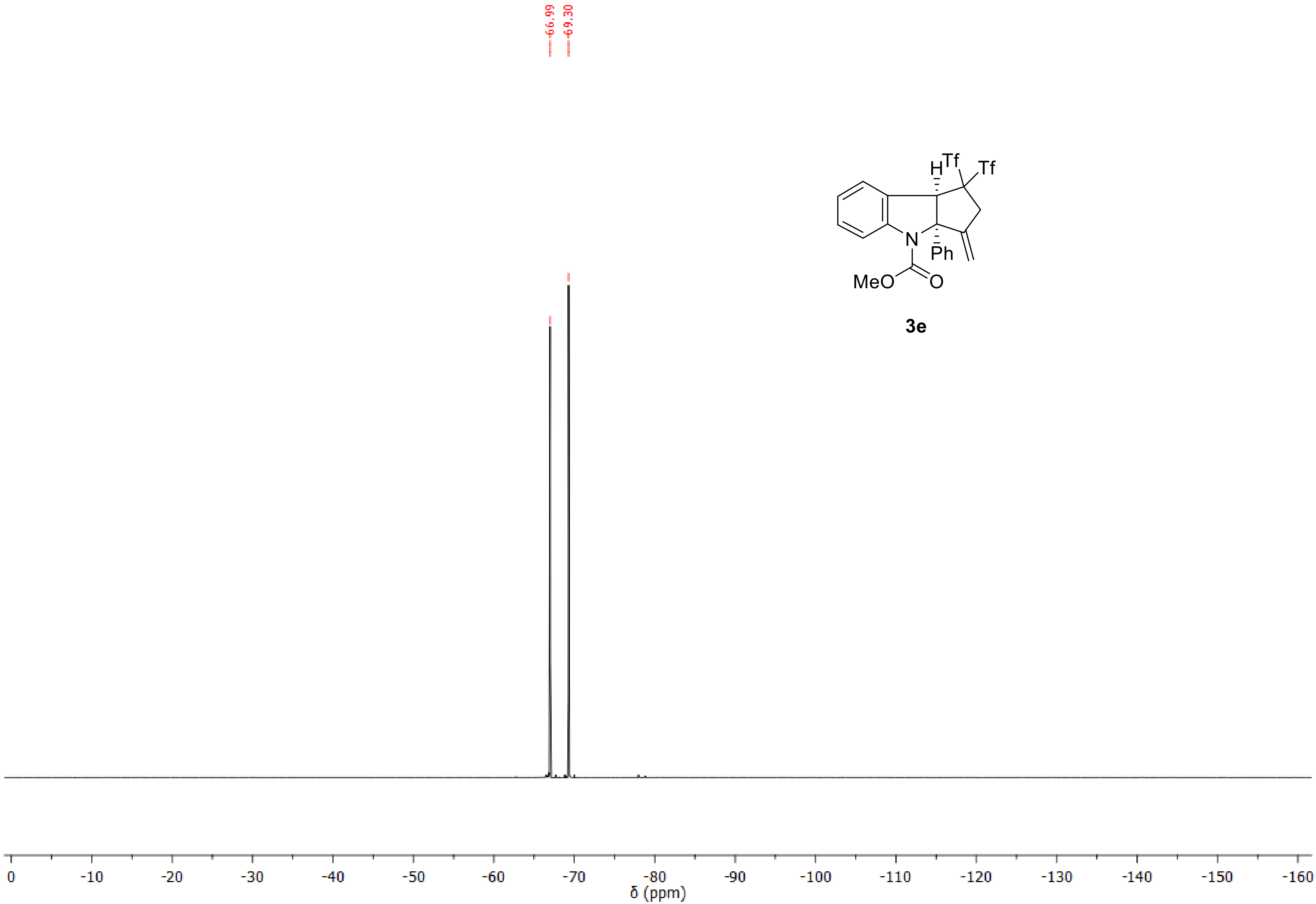

3e

NOE Experiment (500 MHz, $CDCl_3$). Irradiation CH

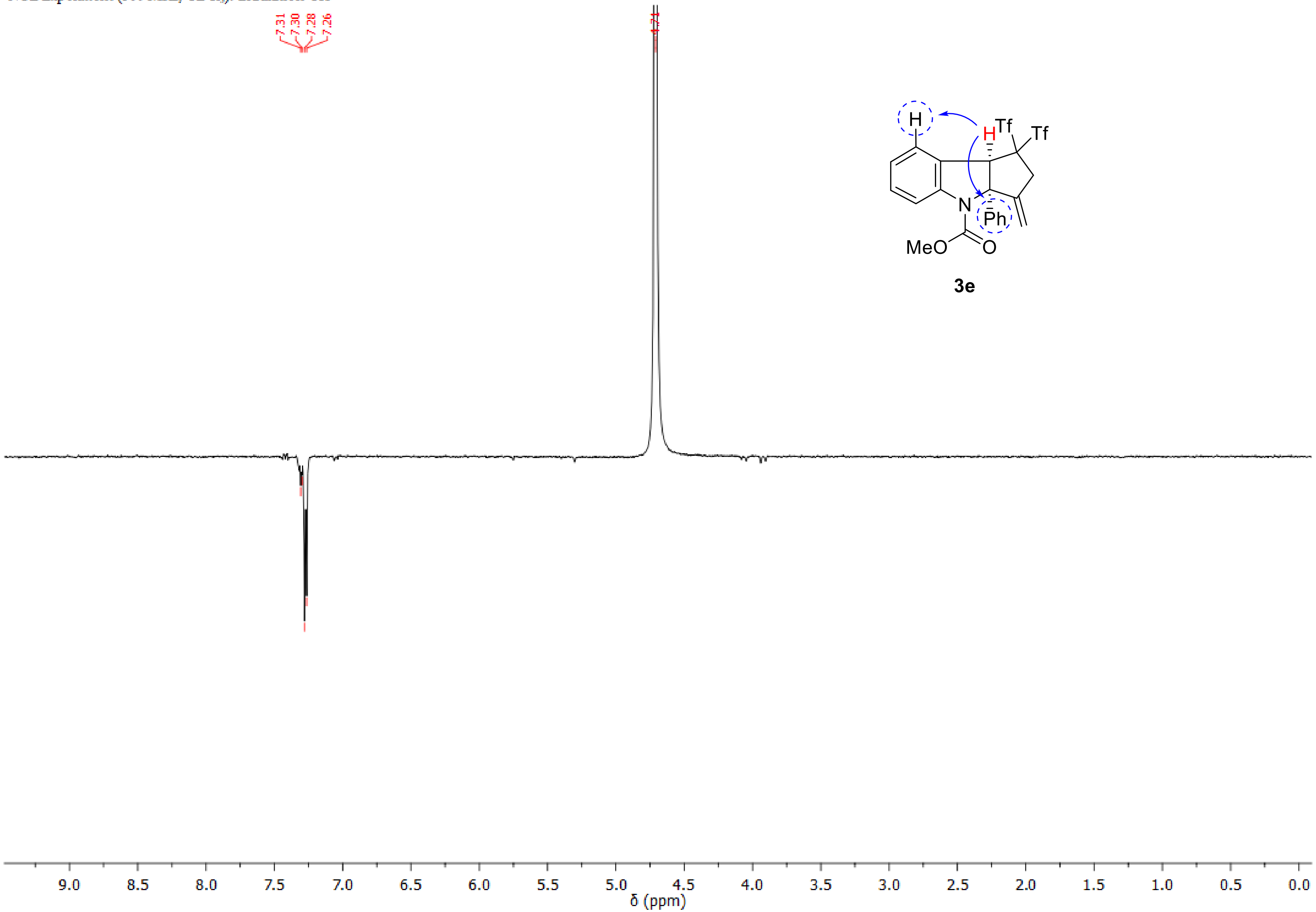

2D - HH COSY - NMR ($CDCl_3$)

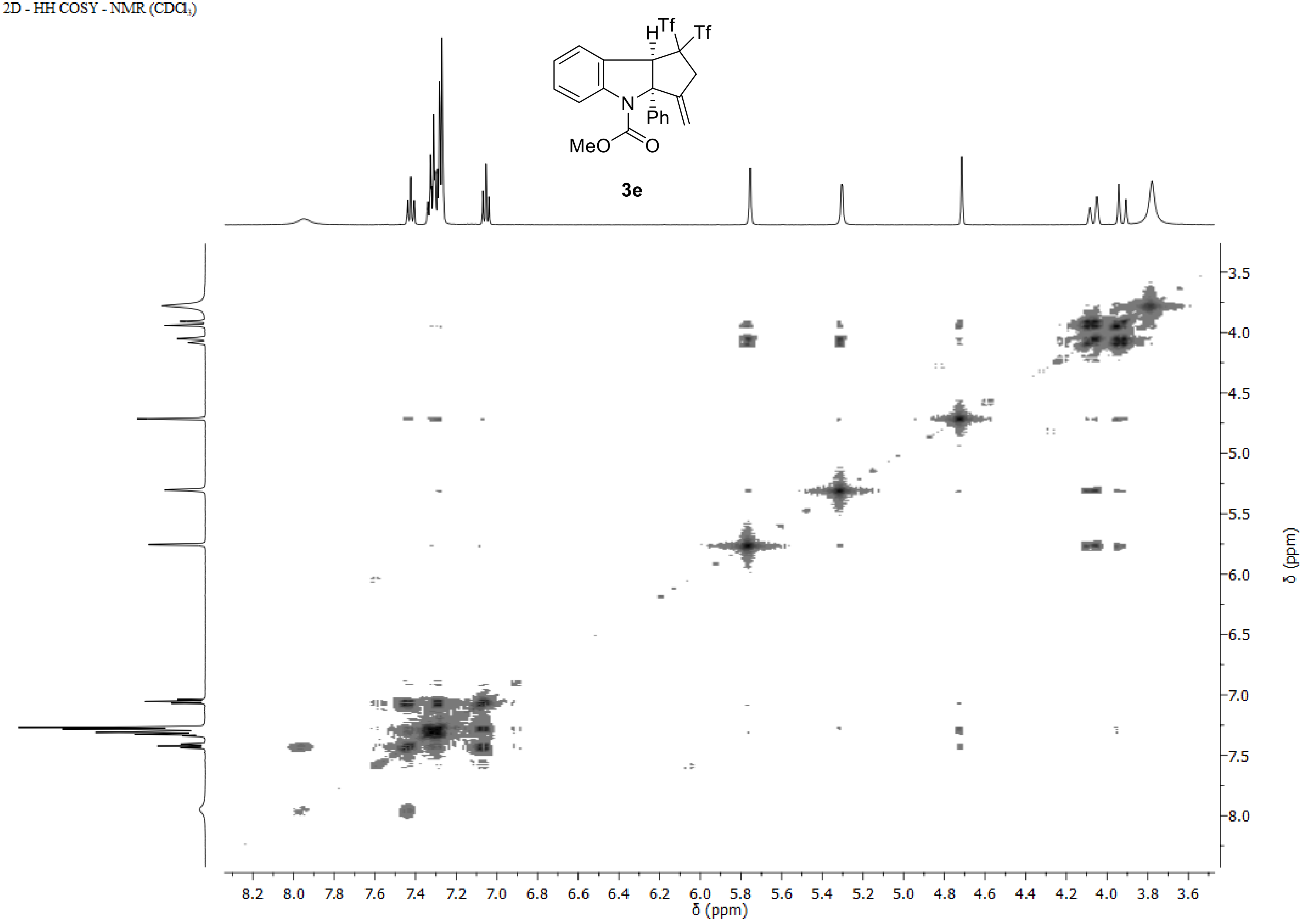

2D - HMQC - NMR ($CDCl_3$)

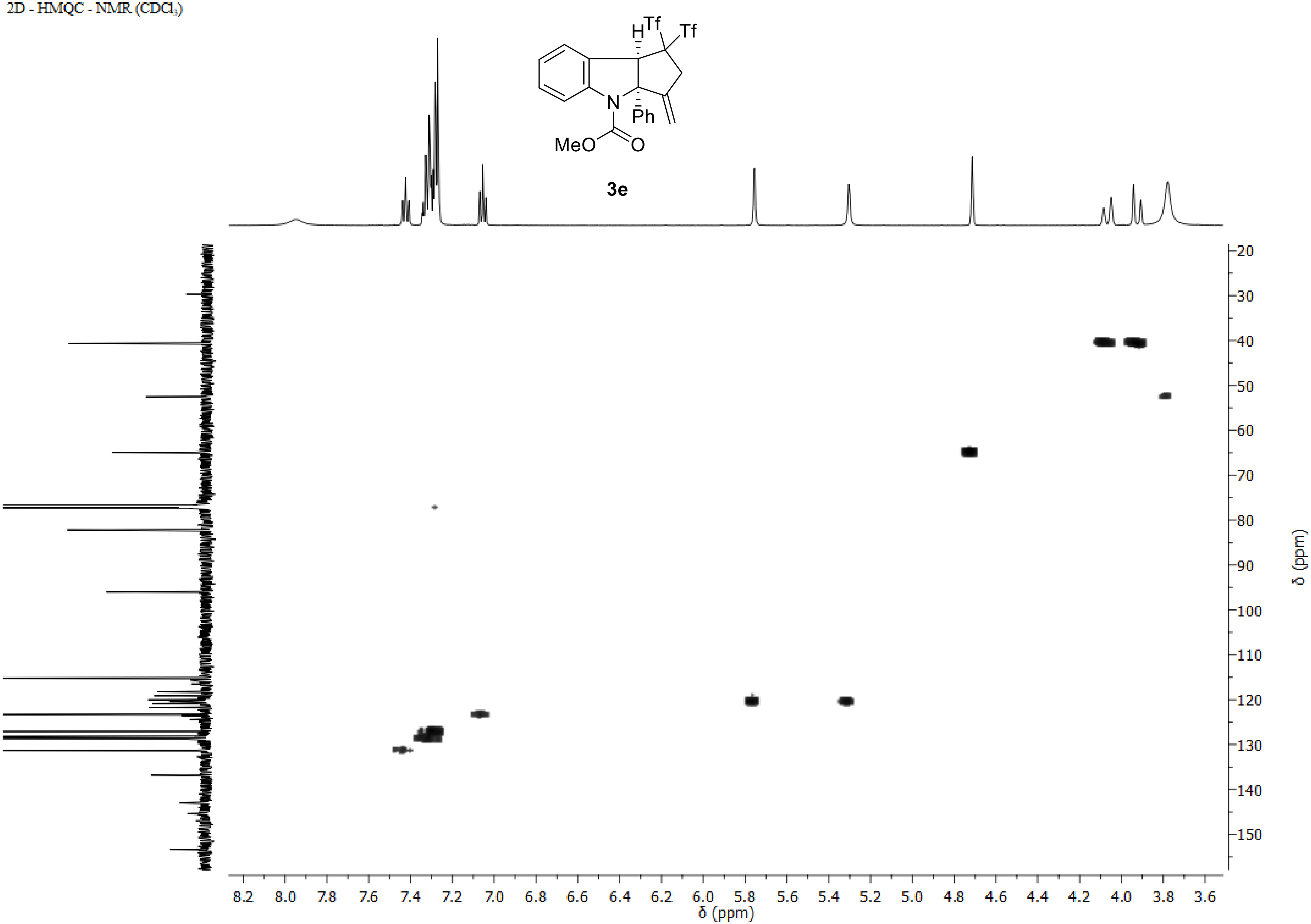

2D - HMBC - NMR ($CDCl_3$)

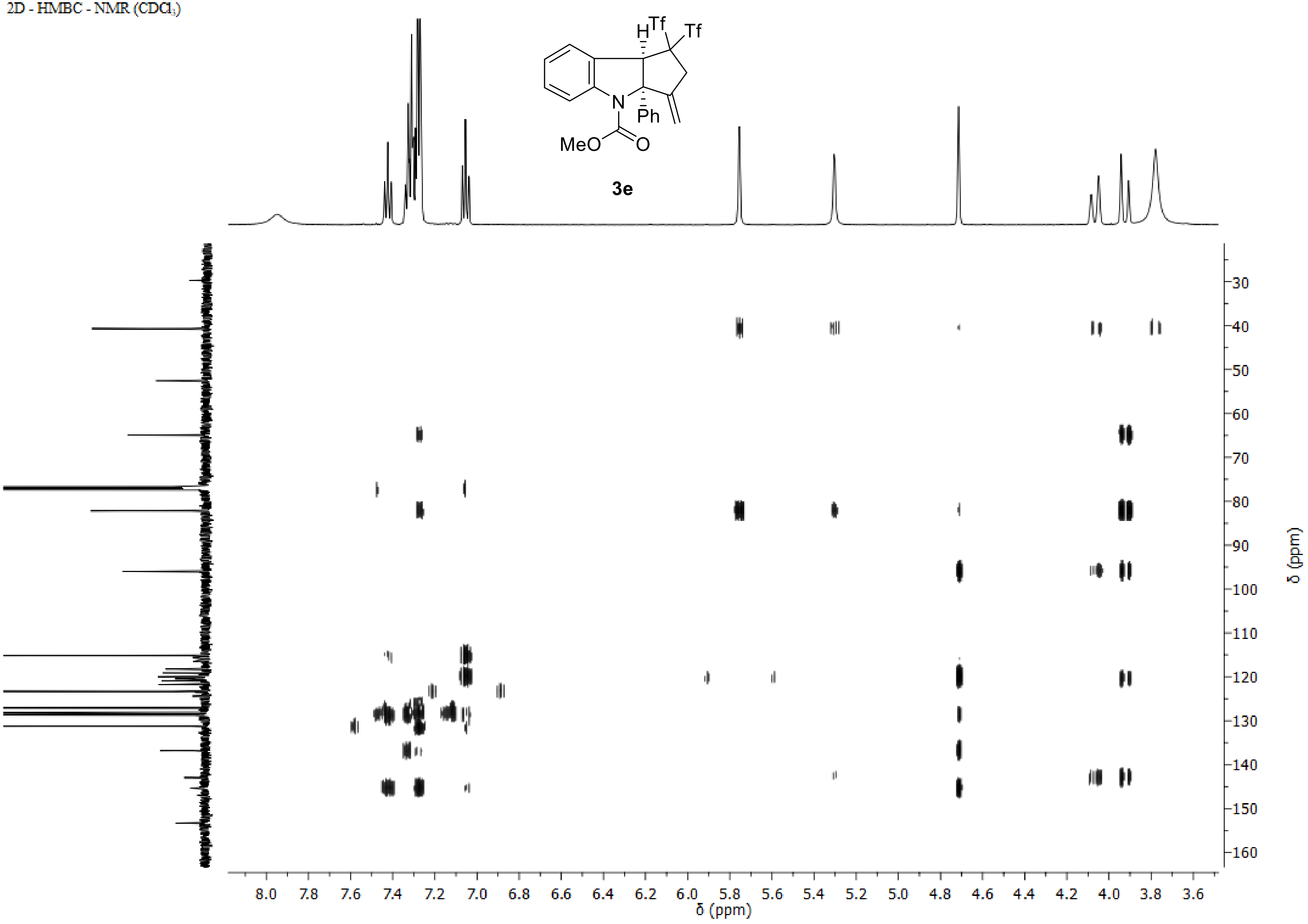

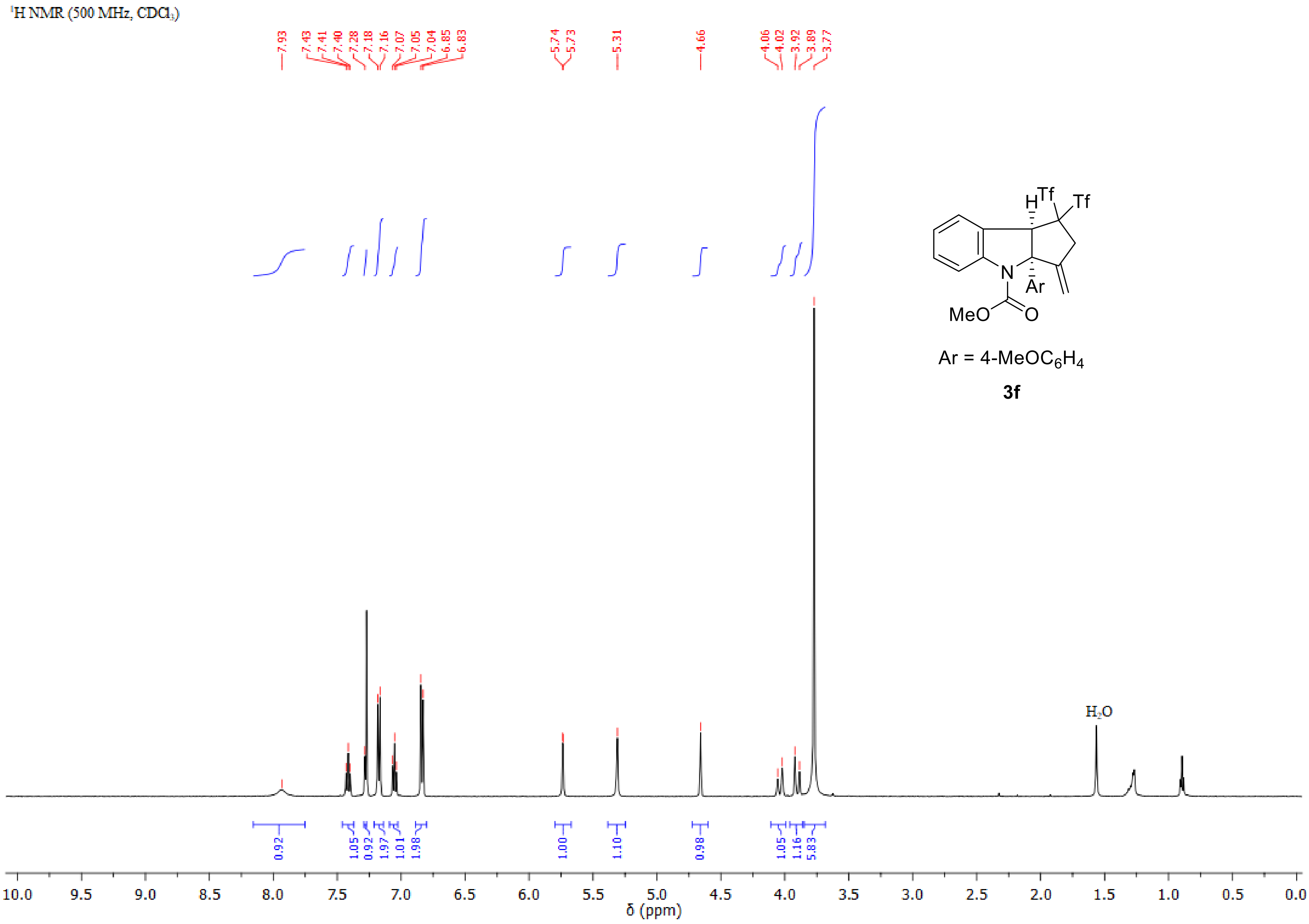

^{1}H NMR (500 MHz, CDCl$_3$)
7.93
7.43
7.41
7.40
7.28
7.18
7.16
7.07
7.05
7.04
6.85
6.83
5.74
5.73
5.31
4.66
4.06
4.02
3.92
3.89
3.77
H$_2$O
0.92
1.05
0.92
1.97
1.01
1.98
1.00
1.10
0.98
1.05
1.16
5.83
10.0 9.5 9.0 8.5 8.0 7.5 7.0 6.5 6.0 5.5 5.0 4.5 4.0 3.5 3.0 2.5 2.0 1.5 1.0 0.5 0.0
δ (ppm)
H
Tf
Tf
N
Ar
MeO
O
Ar = 4-MeOC$_6$H$_4$

3f

^{13}C NMR (125 MHz, $CDCl_3$)

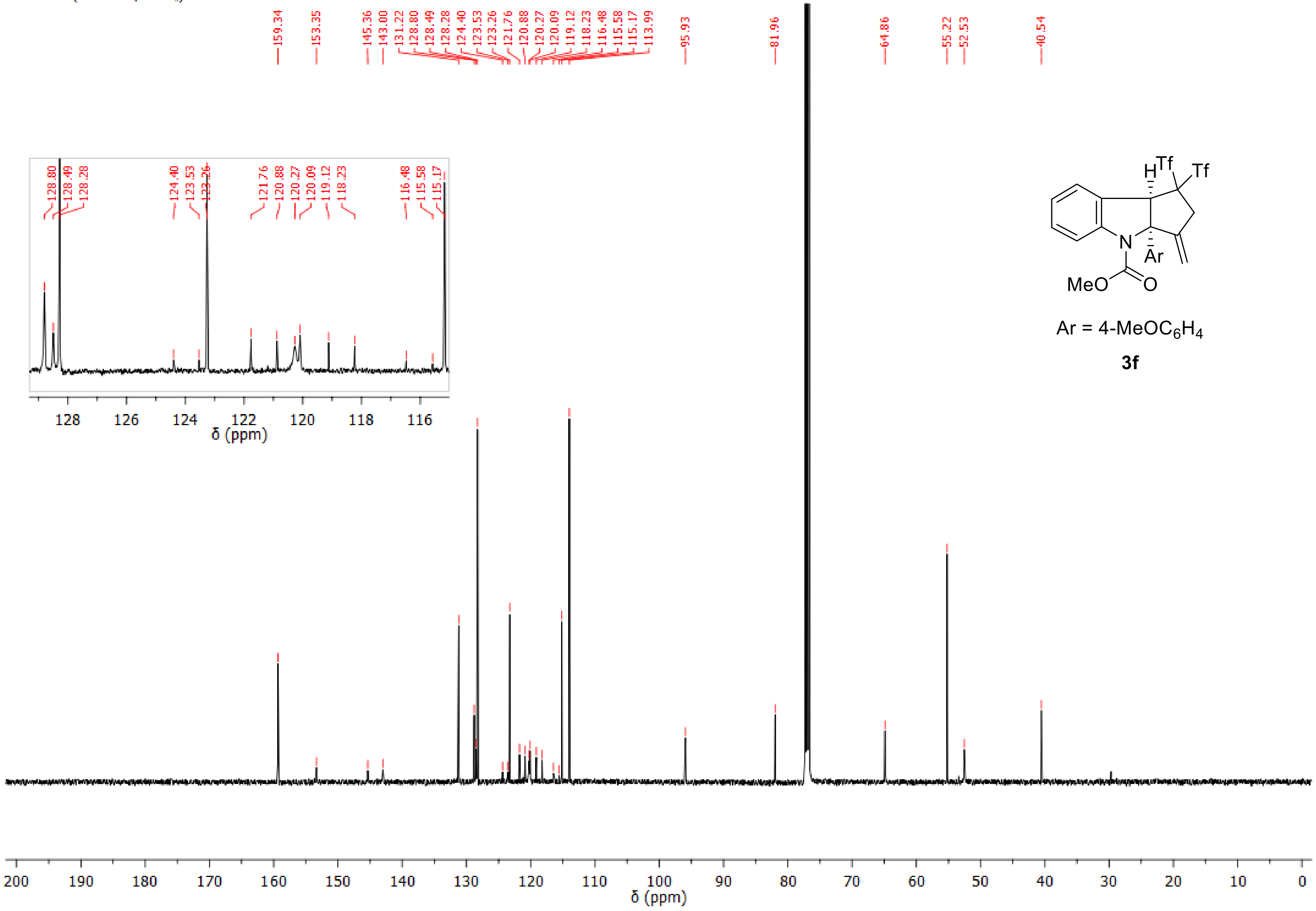

Ar = 4-MeOC$_6$H$_4$

3f

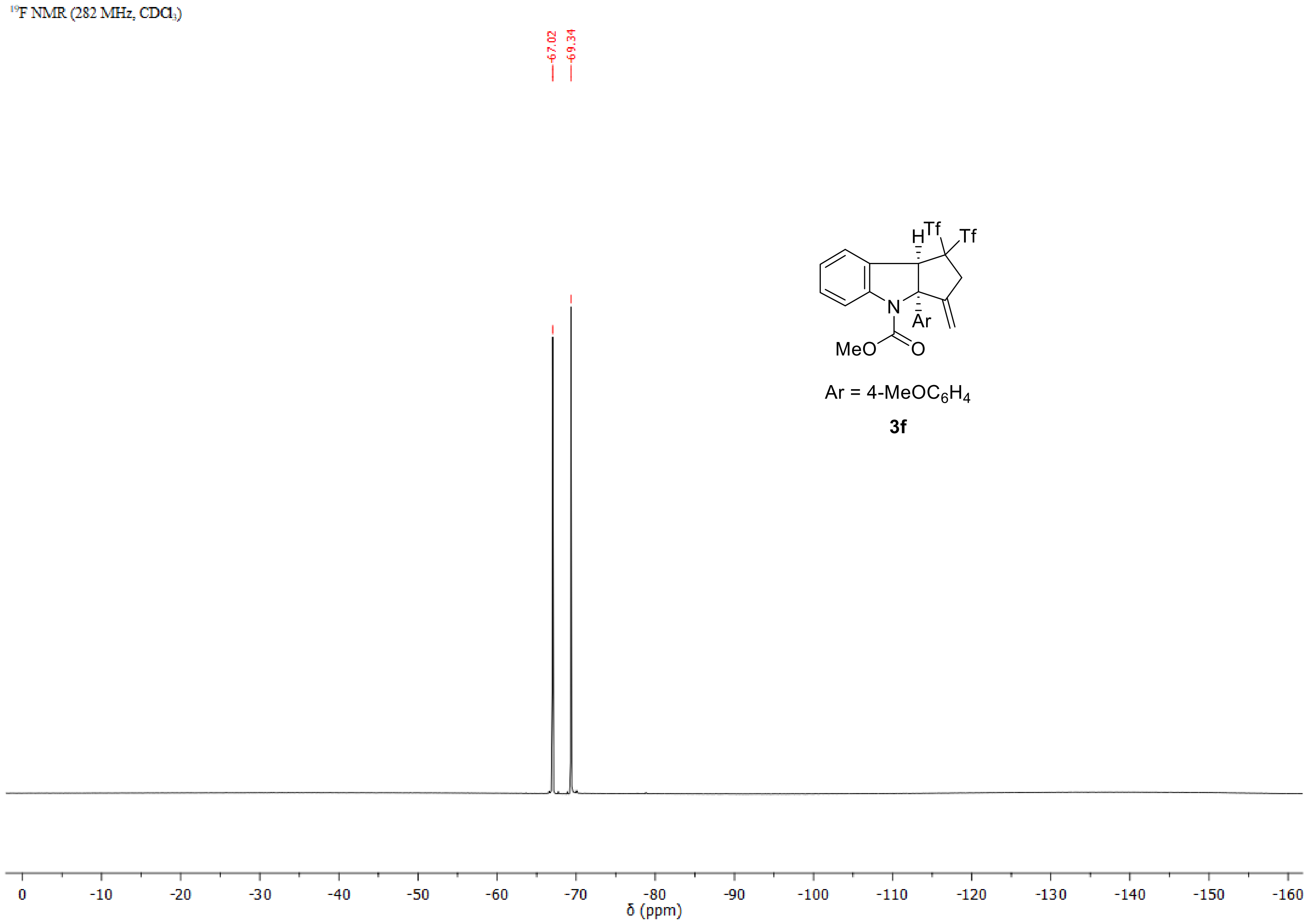

^{19}F NMR (282 MHz, CDCl$_3$)
-67.02
-69.34
H
Tf
Tf
N
Ar
MeO
O
Ar = 4-MeOC$_6$H$_4$
3f
0
-10
-20
-30
-40
-50
-60
-70
-80
-90
-100
-110
-120
-130
-140
-150
-160
δ (ppm)

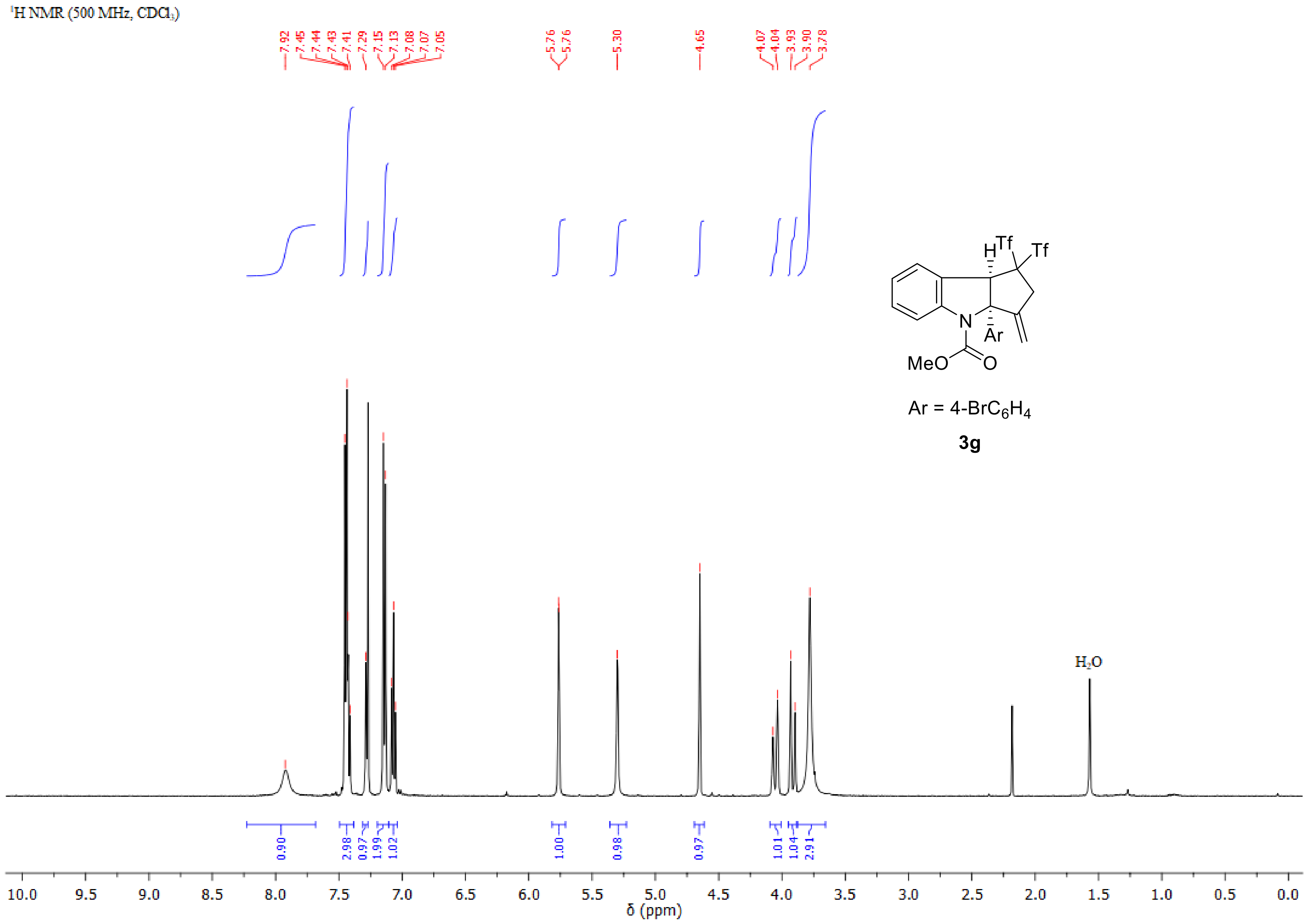

1H NMR (500 MHz, CDCl3)
7.92
7.45
7.44
7.43
7.41
7.29
7.15
7.13
7.08
7.07
7.05
5.76
5.76
5.30
4.65
4.07
4.04
3.93
3.90
3.78
H2O
0.90
2.98
0.97
1.99
1.02
1.00
0.98
0.97
1.01
1.04
2.91
δ (ppm)
H
Tf
Tf
N
Ar
MeO
O
Ar = 4-BrC6H4
3g

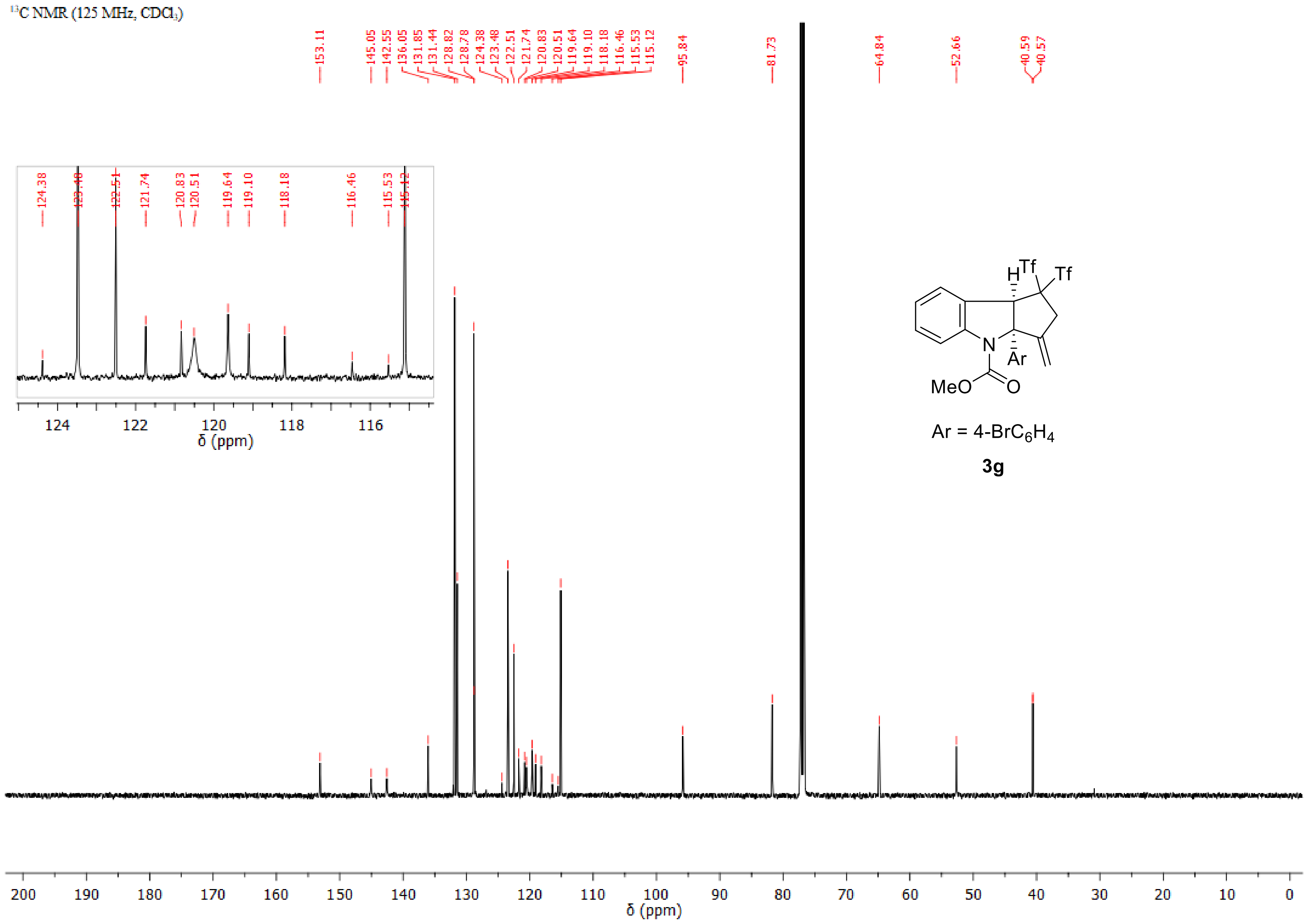
13C NMR (125 MHz, CDCl3)
153.11
145.05
142.55
136.05
131.85
131.44
128.82
128.78
124.38
123.48
122.51
121.74
120.83
120.51
119.64
119.10
118.18
116.46
115.53
115.12
95.84
81.73
64.84
52.66
40.59
40.57
δ (ppm)
H
Tf
Tf
N
Ar
MeO
O
Ar = 4-BrC6H4
3g

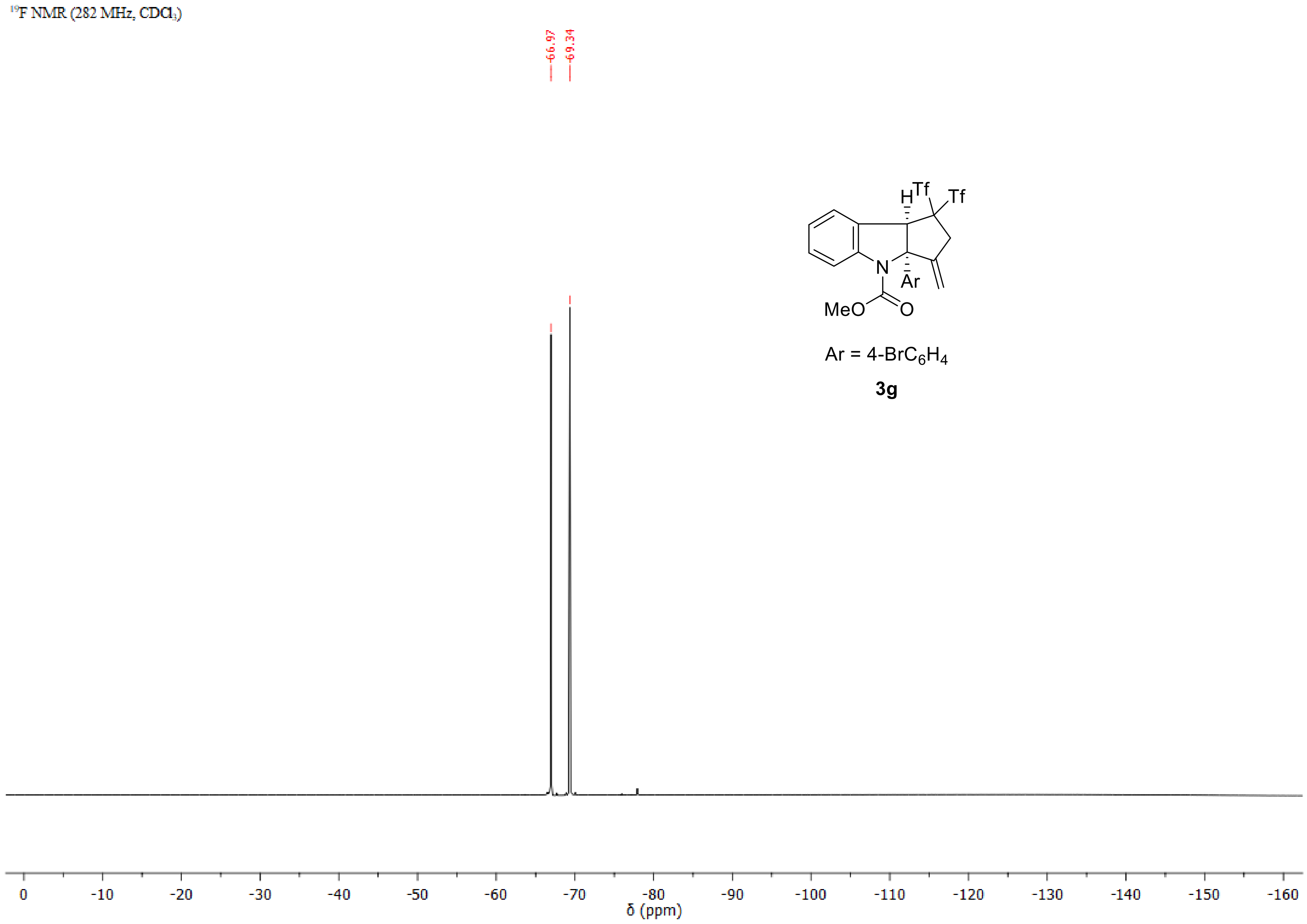

Ar = 4-BrC_6H_4

3g

^{1}H NMR (500 MHz, CDCl$_3$)

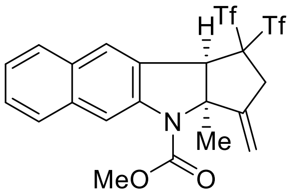

3h

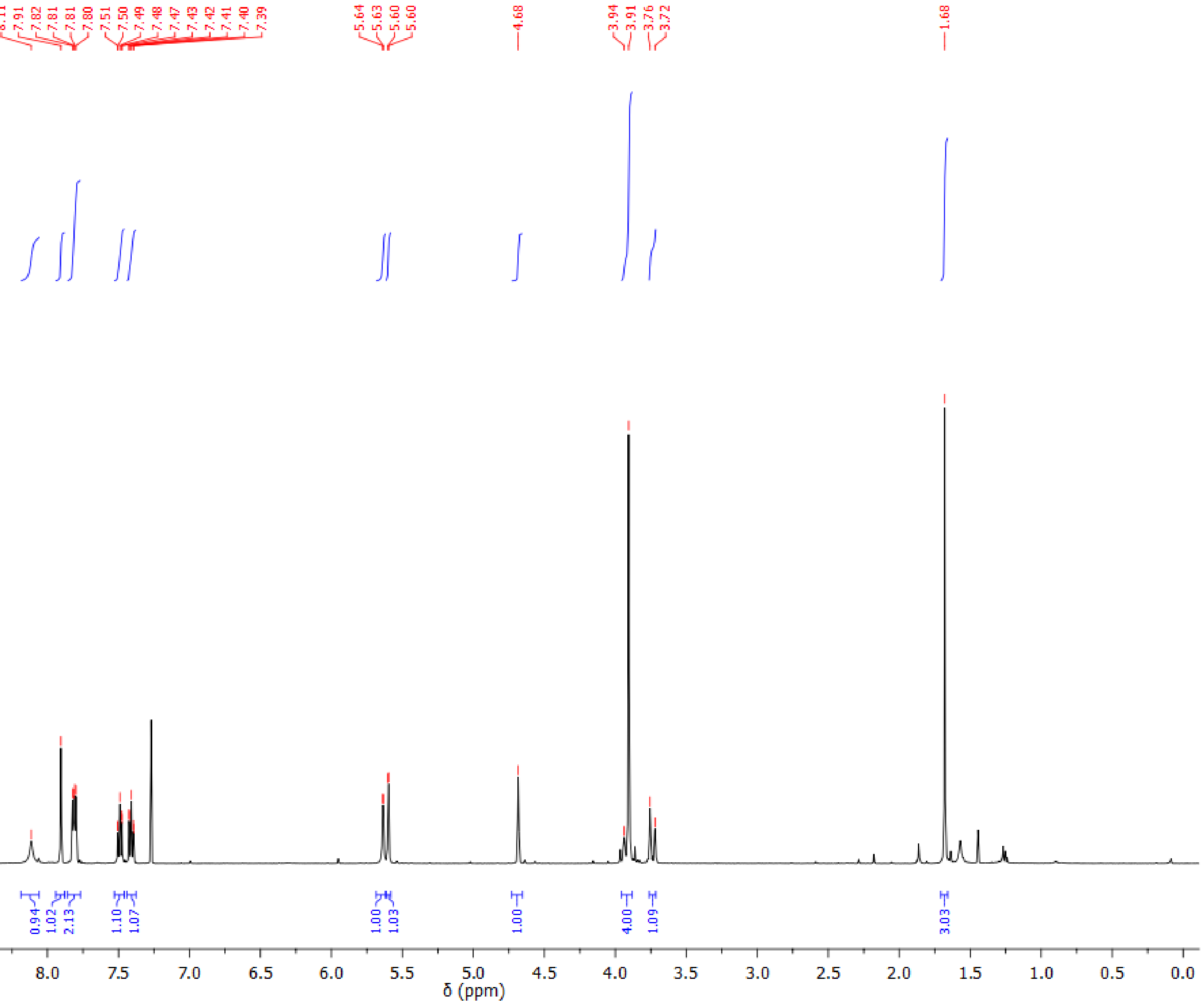

^{13}C NMR (125 MHz, $CDCl_3$)

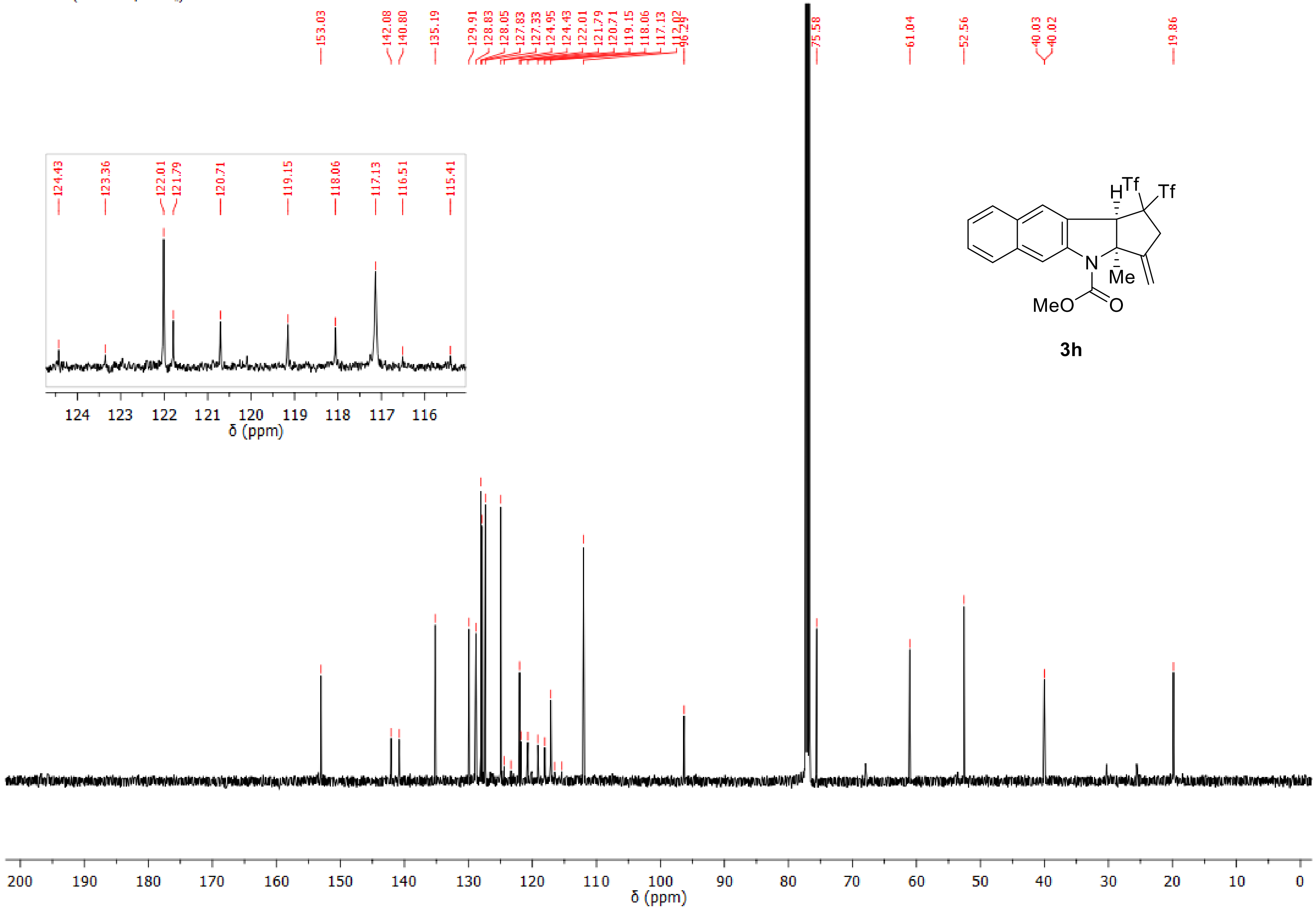

^{19}F NMR (282 MHz, $CDCl_3$)

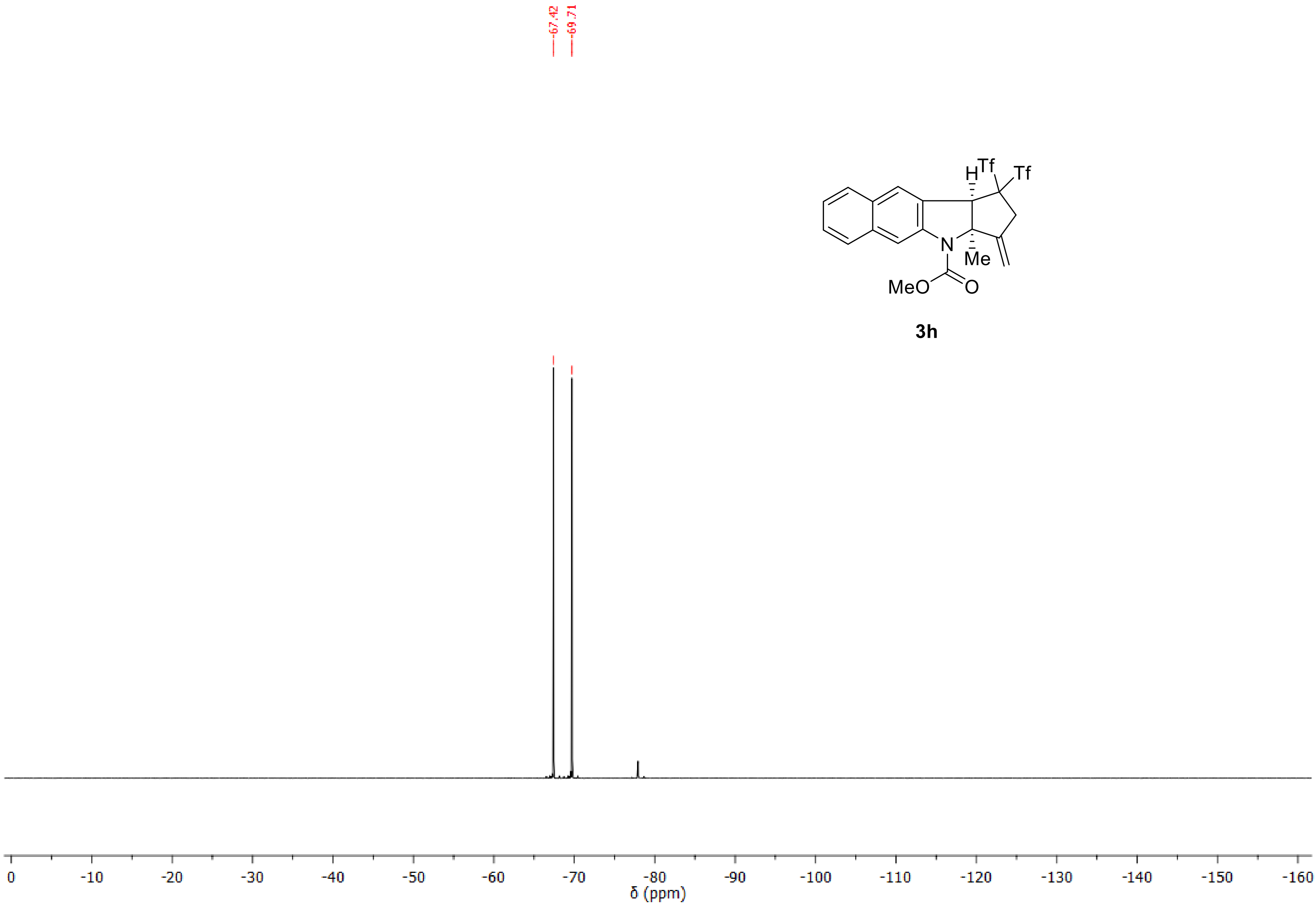

3h

NOE Experiment (500 MHz, $CDCl_3$). Irradiation CH_3

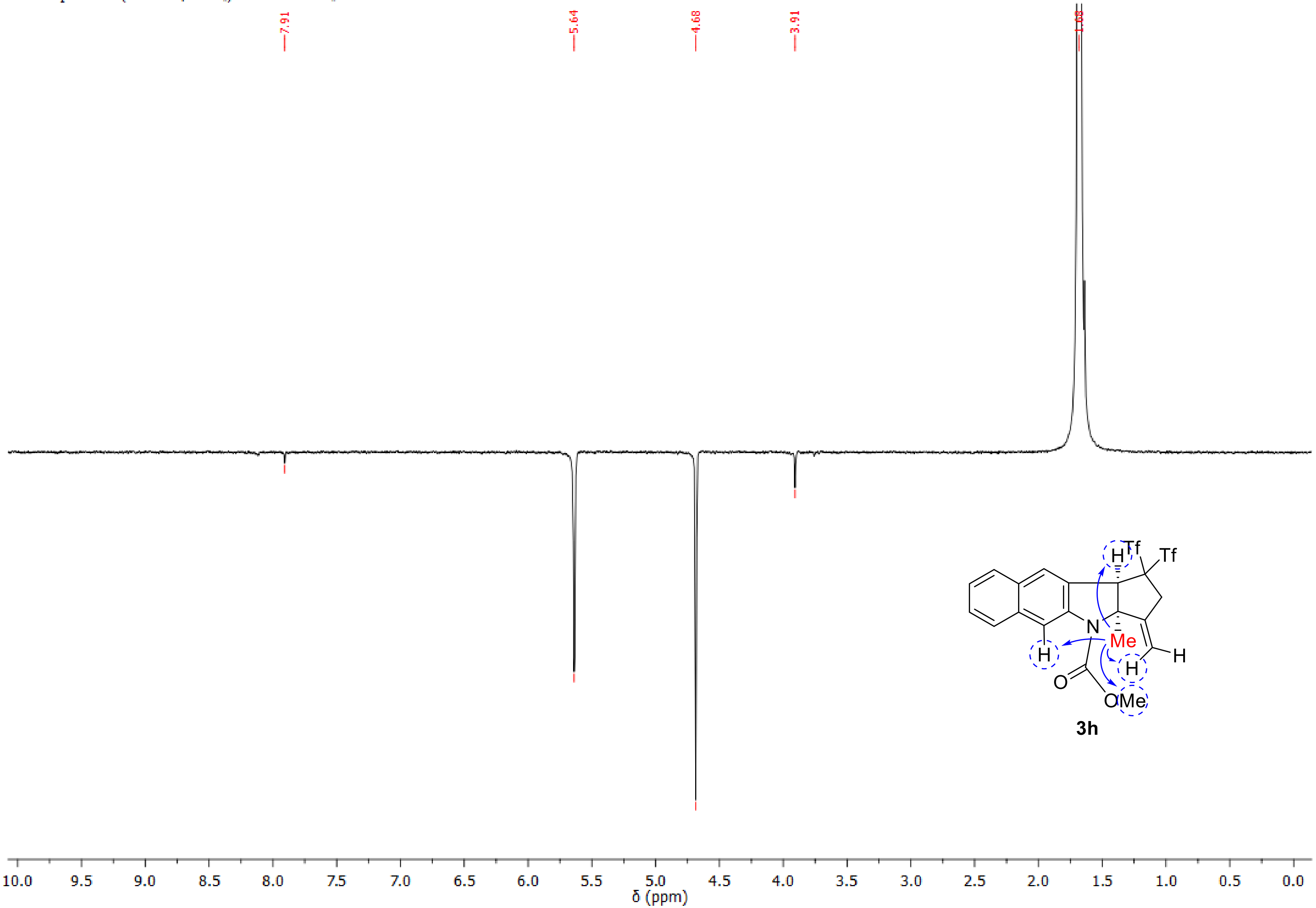

3h

NOE Experiment (500 MHz, $CDCl_3$). Irradiation CH

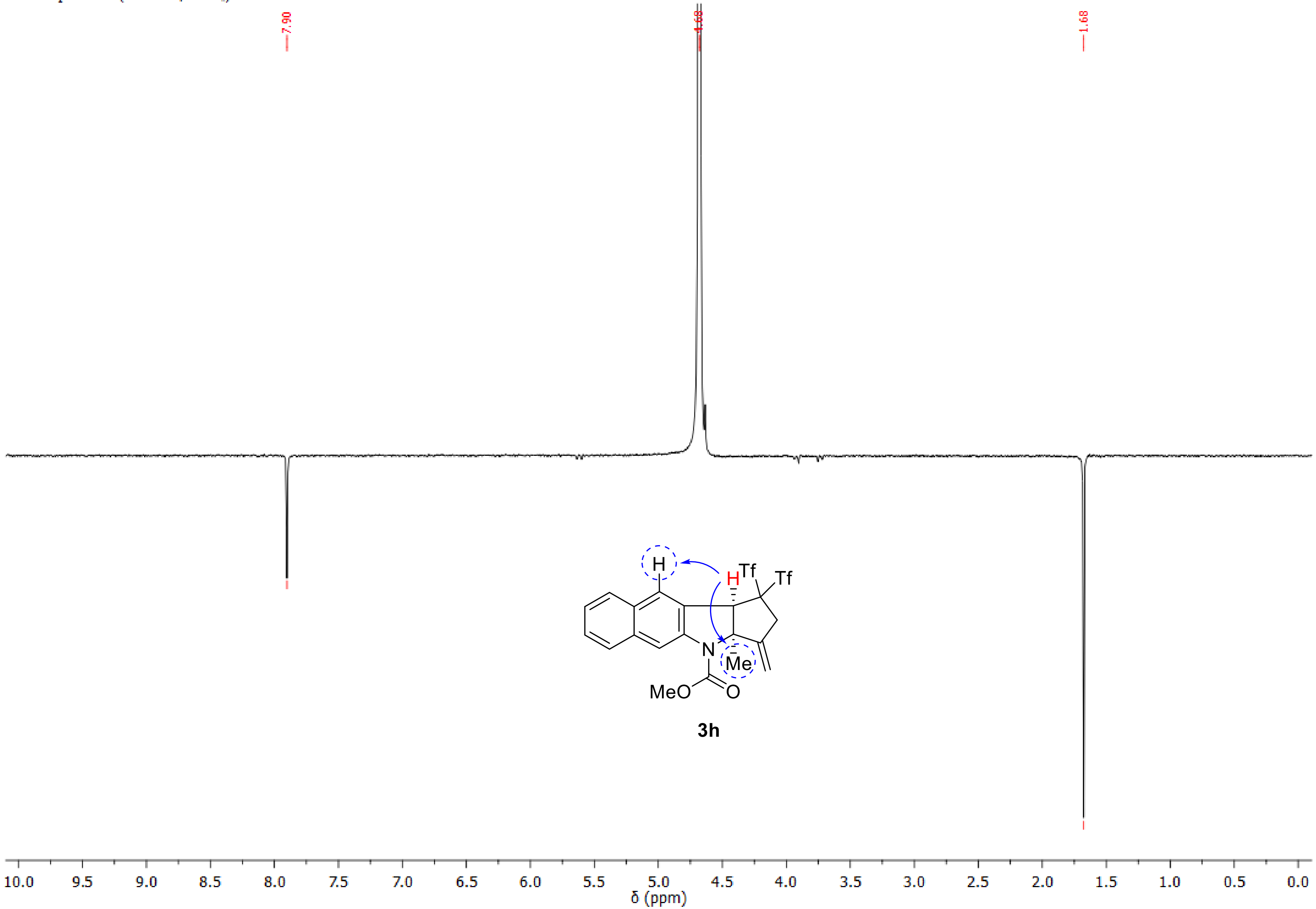

^{1}H NMR (500 MHz, $CDCl_3$)

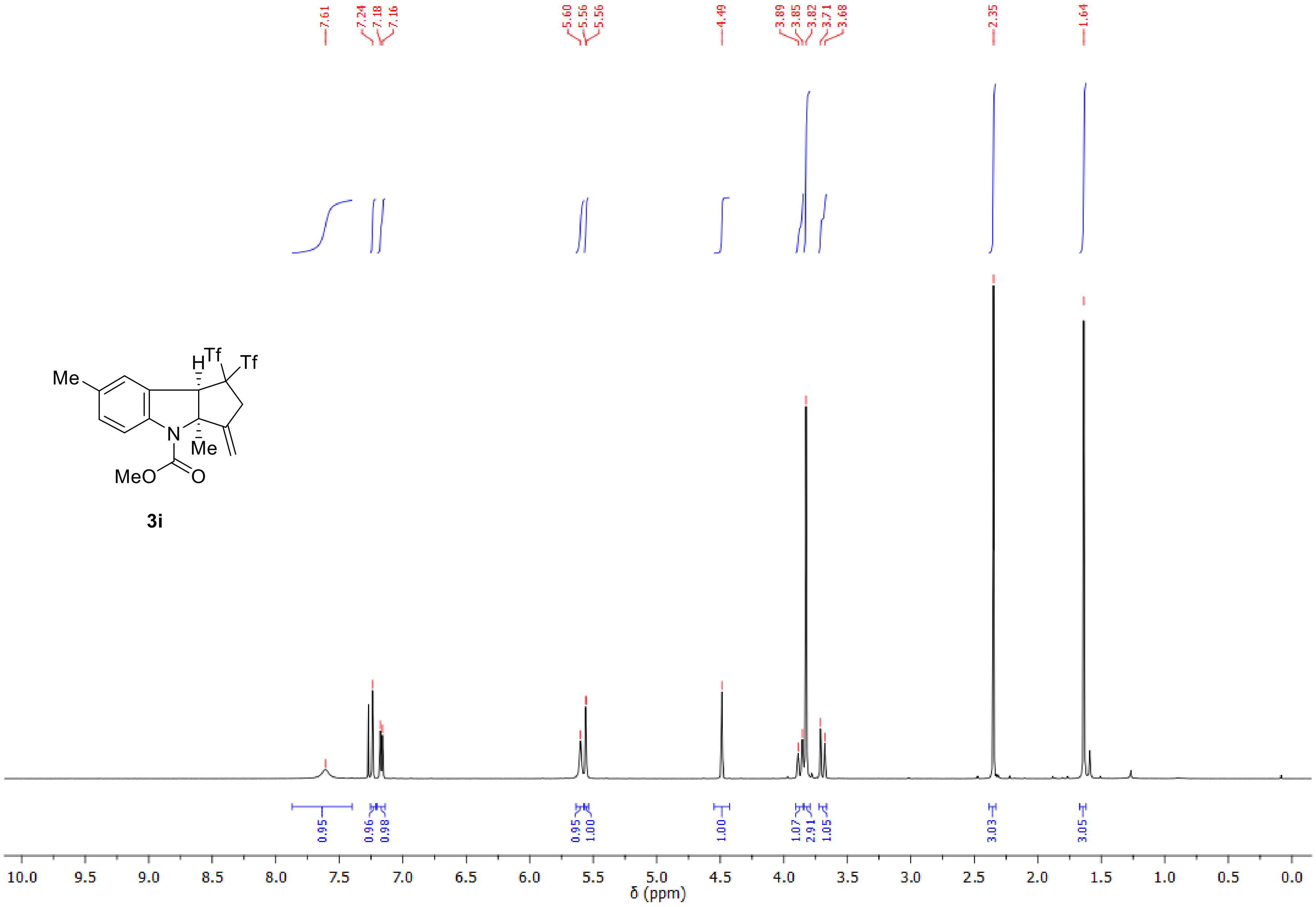

3i

^{13}C NMR (125 MHz, $CDCl_3$)

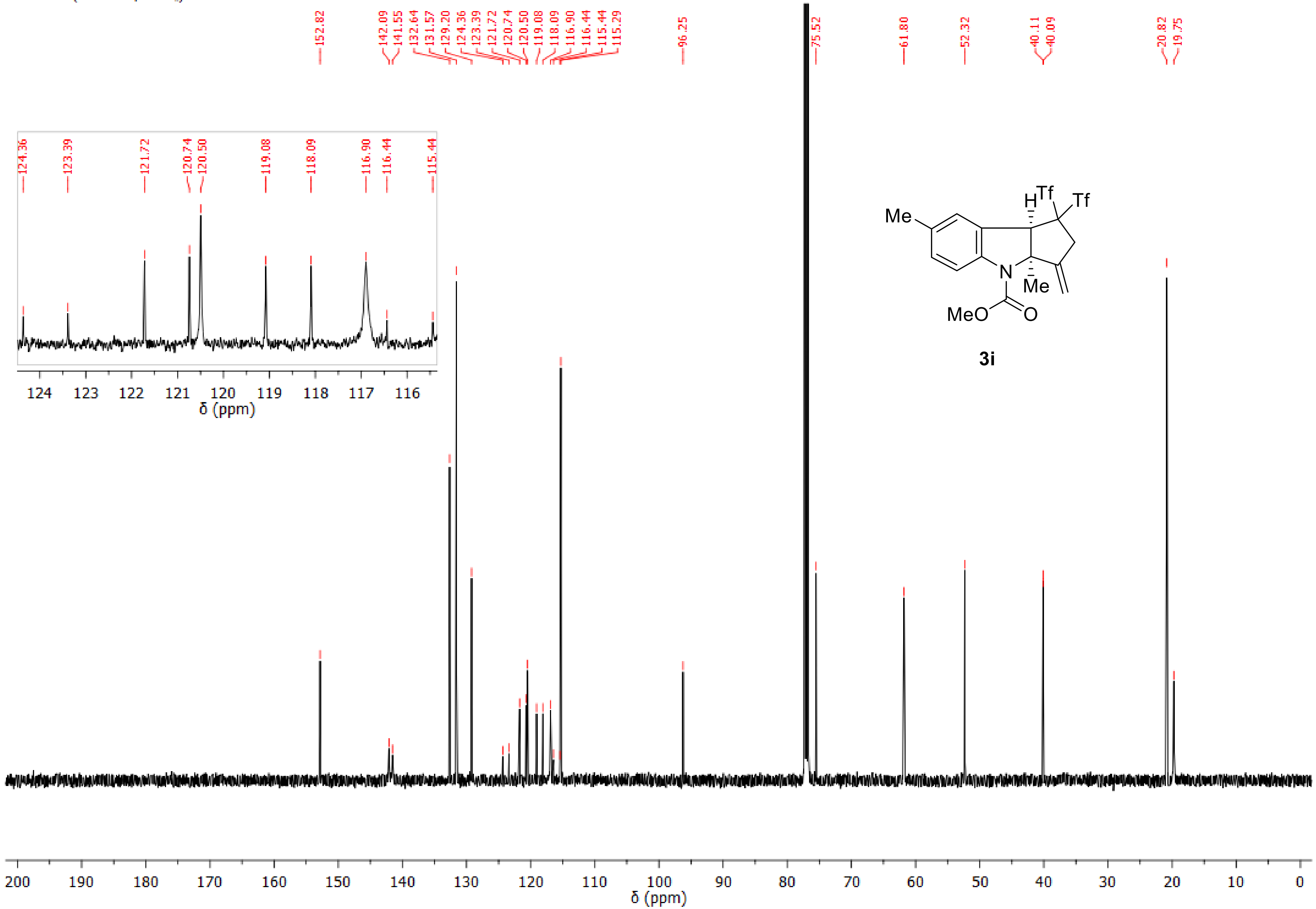

3i

^{19}F NMR (282 MHz, $CDCl_3$)

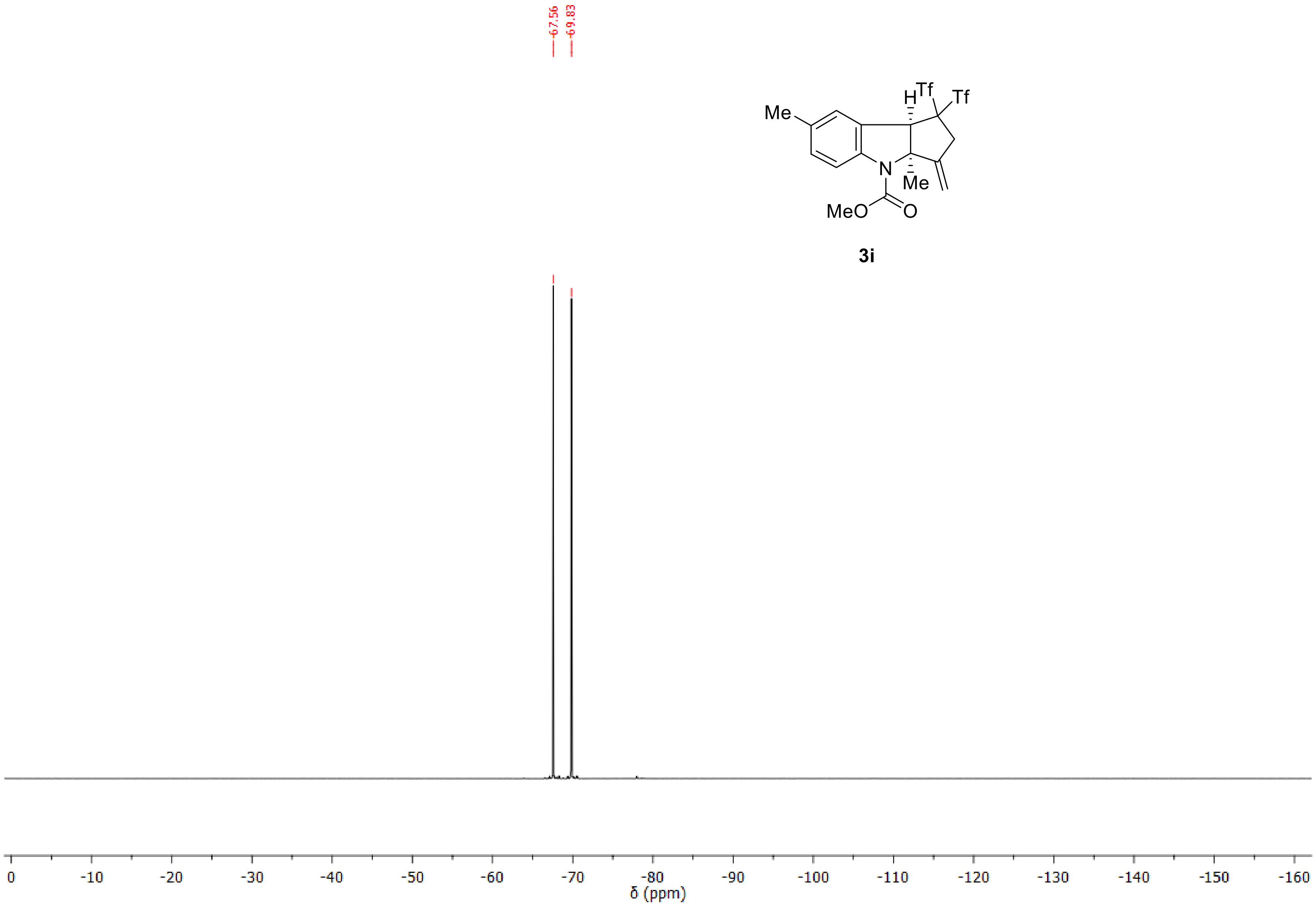

^1H NMR (500 MHz, $CDCl_3$)

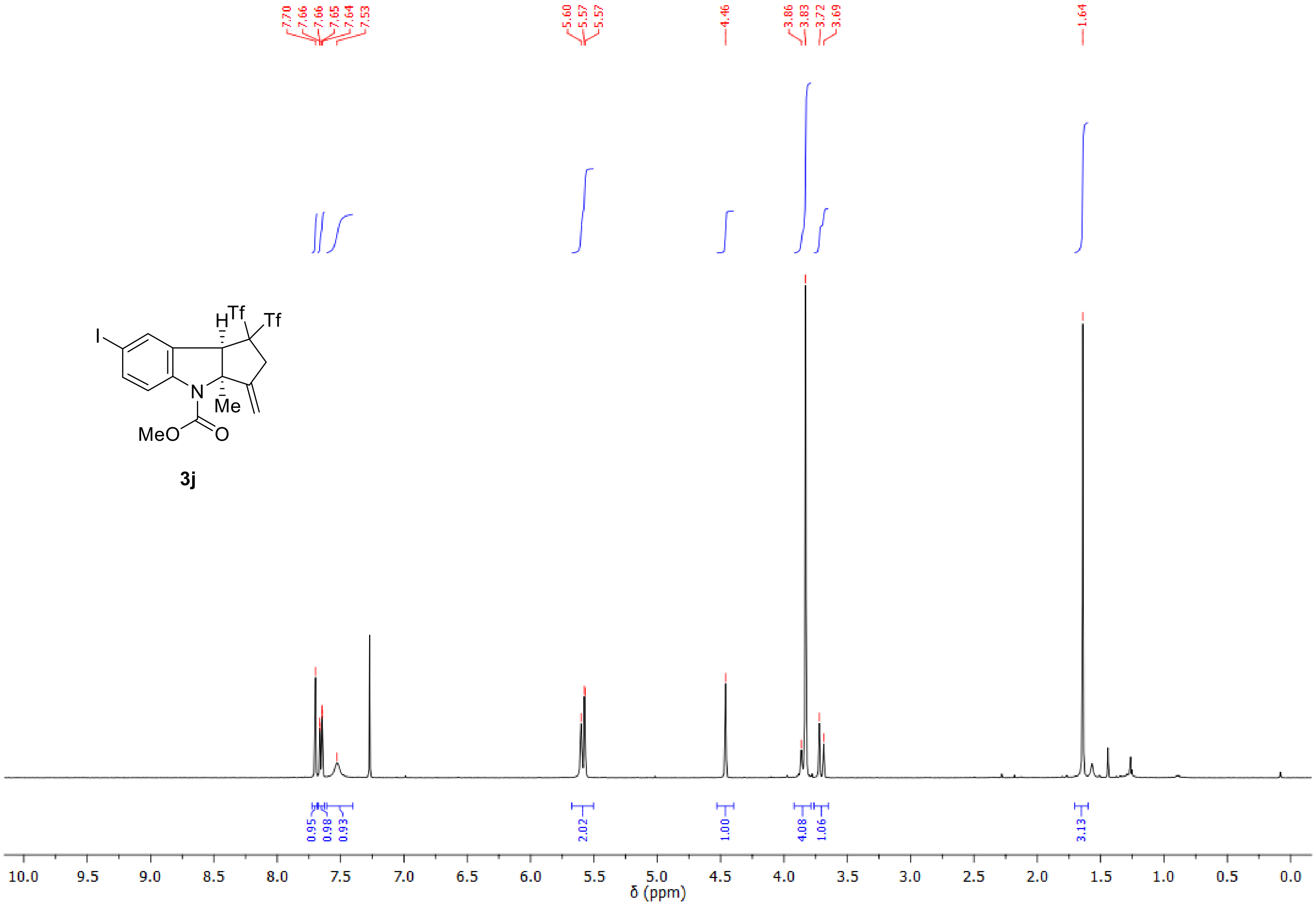

^{13}C NMR (125 MHz, $CDCl_3$)

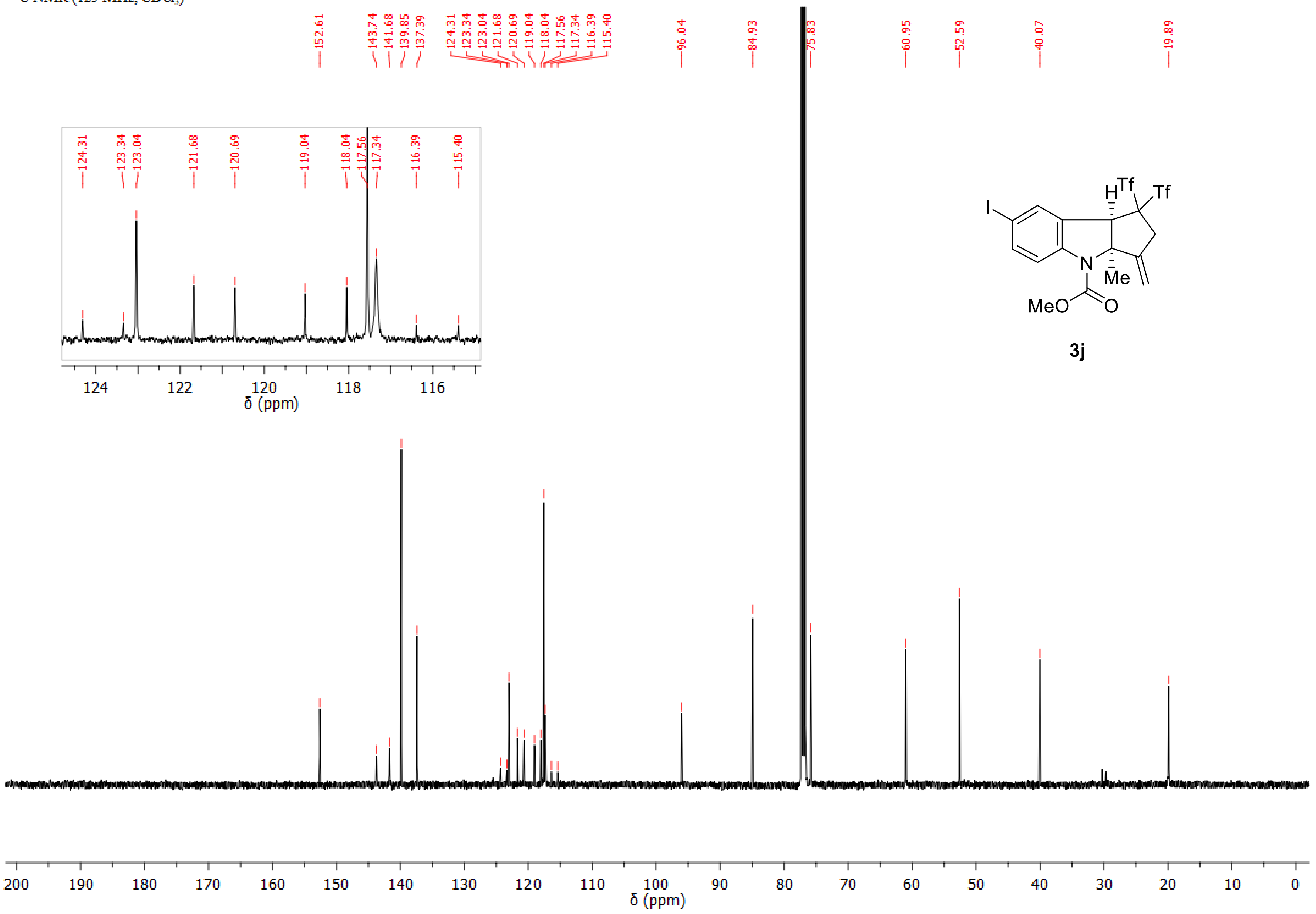

^{19}F NMR (282 MHz, CDCl$_3$)

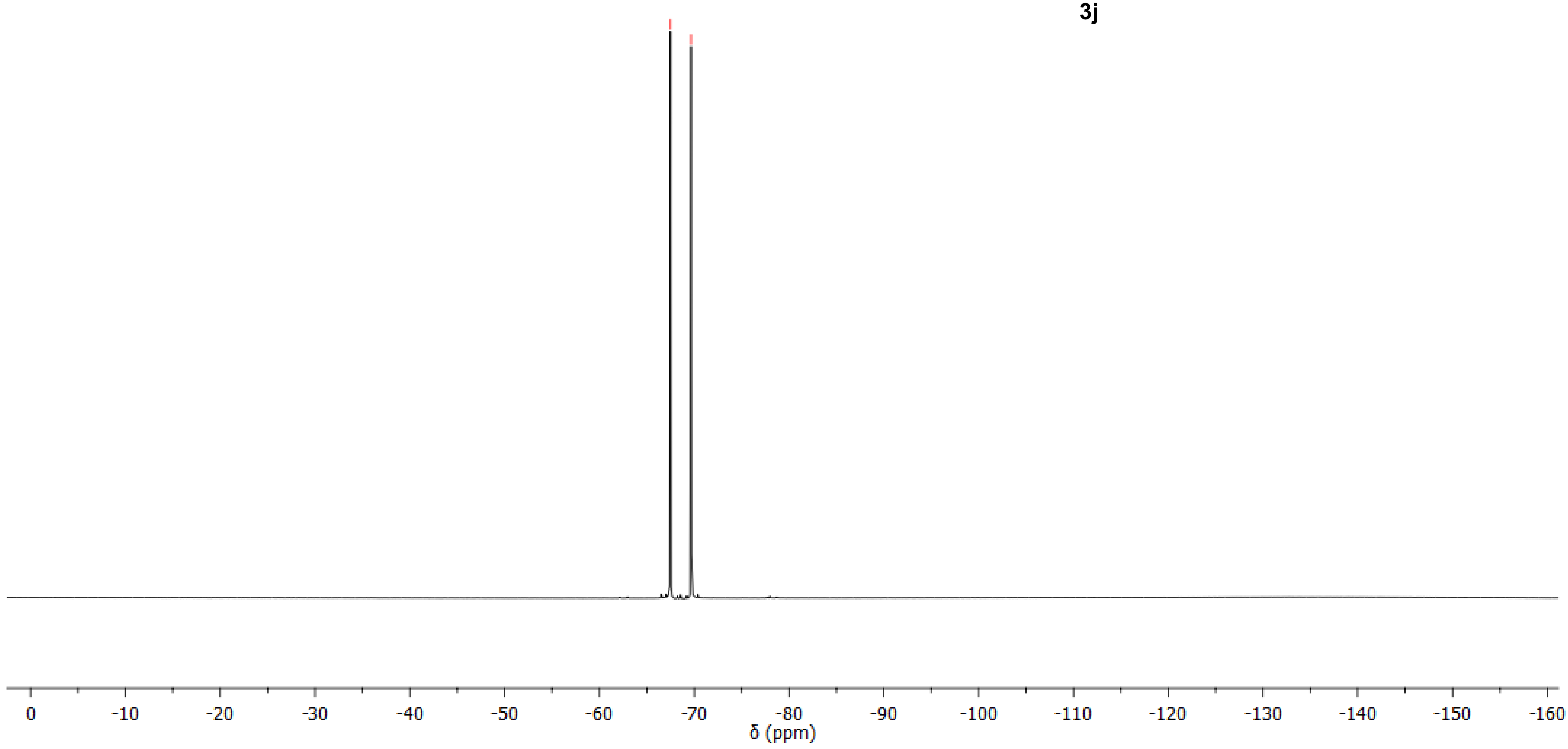

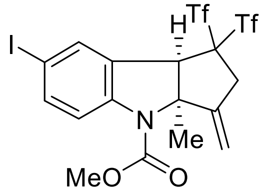

3j

^{1}H NMR (500 MHz, $CDCl_3$)

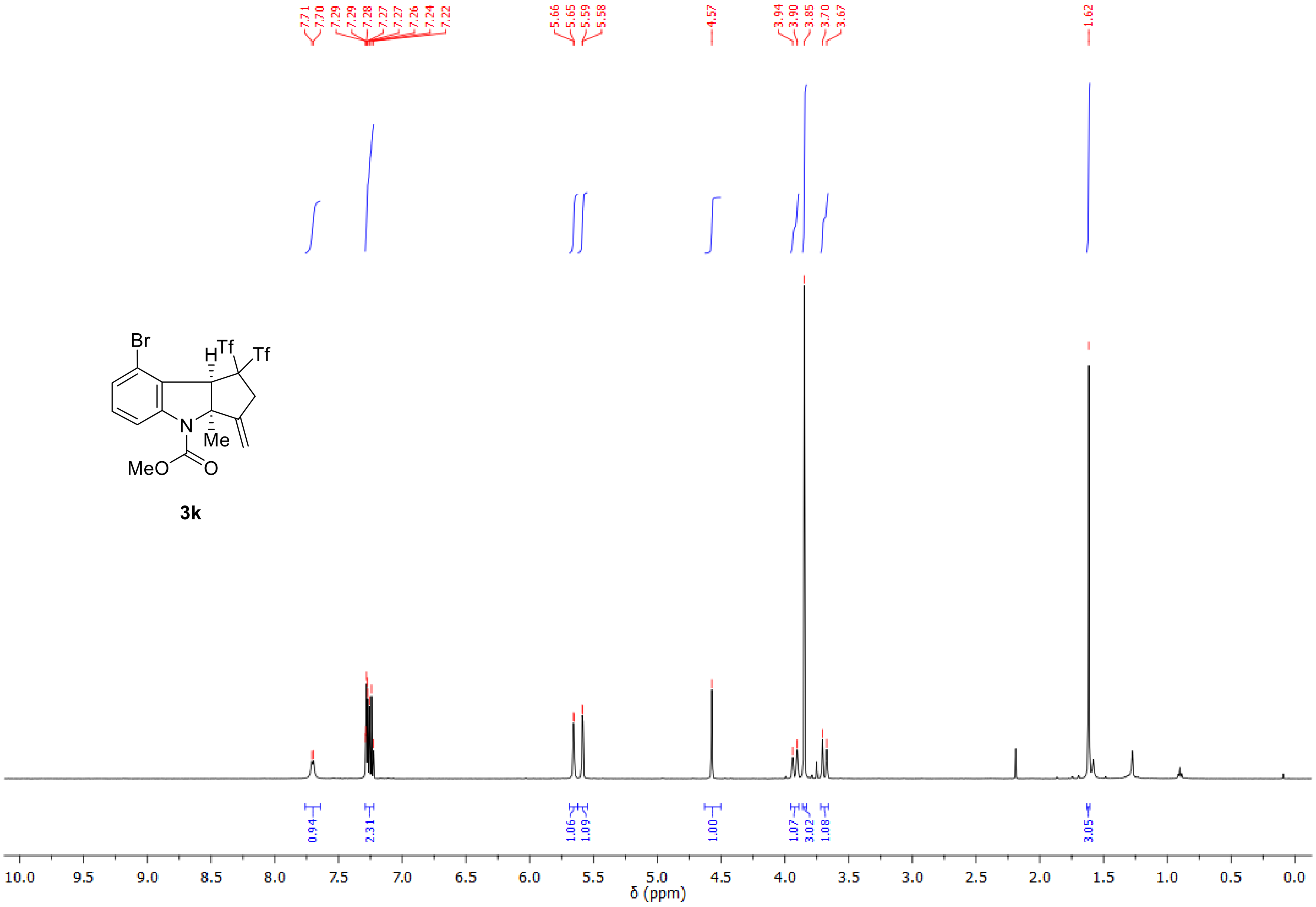

^{13}C NMR (125 MHz, $CDCl_3$)

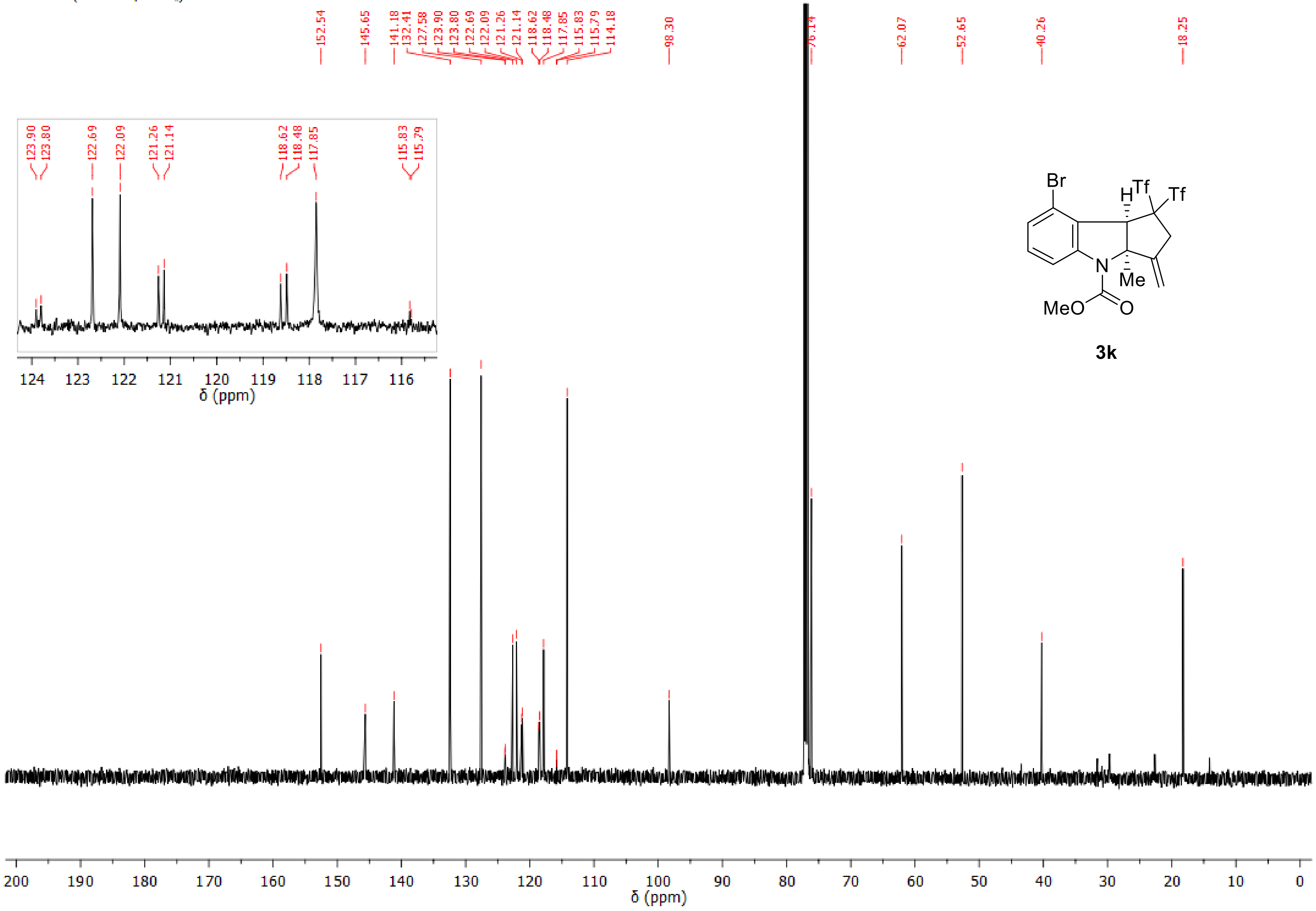

3k

^{19}F NMR (282 MHz, $CDCl_3$)

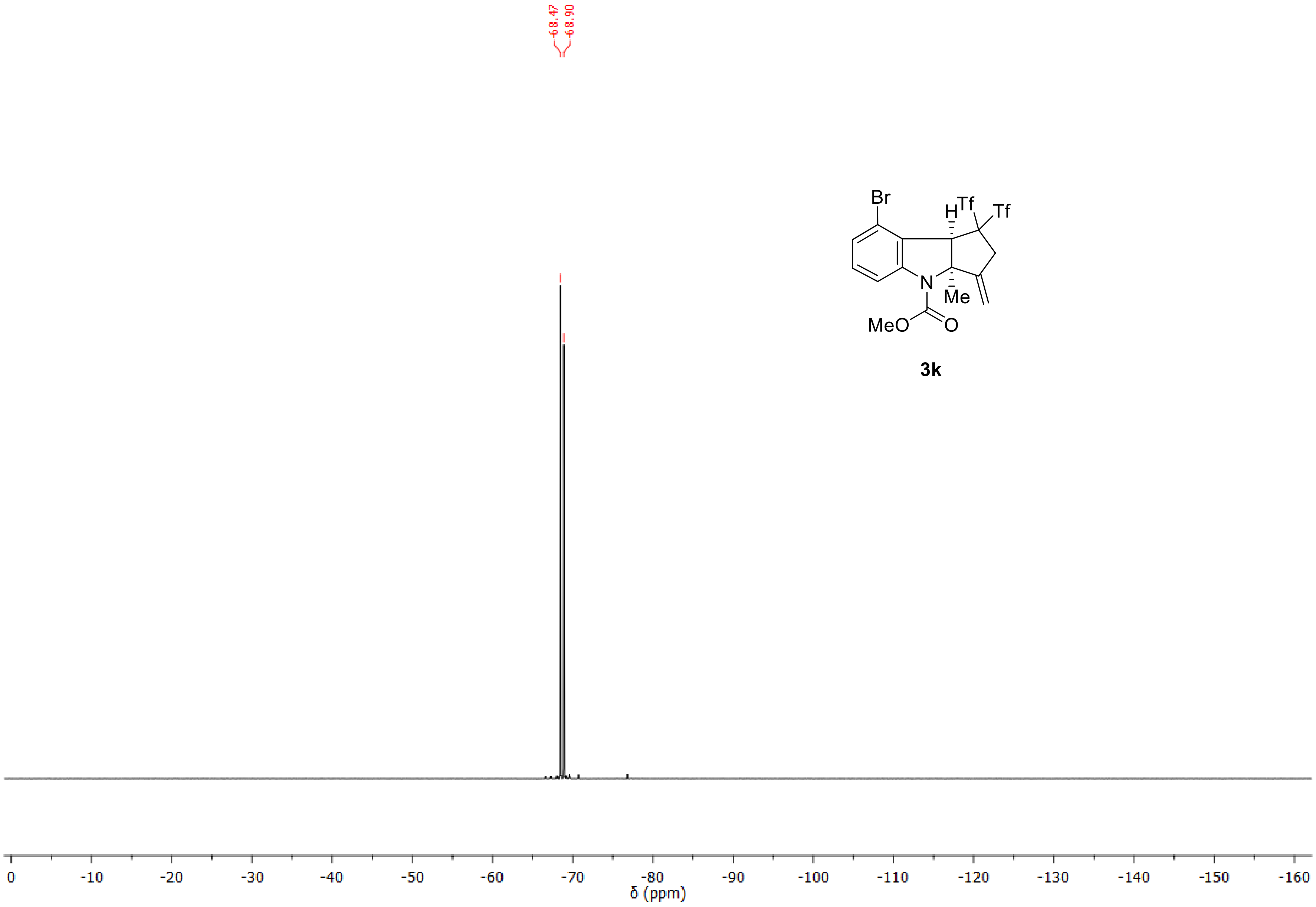

3k

^{1}H NMR (500 MHz, $CDCl_3$)

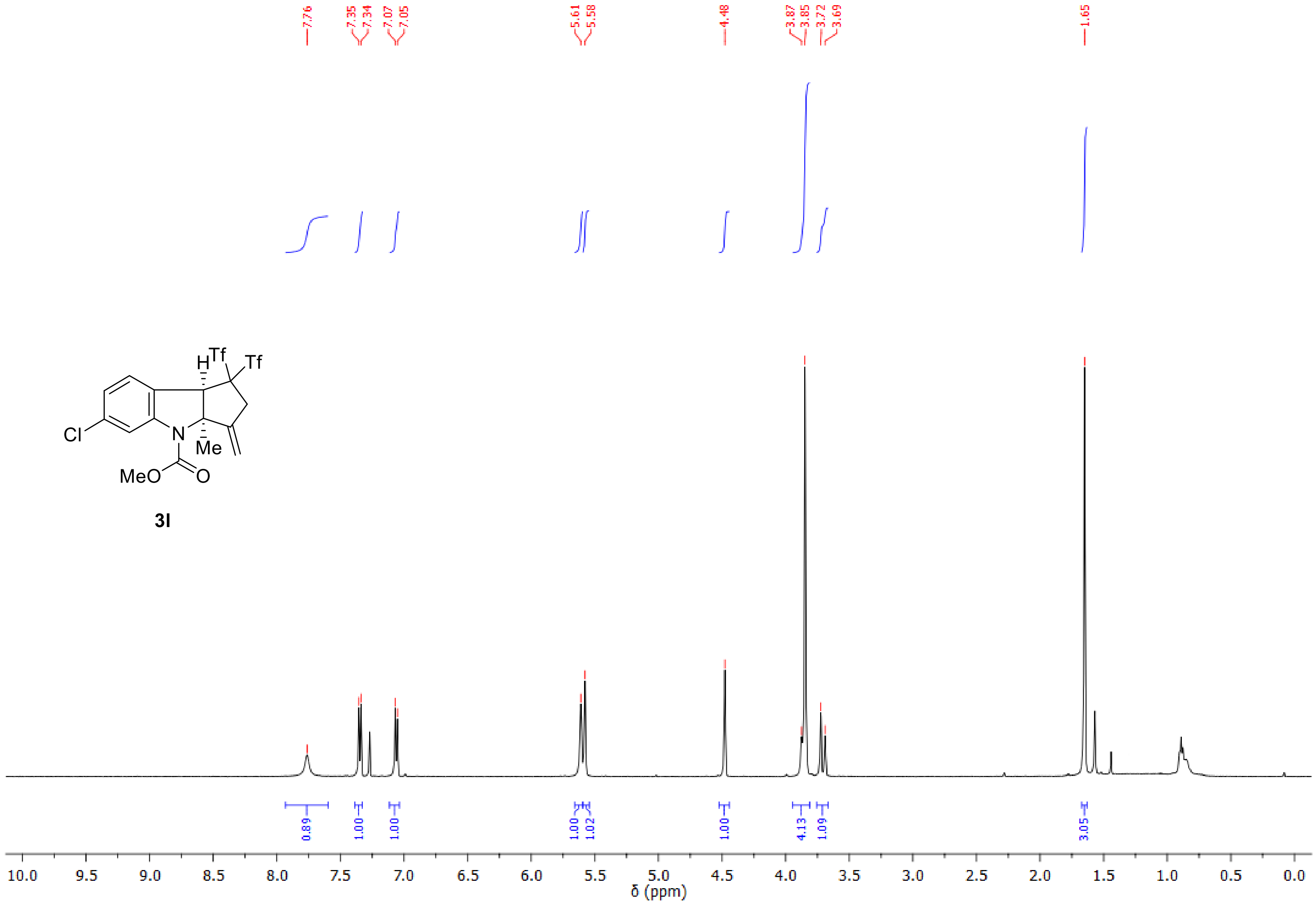

^{13}C NMR (125 MHz, CDCl$_3$)

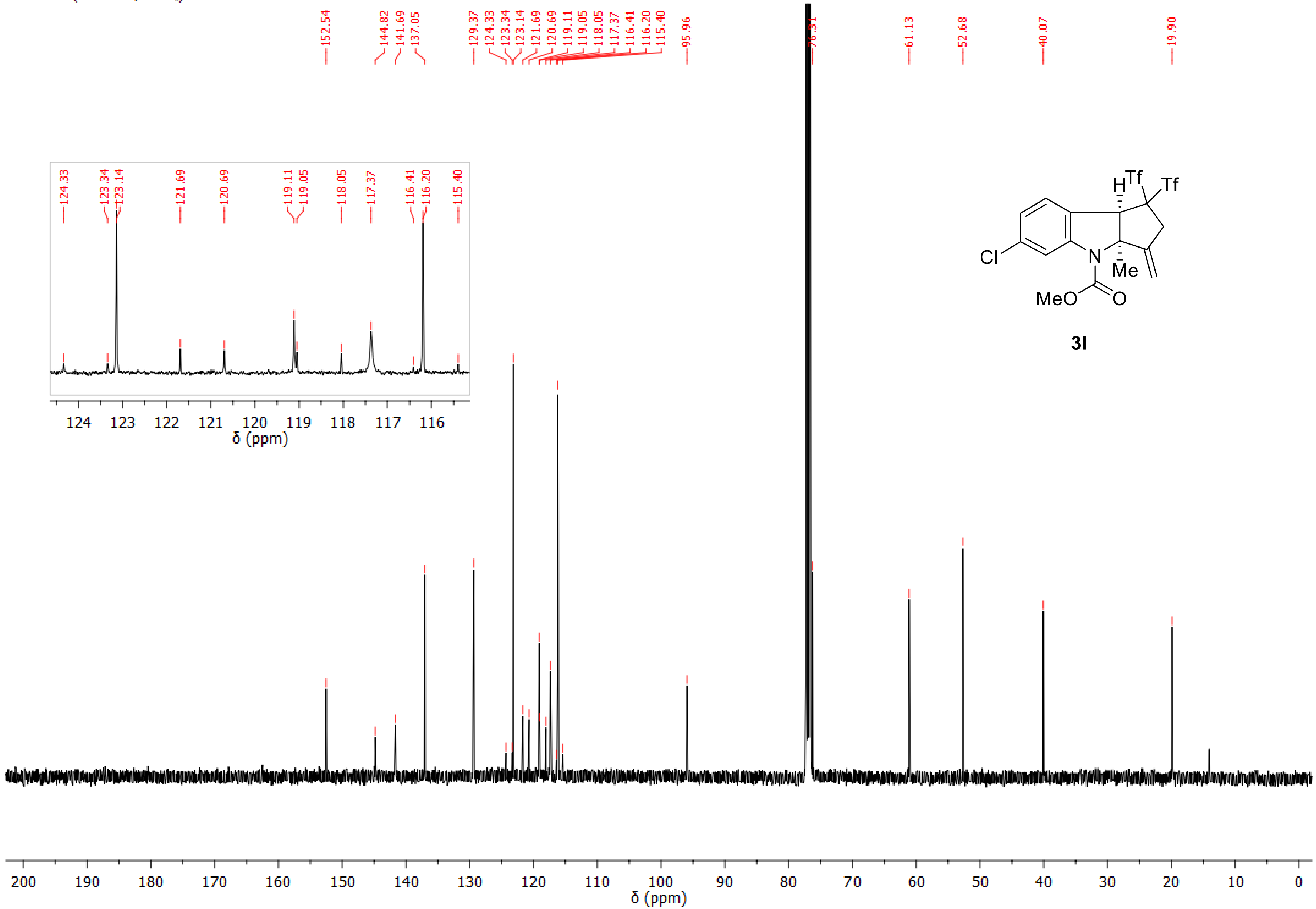

3l

^{19}F NMR (282 MHz, $CDCl_3$)

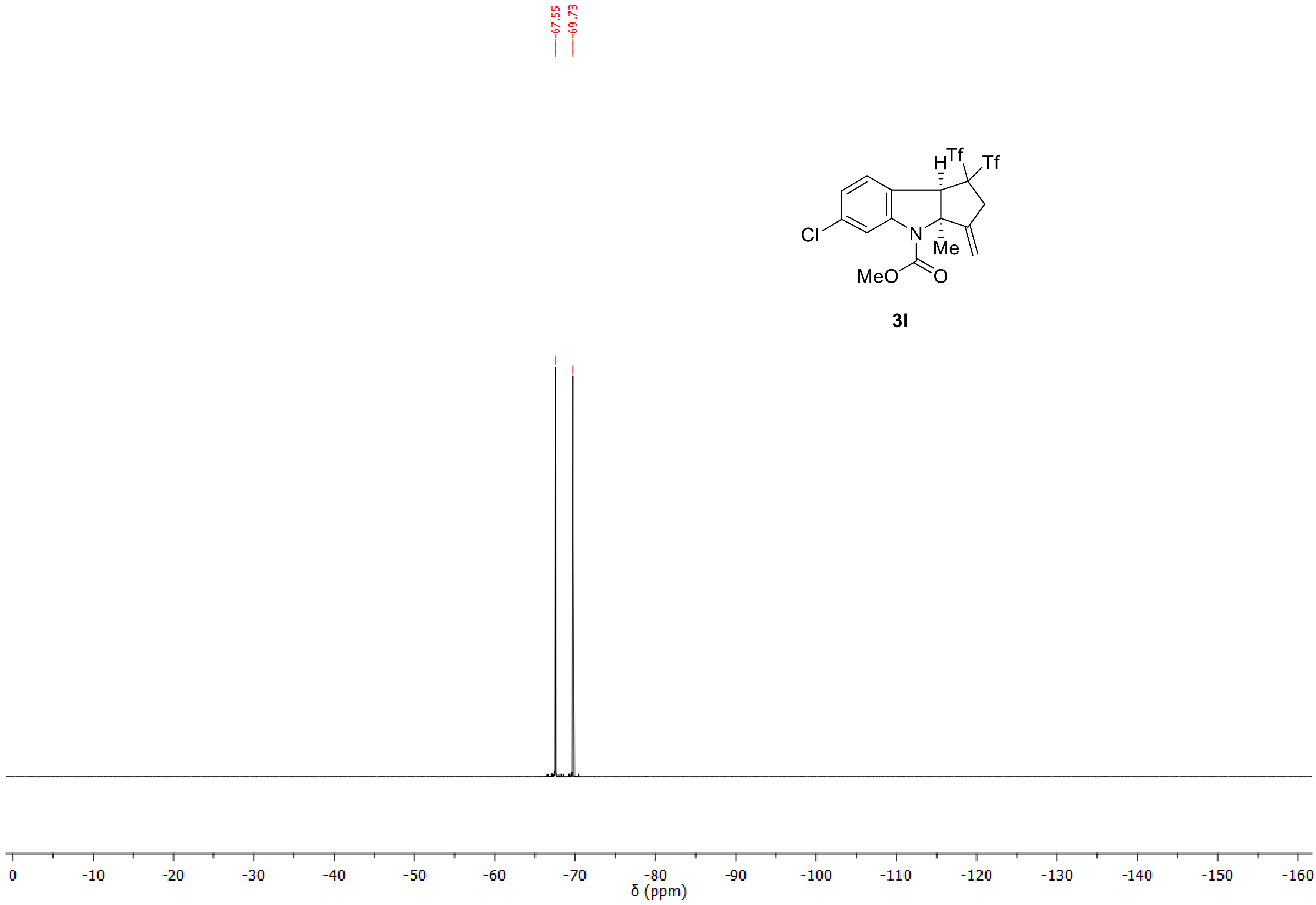

3l

^{1}H NMR (500 MHz, $CDCl_3$)

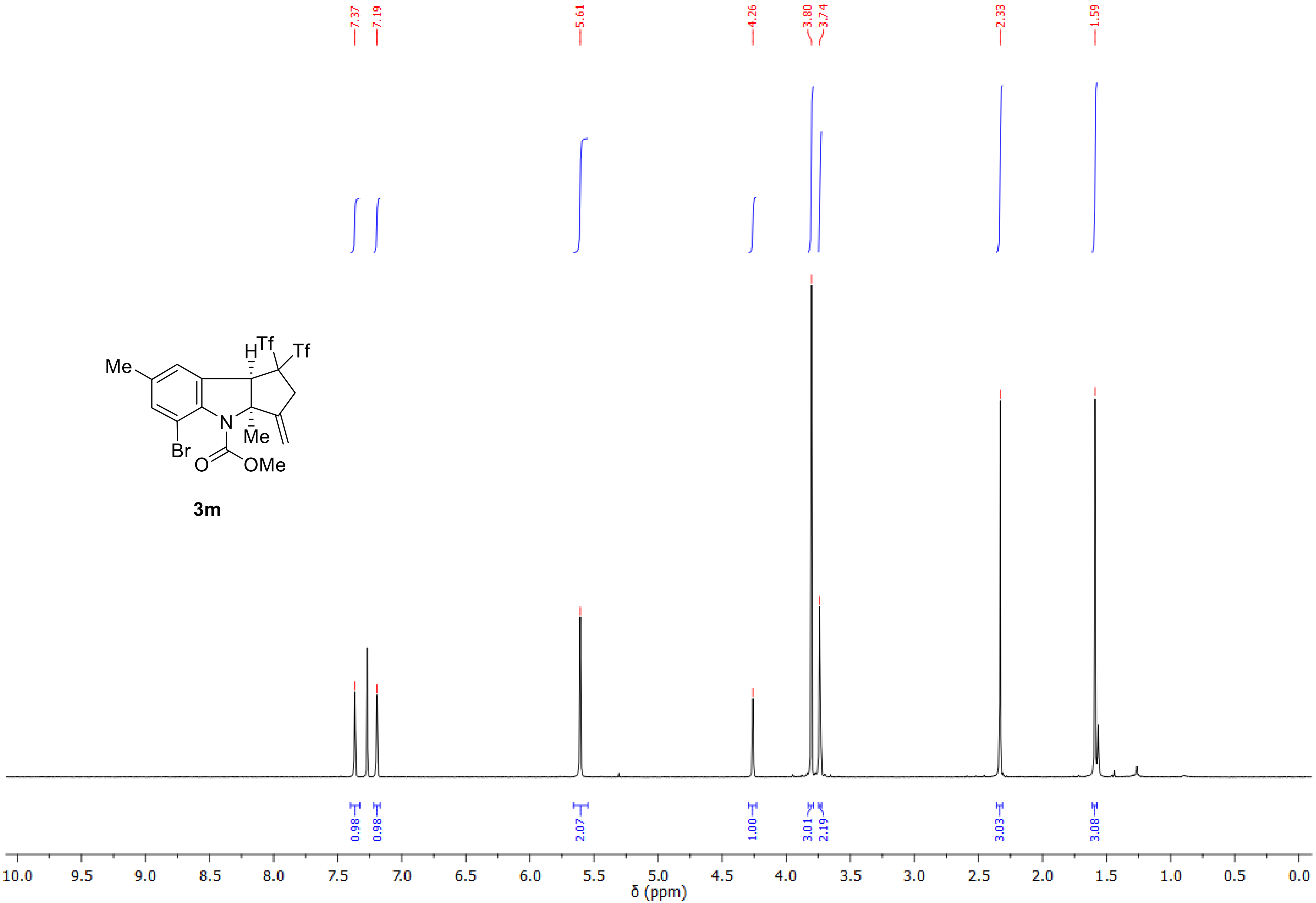

^{13}C NMR (125 MHz, $CDCl_3$)

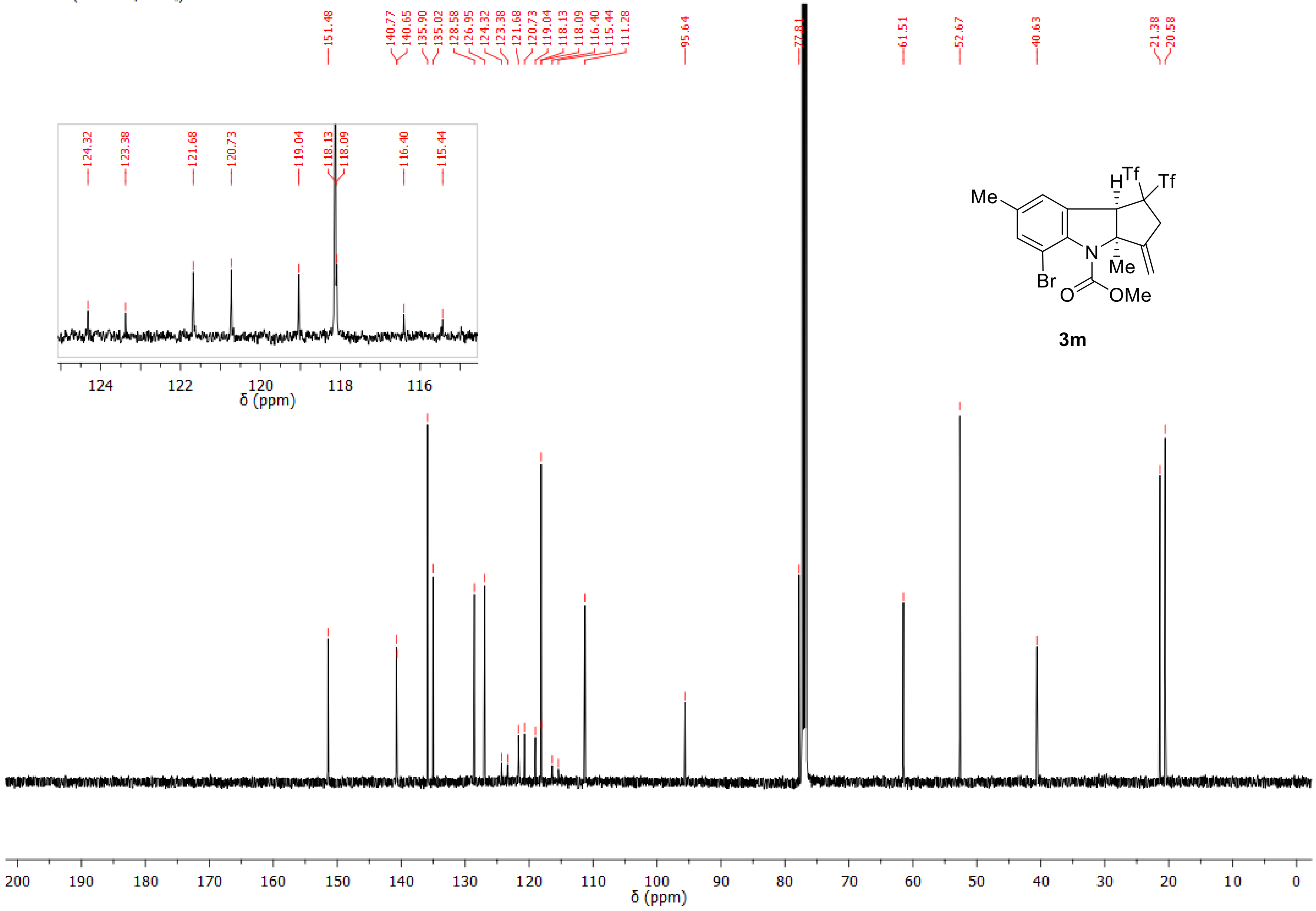

3m

^{19}F NMR (282 MHz, $CDCl_3$)

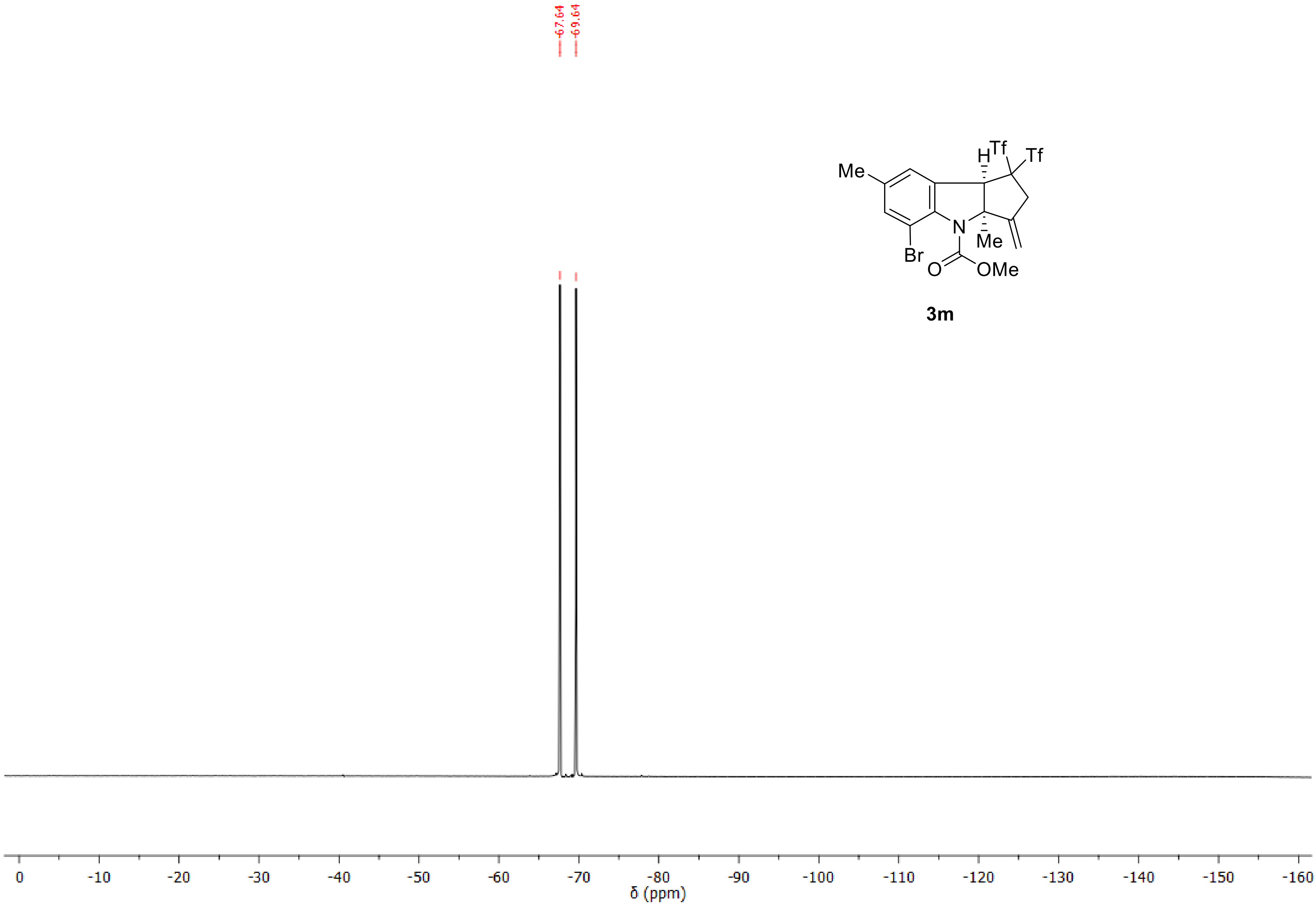

3m

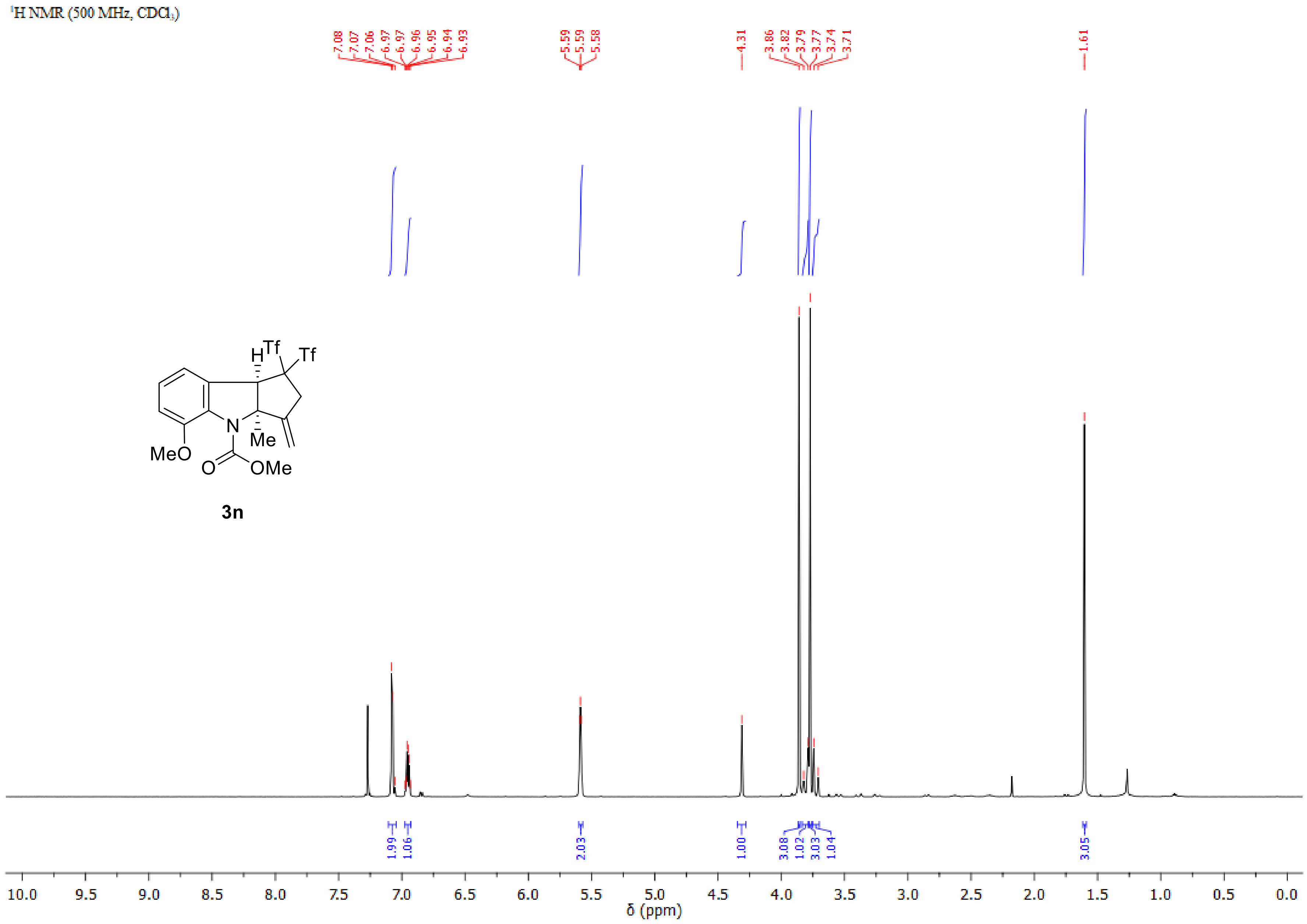
1H NMR (500 MHz, CDCl3)
7.08 7.07 7.06 6.97 6.97 6.96 6.95 6.94 6.93
5.59 5.59 5.58
4.31
3.86 3.82 3.79 3.77 3.74 3.71
1.61
H Tf Tf
N Me
MeO
O OMe
3n
1.99 1.06 2.03 1.00 3.08 1.02 3.03 1.04 3.05
10.0 9.5 9.0 8.5 8.0 7.5 7.0 6.5 6.0 5.5 5.0 4.5 4.0 3.5 3.0 2.5 2.0 1.5 1.0 0.5 0.0
δ (ppm)

^{13}C NMR (125 MHz, $CDCl_3$)

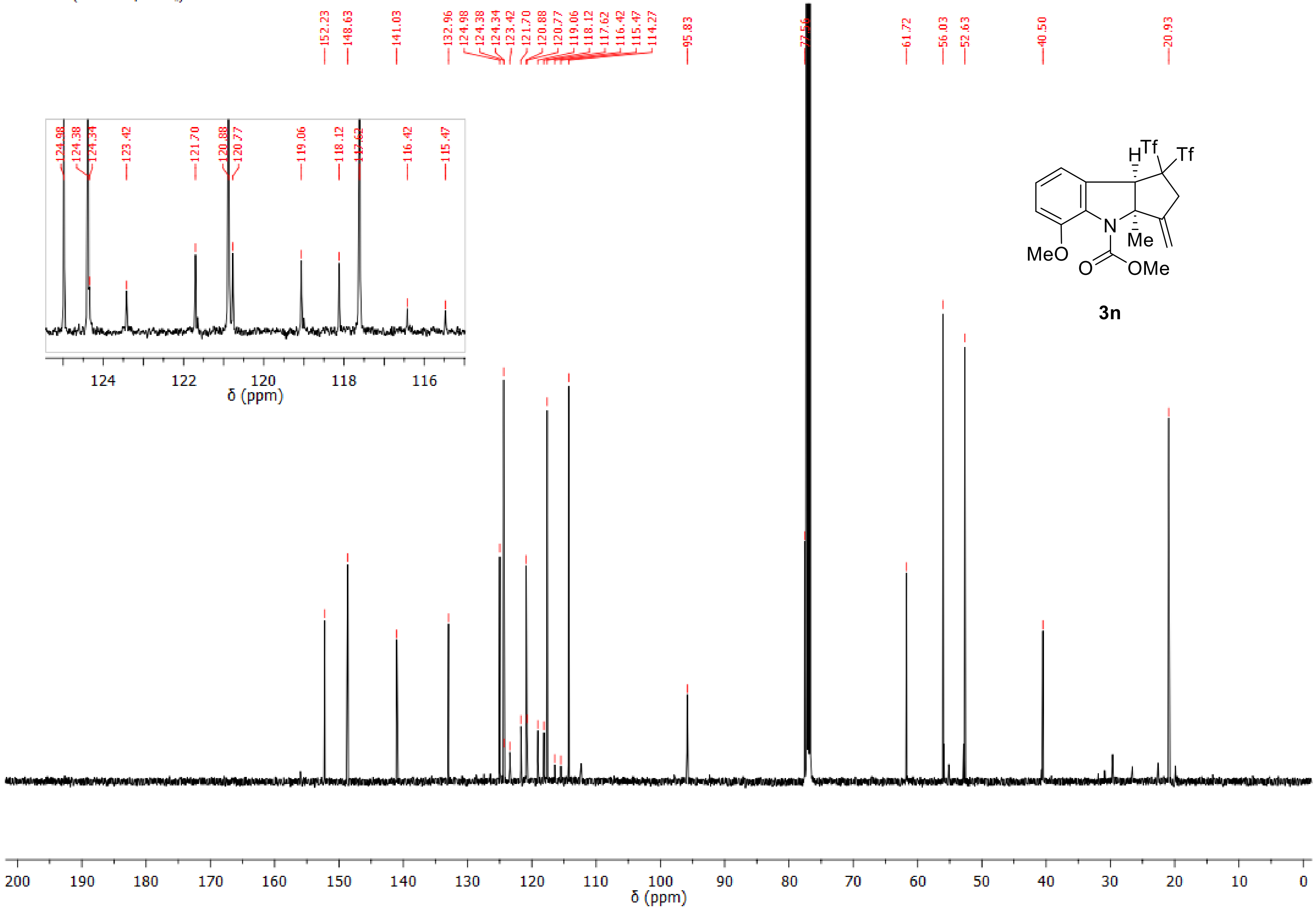

^{19}F NMR (282 MHz, $CDCl_3$)

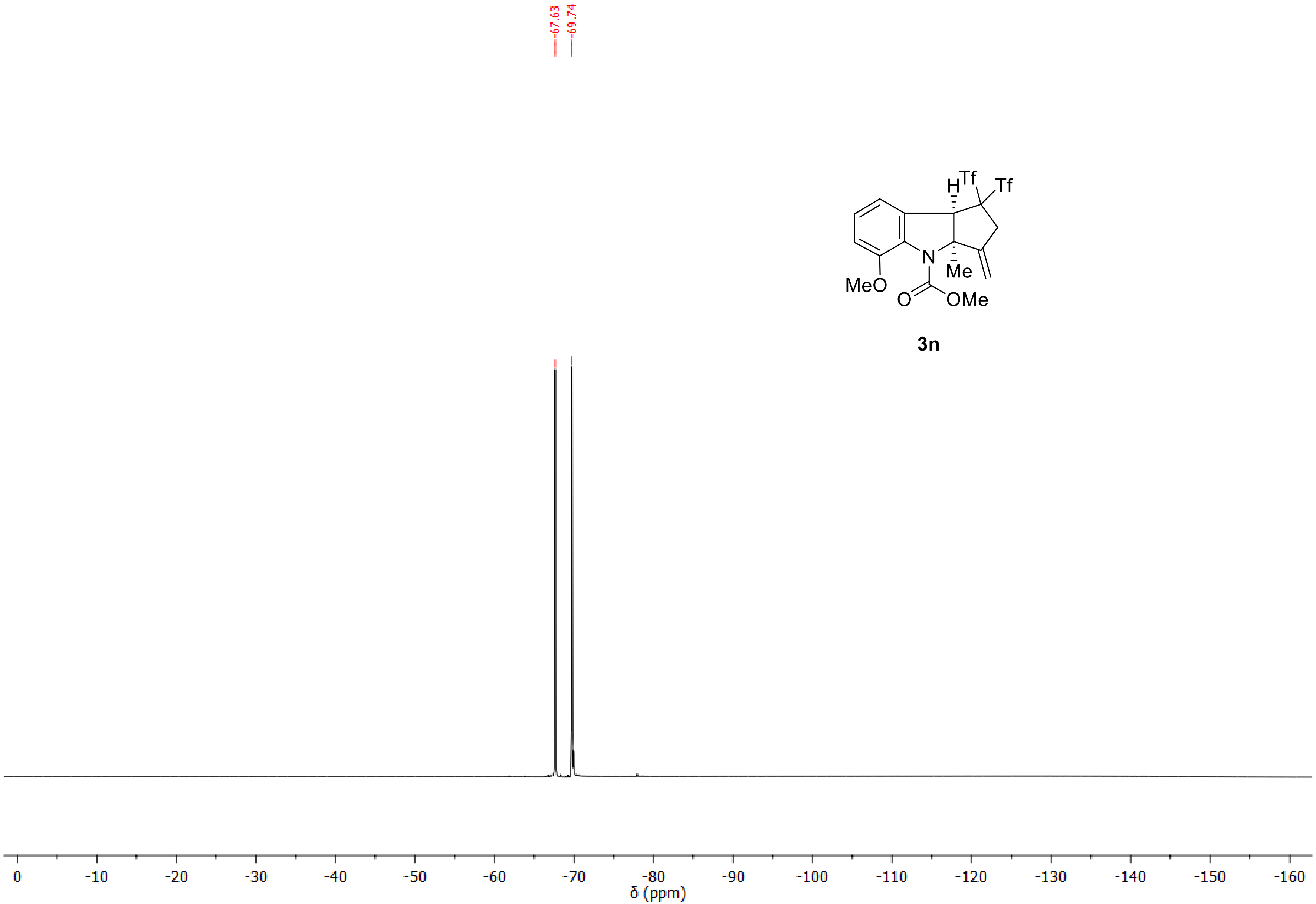

^{1}H NMR (500 MHz, $CDCl_3$)

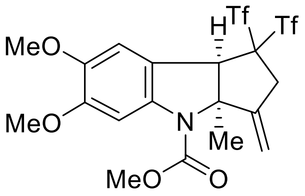

3o

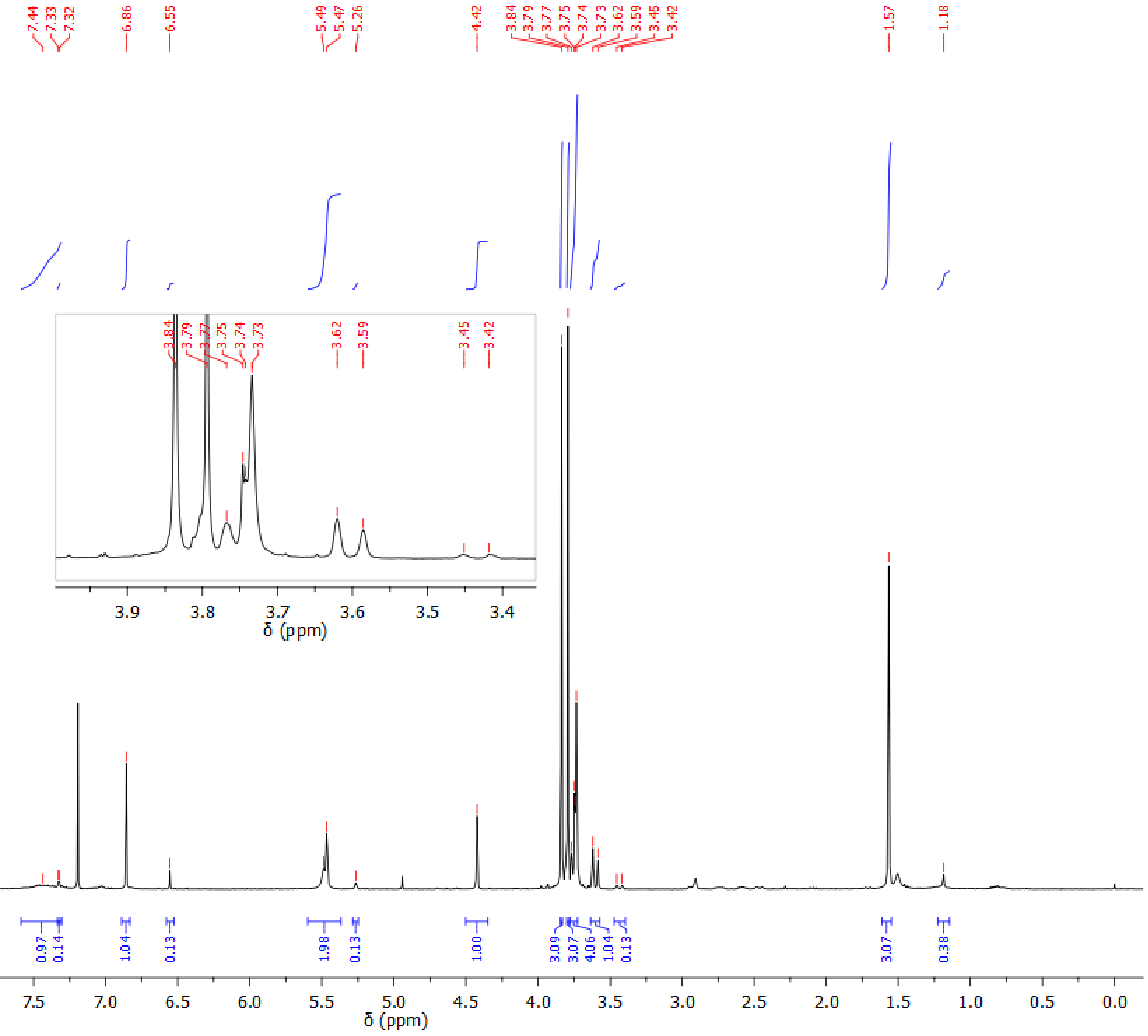

^{13}C NMR (125 MHz, $CDCl_3$)

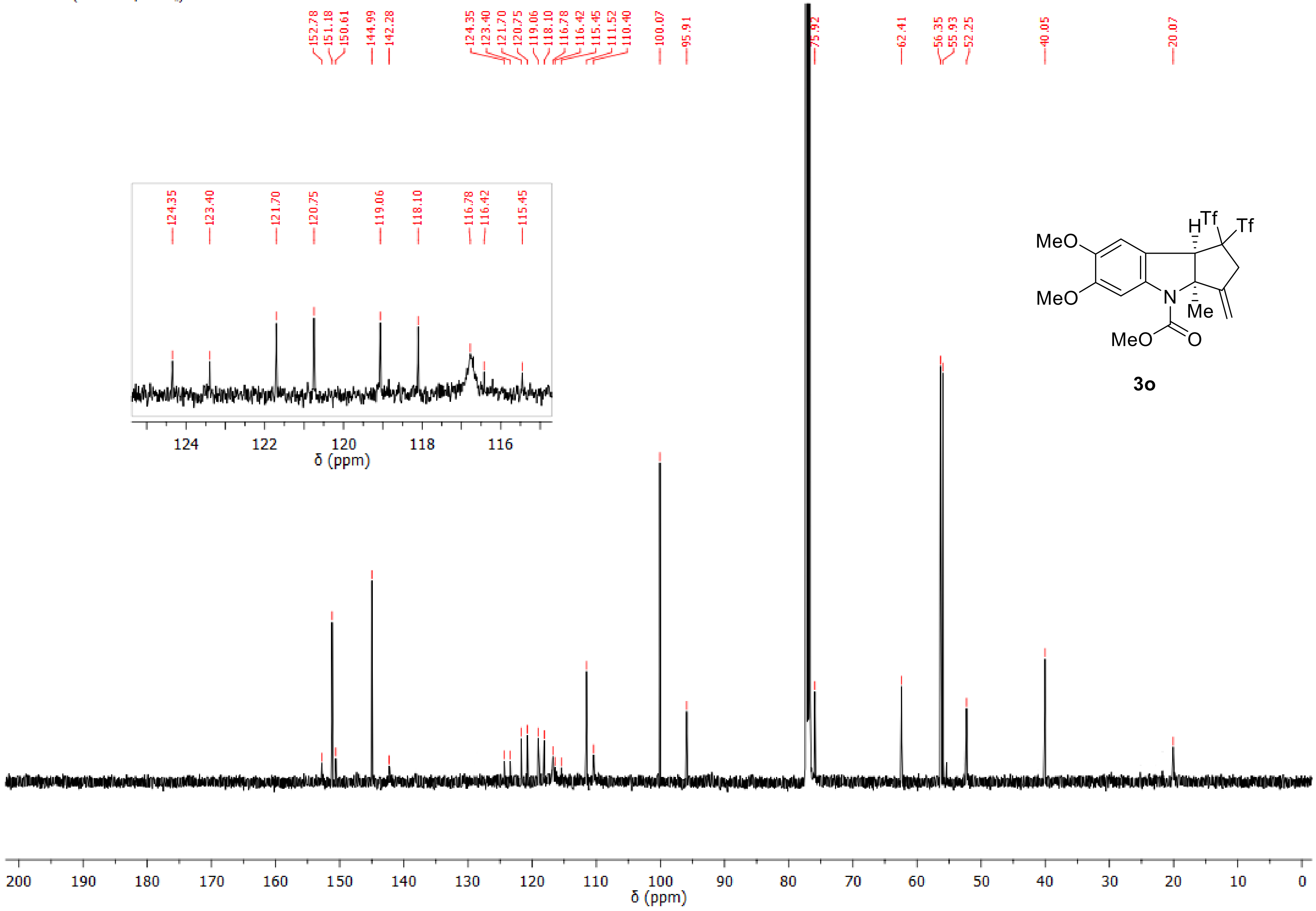

3o

^{19}F NMR (282 MHz, CDCl$_3$)

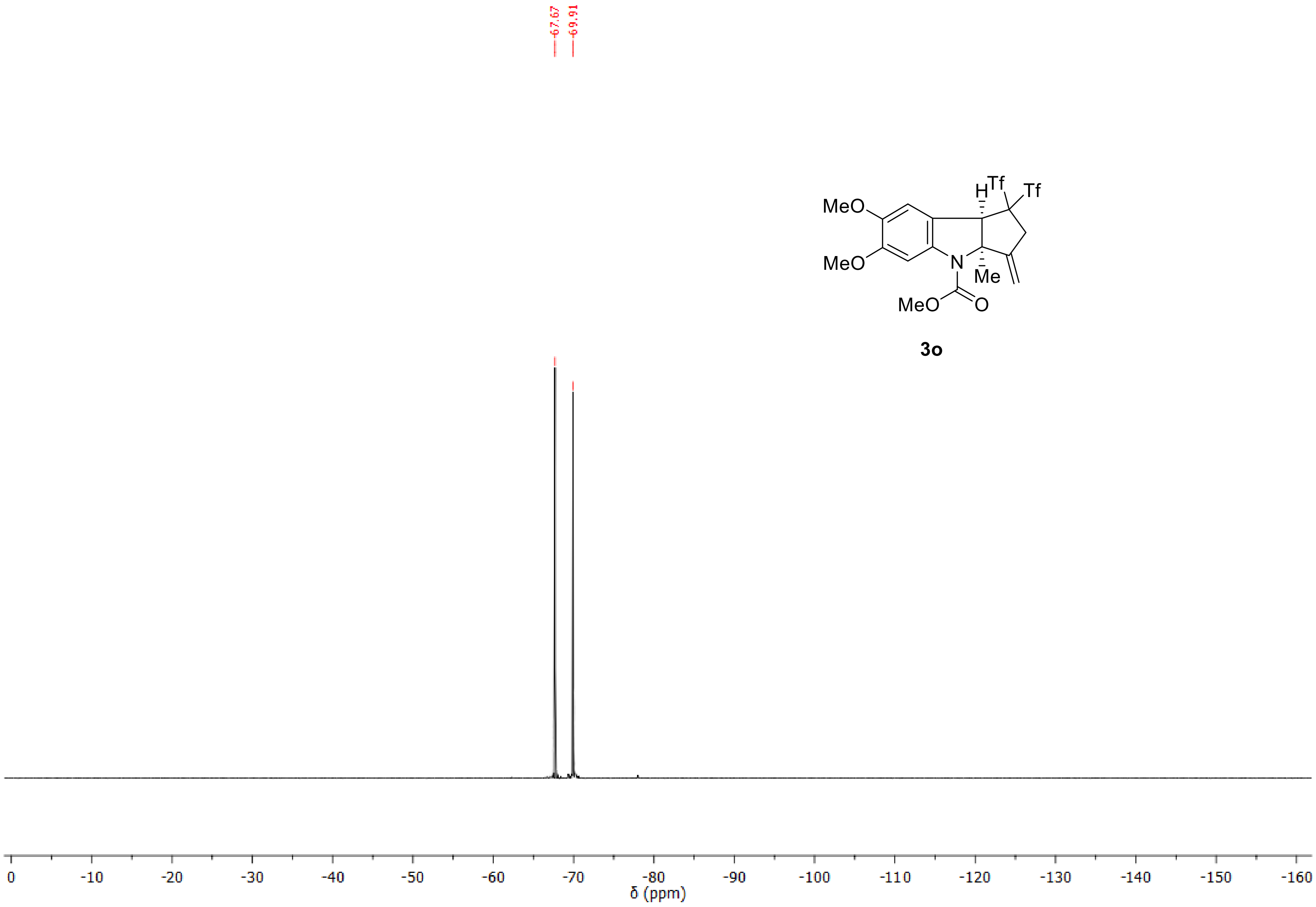

3o

^1H NMR (500 MHz, $CDCl_3$)

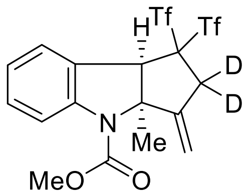

[D]-3a

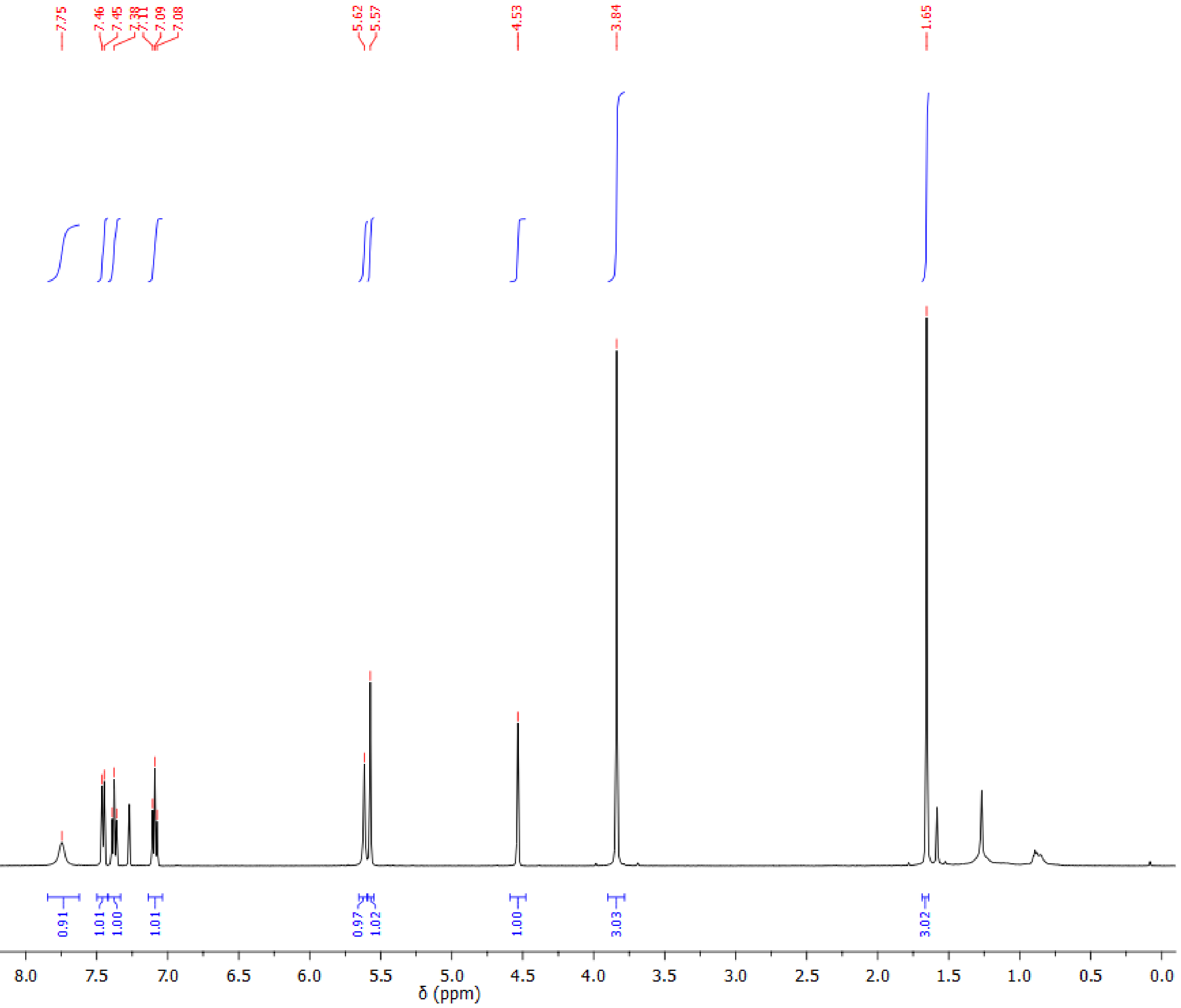

^{13}C NMR (125 MHz, $CDCl_3$)

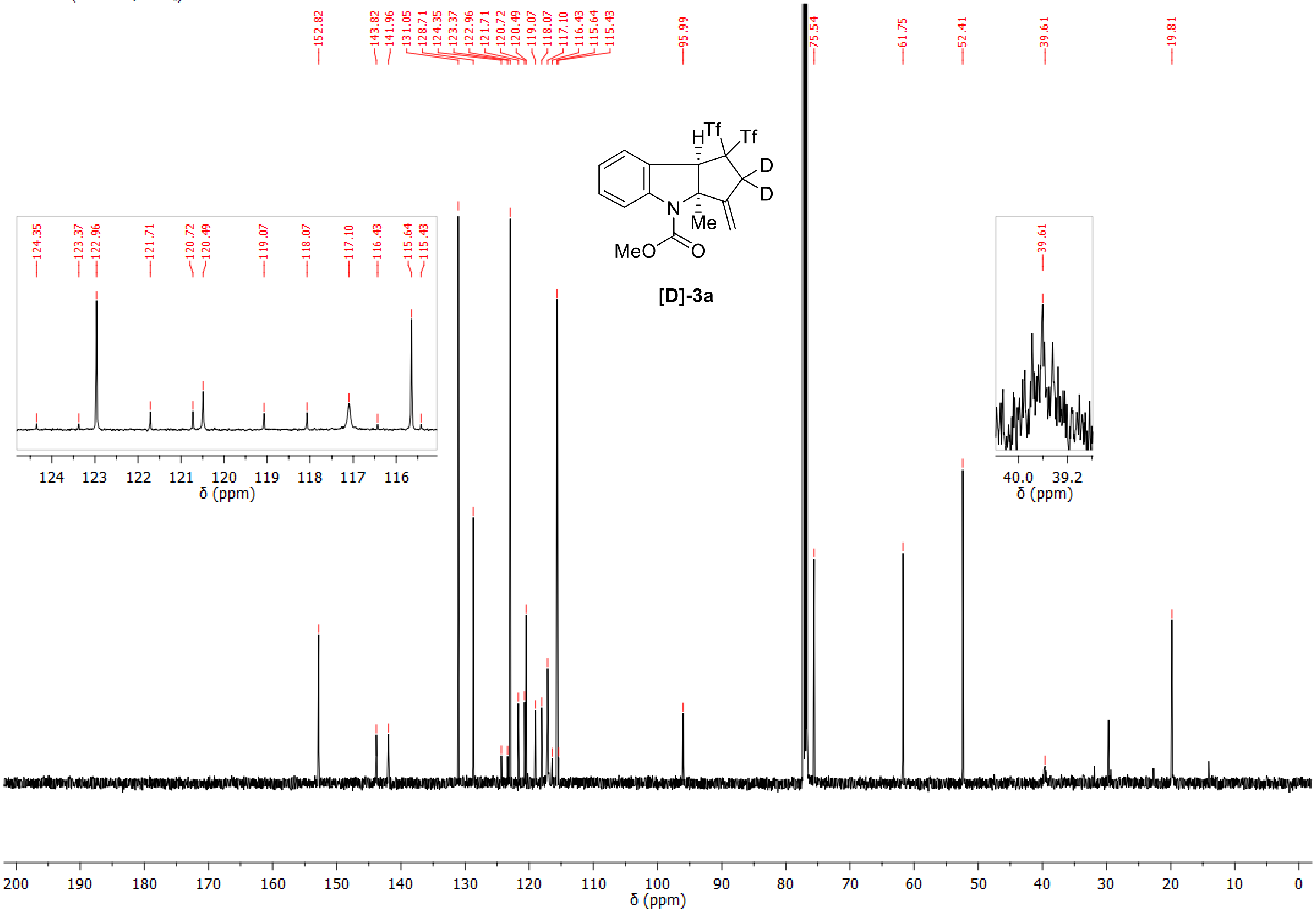

^{19}F NMR (282 MHz, $CDCl_3$)

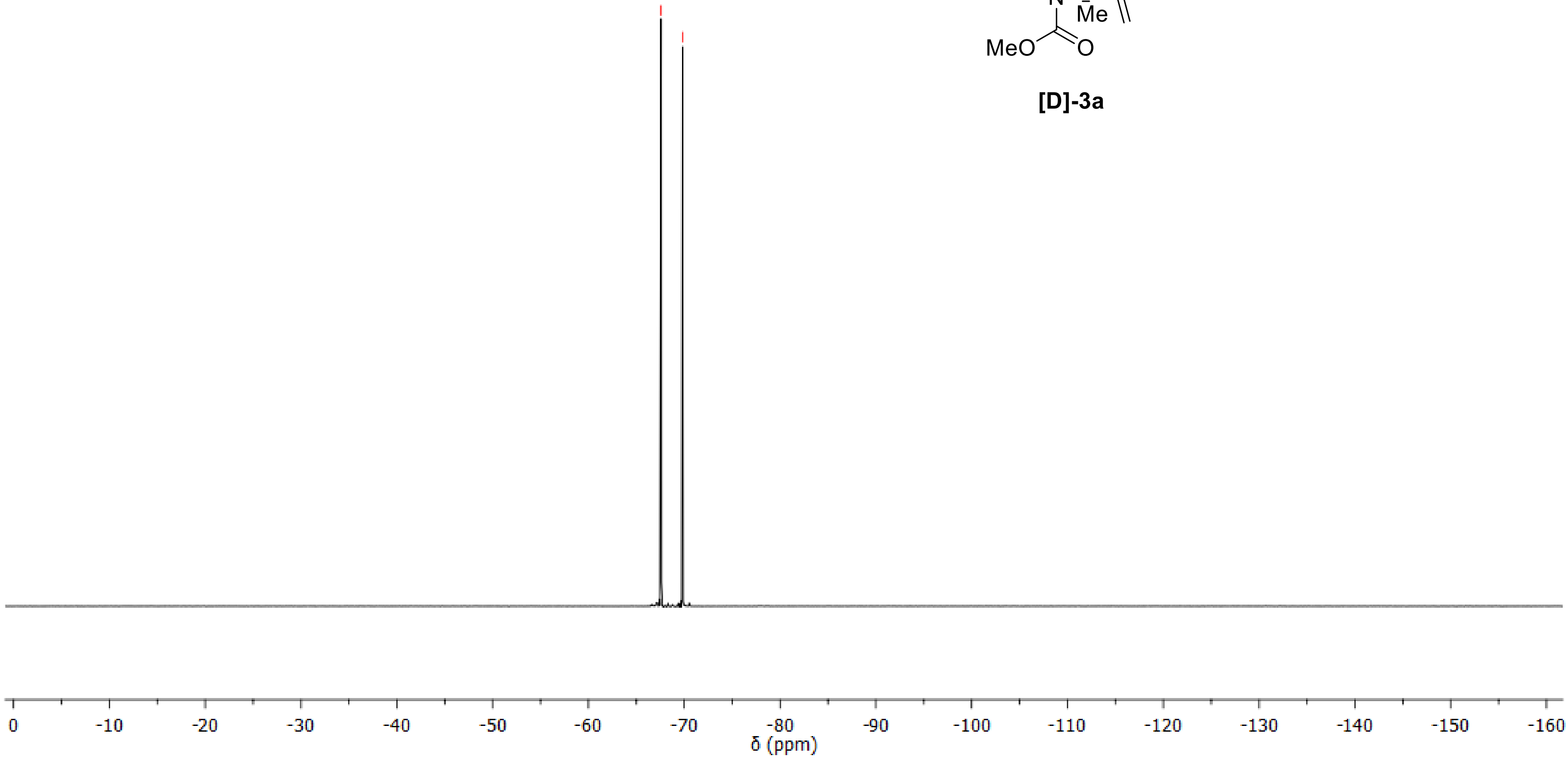

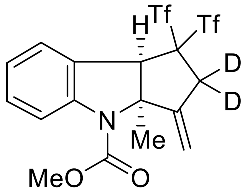

[D]-3a

D(^{2}H) NMR (107 MHz, $CDCl_3$)

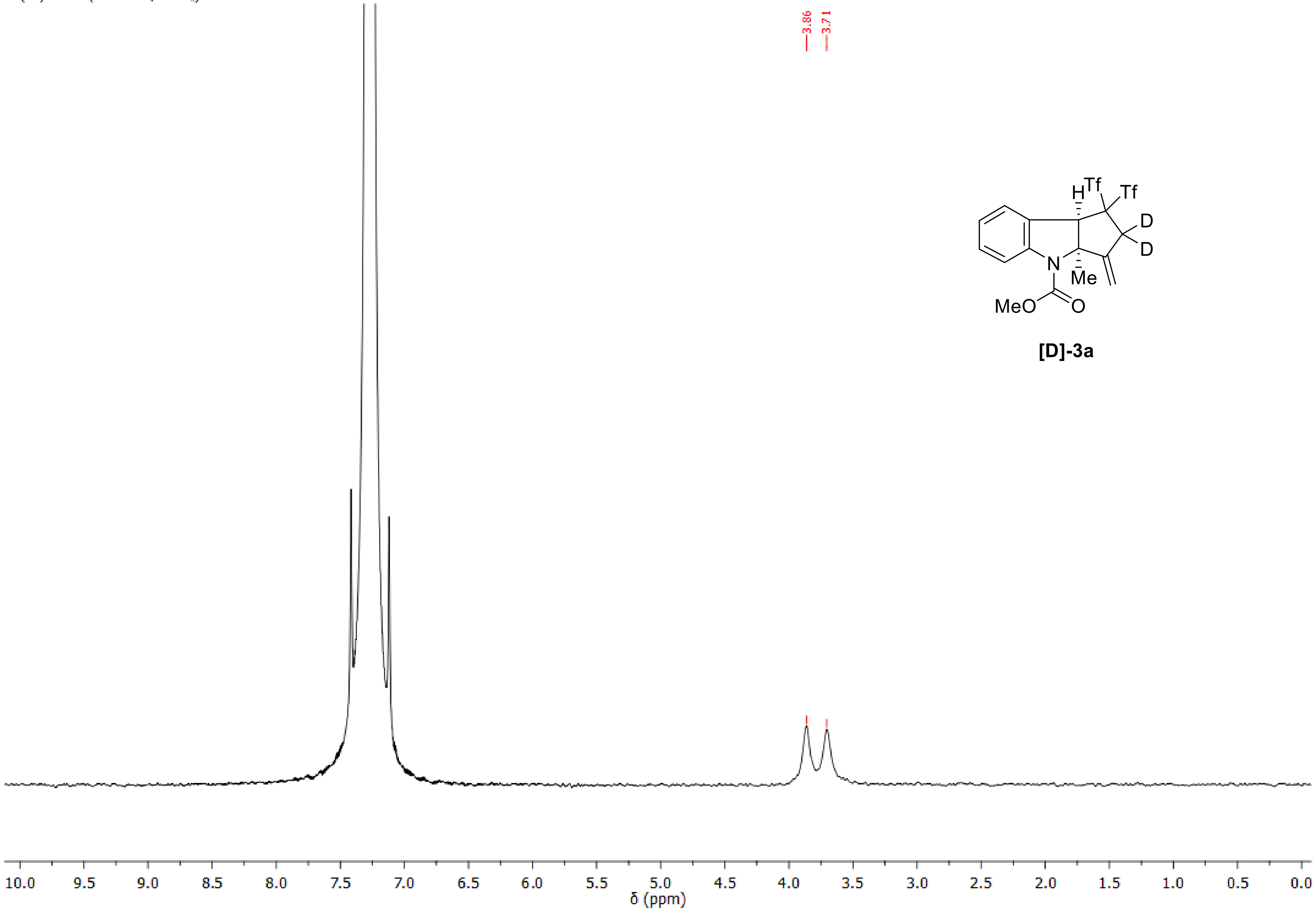

[D]-3a

^{1}H NMR (300 MHz, $CDCl_3$)

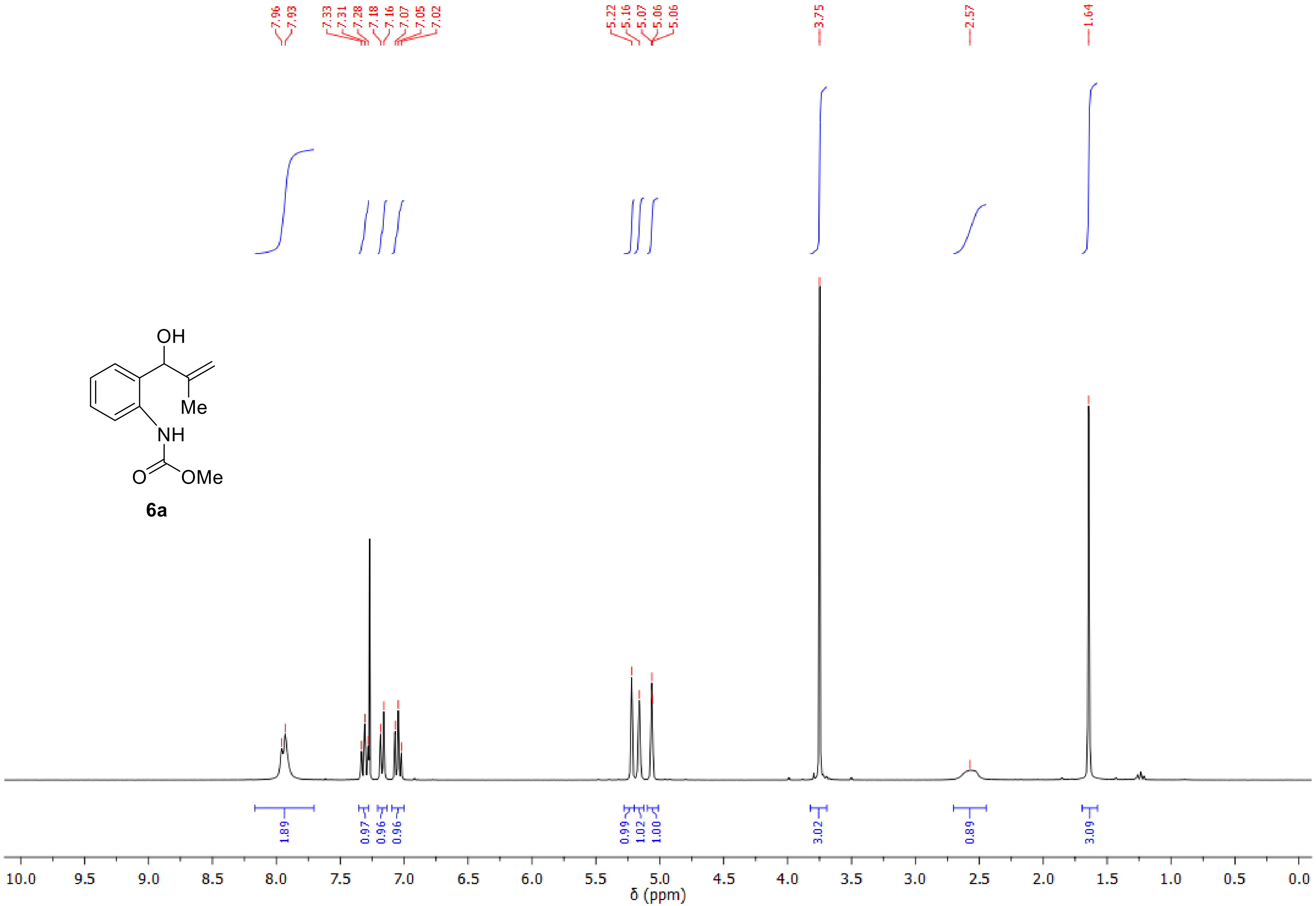

^{13}C NMR (75 MHz, $CDCl_3$)

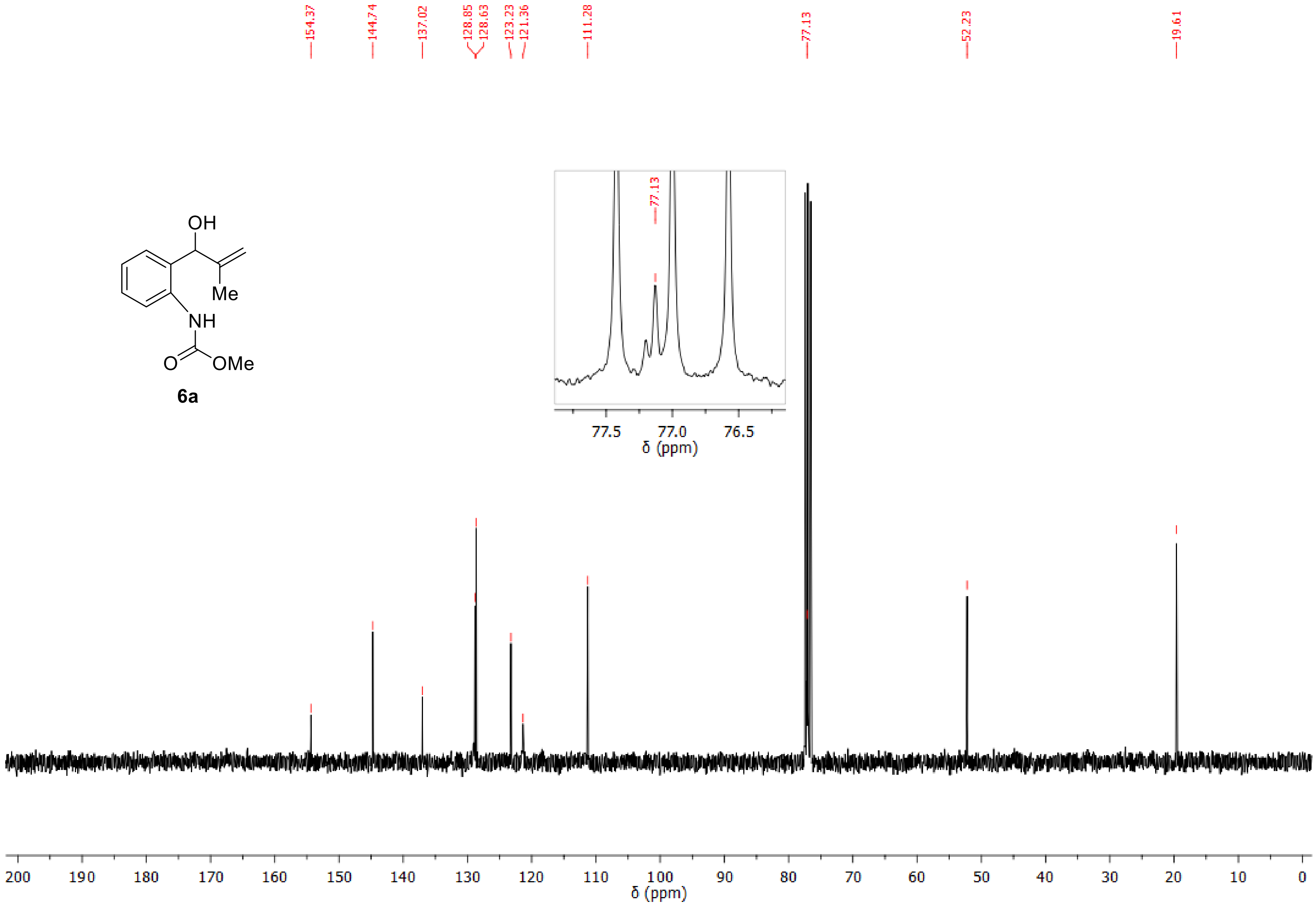

^{1}H NMR (300 MHz, $CDCl_3$)

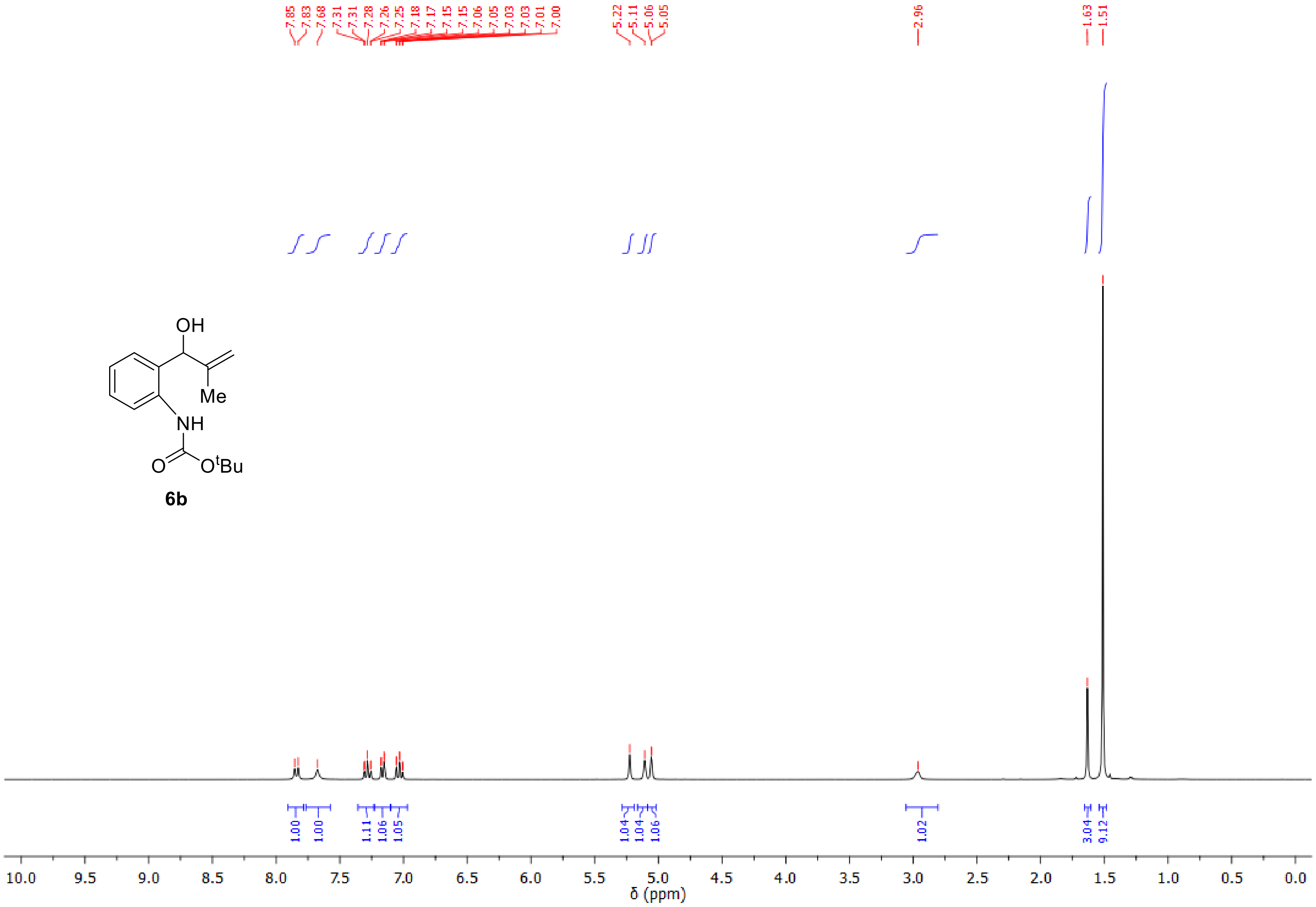

^{13}C NMR (75 MHz, CDCl$_3$)

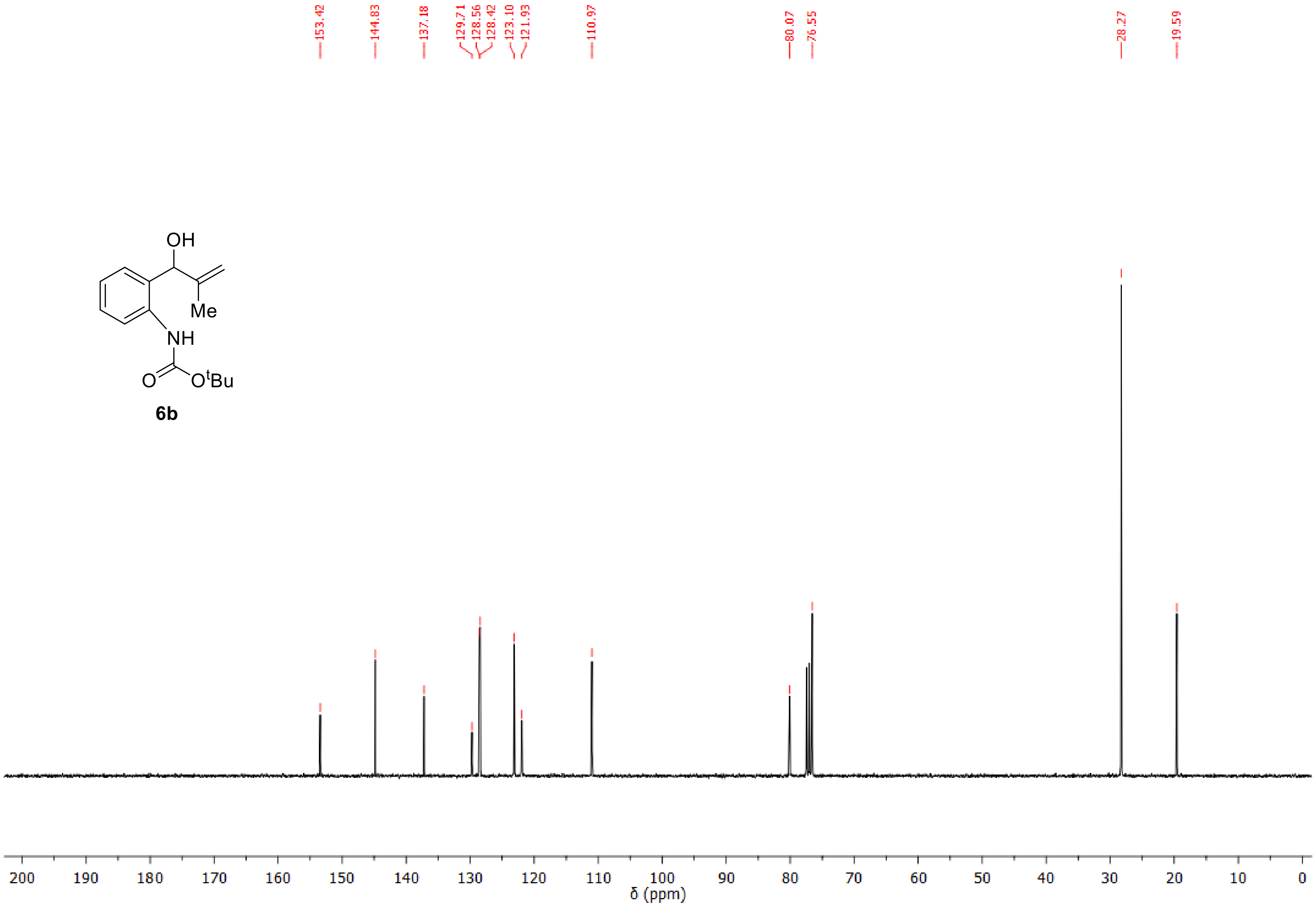

^{1}H NMR (300 MHz, $CDCl_3$)

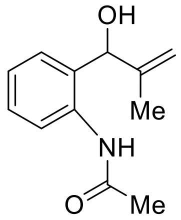

6c

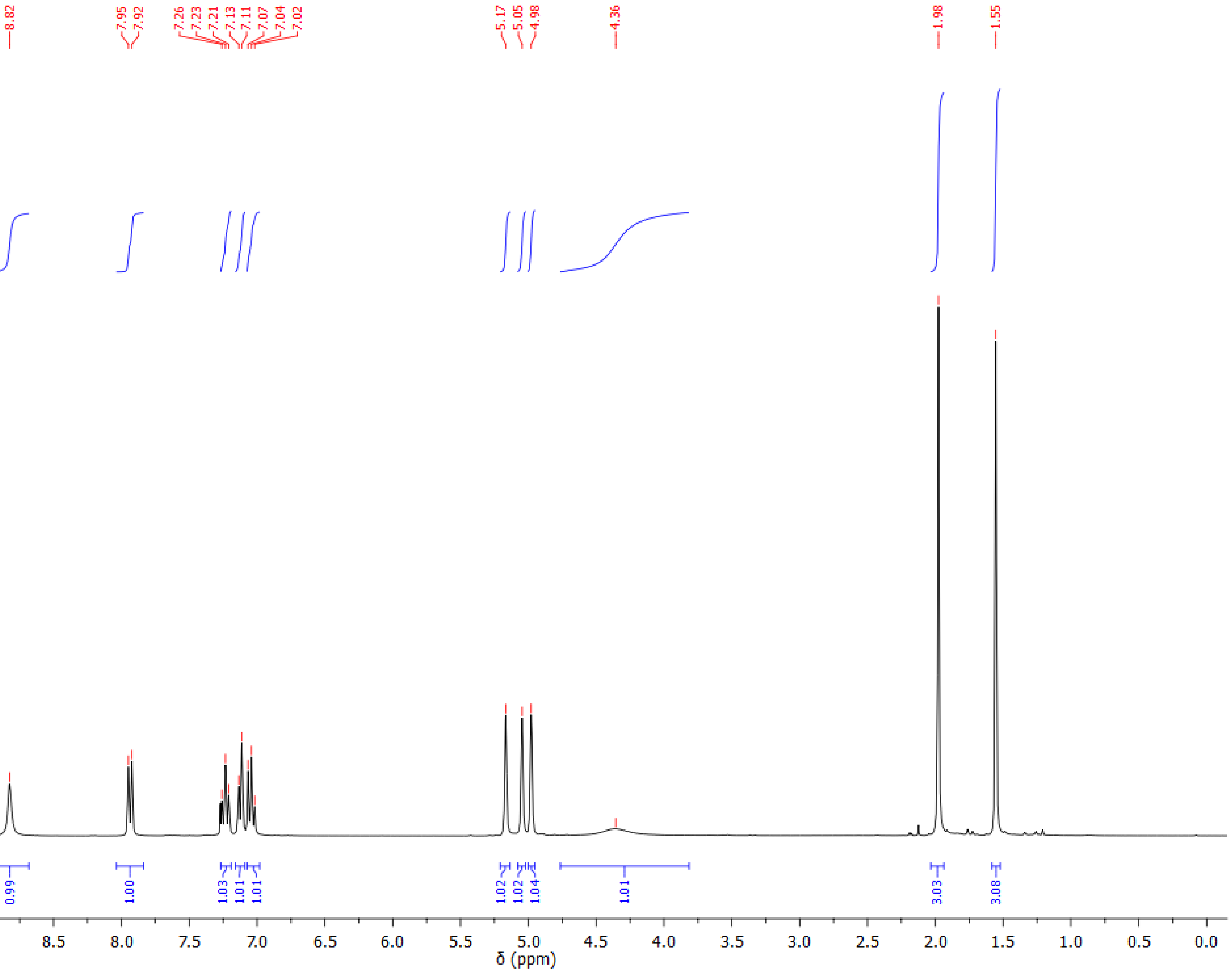

^{13}C NMR (75 MHz, $CDCl_3$)

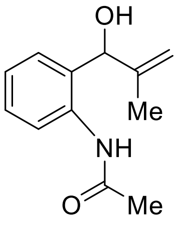

6c

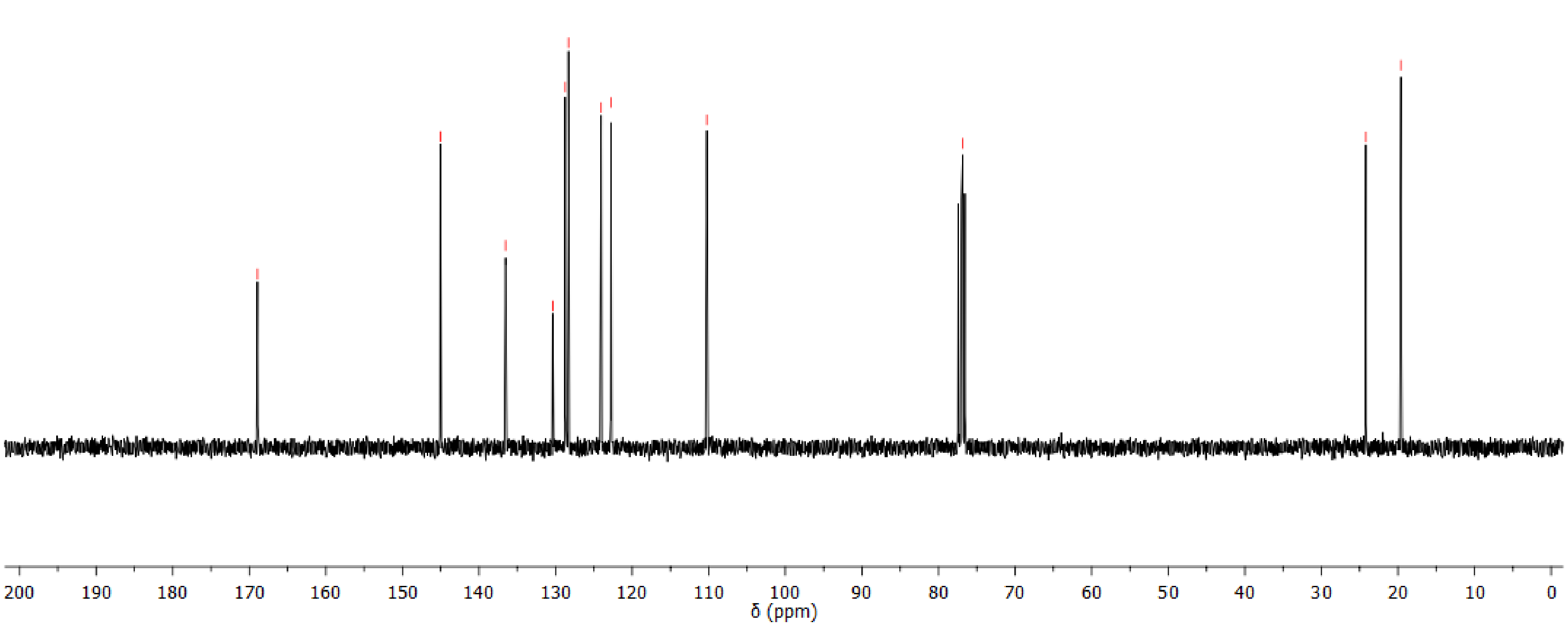

^{1}H NMR (300 MHz, $CDCl_3$)

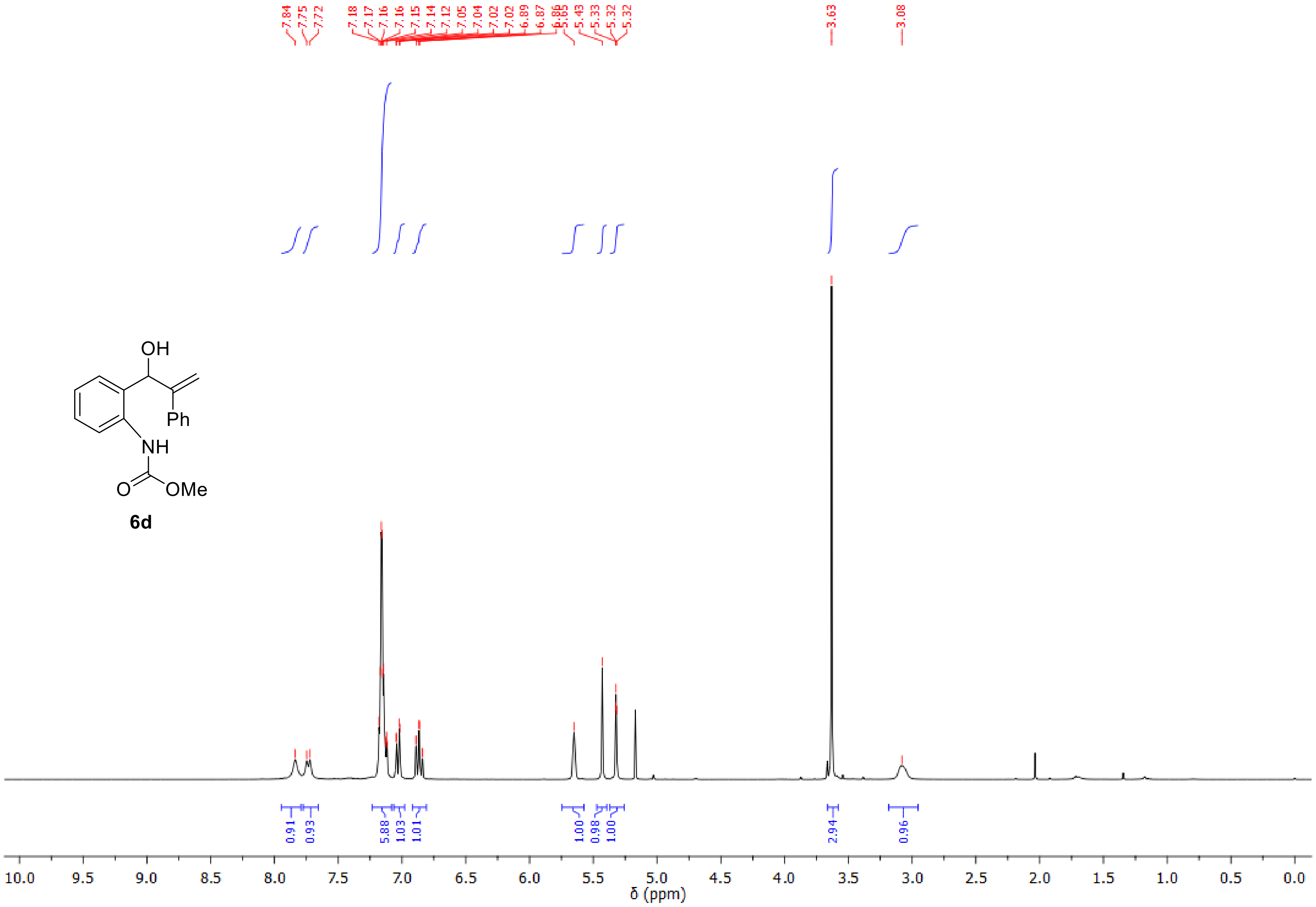

6d

^{13}C NMR (75 MHz, $CDCl_3$)

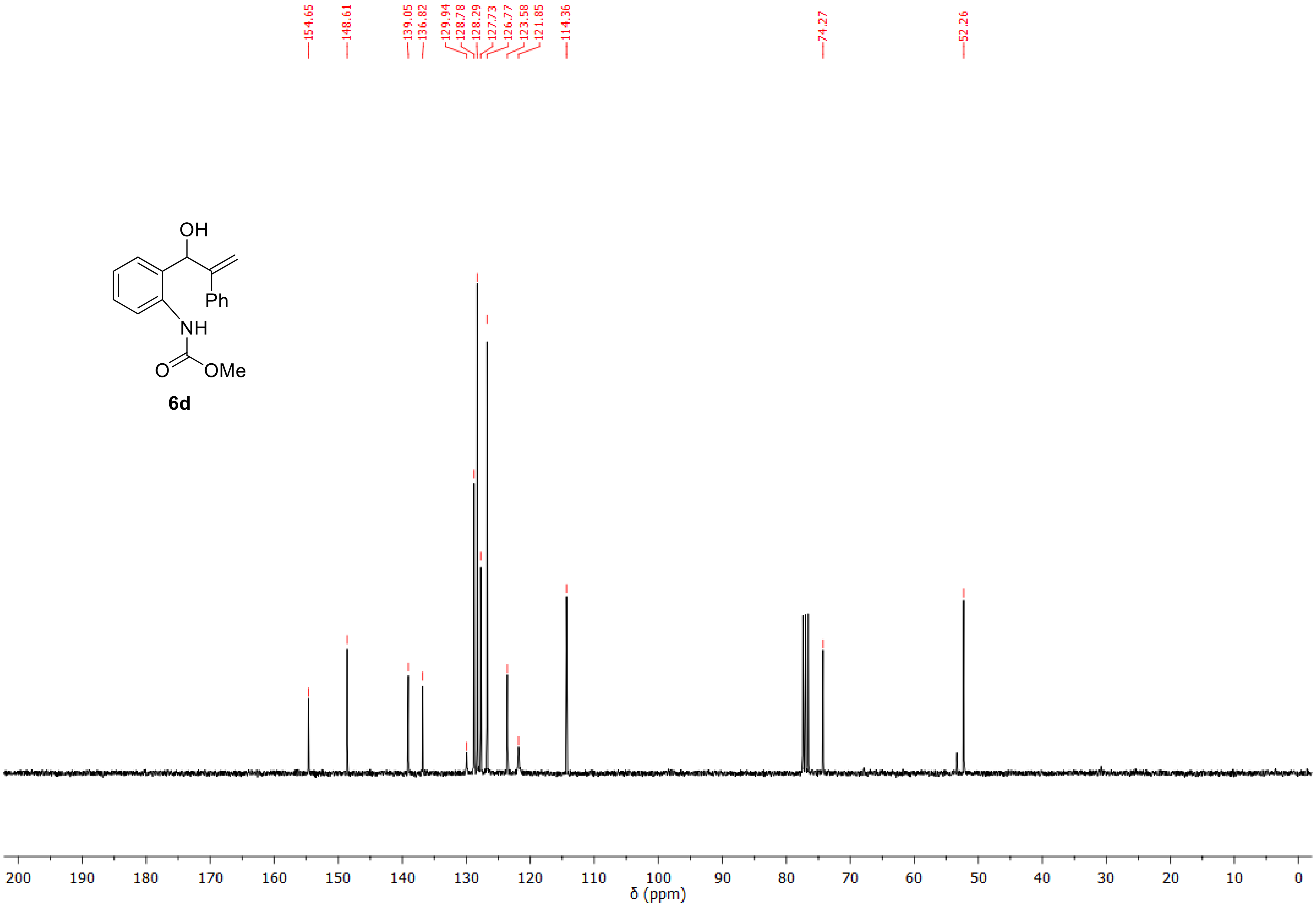

6d

^{1}H NMR (300 MHz, $CDCl_3$)

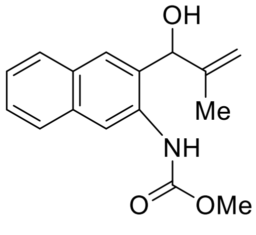

6e

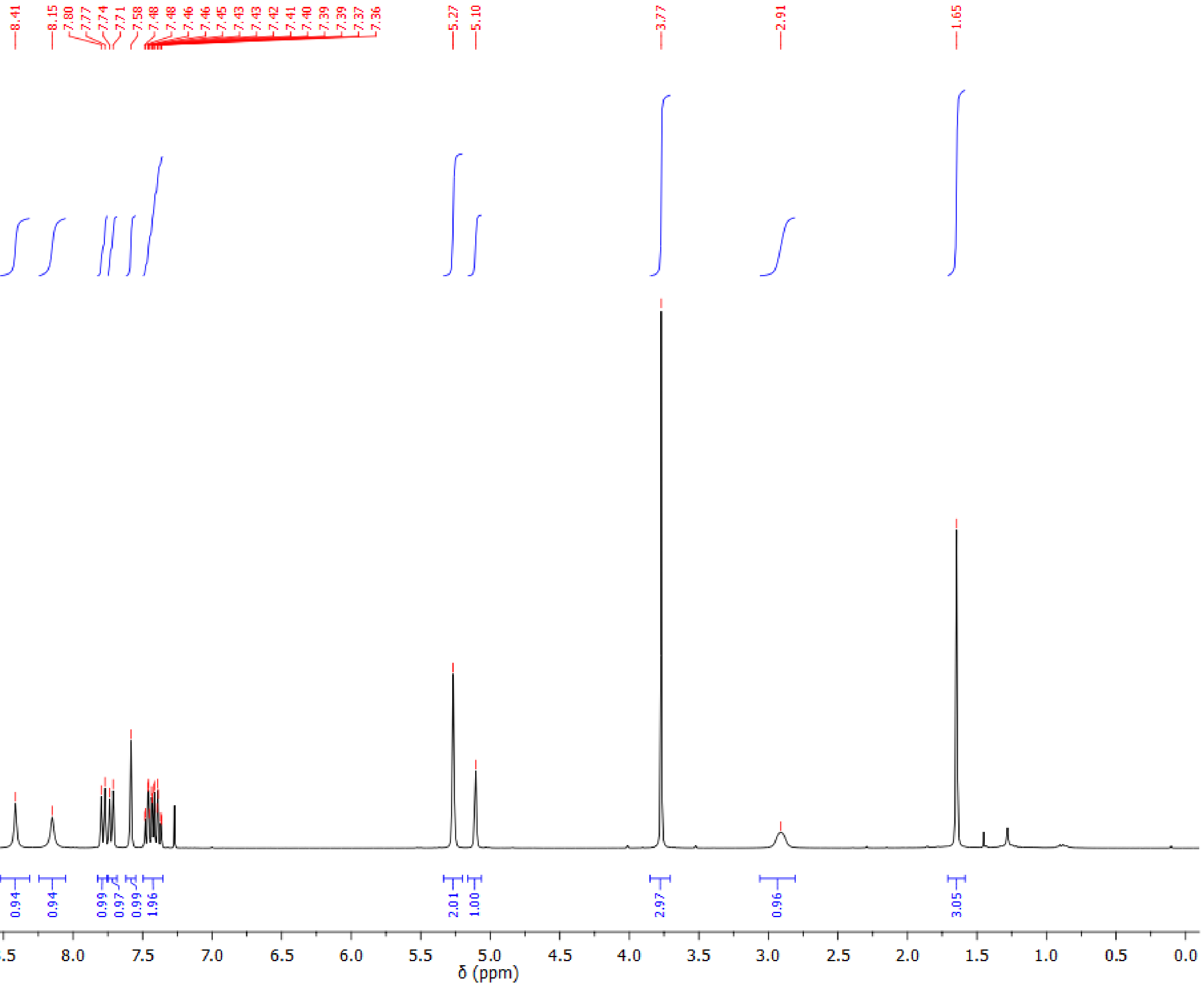

^{13}C NMR (75 MHz, $CDCl_3$)

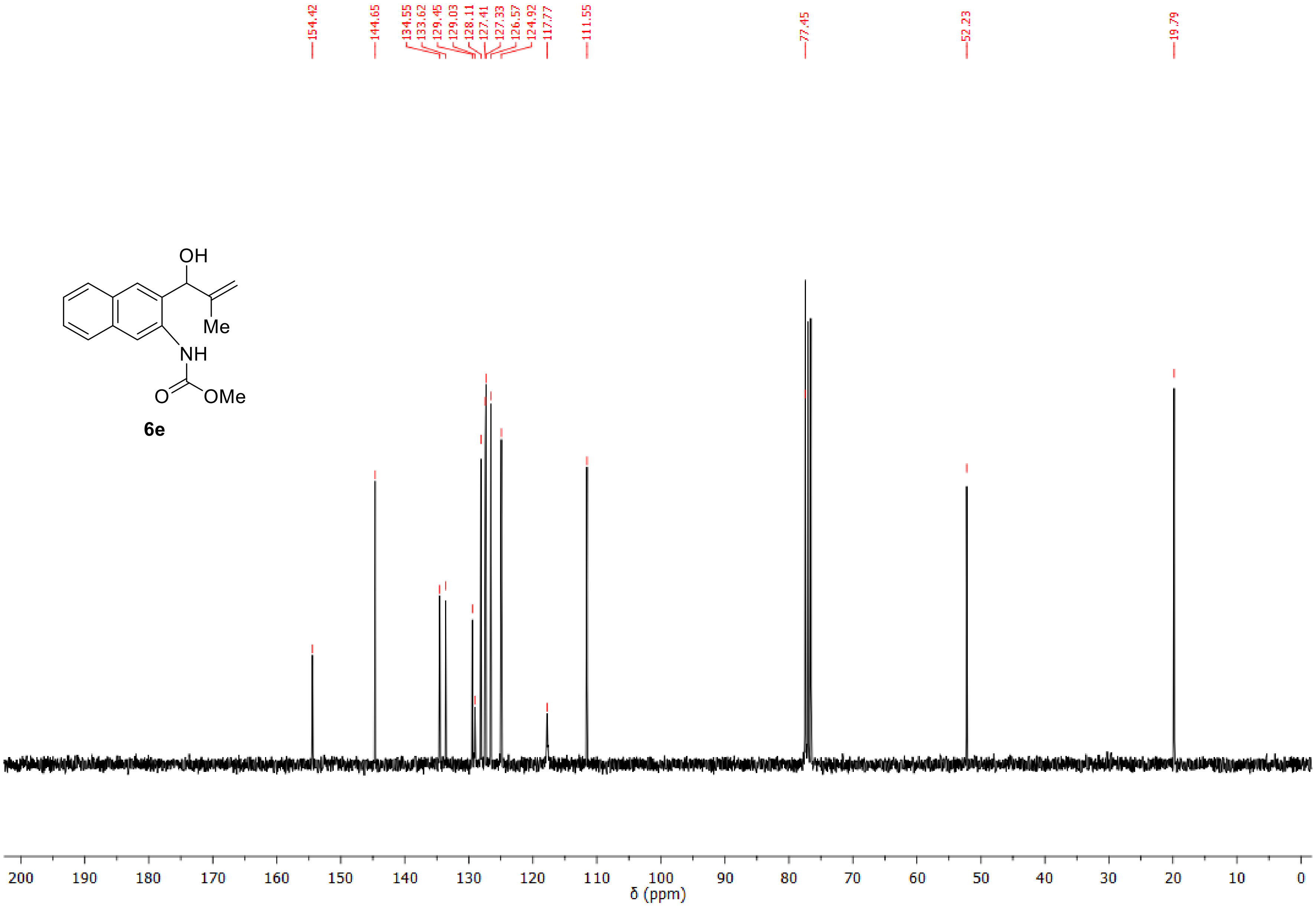

1H NMR (300 MHz, CDCl3)

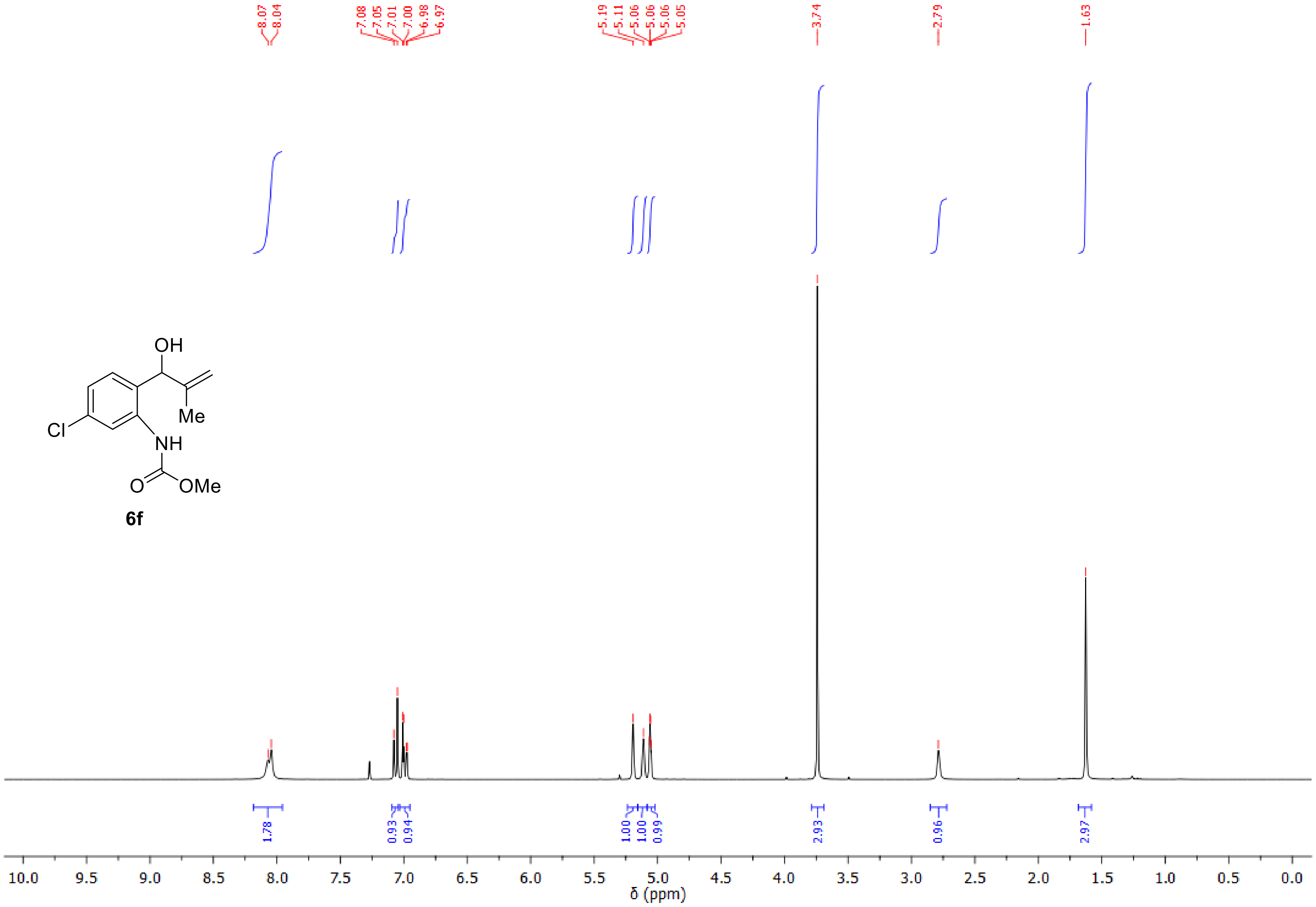

^{13}C NMR (75 MHz, $CDCl_3$)

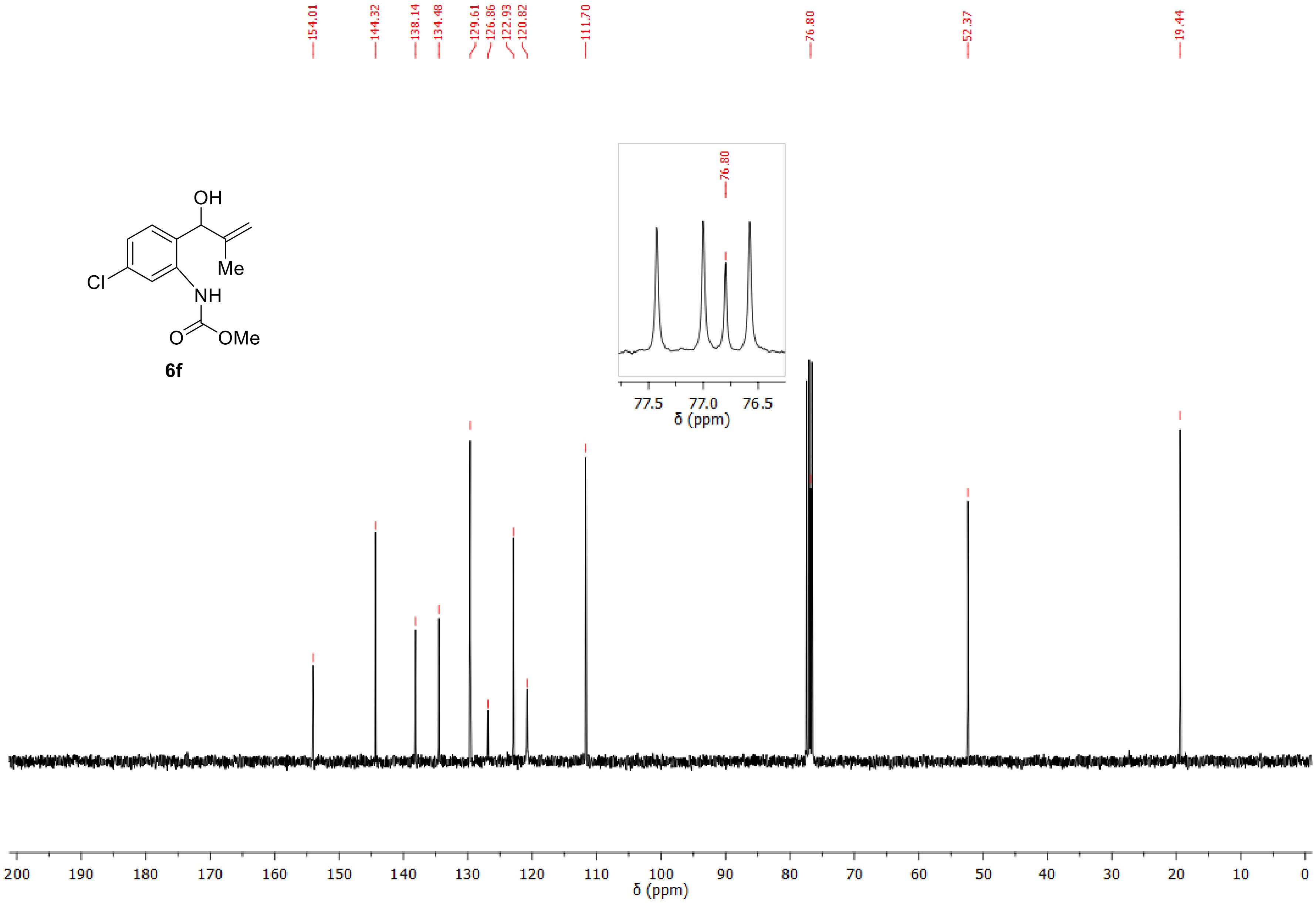

^{1}H NMR (300 MHz, CDCl$_3$)

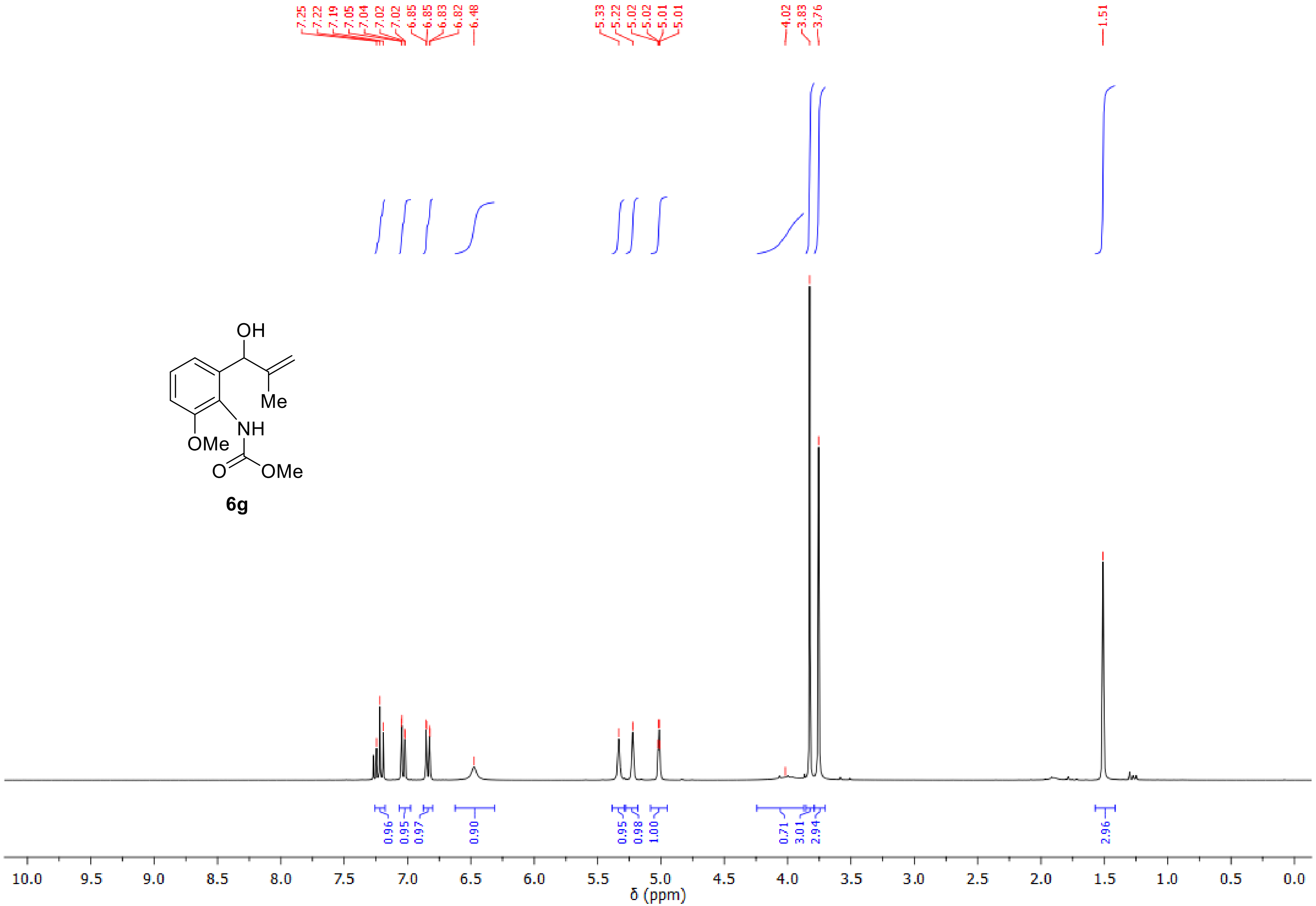

6g

^{13}C NMR (75 MHz, $CDCl_3$)

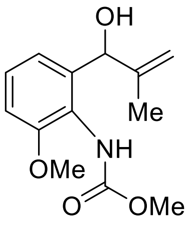

6g

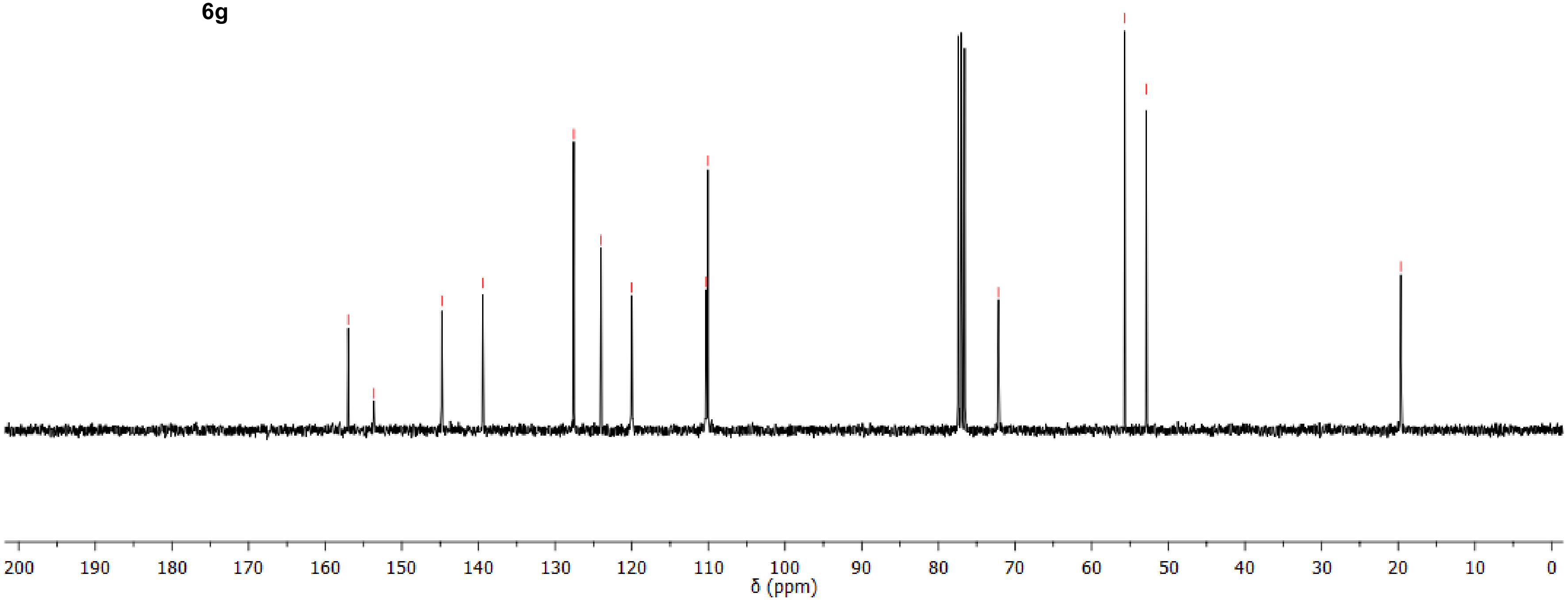

^{1}H NMR (500 MHz, $CDCl_3$)

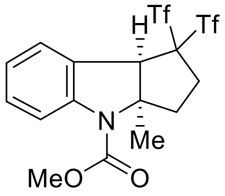

7a

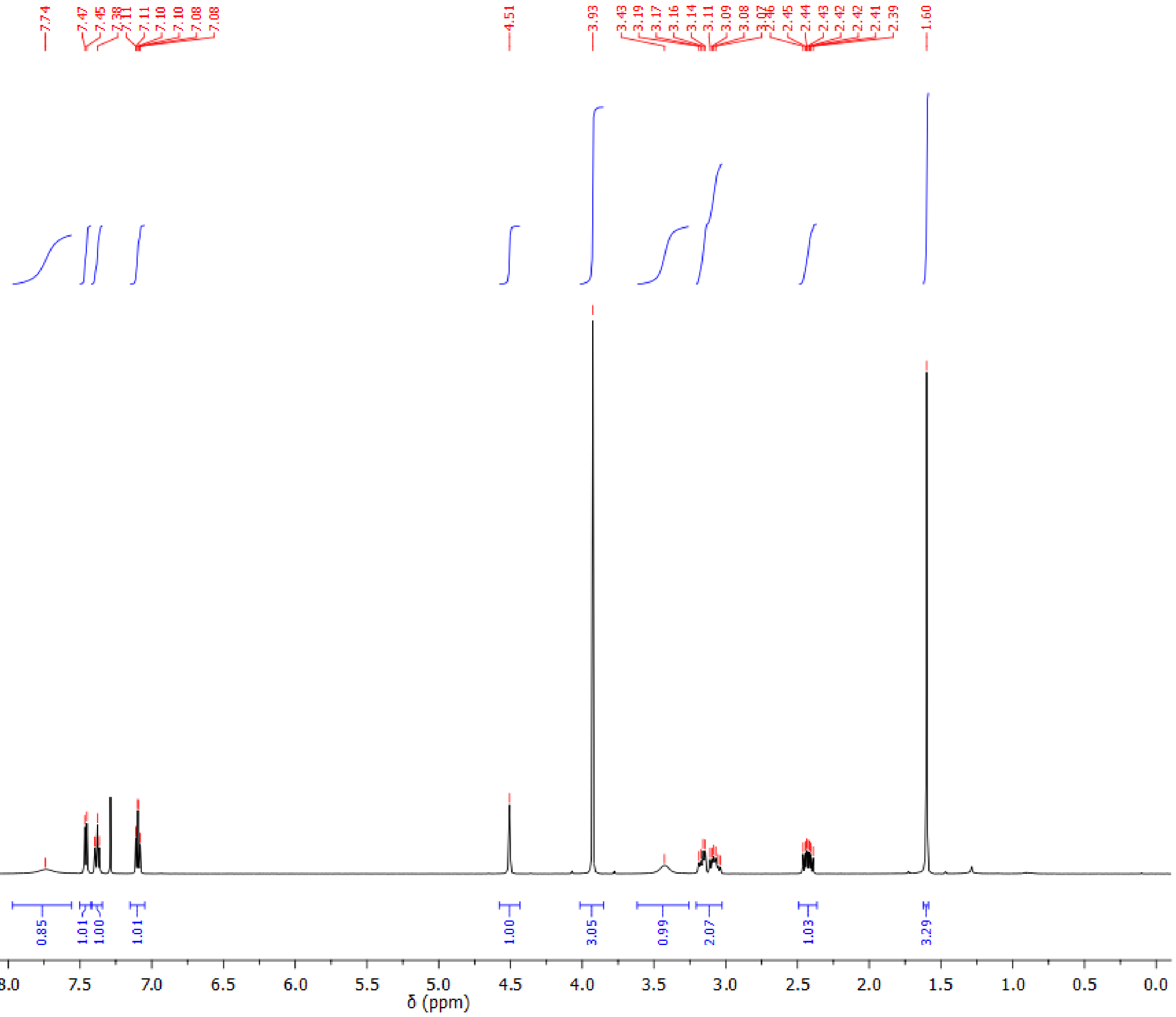

13C NMR (125 MHz, CDCl3)

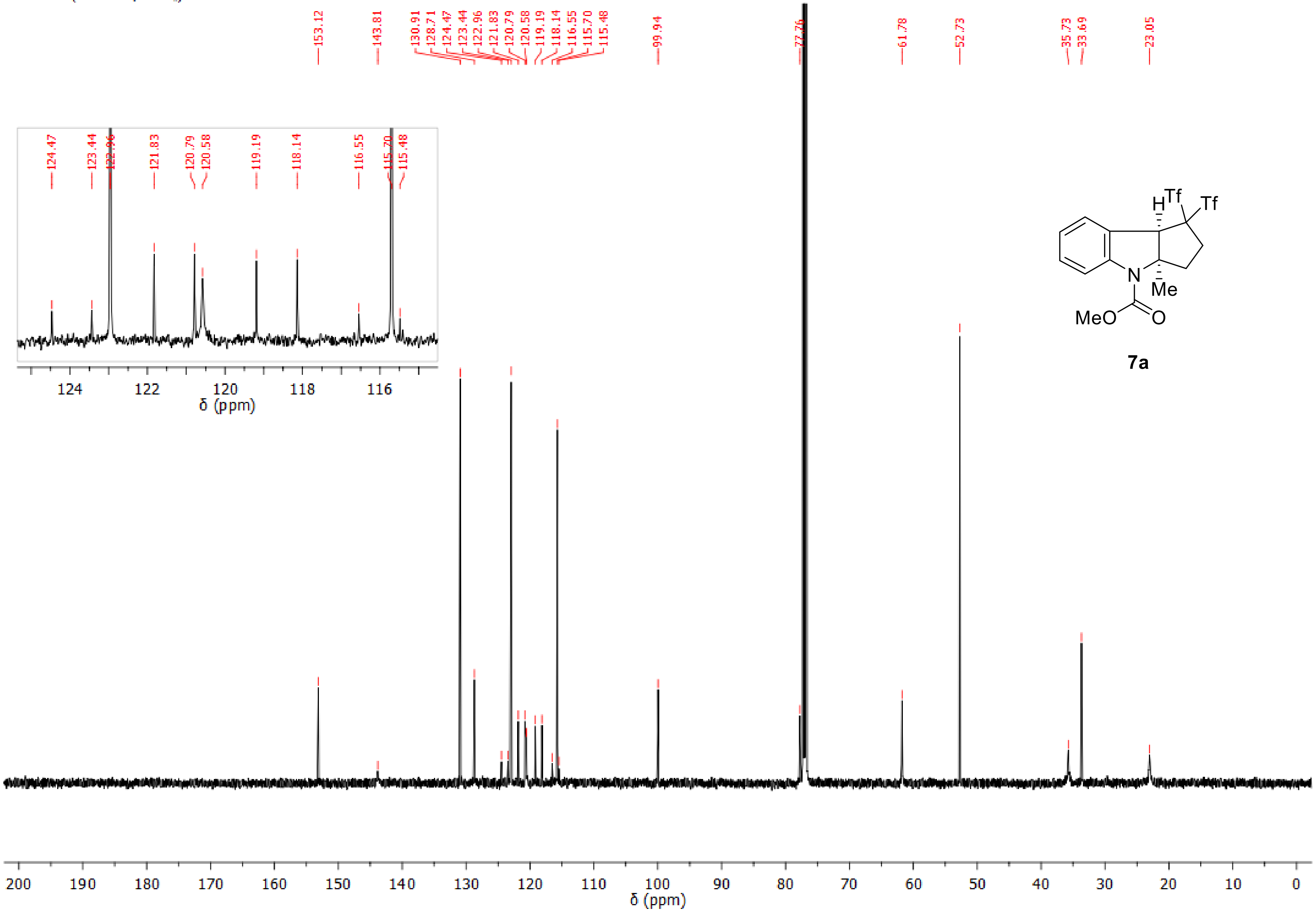

^{19}F NMR (282 MHz, CDCl$_3$)

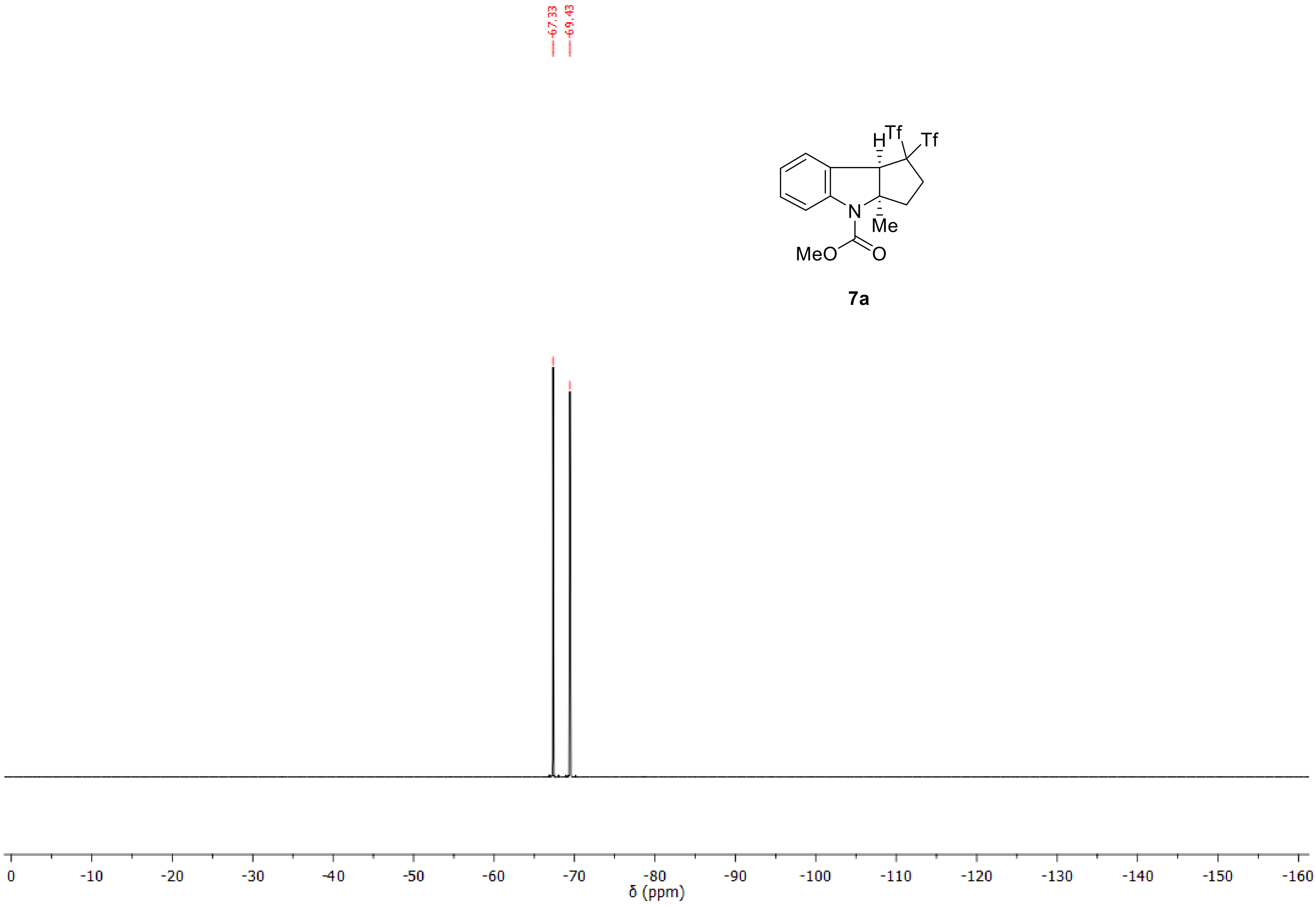

7a

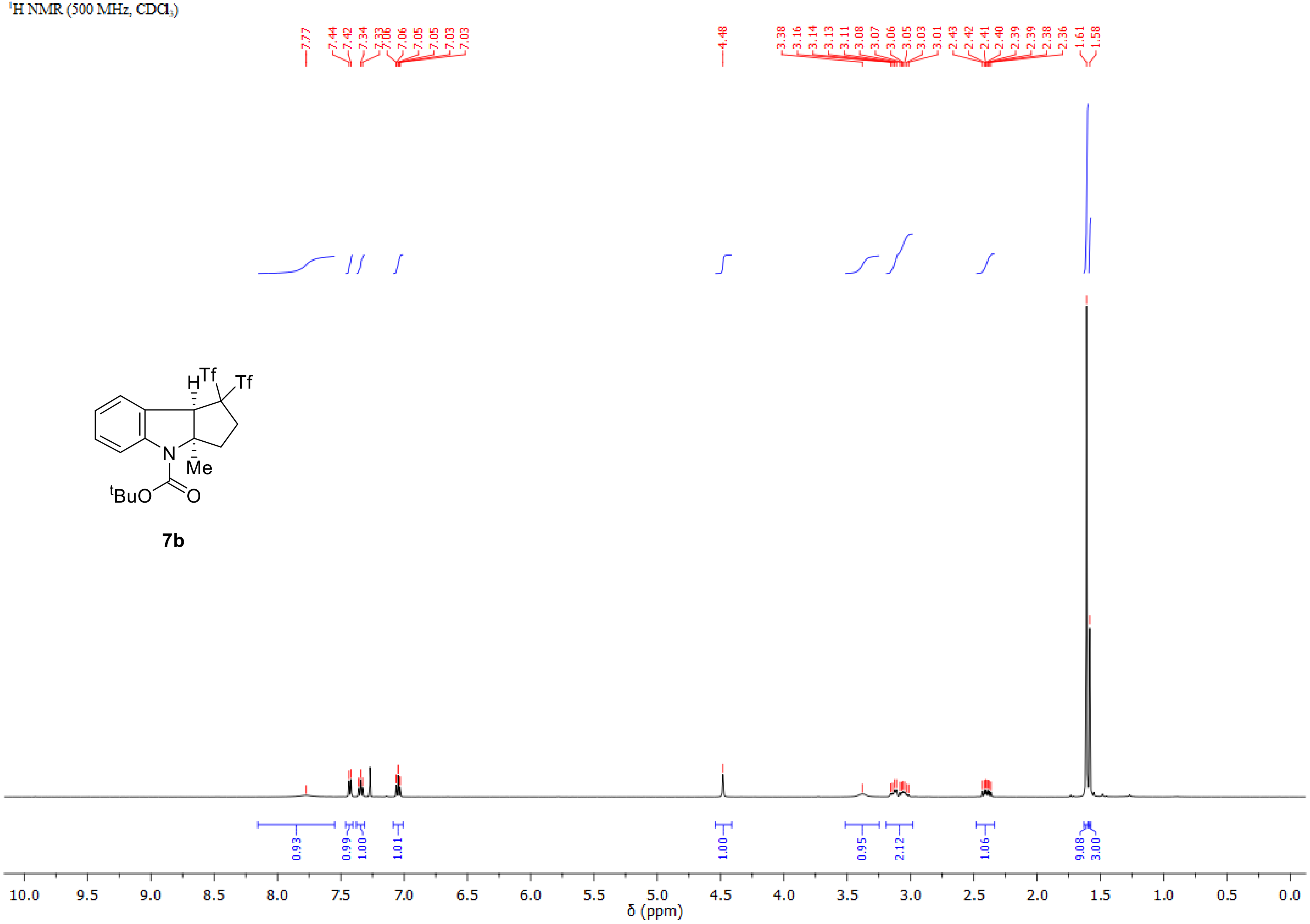

^{1}H NMR (500 MHz, CDCl$_3$)
H
Tf
Tf
N
Me
^{t}BuO
O
7b
δ (ppm)

^{13}C NMR (125 MHz, CDCl$_3$)

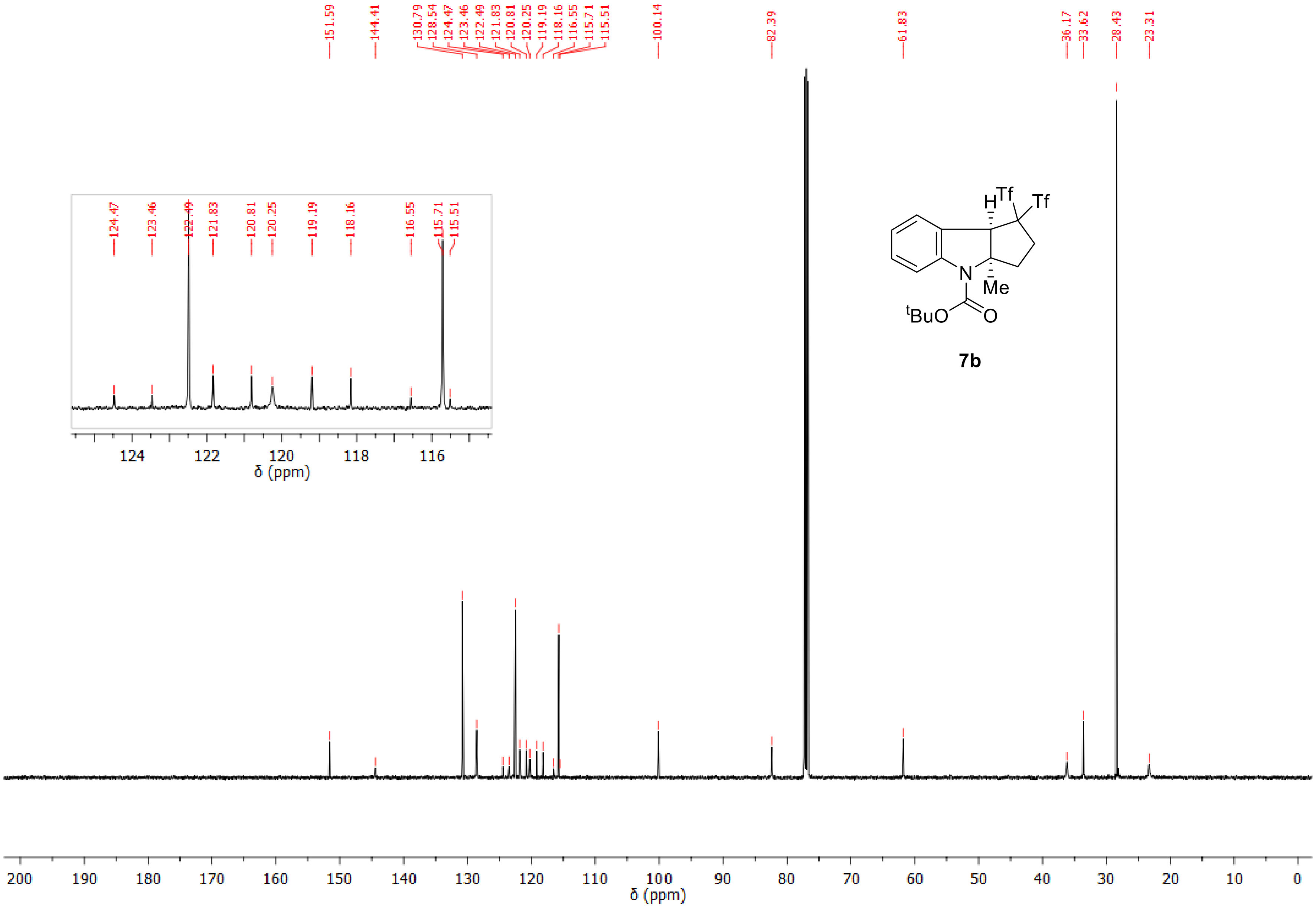

7b

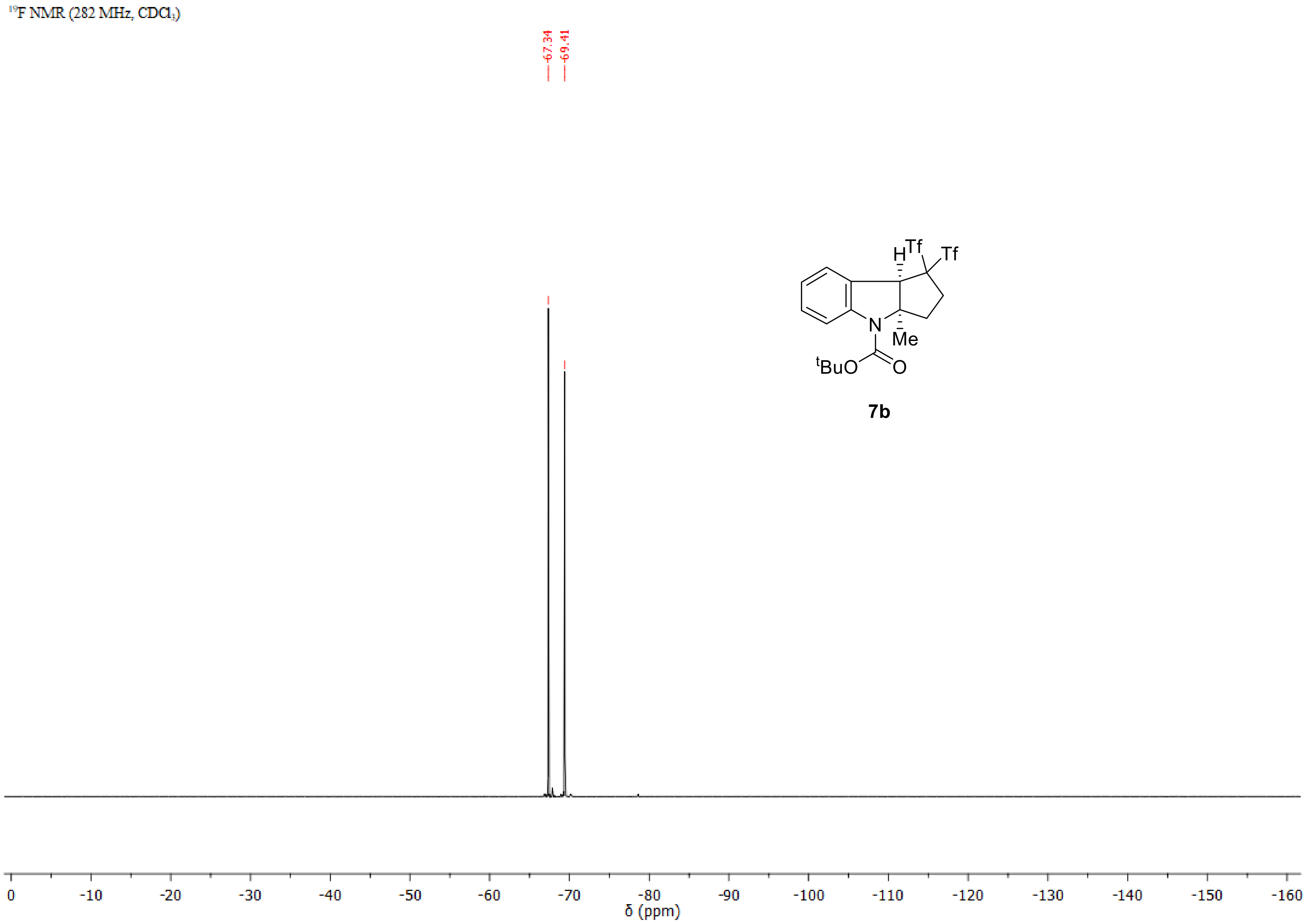
19F NMR (282 MHz, CDCl3)
-67.34
-69.41
0
-10
-20
-30
-40
-50
-60
-70
-80
-90
-100
-110
-120
-130
-140
-150
-160
δ (ppm)
H
Tf
Tf
N
Me
tBuO
O

7b

^{1}H NMR (500 MHz, CDCl$_3$)

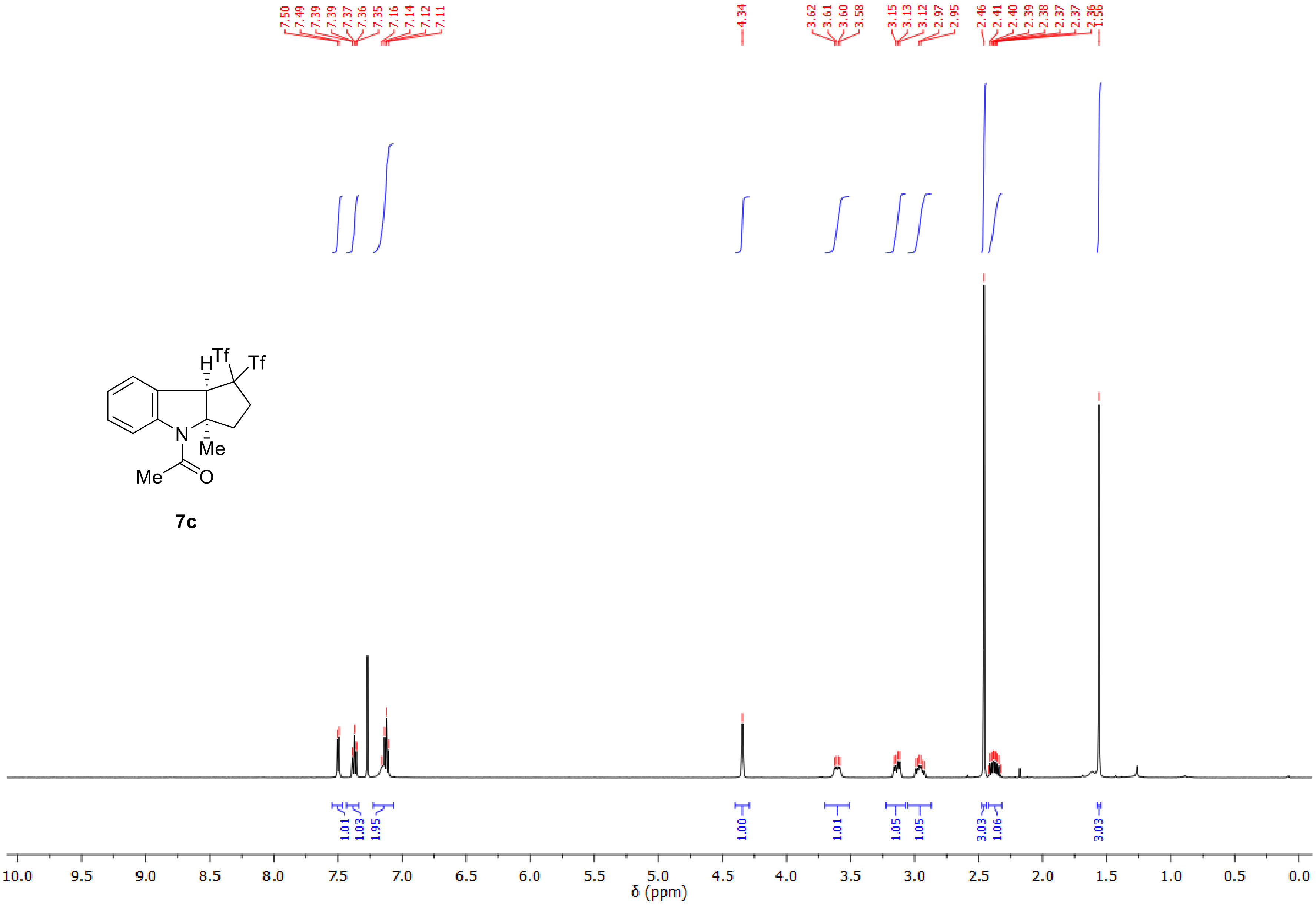

^{13}C NMR (125 MHz, $CDCl_3$)

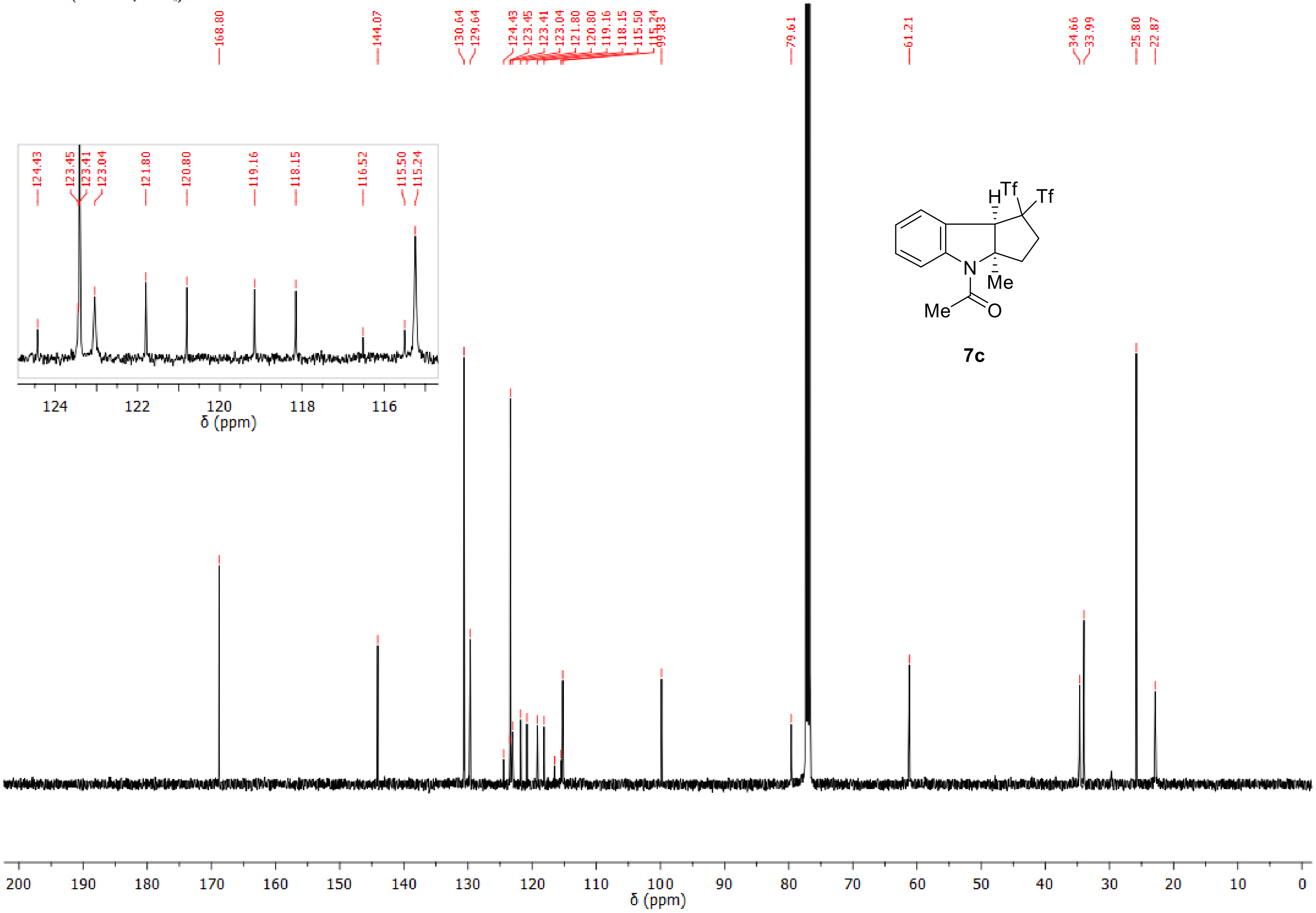

^{19}F NMR (282 MHz, CDCl$_3$)

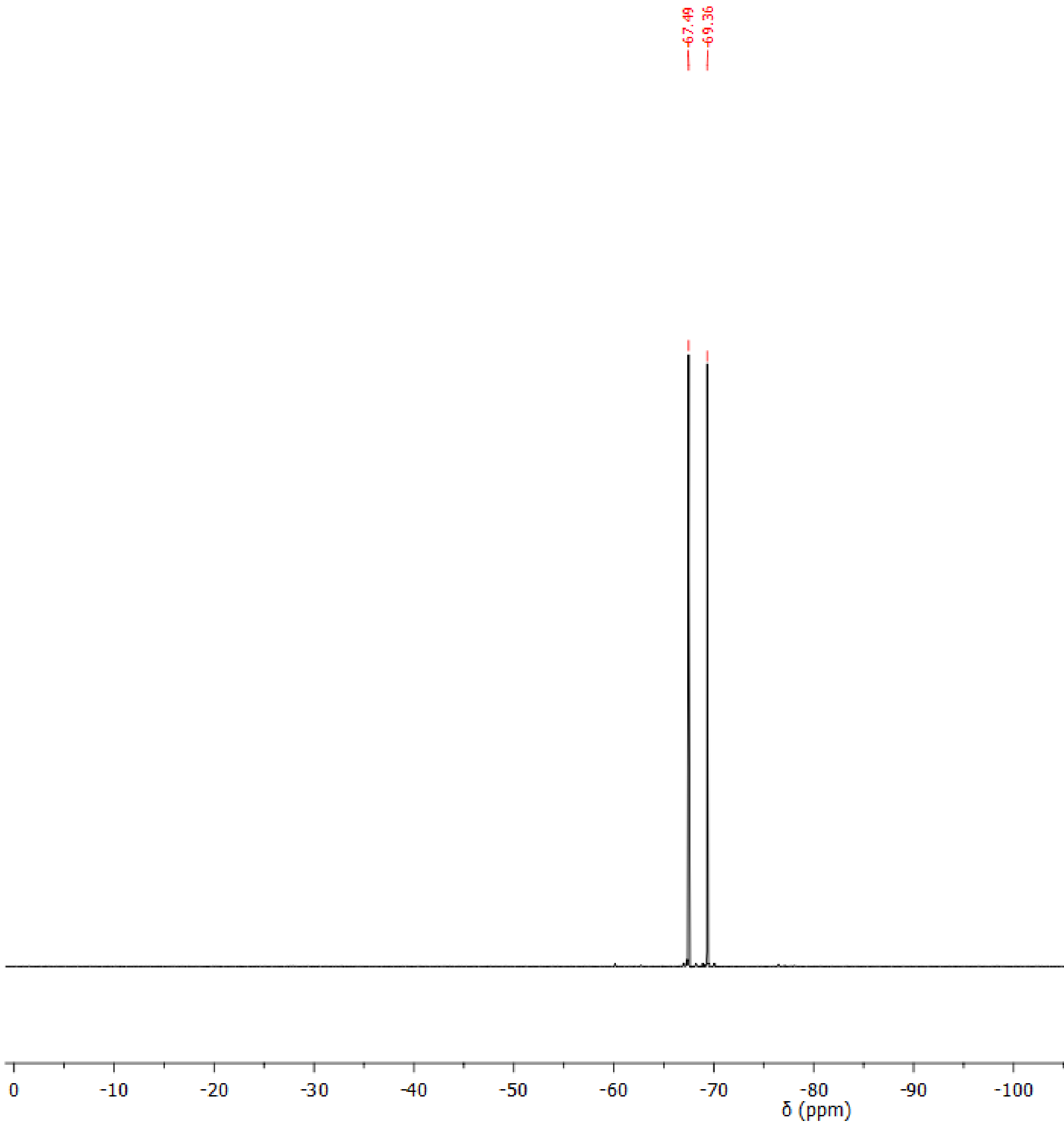

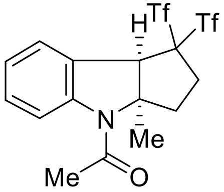

7c

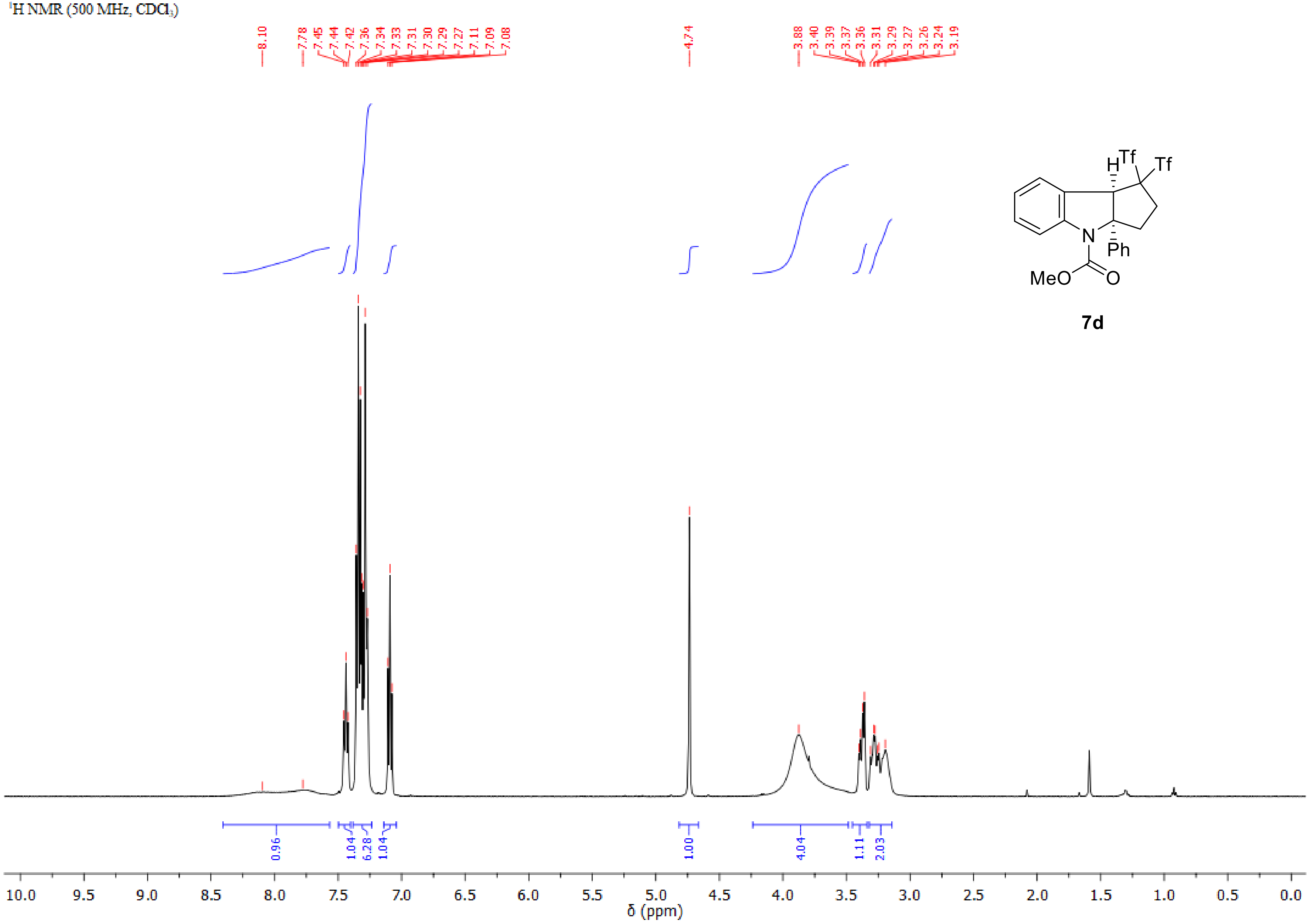
^{1}H NMR (500 MHz, CDCl$_3$)
8.10 7.78 7.45 7.44 7.42 7.36 7.34 7.33 7.31 7.30 7.29 7.27 7.11 7.09 7.08
4.74
3.88 3.40 3.39 3.37 3.36 3.31 3.29 3.27 3.26 3.24 3.19
0.96 1.04 6.28 1.04 1.00 4.04 1.11 2.03
H Tf Tf N Ph MeO O
δ (ppm)
7d

^{13}C NMR (125 MHz, $CDCl_3$)

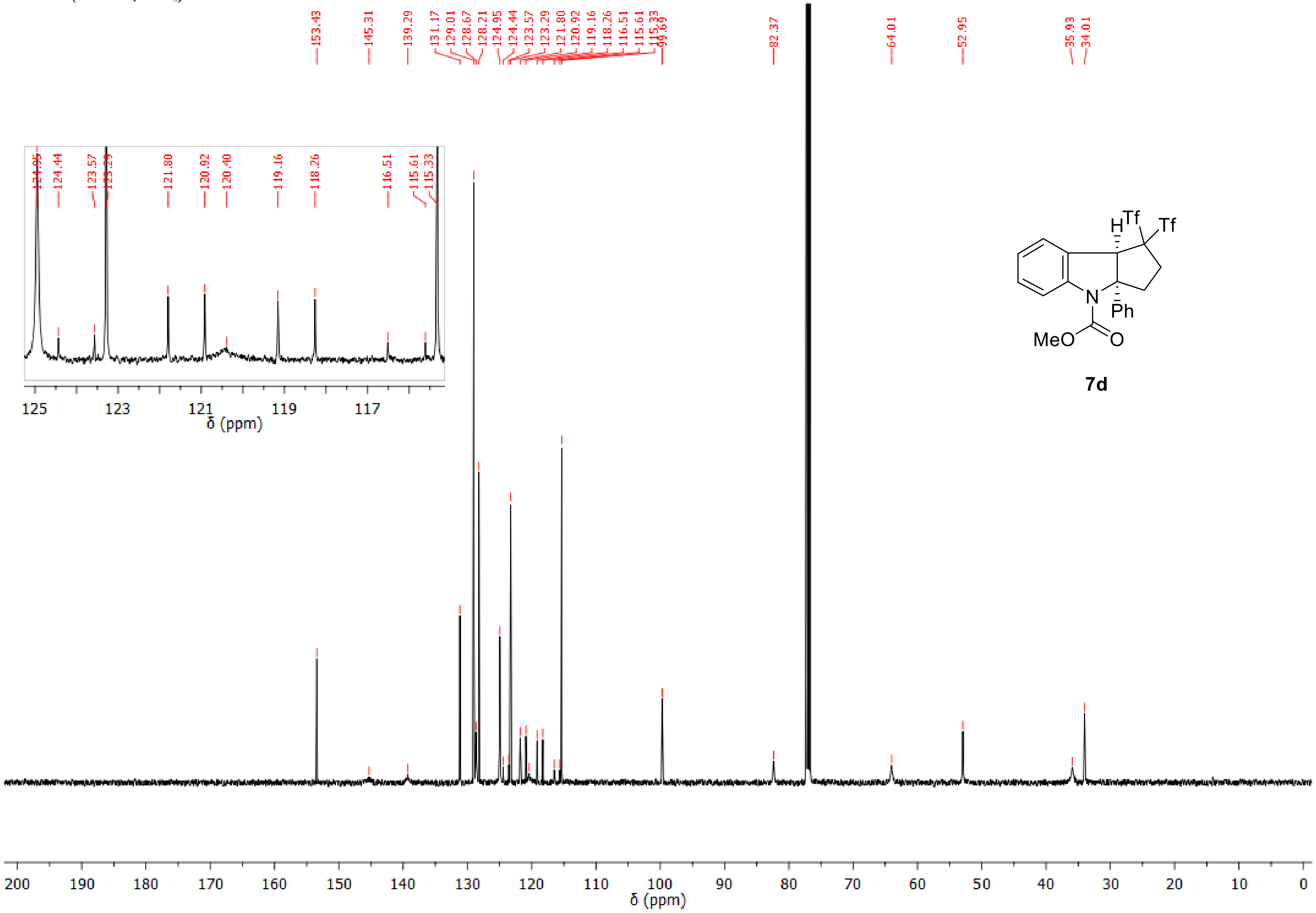

7d

^{19}F NMR (282 MHz, $CDCl_3$)

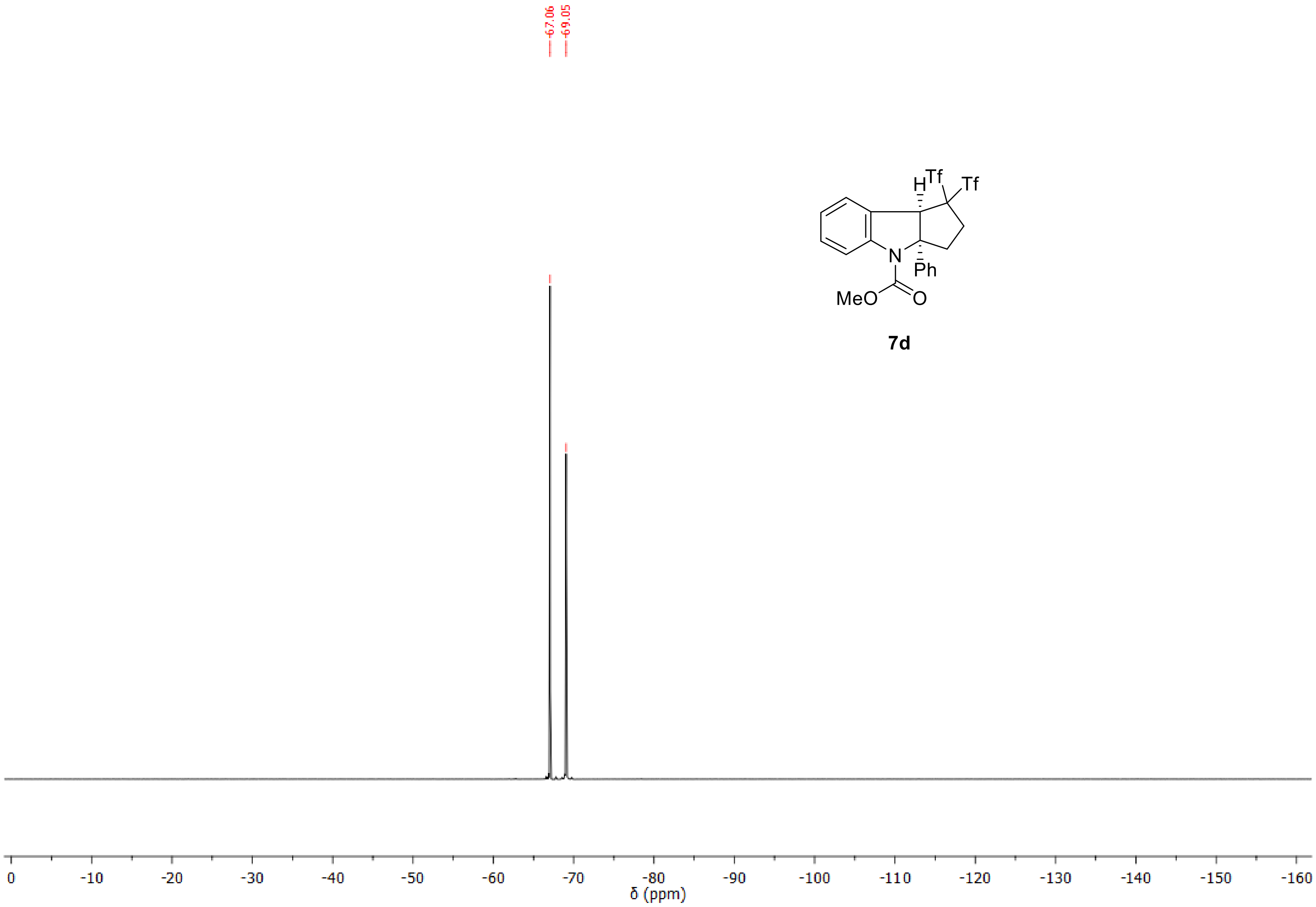

^1H NMR (500 MHz, $CDCl_3$)

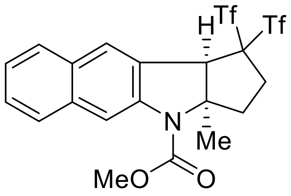

7e

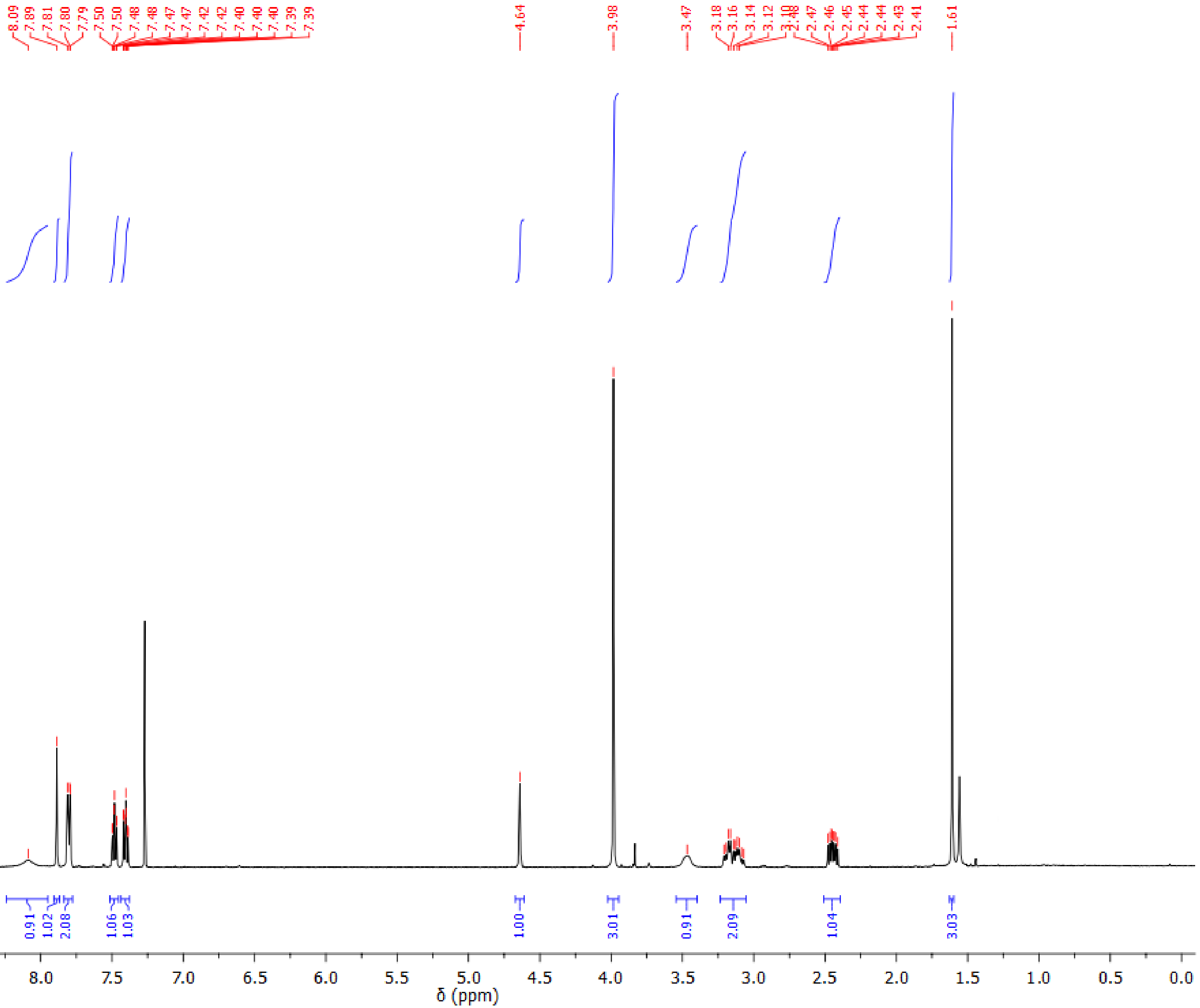

^{13}C NMR (125 MHz, $CDCl_3$)

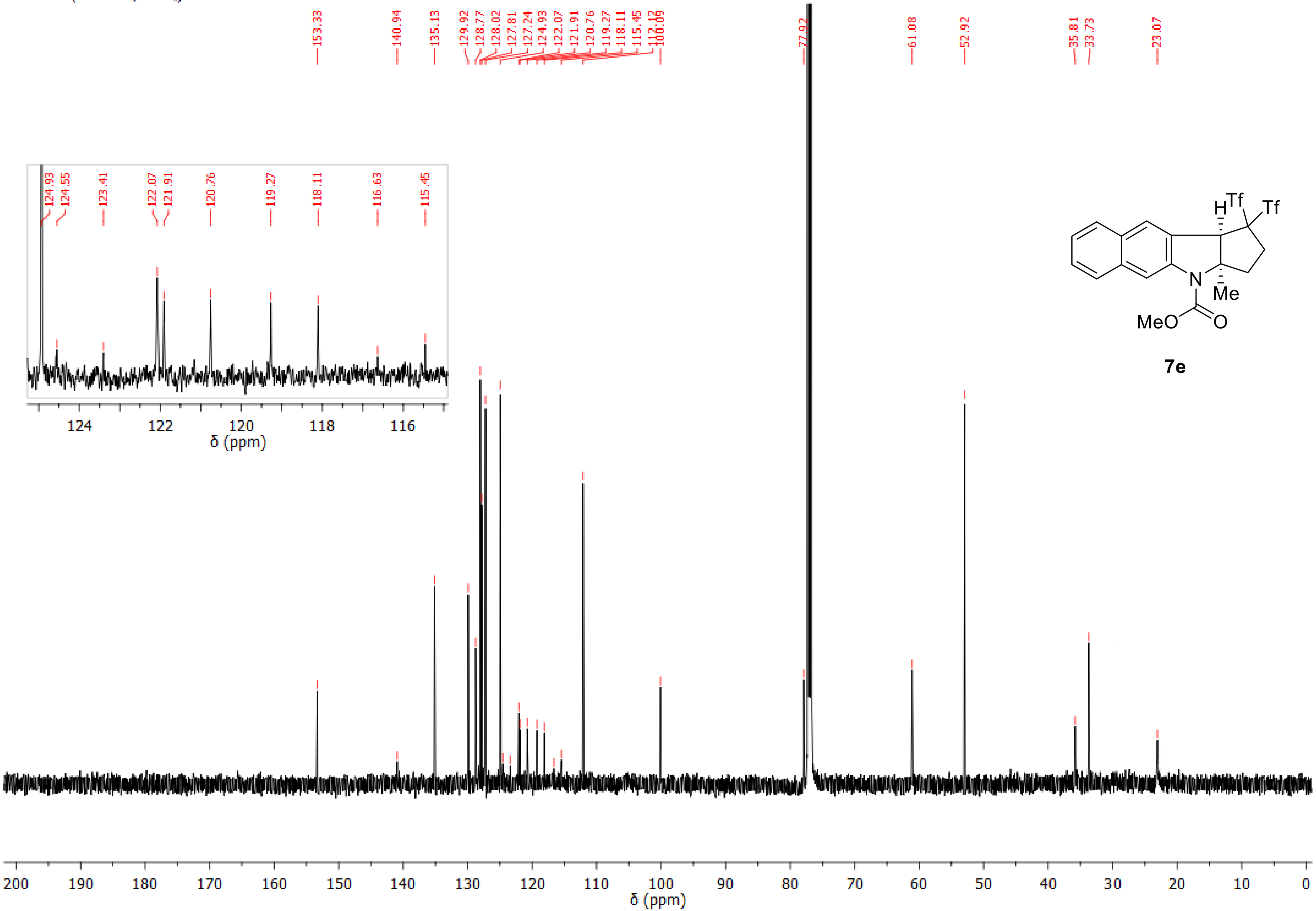

7e

^{19}F NMR (282 MHz, $CDCl_3$)

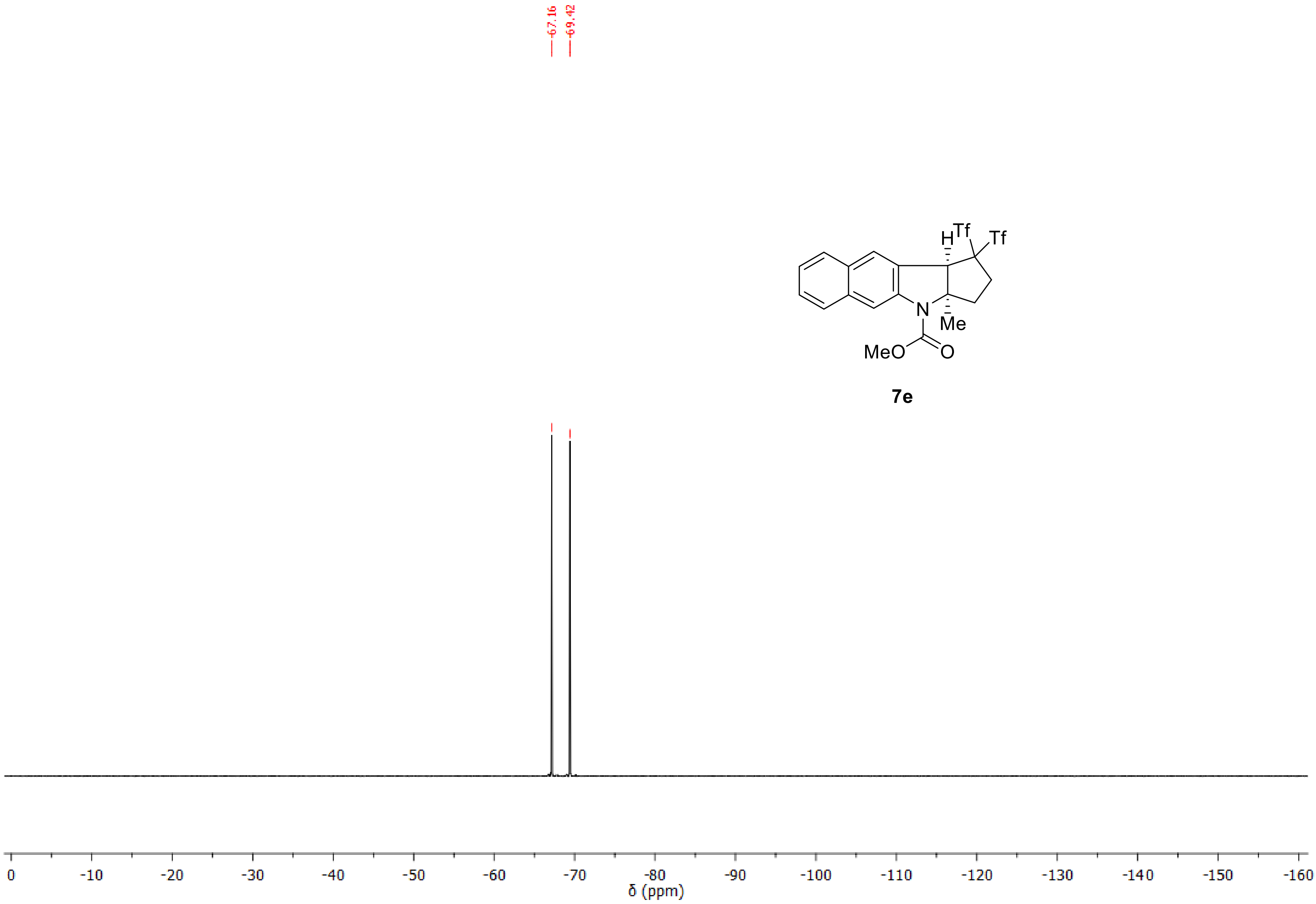

^{1}H NMR (500 MHz, $CDCl_3$)

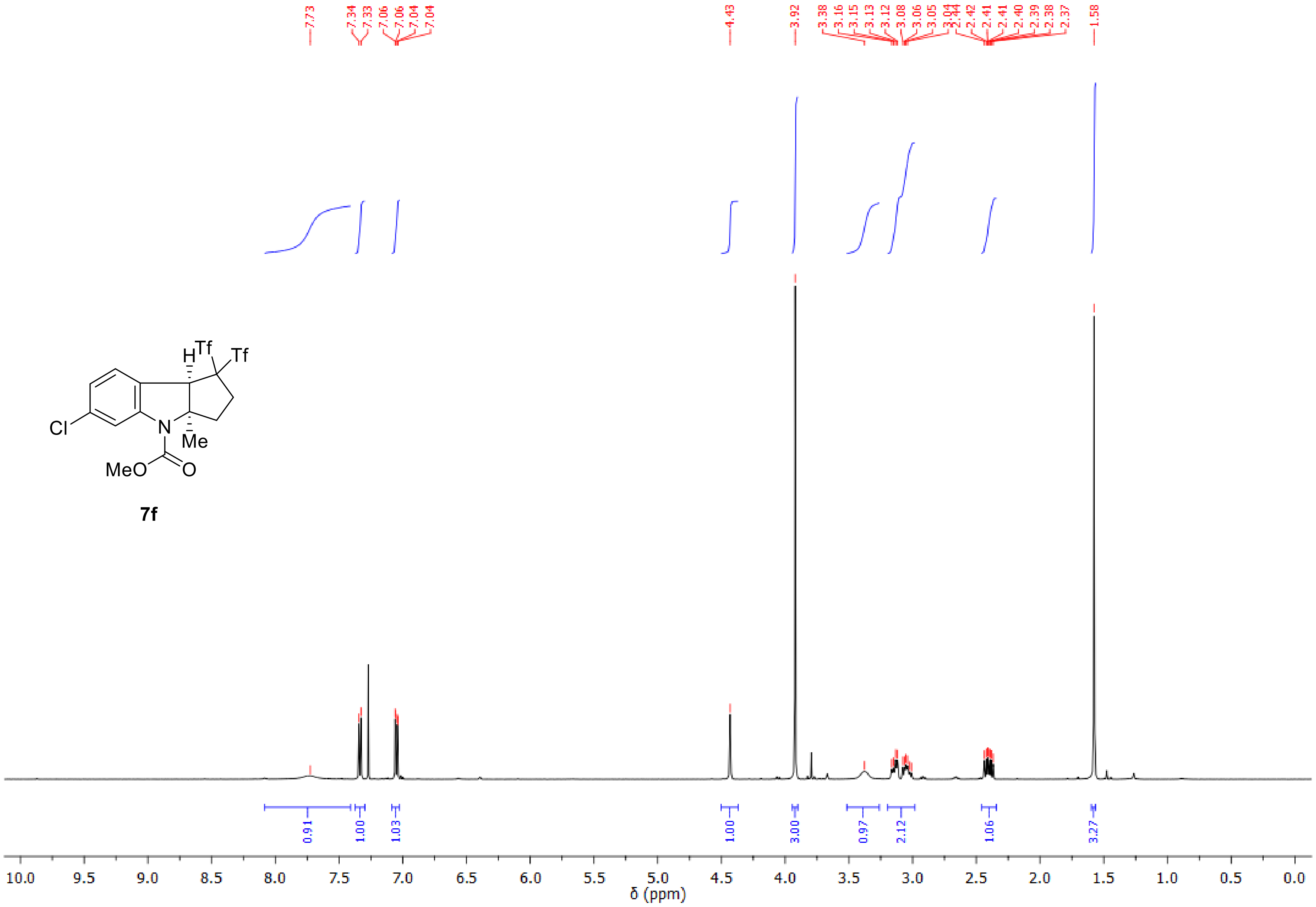

^{13}C NMR (125 MHz, CDCl$_3$)

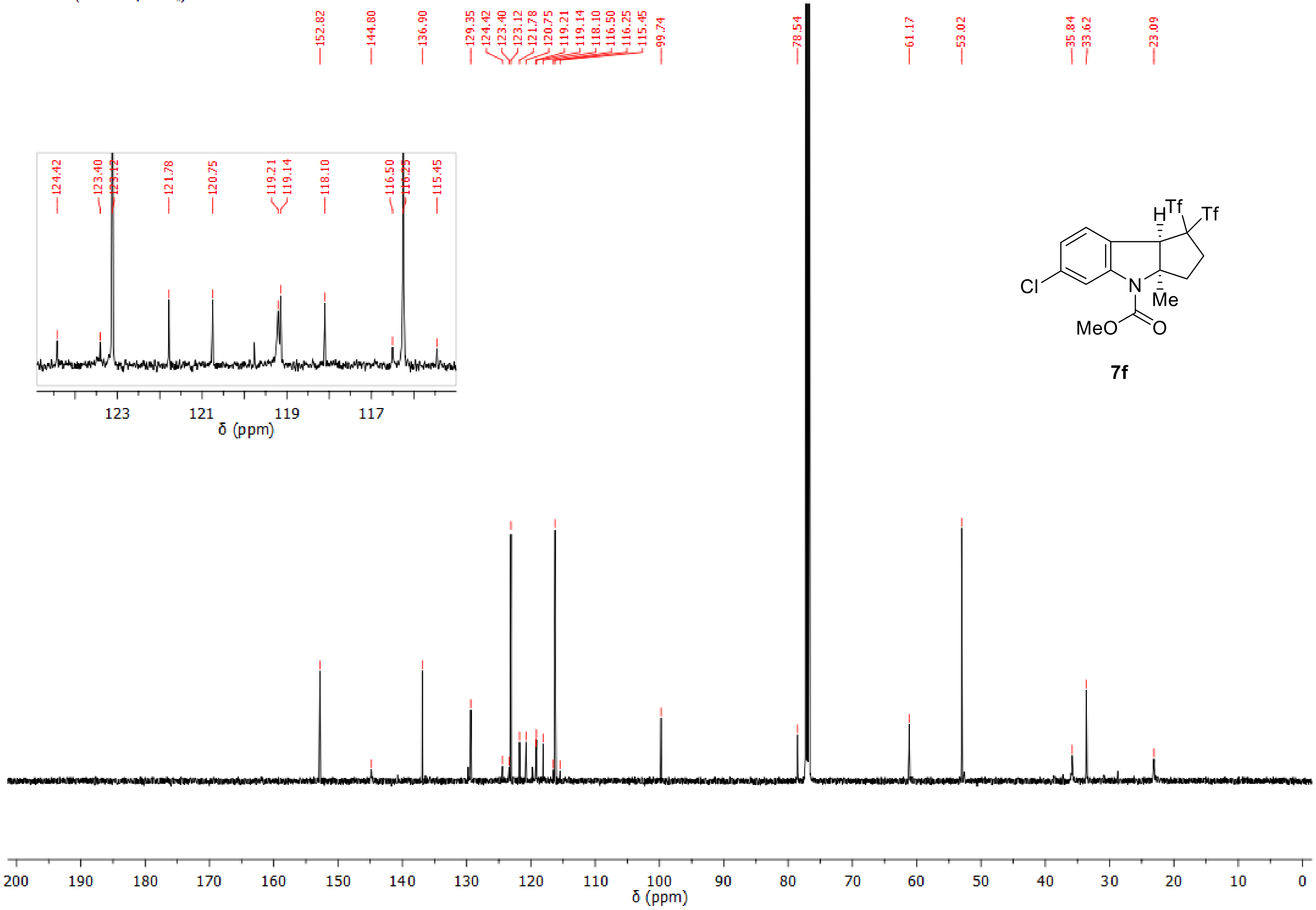

7f

^{19}F NMR (282 MHz, $CDCl_3$)

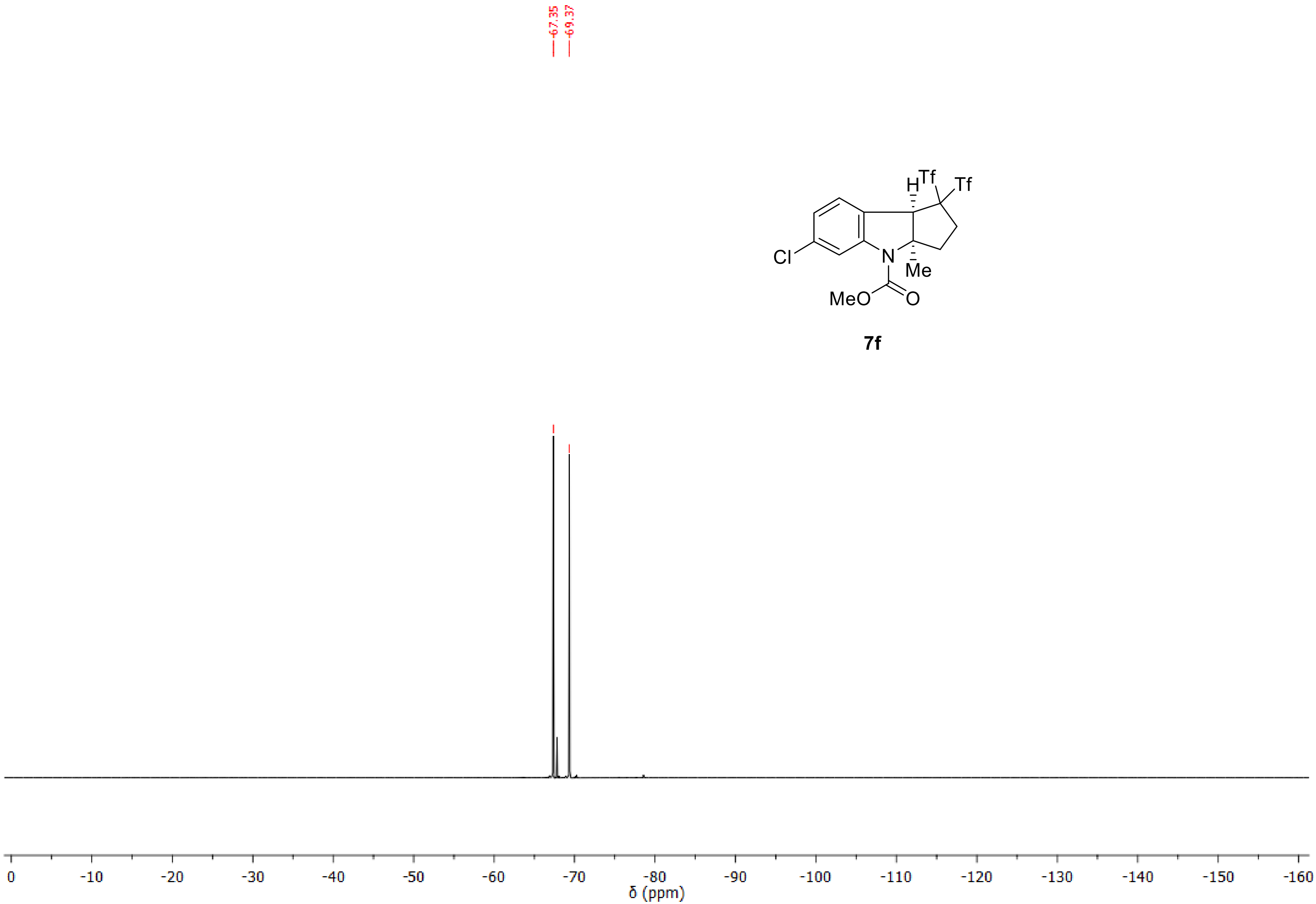

7f

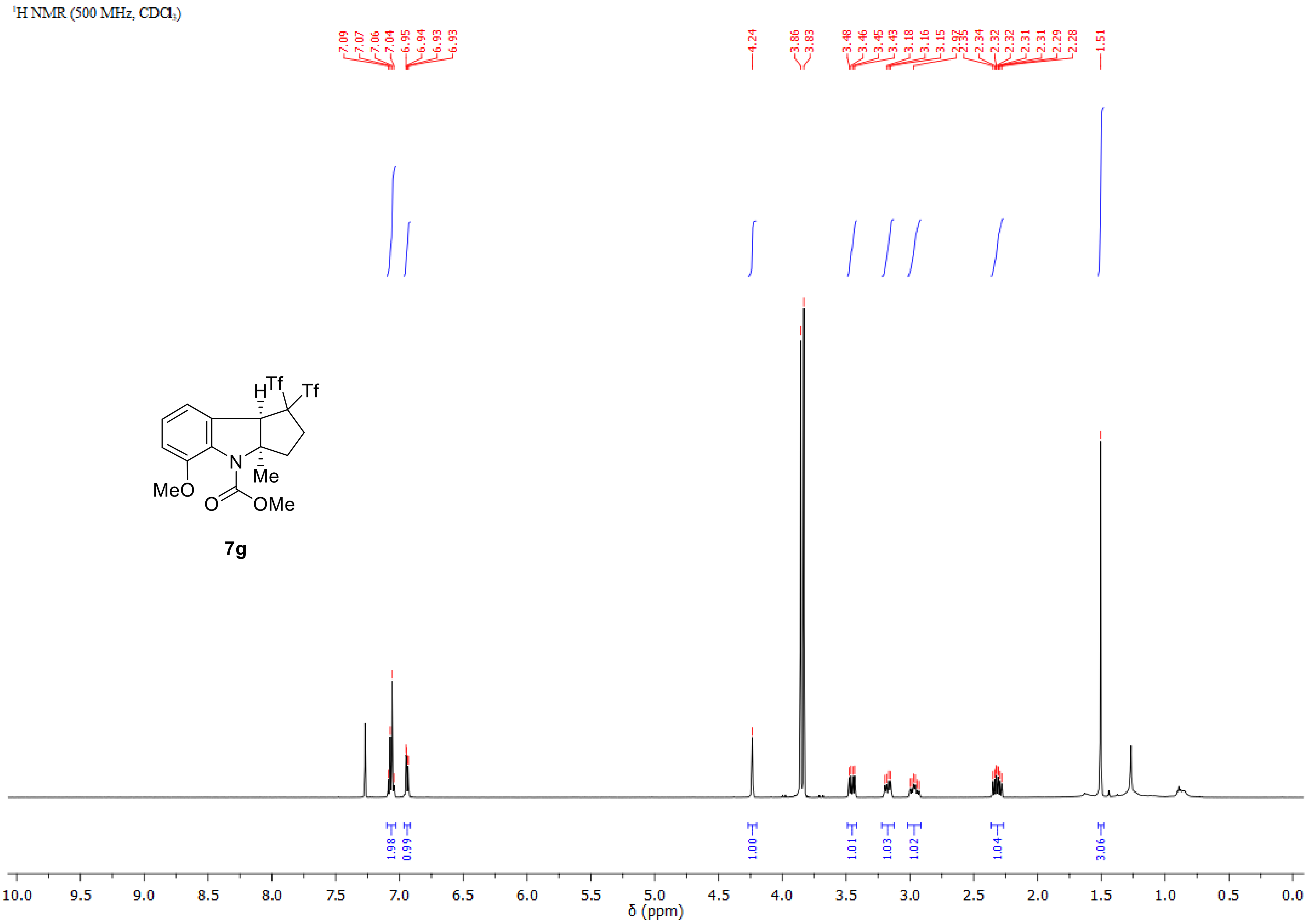

¹H NMR (500 MHz, CDCl₃)
7.09
7.07
7.06
7.04
6.95
6.94
6.93
6.93
4.24
3.86
3.83
3.48
3.46
3.45
3.43
3.18
3.16
3.15
2.97
2.35
2.34
2.32
2.32
2.31
2.31
2.29
2.28
1.51
1.98
0.99
1.00
1.01
1.03
1.02
1.04
3.06
H
Tf
Tf
N
Me
MeO
O
OMe
7g
δ (ppm)

^{13}C NMR (125 MHz, CDCl$_3$)

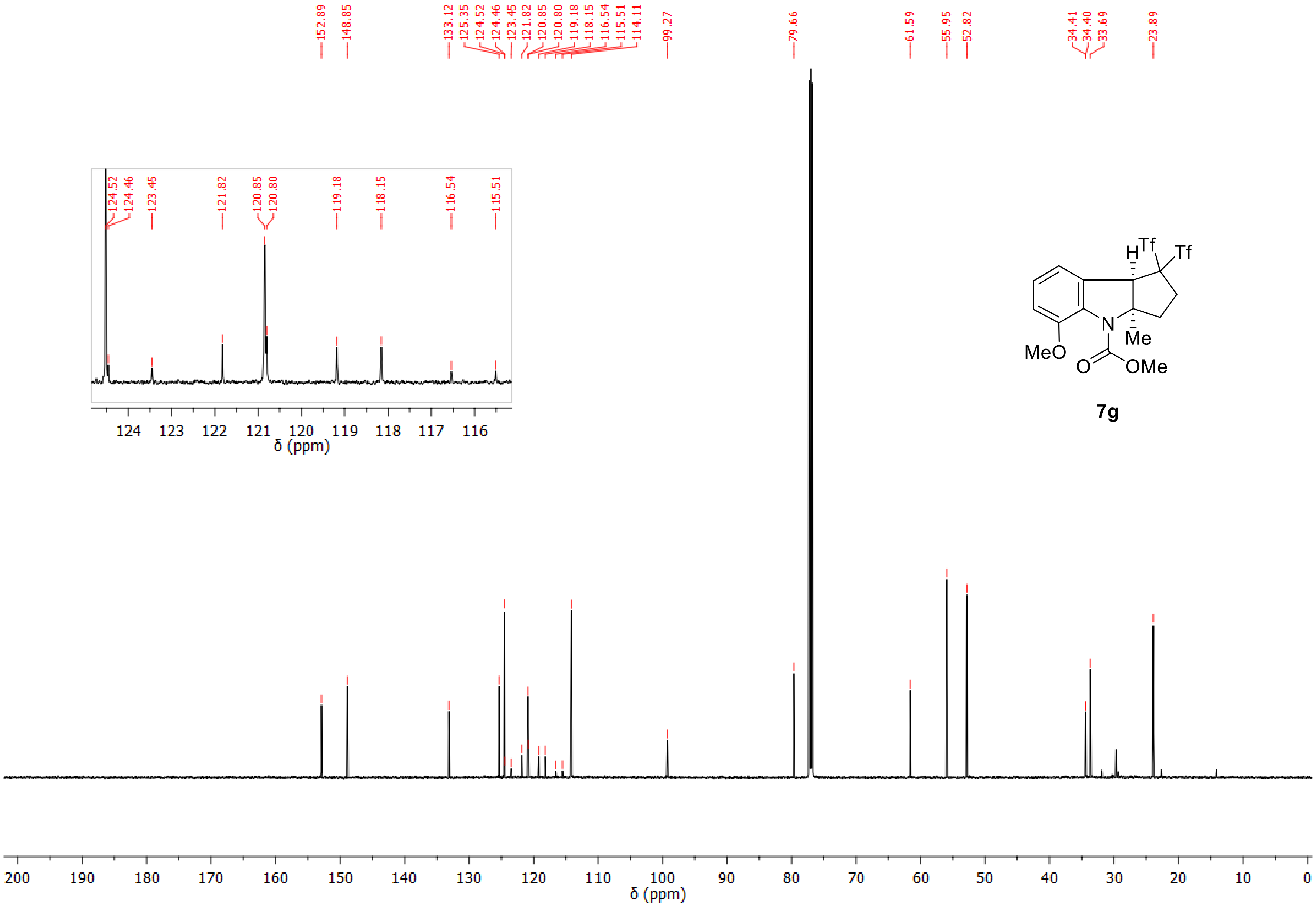

7g

^{19}F NMR (282 MHz, CDCl$_3$)

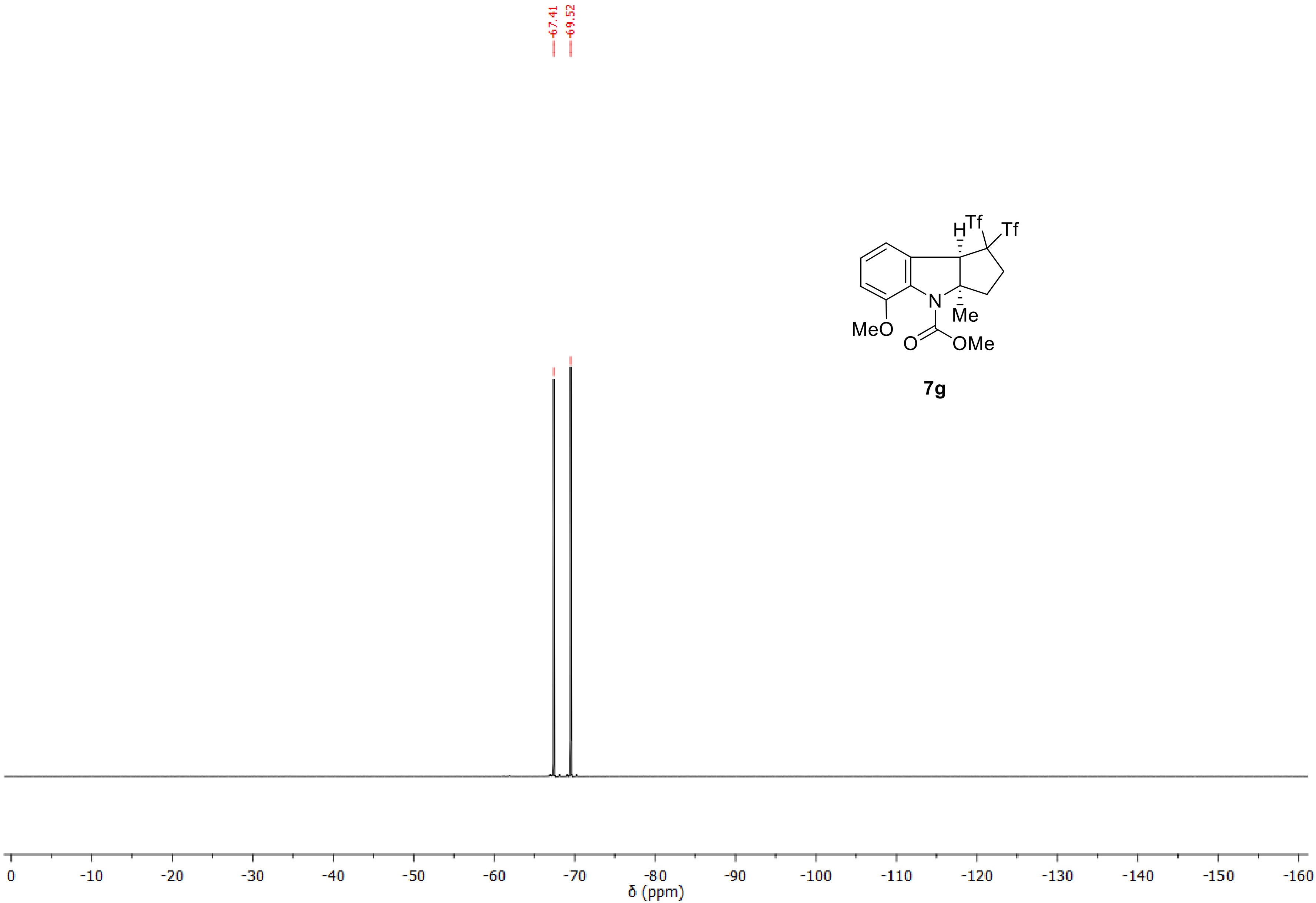

^{1}H NMR (500 MHz, $CDCl_3$)

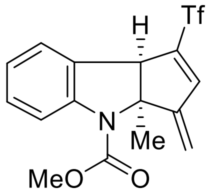

8

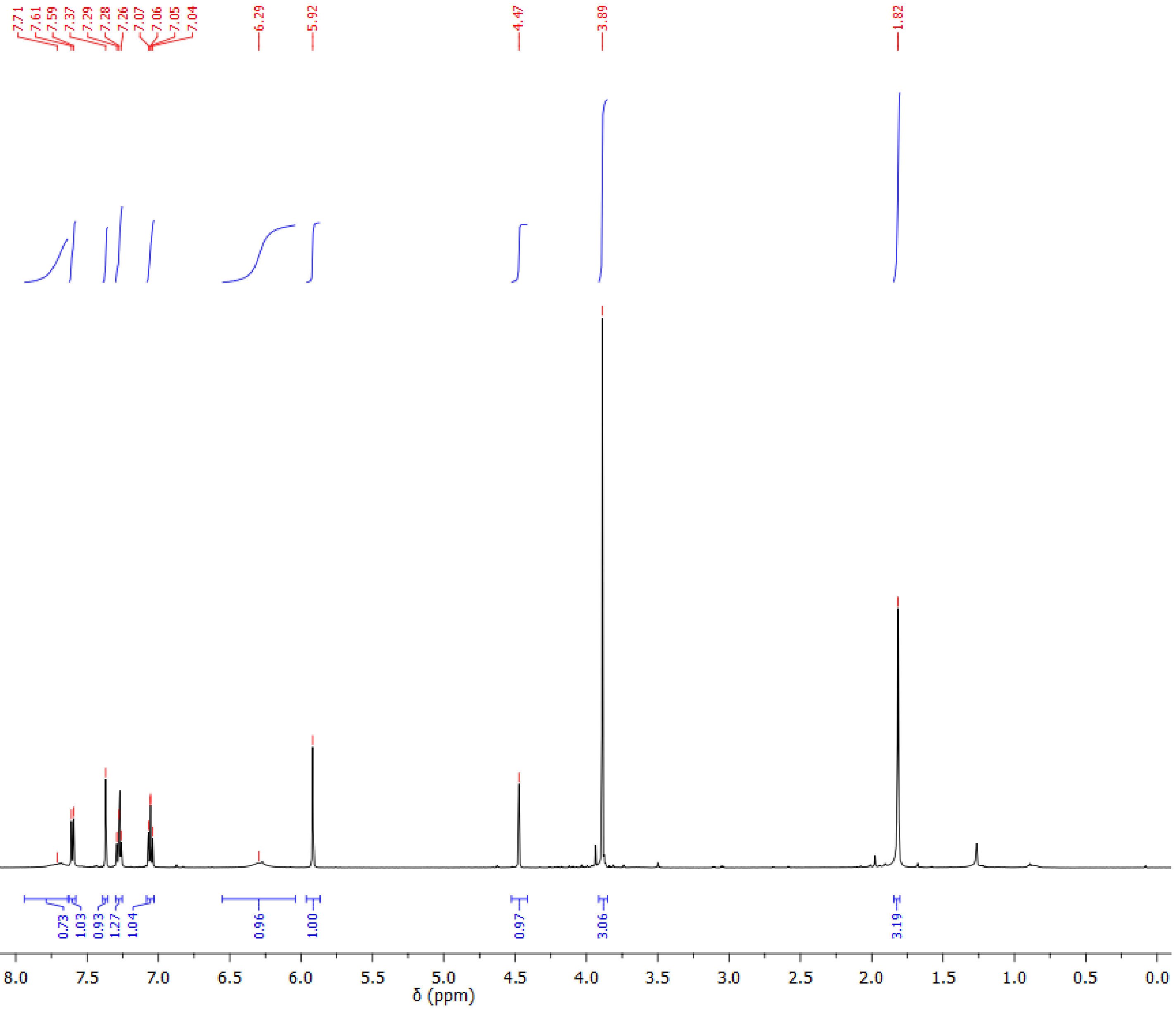

^{13}C NMR (125 MHz, $CDCl_3$)

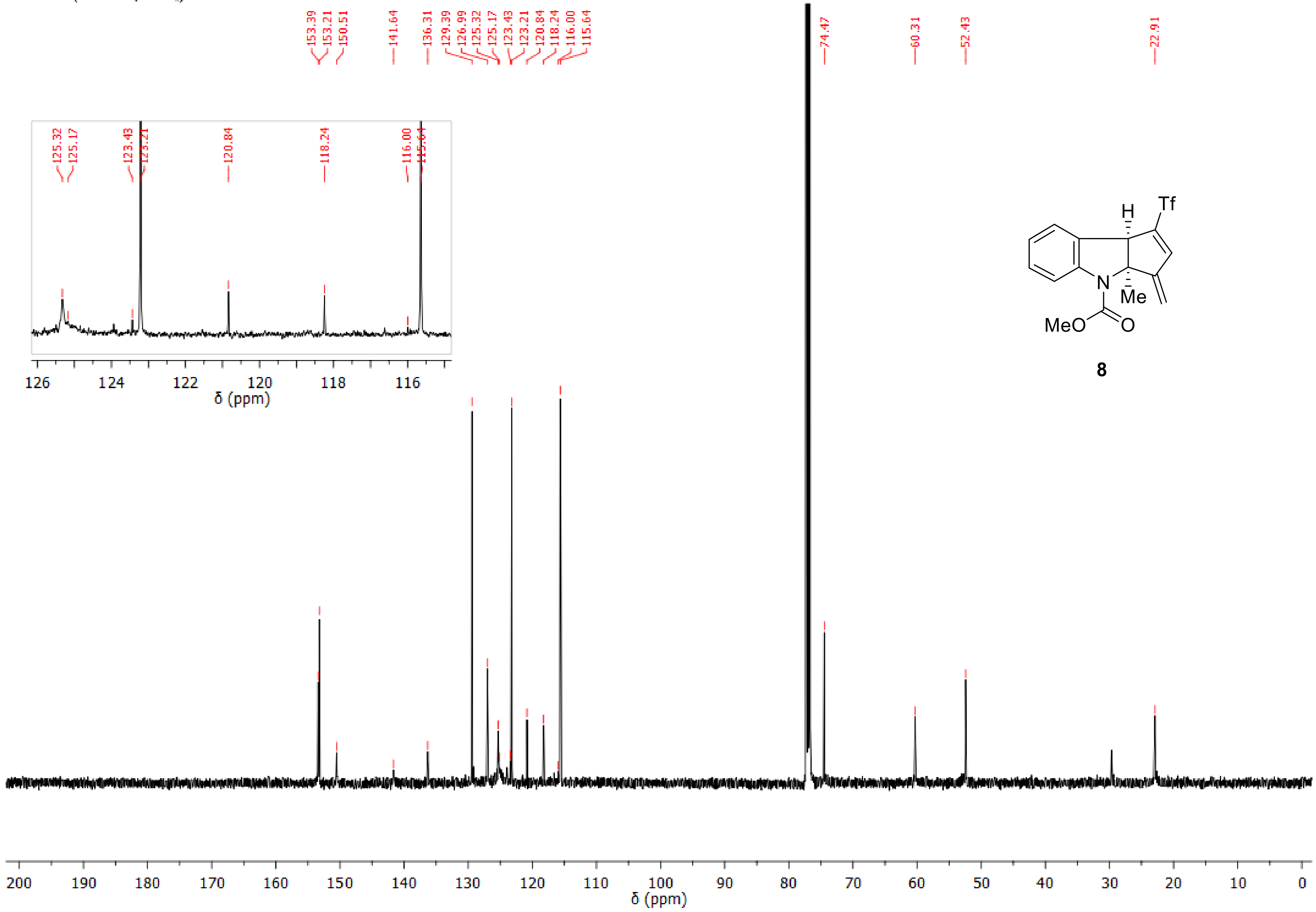

8

^{19}F NMR (282 MHz, CDCl$_3$)

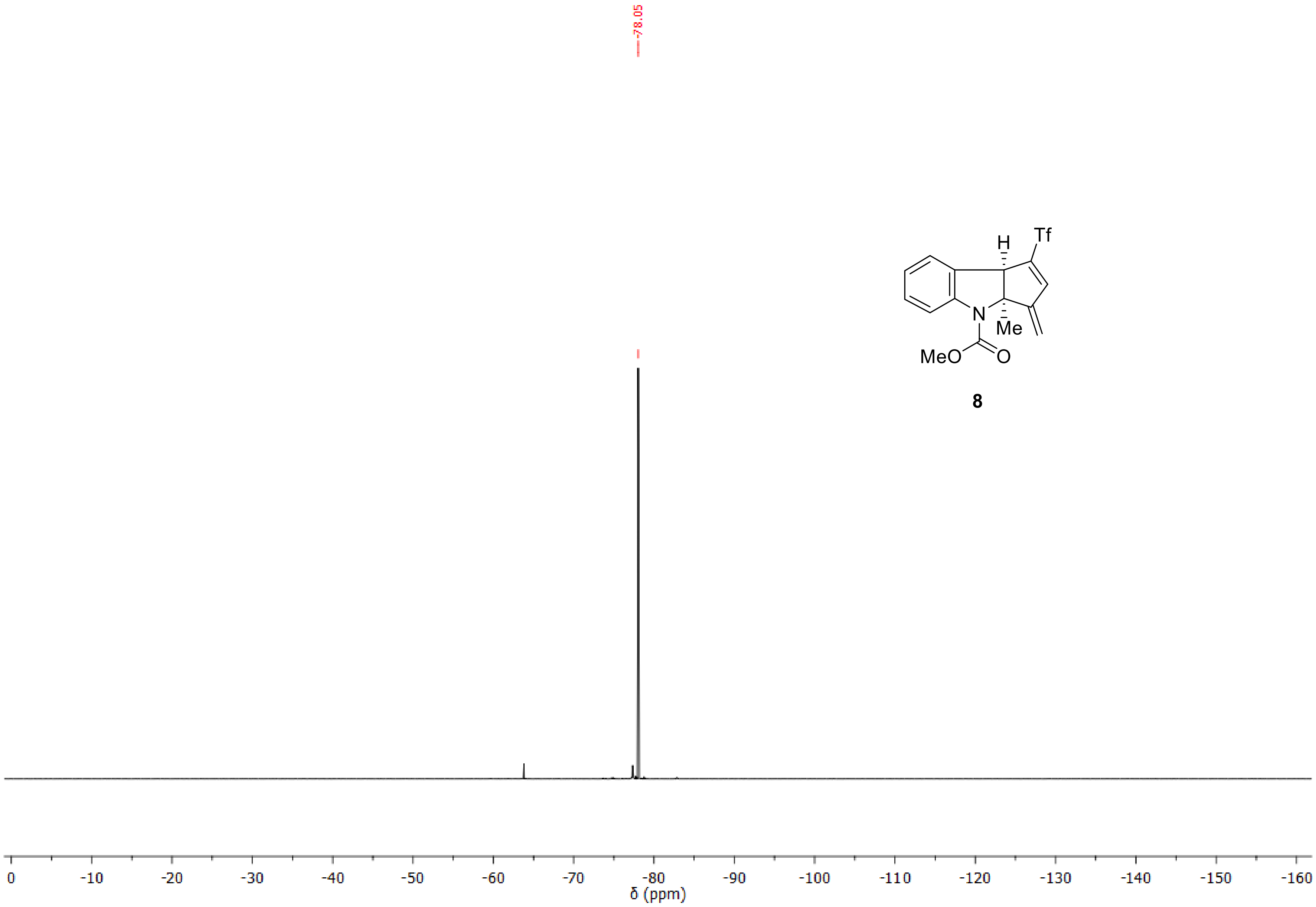

8

^{1}H NMR (500 MHz, $CDCl_3$)

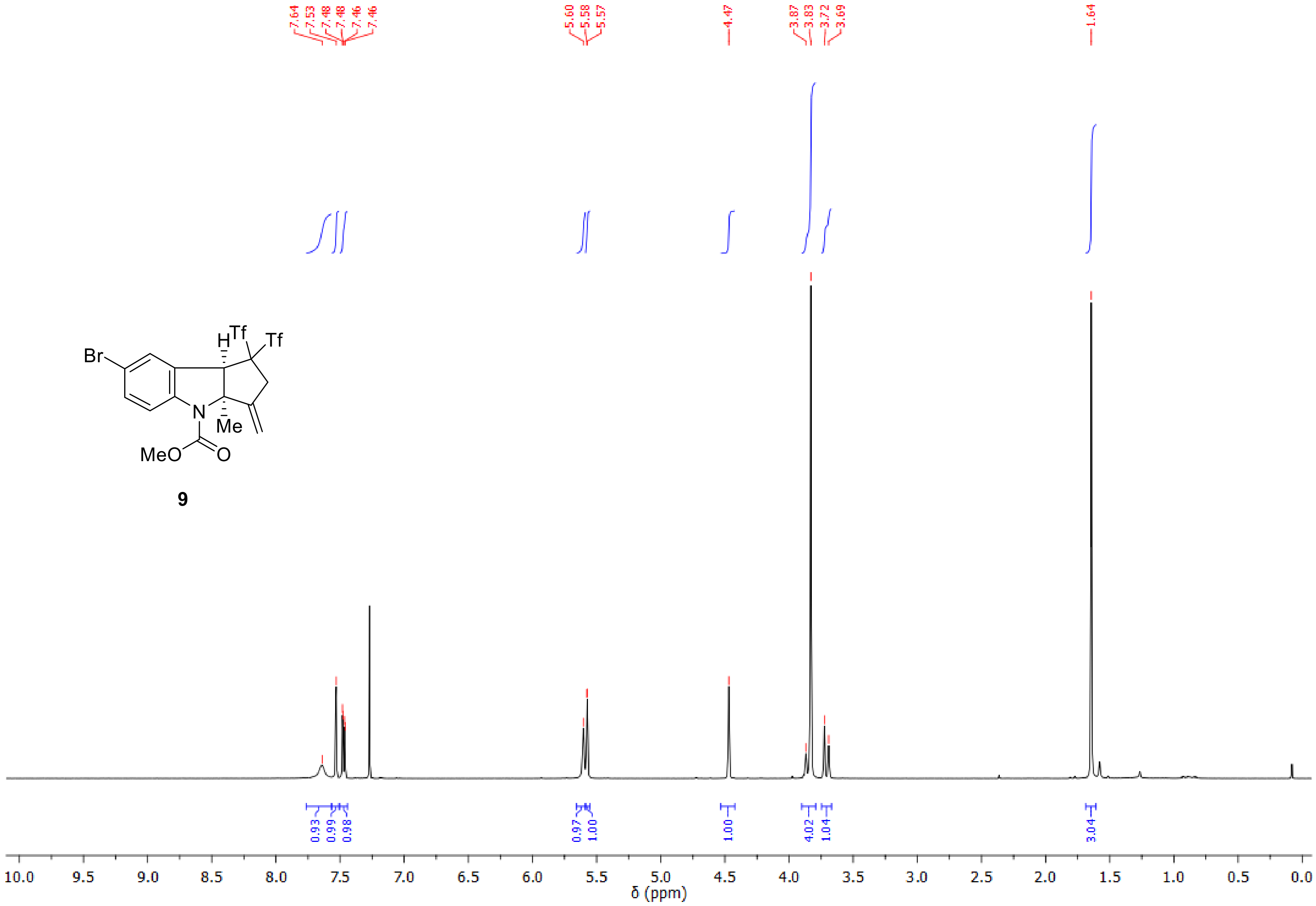

^{13}C NMR (125 MHz, $CDCl_3$)

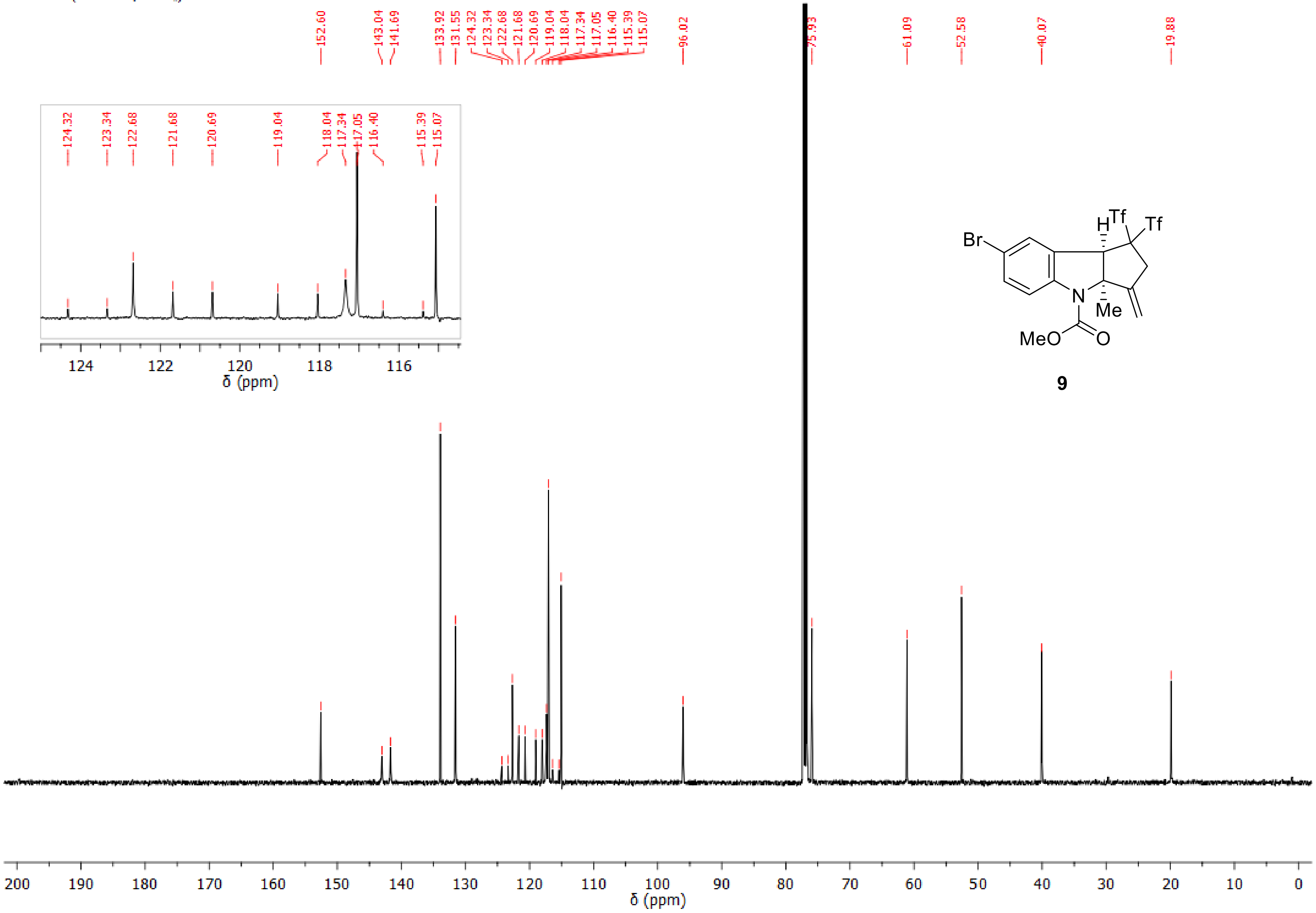

9

^{19}F NMR (282 MHz, $CDCl_3$)

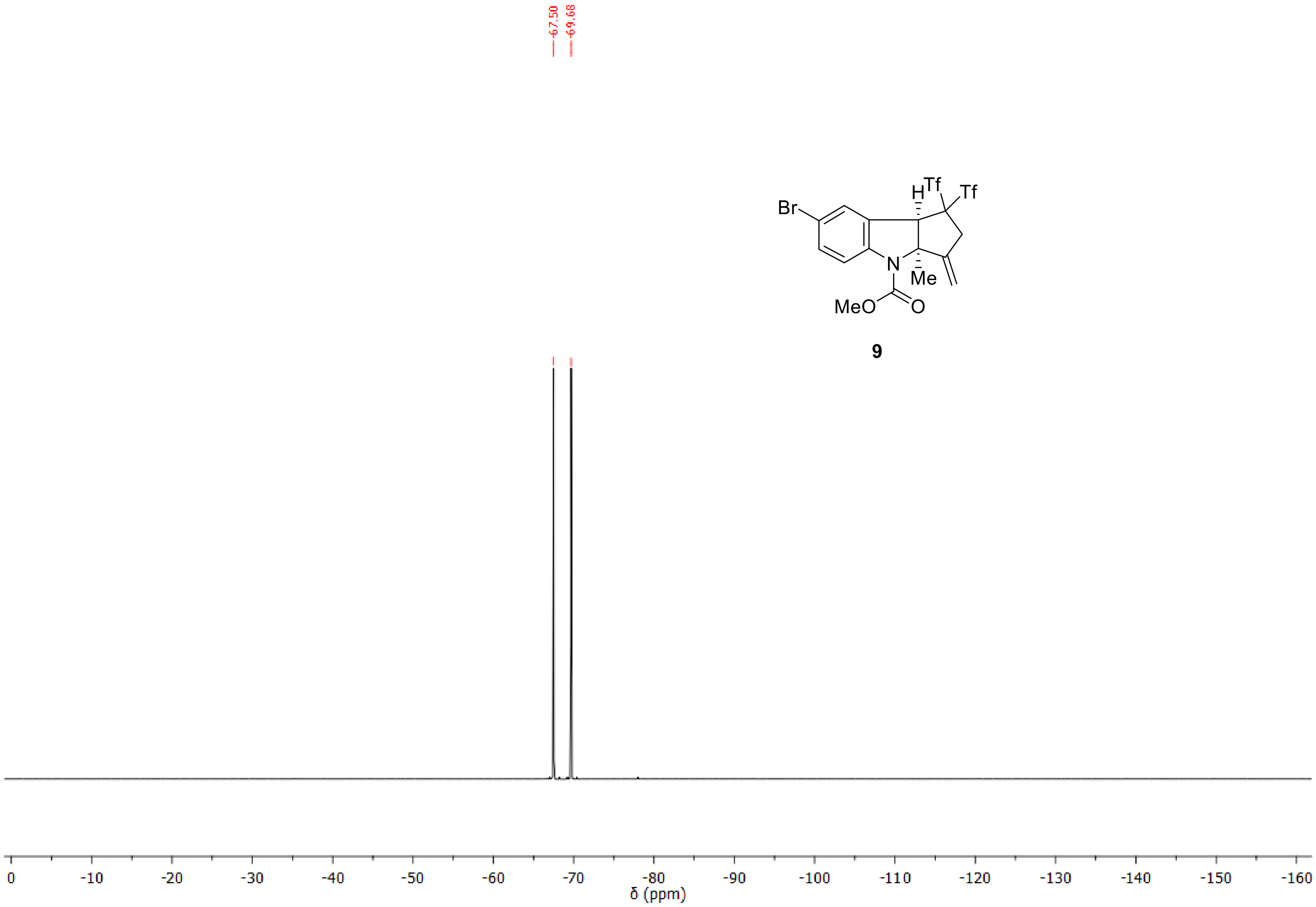

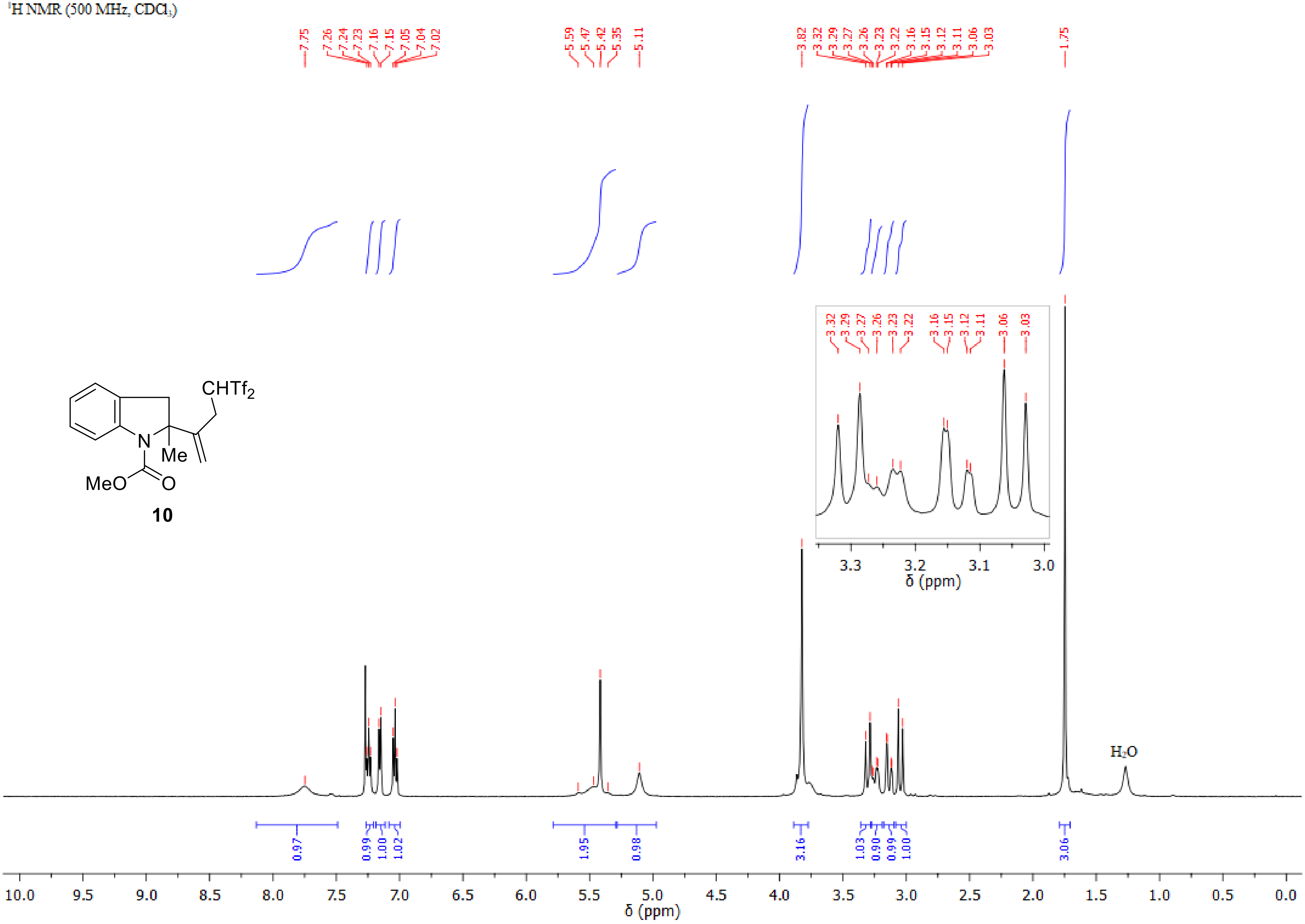

1H NMR (500 MHz, CDCl3)
7.75
7.26
7.24
7.23
7.16
7.15
7.05
7.04
7.02
5.59
5.47
5.42
5.35
5.11
3.82
3.32
3.29
3.27
3.26
3.23
3.22
3.16
3.15
3.12
3.11
3.06
3.03
1.75
CHTf2
N
Me
MeO
O
10
H2O
0.97
0.99
1.00
1.02
1.95
0.98
3.16
1.03
0.90
0.99
1.00
3.06
δ (ppm)

^{13}C NMR (125 MHz, $CDCl_3$)

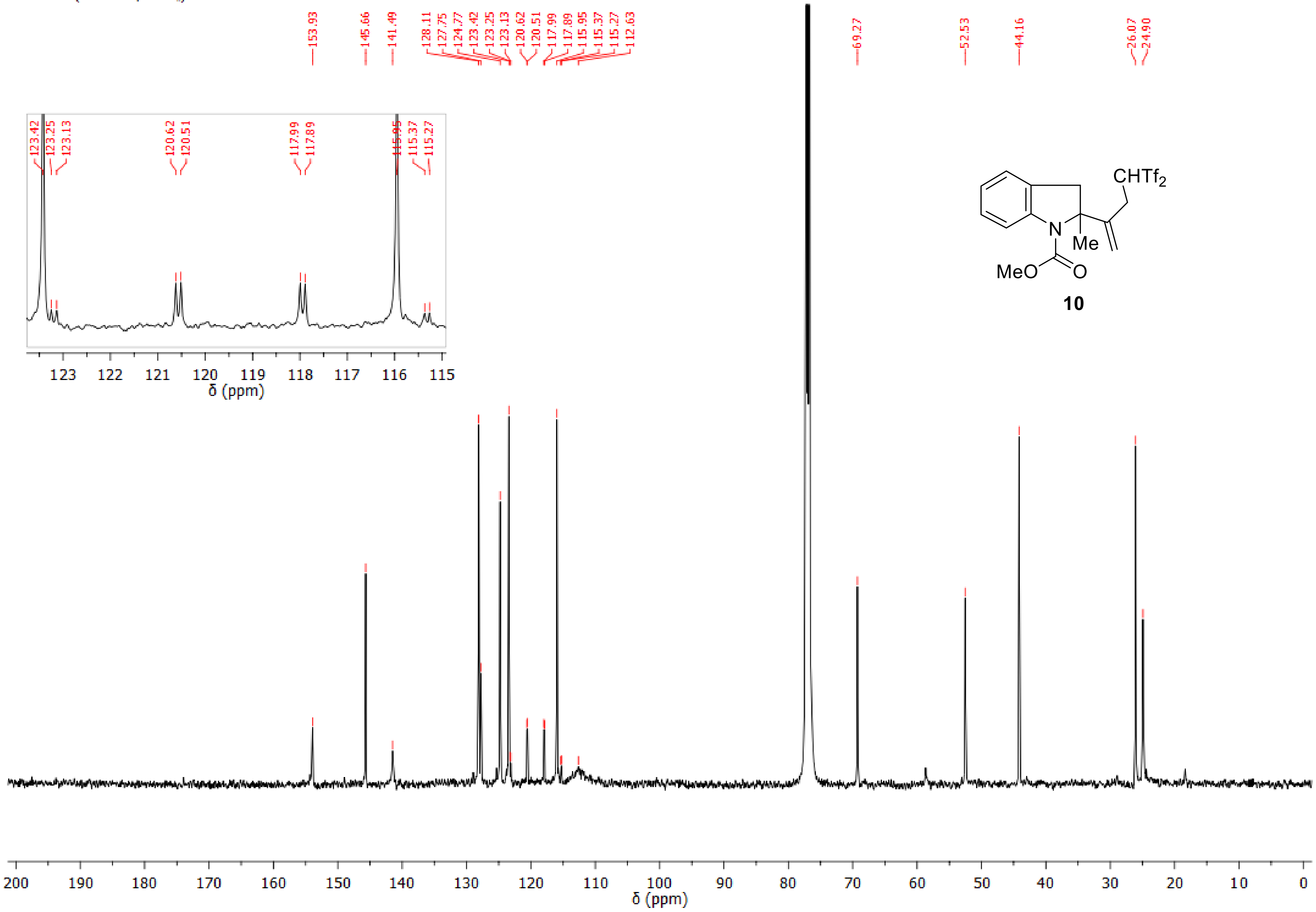

^{19}F NMR (282 MHz, $CDCl_3$)

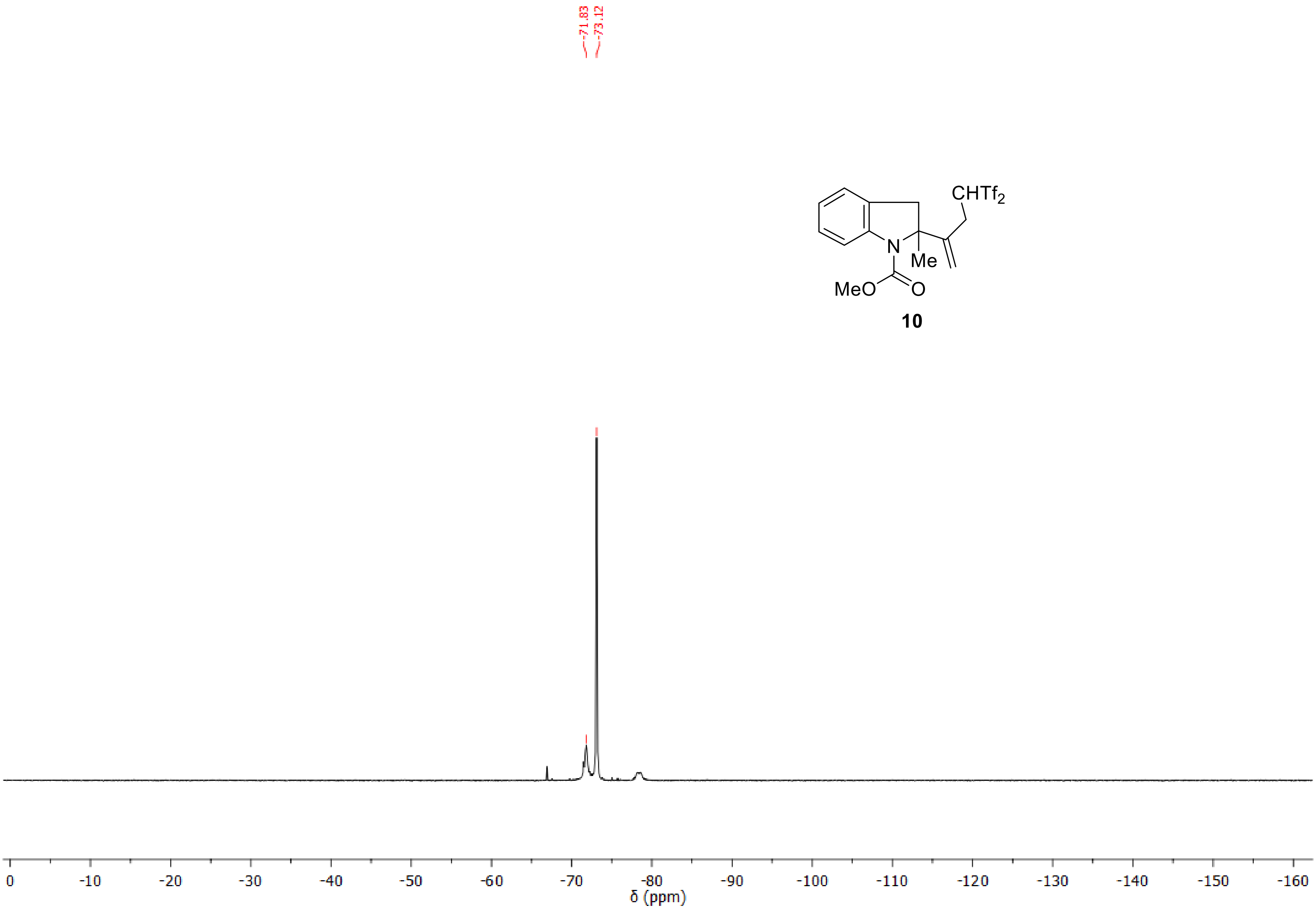

10

^1H NMR (500 MHz, Acetone)

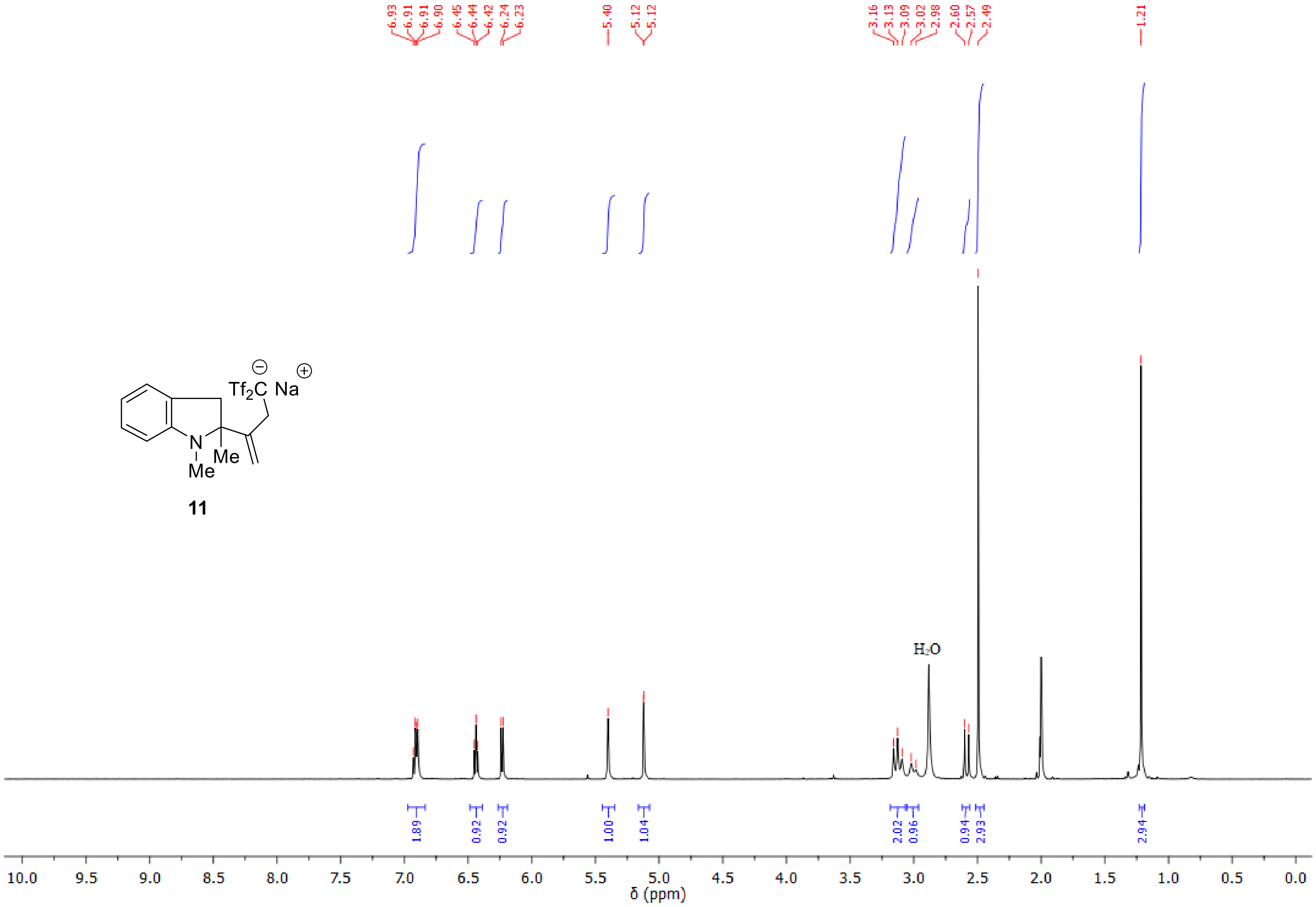

11

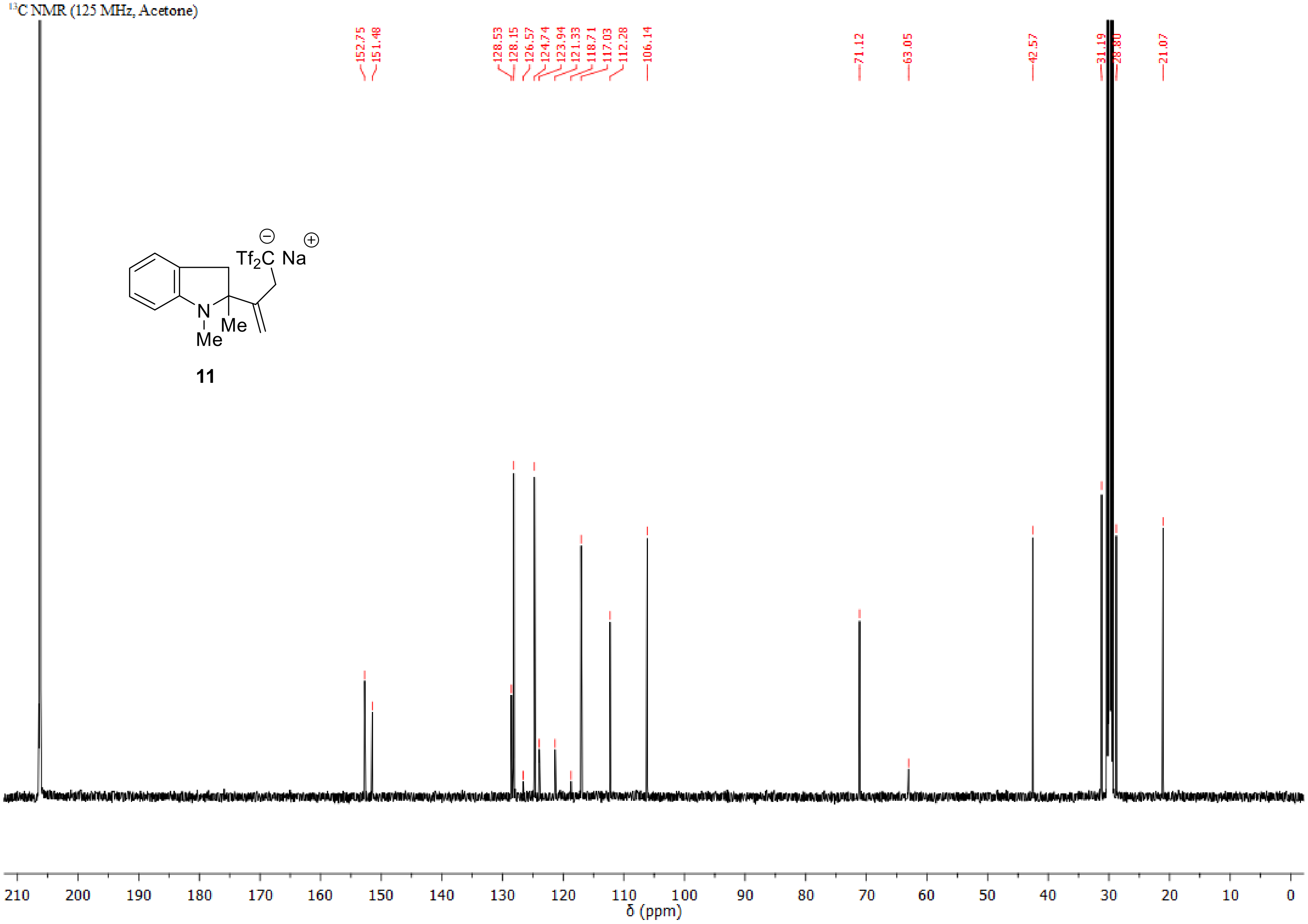
13C NMR (125 MHz, Acetone)
152.75
151.48
128.53
128.15
126.57
124.74
123.94
121.33
118.71
117.03
112.28
106.14
71.12
63.05
42.57
31.19
28.80
21.07
Tf2C Na
N
Me
Me
11
210 200 190 180 170 160 150 140 130 120 110 100 90 80 70 60 50 40 30 20 10 0
δ (ppm)

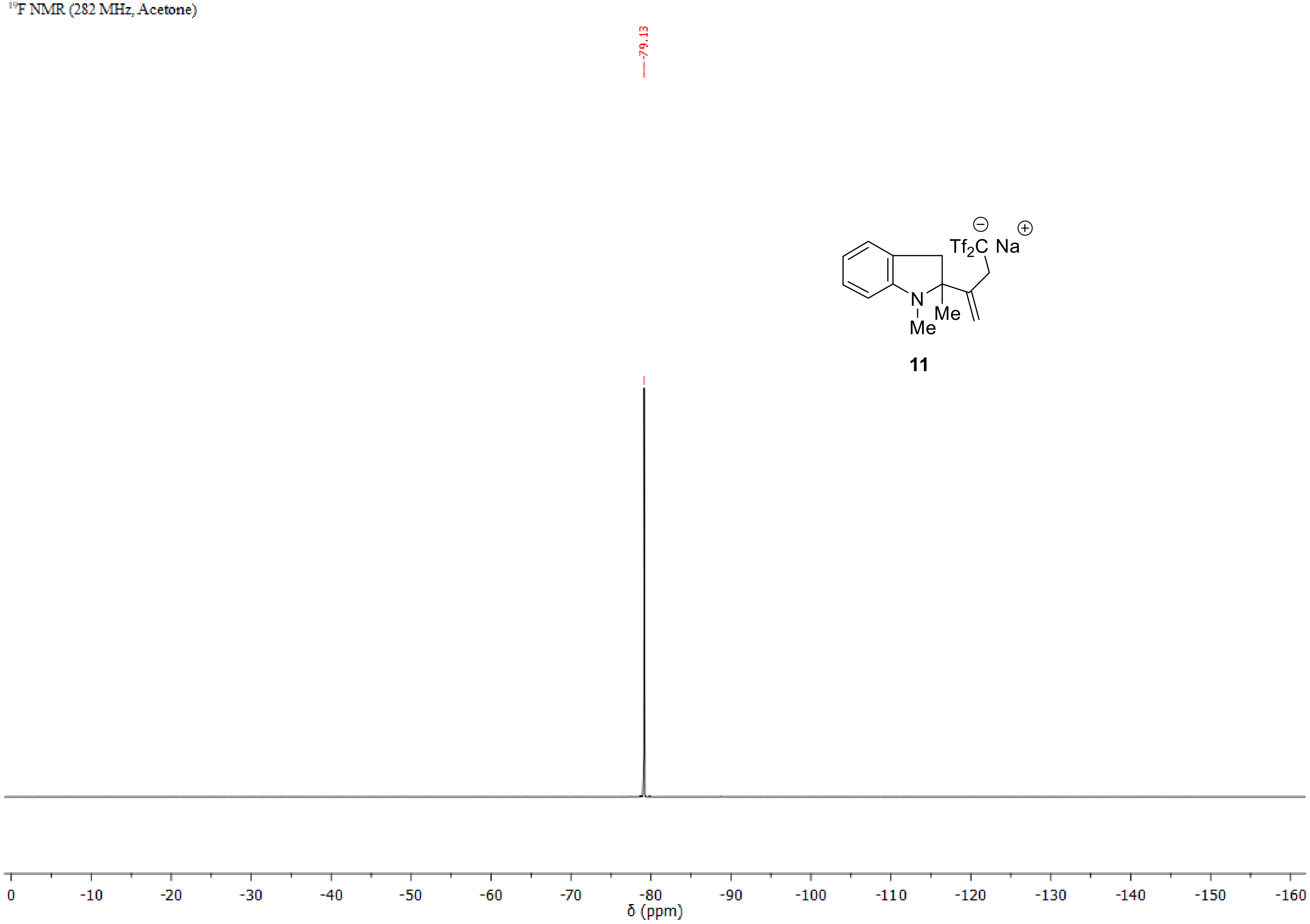
19F NMR (282 MHz, Acetone)
-79.13
Tf2C Na
N
Me
Me
11
0
-10
-20
-30
-40
-50
-60
-70
-80
-90
-100
-110
-120
-130
-140
-150
-160
δ (ppm)

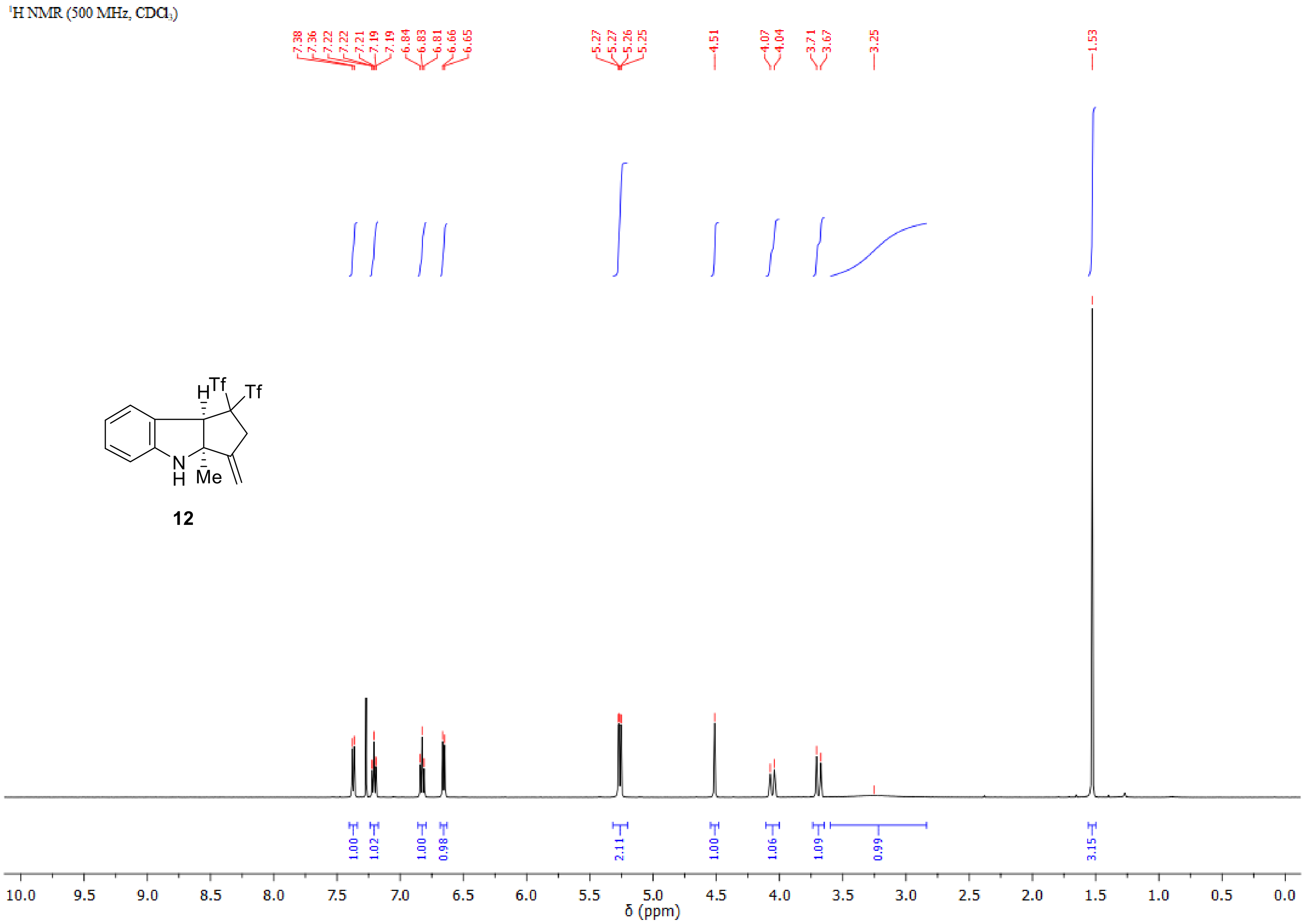

^{1}H NMR (500 MHz, $CDCl_3$)
7.38 7.36 7.22 7.22 7.21 7.19 7.19 6.84 6.83 6.81 6.66 6.65
5.27 5.27 5.26 5.25
4.51
4.07 4.04
3.71 3.67
3.25
1.53
1.00 1.02 1.00 0.98 2.11 1.00 1.06 1.09 0.99 3.15
H Tf Tf N H Me
12
10.0 9.5 9.0 8.5 8.0 7.5 7.0 6.5 6.0 5.5 5.0 4.5 4.0 3.5 3.0 2.5 2.0 1.5 1.0 0.5 0.0
δ (ppm)

^{13}C NMR (125 MHz, $CDCl_3$)

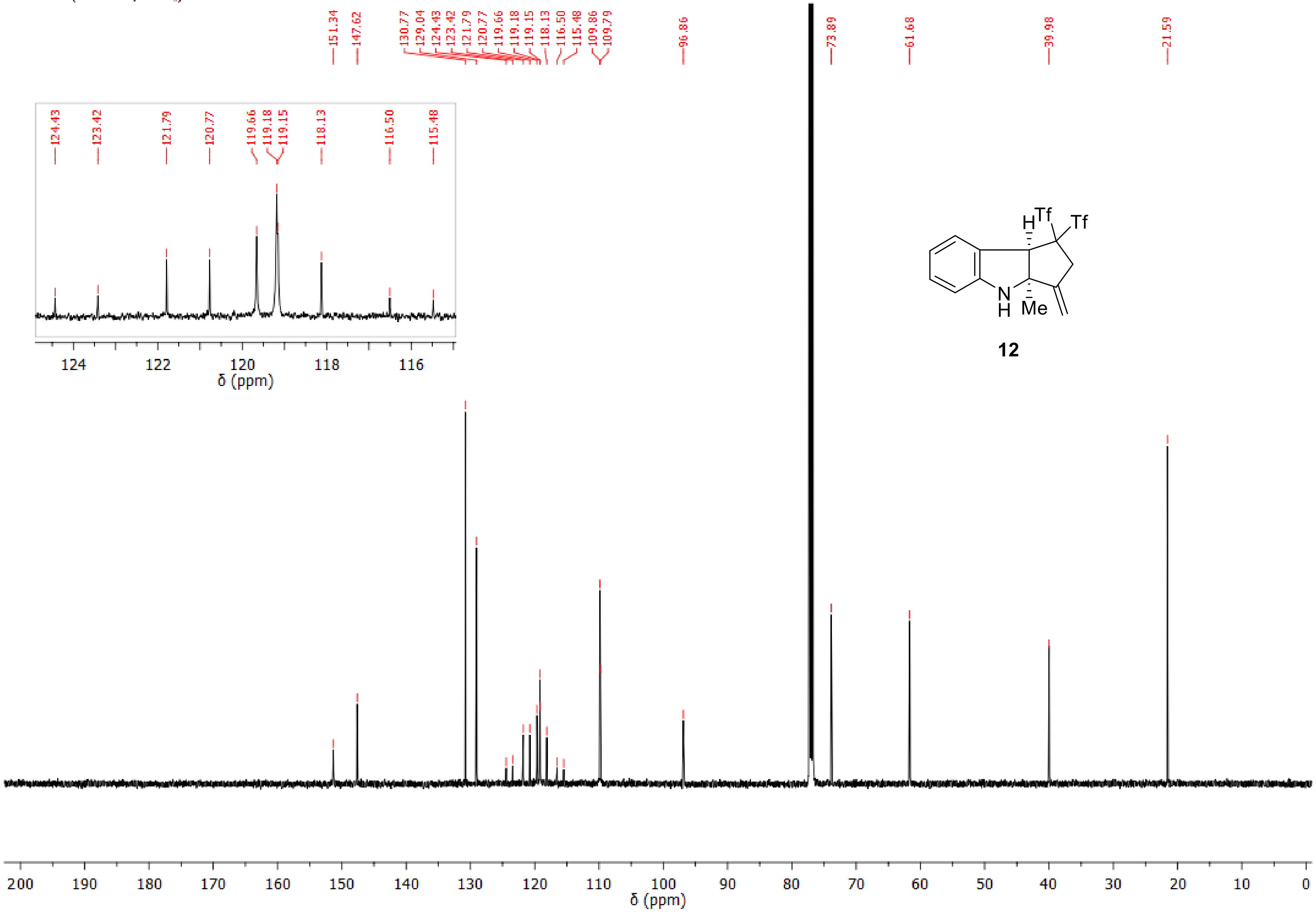

12

^{19}F NMR (282 MHz, $CDCl_3$)

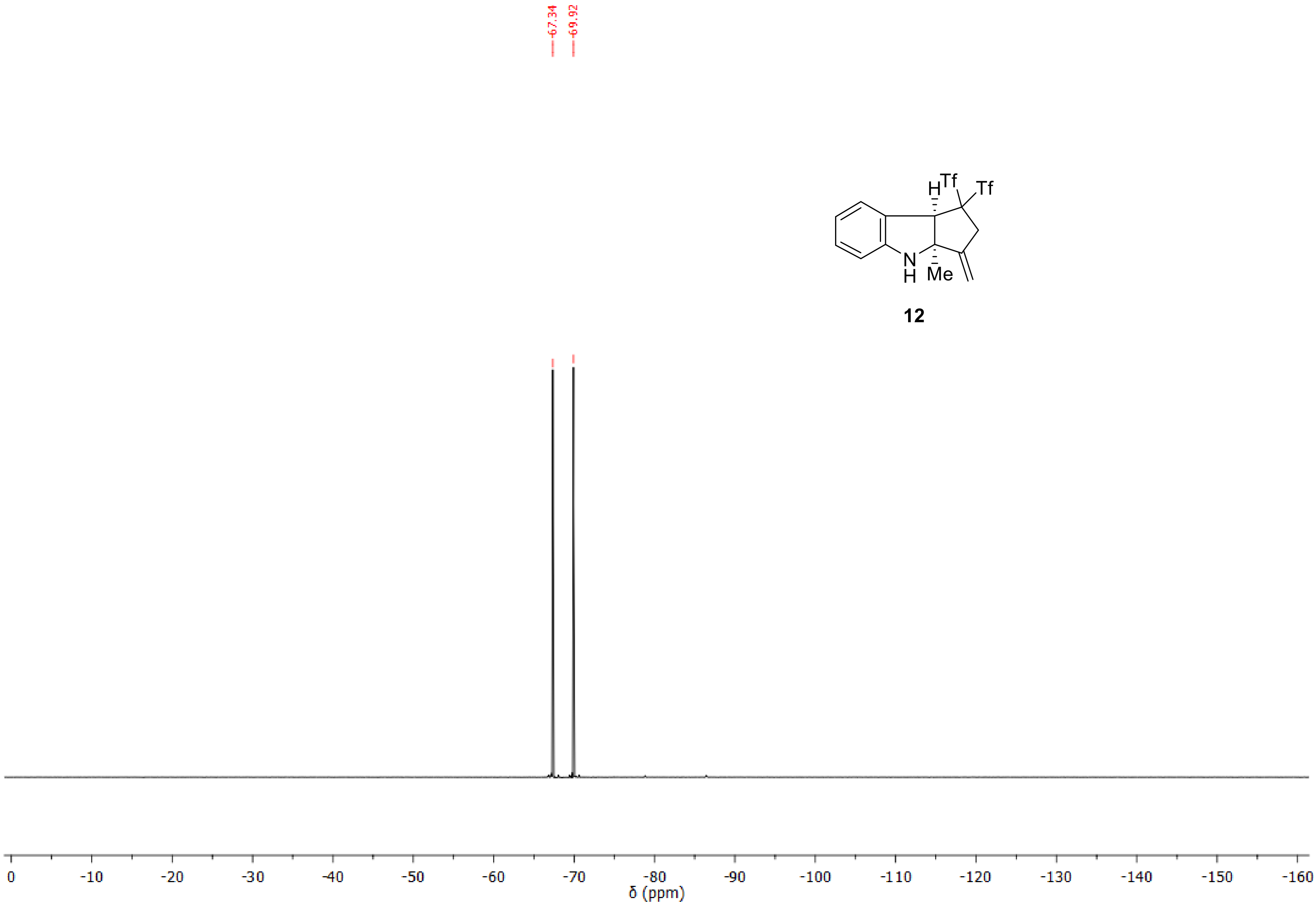

^{1}H NMR (500 MHz, Acetone)

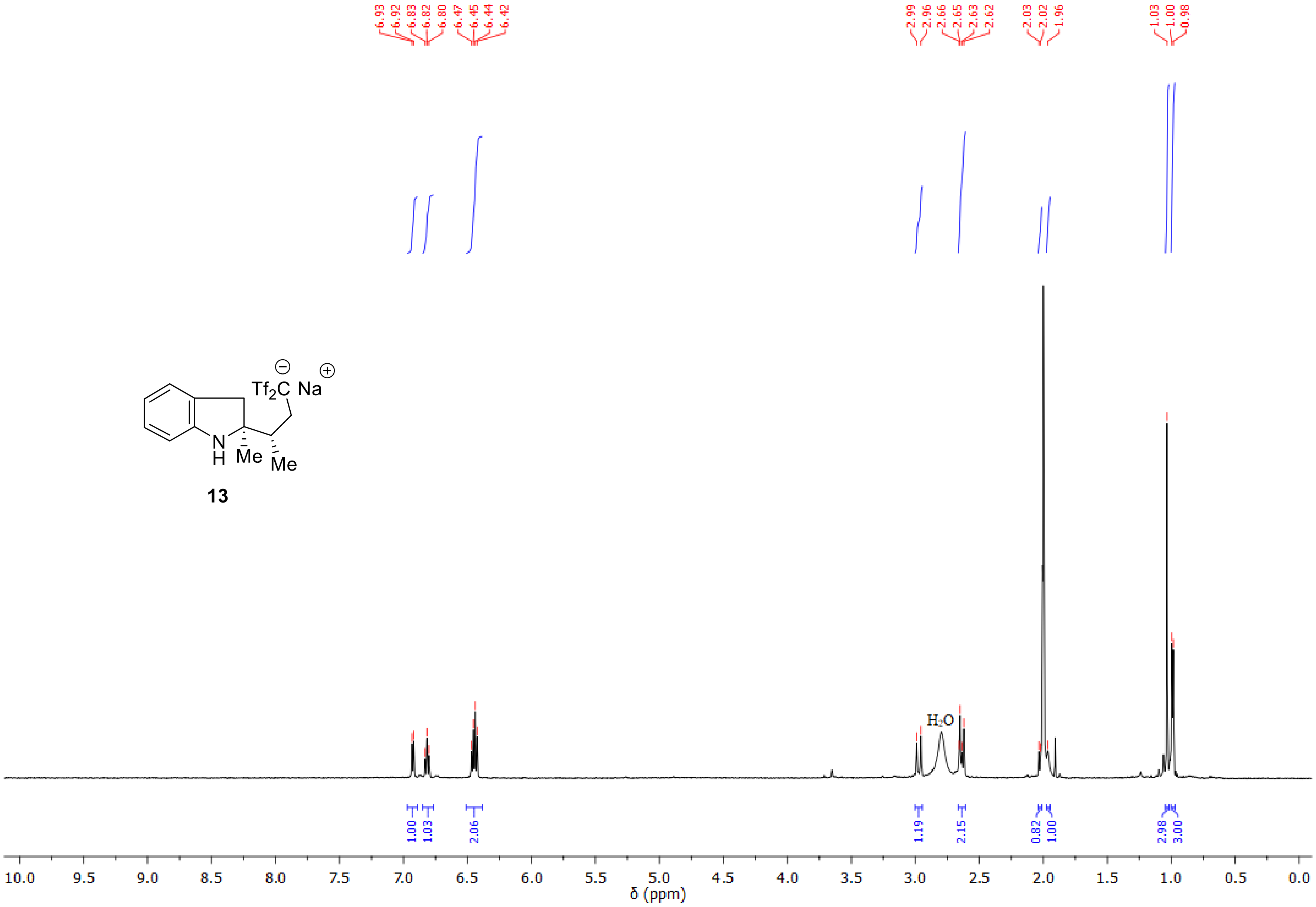

^{13}C NMR (125 MHz, Acetone)

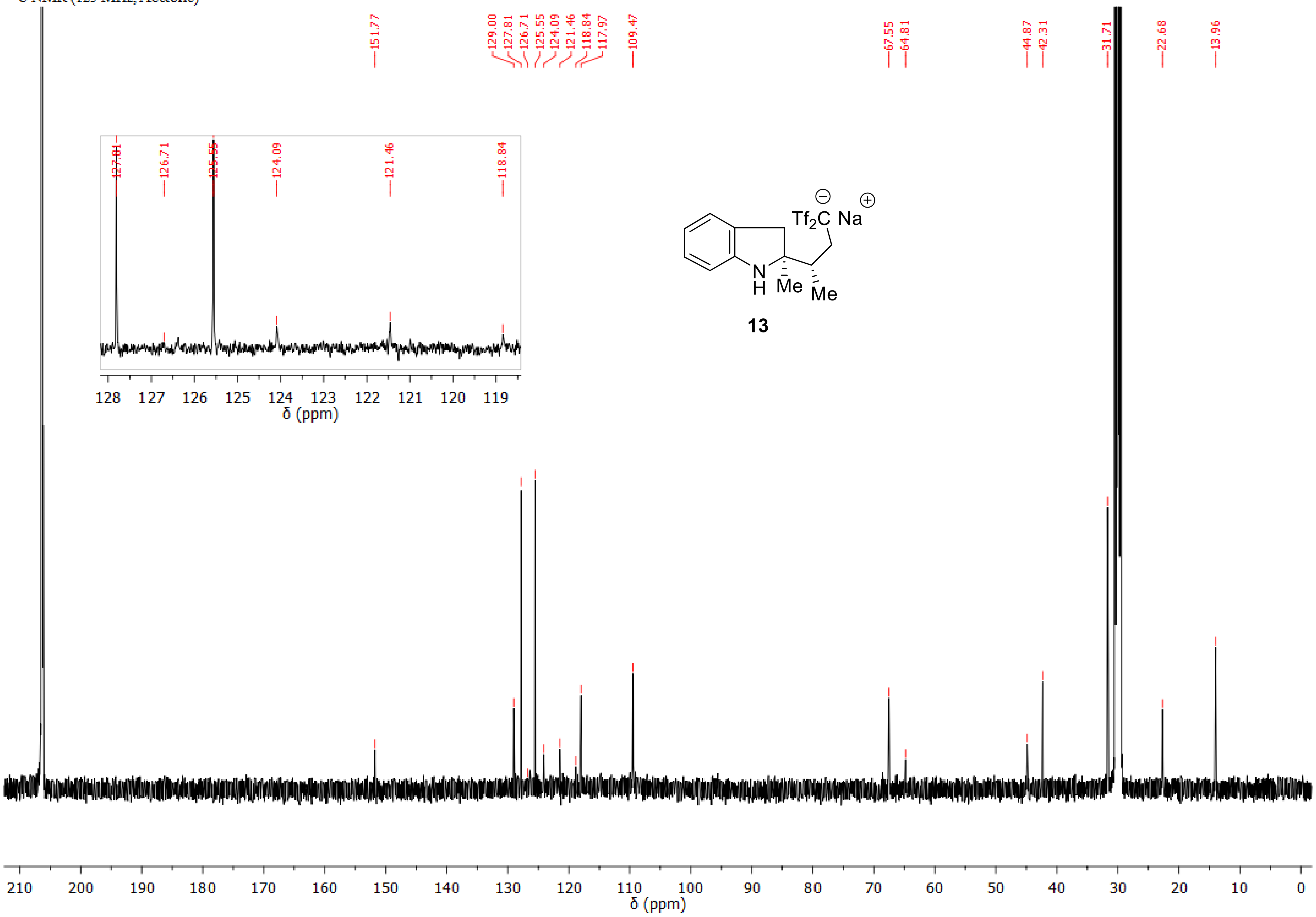

13

^{19}F NMR (282 MHz, Acetone)

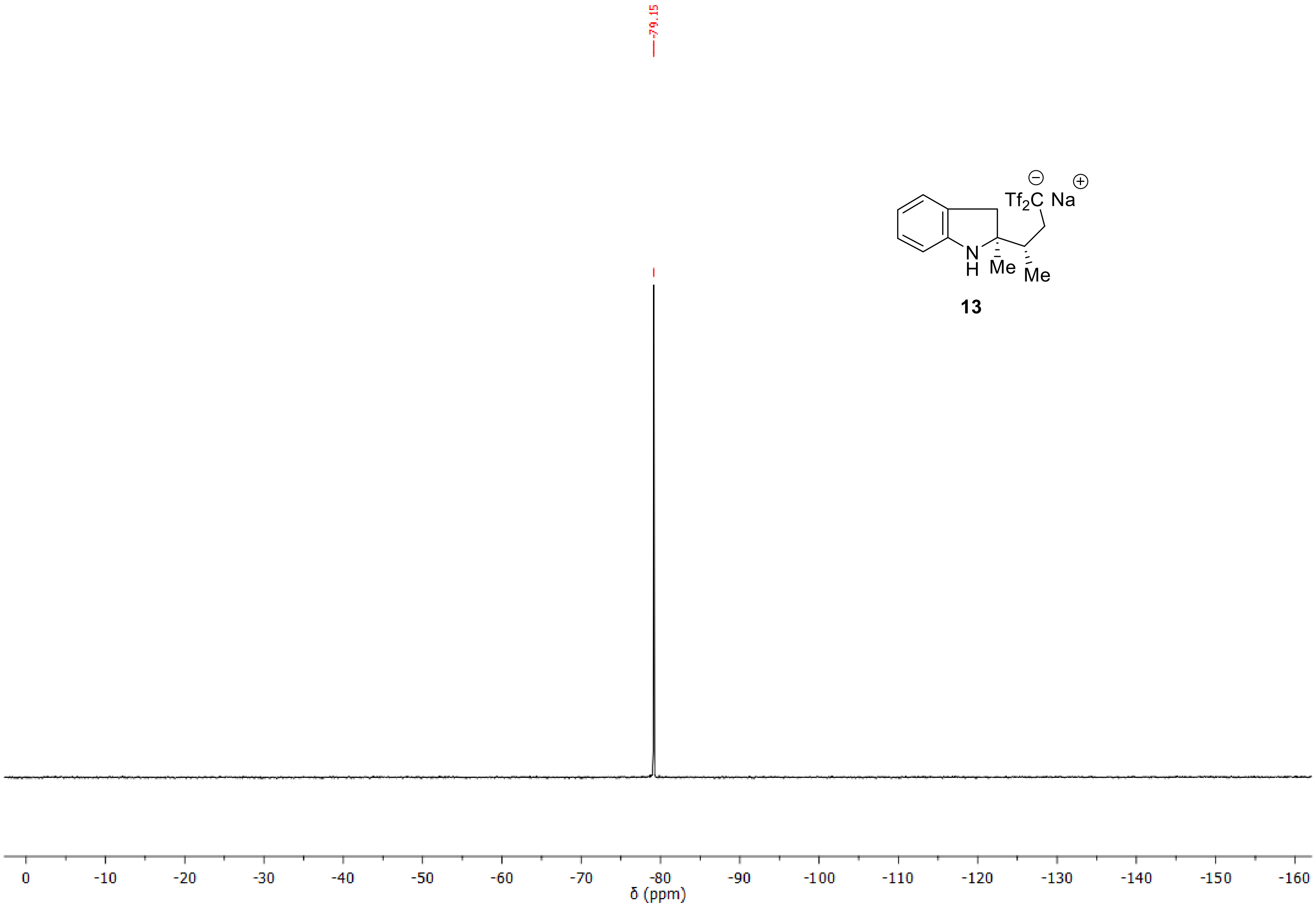

13

^{1}H NMR (500 MHz, $CDCl_3$)

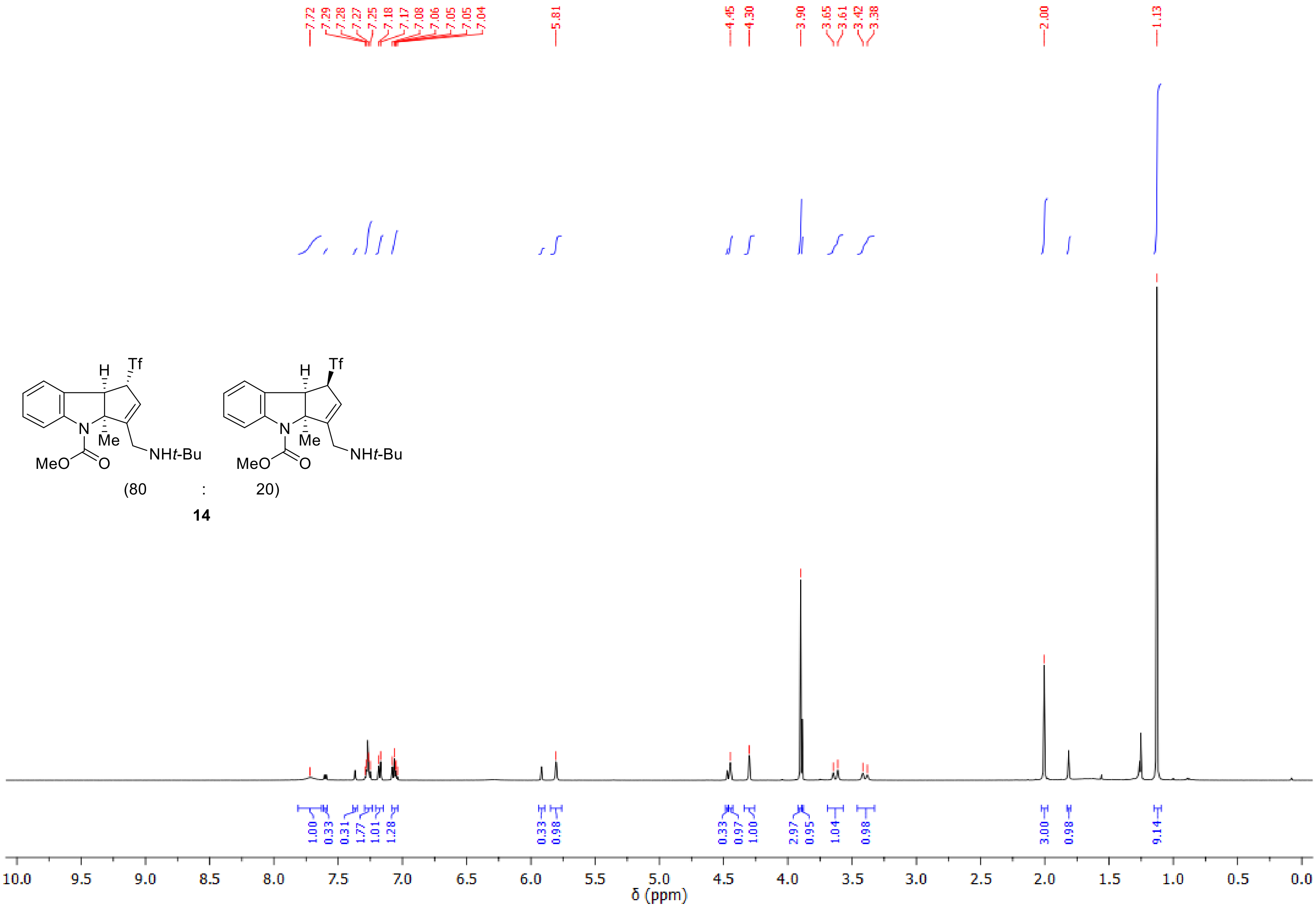

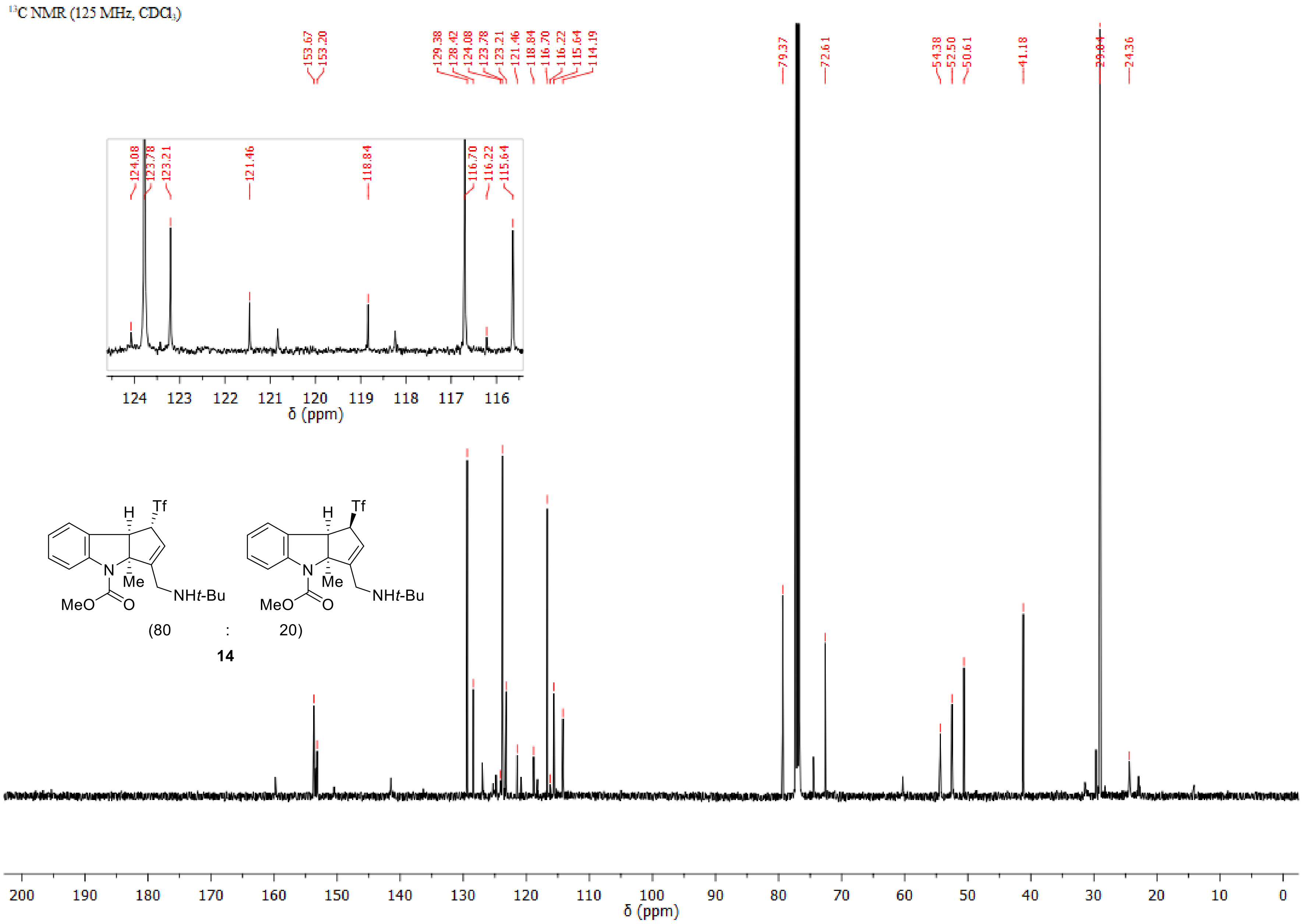
13C NMR (125 MHz, CDCl3)
153.67
153.20
129.38
128.42
124.08
123.78
123.21
121.46
118.84
116.70
116.22
115.64
114.19
79.37
72.61
54.38
52.50
50.61
41.18
29.84
24.36
Tf
H
N
Me
MeO
O
NHt-Bu
(80 : 20)
14
δ (ppm)

^{19}F NMR (282 MHz, $CDCl_3$)

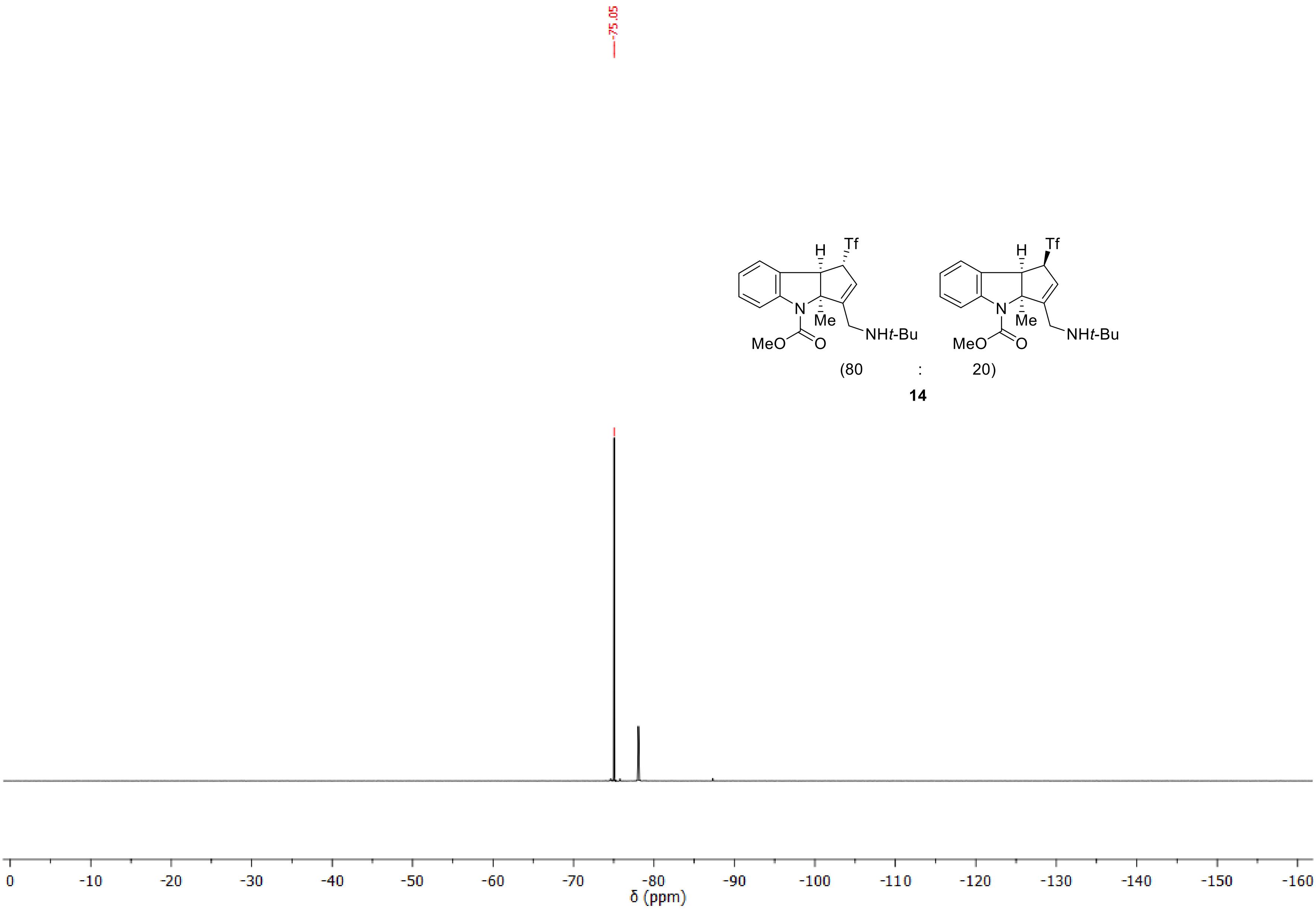

(80 : 20)

14

^1H NMR (500 MHz, $CDCl_3$)

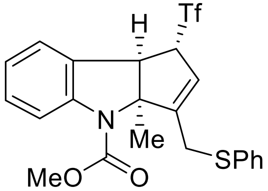

15

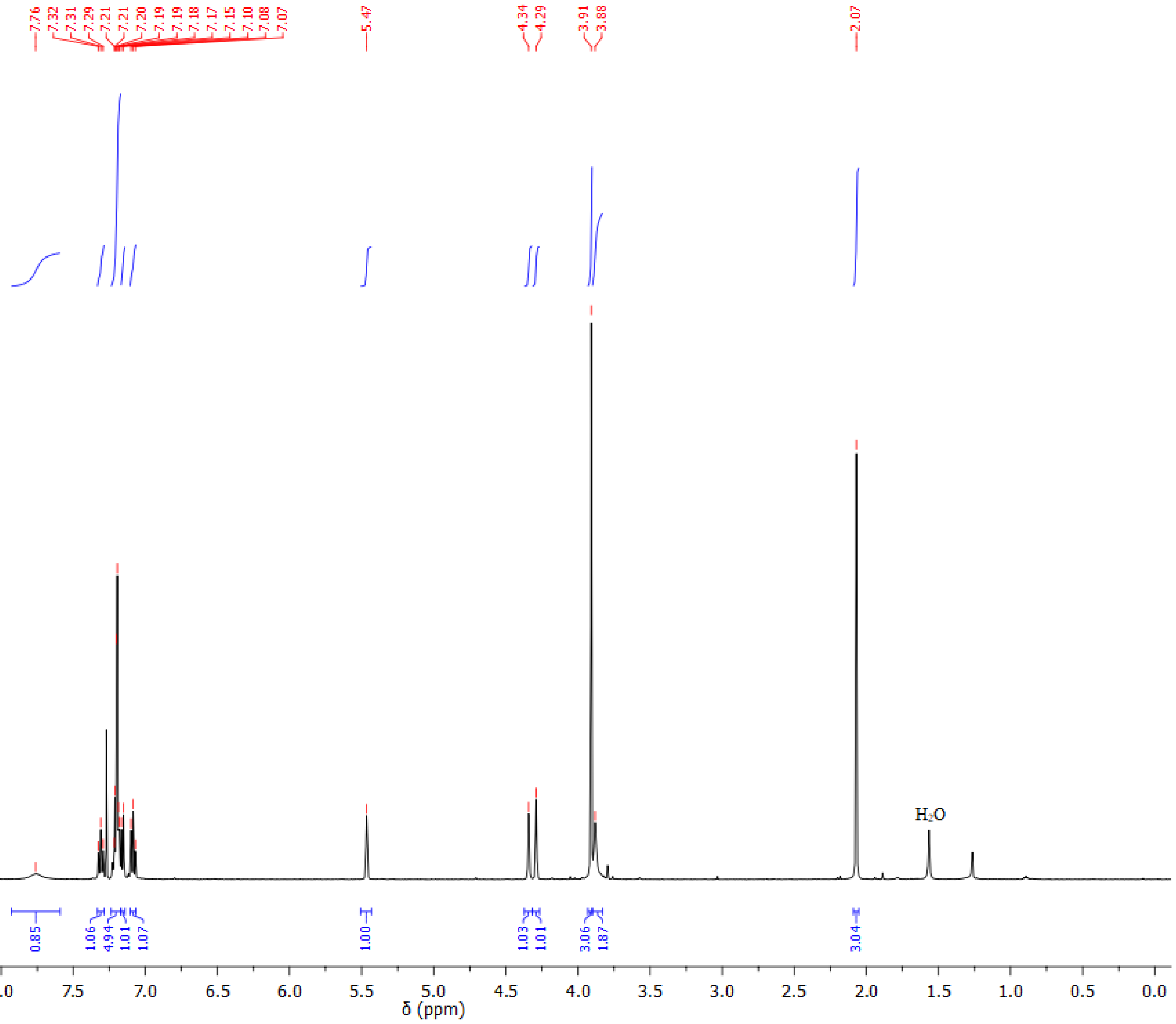

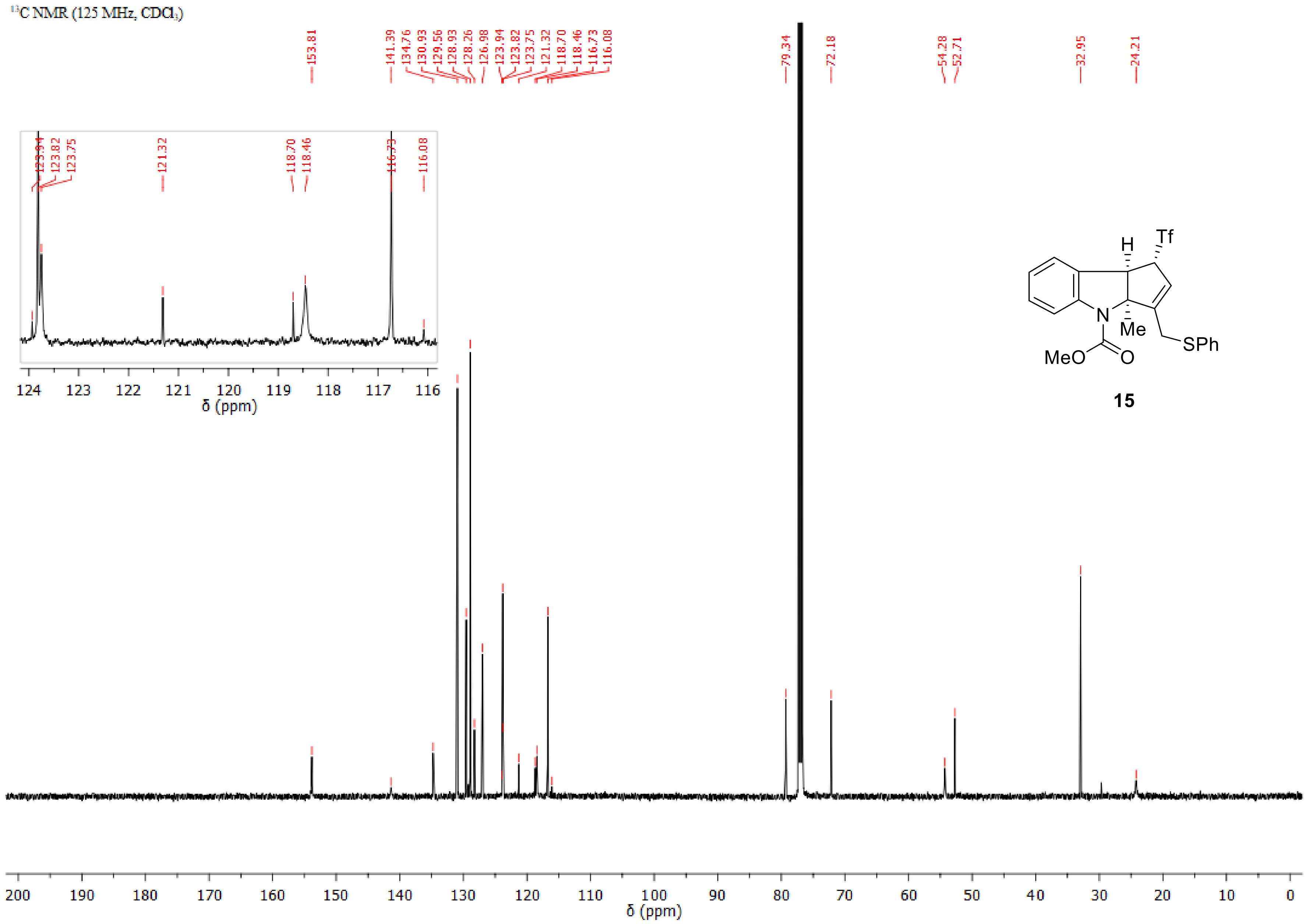
13C NMR (125 MHz, CDCl3)
153.81
141.39
134.76
130.93
129.56
128.93
128.26
126.98
123.94
123.82
123.75
121.32
118.70
118.46
116.73
116.08
79.34
72.18
54.28
52.71
32.95
24.21
H
Tf
N
Me
MeO
O
SPh
15
δ (ppm)

^{19}F NMR (282 MHz, $CDCl_3$)

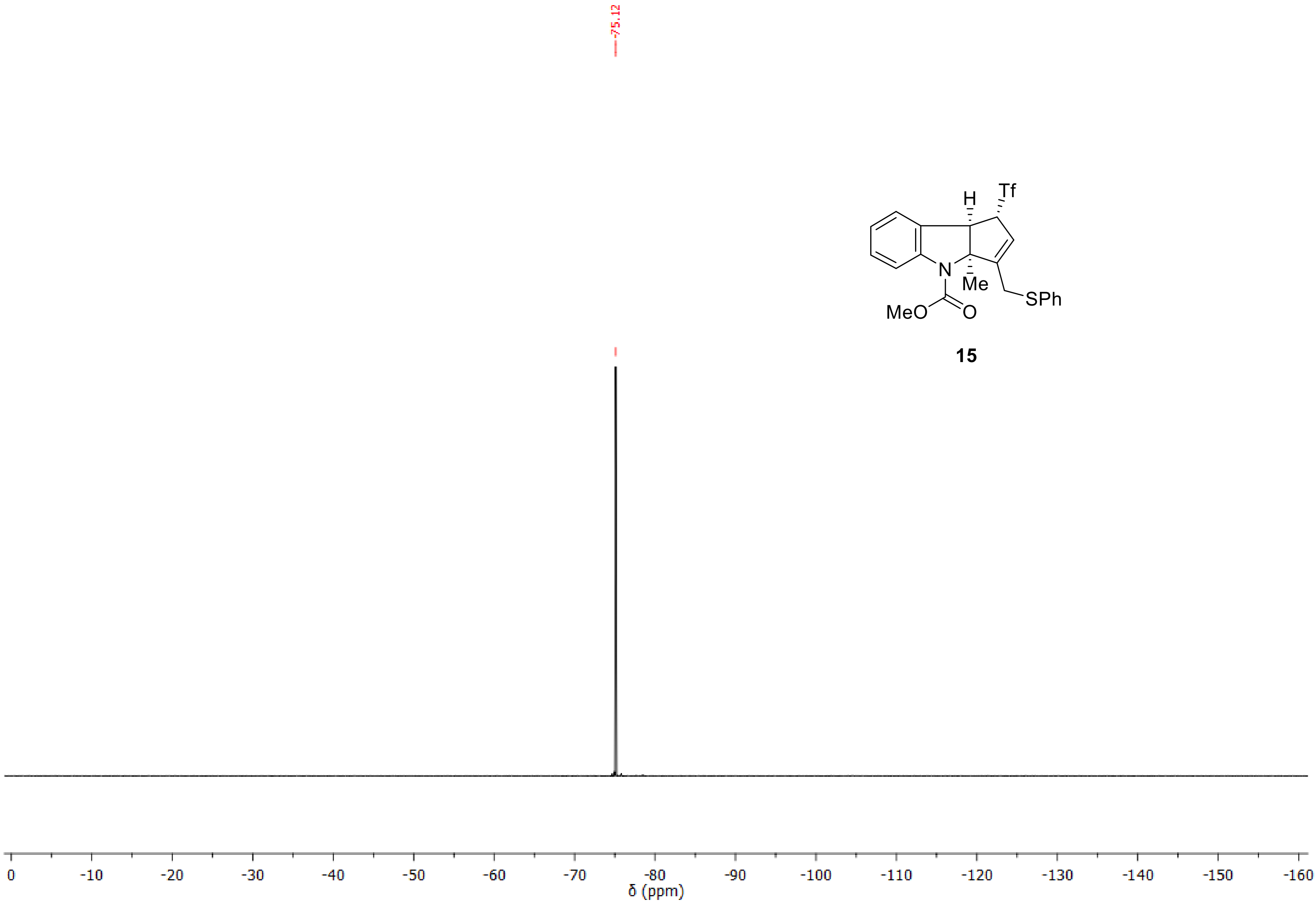

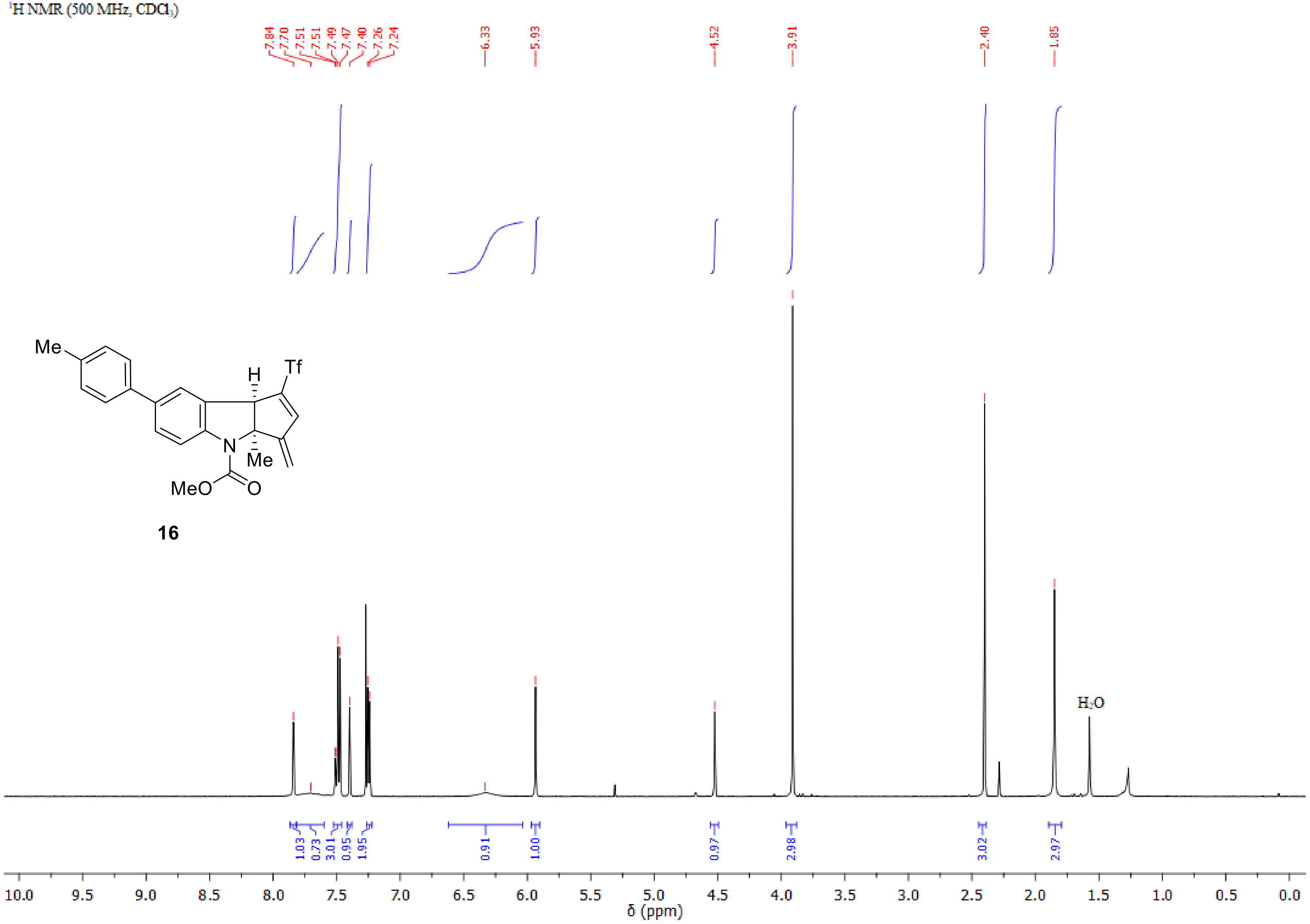

¹H NMR (500 MHz, CDCl₃)
7.84
7.70
7.51
7.51
7.49
7.47
7.40
7.26
7.24
6.33
5.93
4.52
3.91
2.40
1.85
Me
H
Tf
N
Me
MeO
O
16
H₂O
1.03
0.73
3.01
0.95
1.95
0.91
1.00
0.97
2.98
3.02
2.97
10.0
9.5
9.0
8.5
8.0
7.5
7.0
6.5
6.0
5.5
5.0
4.5
4.0
3.5
3.0
2.5
2.0
1.5
1.0
0.5
0.0
δ (ppm)

^{13}C NMR (125 MHz, $CDCl_3$)

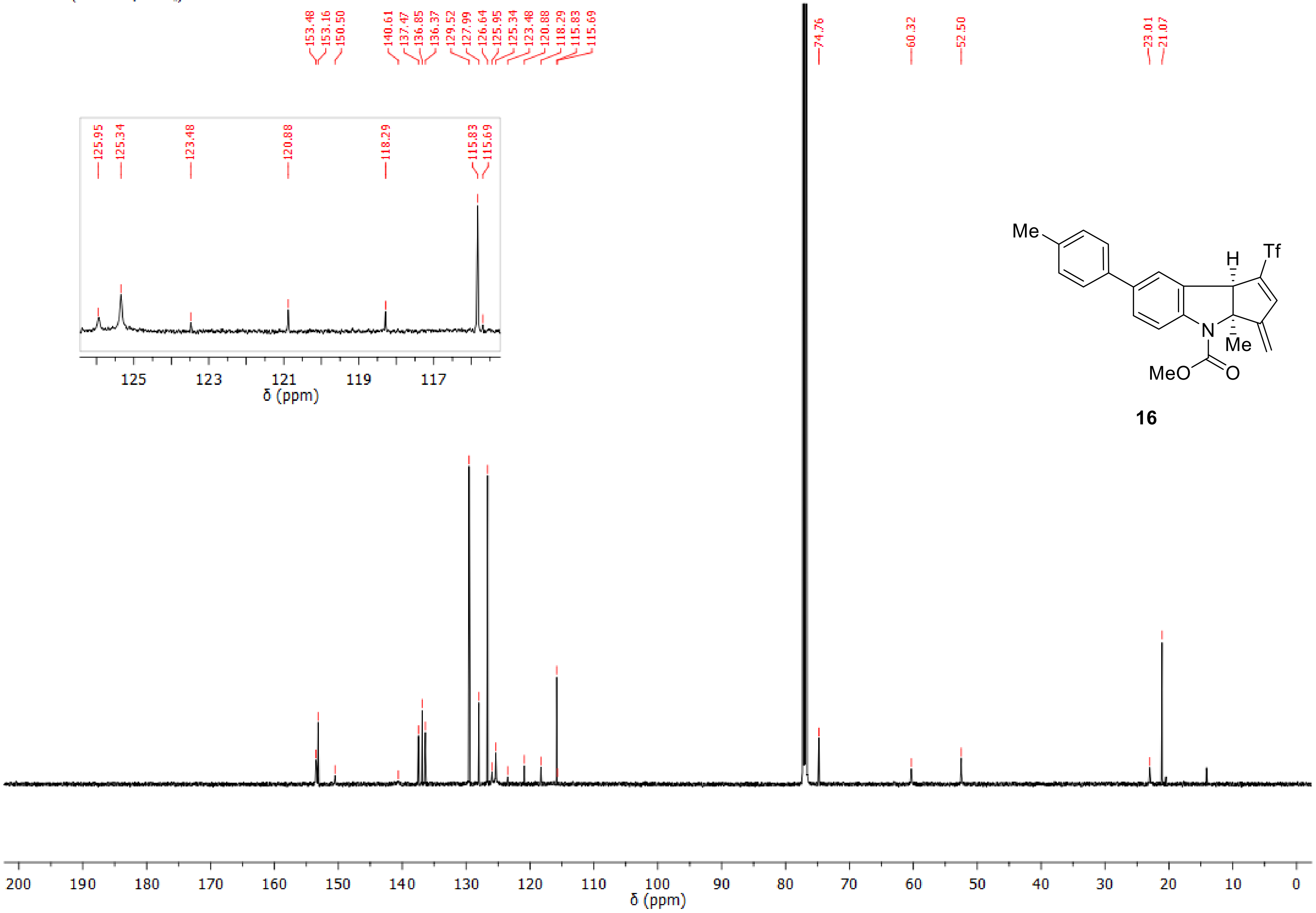

16

^{19}F NMR (282 MHz, $CDCl_3$)

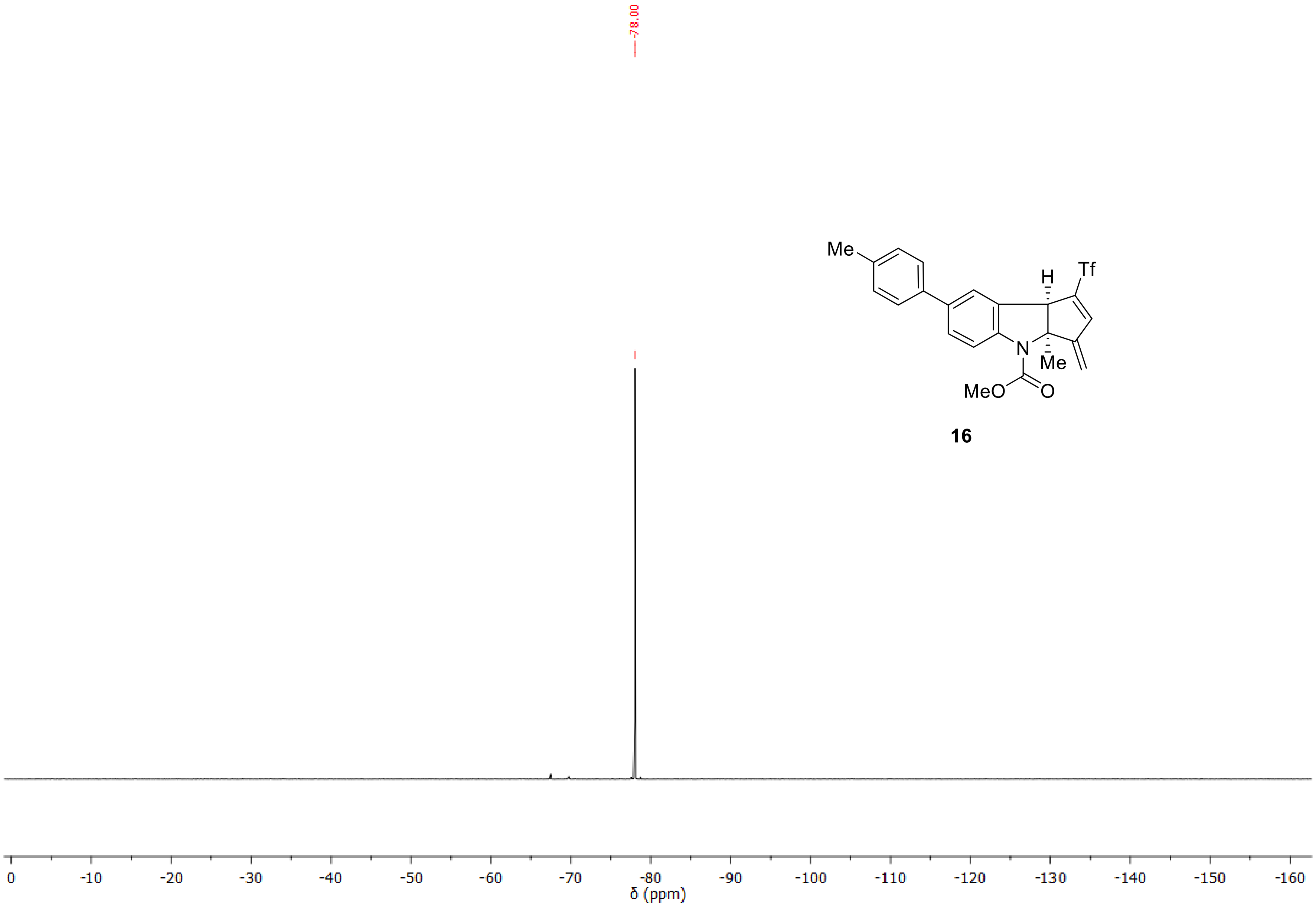

16

^{1}H NMR (500 MHz, $CDCl_3$)

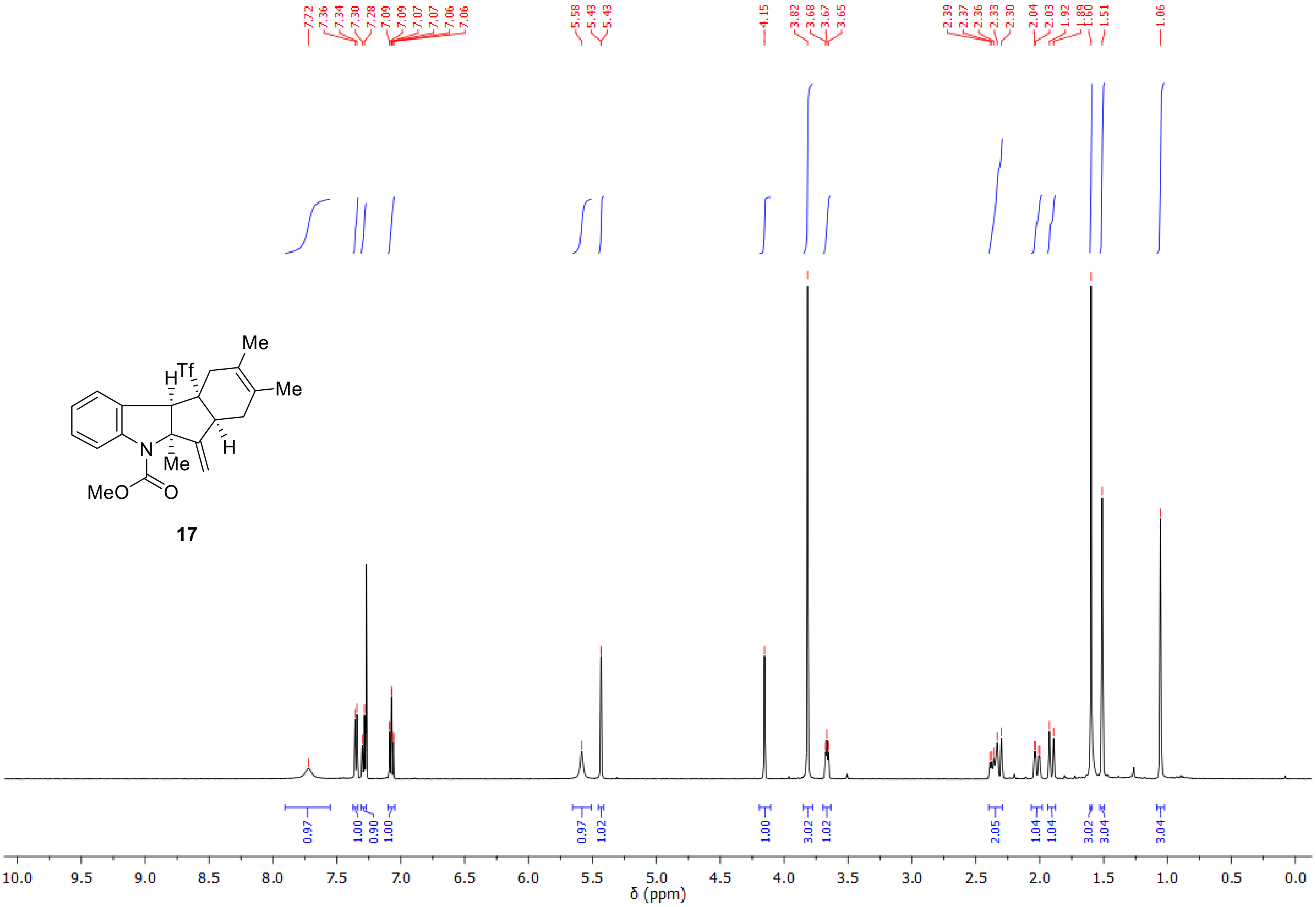

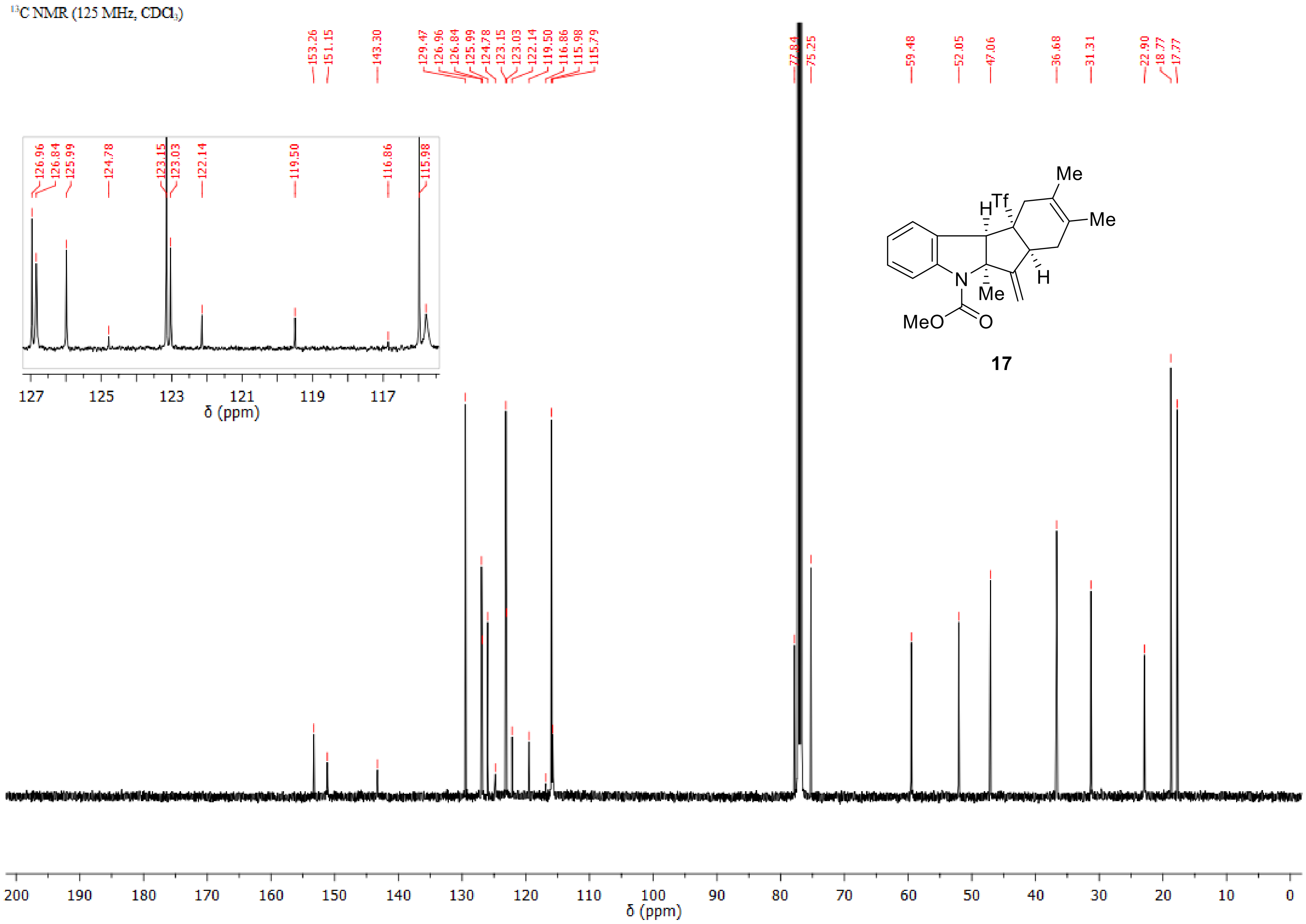
13C NMR (125 MHz, CDCl3)
153.26
151.15
143.30
129.47
126.96
126.84
125.99
124.78
123.15
123.03
122.14
119.50
116.86
115.98
115.79
77.84
75.25
59.48
52.05
47.06
36.68
31.31
22.90
18.77
17.77
δ (ppm)
Me
H
Tf
Me
N
Me
H
MeO
O
17

^{19}F NMR (282 MHz, $CDCl_3$)

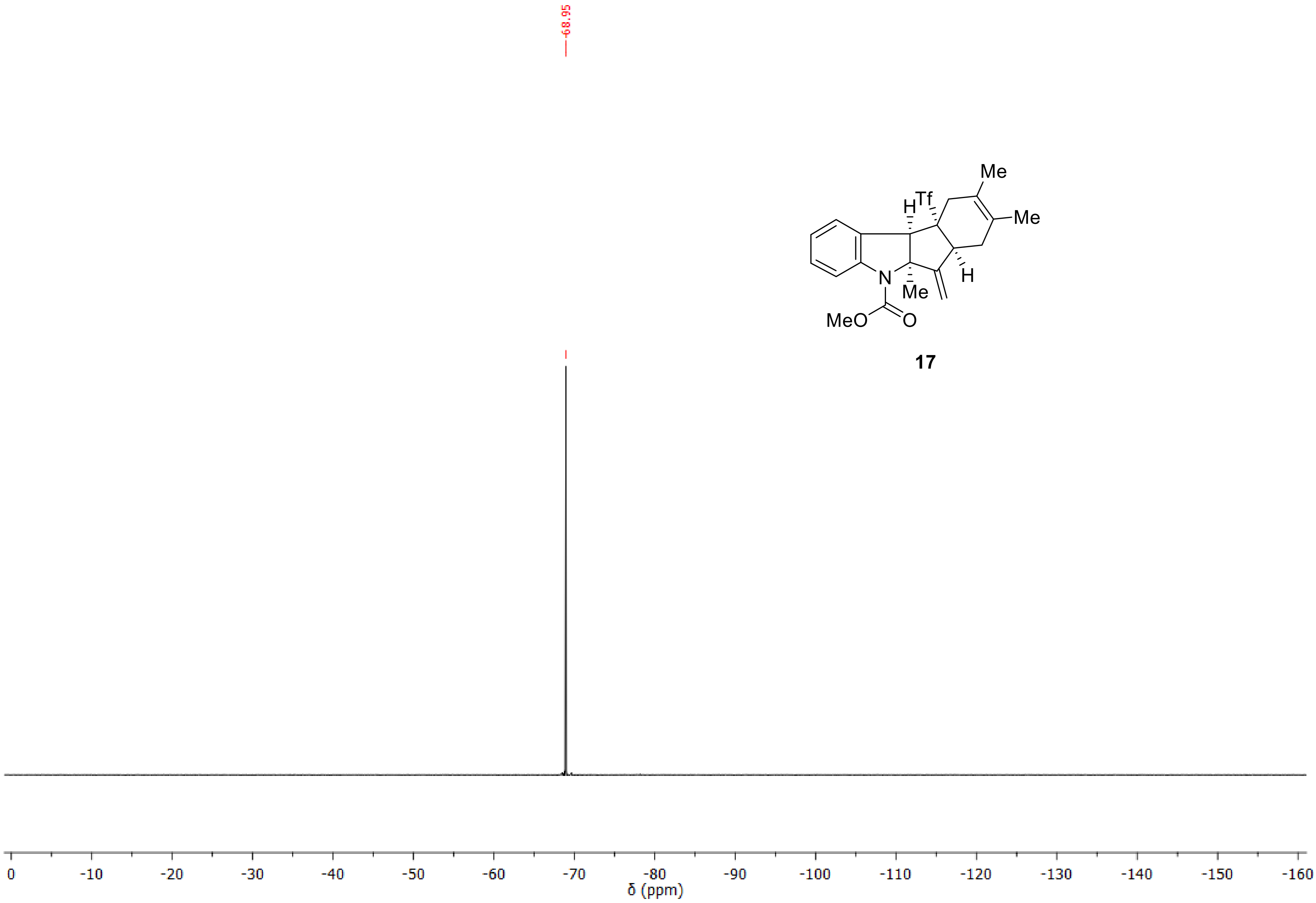

17

^1H NMR (500 MHz, $CDCl_3$)

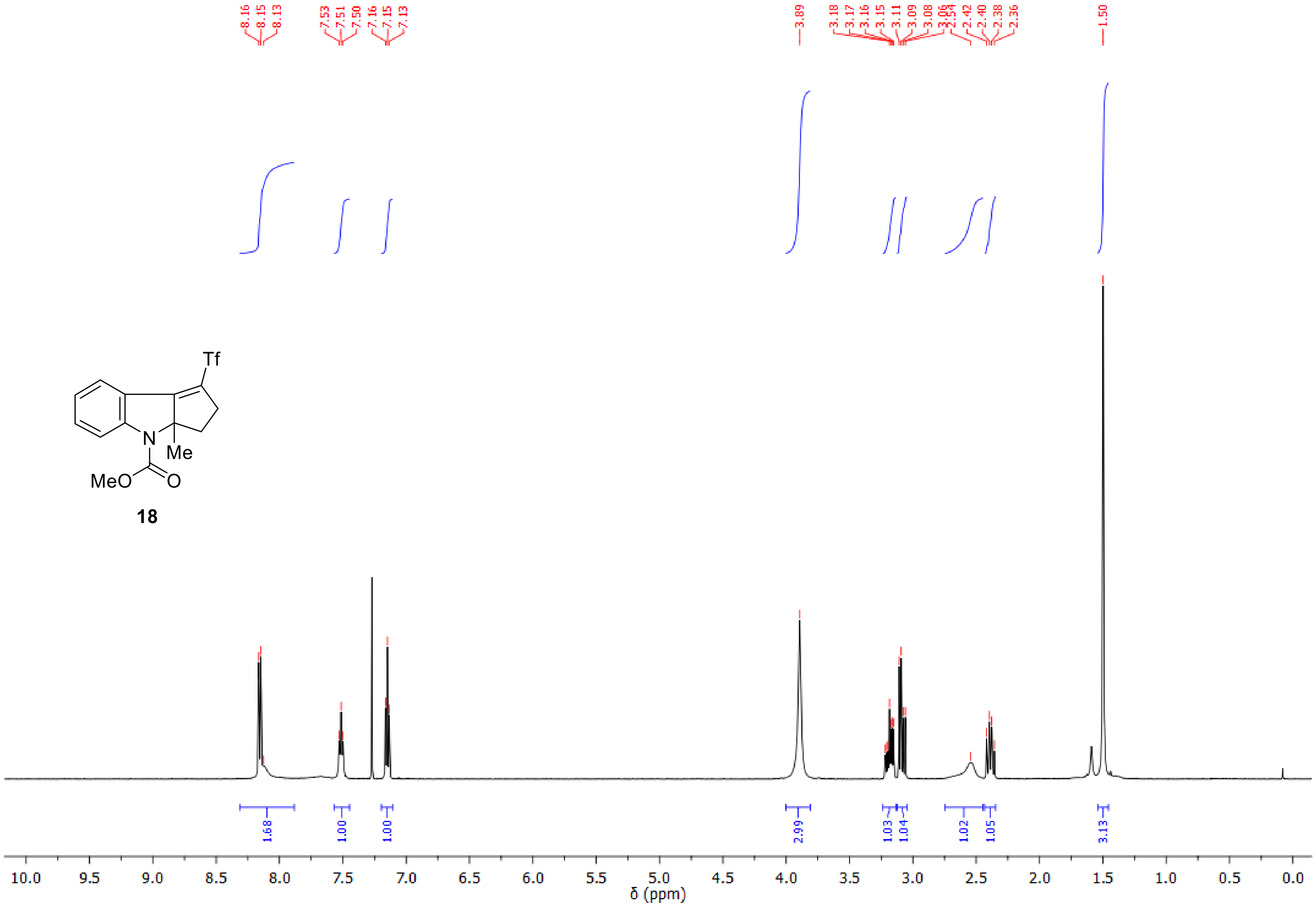

^{13}C NMR (125 MHz, $CDCl_3$)

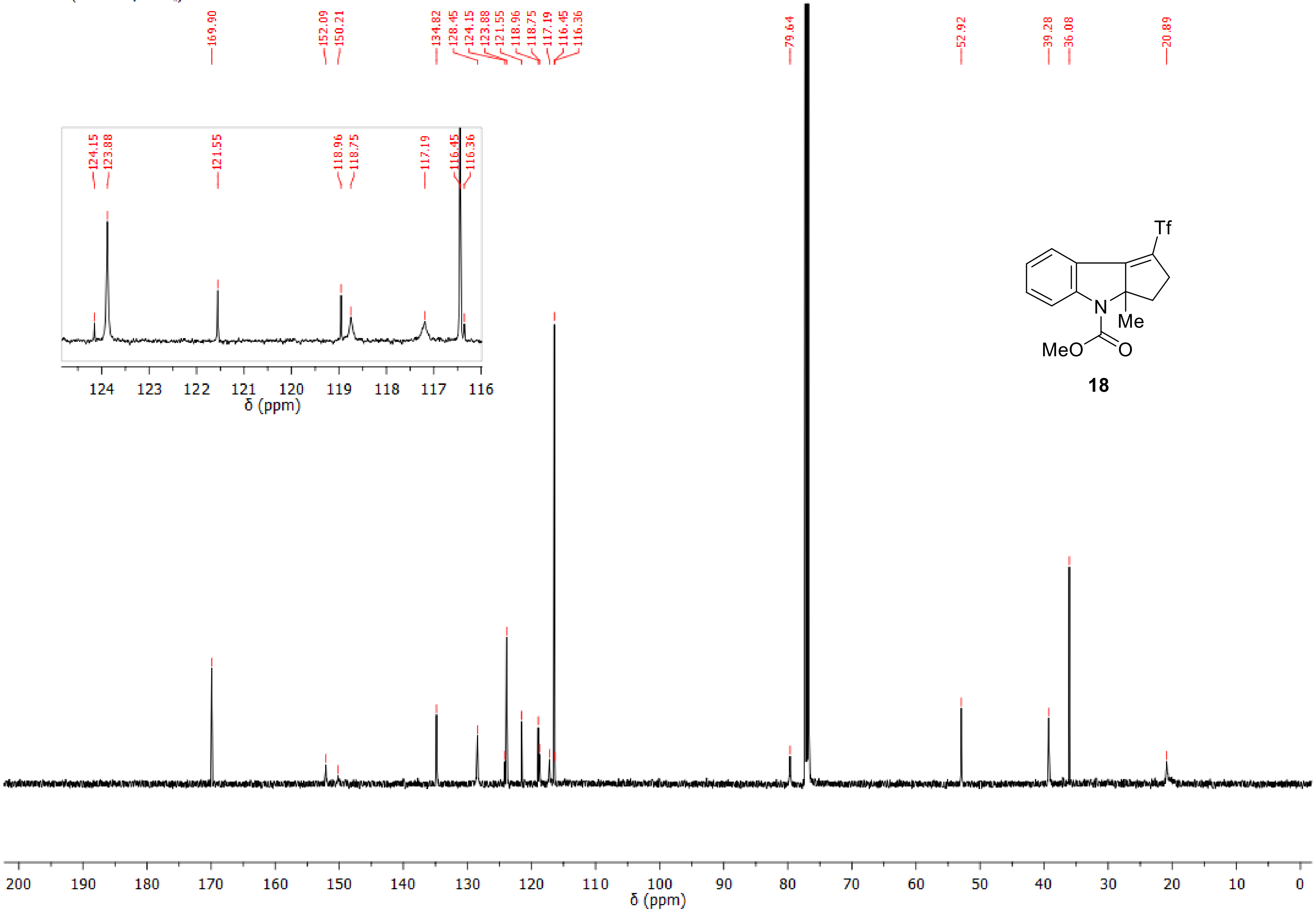

18

^{19}F NMR (282 MHz, $CDCl_3$)

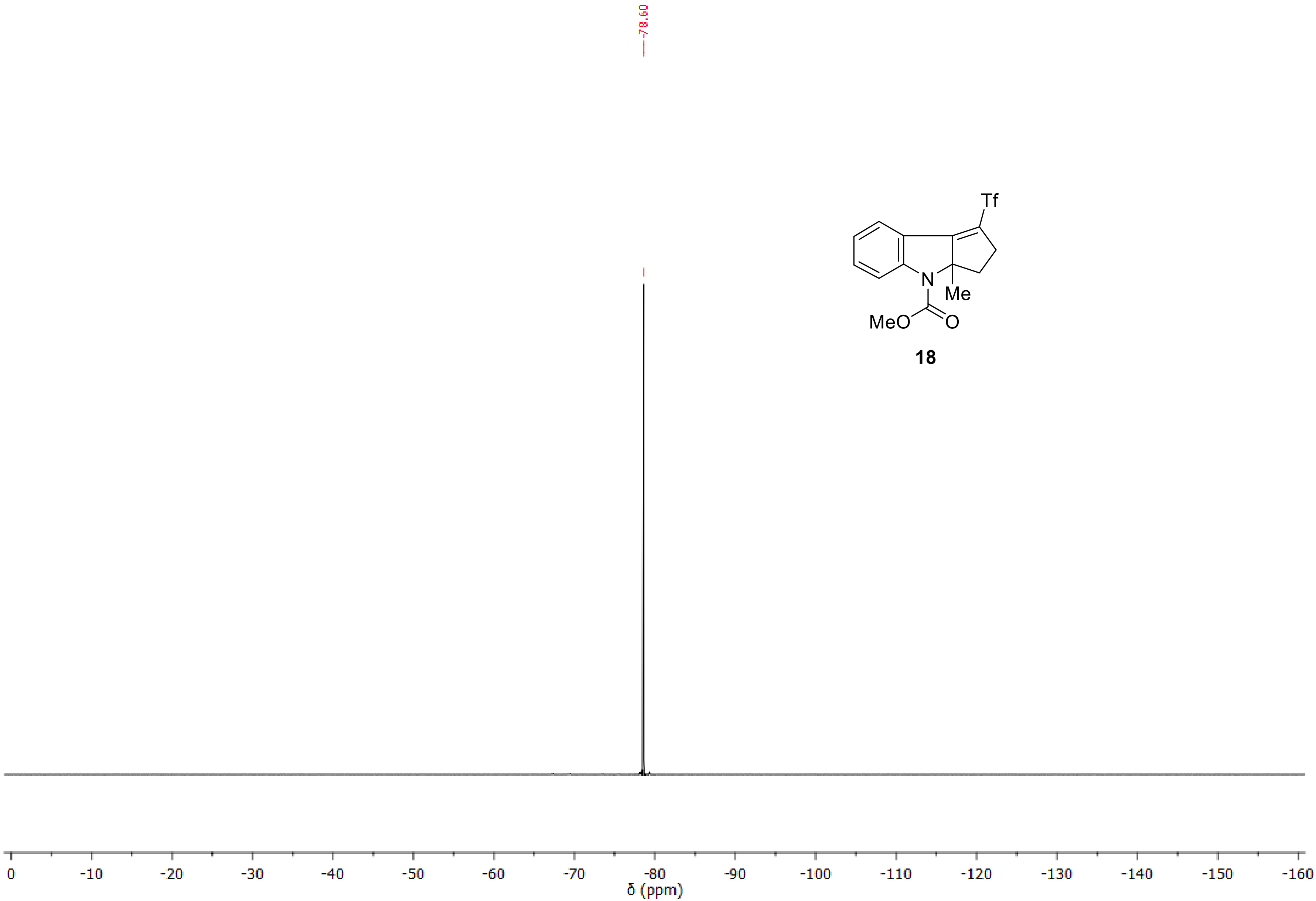

^{1}H NMR (500 MHz, Acetone)

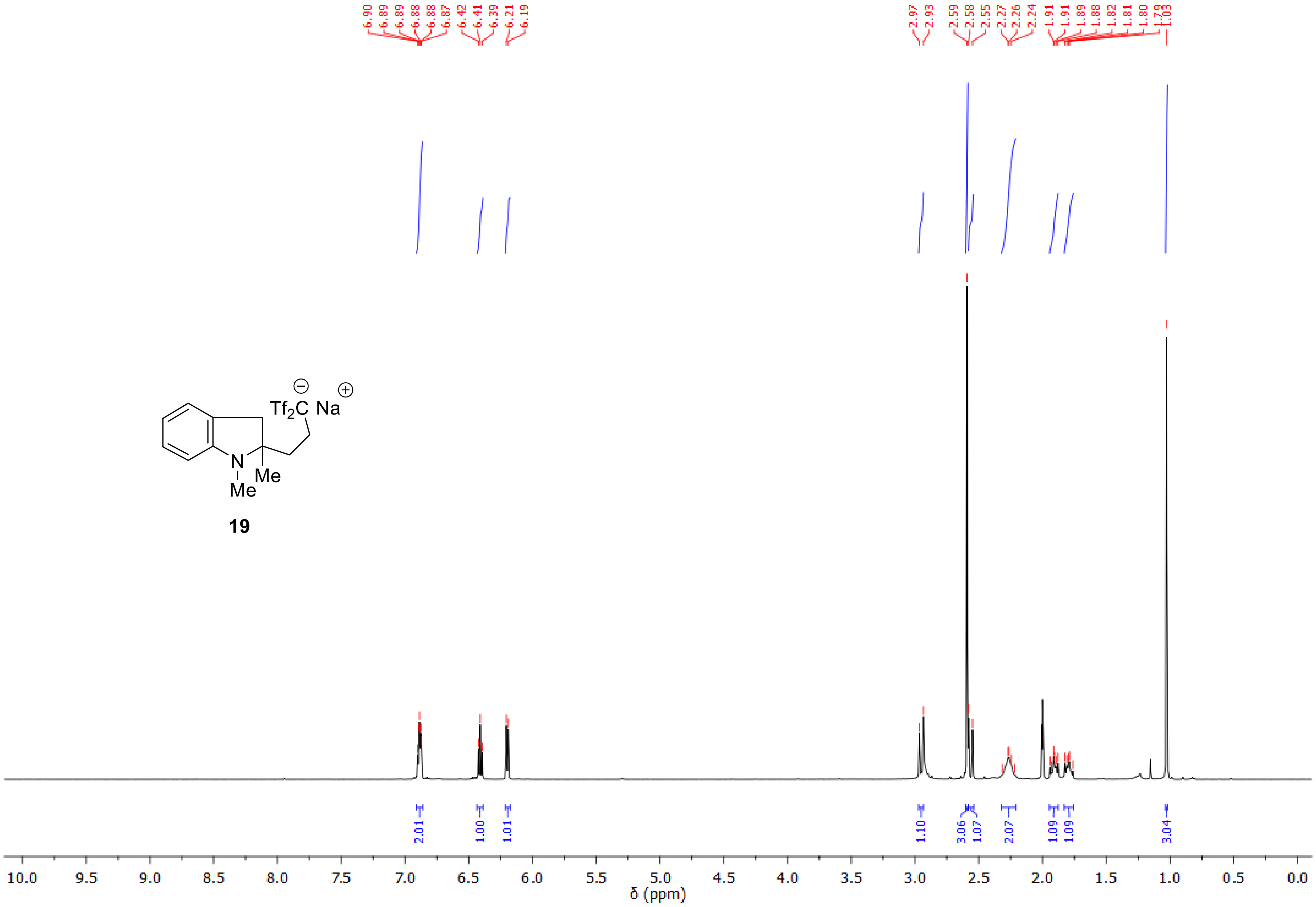

19

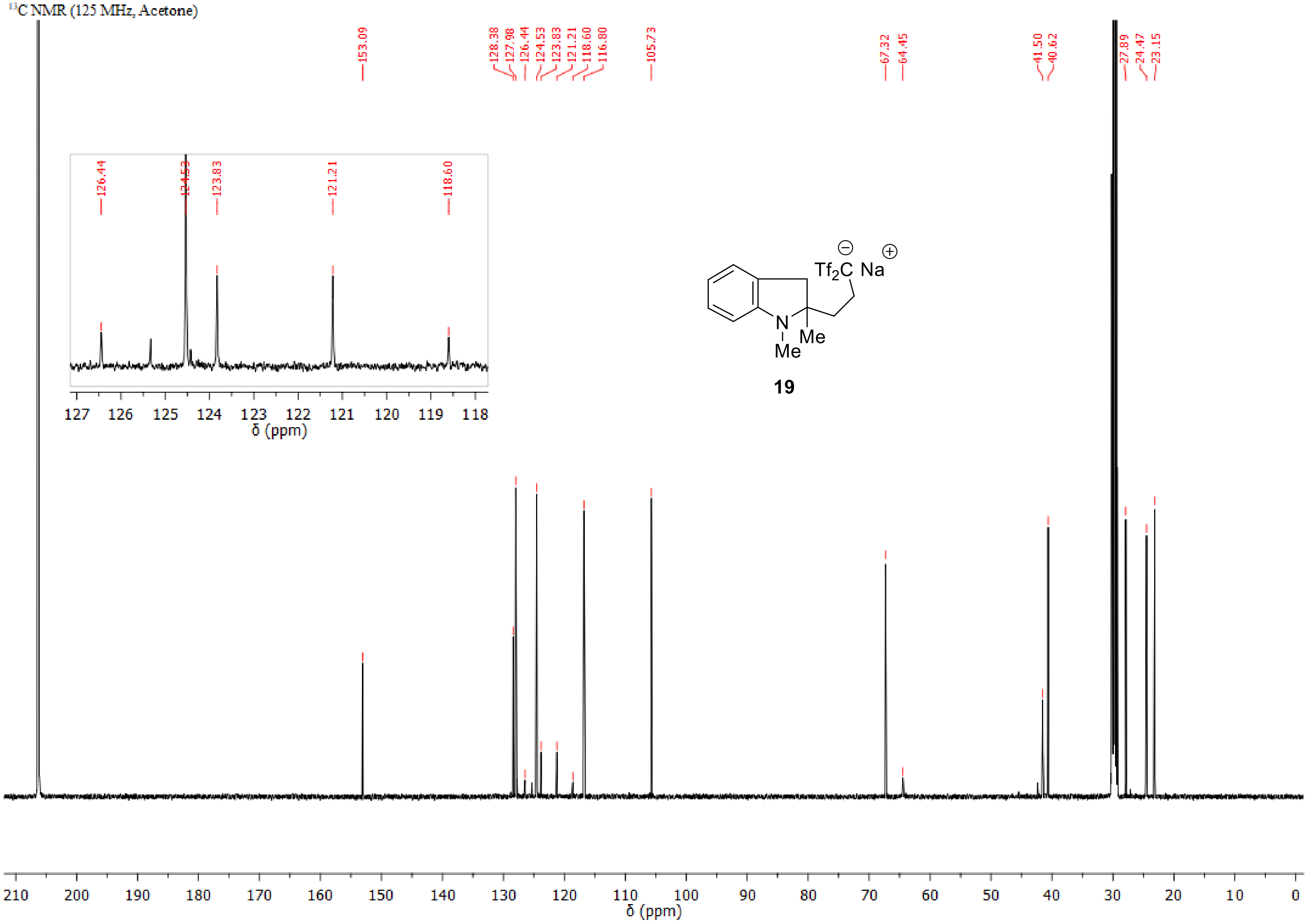

13C NMR (125 MHz, Acetone)
153.09
128.38
127.98
126.44
124.53
123.83
121.21
118.60
116.80
105.73
67.32
64.45
41.50
40.62
27.89
24.47
23.15
δ (ppm)
Tf2C Na
N
Me
Me

19

^{19}F NMR (282 MHz, Acetone)

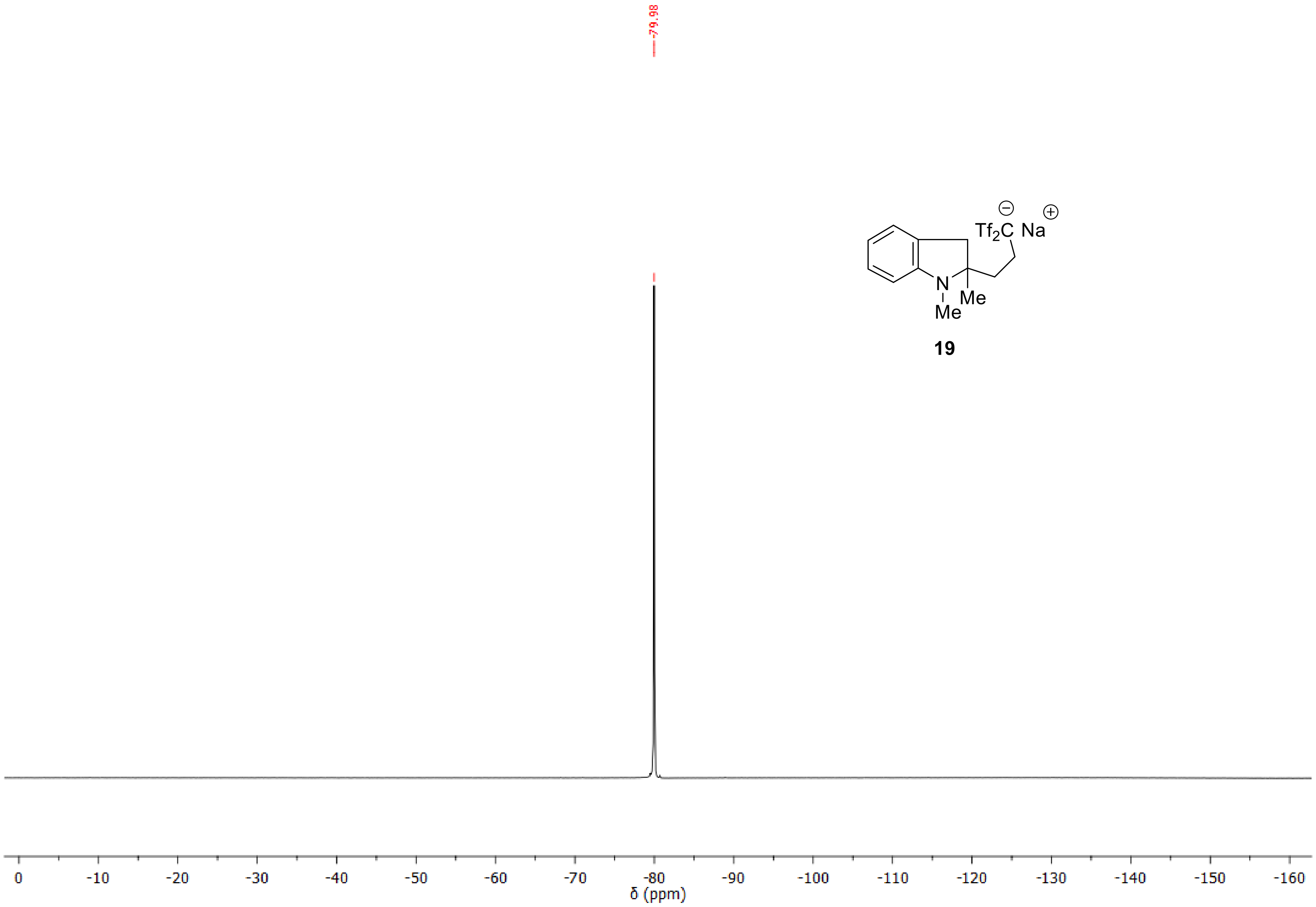

19

DFT calculations

All calculations were carried out by using *Gaussian 09* program, revision D.01.[i] Molecular geometries were optimized and characterized by frequency analysis using a hybrid density functional (M06-2X)[ii] and the 6-31+G(d) basis set as implemented in the *Gaussian 09* program. Single imaginary frequency was obtained in all transition states, which were supported by the intrinsic reaction coordinate (IRC) calculations using the 'lqa' keyword. Each geometry of intermediates was obtained by optimization of the IRC geometries.

Table S1. Coordinates and energies for optimized geometry of **2a**

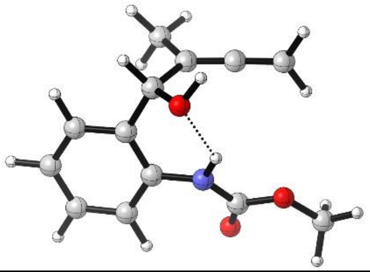

Center Number	Atomic Number	Atomic Type	Coordinates (Angstroms) X	Y	Z
1	6	0	1.209386	1.510792	-0.721716
2	6	0	1.622655	0.084172	-0.419344
3	6	0	2.440425	-2.495451	0.293631
4	6	0	2.968433	-0.202819	-0.193363
5	6	0	0.674884	-0.950558	-0.295732
6	6	0	1.092333	-2.238686	0.053731
7	6	0	3.387079	-1.481443	0.169839
8	8	0	0.412513	1.511430	-1.898563
9	6	0	0.497984	2.137482	0.477634
10	6	0	-0.764738	2.477909	0.399235
11	7	0	-0.677532	-0.663073	-0.563987
12	6	0	-1.752201	-1.250639	0.029520
13	8	0	-2.885191	-0.669273	-0.413292
14	8	0	-1.730750	-2.161756	0.837273
15	6	0	-4.096511	-1.192186	0.137541
16	6	0	1.334275	2.323338	1.719936
17	6	0	-2.029766	2.799446	0.293305
18	1	0	2.124933	2.094029	-0.898249
19	1	0	2.747922	-3.500386	0.567529
20	1	0	3.697879	0.597605	-0.294581
21	1	0	0.358686	-3.030579	0.139207
22	1	0	4.438910	-1.682045	0.347689
23	1	0	0.044868	2.401603	-2.024381
24	1	0	-0.859910	0.150046	-1.146728
25	1	0	-4.114351	-1.049998	1.220034
26	1	0	-4.192335	-2.254534	-0.095583
27	1	0	-4.897460	-0.625588	-0.334252
28	1	0	0.775350	2.851076	2.494697
29	1	0	2.240174	2.895676	1.487693
30	1	0	1.650884	1.351928	2.115988
31	1	0	-2.811575	2.085634	0.545383
32	1	0	-2.339261	3.785699	-0.045178

E(RM062X) = -784.505724922

Zero-point correction = 0.261950 (Hartree/Particle)

Sum of electronic and thermal Enthalpies = -784.213291

Sum of electronic and thermal Free Energies = -784.314103

Table S2. Coordinates and energies for optimized geometry of $Tf_2C{=}CH_2$

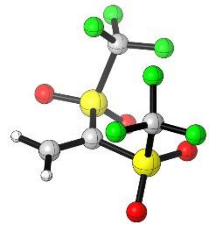

Center Number	Atomic Number	Atomic Type	Coordinates (Angstroms) X	Y	Z
1	6	0	0.216714	-1.674729	1.663132
2	1	0	-0.622436	-1.912748	2.311548
3	1	0	1.219302	-1.944192	1.986454
4	6	0	0.012864	-1.077705	0.488444
5	16	0	-1.677790	-0.818587	-0.058444
6	8	0	-1.825569	-1.230527	-1.439105
7	8	0	-2.536645	-1.321287	0.999658
8	16	0	1.411374	-0.710903	-0.568632
9	8	0	0.948841	-0.266224	-1.867238
10	8	0	2.406635	-1.750872	-0.392972
11	6	0	2.116354	0.796226	0.278989
12	9	0	3.113797	1.252158	-0.458216
13	9	0	1.175424	1.724005	0.390944
14	9	0	2.557230	0.465552	1.484317
15	6	0	-1.889679	1.034293	-0.012710
16	9	0	-1.509184	1.486399	1.173938
17	9	0	-1.166912	1.600910	-0.963496
18	9	0	-3.172334	1.294484	-0.202447

E(RM062X) = -1849.36746136
Zero-point correction = 0.083638 (Hartree/Particle)
Sum of electronic and thermal Enthalpies = -1849.256781
Sum of electronic and thermal Free Energies = -1849.351150

To evaluate the importance of intermolecular hydrogen bonding in the initial electrophilic attack of $Tf_2C{=}CH_2$ on the allenol **2a**, two transition states **TS-1** and **TS-1B** were computed. Among these, **TS-1** bearing an intramolecular hydrogen bond was 3.7 kcal mol^{-1} more stable than **TS-1B** without that bond.

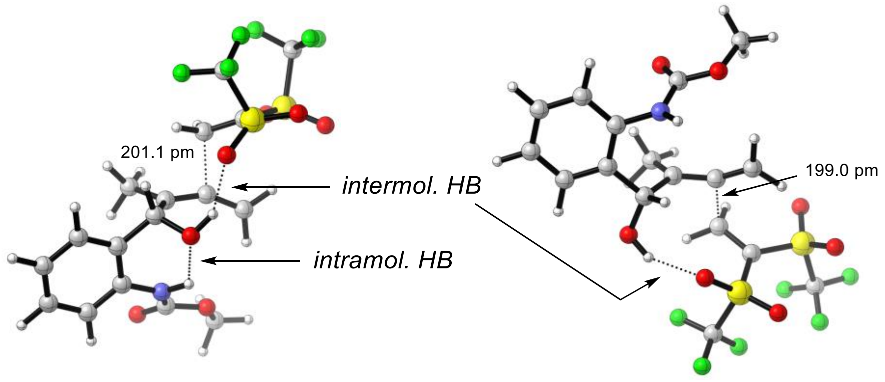

TS-1
+23.1 kcal mol^{-1}

TS-1B
+26.8 kcal mol^{-1}

Table S3. Coordinates and energies for optimized geometry of **TS-1**

Center	Atomic	Atomic	Coordinates (Angstroms)		
Number	Number	Type	X	Y	Z
1	6	0	-2.309275	-1.120020	-0.640771
2	6	0	-3.803883	-1.190775	-0.376461
3	6	0	-6.556784	-1.346678	0.100934
4	6	0	-4.411197	-2.424388	-0.141844
5	6	0	-4.590011	-0.032420	-0.378323
6	6	0	-5.959627	-0.110853	-0.139750
7	6	0	-5.781999	-2.504721	0.096102
8	8	0	-2.061852	-0.756465	-1.968191
9	6	0	-1.553707	-0.336908	0.402424
10	6	0	-0.538679	0.612363	0.085553
11	7	0	-3.980077	1.230585	-0.645831
12	6	0	-3.696457	2.122092	0.347047
13	8	0	-3.275969	3.283435	-0.171950
14	8	0	-3.762940	1.881283	1.540298
15	6	0	-2.882201	4.279863	0.781649
16	6	0	-1.850087	-0.696256	1.796607
17	6	0	-0.721681	1.505355	-0.916823
18	1	0	-1.902364	-2.130425	-0.443771
19	1	0	-7.624535	-1.402705	0.287666
20	1	0	-3.808306	-3.328365	-0.146737
21	1	0	-6.547546	0.802299	-0.144385
22	1	0	-6.241708	-3.471414	0.275670
23	1	0	-1.140769	-1.005306	-2.172987
24	1	0	-4.002421	1.584929	-1.596508
25	1	0	-2.044725	3.916714	1.380816
26	1	0	-3.722648	4.532293	1.430108
27	1	0	-2.582724	5.141920	0.189413
28	1	0	-1.028080	-0.510295	2.487083
29	1	0	-2.242089	-1.711114	1.898856
30	1	0	-2.667956	-0.003726	2.078320
31	1	0	-1.615313	1.517417	-1.525657
32	1	0	0.027264	2.278775	-1.081487
33	6	0	0.792732	0.615978	0.847311
34	1	0	1.010998	1.622704	1.210581
35	1	0	0.752733	-0.035582	1.721193
36	6	0	1.896659	0.196676	-0.097299
37	16	0	3.001230	1.349345	-0.669182
38	8	0	3.490592	1.104483	-2.020428
39	8	0	2.503125	2.677806	-0.299267
40	16	0	1.877476	-1.394635	-0.662982
41	8	0	2.829203	-1.675000	-1.729279
42	8	0	0.480889	-1.856249	-0.788057
43	6	0	2.475330	-2.459517	0.743799
44	9	0	2.428840	-3.739972	0.386445
45	9	0	3.724379	-2.144360	1.069213
46	9	0	1.697706	-2.287446	1.816946
47	6	0	4.534474	1.181445	0.364993
48	9	0	4.221370	1.228464	1.660605
49	9	0	5.137428	0.022852	0.110525
50	9	0	5.378656	2.174985	0.093086

E(RM062X) = -2633.86934653

Zero-point correction = 0.347112 (Hartree/Particle)

Sum of electronic and thermal Enthalpies = -2633.465202

Sum of electronic and thermal Free Energies = -2633.628432

Table S4. Coordinates and energies for optimized geometry of **INT-1**

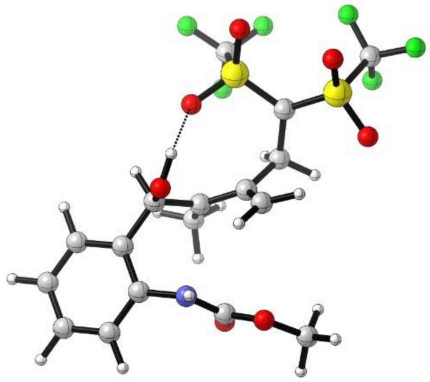

Center Number	Atomic Number	Atomic Type	Coordinates (Angstroms) X	Y	Z
1	6	0	-2.059333	-1.209811	-0.184784
2	6	0	-3.509570	-1.540231	0.100823
3	6	0	-6.185827	-2.060988	0.679818
4	6	0	-3.883566	-2.772348	0.629040
5	6	0	-4.489559	-0.569682	-0.158328
6	6	0	-5.827774	-0.830254	0.131145
7	6	0	-5.220947	-3.036234	0.924192
8	8	0	-1.921806	-0.960837	-1.563073
9	6	0	-1.614581	-0.022807	0.673197
10	6	0	-0.773733	0.893588	0.141269
11	7	0	-4.069868	0.660204	-0.716563
12	6	0	-4.495260	1.887212	-0.295934
13	8	0	-3.848717	2.850180	-0.977544
14	8	0	-5.320232	2.097215	0.572200
15	6	0	-4.192729	4.193616	-0.619087
16	6	0	-1.974178	-0.036656	2.120398
17	6	0	-0.527121	1.896695	-0.671482
18	1	0	-1.437975	-2.068663	0.122632
19	1	0	-7.229087	-2.259414	0.905871
20	1	0	-3.121986	-3.524250	0.819094
21	1	0	-6.576617	-0.072567	-0.070541
22	1	0	-5.505850	-3.996857	1.341458
23	1	0	-0.972887	-0.968838	-1.788389
24	1	0	-3.339580	0.627314	-1.424080
25	1	0	-3.970314	4.372010	0.434536
26	1	0	-5.251785	4.375509	-0.810650
27	1	0	-3.575705	4.827962	-1.252480
28	1	0	-1.464146	0.748114	2.679519
29	1	0	-1.767070	-1.018000	2.566618
30	1	0	-3.057584	0.120970	2.203280
31	1	0	-1.358755	2.468082	-1.079540
32	1	0	0.480288	2.205139	-0.936035
33	6	0	0.847290	0.087069	1.016906
34	1	0	0.843594	0.771169	1.862523
35	1	0	0.517182	-0.920363	1.249380
36	6	0	1.909429	0.208944	0.102240
37	16	0	2.866403	1.662544	0.095143
38	8	0	3.140147	2.172209	-1.238944
39	8	0	2.315334	2.529845	1.131603
40	16	0	2.118574	-1.038657	-1.072704
41	8	0	3.079115	-0.675694	-2.099612
42	8	0	0.804206	-1.595572	-1.405352

43	6	0	2.913383	-2.437235	-0.132613
44	9	0	3.122034	-3.447022	-0.965148
45	9	0	4.067888	-2.040351	0.385258
46	9	0	2.109078	-2.835233	0.850950
47	6	0	4.533007	1.160304	0.756797
48	9	0	4.389268	0.501438	1.900778
49	9	0	5.165037	0.392277	-0.119930
50	9	0	5.240001	2.262582	0.970083

E(RM062X) = -2633.88979271
Zero-point correction = 0.349937 (Hartree/Particle)
Sum of electronic and thermal Enthalpies = -2633.483115
Sum of electronic and thermal Free Energies = -2633.645754

Table S5. Coordinates and energies for optimised geometry of **TS-2**

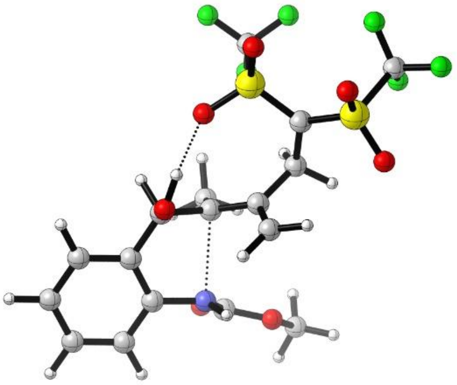

Center Number	Atomic Number	Atomic Type	Coordinates (Angstroms) X	Y	Z
1	6	0	-2.275148	-1.346768	-0.537928
2	6	0	-3.788865	-1.232547	-0.544499
3	6	0	-6.576662	-1.069514	-0.611554
4	6	0	-4.555764	-2.389252	-0.695496
5	6	0	-4.427965	0.002953	-0.444574
6	6	0	-5.819611	0.088410	-0.470196
7	6	0	-5.943956	-2.308823	-0.729763
8	8	0	-1.833027	-1.650308	-1.832703
9	6	0	-1.573740	-0.179774	0.121153
10	6	0	-0.534808	0.569718	-0.529103
11	7	0	-3.614635	1.175522	-0.328877
12	6	0	-3.773806	2.030334	0.753240
13	8	0	-3.168028	3.192567	0.504830
14	8	0	-4.328026	1.731239	1.787431
15	6	0	-3.220837	4.157352	1.567414
16	6	0	-1.741166	-0.111567	1.589751
17	6	0	-0.64050	0.916375	-1.829137
18	1	0	-2.017436	-2.176078	0.145995
19	1	0	-7.659932	-1.005393	-0.631020
20	1	0	-4.058685	-3.351607	-0.782168
21	1	0	-6.295886	1.060161	-0.379898
22	1	0	-6.533723	-3.213365	-0.840249
23	1	0	-0.909952	-1.957755	-1.755669
24	1	0	-3.378014	1.652097	-1.196112
25	1	0	-2.720240	3.765465	2.454413
26	1	0	-4.259291	4.397438	1.800458
27	1	0	-2.699108	5.032474	1.186575
28	1	0	-1.562551	0.886954	1.994062

29	1	0	-0.974867	-0.784385	2.010032
30	1	0	-2.717291	-0.487061	1.906794
31	1	0	-1.498101	0.649822	-2.436551
32	1	0	0.139831	1.519806	-2.290002
33	6	0	0.739190	0.923768	0.244233
34	1	0	0.959147	1.986459	0.121055
35	1	0	0.615043	0.751817	1.315358
36	6	0	1.907975	0.134532	-0.308470
37	16	0	3.082935	0.916986	-1.246004
38	8	0	3.668424	0.094348	-2.297944
39	8	0	2.592094	2.263737	-1.554833
40	16	0	1.895544	-1.536070	-0.086380
41	8	0	2.889290	-2.268131	-0.859808
42	8	0	0.497931	-2.014193	-0.051261
43	6	0	2.406847	-1.843638	1.677864
44	9	0	2.343582	-3.145165	1.944828
45	9	0	3.647595	-1.417469	1.885418
46	9	0	1.588057	-1.195458	2.514547
47	6	0	4.535583	1.259161	-0.138061
48	9	0	4.129208	1.851749	0.985811
49	9	0	5.157796	0.125696	0.176341
50	9	0	5.396871	2.064142	-0.757804

E(RM062X) = -2633.89208947
Zero-point correction = 0.350621 (Hartree/Particle)
Sum of electronic and thermal Enthalpies = -2633.486363
Sum of electronic and thermal Free Energies = -2633.643426

Table S6. Coordinates and energies for optimised geometry of **INT-2**

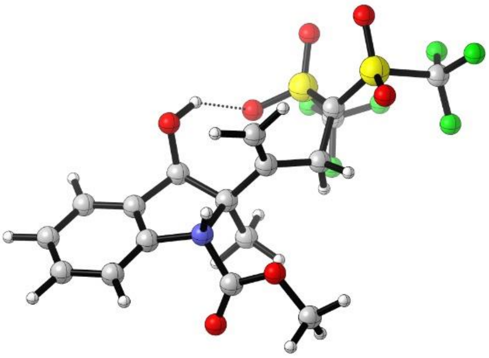

Center Number	Atomic Number	Atomic Type	Coordinates (Angstroms) X	Y	Z
1	6	0	-2.384984	-1.392219	-0.398299
2	6	0	-3.882915	-1.262309	-0.458902
3	6	0	-6.567803	-0.566978	-0.641437
4	6	0	-4.826416	-2.262049	-0.675237
5	6	0	-4.305333	0.047368	-0.342395
6	6	0	-5.630660	0.443166	-0.430566
7	6	0	-6.170665	-1.903309	-0.760313
8	8	0	-1.917574	-1.735271	-1.670679
9	6	0	-1.906175	0.011020	0.149593
10	6	0	-0.691944	0.603780	-0.550266
11	7	0	-3.151518	0.944699	-0.179563
12	6	0	-3.319727	2.023292	0.842811
13	8	0	-2.402649	2.925602	0.621019
14	8	0	-4.155809	1.967441	1.689718
15	6	0	-2.379851	4.039187	1.549796

16	6	0	-1.813406	-0.098764	1.667150
17	6	0	-0.743630	0.903248	-1.849974
18	1	0	-2.069745	-2.142713	0.335285
19	1	0	-7.619171	-0.308497	-0.712054
20	1	0	-4.514083	-3.296804	-0.777240
21	1	0	-5.931022	1.481277	-0.337653
22	1	0	-6.921532	-2.670036	-0.921735
23	1	0	-0.967644	-1.937999	-1.570722
24	1	0	-2.935725	1.417434	-1.068923
25	1	0	-2.201715	3.660641	2.556701
26	1	0	-3.331954	4.567133	1.496933
27	1	0	-1.559550	4.666696	1.213349
28	1	0	-1.546195	0.845276	2.150361
29	1	0	-1.039735	-0.832400	1.906875
30	1	0	-2.759312	-0.458176	2.084602
31	1	0	-1.615361	0.716025	-2.473918
32	1	0	0.124110	1.325991	-2.350504
33	6	0	0.603147	0.831044	0.226327
34	1	0	0.842948	1.898138	0.195911
35	1	0	0.483735	0.585044	1.282020
36	6	0	1.789009	0.081822	-0.352111
37	16	0	2.961723	0.905845	-1.247790
38	8	0	3.559204	0.131369	-2.330879
39	8	0	2.469642	2.263633	-1.505663
40	16	0	1.807517	-1.588431	-0.162907
41	8	0	2.805153	-2.293299	-0.959335
42	8	0	0.419346	-2.088071	-0.114146
43	6	0	2.347082	-1.934734	1.586791
44	9	0	2.296530	-3.243870	1.825488
45	9	0	3.590381	-1.508353	1.787134
46	9	0	1.541176	-1.313182	2.455388
47	6	0	4.417424	1.222969	-0.134788
48	9	0	4.012079	1.765323	1.015638
49	9	0	5.062447	0.089470	0.132278
50	9	0	5.263753	2.066280	-0.725989

E(RM062X) = -2633.91524207
Zero-point correction = 0.354310 (Hartree/Particle)
Sum of electronic and thermal Enthalpies = -2633.506162
Sum of electronic and thermal Free Energies = -2633.662014

Table S7. Coordinates and energies for optimised geometry of **INT-3**

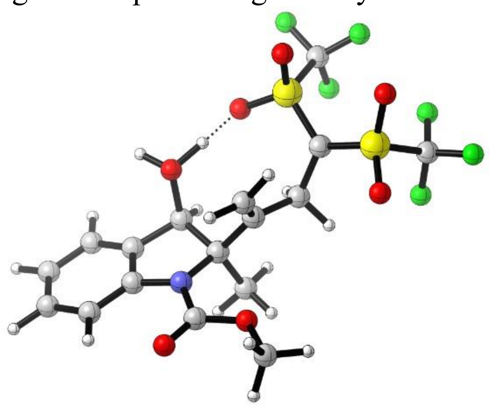

Center Number	Atomic Number	Atomic Type	Coordinates (Angstroms) X	Y	Z
1	6	0	-2.286680	-1.308261	0.629270

2	6	0	-3.762971	-1.471928	0.528520
3	6	0	-6.456988	-1.344882	0.011201
4	6	0	-4.541451	-2.592448	0.786974
5	6	0	-4.315735	-0.288805	0.026793
6	6	0	-5.681576	-0.211975	-0.246898
7	6	0	-5.909652	-2.522551	0.526220
8	8	0	-1.702200	-1.918391	-0.602660
9	6	0	-2.065918	0.230549	0.586351
10	6	0	-0.790594	0.550380	-0.181697
11	7	0	-3.313692	0.695879	-0.083539
12	6	0	-3.603122	2.014388	-0.367522
13	8	0	-2.595324	2.825011	-0.042496
14	8	0	-4.648192	2.387415	-0.865637
15	6	0	-2.769597	4.202442	-0.400504
16	6	0	-2.063178	0.737134	2.039506
17	6	0	-0.799945	0.854230	-1.477260
18	1	0	-1.795832	-1.787752	1.477815
19	1	0	-7.522807	-1.299100	-0.192340
20	1	0	-4.094241	-3.498398	1.187263
21	1	0	-6.124229	0.691774	-0.641032
22	1	0	-6.546117	-3.378615	0.723826
23	1	0	-0.623332	-1.980671	-0.591823
24	1	0	-3.633618	4.619769	0.119311
25	1	0	-2.904033	4.294277	-1.479716
26	1	0	-1.853577	4.697591	-0.084710
27	1	0	-1.937400	1.820842	2.054509
28	1	0	-1.261142	0.280105	2.623330
29	1	0	-3.016292	0.484927	2.515325
30	1	0	-1.727186	0.950673	-2.033475
31	1	0	0.124982	1.020549	-2.020544
32	6	0	0.494505	0.398994	0.624428
33	1	0	0.660211	1.314239	1.206569
34	1	0	0.387568	-0.398043	1.369456
35	6	0	1.722154	0.122438	-0.211566
36	16	0	2.806180	1.370038	-0.578251
37	8	0	3.777048	0.988404	-1.599093
38	8	0	2.104143	2.650654	-0.643691
39	16	0	1.925904	-1.416056	-0.842304
40	8	0	2.381942	-1.530722	-2.218724
41	8	0	0.723248	-2.215169	-0.421326
42	6	0	3.211086	-2.363957	0.118524
43	9	0	3.136914	-3.654234	-0.190513
44	9	0	4.420657	-1.910667	-0.183948
45	9	0	2.998976	-2.216371	1.423587
46	6	0	3.846593	1.573971	0.950330
47	9	0	3.069200	1.829149	2.005255
48	9	0	4.538674	0.463029	1.194077
49	9	0	4.696006	2.585068	0.788698
50	1	0	-2.081211	-2.806729	-0.761550

E(RM062X) = -2633.92514400

Zero-point correction = 0.351047 (Hartree/Particle)

Sum of electronic and thermal Enthalpies = -2633.519364

Sum of electronic and thermal Free Energies = -2633.674015

Table S8. Coordinates and energies for optimised geometry of **TS-3**

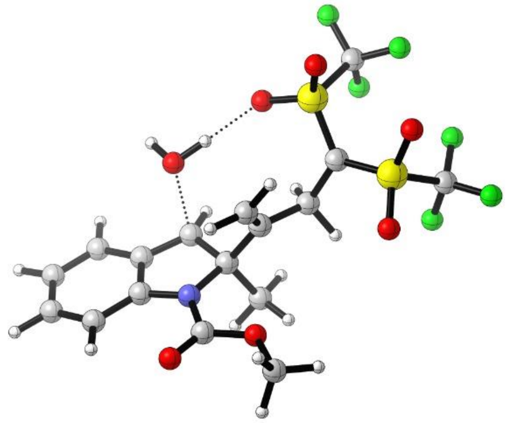

Center Number	Atomic Number	Atomic Type	Coordinates (Angstroms) X	Y	Z
1	6	0	-2.511921	-1.237669	0.968197
2	6	0	-3.881701	-1.400421	0.745600
3	6	0	-6.512406	-1.237099	-0.015365
4	6	0	-4.733445	-2.492871	1.029469
5	6	0	-4.358368	-0.238219	0.082123
6	6	0	-5.702947	-0.151267	-0.308877
7	6	0	-6.049866	-2.401238	0.647632
8	8	0	-1.765355	-2.355749	-0.685672
9	6	0	-2.118588	0.179992	0.647825
10	6	0	-0.845074	0.279697	-0.194219
11	7	0	-3.335393	0.678047	-0.035651
12	6	0	-3.518700	1.978468	-0.506836
13	8	0	-2.475029	2.742431	-0.220578
14	8	0	-4.512620	2.336209	-1.098759
15	6	0	-2.529611	4.086638	-0.727480
16	6	0	-1.992271	0.879560	2.031827
17	6	0	-0.893109	0.431787	-1.514392
18	1	0	-1.896018	-1.856547	1.609688
19	1	0	-7.556958	-1.191404	-0.309741
20	1	0	-4.341008	-3.371245	1.532778
21	1	0	-6.088481	0.720393	-0.817904
22	1	0	-6.741497	-3.212548	0.844991
23	1	0	-0.784614	-2.297971	-0.761062
24	1	0	-3.380988	4.611989	-0.292362
25	1	0	-2.613366	4.068486	-1.814991
26	1	0	-1.591941	4.543573	-0.419539
27	1	0	-1.688245	1.915581	1.875766
28	1	0	-1.252522	0.377537	2.657621
29	1	0	-2.958100	0.858735	2.543288
30	1	0	-1.831927	0.510934	-2.053703
31	1	0	0.022267	0.477944	-2.096084
32	6	0	0.446848	0.144561	0.596546
33	1	0	0.546596	1.015436	1.254869
34	1	0	0.382317	-0.721676	1.269258
35	6	0	1.696330	0.029694	-0.242639
36	16	0	2.634007	1.406584	-0.512661
37	8	0	3.677874	1.199326	-1.512733
38	8	0	1.793024	2.604406	-0.543877
39	16	0	2.097617	-1.465895	-0.909119
40	8	0	2.641961	-1.443088	-2.261854
41	8	0	0.990894	-2.393064	-0.593783

42	6	0	3.482341	-2.226528	0.076890
43	9	0	3.618928	-3.508140	-0.260815
44	9	0	4.628997	-1.597199	-0.159274
45	9	0	3.208857	-2.154891	1.380456
46	6	0	3.608943	1.674136	1.052034
47	9	0	2.786076	1.822642	2.095370
48	9	0	4.406872	0.635824	1.292610
49	9	0	4.356372	2.771208	0.941555
50	1	0	-1.963392	-3.290662	-0.505902

E(RM062X) = -2633.90612339
Zero-point correction = 0.349445 (Hartree/Particle)
Sum of electronic and thermal Enthalpies = -2633.500914
Sum of electronic and thermal Free Energies = -2633.659075

Table S9. Coordinates and energies for optimised geometry of H_2O

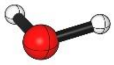

Center Number	Atomic Number	Atomic Type	Coordinates (Angstroms) X	Y	Z
1	8	0	0.000000	0.117737	0.000000
2	1	0	0.767958	-0.470846	0.000000
3	1	0	-0.767958	-0.471053	0.000000

E(RM062X) = -76.3914479629
Zero-point correction = 0.021361 (Hartree/Particle)
Sum of electronic and thermal Enthalpies = -76.364960
Sum of electronic and thermal Free Energies = -76.396393

Table S10. Coordinates and energies for optimised geometry of **INT-4’**

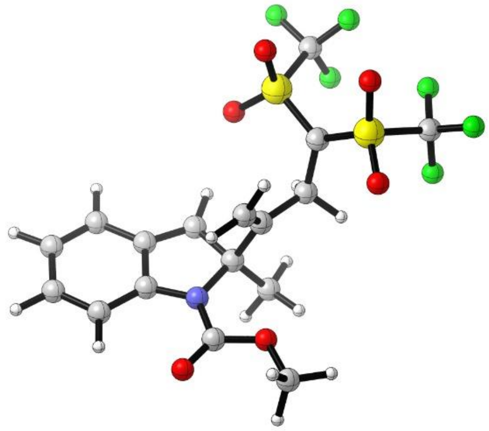

Center Number	Atomic Number	Atomic Type	Coordinates (Angstroms) X	Y	Z
1	6	0	-2.246697	-1.222033	0.961184
2	6	0	-3.438056	-1.648213	0.449563
3	6	0	-5.937321	-1.905719	-0.669193
4	6	0	-3.981959	-2.962643	0.293468
5	6	0	-4.174709	-0.482069	0.022705
6	6	0	-5.454256	-0.619234	-0.546786
7	6	0	-5.221988	-3.075846	-0.259539
8	6	0	-2.134506	0.261874	0.884859
9	6	0	-0.932162	0.511432	-0.046156
10	7	0	-3.426734	0.625567	0.265700
11	6	0	-3.825446	1.934548	-0.064551
12	8	0	-2.887999	2.801415	0.267776

13	8	0	-4.883218	2.186970	-0.588243
14	6	0	-3.165063	4.175310	-0.065205
15	6	0	-2.006386	0.883027	2.293291
16	6	0	-1.086974	0.696368	-1.355039
17	1	0	-1.439989	-1.842740	1.340777
18	1	0	-6.922096	-2.040390	-1.107264
19	1	0	-3.405763	-3.824211	0.613662
20	1	0	-6.025608	0.237397	-0.872899
21	1	0	-5.681915	-4.047386	-0.399227
22	1	0	-4.055624	4.510100	0.468105
23	1	0	-3.309568	4.268500	-1.142114
24	1	0	-2.285639	4.726936	0.257546
25	1	0	-1.819582	1.953519	2.210047
26	1	0	-1.188872	0.415064	2.842693
27	1	0	-2.934915	0.715331	2.845005
28	1	0	-2.061064	0.742122	-1.832974
29	1	0	-0.218093	0.812778	-1.995145
30	6	0	0.420280	0.444309	0.647384
31	1	0	0.625760	1.422614	1.099427
32	1	0	0.378045	-0.261744	1.483731
33	6	0	1.566255	0.054228	-0.253577
34	16	0	2.582718	1.241919	-0.875079
35	8	0	3.475061	0.752155	-1.923079
36	8	0	1.847650	2.498146	-1.047973
37	16	0	1.720533	-1.585789	-0.635628
38	8	0	2.161231	-1.881390	-1.995290
39	8	0	0.541990	-2.270338	-0.084012
40	6	0	3.097281	-2.299801	0.394940
41	9	0	3.067195	-3.631397	0.327748
42	9	0	4.282746	-1.878038	-0.036509
43	9	0	2.950327	-1.936979	1.671503
44	6	0	3.758825	1.696910	0.495924
45	9	0	3.077017	2.116329	1.567139
46	9	0	4.499951	0.650599	0.856027
47	9	0	4.569386	2.677787	0.099312

E(RM062X) = -2557.50813009
Zero-point correction = 0.324269 (Hartree/Particle)
Sum of electronic and thermal Enthalpies = -2557.130885
Sum of electronic and thermal Free Energies = -2557.284051

Table S11. Coordinates and energies for optimised geometry of **INT-4**

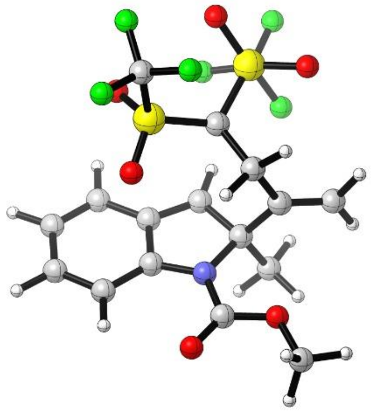

Center Number	Atomic Number	Atomic Type	Coordinates (Angstroms) X	Y	Z
1	6	0	0.950884	-1.808960	-0.710705

2	6	0	1.781672	-1.296203	0.469288
3	6	0	2.448626	0.911962	0.920747
4	6	0	3.094554	2.157338	0.996527
5	1	0	3.914220	2.413343	0.341324
6	6	0	2.615661	3.043843	1.941147
7	1	0	3.091905	4.017496	2.015526
8	6	0	1.528421	2.762838	2.821816
9	1	0	1.208948	3.523806	3.524923
10	6	0	0.900925	1.551751	2.769712
11	1	0	0.062299	1.301474	3.410855
12	6	0	1.368328	0.600722	1.814968
13	6	0	0.925787	-0.661197	1.515577
14	1	0	0.162217	-1.217360	2.044601
15	6	0	-0.986531	-0.231282	-0.505735
16	6	0	0.105992	-0.767647	-1.409593
17	1	0	-0.313692	-1.208738	-2.318555
18	1	0	0.728870	0.081116	-1.713732
19	6	0	0.906211	-3.095293	-1.048186
20	1	0	0.252675	-3.415908	-1.854429
21	1	0	1.489782	-3.863578	-0.552521
22	6	0	2.613281	-2.392951	1.175869
23	1	0	1.948965	-3.155612	1.587135
24	1	0	3.191264	-1.948459	1.989729
25	1	0	3.293836	-2.850389	0.455582
26	6	0	3.683584	-0.202970	-0.864391
27	6	0	4.701548	-1.541603	-2.486588
28	1	0	4.548247	-0.796352	-3.267839
29	1	0	4.553343	-2.546632	-2.874490
30	1	0	5.696422	-1.433579	-2.052465
31	6	0	-2.428597	-1.959702	1.284059
32	6	0	-2.213305	2.221757	-1.553707
33	9	0	-3.567908	-2.623771	1.452836
34	9	0	-1.413011	-2.822794	1.381874
35	9	0	-2.305191	-1.056991	2.256496
36	9	0	-2.251205	1.423891	-2.623932
37	9	0	-1.695702	3.393814	-1.919272
38	9	0	-3.447699	2.425110	-1.118763
39	7	0	2.672384	-0.158536	0.103817
40	8	0	4.418356	0.726074	-1.102981
41	8	0	3.704721	-1.384123	-1.460964
42	8	0	-2.385976	-2.279091	-1.298863
43	8	0	-3.611344	-0.281736	-0.311906
44	8	0	0.196547	2.044050	-0.519885
45	8	0	-1.792505	1.775890	1.024955
46	16	0	-2.427701	-1.138289	-0.384327
47	16	0	-1.111684	1.450342	-0.225247

E(RM062X) = -2557.51805172

Zero-point correction = 0.324260 (Hartree/Particle)

Sum of electronic and thermal Enthalpies = -2557.141138

Sum of electronic and thermal Free Energies = -2557.291563

Table S12. Coordinates and energies for optimised geometry of **TS-4**

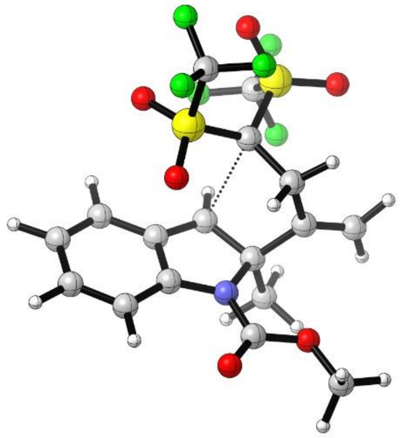

Center Number	Atomic Number	Atomic Type	Coordinates (Angstroms) X	Y	Z
1	6	0	-0.964217	-1.801613	0.749638
2	6	0	-1.755979	-1.274760	-0.447224
3	6	0	-2.431662	0.930975	-0.915940
4	6	0	-3.094366	2.164053	-1.013162
5	1	0	-3.931664	2.410673	-0.376710
6	6	0	-2.611102	3.054984	-1.954719
7	1	0	-3.101653	4.020185	-2.044433
8	6	0	-1.505664	2.783412	-2.808786
9	1	0	-1.182678	3.540764	-3.514289
10	6	0	-0.858403	1.580649	-2.729009
11	1	0	-0.001070	1.339614	-3.348592
12	6	0	-1.328131	0.634324	-1.777309
13	6	0	-0.859778	-0.621092	-1.451335
14	1	0	-0.112248	-1.181313	-1.997179
15	6	0	0.944613	-0.234868	0.461269
16	6	0	-0.101390	-0.761208	1.424380
17	1	0	0.353420	-1.195735	2.319328
18	1	0	-0.716892	0.088296	1.741864
19	6	0	-0.954482	-3.082772	1.106788
20	1	0	-0.317788	-3.410056	1.923747
21	1	0	-1.556359	-3.840868	0.616919
22	6	0	-2.558271	-2.361695	-1.199342
23	1	0	-1.879811	-3.124003	-1.587906
24	1	0	-3.100546	-1.908116	-2.032624
25	1	0	-3.271105	-2.822041	-0.512659
26	6	0	-3.699867	-0.201970	0.836095
27	6	0	-4.762289	-1.558499	2.415823
28	1	0	-4.629021	-0.823411	3.210430
29	1	0	-4.626568	-2.568494	2.795686
30	1	0	-5.745617	-1.443138	1.957808
31	6	0	2.415146	-1.952926	-1.317907
32	6	0	2.238994	2.158955	1.591840
33	9	0	3.559251	-2.607180	-1.482439
34	9	0	1.405551	-2.821767	-1.415401
35	9	0	2.288046	-1.047716	-2.286638
36	9	0	2.333094	1.282541	2.594313
37	9	0	1.705087	3.283584	2.063980
38	9	0	3.449202	2.427720	1.128621
39	7	0	-2.662745	-0.144121	-0.097476
40	8	0	-4.445935	0.721886	1.065044
41	8	0	-3.738557	-1.391043	1.419893

42	8	0	2.368065	-2.275593	1.267479
43	8	0	3.580136	-0.266799	0.289791
44	8	0	-0.187062	2.070604	0.562203
45	8	0	1.785061	1.803909	-1.005388
46	16	0	2.406721	-1.137283	0.351615
47	16	0	1.107289	1.464311	0.241524

E(RM062X) = -2557.51794717
Zero-point correction = 0.323919 (Hartree/Particle)
Sum of electronic and thermal Enthalpies = -2557.142503
Sum of electronic and thermal Free Energies = -2557.289077

Table S13. Coordinates and energies for optimised geometry of **P**

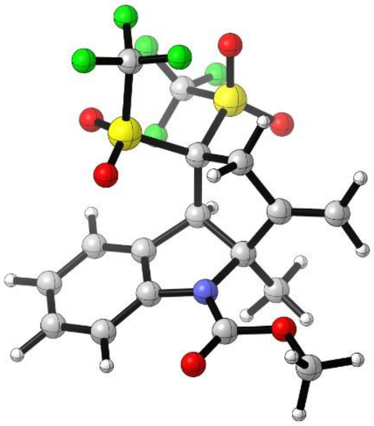

Center Number	Atomic Number	Atomic Type	Coordinates (Angstroms) X	Y	Z
1	6	0	-1.301664	-1.999425	0.313216
2	6	0	-1.770123	-0.987564	-0.709575
3	6	0	-2.267281	1.339531	-0.566866
4	6	0	-2.952471	2.550684	-0.503548
5	1	0	-3.918409	2.623850	-0.023804
6	6	0	-2.348511	3.667674	-1.087659
7	1	0	-2.869524	4.620015	-1.049479
8	6	0	-1.110325	3.586111	-1.723993
9	1	0	-0.672486	4.467996	-2.180387
10	6	0	-0.430014	2.367271	-1.769228
11	1	0	0.543083	2.293939	-2.244069
12	6	0	-1.009261	1.257847	-1.171316
13	6	0	-0.490994	-0.151695	-1.044355
14	1	0	-0.060140	-0.510873	-1.981374
15	6	0	0.530701	-0.454380	0.130421
16	6	0	-0.224983	-1.356076	1.142012
17	1	0	0.421903	-2.070244	1.657263
18	1	0	-0.672273	-0.695230	1.896303
19	6	0	-1.679138	-3.268609	0.403701
20	1	0	-1.220604	-3.941745	1.122649
21	1	0	-2.463097	-3.671162	-0.230743
22	6	0	-2.390899	-1.571759	-1.972489
23	1	0	-1.693493	-2.274653	-2.437488
24	1	0	-2.612389	-0.767648	-2.681899
25	1	0	-3.319869	-2.095545	-1.737642
26	6	0	-3.877672	-0.185743	0.471542
27	6	0	-5.369062	-1.847364	1.180920
28	1	0	-5.351030	-1.505557	2.217329
29	1	0	-5.414805	-2.934119	1.137103

30	1	0	-6.220179	-1.405180	0.660075
31	6	0	2.936938	-0.449924	-1.740164
32	6	0	2.289742	0.605954	2.375585
33	9	0	3.919871	0.175445	-1.127526
34	9	0	3.433824	-1.321296	-2.604228
35	9	0	2.162118	0.413077	-2.383276
36	9	0	3.533342	0.491296	1.956377
37	9	0	1.892043	-0.507482	2.972175
38	9	0	2.206239	1.615460	3.230817
39	7	0	-2.662223	0.059520	-0.118936
40	8	0	-4.619616	0.677340	0.903239
41	8	0	-4.147551	-1.494782	0.522027
42	8	0	1.348752	-2.540957	-1.287103
43	8	0	2.831925	-1.730882	0.613612
44	8	0	0.006264	1.701025	1.600559
45	8	0	2.000708	1.789632	0.003784
46	16	0	1.934207	-1.468610	-0.500300
47	16	0	1.144430	1.082661	0.943518

E(RM062X) = -2557.56627582
Zero-point correction = 0.326298 (Hartree/Particle)
Sum of electronic and thermal Enthalpies = -2557.188537
Sum of electronic and thermal Free Energies = -2557.332811

We also examined a possibility of dehydration reaction from starting allenol **2a** under the same level of DFT calculation. This process is highly up-hill process with +48.3 kcal mol^{-1} of activation energy (vs Gibbs energy of **2a** at 403 K). Therefore, we have concluded that the quinone imine forming process is unlikely under the reaction conditions.

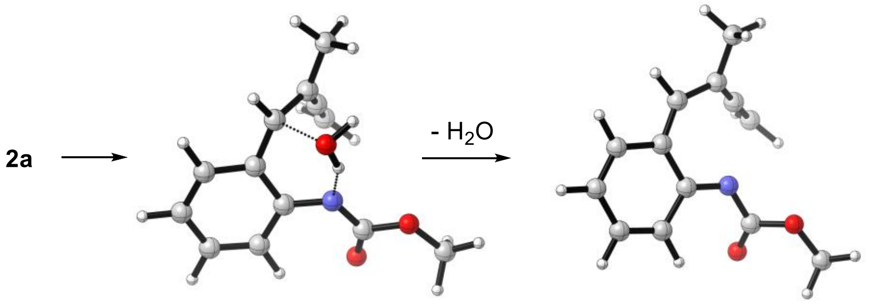

TS-5 +48.3 kcal mol^{-1} ***QI*** +18.0 kcal mol^{-1}

Table S14. Coordinates and energies for optimized geometry of **TS-5**

Center Number	Atomic Number	Atomic Type	Coordinates (Angstroms) X	Y	Z
1	6	0	1.556655	1.333377	-0.039824
2	6	0	1.630517	-0.089687	0.026273
3	6	0	1.923998	-2.807652	0.487429
4	6	0	2.922343	-0.656270	0.147887
5	6	0	0.461836	-0.926827	-0.019003
6	6	0	0.653686	-2.302922	0.278648
7	6	0	3.077307	-2.001107	0.404910
8	8	0	0.583851	1.494729	-1.921062
9	6	0	0.673456	2.201136	0.726512
10	6	0	-0.059000	1.667534	1.691022
11	7	0	-0.673586	-0.345549	-0.487575
12	6	0	-1.877543	-0.941130	-0.369258

13	8	0	-2.801670	-0.259871	-1.107355
14	8	0	-2.210085	-1.920198	0.298839
15	6	0	-4.129416	-0.769349	-1.048876
16	6	0	0.728435	3.691653	0.478176
17	6	0	-0.758706	1.125938	2.642143
18	1	0	2.457682	1.831459	-0.399993
19	1	0	2.035545	-3.869731	0.691804
20	1	0	3.788638	-0.000926	0.096083
21	1	0	-0.207497	-2.957368	0.299313
22	1	0	4.063247	-2.431819	0.541991
23	1	0	0.142682	2.343782	-2.080245
24	1	0	-0.121177	0.867334	-1.543644
25	1	0	-4.512569	-0.737397	-0.025662
26	1	0	-4.166940	-1.799146	-1.412978
27	1	0	-4.723649	-0.121250	-1.692683
28	1	0	0.195045	4.233492	1.260523
29	1	0	0.272338	3.947643	-0.483674
30	1	0	1.767065	4.036852	0.457178
31	1	0	-0.349653	0.986240	3.639994
32	1	0	-1.774842	0.785884	2.448024

E(RM062X) = -784.424967548
Zero-point correction = 0.256863 (Hartree/Particle)
Sum of electronic and thermal Enthalpies = -784.137571
Sum of electronic and thermal Free Energies = -784.237093

Table S15. Coordinates and energies for optimized geometry of **QI**

Center Number	Atomic Number	Atomic Type	Coordinates (Angstroms) X	Y	Z
1	6	0	1.725220	1.317559	0.513683
2	6	0	1.686414	-0.029591	0.264275
3	6	0	1.904590	-2.828546	-0.171791
4	6	0	2.979284	-0.687344	0.118324
5	6	0	0.466834	-0.855528	0.089172
6	6	0	0.664970	-2.289625	-0.090102
7	6	0	3.096562	-2.018190	-0.081177
8	6	0	0.717502	2.325159	0.822523
9	6	0	-0.358621	2.056210	1.540638
10	7	0	-0.694080	-0.274373	0.032540
11	6	0	-1.869774	-0.988315	0.018407
12	8	0	-2.718944	-0.472322	-0.888235
13	8	0	-2.185018	-1.899005	0.768234
14	6	0	-4.017414	-1.070905	-0.925451
15	6	0	1.048802	3.748905	0.416986
16	6	0	-1.407030	1.819755	2.274691
17	1	0	2.728463	1.748199	0.477679
18	1	0	2.010091	-3.899604	-0.319841
19	1	0	3.866034	-0.064043	0.194694
20	1	0	-0.215803	-2.915103	-0.168639
21	1	0	4.070874	-2.484823	-0.172901
22	1	0	-4.521025	-0.941373	0.034670
23	1	0	-3.941068	-2.135532	-1.154708
24	1	0	-4.557482	-0.549614	-1.713321
25	1	0	0.281373	4.440562	0.766265
26	1	0	1.121823	3.827539	-0.671730
27	1	0	2.012196	4.050554	0.841062
28	1	0	-1.360697	1.855065	3.359746
29	1	0	-2.354130	1.555532	1.807477

E(RM062X) = -708.052003531
Zero-point correction = 0.231196 (Hartree/Particle)
Sum of electronic and thermal Enthalpies = -707.792581
Sum of electronic and thermal Free Energies = -707.889049

Single crystal X-ray diffraction analysis

Single crystals were obtained by recrystallization through slow evaporation from a mixture of hexane and ethyl acetate (**3a**, **3e** and **7d**) or vapor diffusion of hexane in a chloroform solution at room temperature (**7g**). A suitable crystal with dimensions 0.16 × 0.15 × 0.07 mm^3 was mounted on a MiTeGen holder in perfluoro-polyether oil on a Bruker SMART APEX II CCD detector diffractometer. The crystal was kept at a steady 90 K during data collection. The structure was solved with *ShelXT 2014/5* solution program[iii] using a dual method and by using *Olex2*[iv] as the graphical interface. The model was refined with *XL*[v] using full matrix least squares minimization on F^2.

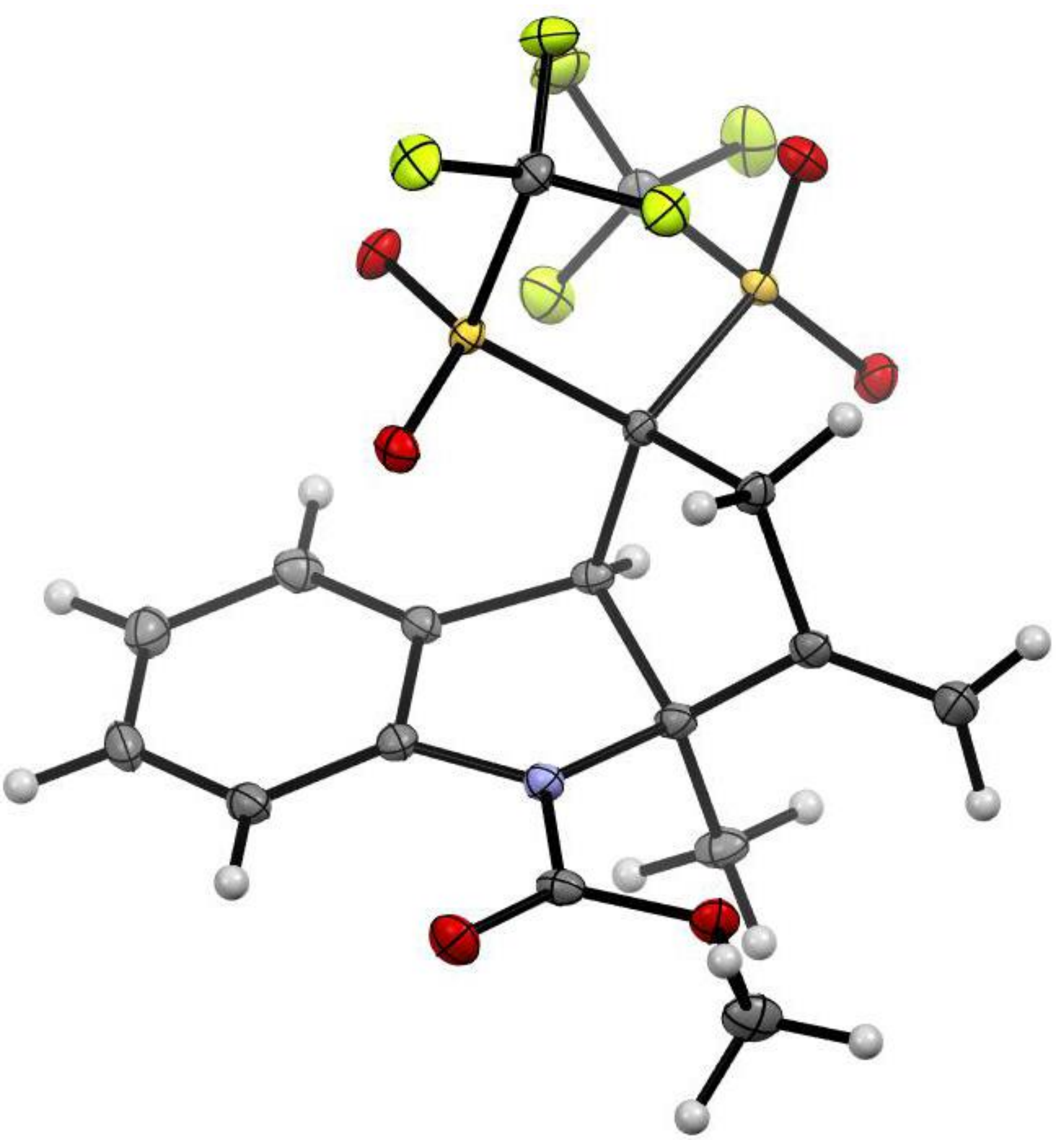

Figure S2. ORTEP drawing of bis(triflyl)-decorated tricyclic indoline **3a**. Thermal ellipsoids shown at 50% probability.

Table S16. Crystal data of **3a**

Formula	$C_{17}H_{15}F_6NO_6S_2$	Z	4
$D_{calc.}$/ g cm^{-3}	1.705	Z'	1
μ/mm^{-1}	0.363	Wavelength/Å	0.71073
Formula Weight	507.42	Radiation type	MoK_{α}
Color	colorless	Θ_{min}/°	1.889
Shape	block	Θ_{max}/°	30.539
Size/mm^3	0.16×0.15×0.07	Measured Refl's.	27377
T/K	90	Indep't Refl's	6023
Crystal System	orthorhombic	Refl's I≥2 σ(I)	5578
Flack Parameter	0.01(3)	R_{int}	0.0491
Hooft Parameter	0.01(3)	Parameters	291
Space Group	$Pna2_1$	Restraints	525
a/Å	18.6410(7)	Largest Peak	0.369
b/Å	13.2144(5)	Deepest Hole	-0.288
c/Å	8.0254(3)	GooF	1.035
α/°	90	wR_2 (all data)	0.0709
β/°	90	wR_2	0.0689
γ/°	90	R_1 (all data)	0.0340
V/Å^3	1976.89(13)	R_1	0.0300

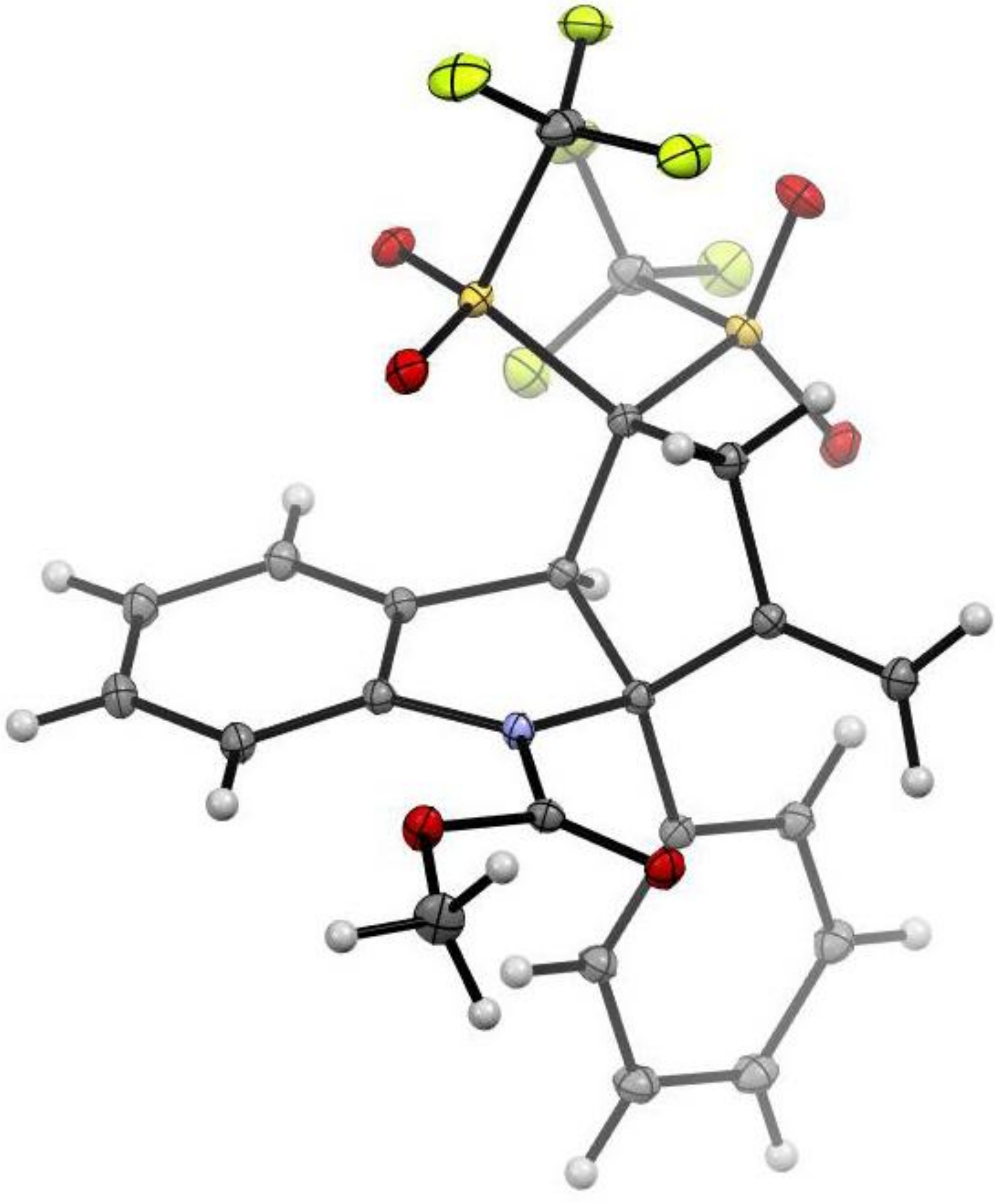

Figure S3. ORTEP drawing of bis(triflyl)-decorated tricyclic indoline **3e**. Thermal ellipsoids shown at 50% probability.

Table S17. Crystal data of **3e**

Formula	$C_{22}H_{17}F_6NO_6S_2$	Z	4
$D_{calc.}$/ g cm^{-3}	1.707	Z'	1
μ/mm^{-1}	0.334	Wavelength/Å	0.71073
Formula Weight	569.48	Radiation type	MoK$_\alpha$
Color	colorless	Θ_{min}/°	2.000
Shape	block	Θ_{max}/°	30.552
Size/mm^3	0.31×0.26×0.11	Measured Refl's.	54497
T/K	90	Indep't Refl's	6790
Crystal System	monoclinic	Refl's I≥2 σ(I)	6249
Space Group	$P2_1/c$	R_{int}	0.0330
a/Å	10.9273(2)	Parameters	335
b/Å	20.3695(4)	Restraints	462
c/Å	10.7249(2)	Largest Peak	0.487
α/°	90	Deepest Hole	-0.395
β/°	111.8660(10)	GooF	1.024
γ/°	90	wR_2 (all data)	0.0795
V/Å^3	2215.45(7)	wR_2	0.0774
		R_1 (all data)	0.0312
		R_1	0.0285

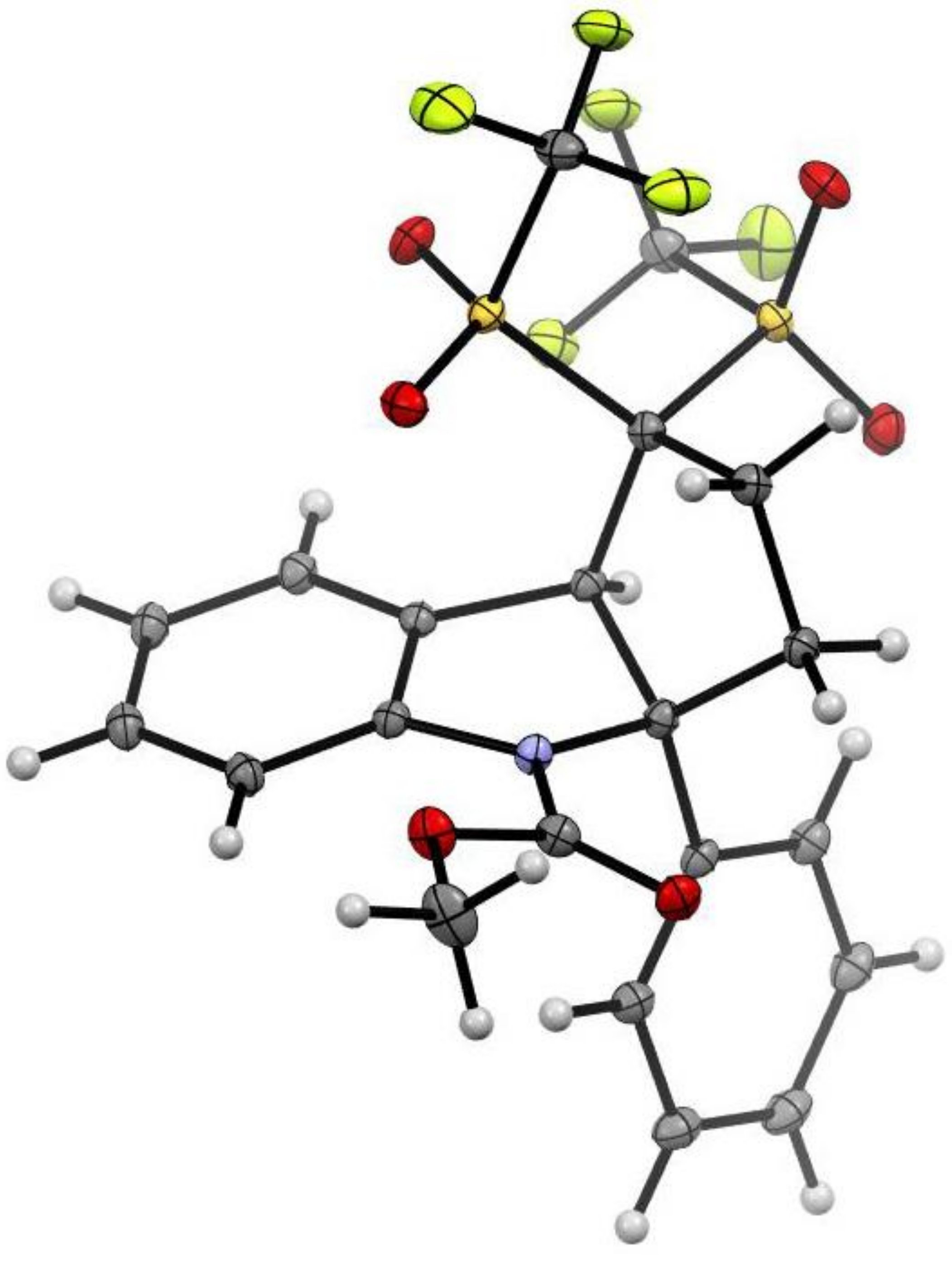

Figure S4. ORTEP drawing of bis(triflyl)-decorated tricyclic indoline **7d**. Thermal ellipsoids shown at 50% probability.

Table S18. Crystal data of **7d**

Formula	$C_{21}H_{17}F_6NO_6S_2$	*Z*	2
$D_{calc.}$/ g cm^{-3}	1.689	*Z'*	1
μ/mm^{-1}	0.336	Wavelength/Å	0.71073
Formula Weight	557.48	Radiation type	MoK_{α}
Color	colorless	Θ_{min}/°	2.055
Shape	block	Θ_{max}/°	30.552
Size/mm^3	0.31×0.27×0.12	Measured Refl's.	32214
T/K	89.95	Indep't Refl's	6720
Crystal System	monoclinic	Refl's I≥2 σ(I)	6573
Flack Parameter	-0.003(13)	R_{int}	0.0256
Hooft Parameter	-0.002(13)	Parameters	326
Space Group	$P2_1$	Restraints	451
a/Å	9.8309(2)	Largest Peak	0.365
b/Å	11.2489(2)	Deepest Hole	-0.218
c/Å	10.0300(2)	GooF	1.032
α/°	90	wR_2 (all data)	0.0614
β/°	98.8630(10)	wR_2	0.0609
γ/°	90	R_1 (all data)	0.0236
V/Å^3	1095.94(4)	R_1	0.0230

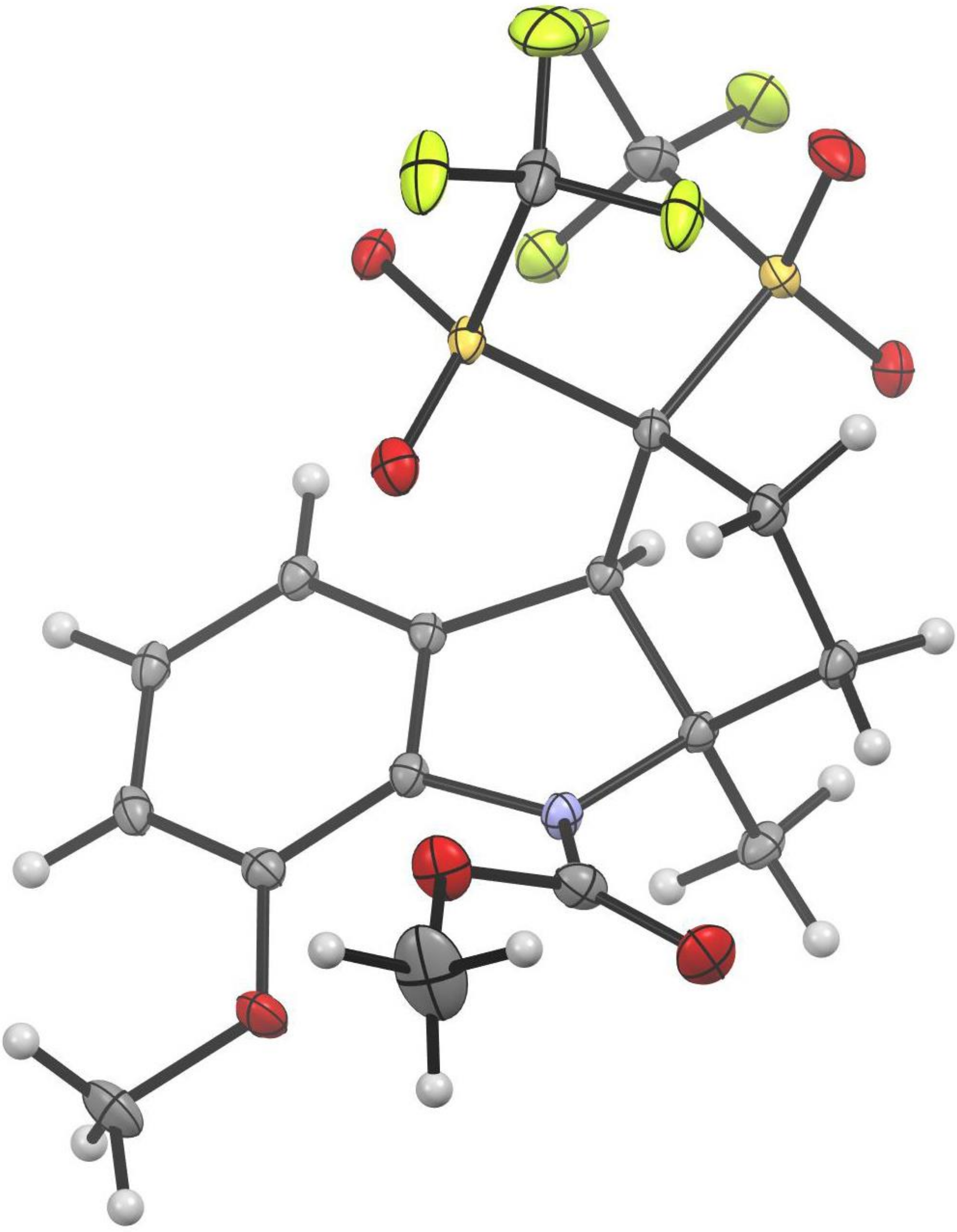

Figure S5. ORTEP drawing of bis(triflyl)-decorated tricyclic indoline **7g**. Thermal ellipsoids shown at 50% probability.

Table S19. Crystal data of **7g**

Formula	$C_{17}H_{17}F_6NO_7S_2$	Z	8
$D_{calc.}$/ g cm^{-3}	1.673	Z'	2
μ/mm^{-1}	0.350	Wavelength/Å	0.71073
Formula Weight	525.43	Radiation type	MoK_{α}
Color	colorless	Θ_{min}/°	1.231
Shape	block	Θ_{max}/°	30.538
Size/mm^3	0.37×0.23×0.16	Measured Refl's.	101376
T/K	90	Indep't Refl's	12771
Crystal System	monoclinic	Refl's I≥2 σ(I)	11582
Space Group	$P2_1/c$	R_{int}	0.0319
a/Å	7.4454(2)	Parameters	601
b/Å	21.2261(5)	Restraints	396
c/Å	26.5606(6)	Largest Peak	0.518
α/°	90	Deepest Hole	-0.402
β/°	96.2620(10)	GooF	1.043
γ/°	90	wR_2 (all data)	0.0804
V/Å^3	4172.51(18)	wR_2	0.0779
		R_1 (all data)	0.0328
		R_1	0.0291

References

[i]. For *Gaussian 09*, Revision D.01, M. J. Frisch, G. W. Trucks, H. B. Schlegel, G. E. Scuseria, M. A. Robb, J. R. Cheeseman, G. Scalmani, V. Barone, B. Mennucci, G. A. Petersson, H. Nakatsuji, M. Caricato, X. Li, H. P. Hratchian, A. F. Izmaylov, J. Bloino, G. Zheng, J. L. Sonnenberg, M. Hada, M. Ehara, K. Toyota, R. Fukuda, J. Hasegawa, M. Ishida, T. Nakajima, Y. Honda, O. Kitao, H. Nakai, T. Vreven, J. A. Montgomery, Jr., J. E. Peralta, F. Ogliaro, M. Bearpark, J. J. Heyd, E. Brothers, K. N. Kudin, V. N. Staroverov, T. Keith, R. Kobayashi, J. Normand, K. Raghavachari, A. Rendell, J. C. Burant, S. S. Iyengar, J. Tomasi, M. Cossi, N. Rega, J. M. Millam, M. Klene, J. E. Knox, J. B. Cross, V. Bakken, C. Adamo, J. Jaramillo, R. Gomperts, R. E. Stratmann, O. Yazyev, A. J. Austin, R. Cammi, C. Pomelli, J. W. Ochterski, R. L. Martin, K. Morokuma, V. G. Zakrzewski, G. A. Voth, P. Salvador, J. J. Dannenberg, S. Dapprich, A. D. Daniels, O. Farkas, J. B. Foresman, J. V. Ortiz, J. Cioslowski, D. J. Fox, Gaussian, Inc., Wallingford CT, 2013.

[ii]. Y. Zhao, D. G. Truhlar, *Theor. Chem. Acc.* **2008**, *120*: 215.

[iii]. G. M. Sheldrick, *Acta Cryst.* **2015**, *A71*, 3.

[iv]. O. V. Dolomanov, L. J. Bourhis, R. J. Gildea, J. A. K. Howard, and H. Puschmann, *J. Appl. Cryst.* **2009**, *42*, 339.

[v]. G. M. Sheldrick, *Acta Cryst.* **2008**, *A64*, 339.
